# Weight Gain in Survivors Living in Temporary Housing in the Tsunami-Stricken Area during the Recovery Phase following the Great East Japan Earthquake and Tsunami

**DOI:** 10.1371/journal.pone.0166817

**Published:** 2016-12-01

**Authors:** Shuko Takahashi, Yuki Yonekura, Ryohei Sasaki, Yukari Yokoyama, Kozo Tanno, Kiyomi Sakata, Akira Ogawa, Seichiro Kobayashi, Taro Yamamoto

**Affiliations:** 1 Department of International Health, Nagasaki University, Graduate School of Biomedical Sciences, Sakamoto, Nagasaki, Japan; 2 Department of Hygiene and Preventive Medicine, School of Medicine, Iwate Medical University, Nishitokuta, Yahaba-cho, Shiwa-Gun, Iwate, Japan; 3 Iwate Tohoku Medical Megabank Organization, Nishitokuta, Yahaba-cho, Shiwa-gun, Iwate, Japan; 4 Department of Social Welfare, Nihon Fukushi University, Egemae, Okuda, Mihama-cho, Chita-gun, Aichi, Japan; 5 Iwate Medical University, Iwate, Japan; 6 Department of Plastic and Reconstructive Surgery, School of Medicine, Iwate Medical University, Iwate, Japan; 7 Department of International Health, Institute of Tropical Medicine, Nagasaki University, Sakamoto, Nagasaki, Japan; Mexican Social Security Institute, MEXICO

## Abstract

**Introduction:**

Survivors who lost their homes in the Great East Japan Earthquake and Tsunami were forced to live in difficult conditions in temporary housing several months after the disaster. Body weights of survivors living in temporary housing for a long period might increase due to changes in their life style and psychosocial state during the medium-term and long-term recovery phases. The aim of this study was to determine whether there were differences between body weight changes of people living in temporary housing and those not living in temporary housing in a tsunami-stricken area during the medium-term and long-term recovery phases.

**Materials and methods:**

Health check-ups were performed about 7 months after the disaster (in 2011) and about 18 months after the disaster (in 2012) for people living in a tsunami-stricken area (n = 6,601, mean age = 62.3 y). We compared the changes in body weight in people living in temporary housing (TH group, n = 2,002) and those not living in temporary housing (NTH group, n = 4,599) using a multiple linear regression model.

**Results:**

While there was no significant difference between body weights in the TH and NTH groups in the 2011 survey, there was a significant difference between the mean changes in body weight in both sexes. We found that the changes in body weight were significantly greater in the TH group than in the NTH group in both sexes. The partial regression coefficients of mean change in body weight were +0.52 kg (P-value < 0.001) in males in the TH group and +0.56 kg (P-value < 0.001) in females in the TH group (reference: NTH group).

**Conclusion:**

Analysis after adjustment for life style, psychosocial factors and cardiovascular risk factors found that people living in temporary housing in the tsunami- stricken area had a significant increase in body weight.

## Introduction

On March 11, 2011, a massive earthquake with a magnitude of 9.0 on the Richter scale and a subsequent tsunami struck the northeast coast of Japan (Great East Japan Earthquake and Tsunami). A total of 18,490 people died or were missing after the tsunami, and the homes of about 470,000 evacuees were greatly damaged [1–4]. The estimated total financial damage was more than 160 billion dollars [5]. The tsunami destroyed commercial and public facilities including gymnasiums and parks. Many of the survivors lost family members and their homes.

After the disaster, most of the survivors did not move to inland towns but stayed in coastal towns that had been destroyed by the tsunami. The homes of some of the survivors were completely destroyed by the tsunami, but some houses remained in fact. Survivors who lost their homes were forced to live in inconvenient conditions; they lived in shelters just after the disaster, and later moved to temporary housing areas on hillsides of the coastal towns. Since large areas of the coastal towns were destroyed by the tsunami, the survivors could not live in the original seaside areas. Many of the survivors could not buy land to build a new house because of their difficult economic situation. In Iwate Prefecture, one of the prefectures damaged by the Great East Japan Earthquake and Tsunami, 19,584 people (1.5% of the prefecture’s population) were still living in temporary housing as of July 2015 [6].

Obesity and weight gain are important risk factors for mortality and morbidity from vascular diseases such as cardiovascular disease, diabetes mellitus, and hypertension [7–9]. Previous studies reported that body weights of survivors of earthquakes or hurricanes increased after the disaster [10–12]. In the Irpinia earthquake, the average weight of survivors had increased by 1.5kg two months after the disaster [10]. The mean body weight of survivors of the Great East Japan Earthquake living in the Soma area of Fukushima prefecture had increased by 0.6kg six months after the disaster [11]. Body mass indexes of displaced survivors of Hurricane Katrina increased from 7 to 19 months after the disaster [12]. Although it has been reported that body weights of survivors of disasters increased in the short term after a disaster, there have been few studies on body weight changes in survivors living in temporary housing a long time after a disaster, changes in body weights depending on living conditions in the affected area were not assessed in previous studies. The subjects of our study lived in shelters a few months after the earthquake. Body weights may have started to increase at that time. They were also forced to live in inconvenient living conditions in temporary housing in affected areas. Most of the temporary houses were container houses made from tinplate. The temporary houses had water and electricity, but they were small, had no soundproofing, and were not sufficiently cold-resistant. Photographs of the temporary houses are shown in S1A and S1B Fig. Residents in temporary housing lost the local community that they previously belonged to and had no opportunity to participate in community life [13, 14]. There was no commercial area such as an area with shops near most of the temporary houses [15].It has been reported that the health status of disaster survivors living in temporary housing deteriorated due to changes in their life style and psychosocial state compared to the health status of people who continued to live in the same house [16, 17]. It is possible that the living conditions in that period caused further weight gain.

The aim of this study was to determine whether there was a difference between changes in body weights of people living in temporary housing and people not living in temporary housing in tsunami-stricken areas during the medium-term and long-term recovery phases after the Great East Japan Earthquake and Tsunami.

## Materials and Methods

The research plan was deliberated and approved by the Ethics Committee of Iwate Medical University (approval no. H23-69). A total of 10,198 participants aged 18 years or older who provided written informed consent comprised the original cohort. The rights and welfare of the participants in this study were protected by the ethical guidelines outlined in the Declaration of Helsinki.

### Study population

**Fig 1:**
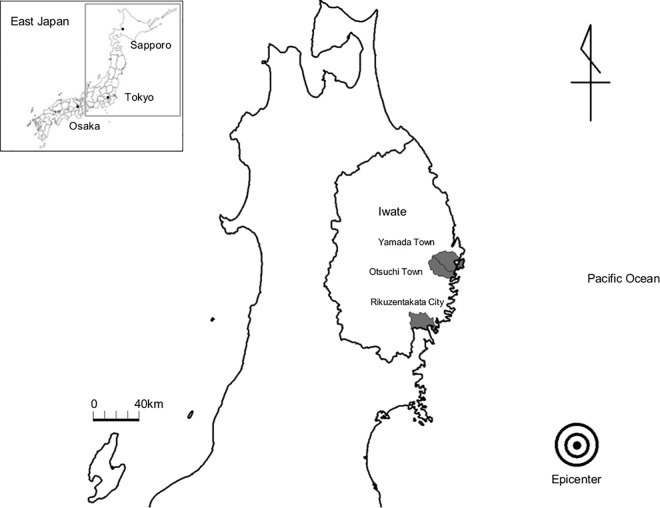
Map of the study area. The gray zone illustrates the study area along the Pacific Ocean coast that was affected by the tsunami. The study area includes Yamada Town, Otsuchi Town, and Rikuzentakata City. The epicenter of the earthquake is marked by a bull’s eye. The numbers of deaths or missing people were 834 in Yamada, 1,279 in Otsuchi, and 1,808 in Rikuzentakata, accounting for 4.5%, 8.4%, and 7.8% of the total populations of those municipalities, respectively.

We analyzed data that were obtained by the Research Project for Prospective Investigation of Health Problems Among Survivors of the Great East Japan Earthquake and Tsunami Disaster (RIAS) described previously [18–20] (Fig 1).

**Fig 2:**
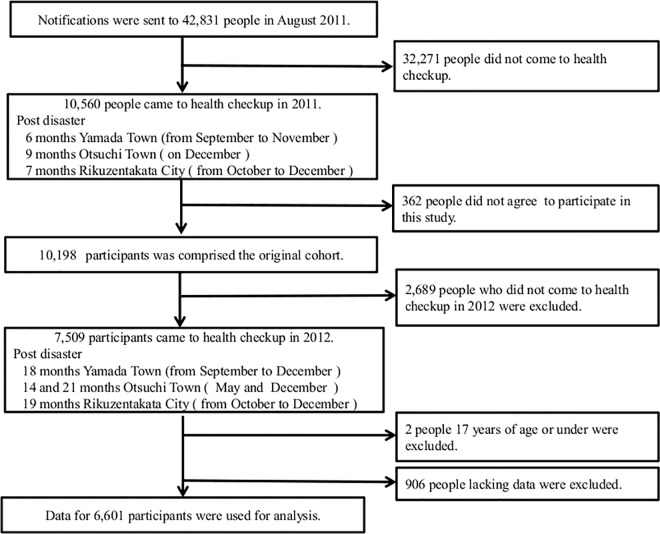
Flow chart of the procedure used to select participants for the study. The original cohort consisted of 10,198 participants in 2011. We excluded 2,689 persons who did not come to the health check-up in 2012, 2 people aged 17 years old or under, and 906 people who lacked at least one variable that was used for analysis. We used data from 6,601 participants for analysis.

The process for selection of participants is illustrated in Fig 2. We sent out notifications of the health survey and several types of questionnaire to all residents aged 18 years or older (total of 42,831 residents). In the 2011 survey, a total of 10,198 participants who provided written informed consent comprised the original cohort (acceptance rate of 96.6%). After excluding people who did not participate in the 2012 survey, those aged 17 years or younger, and those who lacked data for at least one variable that was necessary for analysis, we analyzed data for 6,601 participants (2,504 males and 4,097 females; 15.4% of the total population). The mean periods from the disaster were 7.7 months in the 2011 survey and 18.4 months in the 2012 survey.

### Measurements

#### Living conditions

**Fig 3:**
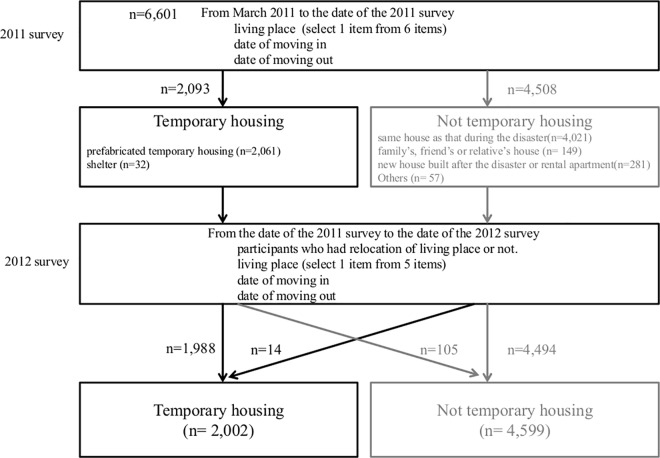
Flow chart of the process for determining living conditions. We determined living conditions by asking questions in the 2011 survey and in the 2012 survey according to the flow chart.

The process for determining living conditions is shown in Fig 3. In the 2011 survey, participants selected one of the following 6 items regarding living place: “prefabricated temporary housing,” “shelter,” “same house as that during the disaster,” “family’s, friend’s or relative’s house,” “new house built after the disaster or rental apartment,” and “others.” We classified the living conditions into two groups: a temporary housing (TH) group (prefabricated temporary housing and shelters) and non- temporary housing (NTH) group (same house as that during the disaster; family’s, friend’s, or relative’s house; new house built after the disaster or rental apartment; and others). In the 2012 survey, we asked participants whether they had relocated from their living place from the date of the 2011 survey to the date of the 2012 survey. Participants selected one of the following 5 items: “prefabricated temporary housing,” “same house as that during the disaster,” “family’s, friend’s, or relative’s house,” “new house built after the disaster or rental apartment,” and “others.” Finally, we classified the living conditions again into TH and NTH groups (2,002 participants in the TH group and 4,599 participants in the NTH group).

#### Anthropometrical examinations

Anthropometrical examinations (body weight (kg) and height (cm)) were performed (body weight: AD-6400, A&D Co., Ltd, Tokyo, Japan; BWB-200, TANITA Corporation, Tokyo, Japan; height: AD-6121A, A&D Co., Ltd, Tokyo, Japan; YG200D, YAGAMI INC, Nagoya, Japan). Body weight was measured with an accuracy of +/- 0.1 kg using a standard scale while dressed in very light clothing without shoes. Body mass index (BMI; kg/m^2^) was calculated as body weight (kg) divided by height (m)^2^.

#### Life style factors

We classified smoking status as current smokers or not current smokers. We additionally classified smoking status as quitting smoking during the follow-up period or not. We classified alcohol drinking status as drinkers or non-drinkers. We classified participants with activity levels <23 Mets per week as having low physical activity and participants with activity levels ≥23 Mets per week as having normal physical activity [21, 22]. To assess the average number of meals per day during the past several days, we classified the participants into two categories: those with a small number of meals (<3 times a day) and those with a normal number of meals (≥3 times a day).

#### Psychosocial factors

The economic status of the participants was assessed by asking “How do you feel about your current economic situation?” with 4 options of very severe, severe, slightly severe and normal. On the basis of their answers, participants were categorized into two groups: severe (very severe, severe, and slightly severe) and normal. The occupational status of the participants was assessed by asking participants “How was your job changed?”. The occupational status of the participants following the disaster was categorized into two groups: unemployed and employed. Based on previous research using the K6 scale in Japan, the participants were classified into those with psychological distress (scores of 5−24) and those with no psychological distress (score of 0−4) [23, 24]. Based on previous research on the Athens Insomnia Scale (AIS), the participants were classified as those with insomnia (scores of 6−24) and those with no insomnia (scores of 0−5) [25–28].

#### Cardiovascular risk factors

Metabolic profiles, including blood pressure, serum cholesterol levels and glycemic hemoglobin, and factors associated with body weight were measured. Blood pressure was consecutively measured twice in the sitting position using an automatic device after urination and a 5-minute rest. We calculated mean values of systolic blood pressure (SBP; mmHg) and diastolic blood pressure (DBP; mmHg). We examined results of blood tests for total cholesterol (TC; mg/dl), high-density lipoprotein cholesterol (HDLC; mg/dl) by using an automated analyzer, and glycosylated hemoglobin (HbA1c [Japan Diabetes Society, JDS]; %) by using an automated analyzer. Non high-density lipoprotein cholesterol (nonHDLC; mg/dl) was calculated as HDLC subtracted from TC. The value for HbA1c (%) was estimated as an NGSP equivalent value (%) calculated by the formula HbA1c (%) = HbA1c (JDS) (%) + 0.4 (%)[29].

Overweight was defined as BMI of ≥25 kg/m^2^. Hypertension was defined as SBP ≥140 mmHg, DBP ≥90 mmHg, and hypertension reported in a questionnaire or a combination of those. Dyslipidemia was defined as serum TC ≥220 mg/dl, serum HDLC <40 mg/dl, and dyslipidemia reported in the questionnaire or a combination of those. Diabetes mellitus was defined as plasma glucose level ≥200 mg/dl, plasma HbA1c level (NSGP) ≥6.5%, and diabetes mellitus reported in the questionnaire or a combination of those.

### Statistical analysis

We compared baseline characteristics in 2011 of the TH and NTH groups for each sex. In crude analysis, the chi-squared test was used for categorical variables and Student’s t-test was used for continuous variables. In age-adjusted analysis, logistic regression analysis was used for categorical variables and analysis of covariance was used for continuous variables. The change in body weight (kg) from 2011 to 2012 was calculated as body weight in 2011 (kg) subtracted from body weight in 2012 (kg). We compared the mean changes in body weight in the TH and NTH groups using Student’s t-test in all participants by age groups (18−44, 45−54, 55−64, 65−74, and ≥75 years). To examine the mean change in body weight from 2011 to 2012, multiple linear regression analysis was performed using mean change in body weight as a dependent variable and age, SBP, TC, HDLC, HbA1c, life style, psychosocial factors, and cardiovascular risk factors as independent variables. We also compared age-adjusted mean changes in the TH group and NTH group stratified by life style, psychosocial factors and cardiovascular risk factors using analysis of covariance.

All P-values were based on two-sided tests, and P-values <0.05 were considered statistically significant. The Statistical Package for Social Sciences (SPSS) version 19.0 (IBM) was used for all analyses.

## Results

Table 1 summarizes the baseline characteristics of participants in the TH and NTH groups by crude and age-adjusted analyses in the 2011 survey. There were 2,002 participants in the TH group and 4,599 participants in the NTH group. The average ages of the participants were 64.0 years for males and 61.4 years for females. The males in the NTH group were significantly older than those in the TH group. In age-adjusted analysis, there was no significant difference in body weight between the TH and NTH groups for either sex. The significant differences in TC levels, nonHDLC levels, and proportion of participants with dyslipidemia in the TH and NTH groups disappeared after age-adjusted analysis. Current smokers, low physical activity, small number of meals (<3 times), poor economic status, unemployed, psychological distress, and insomnia were more prevalent in the TH group than in the NTH group even after age-adjusted analysis. In females, the significant difference between the TH and the NTH groups in the prevalence of small number of meals (<3 times a day) disappeared after age-adjusted analysis. Even after age-adjusted analysis, the NTH group had significantly higher SBP and HbA1c levels than those in the TH group. However, low physical activity, poor economic status, unemployed, psychological distress, and insomnia were more prevalent in the TH group than in the NTH group.

**Table 1:** Baseline characteristics of participants in the TH group and the NTH group in the 2011 survey.

			Males (N = 2,504)			Females (N = 4,097)		
Living condition	Living conditions		TH group (N = 775)	NTH group (n = 1,729)	P-value	TH group (N = 1,227)	NTH group (N = 2,870)	P-value
**Age**	**Age (yr)**	**Mean (SD)**	62.4 (13.3)	64.6 (12.9)	< 0.001	61.0 (14.0)	61.5 (13.1)	0.314
	**Age class (18–44)**	**n (%)**	103 (13.3)	170 (9.8)	< 0.001	196 (16.0)	369 (12.9)	0.019
	**(45–54)**		97 (12.5)	144 (8.3)		144 (11.7)	329 (11.5)	
	**(55–64)**		165 (21.3)	397 (23.0)		321 (26.2)	870 (30.3)	
	**(65–75)**		279 (36.0)	650 (37.6)		380 (31.0)	896 (31.2)	
	**(≥75)**		131 (16.9)	368 (21.3)		186 (15.2)	406 (14.1)	
**Anthropometrical examination**	**Body weight (kg)**	**Mean (SD)**	66.5 (11.4)	65.0 (10.1)	0.001	53.8 (9.3)	53.7 (8.7)	0.695
		**Adjusted mean (SE)**	66.0 (0.4)	65.2 (0.2)	0.057	53.8 (0.3)	53.7 (0.2)	0.822
	**BMI (kg/m2)**	**Mean (SD)**	24.4 (3.6)	24.2 (3.1)	0.063	23.3 (3.6)	23.4 (3.5)	0.833
		**Adjusted mean (SE)**	24.4 (0.1)	24.2 (0.1)	0.181	23.3 (0.1)	23.4 (0.1)	0.926
**Blood pressure**	**SBP (mmHg)**	**Mean (SD)**	128.4 (17.2)	129.5 (17.4)	0.145	123.6 (18.1)	125.2 (19.1)	0.014
		**Adjusted mean (SE)**	128.6 (0.6)	129.3 (0.4)	0.357	123.8 (0.5)	125.1 (0.3)	0.029
	**DBP (mmHg)**	**Mean (SD)**	77.2 (10.6)	76.5 (10.7)	0.128	72.3 (10.7)	72.9 (10.5)	0.067
		**Adjusted mean (SE)**	77.1 (0.4)	76.5 (0.3)	0.205	72.3 (0.3)	72.9 (0.2)	0.094
**Blood tests**	**TC (mg/dl)**	**Mean (SD)**	199.7 (33.8)	196.6 (33.9)	0.037	210.4 (34.1)	209.6 (35.2)	0.515
		**Adjusted mean (SE)**	199.0 (1.2)	196.9 (0.8)	0.140	210.5 (1.0)	209.6 (0.7)	0.477
	**HDLC (mg/dl)**	**Mean (SD)**	59.0 (16.9)	59.2 (16.5)	0.865	67.2 (16.6)	66.6 (16.4)	0.272
		**Adjusted mean (SE)**	59.1 (0.6)	59.1 (0.4)	0.930	67.1 (0.5)	66.6 (0.3)	0.348
	**nonHDLC (mg/dl)**	**Mean (SD)**	140.7 (35.7)	137.5 (34.9)	0.037	143.2 (34.8)	143.0 (36.0)	0.897
		**Adjusted mean (SE)**	140.0 (1.3)	137.8 (0.8)	0.144	143.3 (1.0)	143.0 (0.7)	0.787
	**HbA1c (%)**	**Mean (SD)**	5.75 (0.74)	5.74 (0.66)	0.717	5.61 (0.46)	5.65 (0.58)	0.023
		**Adjusted mean (SE)**	5.76 (0.03)	5.74 (0.02)	0.368	5.61 (0.02)	5.65 (0.01)	0.038
**Life style factors**	**Current smokers**	**n (%)**	250 (32.3)	444 (25.7)	0.001	75 (6.1)	146 (5.1)	0.183
		**Adjusted proportion (SE)**	30.0 (1.7)	25.0 (1.1)	0.013	4.0 (0.5)	3.0 (0.3)	0.437
	**Quitting smoking**	**n (%)**	2.0 (0.5)	2.0 (0.3)	0.605	0.0 (0.2)	0.0 (0.1)	0.936
		**Adjusted proportion (SE)**	490 (63.2)	1073 (62.1)	0.577	168 (13.7)	368 (12.8)	0.45
	**Drinkers**	**n (%)**	490 (63.2)	1073 (62.1)	0.577	168 (13.7)	368 (12.8)	0.450
		**Adjusted proportion (SE)**	63.0 (1.7)	62.0 (1.2)	0.674	12.0 (0.9)	11.0 (0.6)	0.707
	**Low physical activity**	**n (%)**	506 (65.3)	1032 (59.7)	0.008	875 (71.3)	1911 (66.6)	0.003
		**Adjusted proportion (SE)**	66.0 (1.7)	60.0 (1.2)	0.004	71.0 (1.3)	67.0 (0.9)	0.002
	**Small number of meals (< 3 times)**	**n (%)**	70 (9.0)	70 (4.0)	< 0.001	69 (5.6)	120 (4.2)	0.044
		**Adjusted proportion (SE)**	7.0 (0.9)	3.0 (0.4)	< 0.001	4.0 (0.5)	3.0 (0.3)	0.113
**Psychosocial factors**	**Poor economic status**	**n (%)**	507 (65.4)	802 (46.4)	< 0.001	724 (59.0)	1324 (46.1)	< 0.001
		**Adjusted proportion (SE)**	65.0 (1.7)	47.0 (1.2)	< 0.001	59.0 (1.4)	46.0 (0.9)	< 0.001
	**Unemployed**	**n (%)**	167 (21.5)	259 (15.0)	< 0.001	338 (27.5)	455 (15.9)	< 0.001
		**Adjusted proportion (SE)**	21.0 (1.5)	15.0 (0.9)	< 0.001	27.0 (1.3)	15.0 (0.7)	< 0.001
	**Psychological distress**	**n (%)**	305 (39.4)	558 (32.3)	0.001	652 (53.1)	1276 (44.5)	< 0.001
		**Adjusted proportion (SE)**	39.0 (1.8)	32.0 (1.1)	0.002	53.0 (1.4)	44.0 (0.9)	< 0.001
	**Insomnia**	**n (%)**	247 (31.9)	373 (21.6)	< 0.001	548 (44.7)	994 (34.6)	< 0.001
		**Adjusted proportion (SE)**	31.0 (1.7)	22.0 (1.0)	< 0.001	45.0 (1.4)	35.0 (0.9)	< 0.001
**Cardiovascular risk factors**	**Overweight**	**n (%)**	288 (37.2)	633 (36.6)	0.792	361 (29.4)	840 (29.3)	0.921
		**Adjusted proportion (SE)**	37.0 (1.7)	37.0 (1.2)	0.999	29.0 (1.3)	29.0 (0.9)	0.872
	**Hypertension**	**n (%)**	392 (50.6)	899 (52.0)	0.513	531 (43.3)	1187 (41.4)	0.255
		**Adjusted proportion (SE)**	52.0 (1.9)	51.0 (1.3)	0.603	41.0 (1.6)	38.0 (1.0)	0.124
	**Dyslipidemia**	**n (%)**	278 (35.9)	541 (31.3)	0.024	588 (47.9)	1321 (46.0)	0.266
		**Adjusted proportion (SE)**	35.0 (1.7)	31.0 (1.1)	0.053	48.0 (1.4)	46.0 (0.9)	0.215
	**Diabetes mellitus**	**n (%)**	121 (15.6)	250 (14.5)	0.453	90 (7.3)	195 (6.8)	0.533
		**Adjusted proportion (SE)**	16.0 (1.3)	14.0 (0.8)	0.224	6.0 (0.7)	6.0 (0.5)	0.521

In the categorical variables, the upper row indicates n (%) and lower row indicates adjusted proportion (standard error).

In the continuous variables, the upper row indicates mean (standard deviation) and lower row indicates adjusted mean (standard error).

Abbreviations: TH group, temporary housing group; NTH group, non- temporary housing group; SBP, systolic blood pressure; DBP, diastolic blood pressure; TC, total cholesterol; HDLC, high-density lipoprotein cholesterol; HbA1c, glycosylated hemoglobin; nonHDLC, non-high-density lipoprotein cholesterol; SD, standard deviation; SE, standard error.

In the crude analysis, P-values were calculated using the chi-square test for categorical values and Student’s t-test for continuous values.

In the age-adjusted analysis, P-values were calculated using logistic regression analysis for categorical values and analysis of covariance for continuous values.

Table 2 presents the mean changes in body weight for all participants according to age in the TH and NTH groups. Body weights of males in the TH group increased except for those aged ≥75 years, while body weights of males in the NTH group decreased for all age groups. Body weights of females in the TH group decreased for all age group, while body weights in the NTH group were almost constant. In all subjects, there was a significant difference in mean changes in body weight between the TH and NTH groups in both sexes. In addition, body weight significantly increased in the TH group compared to that in the NTH group (mean change in body weight [95% CI] in males: TH group, +0.19 kg [0.00–0.37]; NTH group, -0.30 kg [-0.42 - -0.19]; P-value < 0.001; females: TH group, +0.57 kg [0.46–0.71]; NTH group, +0.02 kg [-0.06–0.10]; P-value < 0.001).

**Table 2:** Mean changes in body weight in all participants according to age in the TH and NTH groups.

		Males (N = 2,504)			Females (N = 4,097)		
Living conditions		TH group (N = 775)	NTH group (n = 1,729)	P-value	TH group (N = 1,227)	NTH group (N = 2,870)	P-value
		Mean change (95% CI)	Mean change (95% CI)		Mean change (95% CI)	Mean change (95% CI)	
**All participants**		0.19 (0.00–0.37)	-0.31 (-0.42 - -0.19)	< 0.001	0.59 (0.46–0.71)	0.02 (-0.06–0.10)	< 0.001
**Age groups**	18–44	0.43 (-0.24–1.10)	-0.26 (-0.75–0.23)	0.101	0.79 (0.68–0.90)	0.20 (-0.08–0.47)	0.014
	45–54	0.23 (-0.29–0.76)	-0.14 (-0.60–0.33)	0.363	0.33 (0.28–0.39)	0.11 (-0.17–0.38)	0.369
	55–64	0.20 (-0.25–0.64)	-0.26 (-0.54–0.02)	0.085	0.65 (0.58–0.72)	0.04 (-0.09–0.17)	<0.001
	65–74	0.19 (-0.09–0.47)	-0.41 (-0.58 - -0.24)	<0.001	0.64 (0.58–0.70)	-0.05 (-0.17–0.08)	<0.001
	≥75	-0.05 (-0.41–0.31)	-0.25 (-0.45 - -0.06)	0.335	0.35 (0.30–0.40)	-0.12 (-0.29–0.06)	0.003

Abbreviations: TH group, temporary housing group; NTH group, non- temporary housing group; BMI, body mass index; CI, confidence interval

P-values were calculated using Student’s t-test.

Table 3 reveals the results of multiple linear regression analysis of variables for examining the mean change in body weight from 2011 to 2012. The partial regression coefficient of mean change in body weight [standard error] in males in the TH group (reference: NTH group) was +0.52 kg [0.11] (P-value < 0.001) and that in females in TH group (reference: NTH group) was +0.56 kg [0.08] (P-value < 0.001). In males, there were significant relationships of the mean change in body weight with living condition (P-value < 0.001), age (P-value = 0.021), body weight (P-value = 0.009), HbA1c (P-value = 0.007) and quitting smoking (P-value = 0.010). In females, there were significant relationships of mean change in body weight with living condition (P-value < 0.001), SBP (P-value < 0.001), HDLC (P-value = 0.013) and quitting smoking (P-value = 0.035).

**Table 3:** Multiple linear regression analysis of variables for examining mean change in body weight between 2011 and 2012.

	Males (N = 2,504)			Females (N = 4,097)		
Variable	B	SE	P-value	B	SE	P-value
**TH group vs NTH group (2012)**	0.52	0.11	<0.001	0.56	0.08	<0.001
**Age (2011)**	-0.01	0.01	0.021	0.00	0.00	0.672
**Body weight (2011)**	-0.01	0.01	0.009	0.01	0.00	0.246
**SBP (2011)**	0.00	0.00	0.253	-0.01	0.00	<0.001
**TC (2011)**	0.00	0.00	0.453	0.00	0.00	0.791
**HDLC (2011)**	0.00	0.00	0.201	0.01	0.00	0.013
**HbA1c (2011)**	-0.20	0.08	0.007	-0.06	0.07	0.331
**Current smokers vs not current smokers (2011)**	-0.01	0.12	0.942	-0.07	0.17	0.661
**Quitting smoking vs not quitting smoking**	1.00	0.39	0.01	1.02	0.48	0.035
**Drinkers vs non-drinkers (2011)**	-0.01	0.11	0.961	-0.11	0.10	0.289
**Low physical activity vs normal physical activity (2011)**	0.01	0.11	0.963	0.03	0.07	0.722
**Small number of meals (< 3 times) vs normal number of meals (≥** **3 times) (2011)**	-0.13	0.23	0.566	0.10	0.17	0.567
**Poor economic status vs normal economic status (2011)**	-0.10	0.11	0.343	-0.04	0.07	0.591
**Unemployed vs employed (2011)**	-0.21	0.14	0.130	-0.17	0.09	0.062
**Psychological distress vs no psychological distress (2011)**	0.04	0.12	0.712	0.05	0.07	0.487
**Insomnia vs no insomnia (2011)**	0.07	0.13	0.608	0.06	0.08	0.416

B: partial regression coefficients.

Abbreviations: TH group, temporary housing group; NTH group, non- temporary housing group; SBP, systolic blood pressure; TC, total cholesterol; HDLC, high-density lipoprotein cholesterol; HbA1c, glycosylated hemoglobin; SE, standard error.

P-values were calculated by multiple linear regression analysis.

Table 4 summarizes the age-adjusted mean changes in body weight in the TH group and NTH group stratified by life style factors, psychosocial factors, and cardiovascular risk factors. The age-adjusted mean change in body weight increased in the TH group in all subjects except for male subjects who were non-drinkers, male subjects who were overweight and male subjects who had dyslipidemia. The age-adjusted mean change in body weight was significantly greater in the TH group than in the NTH group stratified by all factors in both sexes except for the male who quit smoking.

**Table 4:** Age-adjusted mean change in body weight between the TH group and NTH group stratified by lifestyle factors, psychosocial factors, and cardiovascular risk factors.

			Males (N = 2,504)							Females (N = 4,097)						
			TH group (N = 775)			NTH group (n = 1,729)				TH group (N = 1,227)			NTH group (n = 2,870)			
			n	Mean	SD	n	Mean	SD	P-value	n	Mean	SD	n	Mean	SD	P-value
**Life style factors**	**Smoking**	**Current smokers**	250	0.22	0.17	444	-0.19	0.13	0.053	75	0.35	0.28	146	0.32	0.20	0.921
		**Not current smokers**	525	0.16	0.11	1285	-0.34	0.07	< 0.001	1152	0.60	0.06	2724	0.00	0.04	< 0.001
	**Quitting smoking**	**Quitting smoking**	16	0.83	0.92	30	0.92	0.67	0.938	7	1.25	1.10	15	1.21	0.73	0.978
		**Not quitting smoking**	759	0.16	0.09	1699	-0.32	0.06	< 0.001	1220	0.58	0.06	2855	0.01	0.04	< 0.001
	**Drinkers**	**Drinkers**	490	0.30	0.11	1073	-0.34	0.07	< 0.001	168	0.64	0.17	368	-0.01	0.11	0.001
		**Non-drinkers**	285	-0.03	0.17	656	-0.24	0.11	0.286	1059	0.58	0.07	2502	0.02	0.04	< 0.001
	**Physical activity**	**Low physical activity**	506	0.19	0.11	1032	-0.32	0.08	< 0.001	875	0.55	0.07	1911	0.04	0.05	< 0.001
		**Normal physical activity**	269	0.15	0.16	697	-0.28	0.10	0.022	352	0.67	0.11	959	-0.03	0.07	< 0.001
	**Diet**	**A small number of meals**	70	0.08	0.37	70	-0.37	0.37	0.393	69	0.42	0.30	120	0.40	0.23	0.950
		**Normal number of meals**	705	0.19	0.09	1659	-0.30	0.06	< 0.001	1158	0.60	0.06	2750	0.00	0.04	< 0.001
**Psychosocial factors**	**Economic status**	**Poor economic status**	507	0.17	0.12	802	-0.35	0.09	0.001	724	0.59	0.09	1324	0.01	0.06	< 0.001
		**Normal**	268	0.20	0.15	927	-0.26	0.08	0.006	503	0.58	0.09	1546	0.03	0.05	< 0.001
	**Occupational status**	**Unemployed**	167	0.01	0.23	259	-0.46	0.18	0.111	338	0.52	0.13	455	-0.13	0.11	< 0.001
		**Employed**	608	0.22	0.10	1470	-0.27	0.06	< 0.001	889	0.61	0.07	2415	0.05	0.04	< 0.001
	**Psychological distress**	**Psychological distress**	305	0.24	0.15	558	-0.27	0.11	0.008	652	0.64	0.09	1276	0.04	0.07	< 0.001
		**No psychological distress**	470	0.14	0.11	1171	-0.32	0.07	0.001	575	0.52	0.08	1594	0.00	0.05	< 0.001
	**Insomnia**	**Insomnia**	247	0.30	0.18	373	-0.31	0.15	0.011	548	0.65	0.10	994	0.05	0.07	< 0.001
		**No insomnia**	528	0.12	0.11	1356	-0.30	0.07	0.001	679	0.53	0.08	1876	0.00	0.05	< 0.001
**Cardiovascular risk factors**	**Overweight**	**Overweight**	288	-0.02	0.17	633	-0.51	0.12	0.018	361	0.62	0.13	840	-0.07	0.09	< 0.001
		**Non overweight**	487	0.29	0.10	1096	-0.18	0.07	< 0.001	866	0.57	0.07	2030	0.06	0.05	< 0.001
	**Hypertension**	**Hypertension**	392	0.12	0.13	899	-0.36	0.09	0.002	531	0.56	0.09	1187	-0.10	0.06	< 0.001
		**Non hypertension**	383	0.25	0.13	8**3**0	-0.24	0.09	0.002	696	0.60	0.08	1683	0.10	0.05	< 0.001
	**Dyslipidemia**	**Dyslipidemia**	278	-0.03	0.16	541	-0.30	0.11	0.163	588	0.66	0.09	1321	-0.04	0.06	< 0.001
		**Non Dyslipidemia**	497	0.29	0.11	1188	-0.30	0.07	< 0.001	639	0.51	0.09	1549	0.07	0.06	< 0.001
	**Diabetes mellitus**	**Diabetes mellitus**	121	0.10	0.27	250	-0.56	0.19	0.043	90	0.27	0.25	195	-0.51	0.17	0.011
		**Non diabetes mellitus**	654	0.19	0.10	1479	-0.26	0.06	< 0.001	1137	0.61	0.06	2675	0.06	0.04	< 0.001

Abbreviations: TH group, temporary housing group; NTH group, non- temporary housing group.

P-values were calculated using analysis of covariance.

## Discussion

In this study, while there was no significant difference between body weights in the TH group and NTH group in the 2011 survey (about 7 months after the Great East Japan Earthquake and Tsunami), there was a significant difference between the mean changes in body weight in the TH group and NTH group from the 2011 survey to the 2012 survey (about 7–18 months after the disaster) for both sexes. Body weight increased in the TH group but decreased in the NTH group for both sexes in all age groups except for male subjects aged ≥75 years. In the multiple linear regression analysis, change in body weight was significantly greater in the TH group than in the NTH group in both sexes. In the stratified analysis, the age-adjusted mean changes in body weight increased in both sexes in the TH group regardless of life style, psychosocial factors and cardiovascular risk factors. The age-adjusted mean change in body weight was significantly greater in the TH group than in the NTH group in both sexes stratified by all factors except for quitting smoking in males. The findings obtained after adjusting for unfavorable risk factors including life style, psychosocial factors and cardiovascular risk factors identified that both males and females living in temporary housing had a significant increase in body weight. This is the first report revealed that body weights of people living in poor conditions in an area affected by the disaster increased significantly during the medium-term and long-term recovery phases after the disaster.

Previous studies have reported that the body weights of survivors in affected areas changed in the short term after an earthquake or hurricane. Survivors of the Irpinia earthquake (1980, Italy) reported increased body weight (+1.4 kg) 2 months after the disaster [10]. In the Great East Japan Earthquake and Tsunami, evacuees in the Soma area in Fukushima Prefecture reported increased body weight (+0.6 kg) 6 months after the disaster [11]. Based on the results of those studies, the participants in our study had significantly increased body weight about 7 months after the disaster. Furthermore, our results provided that body weights of participants in the NTH group were decreased about 18 months after the disaster indicate that body weights might decrease to weights prior to the disaster. On the other hand, the results revealed that body weights in the TH group were increased about 18 months after the disaster indicate that subjects in the TH group might have had increase in body weight at about 7 months, which further increased at about 18 months after the disaster (males, 0.52 kg; females, 0.56 kg). The difference in changes in body weight was related to the difference in living conditions. Arcaya et al. reported that survivors of Hurricane Katrina revealed an increase in BMI from 7 to 19 months after the disaster [12]. The results of our study were compatible with regard to increased body weight in the TH group but were not compatible regarding other points, the fact that the NTH group identified decreased body weight during the medium-term and long-term recovery phases (about 18 months after the disaster). The subjects of our study were people who had lost their homes in the tsunami and had lived in shelters for several months after the disaster. Furthermore, most of the subjects continued to live in temporary housing (container houses) made from tinplate in tsunami-stricken areas for more than 1 year after the disaster. Thus, body weight in the TH group increased from several months after the disaster due to transfer from shelters to container-type temporary housing. Prolonged stay in temporary housing might therefore be related to body weight gain. Since an increase in body weight of 1 kg increases the risk of cardiovascular disease mortality, the continuous increase in body weight every year after the disaster would cause future cardiovascular disease subjects in the TH group [30–32]. Thus, appropriate care is needed for subjects in the TH group.

In our study, we found that there was a significant difference between changes in body weight in the TH group and NTH group in both sexes. In males, body weight tended to increase or remain constant in the TH group but decreased in the NTH group. On the other hand, in females, body weight increased in the TH group but decreased or remained constant body weight in the NTH group. Some studies have shown that there are differences between body weights of males and females depending on life style and psychosocial factors [33, 34]. It has also been characterized that the association between higher level of stress and greater body weight was more pronounced in females than in males [35]. In our study, a comparison of life style and psychosocial factors between males and females revealed that most of the factors of baseline were worse in females than in males (data not shown). The female subjects in our study might have had changes in hormone levels caused by menopause because about 50% of the participants were older than > 65 years of age. Therefore, the differences in hormonal effects and life style and psychosocial factors in females might have caused more body weight gain than in males.

In addition to the relationship between living conditions and change in body weight, our study revealed several factors that are related to change in body weight. In males, body weight was positively associated with quitting smoking and was negatively associated with older age, greater body weight in 2011 and higher HbA1c. In females, body weight was positively associated with higher HDLC and quitting smoking and was negatively associated with higher SBP. Past studies reported that quitting smoking was associated with body weight gain and that older age was associated with body weight loss [36, 37]. On the other hand, our results identified there were negative associations of body weight with other factors including greater body weight in 2011 and higher HbA1c in males and lower HDLC and higher SBP in females who were in an unfavorable health condition in 2011. One possible explanation for the loss in body weight is that they might have changed their behavior due to awareness of unhealthy conditions in 2011 or they might have received medical intervention. Secondly, it is possible that there might be reverse causation between those factors and change in body weight.

A major factor causing body weight gain is energy imbalance, which is linked to a sedentary lifestyle and irregular dietary patterns [7, 38, 39]. Previous studies showed that survivors living in shelters and temporary housing tended to be immobile [14, 40] and tended to change their dietary pattern [41, 42]. We showed that the subjects in the TH group tended to have a lower level of physical activity and eat a smaller number of meals per day than subjects in the NTH group. However, we did not ask the subjects about changes in access to certain kinds of food, changes in the ability to prepare foods, and changes in the type and amount of food. Nishi et al. reported that people who were in difficult living conditions after Great East Japan Earthquake were likely to have a lower prudent dietary pattern (high intake of vegetables, fruits, seafood, and soy foods) [43]. Dietary patterns including prudent dietary pattern are associated with body weight changes [44–46]. Body weight gain in the TH group might be related to dietary pattern.

Although psychosocial factors were not related to changes in body weight in the TH group and the NTH group in multivariate analysis, living in temporary housing had an influence on body weight changes. It was previously reported that possible reasons for changes in body weight are environmental and psychosocial factors. Community environment factors, such as the home, health care and workplace, and social factors, such as marital status and educational level, all influence daily behavior [38, 47–52]. Daily behavior, which plays an important role in increasing energy consumption and decreasing energy expenditure, influences body weight [50]. However, there have been no reports the relationships of change in body weight with environmental and psychosocial factors after the disaster. Living in a small and inconvenient home might have an influence the behavior of survivors. Another possible explanation in the Great East Japan Earthquake was the difference in ease of access to medical services after the disaster. People living in temporary housing, for whom access to medical/health services might be difficult, could not receive appropriate medical guidance. Those factors might have affected body weight in the TH group during the medium-term and long-term recovery phases after the disaster. In this study, we could not investigate these possibilities. Further study is therefore needed.

The government has not been able to meet all of the demands of the survivors because the Great East Japan Earthquake and Tsunami destroyed a large area, which was already economically and socially weak owing to the aging society in the area. Owing to the limitations of the government, recovery will take a long time and survivors will have to continue to live in temporary housing for a long time. After the Great Hanshin Awaji Earthquake in 1995 in Japan, it took 5 years for all of the residents to move out of temporary housing. Considering the association between body weight gain and living in difficult conditions and the probability that survivors will continue to live in temporary housing for a long time, we must continue to monitor the body weights of people living in temporary housing.

### Limitations

The present study had several limitations. First, generalizability of the results is limited because the percentage of subjects aged 65 years or older in this study was larger than the total population in the affected area in October 2011 (49.9% versus 42%). Our sample may not be representative of the general population. We conducted additional analysis stratified by age of 65 years (S1 Table). There were significant relationships between mean change in body weight and living conditions in both sexes and in all age groups. The results suggested that the large percentage of elderly in our study did not affect the generality of the main conclusion. Another limitation is that we selected only people who took part in our surveys in 2011 and 2012. Unfortunately, we did not have information regarding for non-participants such as occupational status and body weight change. There was a possibility that our participants were likely to gain body weight because non-participants who were more prevalent in young male who were busy with jobs. We provided a comparison of baseline characteristics of the non-participants and participants (S2 Table). The percentage of elderly females in participants was large, than that in non-participants. Our results might not be valid for young males. However, considering life style factors, participants tended to be healthier than non-participants. Since non-participants had a more unfavorable lifestyle, large proportions of them being smokers and eating a small number of meals, their body weight may have increased more than that of the participants. We do not know whether the results of our study were overestimations or underestimations. Third, although we assessed psychological distress by the K6 scale, which has been validated as having high screening performance for measurement of mood disorders including depression, we did not asses PTSD. Because the Great East Japan Earthquake was of an unprecedented great scale, we made ethical decisions regarding the exclusion of an enormous questionnaire to vulnerable participants after the disaster. It has been reported that people with higher psychological stress are more likely to exhibit less healthy dietary behaviors with higher body weight [53]. Changes in the frequency of meals and types of foods consumed as a result of psychological trauma might have caused body weight gain in the TH group. Finally, we could not investigate details of food consumption and physical activity because we did not ask about details of these factors. Notably, we could not verify whether the calorie intake in the TH group was higher. Moreover, we did not ask about environmental and socioeconomic factors, including income, type of employment, educational level, relevant medications and disaster-related physical disabilities in our questionnaires. Since we started our study soon after the disaster, we had to reduce items of the questionnaire due to consideration of ethical aspects. If we had asked about these items, we may explain the relationship temporary housing and body weight gain more strongly.

## Conclusion

Analysis after adjusting for possible confounding factors showed that body weights significantly increased by +0.52 kg in males and by +0.56 kg in females (reference; NTH group) living in temporary housing in tsunami-stricken areas from 7 months to 18 months after the disaster. The results suggest that living in temporary housing after a catastrophic disaster is an important risk factor of body weight gain. Survivors have now been living in poor conditions such as temporary housing for more than 4 years. Therefore, we should plan to control body weights of disaster survivors living in temporary housing during the medium-term and long-term recovery phases after the disaster.

## Supporting Information

S1 Fig Photograph of temporary housing. **(A)** Total image. **(B**)Extended image.
(TIF)

S1 Table Multiple linear regression analysis of variables for examining mean change in body weight stratified by age of 65 years from 2011 to 2012. (PDF)

S2 Table Comparison of baseline characteristics of the non-participants and participants. (PDF)

S3 Table The data in the present study. (PDF)
