# Supplementary figures and images for: Weight Gain in Survivors Living in Temporary Housing in the Tsunami-Stricken Area during the Recovery Phase following the Great East Japan Earthquake and Tsunami
Source: PLoS One. 2016 Dec 1;11(12):e0166817. doi: 10.1371/journal.pone.0166817 (PMC5131987; doi:10.1371/journal.pone.0166817)

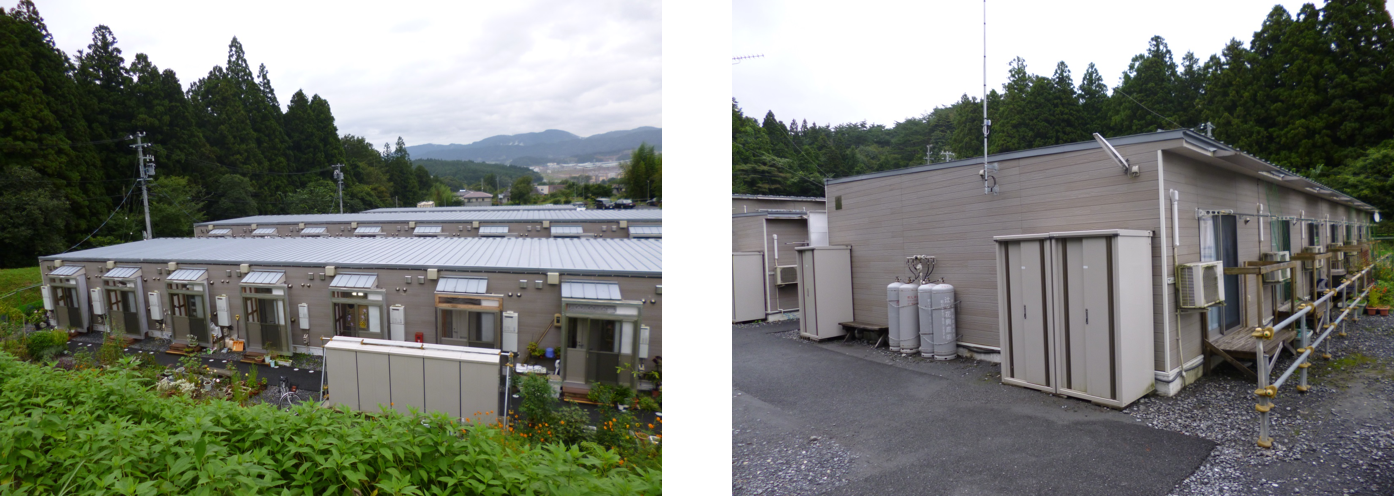

Supplement: S1 Fig — (A) Total image. (B)Extended image. (TIF) [file pone.0166817.s001.tif]
