# Supplementary material for: Weight Gain in Survivors Living in Temporary Housing in the Tsunami-Stricken Area during the Recovery Phase following the Great East Japan Earthquake and Tsunami
Source: PLoS One. 2016 Dec 1;11(12):e0166817. doi: 10.1371/journal.pone.0166817 (PMC5131987; doi:10.1371/journal.pone.0166817)
Supplement: S1 Table — (PDF) [file pone.0166817.s002.pdf]

S1 Table. Multiple linear regression analysis of variables for examining mean change in body weight stratified by age of 65 years from 2011 to 2012.

	Males (N=2,504)						Females (N=4,097)					
Age groups	Males < 65 (N=1,076)			Male ≥ 65 (N=1,428)			Females < 65 (N=2,229)			Females ≥ 65 (N=1,868)		
Variable	B	SE	P-value	B	SE	P-value	B	SE	P-value	B	SE	P-value
TH group vs NTH group (2012)	0.51	0.20	0.008	0.48	0.13	<0.001	0.54	0.11	<0.001	0.58	0.10	<0.001
Age (2011)	-0.01	0.01	0.517	-0.02	0.01	0.001	0.01	0.01	0.279	0.00	0.01	0.813
Body weight (2011)	-0.01	0.01	0.349	0.00	0.01	0.912	0.00	0.01	0.599	0.00	0.01	0.649
SBP (2011)	0.00	0.01	0.470	0.00	0.00	0.430	-0.01	0.00	0.032	-0.01	0.00	<0.001
TC (2011)	-0.01	0.00	0.090	0.00	0.00	0.249	0.00	0.00	0.051	0.00	0.00	0.093
HDLC (2011)	0.01	0.01	0.162	0.00	0.00	0.916	0.00	0.00	0.483	0.01	0.00	0.001
HbA1c (2011)	-0.28	0.13	0.035	-0.15	0.09	0.076	0.01	0.10	0.932	-0.17	0.08	0.036
Current smokers vs not current smokers (2011)	0.14	0.19	0.456	-0.19	0.16	0.235	0.05	0.19	0.801	-0.86	0.47	0.067
Quitting smoking vs not quitting smoking	0.70	0.65	0.285	1.37	0.46	0.003	0.77	0.55	0.161	3.46	1.42	0.015
Drinkers vs non-drinkers (2011)	-0.03	0.20	0.867	0.04	0.12	0.760	-0.15	0.13	0.239	0.12	0.19	0.537
Low physical activity vs normal physical activity (2011)	-0.09	0.19	0.626	0.08	0.12	0.527	-0.01	0.11	0.934	0.08	0.10	0.436
Small number of meals (< 3 times) vs normal number of meals (> 3 times) (2011)	0.07	0.31	0.834	-0.69	0.38	0.073	0.00	0.20	0.989	0.78	0.38	0.043
Poor economic status vs normal economic status (2011)	-0.29	0.19	0.131	0.02	0.12	0.889	-0.03	0.11	0.781	-0.07	0.10	0.493
Unemployed vs employed (2011)	-0.30	0.22	0.174	-0.07	0.17	0.703	-0.29	0.12	0.012	0.16	0.15	0.298
Psychological distress vs no psychological distress (2011)	-0.08	0.20	0.684	0.15	0.14	0.276	0.10	0.11	0.385	0.00	0.10	0.971
Insomnia vs no insomnia (2011)	0.21	0.21	0.321	-0.06	0.15	0.696	0.08	0.11	0.466	0.01	0.10	0.916

Abbreviations: TH group, temporary housing group; NTH group, non-temporary housing group; SBP, systolic blood pressure

TC, total cholesterol; HDLC, high-density lipoprotein cholesterol; HbA1c, glycosylated hemoglobin; SE, standard error

B: partial regression coefficients.

P-values were calculated by multiple linear regression analysis.
