# Supplementary material for: Weight Gain in Survivors Living in Temporary Housing in the Tsunami-Stricken Area during the Recovery Phase following the Great East Japan Earthquake and Tsunami
Source: PLoS One. 2016 Dec 1;11(12):e0166817. doi: 10.1371/journal.pone.0166817 (PMC5131987; doi:10.1371/journal.pone.0166817)
Supplement: S2 Table — (PDF) [file pone.0166817.s003.pdf]

S2 Table. Comparison of baseline characteristics of the non participants and participants.

			Males(n= 3,677)			Females (n= 5,613)		
			Non participants (n = 1,173)	Participants (n = 2,504)	*p*	Non participants (n = 1,516)	Participants (n = 4,097)	*p*
Age	Age (yr)	Mean (SD)	58.0 (16.5)	64.3 (12.5)	< 0.001	56.4 (17.3)	61.4 (12.8)	< 0.001
Age groups	18-44	n (%)	285 (24.3)	273 (10.9)	< 0.001	429 (28.3)	565 (13.8)	< 0.001
	45-54		142 (12.1)	241 (9.6)		234 (15.4)	473 (11.5)	
	55-64		293 (25.0)	562 (22.4)		291 (19.2)	1191 (29.1)	
	65-75		265 (22.6)	929 (37.1)		294 (19.4)	1276 (31.1)	
	≥75		188 (16.0)	499 (19.9)		268 (17.7)	592 (14.4)	
Anthropometric data	Body weight (kg)	Mean (SD)	66.1 (11.5)	65.3 (10.3)	0.043	54.4 (9.9)	53.7 (8.8)	0.024
		Adjusted mean (SE)	65.0 (0.3)	65.9 (0.2)	0.015	54.0 (0.2)	53.9 (0.1)	0.691
	BMI (kg/m2)	Mean (SD)	24.2 (3.4)	24.2 (3.2)	0.761	23.4 (4.0)	23.3 (3.5)	0.323
		Adjusted mean (SE)	24.1 (0.1)	24.3 (0.1)	0.141	23.5 (0.1)	23.3 (0.1)	0.043
Blood pressure	SBP (mmHg)	Mean (SD)	128.6 (18.4)	129.1 (17.3)	0.476	123.1 (20.3)	124.6 (18.9)	0.016
		Adjusted mean (SE)	129.5 (0.5)	128.7 (0.3)	0.172	125.0 (0.5)	124.0 (0.3)	0.066
	DBP (mmHg)	Mean (SD)	77.0 (11.7)	76.7 (10.5)	0.419	71.9 (11.3)	72.7 (10.5)	0.014
		Adjusted mean (SE)	77.1 (0.3)	76.7 (0.2)	0.341	72.5 (0.3)	72.5 (0.2)	0.962
Blood tests	TC (mg/dl)	Mean (SD)	198.4 (37.5)	197.9 (32.7)	0.704	206.1 (38.1)	210.1 (34.7)	0.001
		Adjusted mean (SE)	197.0 (1.0)	198.2 (0.7)	0.335	207.0 (0.9)	209.5 (0.6)	0.019
	HDLC (mg/dl)	Mean (SD)	59.0 (17.1)	59.2 (16.4)	0.765	66.7 (16.4)	66.7 (16.3)	0.986
		Adjusted mean (SE)	59.0 (0.5)	59.1 (0.3)	0.871	66.0 (0.4)	67.0 (0.3)	0.045
	nonHDLC (mg/dl)	Mean (SD)	139.5 (39.0)	138.8 (34.3)	0.618	139.4 (39.7)	143.4 (35.4)	0.001
		Adjusted mean (SE)	138.0 (1.1)	139.1 (0.7)	0.394	140.9 (0.9)	142.5 (0.6)	0.157
	HbA1c (%)	Mean (SD)	5.74 (0.89)	5.76 (0.70)	0.677	5.59 (0.58)	5.64 (0.54)	0.007
		Adjusted mean (SE)	5.78 (0.02)	5.73 (0.02)	0.061	5.63 (0.01)	5.62 (0.01)	0.722
Life Style factors	Current smokers	n (%)	455 (38.8)	694 (27.7)	< 0.001	184 (12.1)	221 (5.4)	< 0.001
		Adjusted proportion (SE)	34.0 (1.5)	28.0 (0.9)	< 0.001	7.0 (0.6)	4.0 (0.3)	< 0.001
	Drinkers	n (%)	696 (59.3)	1563 (62.4)	0.073	290 (19.1)	536 (13.1)	< 0.001
		Adjusted proportion (SE)	59.0 (1.5)	63.0 (1.0)	0.035	15.0 (0.9)	12.0 (0.5)	0.024
	Low physical activity	n (%)	691 (59.3)	1538 (61.4)	0.212	1028 (68.2)	2786 (68.0)	0.904
		Adjusted proportion (SE)	60.0 (1.5)	61.0 (1.0)	0.626	69.0 (1.2)	68.0 (0.7)	0.380
	Small number of meals (< 3 times)	n (%)	133 (11.4)	140 (5.6)	< 0.001	121 (8.0)	189 (4.6)	< 0.001
		Adjusted proportion (SE)	8.0 (0.8)	5.0 (0.4)	0.001	4.0 (0.5)	4.0 (0.3)	0.172
Psychosocial factors	Poor economic status	n (%)	639 (54.9)	1309 (52.3)	0.139	796 (52.6)	2048 (50.0)	0.077
		Adjusted proportion (SE)	52.0 (1.5)	54.0 (1.0)	0.498	51.0 (1.3)	51.0 (0.8)	0.838
	Unemployed	n (%)	207 (18.2)	426 (17.0)	0.373	315 (21.5)	793 (19.4)	0.076
		Adjusted proportion (SE)	17.0 (1.1)	17.0 (0.8)	0.927	19.0 (1.0)	19.0 (0.6)	0.962
	Psychological distress	n (%)	435 (37.5)	863 (34.5)	0.077	718 (48.0)	1928 (47.1)	0.521
		Adjusted proportion (SE)	36.0 (1.4)	35.0 (1.0)	0.545	48.0 (1.3)	47.0 (0.8)	0.795
	Insomnia	n (%)	298 (25.7)	620 (24.8)	0.546	555 (37.2)	1542 (37.6)	0.751
		Adjusted proportion (SE)	25.0 (1.3)	25.0 (0.9)	0.965	38.0 (1.3)	37.0 (0.8)	0.991
Cardiovascular risk factors	Overweight	n (%)	449 (38.3)	921 (36.8)	0.382	464 (30.7)	1201 (29.3)	0.318
		Adjusted proportion (SE)	37.0 (1.4)	37.0 (1.0)	0.855	31.0 (1.2)	29.0 (0.7)	0.063
	Hypertension	n (%)	509 (43.4)	1291 (51.6)	< 0.001	583 (38.5)	1718 (41.9)	0.020
		Adjusted proportion (SE)	47.0 (1.6)	49.0 (1.1)	0.276	40.0 (1.5)	36.0 (0.9)	0.019
	Dyslipidemia	n (%)	419 (35.7)	819 (32.7)	0.072	635 (41.9)	1909 (46.6)	0.002
		Adjusted proportion (SE)	35.0 (1.4)	33.0 (0.9)	0.352	43.0 (1.3)	46.0 (0.8)	0.127
	Diabetes mellitus	n (%)	154 (13.1)	371 (14.8)	0.173	107 (7.1)	285 (7.0)	0.890
		Adjusted proportion (SE)	13.0 (1.0)	13.0 (0.7)	0.911	7.0 (0.6)	6.0 (0.4)	0.252

In crude analysis, the chi-squared test was used for categorical variables and Student’s t-test was used for continuous variables. Categorical variables are shown as n (%) and continuous variables are shown as mean (SD).

In age-adjusted analysis, logistic regression analysis was used for categorical variables and analysis of covariance was used for continuous variables. Categorical variables are shown as adjusted proportion (SE) and continuous variables are shown as adjusted mean (SE).

Abbreviation: SBP, systolic blood pressure; DBP, diastolic blood pressure

TC, total cholesterol; HDLC, high-density lipoprotein cholesterol; HbA1c, glycosylated hemoglobin; nonHDLC, non-high-density lipoprotein cholesterol; SD, standard deviation; SE, standard error.
