# Supplementary material for: Weight Gain in Survivors Living in Temporary Housing in the Tsunami-Stricken Area during the Recovery Phase following the Great East Japan Earthquake and Tsunami
Source: PLoS One. 2016 Dec 1;11(12):e0166817. doi: 10.1371/journal.pone.0166817 (PMC5131987; doi:10.1371/journal.pone.0166817)
Supplement: S3 Table — (PDF) [file pone.0166817.s004.pdf]

S3 Table. The data in the present study.

LP_2011_d	LP_2012_d	age	sex	height	weight	dweight	BMI	sbp	dbp	TC	HDL	nonHDLC	Hba1c_N	smoke_ph1	R_quitSM	drink_ph1	pas	q3_1c	q13c	q5_2_c	k6c	aisc	obesity	R_t1ht	R_t1lipid_o	t1dmt_i
1	1	68	1	162	56.4	.30	21.5	137	74	166	61	105.00	5.10	1	1.00	0	0	1	1	1	0	1	1	1	1	1
1	1	58	2	148	53.7	1.60	24.3	114	73	222	51	171.00	5.40	1	1.00	1	0	1	0	1	0	1	1	1	0	1
0	0	75	1	164	54.0	-1.00	20.0	153	88	202	69	133.00	5.60	1	1.00	1	1	0	0	1	1	1	1	0	1	1
0	0	72	2	152	66.3	-2.00	28.6	151	89	260	58	202.00	6.10	1	1.00	1	0	1	1	1	1	1	0	0	0	1
1	1	45	2	161	52.6	.10	20.4	99	69	170	70	100.00	5.20	0	1.00	0	0	1	1	1	0	1	1	1	1	1
1	1	76	2	147	59.7	-3.40	27.6	132	78	136	54	82.00	5.40	1	1.00	1	0	1	1	1	0	0	0	1	1	1
1	1	68	2	150	61.5	.30	27.3	106	64	245	59	186.00	5.60	1	1.00	1	0	1	0	1	1	0	0	1	0	1
1	1	47	2	152	63.1	-.60	27.3	104	68	176	69	107.00	5.70	1	1.00	1	1	0	0	1	1	1	0	1	1	1
0	0	76	2	148	54.6	.20	24.8	126	69	201	51	150.00	5.70	1	1.00	1	0	1	0	1	0	1	1	0	1	1
1	1	44	2	160	51.6	2.80	20.2	106	59	175	48	127.00	5.40	1	1.00	1	0	1	1	1	1	1	1	1	1	1
1	1	50	2	151	49.2	3.30	21.4	112	64	204	70	134.00	4.40	1	1.00	1	1	1	1	1	0	0	1	1	1	1
1	1	71	1	155	63.8	-1.30	26.4	122	75	210	57	153.00	7.80	0	1.00	1	1	1	0	1	1	1	0	1	1	0
1	1	38	2	158	43.6	-1.40	17.4	92	52	129	63	66.00	4.80	1	1.00	0	0	0	1	1	1	1	1	1	1	1
0	0	74	2	145	50.2	-.60	23.8	127	62	190	54	136.00	5.70	1	1.00	1	0	1	0	0	0	0	1	0	1	1
1	1	68	1	164	85.7	4.30	31.7	118	76	209	48	161.00	5.20	1	1.00	0	0	1	0	0	0	1	0	0	1	1
1	1	66	2	149	62.6	-8.40	28.3	120	77	200	67	133.00	5.80	1	1.00	1	0	1	0	1	0	1	0	1	1	1
1	1	79	2	144	35.2	1.90	16.9	158	78	171	47	124.00	5.90	1	1.00	1	1	1	1	1	1	1	1	0	1	1
1	1	37	2	164	52.0	.60	19.3	99	65	155	52	103.00	5.30	0	1.00	0	0	0	0	1	0	0	1	1	1	1
1	1	49	1	181	76.3	4.30	23.2	128	80	180	77	103.00	5.40	0	1.00	0	1	1	0	1	1	1	1	1	1	1
1	1	63	2	159	56.1	1.70	22.2	137	92	329	75	254.00	5.40	1	1.00	1	0	1	1	1	0	1	1	0	0	1
1	1	54	2	157	77.7	3.30	31.3	159	81	229	54	175.00	5.30	1	1.00	1	0	1	1	1	1	1	0	0	0	1
1	1	64	2	155	54.1	.40	22.4	119	72	179	42	137.00	5.60	1	1.00	1	1	1	1	1	0	1	1	1	1	1
1	1	48	2	165	48.7	-.70	17.9	126	67	194	102	92.00	5.20	1	1.00	0	0	1	0	1	1	1	1	1	1	1
1	1	58	2	159	60.5	2.00	23.9	163	100	186	57	129.00	5.50	1	1.00	1	0	1	1	1	1	1	1	0	1	1
1	1	60	2	151	56.0	-3.60	24.5	142	82	237	50	187.00	6.50	1	1.00	1	0	1	0	0	1	0	1	0	0	0
1	1	73	2	140	67.9	-1.40	34.4	105	68	192	66	126.00	5.60	1	1.00	1	0	1	1	1	0	1	0	0	1	1
1	1	82	2	145	39.0	.80	18.4	96	59	158	56	102.00	6.00	1	1.00	1	1	1	0	1	0	0	1	0	1	1
1	1	58	1	169	65.8	-1.60	23.1	125	76	162	52	110.00	5.40	1	1.00	0	1	1	1	1	1	1	1	0	1	1
1	1	55	2	149	53.0	2.80	23.8	125	83	202	60	142.00	5.90	1	1.00	1	0	1	1	1	0	0	1	1	1	1
0	0	65	2	148	57.8	6.00	26.5	95	52	211	79	132.00	5.60	1	1.00	1	0	1	0	0	0	1	0	0	1	1
1	1	70	2	144	50.5	-.30	24.4	108	63	170	69	101.00	5.70	1	1.00	0	1	1	1	1	0	1	1	1	1	1
1	1	73	1	165	61.8	1.40	22.8	124	70	212	36	176.00	6.00	0	1.00	1	1	1	0	1	1	1	1	1	0	1
1	1	67	2	150	50.6	-2.40	22.5	86	58	184	59	125.00	6.10	1	1.00	1	0	1	1	1	0	1	1	1	0	1
1	1	60	2	156	56.4	-1.40	23.0	101	63	203	40	163.00	5.50	1	1.00	1	1	1	0	1	0	1	1	1	1	1
1	1	63	1	172	73.6	-1.30	24.9	104	66	233	44	189.00	5.60	1	1.00	0	0	1	0	1	1	0	1	1	0	1
1	1	60	2	153	55.8	1.50	23.9	99	65	256	66	190.00	5.50	1	1.00	1	0	1	0	1	1	1	1	0	0	1

1	1	53	2	149	57.7	1.10	26.1	121	70	215	94	121.00	5.40	1	1.00	0	1	0	0	1	0	0	0	1	1	1
1	1	39	2	151	42.1	1.80	18.5	100	66	191	71	120.00	5.60	1	1.00	1	1	1	0	1	0	0	1	1	1	1
1	1	69	2	150	45.7	-.90	20.3	126	75	191	49	142.00	5.60	1	1.00	1	0	1	1	1	1	0	1	0	1	1
1	1	58	2	151	61.6	-.10	27.1	104	59	240	60	180.00	5.30	1	1.00	1	0	1	0	0	1	1	0	1	0	1
1	1	62	2	159	48.6	1.80	19.1	104	62	187	67	120.00	5.40	1	1.00	0	1	1	1	1	1	1	1	1	1	1
0	0	18	2	158	58.5	.20	23.4	105	52	221	65	156.00	5.30	1	1.00	1	0	1	1	1	0	1	1	1	0	1
1	1	64	2	151	51.3	1.00	22.4	109	61	203	76	127.00	5.60	1	1.00	1	0	0	1	1	0	0	1	1	1	1
1	1	30	1	169	83.9	-3.60	29.4	125	73	229	43	186.00	5.60	1	1.00	1	0	0	0	1	0	0	0	1	0	1
0	0	73	1	158	66.9	6.50	26.9	160	84	271	92	179.00	5.50	1	1.00	0	0	1	1	0	0	1	0	0	0	1
1	1	40	1	173	70.1	-2.70	23.4	109	62	166	65	101.00	5.40	0	1.00	0	1	1	0	1	1	1	1	1	1	1
1	1	71	2	154	51.5	-.10	21.6	121	79	248	75	173.00	5.70	1	1.00	1	0	1	0	1	0	0	1	1	0	1
0	0	46	2	159	61.3	3.10	24.3	132	72	222	87	135.00	5.70	0	1.00	0	1	1	0	0	0	0	1	0	0	1
1	1	58	2	154	43.7	-1.40	18.4	117	68	247	53	194.00	5.80	1	1.00	1	0	1	0	1	0	0	1	1	0	1
1	1	74	2	150	51.9	.80	23.1	104	60	234	75	159.00	5.90	1	1.00	1	0	1	1	1	1	1	1	1	0	1
1	1	67	1	160	52.5	-1.30	20.4	138	75	183	96	87.00	5.40	0	1.00	0	0	1	1	1	1	1	1	0	1	1
0	0	58	1	156	60.7	4.00	24.8	104	74	169	65	104.00	5.50	1	1.00	0	0	1	0	1	1	0	1	0	1	1
1	1	61	2	146	46.1	-2.60	21.6	142	79	265	67	198.00	5.90	0	1.00	1	0	0	0	0	0	0	1	0	0	1
0	0	78	1	159	53.9	-.10	21.2	101	67	142	64	78.00	5.50	1	1.00	1	0	1	1	1	0	1	1	1	1	1
1	1	68	1	157	61.5	-1.20	25.1	141	71	255	76	179.00	5.70	1	1.00	1	0	1	1	1	1	1	0	0	0	1
0	0	42	2	162	47.0	-5.60	17.9	125	79	190	106	84.00	5.60	1	1.00	1	1	1	0	1	0	0	1	1	1	1
1	1	59	2	147	49.2	1.90	22.7	111	59	206	65	141.00	5.80	1	1.00	1	1	1	0	0	0	0	1	1	0	1
1	1	63	2	151	51.1	-7.30	22.3	134	77	230	69	161.00	6.50	1	1.00	1	0	1	1	1	0	1	1	1	0	0
0	0	73	2	155	68.4	.90	28.6	131	80	209	42	167.00	5.80	1	1.00	1	0	1	0	0	1	1	0	1	1	1
1	1	69	1	150	56.3	-1.40	24.9	142	79	243	64	179.00	6.60	1	1.00	0	0	1	1	1	1	1	1	0	0	0
0	0	70	1	159	69.5	3.40	27.4	117	72	217	40	177.00	5.30	1	1.00	0	1	1	0	0	0	1	0	0	1	1
0	0	69	2	146	59.7	-2.70	28.0	144	82	224	68	156.00	6.00	1	1.00	1	1	1	0	1	0	0	0	0	0	1
0	0	77	1	161	57.1	2.50	21.9	114	78	215	54	161.00	5.40	1	1.00	1	0	1	0	1	0	0	1	0	1	1
0	0	52	2	154	47.5	1.00	19.9	110	59	217	77	140.00	5.50	1	1.00	1	0	1	0	1	1	1	1	1	1	1
0	0	64	2	149	49.9	3.30	22.5	152	80	220	76	144.00	5.90	1	1.00	1	0	0	0	1	0	0	1	0	0	1
1	1	81	1	156	50.1	2.20	20.6	105	60	192	56	136.00	6.10	1	1.00	0	0	1	0	1	0	1	1	1	1	1
0	0	69	2	153	51.3	2.00	22.0	110	68	273	44	229.00	6.00	1	1.00	0	0	1	0	1	0	0	1	1	0	1
1	1	60	2	148	48.6	1.50	22.0	113	66	222	61	161.00	5.70	1	1.00	1	0	1	0	1	1	1	1	1	0	1
0	0	73	1	163	69.4	-2.70	26.0	99	65	169	61	108.00	6.20	1	1.00	0	0	1	1	1	1	1	0	0	0	0
1	1	70	2	137	49.2	.80	26.2	142	75	201	65	136.00	6.60	1	1.00	1	1	1	1	1	1	1	0	0	1	0
0	0	70	1	157	53.3	-1.70	21.5	123	71	177	76	101.00	8.10	1	1.00	0	1	1	0	1	1	1	1	1	1	0
1	1	66	1	160	69.4	1.50	27.2	158	97	204	45	159.00	6.20	1	1.00	0	1	1	1	1	0	0	0	0	1	1
1	1	39	2	158	55.5	-3.50	22.3	116	72	199	55	144.00	5.90	1	1.00	1	0	1	1	1	1	0	1	1	1	1
1	1	41	1	169	90.3	-.30	31.5	118	78	183	48	135.00	5.10	1	1.00	0	0	1	0	1	1	1	0	1	1	1
1	1	61	2	160	50.7	1.50	19.8	111	70	217	55	162.00	5.60	1	1.00	1	1	1	1	1	1	1	1	0	1	1

1	1	73	1	164	57.9	.90	21.4	119	68	202	55	147.00	5.40	0	1.00	1	0	1	0	1	1	1	1	1	1	1
0	0	51	2	150	65.4	1.30	29.0	123	73	196	67	129.00	5.70	1	1.00	1	0	1	0	0	1	0	0	1	1	1
1	1	68	1	156	60.5	.40	24.9	127	92	202	77	125.00	5.60	1	1.00	0	0	1	0	1	0	0	1	0	1	1
1	1	70	1	162	67.8	-2.00	25.8	147	80	226	69	157.00	5.60	1	1.00	0	1	1	0	1	0	1	0	0	0	1
1	1	39	2	160	72.6	-1.60	28.3	122	73	187	62	125.00	6.00	1	1.00	1	1	1	1	1	0	1	0	1	1	1
1	1	69	2	144	58.7	.40	28.1	112	76	229	48	181.00	6.30	1	1.00	1	0	1	0	1	0	1	0	0	0	0
0	0	26	1	172	63.2	5.20	21.4	98	57	224	61	163.00	5.40	0	1.00	0	1	1	0	1	0	1	1	1	0	1
0	0	57	1	170	69.8	3.20	24.2	128	83	185	55	130.00	6.00	1	1.00	1	1	1	1	1	1	1	1	1	1	1
1	1	70	1	161	56.2	-1.30	21.6	122	76	170	41	129.00	6.00	1	1.00	1	0	1	1	1	1	0	1	1	1	1
1	1	77	2	144	44.7	1.20	21.7	109	56	171	59	112.00	5.50	1	1.00	1	0	1	0	1	0	0	1	1	1	1
1	1	69	2	154	51.0	-1.60	21.4	119	62	205	60	145.00	5.70	1	1.00	1	0	1	1	1	1	1	1	1	1	1
1	1	37	1	171	53.1	-2.40	18.1	119	78	177	112	65.00	5.30	1	1.00	0	0	1	0	1	0	0	1	1	1	1
1	1	73	1	165	73.1	-2.10	27.0	127	70	148	51	97.00	5.10	1	1.00	0	1	1	0	0	1	0	0	0	1	1
1	1	67	1	159	62.1	-.60	24.5	120	69	203	55	148.00	5.60	1	1.00	0	0	1	0	0	1	1	1	0	0	1
1	1	57	2	155	48.3	-1.10	20.1	103	53	178	64	114.00	5.70	1	1.00	1	0	1	0	1	0	0	1	1	0	1
1	1	62	1	170	71.6	.90	24.8	139	85	204	68	136.00	5.70	1	1.00	0	0	1	0	1	0	0	1	0	1	1
1	1	78	1	157	54.2	-.40	22.1	155	80	152	49	103.00	6.00	0	2.00	0	1	1	1	1	1	1	1	0	1	1
1	1	73	1	160	49.6	.90	19.2	112	73	217	72	145.00	5.20	1	1.00	1	1	1	0	0	1	1	1	1	1	1
1	1	44	1	151	55.3	1.00	24.3	115	69	195	35	160.00	5.40	0	1.00	0	0	1	1	1	0	1	1	0	0	1
1	1	67	2	154	61.4	-.70	25.9	134	77	226	59	167.00	5.60	1	1.00	0	0	1	1	1	1	1	0	1	0	1
1	1	66	2	141	43.8	-.50	22.1	114	67	260	66	194.00	5.70	1	1.00	1	1	1	1	1	1	0	1	1	0	1
1	1	62	1	168	67.1	1.00	23.7	133	72	171	48	123.00	5.60	1	1.00	0	1	1	1	1	1	1	1	1	1	1
1	1	71	1	154	50.7	1.10	21.4	152	83	204	65	139.00	6.30	1	1.00	1	1	1	1	1	1	1	1	0	1	1
0	0	49	1	162	64.8	2.00	24.8	136	79	222	34	188.00	5.80	0	1.00	1	0	1	0	1	1	0	1	1	0	1
1	1	61	2	147	49.3	-.50	22.8	133	77	192	65	127.00	5.80	1	1.00	1	0	1	1	1	1	0	1	1	1	1
1	1	63	2	140	52.5	-2.60	26.7	98	59	194	47	147.00	5.60	1	1.00	1	0	1	1	1	0	1	0	1	1	1
1	1	72	1	172	64.4	.40	21.8	97	72	201	57	144.00	5.40	1	1.00	1	1	1	1	1	1	1	1	1	1	1
0	0	76	1	161	64.8	3.30	25.0	132	87	228	52	176.00	5.00	1	1.00	0	0	1	0	0	1	0	0	1	0	1
1	1	69	2	149	42.5	-1.70	19.1	101	63	196	80	116.00	6.10	1	1.00	1	0	1	1	1	0	1	1	1	1	1
1	1	76	2	151	43.3	-4.40	19.0	137	82	233	84	149.00	5.40	1	1.00	1	0	1	0	1	1	1	1	0	0	1
1	1	69	1	153	56.9	-3.50	24.4	158	105	164	74	90.00	5.40	1	1.00	0	0	1	1	1	1	1	1	0	1	1
1	1	61	2	146	51.7	.70	24.2	101	61	207	68	139.00	5.50	1	1.00	1	0	1	1	1	1	1	1	1	1	1
1	1	58	2	154	64.6	-5.70	27.3	154	88	251	60	191.00	6.10	1	1.00	1	1	1	0	0	1	1	0	0	0	1
1	1	52	2	156	41.9	1.70	17.2	110	57	239	87	152.00	5.60	0	1.00	1	0	1	1	1	1	1	1	1	0	1
1	1	62	2	152	66.1	.40	28.7	121	76	197	58	139.00	6.00	1	1.00	1	1	1	1	1	1	1	0	1	1	1
1	1	69	1	165	60.7	-4.70	22.1	99	58	199	42	157.00	6.20	1	1.00	0	1	1	1	1	1	1	1	1	1	1
1	1	63	1	162	63.0	-1.70	24.0	126	80	163	47	116.00	6.00	1	1.00	0	1	1	0	0	0	1	1	1	1	1
1	1	52	2	160	58.5	2.00	22.7	125	78	280	49	231.00	6.00	1	1.00	1	1	1	0	1	0	0	1	1	0	1
1	1	63	2	153	64.6	-3.80	27.6	125	76	189	44	145.00	5.60	1	1.00	1	1	1	1	1	1	1	0	1	0	1

1	1	72	2	146	47.8	-.60	22.4	148	80	233	57	176.00	5.50	1	1.00	1	0	1	0	1	1	1	1	0	0	1
1	1	60	2	144	53.7	3.10	25.8	115	66	185	79	106.00	5.70	1	1.00	1	0	1	0	1	0	0	0	1	0	1
0	0	60	2	155	43.5	1.20	18.1	108	65	246	89	157.00	5.60	1	1.00	1	1	1	0	1	0	0	1	1	0	1
1	1	66	2	156	51.7	-.20	21.2	143	74	221	92	129.00	5.40	1	1.00	0	1	1	1	1	1	0	1	0	0	1
0	0	77	1	158	66.3	1.20	26.4	151	77	222	44	178.00	6.70	1	1.00	0	1	1	0	1	0	1	0	0	0	0
1	1	69	1	162	60.5	-.20	23.0	109	69	178	86	92.00	5.80	1	1.00	0	1	1	1	1	1	1	1	1	1	1
1	1	72	2	151	55.7	1.00	24.4	131	79	262	66	196.00	5.90	1	1.00	1	0	1	1	1	0	0	1	1	0	1
0	0	79	1	157	59.3	1.00	23.9	146	80	223	63	160.00	5.50	1	1.00	0	0	1	0	0	0	1	1	0	0	1
1	1	58	2	150	50.7	-.10	22.6	147	86	223	48	175.00	6.10	1	1.00	1	1	1	0	1	0	1	1	0	0	1
0	0	73	1	153	57.8	.20	24.6	120	71	209	39	170.00	6.80	1	1.00	1	0	1	0	1	0	0	1	1	0	0
1	1	54	2	159	56.4	-.90	22.4	101	66	212	72	140.00	5.50	1	1.00	0	0	1	1	1	1	1	1	1	1	1
0	0	59	1	165	61.2	3.20	22.4	145	82	188	95	93.00	5.20	1	1.00	0	0	1	0	1	1	1	1	0	1	1
1	1	43	1	174	98.0	-5.50	32.4	137	84	166	48	118.00	5.50	1	1.00	0	1	1	0	1	1	0	0	1	1	1
0	0	61	2	154	71.5	.80	30.1	154	83	193	55	138.00	6.30	1	1.00	1	0	1	0	1	0	1	0	0	1	0
1	1	71	2	154	58.1	2.20	24.6	115	71	216	35	181.00	5.40	1	1.00	1	1	1	1	0	0	1	1	0	0	1
1	1	70	1	165	65.0	-1.10	23.9	111	61	182	79	103.00	6.10	1	1.00	0	0	1	0	1	1	1	1	0	1	1
1	1	64	2	148	38.1	-1.10	17.3	87	59	156	61	95.00	5.40	1	1.00	1	1	1	0	1	1	1	1	1	1	1
1	1	34	1	176	61.9	-2.90	19.9	104	62	217	63	154.00	4.90	1	1.00	1	1	0	1	1	1	1	1	1	1	1
1	1	64	2	151	45.8	.40	20.0	97	52	140	52	88.00	5.90	1	1.00	1	1	1	0	0	1	1	1	1	1	1
0	0	77	1	153	46.1	4.40	19.7	127	66	138	58	80.00	5.70	1	1.00	0	0	1	0	1	1	1	1	0	0	0
1	1	76	1	158	59.6	-.80	23.9	107	65	151	29	122.00	5.30	1	1.00	1	1	1	1	0	0	1	1	1	0	1
1	1	32	2	155	45.0	1.50	18.6	109	63	180	79	101.00	5.40	1	1.00	1	0	1	1	1	0	1	1	1	1	1
1	1	63	2	154	47.5	.20	20.0	133	77	236	78	158.00	5.40	1	1.00	1	1	1	0	0	0	0	1	1	0	1
1	1	68	1	164	63.1	-.60	23.3	163	81	128	43	85.00	5.40	1	1.00	1	0	1	1	1	1	1	1	0	1	1
1	1	66	2	139	45.2	2.50	23.2	112	66	229	54	175.00	6.20	1	1.00	1	0	1	1	1	1	1	1	1	0	0
0	0	57	2	150	47.3	5.10	21.0	122	83	261	64	197.00	5.30	1	1.00	1	1	1	0	1	0	0	1	1	0	1
0	0	49	1	171	80.5	-.40	27.4	116	69	129	38	91.00	5.60	0	1.00	0	0	1	0	1	1	1	0	1	0	1
0	0	75	1	155	56.6	3.10	23.7	121	65	224	64	160.00	5.90	1	1.00	0	1	1	0	1	1	1	1	0	0	1
1	1	72	1	159	49.5	.60	19.6	118	67	156	58	98.00	5.50	1	1.00	0	1	1	0	1	0	0	1	1	0	1
1	1	45	1	180	90.6	5.50	28.0	112	74	203	43	160.00	5.70	1	1.00	1	1	1	1	1	0	1	0	1	1	1
1	1	61	2	153	57.6	-1.90	24.6	99	63	229	47	182.00	5.10	1	1.00	1	0	1	0	0	0	0	1	1	0	1
0	0	51	2	163	69.2	3.40	26.2	118	70	194	61	133.00	5.70	0	1.00	1	1	1	0	1	0	0	0	1	1	1
1	1	81	2	142	47.1	3.40	23.4	139	74	168	66	102.00	5.50	1	1.00	1	1	1	1	1	1	1	1	1	1	1
1	1	48	1	164	74.0	.40	27.6	119	84	262	56	206.00	5.90	1	1.00	1	0	1	0	1	0	1	0	1	0	1
1	1	56	2	156	62.9	1.00	25.9	137	81	289	71	218.00	6.00	1	1.00	1	1	1	1	1	1	1	0	1	0	1
1	1	62	1	167	52.5	-.60	18.8	127	78	168	84	84.00	5.80	1	1.00	1	0	1	1	1	0	1	1	1	1	1
1	1	53	1	162	62.2	1.90	23.6	118	81	203	61	142.00	5.00	1	1.00	0	1	1	1	1	0	1	1	1	1	1
1	1	72	2	153	64.6	1.70	27.7	115	64	227	58	169.00	5.70	1	1.00	1	1	1	1	1	1	1	0	1	0	1
1	1	47	2	155	46.0	1.80	19.1	108	61	213	37	176.00	5.30	1	1.00	1	0	0	0	0	0	1	1	1	0	1

1	1	60	2	157	45.7	-.10	18.4	117	64	215	82	133.00	5.40	1	1.00	0	0	1	1	1	1	1	1	0	1	1
1	1	59	2	147	51.0	-2.80	23.6	161	94	279	67	212.00	6.00	1	1.00	1	0	1	1	1	1	1	1	0	0	1
0	0	62	2	149	50.2	1.70	22.5	114	69	209	64	145.00	5.50	1	1.00	1	0	1	0	1	0	0	1	0	1	1
0	0	54	1	171	66.0	1.10	22.5	131	85	205	97	108.00	4.90	0	1.00	0	0	0	0	1	1	0	1	1	1	1
1	1	58	2	153	58.4	3.80	24.9	120	65	182	80	102.00	5.50	1	1.00	1	0	1	0	0	0	0	1	0	0	1
1	1	44	1	169	53.3	.80	18.7	124	70	152	59	93.00	5.20	1	1.00	1	1	0	0	1	0	1	1	1	1	1
1	1	49	2	160	56.8	-3.90	22.1	127	78	242	92	150.00	5.20	0	1.00	0	1	0	0	0	0	0	1	0	0	1
0	0	67	1	165	65.2	3.70	24.0	104	68	220	62	158.00	5.90	1	1.00	0	1	1	0	0	1	1	1	1	0	1
1	1	66	1	163	73.0	.40	27.4	133	79	194	54	140.00	5.80	1	1.00	0	1	1	0	1	1	1	0	0	1	1
1	1	56	2	148	60.6	.60	27.5	121	77	222	52	170.00	5.80	1	1.00	1	1	1	1	1	1	1	0	0	0	1
0	0	28	1	168	78.9	-.80	28.0	150	86	215	89	126.00	5.50	1	1.00	0	1	1	0	1	0	0	0	0	1	1
1	1	74	1	161	76.4	-6.60	29.5	123	69	215	129	86.00	5.60	1	1.00	1	1	1	1	0	0	0	0	1	1	1
0	1	61	1	174	67.9	2.30	22.5	141	89	200	53	147.00	5.70	1	1.00	0	0	1	1	0	0	1	1	0	1	1
0	0	43	2	153	46.7	-.40	19.8	120	73	175	80	95.00	5.20	1	1.00	0	1	1	1	1	0	1	1	1	1	1
1	1	41	1	166	69.1	.40	25.1	111	65	234	50	184.00	5.80	1	1.00	0	1	1	0	1	0	0	0	1	0	1
1	1	56	1	173	75.7	.70	25.3	108	72	260	126	134.00	5.70	1	1.00	0	0	1	1	1	1	0	0	1	0	1
1	1	69	2	147	59.5	.20	27.4	134	70	201	43	158.00	5.80	1	1.00	1	0	1	0	1	1	1	0	1	1	1
0	0	45	2	162	50.2	-2.30	19.1	117	78	176	57	119.00	5.10	0	1.00	1	1	1	1	0	0	1	1	1	1	1
1	1	61	1	164	62.7	-4.60	23.4	119	77	188	54	134.00	5.50	1	1.00	1	1	1	0	1	1	1	1	1	1	1
0	0	28	2	162	76.5	10.40	29.1	115	71	215	102	113.00	5.10	1	1.00	1	0	1	0	0	1	1	0	1	1	1
1	1	63	2	151	63.5	-.40	27.9	107	63	228	100	128.00	5.60	1	1.00	1	0	1	1	1	1	1	0	1	0	1
1	1	52	2	157	62.1	-.60	25.0	144	74	207	82	125.00	5.70	1	1.00	1	0	1	1	1	1	0	0	0	1	1
1	1	60	2	154	58.6	1.50	24.6	101	65	196	82	114.00	5.30	1	1.00	1	1	1	1	1	0	0	1	0	1	1
1	1	63	2	148	49.9	1.50	22.8	127	73	182	52	130.00	5.50	1	1.00	1	1	1	1	1	1	1	1	0	1	1
1	1	73	2	145	49.4	.30	23.4	112	54	211	73	138.00	5.60	1	1.00	1	1	1	1	1	1	1	1	1	1	1
1	1	40	1	178	66.3	1.30	20.8	96	65	243	74	169.00	5.30	1	1.00	0	1	1	1	0	1	1	1	1	0	1
1	1	56	2	152	45.6	1.00	19.7	129	77	242	80	162.00	5.40	1	1.00	1	0	1	0	0	1	1	1	1	0	1
0	0	56	2	146	56.0	-1.60	26.2	95	58	249	71	178.00	5.50	1	1.00	1	0	1	0	1	1	1	0	1	0	1
0	0	27	1	181	107.1	-.90	32.6	142	77	207	46	161.00	4.90	0	1.00	0	1	1	0	1	0	0	0	0	1	1
1	1	56	2	159	64.6	2.20	25.4	123	67	201	63	138.00	5.40	1	1.00	0	0	1	0	1	1	1	0	0	1	1
0	0	62	2	156	62.4	5.30	25.5	107	72	174	69	105.00	5.60	1	1.00	1	1	1	0	1	0	1	0	0	1	1
1	1	49	1	170	60.2	1.30	20.8	153	96	192	96	96.00	4.80	1	1.00	0	0	1	0	1	0	1	1	0	1	1
0	0	72	2	145	49.3	-1.30	23.3	113	66	191	69	122.00	5.10	1	1.00	1	1	1	1	0	1	1	1	0	1	1
0	0	71	2	152	50.6	2.60	21.9	105	57	181	75	106.00	5.60	1	1.00	1	1	1	0	1	1	1	1	1	1	1
0	0	72	2	143	49.0	.40	23.9	137	82	177	61	116.00	6.00	1	1.00	1	0	1	1	1	0	0	1	0	1	1
0	0	77	2	151	41.4	3.00	18.1	117	70	177	64	113.00	5.50	1	1.00	1	0	1	0	1	0	0	1	0	1	1
0	0	53	1	166	70.7	.10	25.6	124	71	167	48	119.00	7.90	0	1.00	1	1	1	1	1	1	1	0	1	1	0
1	1	48	1	180	72.5	.80	22.4	146	85	230	52	178.00	5.60	0	1.00	0	1	1	1	1	0	1	1	0	0	1
0	0	54	2	160	53.5	-.70	21.0	121	84	217	100	117.00	5.40	0	1.00	0	1	1	0	1	1	1	1	0	1	1

0	0	58	2	148	64.2	-1.20	29.4	106	73	197	63	134.00	5.40	1	1.00	1	0	1	0	1	1	0	0	1	1	1
0	0	64	1	165	69.8	-.10	25.5	135	83	258	50	208.00	5.40	1	1.00	1	0	1	0	1	1	1	0	1	0	1
0	0	61	2	159	54.1	2.20	21.3	106	75	195	71	124.00	5.80	1	1.00	0	0	1	0	0	0	0	1	0	1	1
1	1	60	1	171	70.9	1.80	24.1	152	86	169	46	123.00	5.40	1	1.00	0	0	1	0	0	1	0	1	0	1	1
1	1	41	2	162	59.8	4.60	22.8	92	60	200	75	125.00	5.40	0	1.00	1	0	1	0	1	0	0	1	1	1	1
0	0	72	2	151	60.8	.80	26.6	144	78	224	74	150.00	5.80	1	1.00	1	1	1	0	1	1	1	0	0	0	1
1	1	62	2	144	42.6	-.40	20.6	116	72	236	59	177.00	5.50	1	1.00	1	1	1	1	1	1	1	1	1	0	1
0	0	61	2	154	47.6	-2.40	20.0	116	76	195	57	138.00	5.50	1	1.00	1	1	1	1	1	0	0	1	1	1	1
0	0	84	2	153	64.2	.80	27.3	129	65	221	45	176.00	5.30	1	1.00	1	0	1	1	1	1	0	0	0	0	1
0	1	39	2	158	68.1	-.20	27.2	98	63	178	59	119.00	5.50	1	1.00	0	0	1	0	0	0	0	0	1	1	1
1	1	61	2	155	59.9	-.90	24.8	104	58	279	96	183.00	5.10	1	1.00	1	0	1	1	1	1	0	1	1	0	1
1	1	49	2	159	52.0	-5.80	20.5	125	78	188	73	115.00	5.70	0	1.00	0	0	1	0	1	0	1	1	1	1	1
1	1	56	2	155	57.9	3.50	24.2	150	96	210	61	149.00	6.10	1	1.00	1	1	1	1	1	0	1	1	0	1	1
0	0	23	2	162	58.1	4.30	22.1	98	56	183	61	122.00	5.60	1	1.00	1	1	1	0	1	1	1	1	1	1	1
0	0	55	2	157	56.6	-.70	22.9	125	77	219	78	141.00	6.20	1	1.00	0	1	1	0	1	1	0	1	1	1	1
1	1	48	2	154	49.5	-1.10	20.8	127	75	241	95	146.00	5.30	1	1.00	0	1	1	0	1	1	1	1	1	0	1
0	0	42	1	167	87.4	-9.00	31.3	114	66	236	52	184.00	5.30	0	1.00	1	0	0	0	0	1	1	0	1	0	1
1	1	64	1	157	61.5	-1.50	24.9	123	66	201	67	134.00	5.20	1	1.00	0	1	1	1	1	1	1	1	1	1	1
1	1	42	1	159	75.8	-2.10	29.8	143	91	293	39	254.00	5.80	0	1.00	0	0	1	1	1	0	0	0	0	0	1
1	1	61	2	155	66.5	.60	27.6	149	82	221	77	144.00	5.70	1	1.00	1	0	1	1	1	0	0	0	0	0	1
1	1	67	2	145	69.6	-.80	33.1	122	71	204	74	130.00	5.60	1	1.00	1	0	1	1	1	1	1	0	1	1	1
1	1	73	2	141	58.5	-2.20	29.5	115	69	123	60	63.00	5.30	1	1.00	1	1	1	1	1	0	0	0	0	0	1
1	1	70	1	165	67.0	-2.20	24.5	136	74	192	56	136.00	5.10	1	1.00	0	0	1	1	1	1	1	1	1	1	1
1	1	59	1	167	66.4	-.40	23.9	135	76	199	37	162.00	7.20	0	2.00	0	1	1	0	1	1	1	1	1	0	0
1	1	66	2	142	43.3	-1.50	21.4	97	65	165	53	112.00	5.40	1	1.00	1	1	1	0	1	1	1	1	1	1	1
1	1	71	2	150	58.3	3.70	25.8	114	75	218	45	173.00	5.80	1	1.00	1	0	1	0	1	0	0	0	0	1	1
0	0	73	2	146	39.2	-1.70	18.4	126	74	145	42	103.00	5.50	1	1.00	1	0	1	0	1	1	1	1	1	1	1
1	1	48	2	160	44.6	-.70	17.3	106	67	160	69	91.00	5.30	1	1.00	1	0	1	0	0	0	1	1	1	1	1
0	0	74	1	154	64.9	2.50	27.2	162	83	155	54	101.00	5.40	1	1.00	0	0	1	0	0	1	0	0	0	1	1
1	1	70	1	153	63.2	.10	27.0	106	58	143	32	111.00	5.90	1	1.00	1	0	1	0	1	0	0	0	1	0	1
1	1	77	2	141	48.0	-1.60	24.2	134	62	218	53	165.00	6.90	1	1.00	1	0	1	1	1	0	1	1	0	1	0
1	1	61	2	144	55.7	-1.10	26.7	136	81	202	73	129.00	5.50	1	1.00	1	0	0	1	1	1	0	0	1	1	1
1	1	45	1	166	58.7	-2.40	21.2	130	83	187	60	127.00	5.30	0	1.00	0	1	0	1	0	1	0	1	1	1	1
1	1	74	2	146	41.0	3.00	19.2	98	54	163	75	88.00	5.70	1	1.00	1	0	1	0	0	0	0	1	0	0	1
1	1	49	2	151	45.5	-1.00	19.9	149	101	203	83	120.00	5.70	1	1.00	1	0	1	0	1	0	0	1	0	1	1
1	1	63	1	162	65.6	-3.90	25.0	110	59	221	48	173.00	6.30	1	1.00	1	0	1	0	0	1	1	0	1	0	1
1	1	45	2	151	58.3	.50	25.4	114	66	196	58	138.00	6.10	1	1.00	1	1	1	1	1	0	1	0	1	1	1
0	0	50	2	152	69.3	.70	29.8	127	76	252	63	189.00	5.60	1	1.00	1	1	1	0	1	0	0	0	0	0	1
1	1	62	1	165	79.6	1.00	29.1	119	80	200	46	154.00	5.50	1	1.00	0	0	1	1	1	1	1	0	0	1	1

1	1	30	1	168	80.2	3.50	28.3	106	59	192	39	153.00	5.50	1	1.00	1	0	1	0	1	0	0	0	1	0	1
1	1	40	1	172	127.5	-5.70	42.8	146	91	192	52	140.00	5.90	1	1.00	1	0	1	0	1	1	1	0	0	1	1
0	1	73	1	159	56.7	-.10	22.3	119	66	143	41	102.00	7.30	1	1.00	0	0	1	1	1	1	1	1	1	1	0
0	0	81	2	149	55.1	-.20	24.8	166	86	241	58	183.00	6.00	1	1.00	0	0	1	1	1	1	1	1	0	0	1
1	1	66	1	160	62.8	-.70	24.4	130	80	147	71	76.00	5.70	1	1.00	0	1	1	1	1	1	1	1	0	1	1
1	1	74	1	163	61.2	-.60	22.9	113	73	208	61	147.00	5.60	1	1.00	0	1	1	1	1	0	0	1	1	1	1
1	1	47	2	150	54.8	-.50	24.3	98	65	202	45	157.00	4.90	1	1.00	1	0	1	0	0	0	0	1	1	1	1
0	0	59	2	148	51.0	3.60	23.2	106	71	216	47	169.00	5.60	1	1.00	1	0	1	1	0	0	0	1	1	1	1
1	1	59	1	171	75.1	6.10	25.7	117	76	183	69	114.00	6.50	1	1.00	0	1	0	0	0	0	0	0	0	1	0
1	1	64	2	146	40.4	3.00	18.9	104	70	206	90	116.00	5.50	1	1.00	1	0	1	1	1	1	1	1	1	1	1
1	1	67	2	146	50.5	-6.80	23.8	106	75	166	60	106.00	5.80	1	1.00	1	0	1	1	1	1	1	1	1	1	1
1	1	73	1	166	54.9	.20	19.8	132	77	166	60	106.00	6.10	1	1.00	0	0	1	1	1	1	1	1	0	1	0
1	1	69	2	148	44.4	-.20	20.3	132	76	205	64	141.00	5.40	1	1.00	1	0	1	0	1	1	1	1	0	1	1
1	1	43	2	158	52.7	12.50	21.2	105	66	177	69	108.00	11.90	1	1.00	1	0	1	0	1	0	1	1	1	1	0
1	1	63	2	158	58.1	.50	23.2	88	50	254	59	195.00	5.70	1	1.00	1	0	1	1	1	1	0	1	1	0	1
1	1	35	2	156	50.4	2.20	20.7	104	62	184	66	118.00	5.50	1	1.00	1	1	1	0	1	0	1	1	1	1	1
1	1	63	1	176	68.5	.40	22.0	122	89	200	56	144.00	5.60	1	1.00	0	0	1	1	1	1	1	1	1	1	1
1	1	39	1	181	86.5	4.40	26.3	130	72	187	43	144.00	5.20	1	1.00	1	0	0	1	0	1	1	0	1	1	1
1	1	55	2	153	73.7	.00	31.6	128	75	286	72	214.00	5.90	1	1.00	1	0	1	0	1	0	0	0	0	0	1
1	1	51	2	161	73.6	5.10	28.5	118	77	209	77	132.00	5.50	1	1.00	0	0	1	0	1	1	1	0	1	1	1
1	1	49	1	176	78.6	-1.80	25.4	110	76	184	37	147.00	5.50	1	1.00	0	1	1	0	1	1	1	0	1	0	1
1	1	70	2	156	57.3	-.70	23.6	120	74	210	45	165.00	5.70	1	1.00	1	0	1	0	1	0	0	1	1	1	1
1	1	73	2	141	40.3	2.00	20.3	127	81	219	99	120.00	5.40	1	1.00	0	0	1	0	1	0	0	1	0	1	1
1	1	74	1	162	61.6	-2.20	23.3	120	74	151	58	93.00	5.70	0	1.00	0	0	1	1	1	1	1	1	0	1	1
1	1	44	1	167	55.2	1.30	19.8	105	73	241	56	185.00	5.60	1	1.00	1	0	1	0	1	1	0	1	1	0	1
1	1	30	2	148	60.8	-7.90	27.9	106	65	201	53	148.00	5.10	1	1.00	1	1	1	1	1	1	1	0	1	1	1
0	0	55	1	165	61.2	.50	22.4	103	64	242	83	159.00	5.50	0	1.00	0	1	1	1	1	1	1	1	1	0	1
0	0	64	2	149	61.0	.20	27.5	121	75	241	53	188.00	5.40	1	1.00	0	1	0	1	1	0	0	0	0	0	1
1	1	57	2	142	46.5	-.80	23.1	114	74	221	68	153.00	5.60	1	1.00	0	0	1	1	1	0	0	1	1	0	1
1	1	71	2	145	55.5	-1.50	26.5	126	77	160	52	108.00	6.20	1	1.00	1	0	1	1	1	1	1	0	0	0	1
1	1	69	2	146	41.1	4.20	19.3	122	74	236	98	138.00	5.90	1	1.00	1	1	1	1	1	1	1	1	1	0	1
1	0	45	2	154	43.4	-1.80	18.2	87	49	259	65	194.00	5.60	0	1.00	0	0	0	0	1	0	0	1	1	0	1
0	0	55	1	176	76.0	8.40	24.6	139	86	199	45	154.00	5.80	0	1.00	0	0	1	0	1	1	0	1	0	1	1
1	1	49	1	169	67.2	-2.40	23.5	98	55	252	40	212.00	5.80	0	1.00	1	1	1	0	1	1	1	1	1	0	1
1	0	69	1	161	52.6	4.90	20.1	97	67	204	56	148.00	12.00	1	1.00	0	0	1	0	0	0	1	1	1	1	0
1	1	82	2	140	43.6	5.00	22.3	108	68	195	49	146.00	9.40	1	1.00	1	0	1	1	1	0	1	1	1	1	0
1	1	55	1	168	75.7	-.40	26.8	141	86	233	68	165.00	6.30	0	1.00	0	1	1	0	0	0	1	0	0	0	1
1	1	81	2	153	47.8	-.50	20.3	100	53	284	88	196.00	6.00	1	1.00	1	0	1	0	1	0	0	1	1	0	1
0	0	64	2	148	52.4	-.20	23.8	143	72	224	46	178.00	7.30	1	1.00	1	1	1	0	0	1	0	1	0	0	0

1	1	49	1	168	70.8	.30	25.0	104	61	210	44	166.00	5.40	0	1.00	0	1	1	1	1	1	1	0	1	1	1
1	1	69	2	141	48.7	.60	24.3	119	71	206	47	159.00	6.20	1	1.00	1	1	1	1	0	1	1	1	0	1	1
1	1	47	1	165	66.4	-1.20	24.3	116	77	216	45	171.00	5.30	1	1.00	0	1	1	1	1	1	1	1	1	1	1
1	1	66	2	155	50.7	-3.00	21.0	134	90	269	82	187.00	5.90	1	1.00	1	0	1	1	1	1	1	1	0	0	1
0	0	79	2	152	50.9	1.20	22.1	119	75	258	60	198.00	5.60	1	1.00	1	1	1	1	1	1	0	1	1	0	1
1	1	50	2	157	64.7	6.20	26.3	115	76	216	55	161.00	5.40	1	1.00	1	0	1	0	0	0	0	0	1	1	1
1	1	54	1	167	56.6	-1.70	20.2	137	89	155	46	109.00	5.90	0	1.00	0	1	1	1	1	1	0	1	1	0	1
1	1	61	2	155	52.3	.00	21.8	92	58	253	66	187.00	5.70	1	1.00	1	0	1	0	0	1	0	1	1	0	1
0	0	61	2	156	57.1	6.30	23.4	102	67	226	58	168.00	6.20	1	1.00	0	0	1	0	0	0	0	1	1	0	1
1	1	75	2	149	42.5	1.50	19.1	132	73	156	73	83.00	6.10	1	1.00	1	0	1	0	1	1	1	1	1	1	1
1	1	70	2	148	46.5	-2.00	21.2	144	94	236	64	172.00	5.20	1	1.00	1	0	1	0	1	0	0	1	0	0	1
1	1	59	2	153	46.1	1.40	19.7	100	64	201	76	125.00	5.50	1	1.00	1	0	1	1	1	1	0	1	1	1	1
1	1	66	1	174	63.6	-2.00	21.0	126	73	230	69	161.00	6.10	1	1.00	0	1	1	1	1	1	1	1	1	0	1
1	1	58	2	157	57.4	-3.30	23.3	107	64	196	71	125.00	5.60	1	1.00	1	0	1	1	0	0	0	1	1	1	1
1	1	61	1	166	68.1	1.90	24.7	124	71	192	53	139.00	5.70	1	1.00	0	0	1	0	1	1	1	1	0	1	1
1	1	53	2	159	49.2	.40	19.3	158	85	246	112	134.00	5.20	1	1.00	1	0	1	1	1	1	1	1	0	0	1
1	1	65	1	170	71.5	1.90	24.8	141	83	185	40	145.00	5.60	1	1.00	1	1	1	1	1	0	1	1	0	1	1
1	1	74	1	160	72.2	-1.80	28.3	143	82	165	48	117.00	6.70	0	1.00	0	0	0	1	1	1	1	0	0	1	0
1	1	83	2	149	45.8	-.30	20.7	116	52	229	75	154.00	5.70	1	1.00	1	0	1	1	1	1	1	1	1	0	1
1	1	56	1	175	75.0	.40	24.4	146	91	237	52	185.00	5.80	1	1.00	0	0	1	0	0	0	0	1	0	0	1
1	1	58	1	162	61.9	.60	23.5	115	73	155	66	89.00	5.20	1	1.00	0	0	1	1	1	1	1	1	1	1	1
1	1	83	1	170	76.5	-1.20	26.4	106	58	157	69	88.00	5.40	1	1.00	1	0	1	1	1	1	1	0	1	1	1
0	0	30	2	156	66.8	7.80	27.4	117	67	227	68	159.00	5.10	0	1.00	0	0	0	0	1	0	0	0	1	0	1
1	1	59	2	154	53.0	.00	22.2	138	80	256	66	190.00	5.60	1	1.00	1	0	1	1	0	1	1	1	1	0	1
1	1	40	2	156	55.4	1.90	22.6	99	59	184	73	111.00	5.40	1	1.00	1	1	1	0	1	0	1	1	1	1	1
1	1	29	2	153	37.3	-.90	16.0	116	63	166	63	103.00	5.60	1	1.00	1	0	1	0	1	1	1	1	1	1	1
1	1	63	1	165	73.7	2.10	27.1	106	69	194	59	135.00	5.90	1	1.00	0	1	1	0	1	0	0	0	1	1	1
1	1	57	2	147	58.4	1.80	27.0	119	80	186	82	104.00	5.60	1	1.00	1	1	1	0	1	1	1	0	1	1	0
0	0	50	1	166	75.1	-.20	27.2	105	68	235	36	199.00	5.50	1	1.00	0	0	1	0	1	1	1	0	1	0	1
1	1	52	2	157	52.8	.70	21.3	112	63	244	94	150.00	5.20	0	1.00	1	0	1	0	1	0	0	1	1	0	1
1	1	61	2	156	52.2	2.10	21.4	110	64	242	54	188.00	5.70	1	1.00	1	0	1	0	1	0	1	1	1	0	1
1	1	68	2	154	53.7	.00	22.6	137	82	218	58	160.00	5.40	1	1.00	0	0	0	1	1	0	0	1	0	1	1
1	1	76	1	155	66.6	-2.50	27.8	152	77	212	33	179.00	5.90	0	1.00	0	0	1	1	1	1	1	0	0	0	1
1	1	37	2	156	74.6	.00	30.6	113	64	245	67	178.00	5.50	1	1.00	1	0	1	0	1	1	1	0	1	0	1
0	0	43	1	176	76.6	4.50	24.7	127	71	211	126	85.00	5.90	1	1.00	0	0	1	1	1	1	1	1	1	1	1
1	1	76	2	135	51.1	1.60	27.9	111	63	157	57	100.00	6.40	1	1.00	1	0	1	1	1	0	1	0	0	0	1
1	1	71	1	165	50.1	-3.60	18.4	101	60	179	81	98.00	5.60	0	1.00	0	1	1	0	1	1	1	1	1	1	1
1	1	74	2	154	55.2	.20	23.3	121	75	195	46	149.00	5.70	1	1.00	1	1	1	1	1	1	1	1	0	1	1
1	1	62	2	152	49.1	-.40	21.3	132	69	164	62	102.00	5.30	1	1.00	1	1	1	0	1	0	0	1	0	1	1

1	1	40	2	151	48.0	-.90	21.1	107	73	236	67	169.00	5.30	1	1.00	1	1	1	0	1	0	0	1	1	0	1
1	1	76	1	161	47.9	-.50	18.5	110	70	222	66	156.00	5.20	1	1.00	1	0	1	1	1	1	1	1	1	0	1
1	1	64	2	155	50.4	-3.40	21.0	97	55	190	78	112.00	6.30	1	1.00	1	1	1	1	1	1	1	1	1	0	0
0	0	50	2	150	60.3	8.40	26.7	118	62	205	70	135.00	5.30	0	1.00	1	1	1	0	1	0	0	0	1	1	1
1	1	67	1	152	61.7	.00	26.7	129	81	195	62	133.00	5.70	1	1.00	0	0	1	1	1	1	1	0	0	1	0
0	0	69	2	143	63.8	8.50	31.2	133	75	218	38	180.00	5.70	1	1.00	1	1	1	0	1	1	1	0	1	0	1
0	0	79	2	150	68.8	.40	30.7	121	65	139	49	90.00	6.00	1	1.00	1	0	1	0	1	0	0	0	0	1	1
1	1	58	1	168	67.1	-.60	23.6	116	76	201	56	145.00	5.50	0	1.00	0	1	1	0	1	1	1	1	1	1	1
1	1	61	2	142	49.7	-3.80	24.5	118	69	187	55	132.00	6.50	1	1.00	1	1	1	1	1	1	1	1	0	0	0
1	1	65	2	142	53.5	-.70	26.6	106	58	229	62	167.00	5.80	1	1.00	1	0	1	1	1	0	1	0	1	0	1
1	1	58	2	148	47.5	.50	21.7	89	56	198	81	117.00	5.30	0	2.00	0	0	0	1	1	0	1	1	1	1	1
1	1	64	2	150	59.8	1.40	26.5	119	76	215	76	139.00	6.20	1	1.00	1	0	1	1	1	0	0	0	1	0	1
1	1	63	1	171	68.5	-1.50	23.4	142	95	234	59	175.00	5.40	0	1.00	0	1	1	1	1	1	1	1	0	0	1
1	1	58	2	148	61.4	-1.90	27.9	121	67	206	57	149.00	5.70	1	1.00	1	1	1	0	1	1	1	0	1	1	1
0	0	52	1	176	74.3	1.30	24.0	124	80	213	52	161.00	5.80	1	1.00	0	1	1	0	1	1	1	1	1	1	1
1	1	67	1	162	59.7	-.70	22.6	124	79	195	40	155.00	6.10	0	1.00	1	0	1	0	0	1	0	1	1	1	1
0	0	70	2	155	54.7	1.00	22.8	133	81	194	46	148.00	5.70	1	1.00	1	1	1	1	1	0	1	1	0	0	1
0	0	65	1	163	58.2	6.10	21.8	116	74	191	42	149.00	5.30	0	1.00	1	1	1	1	0	1	1	1	1	1	1
1	1	62	2	145	37.8	.20	18.0	105	62	233	76	157.00	6.10	1	1.00	1	0	1	0	0	0	0	1	1	0	1
1	1	75	1	158	52.1	1.50	20.9	120	79	206	72	134.00	6.20	1	1.00	0	1	1	0	1	1	1	1	1	1	1
1	1	64	1	159	58.9	.50	23.2	161	66	185	43	142.00	7.30	0	1.00	0	0	1	0	0	1	1	1	0	1	0
1	1	68	1	161	65.5	-.10	25.1	137	91	154	42	112.00	6.40	1	1.00	1	0	1	0	0	1	1	0	0	1	1
1	1	70	1	160	69.0	2.30	27.0	135	78	251	47	204.00	6.30	1	1.00	1	0	1	1	1	1	1	0	0	0	1
0	0	72	1	154	60.2	2.50	25.3	135	79	180	65	115.00	5.60	0	1.00	0	0	0	0	0	0	0	0	0	1	1
0	0	69	2	154	53.9	1.70	22.8	110	74	231	74	157.00	6.50	1	1.00	1	0	1	0	0	0	0	1	0	0	0
1	1	83	2	139	52.0	.00	26.8	109	59	193	60	133.00	5.20	1	1.00	1	0	1	0	0	1	1	0	0	1	1
1	1	66	2	148	50.2	2.90	22.8	133	81	224	71	153.00	5.80	1	1.00	1	1	1	0	1	1	1	1	1	0	1
0	0	69	1	162	54.6	5.40	20.8	132	79	199	29	170.00	5.20	1	1.00	0	0	1	0	0	0	0	1	0	0	1
1	1	56	2	143	38.7	-.70	18.8	102	62	256	79	177.00	5.60	1	1.00	1	1	1	1	1	1	1	1	1	0	1
1	1	67	2	144	57.4	.40	27.7	109	69	159	51	108.00	5.40	1	1.00	1	0	1	1	1	1	1	0	0	1	1
0	0	62	2	145	54.2	1.50	25.8	106	66	219	97	122.00	5.80	1	1.00	1	0	1	0	1	0	0	0	1	1	1
1	1	75	2	149	45.5	-.50	20.6	88	56	218	55	163.00	5.30	1	1.00	1	1	1	0	1	0	0	1	1	1	1
1	1	50	1	167	59.3	1.70	21.3	111	70	161	42	119.00	5.40	0	1.00	1	0	1	0	0	0	1	1	1	1	1
1	1	72	1	157	66.4	.80	26.9	158	92	251	43	208.00	5.70	1	1.00	0	1	1	1	1	1	1	0	0	0	1
1	1	68	1	160	57.9	1.80	22.6	107	55	154	44	110.00	7.30	0	1.00	0	0	1	1	1	1	1	1	1	0	0
1	1	63	2	149	44.6	-.70	20.1	108	76	262	75	187.00	5.40	1	1.00	1	0	1	0	1	1	1	1	1	0	1
1	1	77	1	156	64.0	.90	26.3	125	69	188	59	129.00	5.30	1	1.00	0	0	1	1	1	1	1	0	0	1	1
1	1	73	2	147	55.5	-.30	25.7	125	66	198	54	144.00	5.60	1	1.00	1	0	1	1	1	0	0	0	0	0	1
1	1	68	1	153	58.2	-.80	25.0	135	81	198	69	129.00	5.40	1	1.00	0	1	1	1	1	1	1	0	1	1	1

1	1	59	2	155	53.8	2.30	22.3	114	65	220	63	157.00	5.90	1	1.00	1	1	1	0	1	1	1	1	1	0	1
1	1	67	1	170	59.3	4.30	20.6	106	59	178	56	122.00	5.90	1	1.00	0	1	1	0	1	1	0	1	1	1	1
0	0	70	2	140	62.5	-4.30	31.7	122	72	162	59	103.00	5.40	1	1.00	1	1	1	0	0	1	1	0	1	1	1
0	0	71	2	145	40.3	1.60	19.0	128	67	246	64	182.00	5.60	1	1.00	1	0	1	1	0	0	0	1	1	0	1
0	0	81	2	153	50.8	1.40	21.7	110	62	238	57	181.00	5.90	1	1.00	1	1	1	1	0	1	1	1	1	0	1
0	0	47	2	150	45.1	2.80	20.1	134	76	191	69	122.00	5.30	1	1.00	0	0	1	0	0	1	1	1	1	1	1
1	1	62	2	144	49.7	-.90	23.8	102	55	255	63	192.00	5.50	1	1.00	1	1	0	0	0	0	0	1	1	0	1
1	1	63	1	161	60.1	6.40	23.3	133	80	220	31	189.00	5.00	1	1.00	0	0	1	1	0	1	1	1	0	0	1
1	1	58	2	156	47.6	2.00	19.5	139	75	237	69	168.00	5.30	1	1.00	1	0	1	0	1	0	1	1	1	0	1
0	0	52	2	159	75.0	3.90	29.6	134	68	204	70	134.00	6.80	0	1.00	0	0	1	0	1	1	0	0	0	0	0
1	1	60	1	176	75.3	-.20	24.3	151	91	271	63	208.00	5.60	1	1.00	0	0	1	1	0	0	0	1	0	0	1
1	1	72	2	134	32.2	.50	17.8	117	70	207	62	145.00	5.30	1	1.00	1	1	1	1	1	1	1	1	1	1	1
0	0	74	2	144	60.7	-.20	29.3	125	73	155	61	94.00	5.40	1	1.00	1	0	1	0	1	0	0	0	0	0	1
0	0	59	1	166	77.0	4.30	28.0	137	75	238	78	160.00	5.40	1	1.00	0	1	1	0	1	1	1	0	1	0	1
0	0	71	2	160	64.7	2.40	25.2	117	64	218	84	134.00	5.50	1	1.00	0	0	1	0	1	1	1	0	0	0	1
0	0	71	2	148	61.6	2.40	28.3	111	64	237	75	162.00	5.80	1	1.00	1	1	1	0	1	1	0	0	0	0	1
0	1	48	1	156	56.1	3.80	23.0	105	67	164	42	122.00	5.40	1	1.00	1	1	1	1	0	1	1	1	1	1	1
1	1	56	1	162	57.1	-5.50	21.6	137	70	262	61	201.00	5.30	0	1.00	1	0	1	0	0	0	0	1	1	0	1
1	1	50	2	156	48.7	5.60	20.1	103	66	162	64	98.00	5.60	1	1.00	0	0	1	1	0	1	1	1	1	1	1
1	1	66	1	156	61.7	2.30	25.3	134	83	180	44	136.00	5.30	1	1.00	0	0	1	1	1	1	1	0	0	1	1
0	0	61	1	163	69.4	-1.40	26.0	158	91	215	47	168.00	5.20	1	1.00	0	1	1	1	1	1	1	0	0	1	1
0	0	64	1	165	67.0	-.40	24.4	120	79	236	97	139.00	6.80	0	1.00	0	1	1	0	0	0	0	1	0	0	0
0	0	73	1	173	70.0	-1.30	23.3	129	83	180	68	112.00	5.50	0	1.00	0	1	0	0	1	1	1	1	1	1	1
0	0	75	1	172	87.4	-2.50	29.4	122	67	196	41	155.00	7.00	1	1.00	0	0	1	1	0	1	0	0	0	1	0
1	1	77	1	157	60.2	.50	24.3	135	79	210	42	168.00	5.70	1	1.00	1	1	1	0	1	0	0	1	0	1	1
0	0	68	2	147	45.2	1.30	20.8	123	67	237	75	162.00	6.30	1	1.00	1	0	1	0	1	0	0	1	0	0	1
0	0	87	1	171	64.3	-.80	22.0	94	59	203	60	143.00	5.40	1	1.00	0	0	0	1	1	1	1	1	0	1	1
0	0	71	2	150	42.8	2.70	18.9	128	65	223	116	107.00	5.70	1	1.00	1	1	1	1	1	0	1	1	0	0	1
1	1	62	2	145	45.2	-1.60	21.3	99	63	267	68	199.00	5.40	1	1.00	1	1	1	0	1	0	1	1	1	0	1
1	1	69	1	154	49.8	-1.40	21.0	129	76	165	48	117.00	6.00	1	1.00	1	0	1	0	1	0	0	1	0	1	1
0	0	29	1	169	61.5	6.70	21.5	120	75	210	58	152.00	5.20	0	1.00	1	0	1	0	1	1	1	1	1	1	1
1	1	62	2	156	55.0	1.80	22.6	139	75	209	78	131.00	11.70	0	1.00	1	1	1	1	1	0	0	1	1	1	0
1	1	59	2	154	44.1	-.80	18.5	104	57	205	79	126.00	5.40	1	1.00	1	0	1	0	0	0	1	1	1	1	1
1	1	70	1	160	52.2	3.50	20.2	111	77	159	76	83.00	5.20	0	1.00	0	1	1	1	0	1	0	1	1	1	1
1	1	54	2	161	75.9	-.10	29.2	135	70	201	67	134.00	5.50	1	1.00	1	0	1	0	0	0	0	0	1	1	1
1	1	71	1	152	53.6	2.70	23.1	109	66	145	51	94.00	5.40	0	1.00	1	0	1	0	1	1	1	1	1	1	1
1	1	73	2	141	50.1	.00	25.0	119	78	209	50	159.00	5.40	1	1.00	1	1	1	1	1	1	1	0	0	1	1
0	0	51	2	149	49.2	-.30	22.0	93	59	264	69	195.00	5.50	1	1.00	1	0	0	0	0	0	0	1	1	0	1
1	1	39	2	159	53.5	4.40	21.1	106	75	259	98	161.00	5.40	0	1.00	0	0	0	0	1	0	0	1	1	0	1

0	0	74	2	142	47.4	-.20	23.5	129	62	198	57	141.00	5.50	1	1.00	1	1	1	0	0	1	1	1	0	0	1
1	1	64	1	176	88.0	-1.60	28.3	144	80	164	37	127.00	5.20	1	1.00	1	0	1	1	1	0	0	0	0	0	1
0	0	79	1	159	65.7	.80	26.0	146	82	187	55	132.00	5.90	1	1.00	1	1	1	1	1	1	1	0	0	1	1
1	1	57	2	144	56.7	1.20	27.3	150	86	299	82	217.00	5.70	1	1.00	1	1	0	0	1	0	1	0	0	0	1
0	0	54	2	160	51.0	4.70	19.9	100	55	267	62	205.00	5.40	1	1.00	0	0	1	1	1	1	1	1	1	0	1
1	1	23	2	149	66.0	3.00	29.6	111	73	184	68	116.00	5.70	1	1.00	0	1	0	1	1	0	0	0	1	1	1
1	1	59	2	150	65.1	.20	29.0	120	75	205	43	162.00	5.80	1	1.00	1	0	1	0	0	0	0	0	1	1	1
0	0	67	1	170	57.9	.80	20.0	127	72	202	73	129.00	5.40	1	1.00	0	1	1	1	1	0	0	1	0	1	1
0	0	59	1	170	82.6	6.10	28.4	106	78	135	40	95.00	5.40	1	1.00	1	0	1	0	1	1	0	0	0	1	0
1	1	40	2	166	73.3	-.70	26.6	125	73	223	69	154.00	5.70	1	1.00	1	1	1	0	1	1	1	0	1	0	1
1	1	40	1	175	50.6	-1.80	16.5	104	71	188	40	148.00	5.00	1	1.00	1	0	1	0	1	1	1	1	1	1	1
1	1	38	2	148	50.0	2.00	22.7	89	59	123	66	57.00	5.40	1	1.00	1	1	1	1	0	0	0	1	1	1	1
0	0	64	1	159	77.5	-1.10	30.5	126	89	248	42	206.00	5.80	1	1.00	0	0	1	0	0	0	0	0	0	0	1
1	1	34	1	169	69.7	5.10	24.4	112	70	240	69	171.00	5.40	0	1.00	0	0	0	0	1	0	1	1	1	0	1
1	1	22	1	176	76.7	-9.80	24.7	118	66	153	40	113.00	4.70	1	1.00	1	1	1	0	1	0	1	1	1	1	1
1	1	52	2	155	63.5	1.20	26.2	108	65	240	66	174.00	6.10	1	1.00	0	1	1	0	1	1	1	0	1	0	1
1	1	61	2	152	55.1	1.10	23.9	118	78	212	77	135.00	5.20	1	1.00	1	1	1	1	1	1	0	1	1	1	1
1	1	56	2	154	60.3	1.90	25.3	127	74	215	102	113.00	5.10	0	1.00	0	0	1	0	0	0	0	0	1	1	1
1	1	38	2	154	49.5	-1.10	20.9	105	65	165	69	96.00	5.50	1	1.00	0	1	1	1	1	1	1	1	1	1	1
1	1	61	2	152	63.7	.00	27.4	150	101	183	57	126.00	6.00	1	1.00	0	1	1	1	1	1	1	0	0	1	1
1	1	58	2	159	63.6	6.10	25.1	108	67	228	89	139.00	5.10	1	1.00	1	0	1	1	1	1	0	0	1	0	1
1	1	54	2	143	69.4	.10	34.0	153	91	231	75	156.00	5.40	1	1.00	0	1	1	0	1	1	1	0	0	0	1
1	1	62	2	157	57.9	.30	23.5	120	70	334	50	284.00	5.80	1	1.00	1	1	1	1	1	0	0	1	1	0	1
0	0	43	2	158	55.9	-1.40	22.3	101	60	191	66	125.00	5.50	1	1.00	0	1	0	0	1	1	1	1	1	1	1
1	1	23	2	165	49.6	-.60	18.2	104	65	205	112	93.00	5.20	0	1.00	0	0	0	0	1	1	1	1	1	1	1
0	0	50	2	157	57.3	1.70	23.1	135	86	227	99	128.00	5.30	1	1.00	1	0	1	0	1	0	0	1	1	0	1
0	1	69	2	154	41.5	5.00	17.5	122	79	193	63	130.00	5.50	1	1.00	1	1	1	1	1	1	1	1	0	1	1
1	1	57	2	149	62.2	-.30	28.1	112	62	260	71	189.00	5.50	1	1.00	1	1	0	0	1	0	1	0	1	0	1
0	0	53	1	174	73.6	-.70	24.3	128	82	190	59	131.00	4.90	0	1.00	0	0	1	0	1	0	1	1	1	1	1
0	1	60	1	174	81.6	-1.70	26.9	125	82	231	43	188.00	7.90	0	1.00	1	1	1	1	1	1	1	0	0	0	0
1	1	38	1	174	75.7	8.00	25.1	121	70	222	56	166.00	5.30	0	1.00	0	0	1	0	0	1	1	0	1	0	1
1	1	51	2	159	51.9	-1.00	20.5	123	68	201	57	144.00	5.40	1	1.00	1	0	1	1	0	1	1	1	1	1	1
1	1	48	1	172	85.9	-2.90	29.1	142	86	280	44	236.00	5.70	1	1.00	1	1	1	0	1	1	1	0	0	0	1
1	1	70	1	153	57.3	-.40	24.4	149	82	220	93	127.00	5.30	0	1.00	0	1	1	0	0	1	0	1	0	0	1
1	1	64	2	159	49.3	-1.20	19.6	125	79	245	60	185.00	5.80	1	1.00	1	0	1	0	0	0	0	1	1	0	1
0	0	71	2	150	54.6	1.40	24.3	133	78	221	61	160.00	5.50	1	1.00	1	0	1	1	1	1	1	1	0	0	1
1	1	41	1	167	80.6	.30	28.8	128	89	206	54	152.00	5.30	0	1.00	0	0	1	0	1	1	0	0	0	1	1
0	1	51	2	152	49.5	-.40	21.3	109	72	225	109	116.00	5.90	1	1.00	1	0	1	0	0	0	0	1	1	0	1
1	1	70	1	167	61.4	-1.60	21.9	125	75	225	39	186.00	5.30	0	1.00	0	0	1	0	0	1	1	1	1	0	1

1	1	68	1	160	68.6	-.10	26.7	115	83	233	49	184.00	5.90	1	1.00	0	1	1	0	0	1	1	0	0	0	1
1	1	76	2	139	39.6	-.60	20.5	121	71	171	67	104.00	7.10	1	1.00	1	0	1	0	0	0	0	1	0	0	0
0	0	51	1	165	55.7	1.20	20.5	121	69	127	70	57.00	5.60	0	1.00	0	1	1	0	0	1	1	1	1	1	1
1	1	66	2	151	59.0	-5.20	25.8	138	75	220	65	155.00	5.70	1	1.00	1	0	1	0	0	1	0	0	1	0	1
1	1	46	1	164	69.1	2.80	25.5	102	63	205	31	174.00	5.50	0	1.00	1	0	1	0	1	0	0	0	1	0	1
1	1	54	2	156	49.1	-1.10	20.1	155	87	184	85	99.00	5.60	1	1.00	1	0	1	1	1	1	1	1	0	1	1
0	0	75	1	152	56.2	-.60	24.1	113	73	199	66	133.00	5.30	0	1.00	0	0	1	0	1	0	0	1	1	1	1
0	0	74	2	148	42.7	-1.10	19.3	118	66	174	64	110.00	5.40	1	1.00	1	0	1	0	1	1	0	1	0	1	1
1	1	70	1	164	63.4	.20	23.5	93	62	207	58	149.00	5.50	1	1.00	1	0	1	0	0	1	0	1	1	1	1
1	1	58	2	150	54.4	-.90	24.1	127	72	219	72	147.00	5.40	1	1.00	1	0	1	0	1	1	0	1	1	1	1
0	1	72	2	153	54.5	3.20	23.4	116	64	186	69	117.00	5.40	1	1.00	1	0	1	1	1	0	0	1	1	1	1
1	1	81	1	155	58.1	2.70	24.1	124	67	204	46	158.00	6.10	1	1.00	1	0	1	1	0	0	0	1	1	1	1
1	1	64	2	161	74.8	2.40	28.7	130	76	225	59	166.00	5.70	1	1.00	1	0	1	0	1	0	0	0	0	0	1
1	1	75	2	142	44.3	4.20	21.9	113	67	174	75	99.00	5.60	1	1.00	1	0	1	1	1	1	1	1	1	1	1
1	1	60	2	143	52.0	1.30	25.2	107	69	240	77	163.00	5.40	1	1.00	1	0	1	0	0	0	1	0	1	0	1
0	0	71	2	152	54.4	3.00	23.4	134	75	217	82	135.00	5.60	1	1.00	1	0	1	0	1	0	1	1	1	1	1
1	1	65	1	177	75.1	.30	23.8	134	83	159	47	112.00	5.30	1	1.00	0	0	1	0	1	1	1	1	1	1	1
1	1	62	2	151	47.5	1.00	20.8	126	77	234	50	184.00	5.40	1	1.00	1	0	1	0	1	0	0	1	0	0	1
0	0	22	1	164	66.2	3.90	24.6	103	55	190	58	132.00	6.90	1	1.00	0	0	0	0	0	1	1	1	1	1	0
1	1	71	2	149	52.2	.60	23.4	112	68	189	51	138.00	5.40	1	1.00	1	0	1	1	1	1	1	1	1	1	1
1	1	60	1	153	47.2	.00	20.2	100	68	186	50	136.00	5.60	0	1.00	0	1	1	0	1	1	1	1	1	1	1
1	1	44	2	158	55.1	-2.90	22.1	115	76	230	76	154.00	5.60	1	1.00	1	0	1	1	1	1	1	1	1	0	1
1	1	63	1	171	66.0	.80	22.6	132	89	207	50	157.00	5.80	1	1.00	0	1	1	1	1	1	1	1	1	1	1
1	1	50	2	156	48.2	1.00	19.7	102	67	251	91	160.00	5.60	1	1.00	1	0	1	1	1	1	1	1	1	0	1
1	1	51	2	154	54.6	-4.90	22.9	137	73	236	89	147.00	5.60	1	1.00	1	0	1	0	1	0	1	1	1	0	1
1	1	63	2	153	62.8	1.00	26.7	112	61	236	68	168.00	5.50	1	1.00	1	0	1	1	1	1	1	0	1	0	1
1	1	63	2	148	73.6	3.50	33.5	114	66	211	40	171.00	5.80	1	1.00	1	0	1	1	1	1	1	0	0	1	1
1	1	73	2	140	46.9	.40	23.8	93	52	144	53	91.00	5.30	1	1.00	1	0	1	1	1	1	0	1	1	1	1
1	1	62	2	152	57.8	-.20	24.8	112	70	200	74	126.00	5.30	1	1.00	1	0	1	0	1	0	1	1	1	1	1
1	1	54	2	154	53.4	1.70	22.6	88	58	218	77	141.00	6.00	1	1.00	1	0	1	0	1	0	0	1	1	1	1
1	1	50	2	162	60.7	-1.30	23.1	113	71	209	96	113.00	5.10	1	1.00	0	0	1	0	0	1	0	1	1	1	1
1	1	65	2	157	50.7	7.60	20.5	105	62	255	84	171.00	5.80	1	1.00	1	0	1	1	0	0	1	1	1	0	1
1	1	38	2	150	43.3	1.70	19.2	93	56	166	66	100.00	5.70	0	1.00	1	0	0	0	1	0	0	1	1	1	1
0	0	54	1	170	55.1	-.60	19.0	101	60	205	60	145.00	5.50	1	1.00	1	0	1	0	0	0	0	1	1	1	1
1	1	71	2	140	32.1	2.50	16.4	102	62	188	64	124.00	5.50	1	1.00	1	0	1	1	1	0	0	1	0	1	1
1	1	72	2	144	51.8	2.40	25.1	109	57	180	40	140.00	5.60	1	1.00	1	1	1	1	1	1	1	0	0	1	1
1	1	65	1	174	65.2	.80	21.5	143	85	150	50	100.00	5.60	0	2.00	0	0	1	1	1	1	1	1	0	1	1
1	1	30	2	163	69.1	-.50	26.0	130	71	198	80	118.00	5.50	1	1.00	0	0	1	0	1	1	1	0	1	1	1
1	1	60	1	167	73.1	1.90	26.0	118	70	178	43	135.00	5.30	1	1.00	0	0	1	0	0	0	0	0	1	1	1

1	1	63	2	151	50.0	.40	22.0	143	81	249	105	144.00	5.50	1	1.00	1	1	1	0	0	0	0	1	0	0	1
1	1	64	2	150	45.9	-1.40	20.4	125	66	177	70	107.00	5.50	1	1.00	1	1	1	0	1	0	0	1	0	1	1
1	1	61	1	170	67.0	-3.30	23.2	131	77	175	73	102.00	6.50	1	1.00	0	0	1	1	1	0	0	1	1	1	0
0	0	72	2	152	69.0	.50	30.0	130	77	164	46	118.00	6.10	1	1.00	1	0	1	1	1	0	1	0	1	0	1
0	0	47	2	159	87.0	-.50	34.5	135	89	232	58	174.00	5.40	1	1.00	1	0	1	0	0	0	0	0	0	0	1
1	1	77	2	145	39.9	-1.20	18.8	114	64	240	98	142.00	5.40	1	1.00	1	0	0	1	1	1	1	1	1	0	1
1	1	70	2	146	52.3	-.10	24.4	130	84	221	57	164.00	5.80	1	1.00	1	0	1	0	0	1	1	1	1	0	1
0	0	65	1	176	64.3	-.10	20.7	150	92	219	57	162.00	5.10	1	1.00	0	1	1	0	1	1	0	1	0	1	1
1	1	79	2	140	47.0	1.20	24.0	153	82	212	90	122.00	5.20	1	1.00	1	1	1	1	1	1	1	1	0	1	1
1	1	63	1	159	46.3	3.80	18.3	128	86	215	60	155.00	5.80	0	1.00	1	0	1	1	1	1	1	1	1	1	1
1	1	58	2	149	45.8	3.70	20.5	138	84	255	52	203.00	5.50	1	1.00	0	0	1	0	1	0	0	1	1	0	1
1	1	48	1	171	58.5	-1.60	20.0	127	80	159	80	79.00	4.90	1	1.00	0	0	1	1	1	1	1	1	1	1	1
1	1	78	1	156	60.4	.90	24.9	123	61	194	43	151.00	5.80	1	1.00	1	1	1	1	1	1	1	1	0	1	1
1	1	25	2	169	74.4	7.30	26.0	116	67	243	83	160.00	5.70	1	1.00	1	1	1	0	1	1	0	0	1	0	1
1	1	28	2	172	49.9	-3.10	16.9	101	54	182	77	105.00	5.10	0	1.00	0	1	1	1	1	0	1	1	1	1	1
1	1	56	2	163	50.3	1.70	19.0	126	68	214	73	141.00	5.40	1	1.00	1	0	1	0	0	0	0	1	1	1	1
0	0	74	1	160	52.3	2.70	20.4	131	69	131	44	87.00	6.40	0	1.00	0	1	1	0	1	1	1	1	1	1	0
0	0	71	1	170	62.8	.60	21.7	119	74	141	46	95.00	5.40	1	1.00	0	0	1	0	0	0	0	1	0	1	1
1	1	63	1	161	58.3	-.40	22.4	109	64	186	61	125.00	5.50	1	1.00	0	0	1	0	1	1	1	1	0	1	1
1	1	57	1	164	70.2	-3.80	26.0	124	75	203	57	146.00	7.20	1	1.00	0	0	1	1	1	0	1	0	0	0	0
1	1	67	2	151	56.0	1.60	24.6	129	79	258	63	195.00	5.40	1	1.00	1	0	1	0	1	1	1	1	1	0	1
1	1	28	2	167	75.1	2.00	27.0	108	63	150	54	96.00	5.40	1	1.00	1	1	1	1	1	1	1	0	1	1	1
1	1	67	2	148	51.7	-.50	23.7	114	65	173	47	126.00	5.80	1	1.00	1	1	1	0	0	0	0	1	0	1	1
1	1	66	1	162	67.2	-.20	25.4	126	89	241	64	177.00	5.30	1	1.00	1	0	1	0	1	0	1	0	1	0	1
0	0	60	2	152	50.5	-1.20	21.8	111	69	226	67	159.00	5.60	1	1.00	1	0	1	0	0	0	0	1	1	0	1
1	1	67	2	160	56.4	.30	21.9	125	78	176	52	124.00	5.40	1	1.00	1	0	1	1	1	0	1	1	0	0	1
1	1	69	2	149	59.9	-.20	27.1	153	85	216	61	155.00	6.10	1	1.00	1	1	1	1	1	0	0	0	0	1	1
0	0	58	2	154	57.9	5.80	24.4	107	65	164	83	81.00	5.40	1	1.00	1	0	1	0	1	0	0	1	0	1	1
1	1	69	2	155	47.3	3.20	19.6	123	71	203	99	104.00	5.30	1	1.00	1	0	1	1	1	1	1	1	1	0	1
1	1	63	2	156	62.9	-2.60	25.8	121	68	240	63	177.00	5.70	1	1.00	1	0	1	0	0	1	1	0	1	0	1
1	1	67	1	167	60.8	4.60	21.8	134	77	200	62	138.00	5.70	1	1.00	0	0	1	1	1	0	0	1	1	1	0
1	1	60	2	157	52.7	1.40	21.3	102	67	237	72	165.00	5.60	1	1.00	1	0	1	0	1	1	1	1	1	0	1
1	1	73	2	142	35.8	.30	17.6	156	81	205	66	139.00	5.90	1	1.00	1	0	1	1	1	0	0	1	0	1	1
0	0	76	1	173	65.3	-1.00	21.9	120	66	166	56	110.00	5.40	1	1.00	0	1	1	1	0	0	0	1	0	1	1
1	1	66	2	144	48.3	-2.00	23.2	105	73	203	71	132.00	5.60	1	1.00	1	0	1	0	0	0	1	1	1	1	1
0	0	63	2	153	57.7	4.40	24.6	92	55	217	57	160.00	5.70	1	1.00	1	1	1	1	0	1	1	1	1	1	1
1	1	59	1	161	65.9	2.90	25.3	142	94	159	61	98.00	5.20	1	1.00	0	0	1	0	1	1	0	0	0	1	1
1	1	46	2	148	50.3	6.00	22.9	116	71	238	102	136.00	5.30	0	1.00	0	1	0	0	1	1	1	1	1	0	1
0	0	60	2	153	52.9	2.60	22.7	103	60	255	95	160.00	5.40	1	1.00	1	0	1	1	1	1	1	1	1	0	1

0	0	34	2	148	37.7	1.90	17.2	106	75	164	91	73.00	5.60	1	1.00	0	1	1	1	1	0	1	1	1	1	1
1	1	65	1	174	63.5	2.60	20.9	135	77	131	53	78.00	5.30	0	1.00	0	0	1	1	0	1	1	1	1	1	0
1	1	40	2	146	65.8	-14.40	30.9	155	84	232	31	201.00	6.10	1	1.00	1	0	1	0	1	0	0	0	0	0	1
0	1	28	1	171	73.4	2.10	25.1	103	57	171	62	109.00	5.20	0	2.00	0	1	1	1	1	0	1	0	1	1	1
1	1	47	1	177	78.3	.60	25.1	125	78	222	48	174.00	5.00	0	1.00	0	0	0	0	1	1	1	0	1	0	1
1	1	62	2	153	43.4	-1.30	18.6	117	66	180	72	108.00	5.40	1	1.00	1	0	1	0	1	0	0	1	1	1	1
0	0	56	2	152	46.3	1.50	19.9	162	98	202	61	141.00	5.10	0	1.00	1	0	0	0	1	0	1	1	0	1	1
1	1	56	2	156	59.6	.20	24.4	132	86	215	55	160.00	5.40	0	1.00	0	1	1	1	1	1	0	1	0	1	1
1	1	77	2	143	47.3	-1.30	23.2	127	83	183	54	129.00	5.80	1	1.00	1	0	1	0	1	0	0	1	1	1	1
0	1	64	1	156	60.8	2.40	24.9	121	69	247	61	186.00	5.90	1	1.00	0	1	1	0	0	1	0	1	1	0	1
1	1	78	1	162	64.6	-3.90	24.4	137	78	197	57	140.00	5.70	1	1.00	0	1	1	1	1	1	1	1	0	1	1
1	1	38	2	166	54.5	1.40	19.6	89	56	174	76	98.00	5.50	0	1.00	0	0	1	0	1	1	1	1	1	1	1
1	1	62	2	154	66.0	-2.10	27.9	131	73	182	49	133.00	6.00	1	1.00	1	0	1	0	1	0	0	0	0	1	1
1	1	54	1	162	68.3	1.60	26.1	131	86	179	46	133.00	6.00	1	1.00	1	1	1	0	1	0	0	0	1	1	1
1	1	55	2	156	63.8	-.90	26.2	121	84	203	55	148.00	6.10	1	1.00	1	1	1	0	1	1	1	0	0	1	1
1	1	70	1	157	59.2	-1.50	24.0	159	94	174	53	121.00	5.70	1	1.00	0	1	1	0	1	1	0	1	0	1	1
1	1	47	2	154	47.3	-.70	19.8	100	65	204	83	121.00	5.30	0	1.00	1	1	1	1	1	0	1	1	1	1	1
1	1	67	2	151	72.5	-2.50	31.9	123	71	191	48	143.00	6.00	1	1.00	1	1	1	1	1	0	1	0	0	0	1
1	1	74	1	163	75.2	1.30	28.4	95	57	201	40	161.00	6.20	1	1.00	1	1	1	0	1	1	1	0	0	1	0
0	1	72	2	147	60.8	-.30	28.2	131	80	211	57	154.00	5.70	1	1.00	1	0	1	0	0	0	0	0	0	1	1
1	1	62	1	161	52.4	.10	20.2	103	67	188	95	93.00	5.50	1	1.00	1	0	1	1	1	1	1	1	1	1	1
1	1	55	2	162	67.2	.90	25.6	110	65	178	59	119.00	6.20	1	1.00	1	1	1	1	1	0	0	0	1	1	1
0	0	72	2	150	44.1	.00	19.7	195	105	192	72	120.00	5.20	1	1.00	1	1	1	0	1	0	0	1	0	1	1
1	1	68	1	159	66.7	.30	26.4	149	82	208	64	144.00	7.10	1	1.00	0	1	1	0	1	1	0	0	0	1	0
0	0	77	2	141	56.2	3.50	28.1	110	61	217	59	158.00	6.00	1	1.00	1	0	1	0	1	0	1	0	0	1	1
1	1	76	2	146	51.4	-.40	24.0	134	73	192	56	136.00	5.20	1	1.00	1	0	1	1	1	0	0	1	0	1	1
0	0	57	2	158	46.4	-.60	18.5	122	73	214	60	154.00	5.20	1	1.00	1	0	1	0	1	0	1	1	1	1	1
1	1	73	1	165	63.0	.20	23.1	96	61	203	36	167.00	7.40	1	1.00	0	0	1	1	1	0	0	1	0	0	0
1	1	83	1	162	75.1	2.70	28.5	122	64	169	46	123.00	6.90	1	1.00	1	0	1	1	1	1	1	0	0	0	0
1	1	61	1	169	63.4	1.40	22.2	126	82	261	105	156.00	5.20	1	1.00	0	1	1	0	1	0	0	1	0	0	1
1	1	50	2	147	45.1	-.10	20.9	175	109	187	76	111.00	5.30	1	1.00	0	0	0	0	1	0	0	1	0	1	1
0	0	68	2	146	47.8	-6.10	22.5	123	72	172	64	108.00	5.20	1	1.00	1	0	1	0	1	0	0	1	1	1	1
1	1	72	2	154	65.9	.30	27.9	152	88	233	61	172.00	5.90	1	1.00	1	0	1	1	1	1	1	0	0	0	1
1	1	55	2	151	45.7	-1.40	19.9	147	82	193	89	104.00	5.20	1	1.00	0	0	1	0	1	0	0	1	0	1	1
1	1	39	2	158	59.2	-3.00	23.5	129	71	217	78	139.00	5.50	1	1.00	1	1	1	0	1	1	1	1	1	1	1
0	0	78	2	141	50.3	.50	25.2	118	74	223	62	161.00	5.70	1	1.00	1	1	1	1	1	1	1	0	1	0	1
1	1	35	1	177	100.8	-6.60	32.1	132	78	251	39	212.00	5.00	1	1.00	1	0	1	0	1	0	1	0	1	0	1
1	1	69	1	161	68.2	-2.80	26.4	125	77	208	68	140.00	5.80	1	1.00	0	1	1	0	0	1	1	0	1	1	1
0	0	64	1	164	55.3	.80	20.4	136	77	173	56	117.00	5.50	0	1.00	1	1	1	0	0	0	1	1	1	1	1

1	1	49	1	164	63.6	1.30	23.7	120	81	187	57	130.00	5.20	0	2.00	0	0	1	0	1	0	1	1	1	1	1
1	1	72	2	149	61.8	1.30	27.8	150	84	161	52	109.00	5.30	1	1.00	1	0	1	1	0	0	1	0	0	1	1
1	1	32	2	157	53.1	.20	21.4	126	82	177	62	115.00	5.20	1	1.00	1	1	0	0	1	0	1	1	1	1	1
1	1	67	1	160	61.8	.70	24.2	122	69	221	81	140.00	6.00	1	1.00	0	0	1	1	0	1	1	1	1	0	1
1	1	55	2	152	58.8	.60	25.4	105	59	206	50	156.00	5.50	1	1.00	1	1	1	1	1	1	1	0	1	1	1
1	1	27	2	160	57.9	.00	22.7	110	65	235	51	184.00	5.40	1	1.00	1	0	1	0	1	0	1	1	1	0	1
1	1	73	1	159	60.4	-2.60	23.9	143	80	178	54	124.00	8.10	1	1.00	0	1	1	0	1	1	1	1	0	1	0
1	1	65	2	146	46.5	-1.30	21.8	126	64	207	73	134.00	5.90	1	1.00	1	1	1	1	1	1	1	1	0	1	0
0	0	81	1	154	53.7	-6.60	22.6	128	65	164	47	117.00	5.60	1	1.00	1	1	1	0	1	1	1	1	1	1	1
0	0	77	1	167	59.3	4.30	21.3	100	65	174	30	144.00	5.50	0	1.00	1	0	1	1	1	1	1	1	0	0	1
1	1	67	2	146	67.8	-3.10	31.8	128	66	218	65	153.00	6.30	1	1.00	1	1	1	1	1	1	1	0	1	1	1
1	1	56	1	162	70.3	4.70	26.8	119	67	194	82	112.00	5.40	1	1.00	0	0	0	0	1	0	1	0	1	1	1
0	0	50	2	149	59.5	3.30	26.8	101	62	241	70	171.00	5.70	1	1.00	1	1	1	1	1	0	1	0	1	0	1
1	1	62	1	172	65.7	3.70	22.2	123	73	223	60	163.00	5.40	1	1.00	1	0	1	0	0	0	0	1	1	0	1
0	0	78	2	144	35.9	-.10	17.3	129	75	208	56	152.00	5.20	1	1.00	1	0	1	1	1	0	1	1	1	1	1
1	1	72	1	162	69.8	.40	26.6	149	72	208	61	147.00	6.00	1	1.00	0	1	1	1	1	0	1	0	0	1	1
0	0	58	2	155	61.9	.80	25.6	134	80	232	68	164.00	5.60	1	1.00	1	0	1	0	0	0	0	0	0	0	1
1	1	42	2	158	61.2	.30	24.6	112	62	156	62	94.00	5.00	1	1.00	1	1	1	1	1	1	1	1	1	1	1
0	0	60	1	161	59.0	.30	22.7	130	80	224	72	152.00	5.30	1	1.00	0	1	1	0	1	1	0	1	1	0	1
0	0	61	1	169	67.2	.90	23.6	115	74	170	54	116.00	5.60	1	1.00	1	0	1	0	1	0	0	1	1	1	1
0	0	62	2	148	67.3	6.20	30.7	127	74	204	62	142.00	5.50	1	1.00	0	1	1	0	0	0	1	0	0	1	1
1	1	64	2	151	48.9	.50	21.3	115	63	162	60	102.00	5.20	1	1.00	0	1	1	1	1	0	0	1	0	1	1
0	0	42	1	186	75.6	.10	21.8	110	72	172	57	115.00	5.70	1	1.00	1	0	1	0	1	0	1	1	1	1	1
1	1	34	2	149	54.0	3.60	24.2	109	72	243	62	181.00	5.40	0	1.00	1	0	1	0	0	1	0	1	1	0	1
1	1	68	2	147	49.9	.80	22.9	110	68	209	43	166.00	6.60	1	1.00	1	1	1	1	1	1	1	1	0	1	0
1	1	29	2	150	47.6	-2.50	21.2	98	57	172	79	93.00	4.70	0	1.00	0	0	1	1	1	1	1	1	1	1	1
0	0	46	2	156	63.7	-5.20	26.0	135	77	203	65	138.00	5.60	0	1.00	1	0	1	0	1	0	0	0	1	1	1
0	0	60	1	165	58.4	-.50	21.5	140	79	254	50	204.00	5.80	0	1.00	0	1	1	0	1	1	1	1	0	0	1
0	0	54	1	156	62.0	.10	25.5	168	110	269	142	127.00	5.30	0	1.00	0	1	1	1	1	1	1	0	0	0	1
1	1	68	1	159	65.3	-1.30	25.8	169	104	229	48	181.00	6.30	1	1.00	0	1	1	0	1	1	1	0	0	0	1
0	0	73	1	161	54.6	1.40	21.0	101	62	122	45	77.00	5.40	1	1.00	0	1	1	1	1	1	1	1	1	1	1
1	1	87	1	148	46.4	2.30	21.3	122	67	211	61	150.00	5.10	1	1.00	1	0	1	1	1	1	1	1	1	1	1
0	0	73	1	168	68.3	1.80	24.1	136	89	159	33	126.00	6.10	1	1.00	1	0	1	0	0	1	1	1	1	0	0
1	1	70	2	146	49.3	-2.00	23.0	140	74	262	67	195.00	5.60	1	1.00	1	0	1	0	1	0	1	1	0	0	1
1	1	55	2	153	52.2	-1.00	22.1	123	73	259	73	186.00	5.20	1	1.00	1	0	1	0	1	0	0	1	1	0	1
1	1	50	2	160	57.0	.30	22.1	111	72	205	97	108.00	5.10	1	1.00	0	0	1	0	0	0	0	1	1	1	1
1	1	67	2	149	56.5	-.70	25.3	95	54	229	57	172.00	5.30	1	1.00	1	0	1	0	1	0	0	0	1	0	1
1	1	62	2	150	41.9	1.20	18.6	86	55	212	75	137.00	5.30	1	1.00	1	1	1	1	1	1	1	1	1	1	1
0	0	58	2	161	65.6	.20	25.3	105	63	165	71	94.00	6.30	1	1.00	1	0	1	0	1	0	0	0	0	1	1

1	1	52	2	157	53.4	-1.60	21.6	114	80	253	64	189.00	5.90	0	1.00	1	1	1	1	1	1	1	1	1	0	1
1	1	55	1	164	79.3	-.10	29.4	137	90	278	52	226.00	6.50	1	1.00	1	0	1	0	1	1	1	0	0	0	0
1	1	57	1	177	90.6	-13.80	29.0	138	86	217	56	161.00	7.40	1	1.00	1	1	1	0	1	1	0	0	1	1	0
0	0	74	2	149	51.3	-1.00	22.9	102	56	175	65	110.00	6.00	0	1.00	0	0	1	0	0	0	0	1	0	1	1
1	1	71	2	148	51.8	.10	23.7	120	69	250	68	182.00	5.80	1	1.00	1	1	1	0	1	0	0	1	0	0	1
1	1	42	1	172	88.3	1.10	29.7	129	83	231	64	167.00	5.50	0	1.00	0	0	1	0	1	0	1	0	1	0	1
1	1	63	2	153	56.3	.00	24.1	129	77	236	59	177.00	5.60	1	1.00	1	1	1	0	0	1	1	1	1	0	1
1	1	28	2	162	43.1	8.90	16.4	88	54	148	67	81.00	5.20	1	1.00	1	1	1	0	1	0	0	1	1	1	1
1	1	69	2	146	70.4	.40	32.9	150	91	256	56	200.00	6.20	1	1.00	1	0	1	0	0	0	1	0	0	0	0
1	1	67	2	150	45.7	-.80	20.3	97	56	180	61	119.00	5.20	1	1.00	1	0	1	0	1	1	1	1	1	1	1
0	0	67	2	151	68.9	3.10	30.0	166	91	204	53	151.00	5.60	1	1.00	1	0	1	0	1	1	0	0	0	1	1
0	0	75	2	146	40.7	-2.90	19.0	108	51	189	59	130.00	5.70	1	1.00	1	0	1	0	0	0	0	1	0	1	1
1	1	59	1	167	64.1	-2.30	23.0	110	73	218	53	165.00	5.40	0	1.00	1	1	1	1	1	0	1	1	1	1	1
1	1	74	1	155	60.8	-3.70	25.4	142	80	202	66	136.00	5.00	1	1.00	0	0	1	1	0	1	1	0	0	1	1
0	0	76	2	144	47.9	-1.80	23.1	131	77	215	74	141.00	5.60	1	1.00	0	0	1	1	1	1	0	1	1	1	1
1	1	57	2	150	64.2	-.10	28.4	114	73	194	58	136.00	6.10	1	1.00	1	1	1	0	1	1	1	0	0	1	1
0	1	51	2	153	55.7	-.30	23.8	103	63	262	102	160.00	5.40	1	1.00	1	1	1	0	0	1	1	1	1	0	1
1	1	56	2	155	70.9	-2.10	29.3	132	85	198	70	128.00	5.80	1	1.00	1	1	1	1	1	1	1	0	1	0	1
0	0	62	2	154	74.0	-.60	31.1	146	76	254	50	204.00	7.60	1	1.00	1	1	1	0	0	0	0	0	0	0	0
0	0	64	2	146	48.3	.90	22.7	124	65	183	68	115.00	6.10	1	1.00	1	1	1	0	1	1	1	1	0	1	0
1	1	73	2	147	59.5	-1.30	27.5	120	61	205	69	136.00	5.20	1	1.00	1	0	1	1	1	0	1	0	0	1	1
0	0	42	1	171	67.6	-.10	23.1	113	73	202	61	141.00	5.30	0	1.00	0	0	1	0	1	0	1	1	1	1	1
1	1	52	1	168	54.8	-2.70	19.3	125	85	213	75	138.00	5.10	0	1.00	0	0	1	0	1	0	1	1	1	1	1
1	1	64	1	163	59.8	.70	22.6	161	92	199	59	140.00	5.30	1	1.00	1	1	1	1	1	0	1	1	0	1	1
0	0	24	1	173	67.4	.80	22.5	112	61	188	64	124.00	5.60	0	1.00	1	1	1	0	1	0	1	1	1	1	1
0	0	70	1	155	66.6	.30	27.7	131	70	223	48	175.00	5.20	1	1.00	0	0	1	1	1	1	1	0	1	0	1
0	0	29	2	159	64.3	3.40	25.4	113	63	186	79	107.00	5.50	1	1.00	0	1	1	1	1	1	1	0	1	1	1
1	1	53	2	162	57.5	-1.00	22.0	114	72	197	64	133.00	5.50	1	1.00	1	1	1	1	0	0	0	1	1	1	1
1	1	71	1	160	55.1	1.60	21.4	112	57	182	55	127.00	5.20	1	1.00	1	0	1	1	1	1	1	1	1	0	1
0	0	64	1	171	74.4	-3.80	25.5	134	80	185	95	90.00	6.10	1	1.00	0	0	1	0	1	1	1	0	1	1	0
0	0	27	2	153	53.8	.80	22.9	107	61	177	65	112.00	5.30	1	1.00	1	0	1	0	1	1	1	1	1	1	1
1	1	50	1	175	77.8	3.20	25.5	123	83	226	50	176.00	5.10	0	1.00	0	1	0	0	0	0	0	0	1	0	1
1	1	60	2	148	70.1	-3.10	32.1	123	81	270	47	223.00	5.10	1	1.00	1	0	1	0	0	1	1	0	1	0	1
0	0	74	1	166	85.3	8.40	30.8	124	62	196	54	142.00	7.70	1	1.00	0	0	1	1	1	0	1	0	1	1	0
0	0	68	1	157	55.8	-.70	22.5	128	68	169	89	80.00	5.20	1	1.00	0	0	1	1	1	1	1	1	0	1	1
0	1	58	1	166	68.8	-.40	24.9	124	72	182	59	123.00	5.50	1	1.00	0	0	1	0	1	1	1	1	0	1	1
1	1	60	2	152	68.7	-1.60	29.9	142	87	187	68	119.00	5.40	1	1.00	1	0	1	0	1	0	1	0	0	0	1
1	1	72	2	148	56.8	1.60	25.7	157	87	225	81	144.00	5.50	1	1.00	1	1	1	1	0	1	1	0	0	0	1
0	0	60	2	150	43.0	1.50	19.1	140	90	193	41	152.00	5.50	1	1.00	1	0	1	1	1	1	1	1	0	0	1

1	1	66	2	149	47.0	-.70	21.2	155	89	223	69	154.00	5.50	1	1.00	1	1	1	0	1	0	0	1	0	0	1
1	1	73	1	159	66.5	.60	26.4	134	75	209	45	164.00	5.70	0	1.00	0	1	1	0	1	1	1	0	1	1	1
0	0	72	2	155	58.5	-.80	24.2	143	83	196	53	143.00	5.70	1	1.00	1	0	1	0	1	0	1	1	0	1	1
1	1	69	2	152	57.9	1.00	25.0	133	83	231	39	192.00	5.40	1	1.00	1	1	1	1	1	0	0	0	1	0	1
1	1	77	2	151	48.1	5.00	21.2	127	64	245	39	206.00	5.80	1	1.00	1	0	1	1	0	0	0	1	0	0	1
1	1	56	2	156	56.1	2.60	23.2	165	90	192	64	128.00	5.30	1	1.00	0	0	1	1	1	1	1	1	0	1	1
0	0	58	1	162	70.0	2.40	26.5	196	124	181	59	122.00	5.90	0	1.00	0	1	1	0	1	1	1	0	0	1	1
1	1	56	1	160	72.6	-3.60	28.5	129	78	137	50	87.00	5.80	1	1.00	1	0	1	1	1	0	1	0	0	1	1
1	1	69	1	163	70.8	-1.20	26.5	162	95	247	49	198.00	5.90	1	1.00	0	0	1	1	1	1	1	0	0	0	1
1	1	66	1	163	47.8	-1.30	18.0	121	70	144	69	75.00	5.20	0	1.00	0	1	1	0	1	1	1	1	1	1	0
0	0	57	2	148	54.9	.30	25.1	143	93	239	36	203.00	5.80	1	1.00	1	0	1	0	0	0	0	0	0	0	1
1	1	43	1	173	69.3	1.10	23.1	99	54	247	66	181.00	5.50	1	1.00	0	1	1	1	0	1	1	1	1	0	1
1	1	62	1	166	56.8	11.00	20.5	126	79	186	61	125.00	5.30	0	1.00	1	1	1	0	1	1	1	1	1	1	1
0	0	78	2	142	54.7	.50	27.0	118	64	231	60	171.00	5.80	1	1.00	1	0	1	0	1	1	1	0	0	0	1
1	1	75	2	156	48.8	2.50	20.0	138	73	210	88	122.00	5.60	1	1.00	0	0	1	0	1	0	0	1	1	0	1
0	0	73	2	140	43.6	-1.00	22.1	119	70	212	61	151.00	5.90	1	1.00	1	0	1	0	1	0	0	1	1	1	1
1	1	51	1	171	55.2	3.90	18.7	116	71	198	65	133.00	5.30	1	1.00	0	1	1	1	1	1	1	1	1	1	1
1	0	66	2	159	47.2	-1.20	18.6	96	55	214	61	153.00	5.60	1	1.00	1	0	1	0	0	0	0	1	1	1	1
1	1	70	2	142	46.0	1.00	22.8	129	74	314	93	221.00	5.50	1	1.00	1	0	0	0	1	0	0	1	1	0	1
1	1	69	2	143	62.9	-1.80	30.6	141	83	198	74	124.00	6.10	1	1.00	1	0	1	1	1	1	1	0	0	1	1
1	1	61	1	166	62.3	2.10	22.7	158	85	202	71	131.00	5.90	1	1.00	0	0	1	1	0	1	0	1	0	1	1
1	1	44	2	151	46.8	.90	20.4	90	54	159	65	94.00	5.10	1	1.00	0	1	1	1	1	0	0	1	1	1	1
1	1	66	2	157	47.1	1.20	19.0	112	73	291	61	230.00	5.30	1	1.00	1	1	1	0	1	0	0	1	1	0	1
1	1	71	2	146	38.7	1.00	18.2	136	81	229	49	180.00	5.70	1	1.00	1	0	1	1	1	1	1	1	1	0	1
1	1	57	2	146	53.9	2.70	25.3	105	66	183	50	133.00	5.60	1	1.00	1	0	1	0	0	0	0	0	1	1	1
1	1	41	2	158	56.1	-4.60	22.5	116	81	177	70	107.00	5.50	1	1.00	1	0	1	1	1	1	1	1	1	1	1
1	1	56	2	156	50.2	-.20	20.5	103	59	172	73	99.00	6.50	1	1.00	1	1	1	1	1	0	0	1	1	1	0
1	1	53	2	149	47.4	-.70	21.4	109	59	165	73	92.00	4.20	1	1.00	1	0	1	1	1	1	1	1	1	1	1
1	1	71	2	147	44.8	-.10	20.6	165	82	202	77	125.00	5.50	1	1.00	1	0	1	1	1	0	1	1	0	0	1
1	1	70	2	147	57.6	-.60	26.6	101	65	244	73	171.00	5.70	1	1.00	1	0	1	0	1	1	1	0	1	0	1
1	1	56	1	167	69.4	.30	24.9	112	68	194	48	146.00	5.60	1	1.00	1	1	1	0	1	0	1	1	0	1	1
1	1	57	2	148	54.4	-2.20	24.7	112	71	277	103	174.00	5.90	1	1.00	0	0	1	0	1	1	1	1	1	0	1
1	1	39	2	156	41.1	1.50	16.8	98	59	172	93	79.00	5.60	1	1.00	1	1	1	1	1	1	0	1	1	1	1
1	1	38	2	156	46.5	4.00	19.0	123	66	223	87	136.00	5.60	0	1.00	1	0	1	0	1	0	0	1	1	0	1
1	1	45	2	151	40.3	2.60	17.6	104	66	162	34	128.00	5.80	1	1.00	1	0	1	0	1	0	1	1	1	0	1
0	0	43	2	163	72.4	7.30	27.1	102	69	262	53	209.00	5.50	1	1.00	1	0	1	1	1	1	1	0	1	0	1
1	1	75	1	166	57.3	1.00	20.9	105	56	175	58	117.00	6.50	1	1.00	0	0	1	1	1	1	0	1	1	1	0
0	0	74	2	153	58.1	-.90	24.7	124	69	275	65	210.00	6.30	1	1.00	1	0	1	1	0	0	0	1	1	0	0
1	1	39	2	156	49.8	-1.20	20.5	93	56	211	76	135.00	5.10	1	1.00	1	0	0	1	1	0	0	1	1	1	1

1	1	60	2	150	48.3	1.90	21.6	121	78	222	110	112.00	5.80	1	1.00	1	0	1	1	1	1	1	1	1	0	1
1	1	66	2	144	42.6	-2.80	20.5	123	74	219	73	146.00	6.40	1	1.00	1	0	1	1	1	0	1	1	1	1	1
1	1	63	2	150	48.3	-.30	21.3	133	92	174	60	114.00	5.70	1	1.00	1	0	1	0	1	1	1	1	0	0	1
1	1	61	2	151	53.3	.80	23.4	120	79	183	62	121.00	7.90	1	1.00	1	0	1	1	1	1	1	1	0	1	0
1	1	81	2	153	54.3	-.40	23.2	104	58	194	53	141.00	5.70	1	1.00	1	0	1	1	1	1	1	1	0	1	1
1	1	60	2	151	48.2	-1.00	21.1	118	72	213	62	151.00	5.30	1	1.00	1	0	1	1	1	0	0	1	1	1	1
0	0	69	2	149	42.1	2.80	19.0	99	60	220	97	123.00	5.40	1	1.00	1	0	1	1	1	0	1	1	1	0	1
1	1	56	1	172	79.2	4.40	26.9	120	74	194	46	148.00	6.00	1	1.00	1	1	1	1	1	1	1	0	1	1	0
1	1	71	1	168	74.3	3.60	26.4	118	64	167	41	126.00	6.20	1	1.00	1	0	1	0	1	1	1	0	0	1	0
1	1	71	2	157	56.8	1.00	22.9	110	60	208	72	136.00	6.10	1	1.00	1	0	1	1	1	0	0	1	1	1	1
1	1	64	1	170	60.6	5.80	21.0	102	62	222	67	155.00	6.00	0	1.00	0	1	1	1	1	1	1	1	1	0	1
1	1	79	1	148	54.5	-2.50	24.9	100	42	196	42	154.00	5.20	1	1.00	0	0	1	1	1	1	0	1	0	1	1
0	0	41	1	171	54.4	.20	18.5	102	68	252	89	163.00	5.20	1	1.00	0	0	1	0	1	1	1	1	1	0	1
1	1	62	2	153	68.8	-1.70	29.4	105	78	244	73	171.00	5.60	1	1.00	0	1	1	1	1	1	0	0	1	0	1
0	0	45	2	152	56.1	-5.10	24.4	93	59	261	83	178.00	5.40	1	1.00	1	1	1	0	0	1	1	1	1	0	1
1	1	67	1	155	47.1	.90	19.5	100	60	201	71	130.00	5.90	1	1.00	0	0	1	1	0	1	1	1	1	1	1
1	1	43	2	152	42.8	3.00	18.4	112	65	220	66	154.00	5.20	1	1.00	1	0	1	0	1	0	0	1	1	0	1
1	1	21	1	177	61.7	1.90	19.8	107	60	149	56	93.00	5.00	1	1.00	0	1	1	1	1	0	1	1	1	1	1
1	1	49	2	150	44.8	-1.50	19.8	151	83	218	107	111.00	5.40	1	1.00	1	1	1	1	1	1	1	1	0	1	1
1	1	63	1	167	50.3	7.20	18.0	106	61	110	30	80.00	5.00	0	1.00	0	0	1	0	0	1	1	1	1	0	1
1	1	50	2	155	49.7	4.30	20.7	125	73	232	85	147.00	5.50	1	1.00	0	0	1	1	0	1	0	1	1	0	1
1	1	56	2	157	57.3	-3.10	23.3	117	72	163	72	91.00	5.20	1	1.00	1	0	1	0	0	1	1	1	1	1	1
0	0	64	2	147	47.0	2.00	21.8	126	75	228	69	159.00	6.00	1	1.00	0	1	1	0	0	0	0	1	0	0	1
1	1	62	2	149	60.7	2.50	27.3	121	76	274	43	231.00	6.40	1	1.00	1	1	1	0	0	0	0	0	1	0	1
1	1	71	1	146	48.3	.80	22.7	129	70	144	69	75.00	5.30	1	1.00	1	0	1	0	1	0	1	1	0	1	1
1	1	53	2	156	56.2	2.50	23.1	114	62	245	53	192.00	6.00	1	1.00	1	1	1	0	0	0	0	1	1	0	1
1	1	62	2	149	65.0	-.40	29.1	127	77	189	48	141.00	5.40	1	1.00	0	0	1	1	0	1	1	0	1	1	1
1	1	68	2	145	54.6	-1.40	25.9	120	66	169	69	100.00	8.30	1	1.00	1	0	1	0	1	0	0	0	1	1	0
1	1	84	1	157	58.6	.70	23.8	132	71	155	43	112.00	5.70	1	1.00	1	0	1	1	1	1	1	1	1	0	1
1	1	75	2	142	51.7	1.30	25.8	129	68	227	55	172.00	6.70	1	1.00	1	0	1	0	1	1	1	0	1	0	0
1	1	50	2	143	46.0	2.90	22.5	128	79	181	66	115.00	5.20	1	1.00	1	0	1	1	1	1	1	1	1	1	1
0	1	62	2	145	41.8	.70	19.9	107	61	261	65	196.00	5.60	1	1.00	1	0	1	0	1	0	0	1	1	0	1
1	1	66	2	158	54.3	2.40	21.7	133	81	185	67	118.00	5.20	1	1.00	1	0	1	1	1	1	1	1	1	1	1
1	1	67	2	149	63.5	3.10	28.7	122	77	200	43	157.00	5.70	1	1.00	1	1	1	1	1	0	0	0	1	1	1
1	1	63	1	169	78.6	-2.60	27.6	152	82	192	40	152.00	5.70	1	1.00	0	1	1	0	0	1	1	0	0	1	1
1	1	56	2	159	51.4	1.20	20.4	94	54	220	74	146.00	5.40	1	1.00	1	0	1	1	1	1	1	1	1	0	1
1	1	46	2	160	55.1	1.00	21.5	106	75	220	54	166.00	5.70	0	2.00	1	0	0	0	0	0	0	1	1	0	1
0	0	69	2	145	53.2	.70	25.1	124	65	229	49	180.00	5.30	1	1.00	1	0	1	1	0	0	1	0	0	0	1
1	1	76	2	144	69.1	1.50	33.2	150	83	211	73	138.00	6.10	1	1.00	1	0	0	0	1	0	1	0	0	1	1

1	1	41	1	164	47.5	.50	17.5	113	76	150	47	103.00	5.40	1	1.00	1	1	1	0	1	1	1	1	1	1	1
1	1	62	2	158	42.0	1.00	16.8	111	66	168	51	117.00	5.60	1	1.00	1	0	1	1	1	1	1	1	1	1	1
1	1	45	2	153	58.1	-3.00	24.8	113	63	165	57	108.00	5.80	1	1.00	1	0	1	0	0	0	1	1	1	1	1
1	1	64	1	156	52.2	-1.70	21.3	134	85	247	110	137.00	5.70	1	1.00	0	1	1	1	1	1	0	1	1	0	1
1	1	52	2	147	50.4	5.40	23.2	141	92	151	55	96.00	5.30	1	1.00	1	1	1	1	1	1	0	1	0	1	1
1	1	60	2	149	62.6	-.20	28.1	126	73	187	43	144.00	5.50	1	1.00	1	0	1	0	1	0	0	0	1	0	1
0	0	50	1	166	69.2	-2.20	25.1	144	83	177	55	122.00	5.60	0	1.00	0	0	0	1	0	0	0	0	0	1	1
1	1	67	2	147	57.0	.80	26.2	131	76	170	61	109.00	6.10	1	1.00	1	0	1	0	1	1	1	0	0	1	1
1	1	68	2	155	63.6	-1.00	26.5	102	72	194	58	136.00	5.70	1	1.00	0	1	1	1	1	1	1	0	0	1	1
1	1	37	1	168	80.9	.90	28.6	130	79	266	60	206.00	5.40	1	1.00	0	1	1	0	0	0	1	0	1	0	1
1	1	70	1	158	67.3	.10	26.8	132	72	214	85	129.00	6.70	1	1.00	0	1	1	1	1	1	1	0	0	1	0
0	0	41	2	164	60.6	1.20	22.5	118	82	184	50	134.00	5.50	1	1.00	1	0	1	0	0	0	0	1	1	1	1
1	1	56	2	155	46.0	-1.20	19.0	111	70	149	60	89.00	5.60	1	1.00	1	1	1	0	1	1	1	1	1	1	1
1	1	68	1	173	70.9	3.10	23.6	120	67	181	66	115.00	5.20	0	1.00	1	1	1	0	1	1	1	1	1	1	1
1	1	66	1	164	74.5	.60	27.7	112	76	212	56	156.00	5.80	1	1.00	0	1	1	1	1	1	1	0	0	1	1
1	1	29	2	139	43.3	1.40	22.5	105	65	268	50	218.00	5.40	1	1.00	1	0	0	1	1	0	0	1	1	0	1
1	1	80	2	146	49.4	-2.80	23.1	149	72	231	75	156.00	7.20	1	1.00	1	0	1	1	1	1	1	1	0	0	0
1	1	61	2	150	51.5	1.10	22.7	134	70	174	59	115.00	5.50	1	1.00	1	1	0	0	1	0	1	1	1	1	1
1	1	64	2	158	57.6	-1.60	23.2	110	70	183	64	119.00	5.50	1	1.00	1	0	1	1	1	1	1	1	0	1	1
1	1	57	2	154	60.6	1.90	25.6	122	74	247	56	191.00	5.80	0	2.00	0	0	1	1	1	1	0	0	1	0	1
1	1	75	1	153	53.9	-.50	22.9	139	78	169	83	86.00	7.00	1	1.00	0	1	1	1	1	1	1	1	0	1	0
1	1	64	2	146	50.4	-.60	23.6	135	80	278	47	231.00	5.70	1	1.00	1	0	1	1	1	1	1	1	1	0	1
1	1	61	1	158	77.7	-5.00	31.2	158	80	234	43	191.00	5.40	1	1.00	0	0	1	1	1	1	1	0	0	0	1
0	0	72	2	148	48.0	.00	21.8	118	64	157	59	98.00	6.40	1	1.00	1	1	1	1	1	1	0	1	0	1	1
1	1	48	1	161	64.4	.20	24.8	100	53	196	56	140.00	5.30	1	1.00	1	1	1	0	1	0	1	1	1	1	1
1	1	67	2	152	59.0	2.00	25.6	136	81	225	52	173.00	5.70	1	1.00	0	1	1	1	1	1	1	0	1	0	1
1	1	73	2	152	58.9	1.50	25.4	122	73	214	65	149.00	5.60	1	1.00	1	1	1	0	1	1	0	0	1	0	1
1	1	72	2	157	57.3	-.20	23.1	110	70	165	37	128.00	5.70	1	1.00	1	1	1	0	1	1	0	1	0	0	1
1	1	26	2	163	53.6	-.70	20.1	102	64	181	69	112.00	5.70	1	1.00	1	0	1	1	1	1	1	1	1	1	1
1	1	88	1	163	58.7	.10	22.0	114	58	192	76	116.00	6.10	1	1.00	0	0	1	0	0	0	1	1	0	1	1
1	1	66	2	151	58.2	1.50	25.4	126	70	197	61	136.00	6.40	1	1.00	1	1	1	0	0	0	0	0	0	0	1
1	1	77	1	158	45.7	-1.00	18.3	114	73	193	37	156.00	5.90	0	1.00	0	0	1	1	1	1	1	1	0	0	1
0	0	75	1	163	54.9	2.30	20.6	118	73	198	68	130.00	7.00	1	1.00	0	1	1	0	0	0	0	1	0	1	0
0	0	67	1	163	72.6	-2.80	27.4	115	73	185	47	138.00	5.50	1	1.00	0	0	1	0	1	1	0	0	0	1	1
0	1	74	2	152	48.8	2.40	21.0	113	67	169	60	109.00	6.90	1	1.00	1	0	1	0	1	0	1	1	0	0	0
0	0	79	1	160	58.0	-.10	22.7	111	68	179	77	102.00	10.60	1	1.00	0	0	1	0	0	0	1	1	1	1	0
1	1	60	2	161	51.0	.50	19.7	119	77	217	81	136.00	6.10	0	1.00	0	0	1	0	0	0	0	1	1	1	1
1	1	56	2	159	57.3	3.90	22.7	139	87	211	72	139.00	5.20	1	1.00	1	0	0	0	0	0	0	1	1	1	1
0	0	61	2	156	52.1	3.10	21.4	112	69	198	65	133.00	6.00	1	1.00	0	1	1	0	0	0	0	1	1	1	1

0	0	51	1	164	72.0	-.50	26.8	117	76	230	51	179.00	5.30	0	2.00	0	0	1	0	1	0	0	0	1	0	1
1	1	38	2	161	69.1	.30	26.5	119	69	221	82	139.00	5.00	1	1.00	0	1	1	1	1	1	1	0	1	0	1
0	0	69	1	162	48.2	1.90	18.3	140	78	199	91	108.00	5.90	1	1.00	0	0	1	0	1	1	1	1	0	1	1
1	1	62	2	149	49.9	-1.30	22.4	109	68	194	59	135.00	5.70	1	1.00	0	1	1	1	1	0	1	1	1	1	1
1	1	39	1	167	75.5	1.40	27.0	122	76	219	76	143.00	5.20	1	1.00	0	1	0	0	1	1	1	0	1	1	1
1	1	67	2	149	51.4	.60	23.0	126	75	201	74	127.00	6.20	1	1.00	1	1	1	1	1	1	1	1	1	0	1
1	1	61	2	152	42.2	.80	18.3	92	45	293	68	225.00	5.60	1	1.00	1	1	1	0	1	0	0	1	1	0	1
1	1	51	2	154	58.4	9.20	24.7	120	71	278	55	223.00	6.10	1	1.00	0	0	1	0	1	0	0	1	1	0	1
1	1	60	1	173	64.6	.10	21.6	123	81	173	65	108.00	5.70	1	1.00	0	1	1	1	1	1	1	1	1	1	1
1	1	73	2	144	51.1	-1.20	24.6	143	75	214	82	132.00	5.40	1	1.00	1	1	1	0	1	0	0	1	0	1	1
0	0	41	2	146	50.7	-.10	23.7	118	80	219	57	162.00	5.00	1	1.00	1	1	1	1	1	0	0	1	1	1	1
0	0	51	1	168	60.7	.90	21.4	119	73	173	33	140.00	5.30	0	1.00	0	0	1	0	1	1	1	1	1	0	1
0	0	69	2	147	43.0	1.30	19.8	86	56	240	78	162.00	5.60	1	1.00	1	0	1	0	0	0	1	1	1	0	1
0	0	41	1	167	71.6	1.10	25.7	126	76	197	59	138.00	5.20	1	1.00	0	1	0	0	1	1	1	0	1	1	1
1	1	61	2	147	38.7	-.70	17.8	102	56	193	86	107.00	5.70	1	1.00	1	0	1	0	0	0	0	1	1	1	1
0	0	63	1	168	69.1	.80	24.5	97	64	243	49	194.00	5.70	1	1.00	0	0	1	0	1	0	0	1	1	0	1
0	0	73	1	172	63.9	1.80	21.5	105	68	157	76	81.00	5.50	1	1.00	0	0	1	0	0	0	0	1	1	1	1
0	0	65	1	168	82.2	-5.90	29.2	111	71	263	41	222.00	8.10	1	1.00	1	0	1	0	0	0	0	0	0	0	0
0	0	61	2	151	55.1	3.70	24.1	96	65	239	73	166.00	5.90	1	1.00	1	1	1	0	1	0	0	1	0	0	1
1	1	69	1	158	60.5	1.00	24.2	160	73	212	42	170.00	5.30	1	1.00	0	0	1	0	0	1	1	1	0	0	1
0	0	49	1	168	65.0	2.10	22.9	122	80	194	73	121.00	5.10	0	1.00	0	1	0	0	1	1	1	1	1	1	1
1	1	71	2	150	46.6	2.80	20.7	126	71	198	80	118.00	6.10	1	1.00	1	0	1	0	1	1	1	1	0	1	0
0	1	56	1	161	64.0	-1.30	24.7	143	82	208	74	134.00	5.50	1	1.00	0	0	1	0	0	0	1	1	0	1	1
1	1	64	1	162	68.2	2.10	26.1	164	83	213	40	173.00	5.80	1	1.00	1	1	1	1	1	1	1	0	0	1	1
0	0	68	2	142	42.7	-.50	21.0	117	75	193	68	125.00	5.80	1	1.00	1	0	1	1	1	1	1	1	0	1	1
0	0	78	2	139	39.1	.20	20.2	103	57	185	78	107.00	5.70	1	1.00	1	0	1	0	1	0	0	1	1	0	1
0	0	83	1	160	58.7	1.10	22.9	112	51	181	60	121.00	6.10	1	1.00	1	0	1	0	1	1	0	1	0	1	1
0	0	67	2	130	37.9	1.60	22.4	142	86	211	72	139.00	5.90	1	1.00	1	0	1	0	0	1	1	1	0	1	1
0	0	53	2	158	54.5	2.40	21.8	100	63	189	66	123.00	5.60	1	1.00	1	0	1	0	0	0	0	1	1	1	1
0	0	84	2	156	49.8	1.60	20.4	107	63	243	72	171.00	5.80	1	1.00	1	0	1	1	1	0	1	1	0	0	1
0	0	66	1	164	78.2	1.30	29.0	120	73	194	44	150.00	5.40	1	1.00	1	1	1	1	1	0	0	0	1	1	1
1	1	60	1	161	56.0	.00	21.6	103	71	208	57	151.00	5.50	0	1.00	0	1	1	0	1	1	1	1	0	1	1
0	1	80	1	156	61.4	3.90	25.3	117	63	206	49	157.00	5.70	1	1.00	1	0	1	1	1	0	1	0	0	1	1
1	1	76	1	167	66.4	.30	23.8	118	66	157	65	92.00	5.20	1	1.00	0	1	1	1	1	1	1	1	1	1	1
0	0	44	1	176	66.0	-1.10	21.3	143	82	239	69	170.00	5.60	1	1.00	1	1	1	0	1	1	0	1	0	0	1
0	0	70	2	159	51.9	-1.40	20.4	108	60	203	78	125.00	5.80	1	1.00	1	1	1	0	1	0	1	1	1	1	1
1	1	47	2	154	61.7	.80	26.1	132	81	191	52	139.00	5.10	1	1.00	1	0	1	1	1	0	0	0	1	1	1
0	0	55	2	159	55.1	2.20	21.7	137	84	220	69	151.00	5.70	1	1.00	0	0	1	0	1	0	0	1	0	0	1
1	1	62	2	142	48.1	-2.80	23.8	121	64	274	72	202.00	5.50	1	1.00	1	1	1	0	1	0	0	1	1	0	1

0	0	61	1	168	63.3	-2.10	22.3	119	80	182	42	140.00	5.10	1	1.00	0	0	1	1	1	1	1	1	1	1	1
1	1	70	2	153	64.1	.50	27.2	138	78	195	57	138.00	5.50	1	1.00	1	1	1	1	1	1	1	0	0	1	1
1	1	66	2	145	51.5	-.90	24.4	110	78	209	62	147.00	5.70	1	1.00	1	0	1	1	1	0	1	1	1	1	1
0	0	65	2	154	48.9	2.80	20.7	121	71	209	80	129.00	6.10	1	1.00	1	1	1	1	0	1	1	1	0	1	1
0	0	24	1	178	82.8	12.20	26.1	138	75	205	79	126.00	5.50	1	1.00	0	1	1	1	1	0	1	0	1	1	1
1	1	47	2	157	45.0	1.20	18.2	104	62	148	66	82.00	5.20	1	1.00	1	0	1	1	1	1	1	1	1	1	1
1	1	46	2	149	46.5	2.70	20.8	108	61	210	89	121.00	4.70	1	1.00	1	1	1	1	1	1	1	1	1	1	1
0	0	44	2	149	53.0	2.30	23.8	107	77	197	81	116.00	5.50	0	1.00	0	1	1	0	1	0	0	1	1	1	1
1	1	40	2	165	56.5	3.00	20.8	108	67	161	49	112.00	5.50	0	1.00	0	1	0	0	1	0	1	1	1	1	1
1	1	77	2	134	34.4	-.90	19.0	165	81	267	62	205.00	5.70	1	1.00	1	0	1	1	1	0	1	1	0	0	1
1	1	54	2	155	58.4	2.50	24.2	130	69	284	83	201.00	5.60	1	1.00	1	1	1	0	0	1	1	1	1	0	1
0	0	73	1	164	74.2	2.40	27.4	157	84	178	57	121.00	5.90	1	1.00	0	0	1	1	0	1	1	0	0	1	1
1	1	59	1	167	71.4	1.50	25.6	129	80	266	44	222.00	5.70	1	1.00	0	0	1	1	1	0	1	0	1	0	1
1	1	72	2	140	64.7	1.00	33.1	159	81	250	80	170.00	5.70	1	1.00	1	1	1	0	1	1	1	0	0	0	1
1	1	72	2	142	51.7	.60	25.7	148	81	165	42	123.00	6.40	1	1.00	1	1	1	1	1	0	1	0	0	1	1
0	0	53	2	153	62.9	3.80	26.8	100	70	195	43	152.00	5.90	1	1.00	1	1	1	0	1	0	1	0	1	1	1
1	1	38	1	177	88.8	-.40	28.2	137	86	217	44	173.00	5.50	1	1.00	0	0	1	0	1	0	0	0	1	1	1
1	1	40	2	159	62.5	7.50	24.6	104	66	210	75	135.00	5.20	1	1.00	1	1	1	0	1	1	1	1	1	1	1
1	1	69	1	160	57.9	1.70	22.7	108	71	195	46	149.00	7.40	1	1.00	1	1	1	0	1	1	0	1	1	1	0
0	0	58	1	166	67.2	3.00	24.3	116	77	229	50	179.00	6.00	1	1.00	1	0	1	0	1	0	1	1	1	0	1
0	0	64	2	147	48.1	2.50	22.1	112	71	207	50	157.00	5.20	1	1.00	1	1	1	1	1	0	1	1	1	1	1
1	1	64	2	153	52.3	-.90	22.4	100	55	251	42	209.00	5.70	1	1.00	1	1	1	1	1	0	0	1	1	0	1
1	1	63	1	156	89.8	-.50	37.0	121	74	158	55	103.00	6.10	1	1.00	1	1	1	0	1	1	1	0	1	1	0
1	1	45	2	151	56.6	-.10	24.9	105	58	202	76	126.00	5.40	1	1.00	1	0	1	1	1	1	1	1	1	1	1
1	1	73	2	149	48.4	-2.30	21.9	116	74	168	57	111.00	5.90	1	1.00	1	0	1	1	1	1	1	1	0	0	1
1	1	57	1	171	76.5	-3.20	26.3	115	75	202	37	165.00	5.50	1	1.00	0	1	1	0	1	1	1	0	1	0	1
0	0	33	1	165	68.4	-1.20	25.2	111	63	201	30	171.00	5.70	0	1.00	1	0	1	1	1	1	1	0	1	0	1
0	0	44	1	172	76.4	-2.40	25.9	126	82	205	59	146.00	5.10	0	1.00	0	1	1	0	1	0	0	0	1	1	1
0	0	58	2	153	45.8	1.00	19.5	106	63	230	62	168.00	5.70	0	1.00	1	1	1	0	1	0	0	1	1	0	1
1	1	56	2	161	63.6	1.20	24.5	132	92	283	84	199.00	5.30	1	1.00	0	0	1	1	0	1	1	1	0	0	1
0	0	61	2	142	72.1	.00	35.6	125	62	199	68	131.00	5.60	1	1.00	1	0	1	0	1	0	1	0	0	1	1
1	1	70	2	143	38.4	-.80	18.8	133	74	219	59	160.00	5.00	1	1.00	0	0	1	0	1	1	1	1	0	1	1
1	1	52	2	153	55.1	2.50	23.5	110	62	241	77	164.00	5.40	1	1.00	1	0	1	0	1	0	0	1	1	0	1
1	1	64	2	148	57.9	3.20	26.3	125	72	233	71	162.00	6.10	1	1.00	1	1	1	1	1	1	1	0	1	0	1
1	1	51	1	169	65.4	1.40	22.7	128	82	230	51	179.00	5.70	1	1.00	0	0	1	0	1	0	0	1	0	0	1
0	0	56	1	168	85.9	-2.40	30.5	136	83	200	49	151.00	7.70	1	1.00	1	0	1	0	1	1	1	0	0	1	0
1	1	63	2	147	48.3	.00	22.3	115	72	192	74	118.00	5.50	1	1.00	1	0	1	0	1	0	0	1	1	0	1
0	0	67	2	147	44.6	-.10	20.7	112	69	216	93	123.00	5.10	0	1.00	0	0	1	0	1	1	1	1	0	1	1
1	1	38	1	170	57.3	1.90	19.8	111	56	193	55	138.00	5.40	0	1.00	0	1	0	1	1	1	1	1	1	1	1

0	0	70	2	144	45.2	1.40	21.6	118	69	184	65	119.00	6.70	1	1.00	1	0	1	1	1	1	1	1	0	1	0
1	1	56	2	156	61.5	2.50	25.1	115	67	207	77	130.00	5.70	1	1.00	1	0	1	0	0	0	0	0	0	1	1
1	1	67	1	153	57.5	-1.30	24.6	107	67	124	50	74.00	5.60	1	1.00	0	0	1	0	1	1	1	1	0	1	1
1	1	69	2	153	54.0	.00	23.0	123	70	247	70	177.00	5.50	1	1.00	1	0	0	0	0	0	0	1	1	0	1
1	1	72	2	148	62.7	1.30	28.6	135	75	175	58	117.00	6.70	1	1.00	1	0	1	0	0	0	0	0	0	1	0
1	1	64	2	155	39.7	-.50	16.5	102	65	204	66	138.00	5.70	1	1.00	1	0	1	1	1	0	0	1	1	1	1
0	0	53	2	157	79.7	2.00	32.4	122	72	249	50	199.00	5.40	1	1.00	1	0	1	1	0	1	1	0	1	0	1
1	1	39	2	160	85.2	.40	33.4	158	85	174	61	113.00	6.80	0	1.00	0	0	1	0	1	0	1	0	0	1	0
1	1	46	2	158	59.4	1.30	23.7	151	81	195	64	131.00	5.20	1	1.00	1	0	1	0	1	1	0	1	0	1	1
1	1	74	2	155	52.1	-.60	21.7	119	76	154	52	102.00	6.40	1	1.00	1	0	1	0	0	1	1	1	0	1	1
1	1	60	2	150	49.3	-1.60	22.0	131	77	204	60	144.00	5.70	1	1.00	1	0	1	0	0	0	1	1	1	1	1
1	1	62	2	144	42.0	-1.70	20.3	97	58	284	58	226.00	5.30	1	1.00	1	0	1	0	0	0	0	1	1	0	1
1	1	71	1	171	69.0	-5.70	23.5	132	79	171	34	137.00	5.60	0	1.00	0	1	1	1	1	1	1	1	1	0	1
0	0	71	2	151	40.9	1.90	17.9	127	67	229	51	178.00	5.70	1	1.00	1	0	1	0	1	1	0	1	1	0	1
1	1	69	2	149	38.4	4.30	17.2	113	71	95	79	16.00	5.20	1	1.00	0	0	1	1	1	0	0	1	0	1	1
1	1	67	1	162	68.4	-1.30	26.0	119	72	159	29	130.00	5.60	1	1.00	0	1	1	1	1	1	1	0	1	0	1
1	1	61	2	150	38.4	5.00	17.1	124	70	182	65	117.00	5.50	1	1.00	1	0	1	1	1	0	1	1	0	0	1
1	1	70	1	166	61.0	1.00	22.2	130	94	150	66	84.00	5.90	1	1.00	0	0	1	0	1	0	1	1	0	1	1
1	1	52	1	165	77.9	-1.60	28.7	126	79	184	47	137.00	5.90	1	1.00	0	0	1	1	1	1	1	0	0	1	1
1	1	60	1	167	66.0	2.80	23.7	131	77	211	60	151.00	5.10	1	1.00	0	0	1	1	1	1	1	1	1	1	1
1	1	61	1	161	69.2	4.40	26.7	164	108	189	83	106.00	5.20	1	1.00	0	0	1	1	1	1	1	0	0	1	1
0	0	51	2	163	54.1	-.50	20.3	113	63	220	73	147.00	6.20	1	1.00	1	0	1	0	0	0	1	1	1	0	1
1	1	64	2	155	54.8	2.20	22.6	126	78	213	72	141.00	5.60	1	1.00	0	0	1	1	1	0	0	1	1	1	1
1	1	58	1	176	63.2	-1.80	20.3	128	81	205	58	147.00	5.70	0	1.00	1	0	1	1	1	1	0	1	1	1	1
1	1	43	2	154	41.3	3.00	17.3	110	66	209	92	117.00	5.50	1	1.00	1	0	1	0	0	0	1	1	1	1	1
0	0	50	2	142	44.9	-.30	22.1	113	62	231	58	173.00	5.80	1	1.00	1	0	1	0	0	0	0	1	1	0	1
1	1	72	2	148	70.7	-.80	32.1	107	59	144	43	101.00	6.70	1	1.00	1	0	1	1	1	1	1	0	0	1	0
1	1	93	1	157	54.7	-1.40	22.2	120	59	180	62	118.00	5.70	1	1.00	1	1	1	1	1	1	1	1	1	1	1
1	1	79	2	142	52.8	-1.00	26.1	131	86	177	48	129.00	5.50	1	1.00	1	1	1	1	1	1	1	0	1	1	1
1	1	73	1	162	72.3	-2.80	27.5	118	68	208	53	155.00	5.70	1	1.00	1	0	1	1	1	1	1	0	1	1	1
1	1	66	2	149	46.7	.80	21.1	128	70	205	64	141.00	5.60	1	1.00	1	1	1	1	1	1	1	1	0	1	1
1	1	68	2	154	45.5	-.30	19.2	117	72	227	91	136.00	5.60	1	1.00	0	1	1	0	1	1	1	1	1	0	1
1	1	66	1	168	67.5	1.60	24.0	164	90	194	40	154.00	6.80	1	1.00	1	0	1	0	0	0	0	1	0	1	0
1	1	63	2	146	60.9	-.10	28.4	157	81	162	61	101.00	6.10	1	1.00	1	0	1	1	1	1	1	0	0	1	0
1	1	81	2	138	39.5	.40	20.7	138	81	235	85	150.00	5.50	1	1.00	1	0	1	1	1	1	1	1	0	0	1
1	1	77	2	144	53.9	3.60	25.9	129	85	253	57	196.00	5.40	1	1.00	1	1	1	1	0	0	1	0	1	0	1
1	1	78	2	154	49.3	-1.70	20.6	109	63	200	68	132.00	7.00	1	1.00	0	0	1	1	1	0	0	1	0	1	0
1	1	75	2	155	58.8	1.60	24.5	117	63	249	50	199.00	6.20	1	1.00	0	0	1	1	1	0	0	1	1	0	1
1	1	74	2	157	65.5	.60	26.7	114	68	183	76	107.00	5.40	1	1.00	1	0	1	1	1	0	1	0	1	0	1

1	1	63	2	146	48.3	1.00	22.6	125	70	197	62	135.00	7.40	1	1.00	1	0	1	1	1	0	1	1	1	0	0
1	1	62	2	150	41.4	.50	18.4	137	85	282	55	227.00	5.90	1	1.00	1	1	1	1	0	1	1	1	1	0	1
1	1	69	2	148	47.1	.80	21.5	127	69	189	62	127.00	5.70	1	1.00	1	0	1	1	1	0	1	1	0	1	1
1	1	69	2	146	42.9	1.80	20.1	128	64	223	100	123.00	5.90	1	1.00	1	1	1	1	1	1	1	1	1	0	1
1	1	68	2	144	46.0	2.60	22.1	104	68	238	48	190.00	5.60	1	1.00	1	0	1	0	1	0	1	1	0	0	1
1	1	67	1	164	69.2	-1.20	25.8	144	91	194	73	121.00	5.90	1	1.00	1	0	1	1	1	1	1	0	0	1	1
1	1	67	1	164	69.1	.70	25.7	134	88	145	50	95.00	6.00	1	1.00	0	1	1	1	1	1	1	0	0	1	0
1	1	51	1	161	53.4	.00	20.6	110	78	210	76	134.00	5.70	1	1.00	0	1	1	0	1	0	1	1	1	1	1
1	1	65	2	150	48.6	-.60	21.7	119	62	235	88	147.00	6.00	1	1.00	1	0	1	1	1	1	1	1	1	0	1
0	0	67	1	160	82.1	6.10	32.2	136	83	296	60	236.00	6.20	1	1.00	0	0	1	0	1	0	1	0	0	0	1
1	1	77	1	158	53.9	.40	21.5	133	70	196	40	156.00	6.20	1	1.00	1	0	1	0	1	1	1	1	0	0	0
1	1	80	2	142	58.4	1.00	29.0	147	76	184	53	131.00	5.70	1	1.00	1	1	1	1	1	1	1	0	0	1	1
1	1	69	2	146	57.2	1.60	26.9	125	72	232	54	178.00	6.70	1	1.00	1	1	1	1	1	1	1	0	0	0	0
1	1	44	2	159	46.8	-.60	18.5	106	66	117	37	80.00	5.70	1	1.00	1	0	1	0	1	0	0	1	1	0	1
1	1	49	2	161	63.3	-.20	24.3	125	60	204	47	157.00	5.90	1	1.00	1	0	1	0	1	0	1	1	1	1	1
0	0	48	1	179	127.7	7.80	39.7	120	74	183	37	146.00	6.20	0	1.00	1	1	1	1	1	1	1	0	0	0	0
1	1	63	2	156	53.2	2.90	21.9	133	75	236	52	184.00	11.40	1	1.00	1	0	0	0	0	0	0	1	0	0	0
1	1	73	1	156	47.4	3.60	19.3	140	75	191	93	98.00	5.10	1	1.00	0	1	1	1	1	1	1	1	0	1	1
1	1	41	1	162	67.1	.30	25.7	128	92	264	59	205.00	5.20	1	1.00	1	1	1	0	1	1	1	0	0	0	1
1	1	68	1	166	48.7	-1.40	17.7	94	55	131	53	78.00	5.60	1	1.00	1	0	1	0	1	0	0	1	1	1	1
1	1	62	2	159	43.2	.50	17.0	143	81	217	69	148.00	5.60	1	1.00	1	1	1	1	1	1	1	1	0	1	1
1	1	68	2	159	53.7	2.70	21.2	107	56	190	72	118.00	6.10	1	1.00	1	0	1	1	1	1	1	1	1	0	1
0	0	80	1	150	57.4	-1.30	25.6	146	91	266	56	210.00	6.10	1	1.00	1	1	1	1	1	1	1	0	0	0	1
1	1	59	1	168	71.7	-2.90	25.3	136	82	246	57	189.00	5.60	1	1.00	0	0	1	0	0	0	0	0	1	0	1
1	1	65	1	165	73.3	2.50	27.0	166	86	164	29	135.00	5.50	0	2.00	1	0	1	1	0	1	0	0	0	0	1
1	1	57	2	154	47.1	-.70	19.8	101	55	193	53	140.00	5.40	1	1.00	1	0	1	0	0	0	0	1	1	1	1
1	1	49	1	165	69.6	.40	25.5	118	80	198	43	155.00	5.20	0	1.00	0	0	0	0	1	1	1	0	0	1	1
1	1	30	1	169	56.0	.10	19.7	119	75	140	64	76.00	5.40	1	1.00	1	0	1	0	1	0	0	1	1	1	1
0	0	66	2	150	46.2	2.90	20.6	100	67	227	62	165.00	5.40	1	1.00	1	0	1	1	1	1	1	1	0	0	1
1	1	82	1	153	48.4	-5.90	20.6	149	85	142	62	80.00	5.30	1	1.00	0	1	1	1	1	1	1	1	0	1	1
1	1	53	2	155	57.2	1.60	23.9	103	66	214	56	158.00	6.00	1	1.00	1	0	1	0	1	1	1	1	1	1	1
1	1	58	2	156	59.8	-2.10	24.4	117	76	230	54	176.00	5.40	1	1.00	1	0	1	0	0	0	1	1	1	0	1
1	1	54	2	151	63.4	-4.40	27.9	138	84	218	50	168.00	6.30	1	1.00	1	1	1	0	1	0	0	0	0	1	1
1	1	55	2	145	41.5	-1.30	19.6	112	62	172	58	114.00	5.60	1	1.00	1	0	1	0	1	0	0	1	1	1	1
1	1	44	2	165	49.2	-1.80	18.0	105	68	150	102	48.00	4.80	0	1.00	0	0	0	0	0	1	1	1	1	1	1
1	1	73	2	142	47.7	4.50	23.7	93	59	194	78	116.00	5.90	1	1.00	1	0	1	0	1	0	0	1	1	1	1
1	1	62	1	168	73.1	-1.70	25.9	118	74	251	33	218.00	5.00	1	1.00	1	1	1	1	1	1	1	0	1	0	1
1	1	63	2	157	69.2	.80	28.1	153	88	245	60	185.00	6.40	1	1.00	0	0	1	0	1	1	0	0	0	0	1
1	1	74	1	165	58.3	.30	21.3	130	63	135	76	59.00	5.10	0	1.00	0	1	1	1	1	0	1	1	0	1	1

0	0	73	1	155	53.5	-1.00	22.4	123	71	193	65	128.00	5.30	1	1.00	0	1	1	0	1	1	1	1	1	1	1
1	1	71	2	138	45.3	-.70	23.8	113	63	201	90	111.00	6.50	1	1.00	1	0	1	1	1	1	1	1	0	1	0
1	1	73	1	163	57.9	2.00	21.8	136	76	176	71	105.00	6.10	1	1.00	0	1	1	1	1	1	1	1	0	1	1
1	1	72	1	164	54.8	.60	20.3	153	84	170	62	108.00	5.10	0	1.00	0	1	1	0	1	0	0	1	0	1	1
1	1	45	2	152	42.1	-.60	18.2	104	61	190	85	105.00	5.80	1	1.00	1	0	1	1	1	0	1	1	1	1	1
1	1	64	2	146	52.3	3.20	24.5	126	83	197	58	139.00	6.10	1	1.00	1	0	1	1	1	1	1	1	1	1	1
0	0	50	2	150	58.5	3.50	25.8	131	82	213	76	137.00	5.30	1	1.00	1	0	1	0	1	0	0	0	1	1	1
1	1	68	1	156	52.6	.00	21.5	125	66	217	53	164.00	6.00	1	1.00	1	1	1	0	1	0	1	1	1	1	1
1	1	65	1	156	54.9	2.00	22.5	104	63	214	70	144.00	5.50	1	1.00	1	1	1	0	1	1	1	1	0	1	1
1	1	68	2	147	65.5	.50	30.3	123	68	242	55	187.00	5.60	1	1.00	1	0	1	1	0	0	1	0	1	0	1
1	1	66	1	163	64.2	2.70	24.0	135	86	211	68	143.00	5.80	1	1.00	0	0	1	0	1	1	0	1	1	1	1
1	1	47	2	151	55.2	.50	24.2	118	76	184	33	151.00	5.60	1	1.00	1	0	1	0	1	1	1	1	1	0	1
1	1	43	1	167	86.0	-5.70	30.6	123	78	237	41	196.00	5.00	1	1.00	1	1	1	0	0	1	0	0	1	0	1
1	1	64	2	151	45.5	-4.00	20.0	137	74	222	60	162.00	5.80	1	1.00	1	0	1	1	1	1	0	1	1	0	1
1	1	20	2	150	59.6	2.90	26.5	112	60	191	52	139.00	5.20	1	1.00	1	0	1	0	1	1	1	0	1	1	1
1	1	62	2	141	50.2	.10	25.3	114	62	191	51	140.00	5.50	1	1.00	0	0	0	0	0	0	0	0	1	1	1
1	1	65	1	165	72.3	-1.30	26.6	131	82	166	39	127.00	6.20	1	1.00	0	1	1	1	0	1	1	0	0	0	1
1	1	64	1	174	90.7	-4.20	29.8	158	91	205	41	164.00	6.00	1	1.00	0	0	0	0	0	0	0	0	0	1	1
0	0	45	2	161	60.4	4.70	23.4	94	50	188	51	137.00	5.30	1	1.00	1	1	1	0	1	0	1	1	1	1	1
1	1	50	2	157	45.9	-.30	18.6	98	63	252	89	163.00	5.60	1	1.00	1	1	1	1	1	1	1	1	1	0	1
1	1	78	1	153	50.0	-2.00	21.2	119	73	222	69	153.00	5.50	1	1.00	0	0	1	1	1	1	1	1	1	0	1
1	1	76	1	150	53.3	-2.50	23.5	128	73	173	26	147.00	6.10	1	1.00	1	1	1	1	1	1	1	1	1	0	0
1	1	67	2	148	49.1	-.60	22.5	140	83	305	78	227.00	5.70	1	1.00	1	0	1	1	1	1	1	1	0	0	1
0	0	73	2	140	50.1	1.60	25.4	132	73	205	100	105.00	5.90	1	1.00	1	1	1	0	1	0	0	0	0	1	1
1	1	67	1	166	55.2	-.70	20.1	106	61	164	89	75.00	5.80	1	1.00	0	1	1	1	1	1	1	1	1	1	1
1	1	43	1	174	63.5	1.80	20.8	111	64	215	74	141.00	5.70	1	1.00	1	1	1	1	1	1	1	1	1	1	1
1	1	70	1	152	56.1	-1.50	24.4	122	71	207	52	155.00	5.70	1	1.00	1	0	1	1	1	0	1	1	0	1	1
1	1	70	1	162	62.2	1.20	23.7	202	96	240	91	149.00	5.70	1	1.00	0	1	1	1	1	1	0	1	0	0	1
1	1	55	2	148	43.8	-4.60	20.0	134	84	182	56	126.00	5.40	1	1.00	1	0	1	1	1	1	1	1	0	1	1
1	1	59	2	165	53.1	-.20	19.5	98	63	202	73	129.00	5.40	1	1.00	1	1	1	1	1	1	1	1	1	1	1
1	1	67	2	142	57.6	-3.20	28.4	127	72	225	65	160.00	5.60	1	1.00	1	0	1	1	1	1	1	0	0	0	1
1	1	51	1	166	58.3	-1.40	21.1	113	72	162	73	89.00	5.40	0	1.00	0	1	1	0	1	0	0	1	1	1	1
1	1	78	1	164	51.2	1.30	19.1	120	70	197	89	108.00	5.40	1	1.00	1	1	1	1	1	0	1	1	0	1	1
1	1	71	1	160	74.3	-.60	29.0	147	84	146	51	95.00	6.50	1	1.00	1	1	1	1	1	0	1	0	0	1	0
1	1	72	2	137	43.2	-1.40	22.8	128	83	157	58	99.00	5.60	1	1.00	1	0	1	0	1	0	0	1	0	0	1
1	1	61	2	162	47.8	2.10	18.1	139	85	197	58	139.00	5.40	1	1.00	0	0	1	1	1	0	1	1	1	1	1
1	1	60	2	152	58.8	3.00	25.5	138	76	254	73	181.00	5.80	1	1.00	1	0	0	0	1	0	0	0	1	0	1
1	1	68	2	157	59.9	-1.60	24.4	122	60	289	43	246.00	6.80	1	1.00	1	1	1	1	1	1	1	1	1	0	0
1	1	44	2	155	47.1	-.80	19.6	102	70	210	75	135.00	5.30	1	1.00	1	0	1	1	1	0	0	1	1	1	1

0	0	59	1	167	65.1	2.60	23.3	121	89	174	81	93.00	5.10	1	1.00	0	0	1	1	1	0	1	1	0	1	1
0	0	64	1	158	64.2	.80	25.6	149	79	174	72	102.00	5.80	1	1.00	0	1	1	0	0	1	1	0	0	0	1
1	1	45	2	156	55.8	-5.90	22.8	97	55	261	74	187.00	5.60	1	1.00	1	0	0	0	1	0	0	1	1	0	1
0	0	34	1	172	63.8	-.30	21.4	93	54	170	41	129.00	5.70	0	1.00	0	0	1	0	1	0	0	1	1	1	1
0	0	50	2	151	51.6	3.00	22.5	112	69	180	74	106.00	5.50	1	1.00	1	0	1	1	1	0	0	1	1	1	1
1	1	59	2	145	47.5	2.00	22.5	99	56	219	64	155.00	5.60	1	1.00	1	0	1	1	1	0	0	1	1	1	1
1	1	52	2	153	47.6	1.30	20.4	156	84	266	73	193.00	5.80	1	1.00	1	0	1	1	1	1	1	1	0	0	1
1	1	44	2	153	58.7	2.00	25.2	112	77	172	47	125.00	5.90	0	1.00	1	1	1	1	1	0	0	0	1	1	1
0	0	90	1	159	60.2	2.20	23.8	99	52	175	42	133.00	5.60	1	1.00	1	0	1	1	1	1	0	1	0	1	1
1	1	70	2	148	53.0	.40	24.1	129	75	197	80	117.00	5.50	1	1.00	1	1	1	0	1	1	0	1	1	1	1
0	0	64	2	150	41.5	-.20	18.3	153	83	198	56	142.00	6.40	1	1.00	1	0	1	0	1	0	0	1	0	0	0
0	0	66	2	149	55.3	9.20	25.0	148	69	209	62	147.00	5.40	1	1.00	1	0	1	0	1	1	1	0	0	1	1
0	0	76	2	142	49.3	-.10	24.5	106	59	179	69	110.00	5.50	1	1.00	1	0	1	0	0	0	1	1	1	1	1
1	1	62	1	161	64.3	-1.20	24.7	112	71	178	62	116.00	6.10	1	1.00	0	1	1	1	1	1	1	1	0	1	0
1	1	81	1	153	62.9	-4.70	26.9	137	79	170	34	136.00	5.60	1	1.00	1	1	1	1	1	1	1	0	0	0	1
0	0	60	2	150	42.0	1.90	18.5	97	62	244	80	164.00	5.50	1	1.00	0	1	0	0	1	0	1	1	1	0	1
0	0	44	2	160	53.9	6.90	21.1	109	72	183	81	102.00	5.00	1	1.00	0	0	1	0	0	0	1	1	1	1	1
1	1	60	1	165	79.3	-2.70	29.2	130	79	171	42	129.00	5.20	1	1.00	1	0	1	1	0	1	1	0	1	1	1
0	0	48	1	162	56.7	.40	21.6	109	62	192	47	145.00	5.80	1	1.00	1	1	1	1	1	1	0	1	1	1	1
1	1	41	1	161	56.5	-1.40	21.8	116	73	199	76	123.00	5.40	0	1.00	0	1	0	1	1	1	1	1	1	1	1
1	1	74	2	146	58.2	-1.20	27.4	115	70	193	64	129.00	6.20	1	1.00	1	0	1	0	1	1	1	0	1	1	1
1	1	57	2	152	50.4	1.00	21.8	107	75	195	64	131.00	5.90	1	1.00	1	0	1	1	1	1	1	1	1	0	1
0	0	64	2	152	57.0	-1.60	24.6	117	80	223	50	173.00	6.30	1	1.00	1	0	1	0	0	0	1	1	1	0	1
0	0	44	1	168	89.1	.00	31.4	143	93	204	46	158.00	6.20	1	1.00	0	0	1	1	1	1	1	0	0	1	1
0	1	69	1	167	71.5	-1.60	25.5	116	75	181	52	129.00	6.40	1	1.00	1	0	1	0	1	0	0	0	1	1	0
0	0	69	2	154	50.4	1.60	21.3	116	70	275	51	224.00	5.70	1	1.00	1	0	1	0	1	0	0	1	0	0	1
1	1	50	1	164	56.0	1.10	20.8	109	66	183	67	116.00	5.20	0	1.00	0	0	1	1	0	0	1	1	1	1	1
0	0	39	1	175	62.4	-2.00	20.4	141	89	232	76	156.00	5.50	1	1.00	0	0	1	1	1	1	1	1	0	0	1
0	0	57	2	151	70.1	-5.40	30.8	115	75	178	80	98.00	5.60	1	1.00	1	0	1	0	1	1	1	0	1	1	1
0	0	71	2	153	73.3	6.80	31.4	132	74	196	65	131.00	5.70	1	1.00	1	0	1	0	0	0	0	0	1	1	1
0	0	39	1	168	99.5	.90	35.1	140	82	218	36	182.00	5.40	1	1.00	1	1	1	1	1	1	1	0	0	0	1
1	1	23	1	165	84.9	-5.10	31.1	130	74	196	64	132.00	5.70	1	1.00	1	0	1	1	1	1	1	0	1	1	1
1	1	48	2	158	53.0	-1.40	21.3	142	83	211	88	123.00	5.70	1	1.00	0	0	1	1	1	1	0	1	0	1	1
1	1	60	2	150	49.6	-2.80	22.1	132	81	197	58	139.00	5.60	1	1.00	1	1	1	0	1	0	1	1	1	0	1
0	0	58	2	156	38.9	-.10	16.0	127	71	266	95	171.00	5.10	1	1.00	0	1	0	1	1	1	1	1	1	0	1
0	0	67	1	159	74.5	-1.70	29.6	142	76	208	44	164.00	6.80	1	1.00	1	1	1	0	1	0	1	0	0	1	0
1	1	83	2	142	56.7	2.50	28.0	126	72	206	45	161.00	5.50	1	1.00	1	0	1	1	1	1	1	0	1	1	1
1	1	60	1	165	64.0	-2.80	23.3	103	58	139	46	93.00	5.00	1	1.00	0	1	1	0	1	0	0	1	0	1	1
0	0	38	2	159	54.2	.70	21.4	141	89	155	57	98.00	5.60	1	1.00	1	0	0	0	0	0	0	1	0	1	1

0	0	52	2	147	47.3	-4.50	21.8	109	65	236	91	145.00	5.30	1	1.00	1	1	1	0	0	1	1	1	1	0	1
1	1	70	1	169	64.8	.00	22.7	116	67	221	67	154.00	5.70	1	1.00	0	1	1	1	1	0	0	1	1	0	1
0	0	28	1	167	78.2	6.00	28.1	127	70	196	54	142.00	5.30	0	1.00	1	0	1	0	1	1	1	0	1	1	1
0	0	62	1	160	69.0	-1.60	26.9	118	62	235	46	189.00	5.70	0	1.00	1	1	1	1	1	0	0	0	1	0	1
0	0	61	2	155	62.5	-3.10	26.1	157	89	238	60	178.00	5.90	1	1.00	1	0	1	0	1	0	0	0	0	0	1
1	1	72	2	147	63.5	1.20	29.5	121	74	182	57	125.00	6.00	1	1.00	1	0	1	1	1	1	1	0	1	1	1
1	1	59	2	151	39.3	1.50	17.1	99	52	169	74	95.00	4.90	0	1.00	1	1	1	1	1	0	1	1	1	1	1
0	0	20	2	148	44.2	-.40	20.1	99	56	160	73	87.00	5.10	1	1.00	1	0	1	0	0	0	1	1	1	1	1
0	0	63	2	152	47.2	2.40	20.3	128	84	197	68	129.00	5.60	1	1.00	1	0	1	1	1	1	1	1	1	0	1
1	1	64	2	142	42.1	1.10	20.8	145	74	235	85	150.00	5.50	1	1.00	1	1	1	1	1	1	1	1	0	0	1
1	1	44	2	151	64.1	1.80	28.0	118	75	238	53	185.00	5.70	1	1.00	1	0	1	0	0	1	0	0	1	0	1
0	0	63	2	149	49.2	.90	22.1	101	59	248	69	179.00	5.40	1	1.00	1	0	1	0	1	0	1	1	1	0	1
0	0	74	2	137	39.8	3.70	21.2	97	64	204	54	150.00	6.60	1	1.00	1	1	1	0	1	0	1	1	0	1	0
0	0	57	2	149	45.7	1.10	20.6	110	65	191	81	110.00	5.20	1	1.00	0	1	1	0	0	0	0	1	0	0	1
0	0	60	2	145	49.7	1.00	23.5	119	67	163	62	101.00	5.70	1	1.00	1	1	1	1	1	0	0	1	0	0	1
0	0	62	2	150	49.3	3.10	22.0	134	76	218	94	124.00	5.30	1	1.00	0	1	1	1	0	0	1	1	0	1	1
0	0	75	2	147	54.4	-.20	25.0	130	74	212	61	151.00	5.50	1	1.00	1	1	1	1	1	1	1	0	0	1	1
0	0	67	1	160	60.2	.20	23.5	138	82	236	75	161.00	5.40	0	1.00	0	0	1	1	1	0	1	1	0	0	1
1	1	66	1	164	72.6	.60	27.0	123	74	178	42	136.00	5.80	1	1.00	1	1	1	1	1	1	1	0	1	1	1
1	1	49	1	177	78.3	.20	24.9	135	86	224	44	180.00	10.60	0	1.00	1	1	0	0	1	1	1	1	1	0	0
1	1	65	2	155	48.2	.50	20.1	120	69	240	94	146.00	5.40	1	1.00	1	1	1	0	1	1	1	1	1	0	1
1	1	46	1	171	70.4	-.30	24.0	121	74	194	58	136.00	4.90	0	1.00	0	0	0	1	1	0	1	1	1	1	1
0	0	59	1	168	64.9	2.10	23.0	124	75	147	40	107.00	5.20	0	1.00	0	0	1	1	1	1	1	1	1	1	1
1	1	66	2	156	55.3	-1.90	22.8	95	58	232	64	168.00	5.30	1	1.00	1	0	1	0	1	0	0	1	0	0	1
1	1	35	2	155	54.4	.50	22.5	99	58	143	59	84.00	5.20	1	1.00	1	1	1	0	1	0	1	1	1	1	1
0	0	73	2	150	54.0	.90	23.9	123	55	230	63	167.00	6.10	1	1.00	1	0	1	0	1	0	1	1	0	0	1
1	1	62	1	177	79.6	6.20	25.5	121	78	173	64	109.00	5.30	1	1.00	1	0	1	0	0	1	1	0	0	1	1
1	1	65	1	166	76.6	4.70	27.8	125	79	173	57	116.00	5.40	1	1.00	0	1	1	1	1	1	1	0	0	1	1
0	0	70	1	163	61.6	.00	23.3	143	80	190	87	103.00	5.70	1	1.00	0	0	1	1	0	1	1	1	0	1	1
1	1	62	1	168	62.0	-4.00	22.0	127	82	207	60	147.00	5.60	1	1.00	0	0	1	0	0	0	0	1	0	1	1
0	0	59	2	145	48.0	3.30	22.8	144	88	183	61	122.00	5.40	1	1.00	1	1	1	1	1	1	0	1	0	0	1
1	1	63	2	143	56.5	2.30	27.7	121	72	182	61	121.00	5.70	1	1.00	1	1	1	0	1	1	1	0	0	1	1
1	1	40	2	159	47.8	1.70	18.9	95	63	252	90	162.00	5.00	1	1.00	1	1	1	0	0	1	0	1	1	0	1
1	1	71	2	156	78.0	.20	31.9	127	84	215	52	163.00	5.60	1	1.00	1	1	1	0	0	0	0	0	0	1	1
0	0	63	2	152	54.3	-.20	23.3	129	86	279	45	234.00	8.20	1	1.00	1	1	1	0	1	0	1	1	1	0	0
1	1	34	2	156	51.8	-2.80	21.4	106	64	162	75	87.00	5.20	1	1.00	1	0	1	1	1	0	1	1	1	1	1
1	1	28	2	154	53.5	.20	22.5	93	55	208	86	122.00	5.20	1	1.00	1	1	1	1	1	0	1	1	1	1	1
0	0	66	2	154	47.2	1.50	19.9	102	69	266	115	151.00	5.50	1	1.00	1	1	1	1	1	1	1	1	1	0	1
1	1	65	2	143	60.7	-2.80	29.5	142	87	248	53	195.00	5.60	1	1.00	1	0	1	0	1	1	1	0	0	0	1

1	1	31	2	155	54.1	5.90	22.5	101	62	212	73	139.00	5.20	1	1.00	1	1	1	0	1	1	1	1	1	1	1
0	0	68	1	159	66.1	-3.70	26.1	138	82	183	73	110.00	5.50	1	1.00	1	1	1	1	1	1	1	0	1	1	1
1	1	70	2	148	48.5	-.70	22.2	129	76	203	75	128.00	5.40	1	1.00	1	0	1	0	1	0	0	1	0	0	1
1	1	60	2	145	35.6	-.40	16.8	114	58	221	76	145.00	5.60	1	1.00	1	0	1	0	1	0	1	1	1	0	1
0	0	63	1	162	72.9	4.10	27.9	123	61	205	49	156.00	5.70	1	1.00	0	0	1	1	1	1	0	0	0	1	1
1	1	68	2	151	52.6	-.10	23.1	126	74	246	76	170.00	5.40	1	1.00	1	1	1	0	1	1	0	1	1	0	1
1	1	36	2	153	72.4	1.50	30.8	132	79	270	74	196.00	5.30	1	1.00	1	1	1	0	1	0	0	0	1	0	1
1	1	63	1	162	75.0	-.80	28.6	127	80	165	41	124.00	5.40	1	1.00	0	1	1	1	0	1	1	0	0	1	1
0	0	47	2	154	57.3	-.30	24.0	127	81	248	59	189.00	6.20	0	1.00	0	1	1	1	1	1	1	1	1	0	1
1	1	69	1	159	64.3	-2.00	25.5	138	73	205	45	160.00	5.80	1	1.00	0	1	1	0	1	1	0	0	1	1	1
1	1	66	2	145	53.2	-.30	25.1	110	65	221	75	146.00	5.50	1	1.00	1	1	1	1	1	1	1	0	1	0	1
0	0	37	1	165	78.2	-.40	28.6	152	80	215	36	179.00	5.50	0	1.00	0	0	1	0	1	1	1	0	0	0	1
0	0	42	2	161	78.9	2.20	30.4	156	82	192	36	156.00	5.40	1	1.00	0	0	1	1	1	1	1	0	0	0	1
1	1	63	2	153	55.8	.90	23.9	144	70	220	74	146.00	5.30	1	1.00	1	0	1	1	1	0	0	1	0	0	0
1	1	28	1	168	56.0	4.80	19.9	105	59	175	85	90.00	4.40	0	1.00	1	0	1	0	1	1	1	1	1	1	1
1	1	70	1	164	77.6	-7.00	28.9	138	84	194	45	149.00	5.90	1	1.00	1	1	1	1	1	1	1	0	1	1	1
0	0	65	2	148	52.7	4.00	24.1	119	57	200	83	117.00	5.40	1	1.00	1	1	1	1	1	1	1	1	0	1	1
1	1	52	2	150	62.5	.20	27.7	147	89	183	69	114.00	6.00	1	1.00	1	0	1	1	1	1	1	0	0	1	1
1	1	63	1	167	60.2	-.60	21.7	145	80	176	43	133.00	6.00	0	1.00	0	0	1	1	1	1	1	1	0	1	0
1	1	66	2	159	50.8	-1.50	20.1	102	62	235	90	145.00	6.30	1	1.00	1	1	1	1	1	1	1	1	1	0	1
1	1	76	2	150	65.2	-2.70	28.9	117	70	190	47	143.00	5.20	1	1.00	1	0	1	0	1	0	0	0	1	1	1
1	1	40	2	166	51.8	-1.50	18.8	106	73	234	61	173.00	5.60	1	1.00	1	1	1	0	0	0	0	1	1	0	1
0	0	28	2	151	52.7	-.50	23.1	126	79	189	107	82.00	5.10	0	2.00	0	1	1	0	1	1	1	1	1	1	1
1	1	39	2	153	53.9	5.30	23.0	131	74	196	80	116.00	5.70	1	1.00	1	1	1	0	1	0	1	1	1	1	1
1	1	70	1	163	58.2	.20	22.0	122	76	235	52	183.00	5.40	0	1.00	1	0	1	0	1	0	1	1	1	0	1
0	0	67	2	150	55.4	-.30	24.7	122	69	249	83	166.00	5.70	1	1.00	1	0	1	0	1	0	0	1	1	0	1
1	1	34	2	155	49.2	-3.40	20.4	105	61	186	86	100.00	5.50	1	1.00	0	1	1	1	1	1	1	1	1	1	1
0	0	51	2	155	57.4	-.60	23.9	121	77	280	97	183.00	6.90	1	1.00	1	0	1	1	0	1	1	1	1	0	0
1	1	49	1	171	65.9	.90	22.6	124	77	252	77	175.00	8.80	1	1.00	0	0	1	0	1	1	1	1	1	0	0
1	1	68	2	148	52.9	3.10	24.0	153	97	246	58	188.00	5.40	1	1.00	1	0	1	0	0	0	1	1	0	0	1
1	1	71	1	167	74.2	.50	26.5	121	65	215	43	172.00	6.20	0	1.00	1	0	1	1	1	1	1	0	0	1	1
1	1	71	2	149	55.0	-1.80	24.6	135	73	238	72	166.00	5.70	1	1.00	1	0	1	1	0	1	1	1	1	0	1
1	1	60	1	163	68.0	-4.40	25.5	146	87	152	79	73.00	5.60	1	1.00	0	0	1	1	1	1	1	0	0	1	1
1	1	62	2	149	46.6	-7.10	21.1	125	81	253	92	161.00	5.60	1	1.00	1	1	1	0	1	0	0	1	1	0	1
1	1	63	1	171	77.2	2.00	26.5	123	77	203	69	134.00	5.70	1	1.00	0	0	1	1	0	0	0	0	0	1	1
1	1	72	1	155	51.3	-2.80	21.2	114	76	176	42	134.00	5.10	1	1.00	1	0	1	0	0	0	0	1	0	1	1
1	1	64	1	159	67.8	-3.30	26.7	163	96	152	56	96.00	5.30	0	1.00	0	1	1	0	1	0	1	0	0	1	1
1	1	54	1	162	69.9	2.00	26.6	117	72	207	68	139.00	5.70	1	1.00	0	1	1	0	1	1	1	0	1	1	1
0	0	54	2	155	52.8	3.10	21.9	108	63	249	34	215.00	5.70	1	1.00	0	1	1	0	1	0	0	1	1	0	1

1	1	54	2	150	52.4	1.20	23.2	117	70	207	69	138.00	5.90	1	1.00	1	0	1	0	1	1	1	1	1	1	1
1	1	69	2	146	61.8	-.70	28.9	121	63	223	45	178.00	5.90	1	1.00	1	0	1	1	1	1	1	0	0	0	1
0	0	40	1	166	86.3	4.10	31.1	116	71	202	57	145.00	5.80	1	1.00	1	1	1	0	1	1	1	0	1	1	1
0	1	49	1	160	50.5	3.40	19.7	142	86	198	52	146.00	5.90	1	1.00	1	0	1	0	0	0	1	1	0	1	1
0	0	83	2	150	60.5	-4.70	26.8	113	60	197	46	151.00	6.40	1	1.00	1	0	1	0	1	0	1	0	0	0	1
1	1	72	1	155	60.7	.40	25.3	118	81	200	54	146.00	6.10	1	1.00	1	0	1	0	1	0	0	0	0	1	1
0	0	75	2	146	44.3	1.20	20.9	137	78	158	64	94.00	5.80	0	1.00	0	1	1	1	1	0	1	1	0	1	1
0	0	66	2	152	47.8	4.00	20.6	155	86	233	81	152.00	5.90	1	1.00	1	0	1	1	1	1	1	1	0	0	1
0	0	63	2	148	49.8	.40	22.8	107	66	206	45	161.00	5.30	1	1.00	0	0	1	1	1	0	0	1	1	1	1
1	1	62	1	159	60.9	.50	24.0	117	64	157	41	116.00	5.30	0	1.00	0	0	1	1	1	1	1	1	0	1	1
1	1	70	2	140	36.4	.10	18.4	117	58	180	81	99.00	5.30	1	1.00	1	0	1	1	1	0	1	1	1	1	1
1	1	52	1	165	58.5	1.30	21.4	117	67	136	51	85.00	6.00	1	1.00	1	0	1	0	1	0	1	1	1	1	1
0	0	62	2	147	48.2	1.70	22.2	105	65	255	70	185.00	5.80	1	1.00	1	0	1	0	0	0	0	1	1	0	1
0	0	69	2	151	46.5	.30	20.5	131	76	200	80	120.00	5.10	1	1.00	1	0	1	1	1	0	0	1	1	1	1
1	1	69	1	163	59.9	4.60	22.5	129	69	211	58	153.00	5.70	1	1.00	0	1	1	0	1	0	1	1	0	1	1
1	1	64	2	158	45.2	3.70	18.1	109	68	89	36	53.00	7.70	1	1.00	1	0	1	1	1	1	0	1	1	0	0
0	0	43	2	155	52.5	4.40	21.8	128	79	190	66	124.00	5.10	1	1.00	1	0	1	0	1	0	0	1	1	1	1
1	1	35	2	155	50.9	.80	21.0	100	59	159	69	90.00	5.10	1	1.00	1	0	1	0	1	1	1	1	1	1	1
1	1	84	1	156	49.8	-1.70	20.4	129	65	176	50	126.00	6.00	1	1.00	1	0	1	1	1	0	1	1	1	0	1
1	1	66	2	154	45.6	.50	19.2	90	59	167	67	100.00	6.10	1	1.00	1	0	1	1	1	1	1	1	1	1	1
1	1	72	1	165	69.2	.00	25.2	136	94	169	49	120.00	5.80	1	1.00	0	0	1	1	1	1	1	0	0	1	1
1	1	66	2	142	50.5	.00	24.9	91	55	225	57	168.00	5.50	1	1.00	1	0	1	1	1	0	1	1	1	0	1
1	1	72	1	167	67.8	-.10	24.4	110	67	207	59	148.00	6.00	1	1.00	1	0	1	0	1	1	1	1	1	1	1
1	1	36	2	154	73.7	3.30	31.1	126	90	191	32	159.00	6.10	1	1.00	1	1	1	0	1	0	0	0	0	0	1
1	1	62	2	146	58.6	-.60	27.6	126	67	176	50	126.00	5.70	1	1.00	1	0	1	0	1	1	0	0	0	1	1
1	1	60	2	155	47.1	.40	19.5	152	92	183	63	120.00	5.50	1	1.00	0	1	1	1	1	1	1	1	0	1	1
1	1	64	1	172	77.0	2.60	26.1	128	76	187	43	144.00	6.50	1	1.00	1	0	1	0	1	0	1	0	0	0	0
1	1	48	1	172	62.0	5.00	20.9	134	78	173	68	105.00	5.20	0	1.00	0	0	1	0	1	1	1	1	1	0	1
1	1	72	1	160	60.0	-.70	23.4	92	50	243	49	194.00	5.40	1	1.00	1	0	1	1	1	1	1	1	1	0	1
1	1	42	2	139	34.6	-.40	17.9	99	71	144	63	81.00	5.50	0	1.00	0	0	0	0	1	0	0	1	1	1	1
1	1	65	1	166	66.7	.70	24.2	125	72	212	38	174.00	5.20	1	1.00	0	0	1	1	1	0	1	1	1	0	1
1	1	57	2	165	54.6	.20	20.1	118	76	266	69	197.00	5.40	1	1.00	1	0	1	1	0	0	1	1	1	0	1
1	1	52	2	161	52.5	1.30	20.1	113	72	226	59	167.00	5.60	1	1.00	1	0	1	1	1	1	0	1	1	0	1
1	1	69	2	152	47.9	3.30	20.7	89	55	192	103	89.00	5.40	1	1.00	1	1	1	1	1	0	1	1	1	1	1
1	1	83	1	157	48.7	2.30	19.8	129	63	149	62	87.00	6.20	1	1.00	0	0	1	0	1	0	1	1	1	1	0
1	1	37	2	155	72.0	4.00	29.8	119	66	203	66	137.00	6.00	1	1.00	1	0	1	1	1	0	1	0	1	1	1
0	1	69	1	156	63.0	.00	25.9	102	66	229	61	168.00	5.80	1	1.00	0	1	1	0	1	1	0	0	1	0	1
0	0	49	2	147	44.0	1.70	20.5	104	69	164	62	102.00	6.00	1	1.00	1	1	1	0	1	1	1	1	1	1	1
1	1	75	2	146	55.1	-.10	25.9	115	66	218	81	137.00	5.70	0	1.00	1	1	1	0	1	0	0	0	1	1	1

0	0	64	1	165	73.6	-2.40	26.9	164	109	260	60	200.00	5.40	1	1.00	1	0	1	0	0	0	0	0	0	0	1
1	1	59	2	142	56.5	-1.40	28.2	115	76	216	62	154.00	5.90	1	1.00	1	0	1	1	1	1	1	0	0	0	1
1	1	62	1	163	71.4	-.20	26.9	119	76	214	60	154.00	5.90	1	1.00	0	1	1	0	1	0	1	0	0	1	1
0	0	50	1	168	80.3	9.10	28.5	135	86	246	62	184.00	10.60	0	1.00	0	1	1	0	1	0	0	0	1	0	0
0	0	39	1	181	153.3	-2.00	46.5	165	93	211	55	156.00	6.10	0	1.00	1	1	0	1	1	1	1	0	0	1	1
1	1	48	1	168	69.8	.30	24.7	117	74	185	47	138.00	5.70	1	1.00	0	0	1	1	1	1	0	1	1	1	1
1	1	73	1	167	60.9	-.80	21.7	118	73	269	68	201.00	8.00	1	1.00	0	0	1	0	0	0	1	1	1	0	0
1	1	39	2	166	66.7	3.40	24.3	95	62	242	85	157.00	5.60	1	1.00	1	1	0	0	0	1	1	1	1	0	1
0	0	62	2	152	59.4	3.80	25.5	101	66	254	76	178.00	5.90	1	1.00	1	0	1	0	0	0	0	0	1	0	1
1	1	51	2	149	49.6	.90	22.3	111	73	225	71	154.00	5.40	1	1.00	1	0	1	1	1	1	0	1	1	0	1
1	1	85	1	145	36.8	1.80	17.5	115	61	198	68	130.00	5.40	1	1.00	1	0	1	0	1	1	1	1	1	1	1
1	1	78	1	158	62.2	-2.10	24.8	143	81	243	42	201.00	5.50	1	1.00	0	0	1	0	1	1	1	1	0	0	1
0	0	67	1	168	65.4	2.70	23.0	112	72	165	61	104.00	5.70	1	1.00	0	0	1	0	1	1	0	1	1	1	1
0	0	50	2	156	56.6	2.30	23.3	103	56	252	67	185.00	5.70	1	1.00	1	1	1	1	1	0	0	1	1	0	1
1	1	46	2	165	53.4	2.00	19.7	101	70	222	55	167.00	5.10	1	1.00	1	0	1	1	1	1	1	1	1	0	1
1	1	63	2	160	49.7	-2.70	19.5	107	75	251	80	171.00	5.70	0	1.00	1	0	1	0	1	0	0	1	0	0	1
0	0	46	2	148	38.7	-.80	17.6	102	59	180	73	107.00	5.70	1	1.00	1	0	1	0	0	1	1	1	1	1	1
1	1	54	2	149	50.1	-2.60	22.6	129	74	220	46	174.00	5.70	1	1.00	1	0	1	0	1	0	0	1	1	0	1
1	1	55	1	160	41.9	.00	16.4	94	55	197	90	107.00	5.70	1	1.00	0	1	1	1	1	0	0	1	1	1	1
0	0	80	1	161	68.9	-2.70	26.7	128	63	220	87	133.00	5.60	0	1.00	0	0	1	0	1	1	1	0	0	0	1
1	1	65	1	160	56.1	-4.20	21.8	115	68	142	47	95.00	6.50	1	1.00	0	1	1	1	0	0	1	1	1	1	0
1	1	61	2	156	67.2	.60	27.6	112	71	230	64	166.00	5.70	1	1.00	1	0	1	0	1	0	1	0	0	0	1
1	1	71	1	165	58.6	1.30	21.4	131	73	183	56	127.00	5.40	1	1.00	1	0	1	1	1	1	1	1	0	1	1
1	1	50	2	152	55.5	.00	24.1	160	92	230	76	154.00	5.20	1	1.00	1	0	1	0	0	0	1	1	0	0	1
1	1	78	2	135	40.4	-1.20	22.2	105	51	206	78	128.00	5.40	1	1.00	1	0	1	0	1	0	0	1	0	1	1
1	1	73	1	157	60.5	3.60	24.4	137	77	142	49	93.00	5.90	1	1.00	1	1	1	1	1	1	1	1	1	1	1
1	1	40	2	154	41.3	1.70	17.3	96	65	159	82	77.00	4.80	1	1.00	1	0	1	1	1	1	1	1	1	1	1
1	1	74	1	161	53.2	-.20	20.4	142	71	191	50	141.00	5.90	1	1.00	0	1	1	0	1	1	1	1	0	1	1
0	0	71	2	149	44.8	4.10	20.3	128	88	253	83	170.00	5.40	1	1.00	1	0	1	1	1	1	1	1	0	0	1
1	1	71	2	151	42.5	.80	18.7	95	59	234	76	158.00	5.40	1	1.00	1	0	1	1	1	1	0	1	1	0	1
0	0	57	2	156	60.1	-3.30	24.5	113	59	219	62	157.00	5.20	1	1.00	1	1	1	0	1	0	0	1	1	1	1
0	0	68	1	159	66.0	1.70	26.2	114	80	166	53	113.00	5.30	1	1.00	0	1	1	1	1	0	0	0	0	1	1
0	0	59	2	151	44.5	4.30	19.6	121	76	204	59	145.00	5.50	1	1.00	1	0	1	0	1	0	0	1	1	1	1
0	0	63	1	161	71.2	-1.00	27.4	129	87	211	92	119.00	6.30	1	1.00	0	0	1	0	0	0	0	0	1	1	1
0	0	68	2	144	57.0	-.10	27.5	141	68	216	97	119.00	5.50	1	1.00	1	0	1	0	1	0	0	0	0	1	1
1	1	57	2	148	46.6	.00	21.3	124	78	229	57	172.00	6.20	1	1.00	1	1	1	1	1	0	0	1	1	0	1
0	0	73	1	163	59.0	-1.60	22.1	131	71	187	55	132.00	5.40	1	1.00	0	0	1	0	0	1	1	1	1	1	1
1	1	65	2	152	49.0	-.20	21.2	110	56	207	62	145.00	5.40	1	1.00	1	0	1	0	1	0	0	1	1	1	1
1	1	70	1	169	86.4	-3.60	30.3	115	62	161	41	120.00	5.90	0	1.00	1	0	1	0	1	1	1	0	1	1	1

1	1	80	2	151	44.9	-3.00	19.6	109	59	227	81	146.00	5.80	1	1.00	1	1	1	1	1	1	1	1	0	0	1
1	1	56	2	153	50.4	.60	21.6	145	83	227	79	148.00	5.90	1	1.00	1	0	1	0	1	1	1	1	0	0	1
1	1	42	1	169	79.4	-2.10	27.9	132	85	262	78	184.00	5.20	1	1.00	0	0	1	1	1	1	1	0	1	0	1
1	1	59	1	163	67.7	1.10	25.4	118	76	155	53	102.00	5.40	1	1.00	0	1	1	0	1	0	0	0	1	1	1
1	1	24	2	155	43.3	1.50	17.9	102	67	282	75	207.00	5.20	1	1.00	1	0	0	1	1	1	1	1	1	0	1
1	1	34	1	178	80.1	-.20	25.1	113	58	197	54	143.00	5.30	0	1.00	0	0	1	0	1	1	1	0	1	1	1
1	1	62	1	170	65.5	-.40	22.7	137	84	206	50	156.00	5.30	1	1.00	0	1	1	1	1	1	1	1	1	1	1
1	1	76	2	149	56.6	.30	25.6	108	63	190	70	120.00	5.80	1	1.00	1	1	1	1	1	0	1	0	0	0	1
1	1	63	2	145	47.1	1.30	22.5	151	80	201	74	127.00	5.30	1	1.00	1	1	1	1	1	0	1	1	0	1	1
1	1	71	2	155	55.1	1.50	23.0	143	85	171	59	112.00	5.80	1	1.00	1	0	1	1	1	1	1	1	0	0	1
1	1	57	2	145	46.6	.10	22.1	112	77	187	47	140.00	6.00	1	1.00	0	1	1	1	1	0	1	1	1	0	1
1	1	60	1	168	61.4	.00	21.8	110	71	175	50	125.00	5.80	1	1.00	0	0	1	0	1	0	0	1	1	1	1
1	1	62	2	150	76.8	1.40	33.9	139	80	177	53	124.00	7.10	1	1.00	1	0	1	0	1	0	1	0	1	0	0
1	1	47	2	153	62.8	-1.60	26.8	142	85	163	34	129.00	5.40	1	1.00	1	1	1	0	1	0	0	0	0	0	1
1	1	72	1	156	61.0	2.30	25.0	143	88	130	41	89.00	5.80	1	1.00	0	0	1	0	1	0	0	0	0	1	1
0	0	58	1	167	58.0	1.30	20.9	136	79	179	48	131.00	5.40	0	1.00	0	1	1	0	1	1	1	1	1	1	1
1	1	45	2	154	48.8	1.90	20.5	98	59	223	67	156.00	5.90	1	1.00	1	0	1	0	1	0	1	1	1	0	1
1	1	69	1	150	62.3	-2.60	27.6	156	90	157	60	97.00	6.00	1	1.00	0	0	1	1	1	1	1	0	0	1	1
1	1	66	2	149	46.7	4.50	20.9	146	82	168	68	100.00	5.50	1	1.00	1	1	1	0	1	1	1	1	0	1	1
1	1	78	2	142	44.2	-1.20	22.0	136	52	184	57	127.00	5.40	1	1.00	1	1	1	0	1	0	1	1	1	1	1
1	1	80	2	153	50.4	1.10	21.6	152	85	238	66	172.00	5.20	1	1.00	1	0	1	0	1	0	0	1	0	0	1
1	1	53	1	157	62.8	-.60	25.3	102	66	238	38	200.00	6.10	1	1.00	1	0	1	1	0	1	1	0	1	0	1
0	0	39	2	150	52.2	5.20	23.2	106	64	164	58	106.00	5.50	1	1.00	1	0	1	0	1	1	1	1	1	1	1
0	0	24	2	149	50.6	-1.30	22.7	88	50	164	85	79.00	5.10	1	1.00	0	1	0	0	1	0	0	1	1	1	1
1	1	38	2	159	51.5	-1.30	20.2	110	69	214	103	111.00	5.30	0	1.00	0	0	0	0	1	1	1	1	1	1	1
1	1	60	2	152	59.9	-.50	26.0	130	81	234	48	186.00	6.00	1	1.00	1	0	1	0	0	1	1	0	1	0	1
0	0	67	1	172	68.1	.30	22.9	167	87	213	94	119.00	5.50	1	1.00	0	0	1	1	1	1	1	1	0	1	1
1	1	74	2	148	41.9	4.20	19.1	125	76	198	77	121.00	13.30	1	1.00	1	0	1	0	1	1	1	1	1	1	0
0	0	77	1	167	58.2	-.10	20.8	143	77	193	61	132.00	5.50	1	1.00	0	1	1	0	0	1	1	1	0	1	1
1	1	68	2	143	48.1	.40	23.4	100	63	202	53	149.00	5.80	1	1.00	1	1	1	1	0	1	1	1	1	1	1
1	1	60	2	152	43.4	3.00	18.7	145	80	224	70	154.00	5.50	1	1.00	1	1	1	0	1	1	0	1	0	0	1
0	0	67	2	142	53.2	1.20	26.2	124	76	162	46	116.00	5.40	1	1.00	1	0	1	0	1	0	1	0	0	1	1
1	1	69	1	166	63.9	2.00	23.1	126	90	208	50	158.00	5.30	1	1.00	1	1	1	1	1	1	1	1	0	1	1
1	1	40	2	156	58.7	.60	24.0	107	67	166	65	101.00	5.50	1	1.00	1	0	1	1	1	1	1	1	1	1	1
1	1	46	2	163	51.4	-.80	19.4	110	76	158	70	88.00	6.10	1	1.00	1	1	1	1	1	0	1	1	1	1	1
1	1	50	2	152	57.1	.80	24.6	116	74	291	72	219.00	5.80	1	1.00	0	1	1	1	1	0	1	1	1	0	1
1	1	60	1	169	66.6	.90	23.3	118	68	209	82	127.00	5.80	0	1.00	0	1	1	1	1	1	1	1	1	1	1
1	1	62	2	149	52.9	-1.50	23.8	130	71	239	41	198.00	6.00	1	1.00	1	1	1	1	0	1	0	1	0	0	1
0	0	68	1	162	69.4	-3.20	26.6	106	64	197	62	135.00	5.60	1	1.00	1	1	1	0	0	0	1	0	0	1	1

1	1	70	1	178	82.4	1.80	25.8	114	73	133	65	68.00	5.30	1	1.00	0	0	1	1	0	1	1	0	0	1	1
0	0	35	2	153	48.2	-.60	20.4	98	60	221	70	151.00	4.80	1	1.00	1	0	1	0	1	0	0	1	1	0	1
1	1	74	2	145	42.0	1.30	20.0	110	69	225	70	155.00	5.40	1	1.00	1	1	1	0	1	1	1	1	1	0	1
0	0	72	2	142	34.3	1.80	17.1	95	55	218	86	132.00	5.80	1	1.00	1	1	1	0	0	1	0	1	1	1	1
0	0	73	2	148	47.1	1.10	21.5	105	65	206	69	137.00	5.50	1	1.00	1	0	1	1	1	1	0	1	1	1	1
1	1	62	2	145	49.3	3.00	23.3	119	71	222	55	167.00	5.30	1	1.00	0	0	1	0	0	0	0	1	1	0	1
1	1	45	1	168	77.5	3.00	27.3	112	64	222	33	189.00	5.60	1	1.00	0	1	1	0	0	1	1	0	1	0	1
1	1	71	2	148	54.7	1.20	24.8	101	57	213	58	155.00	7.10	1	1.00	1	0	1	0	0	1	1	1	0	1	0
1	1	43	2	161	46.1	-1.00	17.8	101	57	164	92	72.00	5.20	0	1.00	0	0	1	1	1	1	1	1	1	1	1
1	1	58	2	143	45.9	.90	22.5	115	76	241	80	161.00	5.60	1	1.00	1	0	1	0	1	1	1	1	1	0	1
0	0	53	2	159	73.2	.20	28.8	119	67	183	51	132.00	6.80	0	1.00	1	1	1	0	1	0	1	0	0	0	0
0	0	80	2	146	58.2	1.40	27.4	129	72	225	87	138.00	5.80	1	1.00	1	1	1	1	1	1	1	0	0	0	1
1	1	75	2	147	52.6	.50	24.2	138	81	211	78	133.00	6.00	1	1.00	1	0	1	1	1	0	1	1	0	1	1
0	0	70	1	157	63.1	2.40	25.7	145	72	251	67	184.00	5.60	1	1.00	0	1	1	0	0	0	0	0	0	0	1
1	1	66	1	160	63.2	1.70	24.5	131	72	150	41	109.00	5.40	1	1.00	1	0	1	0	1	0	0	1	0	1	1
0	0	85	2	141	47.5	.50	23.8	168	90	207	39	168.00	5.80	1	1.00	1	0	1	1	1	1	1	1	0	0	1
0	0	69	1	169	61.6	-1.20	21.6	107	58	157	68	89.00	5.80	1	1.00	0	0	1	0	0	1	1	1	0	1	1
0	0	50	2	151	47.1	.40	20.6	97	59	265	62	203.00	5.30	1	1.00	1	1	1	0	0	0	0	1	1	0	1
0	0	62	2	141	44.7	1.70	22.6	130	79	188	68	120.00	5.40	1	1.00	1	0	1	0	0	0	0	1	1	1	1
0	0	70	2	152	56.1	1.80	24.4	129	66	239	91	148.00	5.70	1	1.00	1	0	1	1	0	0	0	1	0	0	1
1	1	74	2	141	42.2	-1.60	21.2	146	81	181	60	121.00	5.80	1	1.00	1	1	1	1	1	1	1	1	0	1	1
0	0	81	2	144	47.8	-.60	22.9	116	56	167	53	114.00	5.60	1	1.00	1	0	1	1	1	1	0	1	0	1	1
0	0	71	1	171	73.4	2.90	25.0	122	75	191	54	137.00	5.60	1	1.00	1	0	1	1	1	1	1	0	1	1	1
1	1	69	1	161	63.0	.00	24.2	135	75	153	70	83.00	6.10	1	1.00	0	1	1	1	1	1	1	1	0	1	0
1	1	38	2	147	79.2	-4.40	36.4	147	83	227	47	180.00	5.40	0	1.00	1	0	1	1	0	1	1	0	0	0	1
1	1	37	2	158	63.3	3.30	25.2	115	70	197	73	124.00	5.20	1	1.00	0	0	1	0	1	0	1	0	0	1	1
1	1	62	1	163	65.9	3.70	24.7	110	79	243	126	117.00	4.80	1	1.00	0	0	1	1	1	1	1	1	1	0	1
1	1	58	2	155	62.7	2.60	26.0	110	63	240	43	197.00	5.70	1	1.00	0	1	1	1	1	1	1	0	1	0	1
0	0	63	1	165	68.7	-3.40	25.3	137	82	216	73	143.00	5.70	1	1.00	0	0	0	0	0	1	1	0	1	1	1
1	1	62	2	159	54.6	2.80	21.5	112	66	209	76	133.00	5.80	1	1.00	1	0	1	1	1	1	1	1	1	1	1
1	1	61	1	163	67.0	.30	25.2	145	89	177	77	100.00	6.00	1	1.00	0	1	1	1	1	1	1	0	0	1	1
1	1	72	1	161	52.5	.60	20.3	118	62	192	102	90.00	5.20	1	1.00	0	1	1	1	1	1	1	1	0	1	1
0	0	43	1	165	70.8	2.00	26.0	124	74	185	50	135.00	5.40	0	1.00	0	0	1	0	1	0	0	0	1	1	1
0	0	41	2	154	52.7	-.10	22.1	102	60	219	130	89.00	4.90	0	1.00	0	1	0	0	1	0	0	1	1	1	1
1	1	31	2	158	53.1	.50	21.1	100	62	198	90	108.00	5.30	1	1.00	1	0	1	1	0	0	0	1	1	1	1
0	1	70	2	144	51.7	-.50	24.9	103	64	211	71	140.00	5.50	1	1.00	1	1	1	0	0	1	0	1	1	1	1
0	0	67	1	166	62.0	-.50	22.5	118	76	225	62	163.00	5.60	1	1.00	0	1	1	1	1	0	0	1	1	0	1
0	0	64	2	155	52.1	-1.00	21.7	137	76	231	61	170.00	5.60	1	1.00	1	0	1	0	1	1	0	1	0	0	1
1	1	74	2	151	57.7	1.70	25.4	109	64	189	58	131.00	6.70	1	1.00	1	0	1	1	1	0	0	0	1	1	0

1	1	61	2	150	54.5	.60	24.1	101	62	172	62	110.00	5.20	1	1.00	1	0	1	1	0	1	1	1	0	1	1
1	1	57	2	161	46.3	-.70	17.8	124	70	202	80	122.00	5.40	1	1.00	1	0	1	0	0	1	0	1	1	1	1
0	0	64	1	170	60.6	-.40	20.9	134	79	247	87	160.00	5.30	1	1.00	0	0	1	0	1	0	1	1	1	0	1
1	1	66	2	149	54.6	2.60	24.6	149	83	219	51	168.00	5.50	1	1.00	0	1	1	1	1	1	1	1	0	1	1
1	1	63	2	155	62.7	1.40	26.2	119	80	212	78	134.00	5.20	1	1.00	0	1	1	1	1	1	1	0	0	1	1
1	1	64	2	150	58.2	-.30	25.8	169	105	228	67	161.00	5.40	1	1.00	1	1	1	0	0	1	0	0	0	0	1
0	0	51	2	150	63.7	-.30	28.3	117	73	194	37	157.00	6.10	1	1.00	1	0	1	0	1	0	1	0	1	0	0
1	1	43	2	155	43.2	-.40	18.0	98	63	165	54	111.00	5.60	1	1.00	1	0	1	1	1	1	1	1	1	1	1
1	1	57	1	170	68.5	1.00	23.8	113	79	206	103	103.00	5.90	1	1.00	0	1	1	1	1	1	1	1	1	1	1
1	1	41	1	175	70.4	3.40	22.9	126	84	253	76	177.00	5.40	0	1.00	0	1	1	0	1	1	1	1	1	0	1
0	0	69	1	167	71.4	-1.70	25.4	129	80	162	40	122.00	6.80	1	1.00	1	1	1	1	1	0	1	0	0	1	0
1	1	66	2	150	64.3	-3.20	28.5	125	71	236	51	185.00	7.30	1	1.00	1	1	1	1	1	0	0	0	1	0	0
1	1	59	1	160	69.3	.00	27.0	125	71	304	31	273.00	5.40	1	1.00	0	0	1	0	1	1	1	0	1	0	1
1	1	71	1	164	63.4	-1.70	23.6	137	87	219	91	128.00	5.20	1	1.00	0	0	1	1	1	1	1	1	0	1	1
1	1	47	1	168	66.9	.10	23.8	126	85	221	56	165.00	5.80	0	1.00	0	1	1	0	1	1	1	1	1	0	1
1	1	71	2	156	61.1	-.90	25.0	115	73	239	58	181.00	5.70	1	1.00	1	0	1	1	0	1	1	0	1	0	1
1	1	39	1	163	78.7	-1.60	29.4	124	84	176	44	132.00	4.90	0	1.00	0	1	0	1	1	1	1	0	1	1	1
0	0	48	1	165	66.9	-1.50	24.6	120	72	293	47	246.00	5.50	0	1.00	0	1	1	0	1	0	0	1	1	0	1
0	0	54	2	150	46.2	-.20	20.4	93	64	269	78	191.00	5.80	1	1.00	0	1	1	1	1	1	1	1	1	0	1
0	0	64	1	169	77.5	1.30	27.1	160	104	228	95	133.00	5.70	1	1.00	0	0	0	0	0	1	1	0	0	0	1
1	1	29	2	149	61.0	3.40	27.5	106	57	165	56	109.00	5.90	1	1.00	1	0	1	0	1	0	1	0	1	1	1
1	1	52	1	166	75.0	-1.50	27.2	139	94	241	56	185.00	5.60	0	1.00	0	0	0	0	1	0	0	0	0	0	1
1	1	64	2	146	42.6	1.90	20.0	104	55	236	88	148.00	5.80	1	1.00	1	1	1	0	1	0	0	1	1	0	1
1	1	60	2	146	40.1	-.20	18.9	136	81	165	120	45.00	6.00	1	1.00	0	0	1	0	1	0	1	1	1	1	1
0	0	69	1	156	71.7	-.60	29.4	123	76	190	86	104.00	5.00	1	1.00	0	0	0	0	1	0	0	0	0	1	1
0	0	70	1	154	58.6	-1.30	24.6	129	85	182	66	116.00	5.70	1	1.00	0	0	1	0	1	0	0	1	1	1	1
0	0	70	2	149	51.0	-.20	22.8	128	70	186	53	133.00	4.80	1	1.00	1	0	1	0	0	1	0	1	0	1	1
1	1	36	1	162	41.8	.80	15.9	107	59	121	53	68.00	5.20	0	1.00	1	0	0	1	0	1	1	1	1	1	1
1	1	57	2	155	72.7	1.40	30.2	111	65	236	58	178.00	5.70	1	1.00	1	0	1	0	1	0	0	0	1	0	1
1	1	42	1	160	64.1	-1.30	25.1	113	68	153	39	114.00	5.50	1	1.00	0	0	1	1	0	1	0	0	1	0	1
1	1	31	2	152	76.6	-1.40	33.1	125	75	208	54	154.00	5.40	1	1.00	1	0	1	0	0	0	0	0	1	1	1
0	0	57	2	151	54.6	-2.70	23.8	115	65	168	71	97.00	5.50	1	1.00	1	0	1	1	0	0	0	1	1	1	1
0	0	64	2	154	54.2	-.90	22.9	124	80	204	46	158.00	5.50	1	1.00	1	0	1	0	1	0	0	1	0	1	1
0	0	64	1	168	84.9	2.80	30.0	101	71	207	48	159.00	6.00	1	1.00	1	0	1	0	0	1	1	0	0	1	0
0	0	62	2	143	54.3	-1.10	26.5	130	78	179	49	130.00	5.40	1	1.00	1	0	1	0	0	0	0	0	1	0	1
1	1	65	1	159	62.7	-2.20	24.7	147	90	217	53	164.00	5.80	1	1.00	1	1	1	1	1	1	1	1	0	1	1
1	1	74	1	167	62.9	.10	22.5	134	72	194	47	147.00	5.90	1	1.00	1	0	1	0	0	1	1	1	1	0	1
1	1	47	2	155	52.9	1.70	22.0	104	55	170	72	98.00	5.50	1	1.00	1	1	1	1	1	1	1	1	1	1	1
1	1	60	2	155	42.4	.10	17.7	130	71	188	78	110.00	5.40	1	1.00	1	1	1	1	0	0	0	1	1	1	1

1	1	82	1	153	46.4	1.00	19.8	94	48	204	67	137.00	6.10	1	1.00	1	0	1	1	1	1	1	1	0	1	1
1	1	43	2	158	47.8	2.20	19.2	138	85	147	84	63.00	5.40	1	1.00	0	0	1	1	1	1	1	1	1	1	1
1	1	70	1	167	79.2	.80	28.2	136	77	121	45	76.00	6.20	1	1.00	0	0	1	1	1	1	1	0	0	1	0
1	1	68	1	155	66.1	-10.40	27.5	115	72	203	38	165.00	6.90	1	1.00	0	0	1	0	1	1	0	0	1	0	0
1	1	71	2	151	51.4	1.20	22.5	146	82	219	42	177.00	5.40	1	1.00	1	0	1	1	1	1	1	1	0	1	1
1	1	48	2	161	52.2	-.30	20.0	111	66	249	75	174.00	5.30	1	1.00	0	0	1	1	1	1	1	1	1	0	1
1	1	62	2	158	60.5	.60	24.2	106	68	255	68	187.00	5.90	1	1.00	1	0	1	0	0	0	0	1	1	0	1
1	1	62	2	142	48.4	-1.30	24.1	124	76	221	73	148.00	5.70	1	1.00	1	0	1	0	1	0	1	1	1	0	1
1	1	43	1	171	67.8	2.00	23.1	116	70	251	51	200.00	5.40	1	1.00	0	1	1	0	1	1	1	1	1	0	1
1	1	73	2	150	65.1	1.10	28.9	132	78	195	49	146.00	5.60	1	1.00	1	0	1	1	1	1	1	0	1	1	1
1	1	62	2	154	46.5	1.00	19.5	110	75	215	101	114.00	5.90	1	1.00	0	0	1	1	1	0	0	1	0	0	1
1	1	79	2	151	43.5	-.90	19.2	120	59	178	49	129.00	5.60	1	1.00	1	0	1	1	1	1	1	1	0	1	1
1	1	60	2	151	47.6	1.50	20.9	153	97	251	89	162.00	5.80	1	1.00	1	0	1	1	1	1	1	1	0	0	1
1	1	70	2	147	63.9	3.80	29.7	134	74	178	72	106.00	5.90	1	1.00	1	0	1	1	1	0	0	0	1	1	1
1	1	79	2	148	60.6	-.20	27.5	114	68	192	58	134.00	6.00	1	1.00	0	0	1	1	1	1	1	0	0	1	1
1	1	71	2	144	51.3	.20	24.6	131	81	205	54	151.00	5.90	1	1.00	1	0	1	1	1	1	1	1	1	1	1
1	1	74	2	140	49.5	.30	25.2	149	81	162	37	125.00	6.50	1	1.00	0	0	1	1	1	1	1	0	0	0	0
1	1	72	2	153	59.6	1.20	25.6	90	63	211	68	143.00	5.50	1	1.00	1	0	1	1	1	1	1	0	1	1	1
1	1	39	2	152	59.6	1.70	25.8	104	62	203	53	150.00	5.20	1	1.00	1	0	1	0	1	1	1	0	1	1	1
1	1	75	2	143	58.3	.60	28.3	129	78	217	44	173.00	5.20	1	1.00	0	0	1	1	1	1	1	0	1	1	1
1	1	69	2	149	62.9	1.00	28.4	131	84	249	42	207.00	6.10	1	1.00	1	0	1	0	1	0	0	0	0	0	1
0	0	74	2	150	63.8	-1.70	28.4	127	75	255	51	204.00	5.50	1	1.00	1	0	1	1	1	1	1	0	1	0	1
1	1	74	1	156	64.5	4.50	26.3	101	66	170	42	128.00	6.00	0	2.00	1	0	1	1	1	1	1	0	1	1	1
1	1	64	2	154	50.9	2.00	21.5	107	68	187	71	116.00	5.70	1	1.00	1	0	1	1	1	1	1	1	1	1	1
1	1	62	2	156	58.6	-.60	24.2	123	76	173	78	95.00	5.70	1	1.00	1	0	1	1	1	0	1	1	1	1	1
1	1	70	1	164	66.9	1.90	24.8	124	60	241	59	182.00	5.60	0	1.00	0	0	1	1	1	1	1	1	0	0	1
1	1	71	1	161	53.3	1.30	20.6	142	74	144	45	99.00	5.90	1	1.00	1	1	1	1	1	1	1	1	0	1	1
1	1	71	2	136	41.3	.80	22.2	111	66	185	62	123.00	5.50	1	1.00	1	0	1	0	1	1	1	1	0	1	1
0	0	61	2	157	56.9	-1.10	23.2	111	76	279	79	200.00	5.90	1	1.00	1	0	1	0	0	1	0	1	1	0	1
1	1	69	2	150	48.8	2.50	21.8	123	72	219	45	174.00	5.90	1	1.00	1	1	1	1	1	1	1	1	1	1	1
0	0	76	1	161	56.2	1.40	21.6	90	55	209	72	137.00	5.30	1	1.00	1	0	1	1	1	0	0	1	1	1	1
1	1	77	1	173	63.0	1.10	21.0	121	69	185	67	118.00	5.70	1	1.00	1	0	1	1	1	1	1	1	0	1	1
1	1	38	2	161	44.3	-.50	17.1	97	60	163	64	99.00	5.30	1	1.00	1	0	1	0	1	0	0	1	1	1	1
1	1	57	1	167	58.6	1.80	20.9	112	65	196	54	142.00	6.20	0	1.00	0	0	1	0	1	1	1	1	1	1	0
1	1	67	1	162	72.4	3.50	27.5	117	68	237	71	166.00	7.00	0	2.00	1	1	1	1	1	1	1	0	1	0	0
1	1	57	1	170	69.4	2.70	23.9	119	78	218	69	149.00	5.20	1	1.00	1	0	1	1	1	1	1	1	0	1	1
1	1	58	2	152	60.9	-.30	26.4	119	74	231	56	175.00	5.70	1	1.00	1	0	1	0	1	0	1	0	1	0	1
0	0	60	2	161	49.3	2.20	19.0	128	81	228	54	174.00	5.20	1	1.00	1	0	1	0	1	0	0	1	1	0	1
1	1	67	1	155	41.1	.40	17.1	119	74	243	77	166.00	5.60	1	1.00	0	0	1	1	1	1	1	1	1	0	1

1	1	53	1	156	64.6	.00	26.4	119	76	205	44	161.00	5.70	0	1.00	0	0	1	0	0	0	0	0	1	1	1
0	0	62	2	160	57.8	.10	22.4	98	56	246	61	185.00	5.50	1	1.00	1	1	1	1	1	1	1	1	1	0	1
1	1	49	2	163	42.1	.60	15.8	103	63	163	63	100.00	4.80	1	1.00	0	0	1	1	1	0	0	1	1	1	1
1	1	76	2	156	61.1	2.90	25.1	123	59	207	33	174.00	5.30	1	1.00	1	0	1	1	1	1	1	0	0	0	1
1	1	43	2	148	45.8	1.10	21.0	126	72	201	63	138.00	5.60	0	1.00	1	0	1	0	0	1	0	1	0	1	1
1	1	65	1	154	53.0	.60	22.4	129	84	166	57	109.00	6.40	1	1.00	1	1	1	0	1	1	1	1	0	0	1
1	1	71	2	155	52.4	.30	21.9	145	81	189	60	129.00	7.00	1	1.00	1	0	1	1	1	0	0	1	0	1	0
1	1	82	1	148	53.6	2.80	24.5	117	67	187	54	133.00	5.30	0	1.00	0	1	1	0	1	0	1	1	0	1	1
1	1	62	1	166	46.3	.20	16.7	124	85	227	65	162.00	5.70	0	1.00	1	0	1	1	1	0	1	1	1	0	1
1	1	61	2	156	56.2	-1.20	23.2	143	87	172	45	127.00	5.10	1	1.00	1	0	1	0	0	0	0	1	0	1	1
1	1	62	1	170	70.8	-5.20	24.4	128	89	203	56	147.00	5.60	1	1.00	0	0	1	0	1	0	1	1	1	1	1
1	1	30	2	154	51.2	-.10	21.7	92	53	173	43	130.00	5.40	1	1.00	1	1	1	1	1	0	0	1	1	1	1
1	1	60	2	149	47.2	-3.20	21.3	94	57	202	79	123.00	5.30	1	1.00	1	1	1	1	0	0	0	1	1	1	1
1	1	26	1	167	67.2	-6.00	24.2	122	68	179	48	131.00	5.20	0	1.00	1	1	1	0	1	0	0	1	1	1	1
1	1	74	2	150	51.5	2.20	23.0	126	64	221	94	127.00	5.40	1	1.00	1	0	1	0	1	0	0	1	1	0	1
1	1	59	2	150	44.3	2.70	19.8	121	70	240	71	169.00	5.50	1	1.00	1	0	0	1	1	0	0	1	1	0	1
1	1	72	2	157	49.1	-.30	19.8	139	73	252	125	127.00	5.70	1	1.00	1	0	1	1	1	1	1	1	1	0	0
1	1	71	1	159	68.6	2.60	27.0	124	78	179	72	107.00	5.70	1	1.00	1	0	1	1	1	0	0	0	0	1	1
1	1	50	1	170	74.2	.60	25.6	136	89	173	58	115.00	5.40	0	1.00	0	0	1	1	0	1	0	0	0	1	1
1	1	51	2	159	59.8	-.10	23.6	119	72	214	62	152.00	5.10	1	1.00	1	0	1	1	1	0	0	1	1	1	1
1	1	60	1	166	65.4	4.20	23.8	99	64	230	65	165.00	5.40	0	2.00	1	0	1	0	1	1	0	1	1	0	1
1	1	73	1	162	67.7	-1.20	25.7	130	75	222	63	159.00	5.50	1	1.00	0	0	1	1	1	1	0	0	0	0	1
0	0	73	1	169	65.6	-1.30	22.9	117	66	127	50	77.00	6.30	0	1.00	0	0	1	1	1	0	0	1	1	1	1
0	0	63	1	164	55.1	4.10	20.4	128	79	217	93	124.00	5.50	1	1.00	0	1	1	0	0	0	0	1	0	0	1
0	0	68	2	151	52.8	-7.50	23.3	136	68	180	56	124.00	6.00	1	1.00	1	0	1	0	1	0	0	1	0	1	1
1	1	62	1	158	68.3	.50	27.3	132	83	197	65	132.00	5.60	1	1.00	0	0	1	1	0	0	0	0	0	1	1
1	1	33	2	158	48.2	.70	19.2	102	69	162	86	76.00	4.90	1	1.00	1	0	1	1	1	1	1	1	1	1	1
1	1	56	1	155	53.6	-2.50	22.2	107	70	185	54	131.00	5.20	0	1.00	0	1	1	0	0	0	1	1	1	1	1
1	1	73	2	151	52.6	-2.30	23.2	140	75	187	47	140.00	5.80	1	1.00	1	0	1	0	1	0	1	1	0	0	1
1	1	48	2	153	45.1	2.00	19.2	109	69	199	80	119.00	5.80	1	1.00	1	1	1	0	1	0	1	1	1	1	1
1	1	68	2	162	50.7	-2.60	19.3	130	75	233	48	185.00	5.80	1	1.00	1	1	1	0	1	1	1	1	1	0	1
0	0	47	2	155	50.5	3.20	21.0	106	59	197	100	97.00	5.20	1	1.00	1	1	1	1	1	0	1	1	1	1	1
1	1	40	2	155	44.6	.90	18.5	93	53	243	87	156.00	5.60	1	1.00	1	0	1	1	1	0	1	1	1	0	1
1	1	58	1	168	59.0	.40	20.8	131	80	214	69	145.00	5.50	1	1.00	1	1	1	0	1	0	0	1	1	1	1
1	1	68	2	147	52.3	1.90	24.1	109	66	214	77	137.00	6.00	1	1.00	0	1	1	1	1	1	1	1	1	1	1
1	1	62	1	165	65.4	-4.10	23.9	147	87	183	81	102.00	5.60	0	1.00	0	0	1	0	0	0	1	1	0	1	1
1	1	59	2	158	58.6	-2.90	23.4	138	78	257	93	164.00	9.30	1	1.00	0	1	1	1	1	1	1	1	1	0	0
1	1	67	2	147	41.3	-.20	19.0	136	78	255	76	179.00	6.90	1	1.00	1	1	1	0	1	1	1	1	0	0	0
0	0	52	1	156	43.3	-.90	17.7	108	72	181	42	139.00	5.20	1	1.00	1	0	1	0	1	0	1	1	1	1	1

1	1	62	1	170	71.1	.60	24.6	118	77	214	53	161.00	5.30	0	1.00	1	0	1	1	1	1	1	1	1	1	1
1	1	53	2	152	51.6	4.90	22.3	124	71	232	52	180.00	5.80	1	1.00	1	1	1	0	0	0	1	1	1	0	1
1	1	75	1	155	62.1	.00	26.0	143	83	174	60	114.00	5.90	1	1.00	1	1	1	1	1	1	1	0	0	1	1
1	1	62	1	159	66.6	1.90	26.3	141	81	231	52	179.00	5.80	1	1.00	0	1	1	1	1	0	0	0	0	0	1
1	1	58	2	152	57.4	-.10	25.0	119	81	192	59	133.00	5.60	1	1.00	1	1	1	0	1	1	0	0	1	1	1
1	1	74	1	159	46.2	-.50	18.2	104	61	163	48	115.00	5.70	1	1.00	1	1	1	1	1	0	1	1	1	1	1
1	1	68	2	147	54.5	3.30	25.0	160	84	230	70	160.00	5.60	1	1.00	0	0	1	1	1	0	1	0	0	0	1
1	1	62	1	157	61.3	-.40	24.7	131	80	196	52	144.00	6.00	1	1.00	1	1	1	0	1	0	0	1	1	1	1
0	0	59	2	153	48.0	1.50	20.3	120	82	166	64	102.00	5.60	1	1.00	1	1	1	0	1	1	0	1	0	1	1
1	1	70	1	166	58.0	.90	21.0	124	83	143	56	87.00	5.20	1	1.00	0	0	1	0	1	0	1	1	0	1	1
1	1	61	2	150	54.0	2.20	23.9	113	70	178	37	141.00	6.30	0	1.00	0	0	0	0	0	1	1	1	1	0	1
1	1	53	1	169	93.8	.20	32.7	132	82	201	44	157.00	6.70	1	1.00	0	1	1	0	0	1	0	0	1	1	0
1	1	63	1	162	64.3	-.60	24.4	169	104	169	53	116.00	6.30	0	1.00	0	0	0	0	1	1	1	1	0	1	1
0	0	73	1	166	67.1	1.20	24.2	113	81	221	56	165.00	5.50	1	1.00	1	1	1	0	1	1	1	1	0	0	1
0	0	54	1	164	76.1	.50	28.1	131	84	200	61	139.00	5.50	1	1.00	0	1	1	0	0	0	0	0	0	1	1
1	1	63	2	159	57.0	-.40	22.5	121	75	195	62	133.00	6.30	1	1.00	1	0	1	0	1	1	0	1	1	1	1
1	1	69	1	155	44.7	-1.40	18.7	132	79	168	80	88.00	6.40	0	1.00	0	0	1	0	1	0	0	1	1	1	1
1	1	69	2	140	40.8	3.00	20.9	97	56	213	51	162.00	5.70	1	1.00	1	1	1	0	0	0	0	1	1	0	1
1	1	59	2	156	51.9	.60	21.3	105	71	228	60	168.00	5.00	1	1.00	1	0	1	0	1	0	0	1	1	0	1
1	1	73	1	163	63.0	1.60	23.7	123	75	213	53	160.00	7.70	1	1.00	1	0	1	1	1	0	1	1	1	1	0
1	1	57	2	158	60.0	-1.70	24.1	130	79	252	54	198.00	5.70	1	1.00	0	0	1	0	0	1	1	1	1	0	1
1	1	70	1	163	77.2	.40	28.9	150	83	174	40	134.00	5.60	1	1.00	1	1	1	1	1	1	1	0	0	1	1
0	0	61	2	149	48.8	-.90	22.0	95	53	167	43	124.00	5.60	1	1.00	1	0	1	0	0	0	0	1	1	1	1
1	1	67	1	156	60.0	-1.10	24.7	128	74	196	45	151.00	5.80	1	1.00	0	1	1	0	1	0	1	1	0	1	1
1	1	41	2	163	50.6	-1.30	19.0	123	74	155	47	108.00	5.00	0	1.00	1	1	1	0	1	1	1	1	1	1	1
0	0	60	2	156	53.7	.80	22.0	93	55	190	85	105.00	5.80	1	1.00	1	1	1	1	0	0	0	1	1	1	1
0	0	66	2	157	66.6	4.90	27.0	116	77	283	74	209.00	5.50	1	1.00	0	1	1	0	1	1	0	0	0	0	1
0	0	79	2	147	47.1	-1.90	21.8	91	53	180	68	112.00	5.50	1	1.00	1	0	1	0	1	0	0	1	0	1	1
1	1	32	2	158	45.7	-.30	18.2	92	56	135	59	76.00	5.50	1	1.00	1	0	1	1	1	0	1	1	1	1	1
1	1	60	2	154	55.3	1.20	23.2	114	72	234	42	192.00	5.40	1	1.00	1	1	1	0	0	0	0	1	1	0	1
1	1	63	1	159	70.2	-.50	27.9	136	88	266	80	186.00	5.30	1	1.00	0	0	0	0	1	0	1	0	1	0	1
1	1	64	2	149	50.0	1.90	22.4	157	88	271	54	217.00	5.50	1	1.00	1	0	1	0	1	0	0	1	0	0	1
1	1	67	2	155	54.2	-2.80	22.6	135	73	295	54	241.00	5.70	1	1.00	1	0	1	0	0	0	1	1	1	0	1
0	0	53	2	148	55.7	.70	25.4	108	64	208	41	167.00	5.30	1	1.00	1	1	1	0	1	0	1	0	1	1	1
1	1	66	1	157	57.2	.00	23.2	128	82	206	66	140.00	6.30	1	1.00	0	1	1	0	0	0	0	1	0	1	1
1	1	36	2	153	51.7	-3.40	21.9	102	61	190	71	119.00	5.00	1	1.00	1	0	1	0	1	1	1	1	1	1	1
1	1	66	1	165	63.7	-.90	23.5	170	101	202	66	136.00	6.00	1	1.00	0	0	1	0	0	0	0	1	0	1	1
1	1	35	1	165	95.9	2.50	35.1	128	83	223	41	182.00	5.40	1	1.00	1	0	1	1	1	1	1	0	1	0	1
1	1	62	2	157	58.2	-1.90	23.7	128	68	264	58	206.00	6.00	1	1.00	0	1	1	1	1	1	1	1	1	0	1

0	0	65	2	147	67.7	-1.40	31.5	140	82	243	44	199.00	5.60	1	1.00	1	0	1	0	0	1	0	0	0	0	1
1	1	50	1	173	85.3	3.00	28.3	141	85	190	76	114.00	5.50	1	1.00	0	1	1	0	1	0	1	0	0	1	0
1	1	54	2	144	47.8	-3.30	22.9	112	65	179	64	115.00	5.70	1	1.00	1	0	1	0	1	1	0	1	1	1	1
1	1	55	2	153	48.0	-.80	20.5	136	81	213	46	167.00	5.20	1	1.00	1	1	1	0	1	1	0	1	1	1	1
0	0	69	2	157	63.1	1.30	25.5	116	60	173	58	115.00	5.70	1	1.00	1	1	1	1	1	1	0	0	0	0	1
0	0	82	2	146	60.7	-.60	28.3	107	64	193	74	119.00	5.50	1	1.00	1	0	1	0	1	0	0	0	0	0	1
1	1	63	2	152	52.0	-.50	22.4	118	65	163	65	98.00	5.60	1	1.00	1	0	1	0	1	1	0	1	1	0	1
1	1	63	2	147	40.4	1.00	18.7	109	67	193	51	142.00	5.50	1	1.00	1	0	1	0	1	1	1	1	1	1	1
0	1	65	2	148	46.7	1.90	21.3	96	63	244	47	197.00	6.00	1	1.00	1	0	1	0	0	1	0	1	1	0	1
0	0	68	1	163	52.0	-4.80	19.6	108	74	203	51	152.00	6.90	1	1.00	1	0	1	0	1	1	1	1	1	1	0
1	1	37	2	154	51.2	-.60	21.6	126	68	155	54	101.00	5.10	1	1.00	1	0	1	1	1	1	0	1	1	1	1
1	1	51	2	150	62.9	-1.40	27.8	116	75	181	54	127.00	5.60	1	1.00	1	0	1	0	0	1	1	0	1	1	1
1	1	63	1	167	74.2	-.90	26.4	131	87	218	69	149.00	5.80	1	1.00	0	1	1	1	1	1	1	0	1	1	1
1	1	68	2	150	52.6	3.60	23.4	108	65	230	57	173.00	5.60	1	1.00	1	0	1	0	1	1	0	1	0	0	1
1	1	42	2	161	62.5	2.70	24.1	119	60	239	100	139.00	5.10	1	1.00	1	0	1	0	0	1	1	1	1	0	1
1	1	55	2	161	71.0	-.10	27.5	122	76	222	80	142.00	5.30	1	1.00	1	1	0	0	1	0	0	0	1	0	1
1	1	60	2	148	58.1	1.60	26.6	107	71	236	70	166.00	6.40	1	1.00	1	0	1	1	0	1	1	0	1	0	1
1	1	59	2	148	60.5	-1.10	27.5	97	61	251	55	196.00	5.80	1	1.00	1	1	1	1	1	1	1	0	0	0	1
1	1	62	1	161	64.5	.50	24.8	117	70	240	44	196.00	5.60	0	1.00	0	0	1	1	1	0	1	1	1	0	1
1	1	57	2	159	50.5	1.10	19.9	108	63	221	99	122.00	5.40	1	1.00	1	1	1	1	1	1	1	1	1	0	1
0	0	62	2	154	66.6	2.20	28.0	113	70	237	79	158.00	5.80	1	1.00	1	0	1	0	0	1	1	0	1	0	1
1	1	61	2	148	53.5	.50	24.5	113	59	196	79	117.00	5.60	1	1.00	1	1	1	1	1	0	1	1	1	1	1
0	0	53	2	161	57.0	2.20	21.9	134	78	222	72	150.00	5.60	1	1.00	1	0	1	0	1	1	1	1	1	0	1
1	1	64	2	152	60.4	-1.00	26.1	121	69	210	77	133.00	5.90	1	1.00	1	0	1	1	1	0	1	0	0	1	1
1	1	31	2	149	52.3	2.10	23.5	101	56	202	80	122.00	4.90	1	1.00	0	1	0	0	0	1	1	1	1	1	1
0	0	67	1	161	64.0	1.20	24.5	142	81	227	57	170.00	5.20	1	1.00	0	1	1	1	1	0	1	1	0	0	1
0	0	56	1	161	61.0	1.70	23.5	138	85	219	90	129.00	5.80	1	1.00	0	0	1	0	1	0	1	1	0	1	0
0	0	42	1	165	66.3	2.10	24.3	135	81	218	77	141.00	5.50	0	1.00	0	1	1	0	1	1	1	1	1	1	1
0	0	35	2	159	49.3	2.70	19.4	96	57	203	72	131.00	5.40	1	1.00	1	1	1	1	1	1	1	1	1	1	1
1	1	74	2	156	48.3	.80	19.8	172	91	194	81	113.00	5.30	1	1.00	1	0	1	1	1	0	0	1	0	1	1
0	0	60	2	148	48.0	1.30	21.7	116	68	175	82	93.00	5.30	1	1.00	1	0	1	0	0	0	1	1	0	1	1
1	1	70	1	159	45.6	-3.70	18.0	140	72	156	79	77.00	5.90	1	1.00	0	1	1	1	1	1	1	1	0	1	1
1	1	54	2	157	58.3	.00	23.7	87	55	273	83	190.00	5.50	1	1.00	1	1	1	1	1	1	1	1	1	0	1
0	0	48	2	161	57.2	-.80	22.0	133	75	263	54	209.00	5.60	1	1.00	1	1	1	0	0	0	0	1	1	0	1
1	1	52	2	158	61.9	-1.20	24.7	93	62	212	87	125.00	5.80	1	1.00	1	1	1	1	1	0	1	1	1	1	1
1	1	42	2	160	56.5	1.80	22.1	97	59	197	66	131.00	5.10	1	1.00	1	1	1	0	1	1	1	1	1	1	1
1	1	47	1	176	64.0	14.10	20.6	118	83	179	100	79.00	4.90	0	1.00	0	1	1	0	1	0	0	1	0	1	1
1	1	70	1	153	44.0	.10	18.7	88	53	151	45	106.00	6.00	1	1.00	0	1	1	1	1	1	0	1	1	1	1
0	0	68	1	164	55.3	-3.90	20.6	109	69	153	61	92.00	6.20	1	1.00	1	1	1	0	1	0	0	1	0	0	1

1	1	56	2	156	62.5	2.10	25.6	118	75	208	94	114.00	5.50	1	1.00	1	1	1	0	1	1	1	0	1	1	1
1	1	60	2	151	54.4	7.80	23.9	109	64	283	48	235.00	6.50	0	2.00	1	1	0	0	0	0	0	1	0	0	0
1	1	63	1	164	65.3	-1.00	24.3	120	80	202	102	100.00	5.50	1	1.00	0	1	1	0	1	1	1	1	1	1	1
0	0	76	2	138	55.1	2.30	28.9	117	67	262	49	213.00	6.50	1	1.00	1	0	1	1	1	1	1	0	1	0	0
1	1	70	1	162	63.5	-4.60	24.1	116	76	200	49	151.00	5.30	1	1.00	0	1	1	1	1	0	0	1	1	1	1
0	0	64	2	147	68.5	-1.80	31.6	123	75	224	36	188.00	6.00	1	1.00	1	0	0	1	1	1	1	0	1	0	1
1	1	66	2	147	52.7	2.60	24.2	107	62	230	89	141.00	5.40	1	1.00	1	0	0	0	1	1	1	1	1	0	1
1	1	66	2	142	37.3	-1.70	18.5	114	67	227	98	129.00	5.30	1	1.00	1	0	1	0	1	1	0	1	1	0	1
1	1	32	2	155	37.6	-.60	15.7	109	65	217	80	137.00	5.20	1	1.00	1	0	1	1	1	1	1	1	1	1	1
1	1	46	1	167	64.7	.40	23.1	145	96	247	55	192.00	5.30	1	1.00	0	1	1	1	1	1	1	1	0	0	1
1	1	43	2	161	58.9	-1.00	22.7	116	62	216	77	139.00	5.60	1	1.00	0	0	1	0	0	0	1	1	1	1	1
1	1	62	1	166	77.1	-.20	27.9	140	77	248	50	198.00	5.60	0	1.00	1	0	1	0	1	0	0	0	0	0	1
1	1	70	1	161	66.1	-1.30	25.6	163	105	201	57	144.00	5.50	1	1.00	0	0	1	1	1	1	1	0	0	1	1
1	1	60	2	147	49.9	-1.00	23.0	123	75	222	81	141.00	5.50	1	1.00	1	1	1	1	1	1	1	1	1	0	1
0	0	69	2	156	49.1	.50	20.2	95	70	274	80	194.00	6.20	1	1.00	1	0	1	1	1	0	0	1	1	0	1
0	0	32	2	161	49.6	-2.30	19.0	112	67	277	91	186.00	5.30	0	1.00	1	0	0	0	0	0	0	1	1	0	1
1	1	66	2	149	45.3	-1.20	20.4	153	92	225	66	159.00	5.20	1	1.00	1	1	1	1	1	1	1	1	0	0	1
1	1	44	2	157	56.5	.20	23.0	126	76	219	44	175.00	6.10	1	1.00	1	0	1	1	1	1	1	1	1	1	1
1	1	76	2	151	63.2	-.60	27.6	137	75	174	52	122.00	6.70	1	1.00	1	0	1	1	1	1	1	0	0	0	0
0	0	20	1	168	106.9	11.10	37.7	144	84	222	38	184.00	5.70	1	1.00	1	1	0	0	0	0	0	0	0	0	0
0	0	48	2	159	107.2	-4.80	42.4	143	79	269	49	220.00	5.70	1	1.00	1	0	1	0	1	1	0	0	0	0	1
1	1	73	2	155	57.6	3.30	24.0	104	60	217	41	176.00	5.60	1	1.00	1	0	1	0	1	1	1	1	0	0	1
1	1	61	2	150	60.6	-1.20	26.9	142	86	179	84	95.00	5.70	1	1.00	1	0	1	0	1	1	1	0	0	0	1
1	1	45	2	162	74.6	-.40	28.5	128	84	182	53	129.00	5.10	1	1.00	1	0	1	1	1	0	0	0	1	1	1
1	1	26	2	153	43.0	-1.90	18.4	105	63	137	62	75.00	5.10	0	1.00	0	0	1	0	1	1	1	1	1	1	1
1	1	53	2	154	53.2	-3.00	22.4	125	75	239	68	171.00	5.40	1	1.00	1	0	1	1	1	0	0	1	1	0	1
1	1	78	1	158	55.9	2.20	22.5	121	71	178	46	132.00	5.80	1	1.00	1	1	1	0	0	0	0	1	0	1	1
0	0	67	2	147	53.9	.00	24.9	110	59	197	56	141.00	5.50	1	1.00	1	0	1	0	1	0	0	1	1	1	1
1	1	80	1	165	51.7	-.40	18.8	97	64	183	56	127.00	5.50	0	1.00	1	1	1	0	0	1	1	1	1	1	1
0	0	75	1	158	59.8	1.20	24.0	120	76	229	40	189.00	5.60	1	1.00	0	1	1	0	0	0	1	1	0	0	1
1	1	47	1	169	66.8	1.50	23.4	110	65	185	47	138.00	5.40	1	1.00	0	1	1	1	1	0	1	1	1	1	1
1	1	65	2	144	47.3	2.00	22.9	107	64	159	49	110.00	6.30	1	1.00	1	1	1	0	0	0	0	1	0	1	1
1	1	66	2	152	51.2	.70	22.1	149	80	263	43	220.00	6.00	1	1.00	1	0	1	0	0	1	1	1	0	0	1
1	1	62	2	158	52.5	-3.30	21.0	105	69	208	74	134.00	5.30	1	1.00	1	1	1	1	1	1	1	1	1	1	1
1	1	76	2	143	49.1	3.00	24.0	103	64	207	32	175.00	5.30	1	1.00	1	0	1	0	1	0	0	1	0	0	1
1	1	74	2	150	32.3	1.20	14.4	98	70	206	75	131.00	5.60	1	1.00	0	0	1	0	1	1	1	1	1	1	1
1	1	59	2	152	45.8	2.80	19.9	129	83	170	65	105.00	5.20	1	1.00	1	0	1	0	1	1	1	1	0	1	1
0	0	52	2	162	50.1	4.10	19.0	114	76	194	79	115.00	5.40	1	1.00	1	0	1	0	0	1	1	1	0	1	1
1	1	49	2	155	59.7	-1.40	24.7	112	67	191	73	118.00	5.30	1	1.00	1	0	1	1	1	1	1	1	1	1	1

0	0	49	2	159	48.0	-1.00	19.1	88	54	192	65	127.00	5.50	1	1.00	1	0	1	0	0	1	0	1	1	1	1
1	1	42	2	159	44.3	.50	17.5	90	61	171	79	92.00	5.20	1	1.00	1	0	1	1	1	1	0	1	1	1	1
1	1	52	1	163	81.1	-.10	30.5	139	84	284	41	243.00	6.20	1	1.00	1	0	1	0	1	0	0	0	0	0	0
1	1	68	1	165	63.0	1.20	23.1	136	81	167	58	109.00	5.20	0	1.00	0	0	1	1	1	1	1	1	1	1	1
1	1	62	1	172	62.4	4.60	21.1	128	80	174	52	122.00	5.90	1	1.00	0	0	1	0	1	0	0	1	1	1	1
1	1	51	1	170	63.9	3.60	22.1	141	92	218	61	157.00	5.60	0	1.00	0	0	0	0	1	0	1	1	0	1	1
1	1	68	2	158	74.9	-2.50	29.9	123	74	196	45	151.00	6.30	1	1.00	1	1	1	0	1	1	1	0	1	1	1
1	1	34	2	164	53.6	2.60	20.0	111	65	165	103	62.00	4.80	0	1.00	0	0	1	0	1	0	1	1	1	1	1
1	1	60	2	153	56.1	5.80	23.8	80	49	197	71	126.00	6.00	1	1.00	1	0	1	0	1	0	0	1	0	1	1
1	1	76	1	155	48.2	-1.60	20.0	97	64	164	71	93.00	5.10	1	1.00	0	0	1	1	1	1	1	1	1	1	1
1	1	63	1	168	70.6	-5.20	25.1	113	64	198	48	150.00	6.60	0	1.00	0	0	1	1	1	0	1	0	0	1	0
1	1	59	1	166	64.6	5.60	23.4	139	96	120	62	58.00	5.40	0	2.00	0	0	1	1	1	1	1	1	0	1	1
1	1	43	2	146	66.8	1.70	31.2	126	78	148	61	87.00	5.80	1	1.00	1	1	1	1	1	0	1	0	1	1	1
1	1	40	2	155	50.2	2.20	20.7	112	71	171	81	90.00	5.20	0	1.00	1	0	1	0	1	1	1	1	1	1	1
1	1	66	2	142	39.4	.60	19.5	116	66	187	45	142.00	5.80	1	1.00	1	1	1	1	1	1	1	1	0	1	1
1	1	57	2	158	52.5	1.00	20.9	119	72	196	72	124.00	6.00	1	1.00	1	0	1	1	1	0	0	1	1	1	1
0	0	67	2	154	62.3	3.50	26.1	116	59	197	56	141.00	6.00	1	1.00	1	0	1	0	0	0	0	0	1	0	1
1	1	64	2	138	45.6	.70	23.9	115	72	210	78	132.00	5.90	1	1.00	1	1	1	1	1	1	1	1	0	1	1
1	1	60	1	172	67.3	3.00	22.8	137	84	214	79	135.00	6.50	0	1.00	0	1	1	1	1	1	0	1	1	1	0
1	1	72	1	166	78.1	.00	28.3	146	91	174	66	108.00	5.10	1	1.00	0	0	1	1	0	1	1	0	0	1	1
1	1	76	1	163	63.3	.10	23.8	153	85	179	82	97.00	5.50	1	1.00	0	0	1	1	1	1	1	1	0	1	1
1	1	61	1	155	58.0	-2.30	24.1	129	78	164	51	113.00	5.60	1	1.00	0	0	1	1	1	1	0	1	0	0	1
1	1	36	2	168	59.7	1.30	21.2	99	56	180	64	116.00	5.20	1	1.00	1	0	1	1	1	1	1	1	1	1	1
1	1	73	1	156	60.3	1.30	24.7	97	60	161	59	102.00	5.30	0	2.00	1	1	1	0	1	1	1	1	1	1	1
1	1	66	2	153	44.1	.00	18.7	94	58	199	65	134.00	6.10	1	1.00	1	0	1	1	1	1	1	1	1	1	0
1	1	77	2	151	61.0	.90	26.7	121	66	183	69	114.00	5.00	1	1.00	1	0	1	1	1	0	1	0	0	1	1
0	0	55	1	168	59.9	-1.90	21.2	120	65	179	52	127.00	5.50	1	1.00	1	0	1	1	0	1	0	1	1	1	1
0	0	60	2	162	48.7	2.90	18.6	112	61	227	71	156.00	5.60	1	1.00	1	0	0	0	1	0	0	1	1	0	1
1	1	69	2	150	50.6	-1.40	22.3	125	78	232	68	164.00	5.90	1	1.00	1	0	1	1	1	0	0	1	1	0	1
1	1	62	2	158	49.4	-.20	19.8	90	58	227	55	172.00	5.60	1	1.00	1	0	1	0	1	0	0	1	1	0	1
1	1	64	2	149	62.7	2.30	28.3	126	82	218	60	158.00	5.90	1	1.00	1	0	0	0	1	1	1	0	1	1	1
1	1	54	2	149	43.8	4.30	19.6	129	83	283	62	221.00	6.20	1	1.00	1	0	1	1	1	0	0	1	0	0	1
1	1	48	2	157	63.9	-.10	25.9	105	66	237	73	164.00	5.40	1	1.00	0	0	1	1	1	1	1	0	1	0	1
1	1	62	2	154	52.5	1.70	22.2	99	56	161	53	108.00	5.50	1	1.00	1	0	1	1	1	1	1	1	1	1	1
0	0	39	1	172	70.0	2.10	23.6	105	69	166	43	123.00	5.50	1	1.00	1	1	1	0	1	1	1	1	1	1	1
0	0	38	2	153	44.2	-.10	18.9	100	62	189	88	101.00	5.30	1	1.00	1	1	1	0	1	1	0	1	1	1	1
1	1	41	2	159	56.0	1.00	22.1	102	59	173	82	91.00	5.60	1	1.00	1	1	1	0	1	1	1	1	1	1	1
1	1	74	1	167	74.8	2.00	26.7	133	76	166	60	106.00	6.60	1	1.00	0	0	1	1	1	1	1	0	0	1	0
0	0	75	2	150	51.1	-.60	22.6	144	85	236	71	165.00	5.80	1	1.00	1	0	1	1	1	1	1	1	0	0	1

1	1	74	1	177	89.6	-7.80	28.7	114	66	211	53	158.00	5.80	1	1.00	0	0	0	1	1	0	1	0	0	1	1
1	1	56	2	150	56.2	3.20	25.1	124	83	266	54	212.00	5.60	1	1.00	1	1	0	1	1	1	1	0	1	0	1
1	1	51	2	159	54.5	-1.50	21.5	144	79	208	67	141.00	5.50	1	1.00	1	0	1	0	0	0	0	1	0	1	1
0	0	55	1	168	73.2	6.00	25.8	125	87	310	63	247.00	5.30	0	1.00	0	0	1	0	0	0	0	0	0	0	1
1	1	45	1	179	68.4	1.40	21.3	101	63	193	42	151.00	5.40	0	1.00	1	1	1	0	1	1	1	1	1	1	1
1	1	57	2	150	58.7	6.20	26.0	115	63	274	50	224.00	7.30	1	1.00	1	0	1	1	0	1	1	0	1	0	0
0	0	78	1	160	57.8	.90	22.5	129	62	209	57	152.00	6.80	1	1.00	1	0	1	1	1	1	1	1	1	1	0
1	1	71	2	154	63.8	2.00	26.9	118	70	179	67	112.00	5.60	1	1.00	1	0	1	1	1	0	0	0	0	1	1
1	1	66	2	145	46.7	-1.10	22.0	142	87	210	69	141.00	5.90	1	1.00	1	1	1	0	1	0	1	1	0	1	1
0	0	64	2	151	59.2	-.40	25.9	154	94	198	66	132.00	6.10	1	1.00	1	0	1	0	1	1	0	0	0	0	1
0	0	68	1	162	75.6	-1.30	28.8	107	64	185	51	134.00	5.40	1	1.00	1	0	1	0	0	1	0	0	0	1	1
1	1	45	2	156	41.8	.00	17.2	102	51	216	76	140.00	5.50	1	1.00	1	1	1	1	1	1	1	1	1	1	1
0	0	37	1	173	64.3	-.10	21.3	110	63	165	53	112.00	5.40	0	1.00	0	0	1	0	1	0	1	1	1	1	1
0	0	36	2	157	45.8	-1.60	18.5	90	50	195	62	133.00	4.80	1	1.00	1	1	1	1	1	1	1	1	1	1	1
1	1	63	1	174	63.4	-4.10	21.0	163	103	181	59	122.00	5.20	1	1.00	0	1	1	0	0	0	0	1	0	1	1
1	1	57	2	163	51.8	3.60	19.4	129	80	192	100	92.00	5.70	1	1.00	1	0	1	0	1	0	0	1	1	1	1
1	1	36	2	159	63.8	3.70	25.3	157	96	133	49	84.00	5.60	0	2.00	1	1	0	0	1	1	1	0	0	1	1
1	1	73	2	143	46.8	2.80	22.8	110	65	171	74	97.00	5.50	1	1.00	1	0	1	1	1	0	0	1	0	1	1
0	0	47	1	165	72.6	.60	26.5	105	76	156	48	108.00	5.20	0	1.00	0	1	1	0	0	1	1	0	1	1	1
1	1	34	2	151	58.8	1.90	25.6	90	47	194	92	102.00	5.00	1	1.00	1	0	1	1	1	1	1	0	1	1	1
1	1	74	2	150	49.2	4.10	21.7	120	64	202	50	152.00	5.30	1	1.00	1	0	1	1	1	0	1	1	0	1	1
1	1	57	2	157	58.3	.20	23.5	124	74	194	71	123.00	5.50	1	1.00	1	0	1	1	0	0	1	1	1	1	1
1	1	68	2	150	37.9	-.70	16.7	144	82	221	86	135.00	5.70	1	1.00	1	0	1	1	1	1	1	1	0	0	1
1	1	66	2	151	52.6	.30	23.1	98	55	257	64	193.00	5.70	1	1.00	1	1	1	1	1	1	1	1	1	0	1
1	1	67	2	151	50.8	1.90	22.1	137	77	216	105	111.00	6.00	1	1.00	0	0	1	1	1	1	1	1	1	1	1
1	1	48	2	159	66.4	-.80	26.1	122	79	169	53	116.00	5.20	1	1.00	1	1	1	0	1	0	0	0	0	1	1
0	0	64	2	151	52.4	.80	22.9	113	61	245	84	161.00	5.60	1	1.00	1	0	1	0	0	1	1	1	1	0	1
1	1	39	2	149	45.5	.50	20.5	105	64	175	67	108.00	5.60	1	1.00	1	0	1	1	1	0	0	1	1	1	1
0	0	45	1	170	59.3	1.10	20.5	100	58	173	73	100.00	5.60	0	1.00	1	0	0	0	1	1	1	1	1	1	1
1	1	68	1	159	55.6	2.80	22.0	129	81	209	50	159.00	5.60	1	1.00	0	1	1	0	0	0	0	1	1	1	1
1	1	54	2	156	56.7	-2.10	23.2	83	55	278	67	211.00	5.40	1	1.00	1	0	1	0	0	1	1	1	1	0	1
1	1	37	2	156	54.0	1.40	22.2	100	58	193	105	88.00	4.90	1	1.00	0	0	1	1	1	0	1	1	1	1	1
1	1	47	2	155	54.3	.60	22.6	112	73	209	76	133.00	5.00	1	1.00	1	0	1	1	1	1	1	1	0	1	1
1	1	64	1	166	67.1	-.70	24.2	134	78	192	63	129.00	5.40	1	1.00	1	0	1	0	1	1	0	1	1	1	1
1	1	70	2	151	53.7	1.40	23.6	129	79	169	47	122.00	5.50	1	1.00	0	0	1	1	1	1	1	1	0	0	1
1	1	62	1	165	65.2	1.60	24.0	130	81	258	61	197.00	5.60	1	1.00	1	1	1	1	1	1	1	1	1	0	1
1	1	56	2	149	51.3	3.70	23.1	116	81	274	56	218.00	6.00	1	1.00	1	0	1	0	1	0	1	1	1	0	1
0	0	60	2	159	53.8	5.80	21.2	129	81	226	65	161.00	5.50	1	1.00	1	0	1	0	1	0	1	1	1	0	1
1	1	71	1	162	76.5	-.10	29.0	146	76	185	24	161.00	6.10	0	1.00	1	1	1	1	1	1	1	0	0	0	1

1	1	73	1	168	81.9	.60	29.0	103	68	199	42	157.00	6.50	1	1.00	0	1	0	1	1	1	0	0	0	1	0
1	1	67	1	160	42.3	-1.70	16.5	107	72	153	54	99.00	5.50	1	1.00	1	0	1	1	0	1	1	1	1	1	1
1	1	69	1	156	61.5	-1.20	25.3	124	74	198	46	152.00	5.80	1	1.00	1	0	1	1	1	1	1	0	1	1	1
1	1	59	1	169	81.4	-.20	28.3	119	68	157	77	80.00	5.80	1	1.00	0	0	1	1	0	1	1	0	0	1	1
1	1	66	2	153	57.3	-.90	24.5	115	85	188	48	140.00	6.60	1	1.00	1	0	1	0	1	0	0	1	1	1	0
1	1	52	2	155	55.5	.00	23.1	108	65	178	74	104.00	5.30	1	1.00	1	0	1	1	1	1	1	1	1	1	1
1	1	61	2	145	46.6	.00	22.2	127	73	232	70	162.00	5.80	1	1.00	1	1	1	1	1	1	1	1	1	0	1
1	1	32	2	156	61.1	5.80	25.1	115	73	144	41	103.00	5.60	1	1.00	1	0	1	0	1	1	1	0	1	1	1
1	1	73	2	148	59.7	-1.20	27.3	156	83	175	49	126.00	7.10	1	1.00	1	1	1	1	1	1	1	0	0	1	0
0	0	30	2	150	52.1	5.30	23.3	100	67	171	63	108.00	5.50	1	1.00	1	0	1	1	1	0	1	1	1	1	1
1	1	55	1	171	57.8	.80	19.7	137	89	236	65	171.00	5.40	1	1.00	0	0	1	1	1	0	0	1	1	0	1
1	1	70	2	144	45.1	-1.90	21.8	131	73	197	65	132.00	5.50	1	1.00	1	0	1	1	1	1	1	1	0	1	1
1	1	47	2	156	50.1	1.90	20.5	97	63	196	78	118.00	5.30	1	1.00	0	0	1	0	1	0	0	1	1	1	1
0	1	53	2	157	50.8	.80	20.5	122	73	172	69	103.00	7.10	1	1.00	1	1	1	1	1	1	1	1	1	1	0
1	1	61	1	163	53.9	2.20	20.3	106	70	154	29	125.00	6.50	1	1.00	0	0	1	0	1	1	0	1	1	0	0
0	0	64	2	149	55.4	3.20	25.0	117	71	201	79	122.00	5.70	1	1.00	1	0	1	0	1	0	1	0	1	1	1
1	1	67	1	160	52.7	.20	20.6	147	76	202	78	124.00	6.80	1	1.00	0	1	1	1	1	1	1	1	0	1	0
1	1	67	2	153	63.4	.30	27.0	132	79	214	52	162.00	5.80	1	1.00	1	0	1	1	0	0	0	0	1	1	1
1	1	69	2	142	64.7	1.70	32.2	121	67	163	50	113.00	5.90	1	1.00	1	0	1	0	1	0	0	0	0	0	1
0	0	76	2	141	51.4	.00	25.7	143	72	163	49	114.00	5.90	1	1.00	1	1	1	1	1	1	1	0	0	1	1
1	1	72	2	144	68.6	-.70	32.9	141	82	197	38	159.00	5.60	1	1.00	1	0	1	1	1	1	1	0	0	0	1
1	1	75	2	147	57.2	-.20	26.3	170	99	259	56	203.00	5.40	1	1.00	1	0	1	1	1	0	0	0	0	0	1
1	1	65	1	157	66.1	-1.30	26.8	139	84	194	47	147.00	5.80	1	1.00	0	0	1	0	0	1	1	0	0	1	1
1	1	61	2	147	45.2	-.80	20.9	129	82	206	77	129.00	5.20	1	1.00	1	1	1	0	1	1	1	1	1	1	1
1	1	58	2	148	71.4	-2.70	32.5	130	73	229	39	190.00	6.70	1	1.00	1	1	1	1	0	0	0	0	0	0	0
1	1	77	2	139	37.4	.40	19.3	98	56	218	88	130.00	5.80	1	1.00	1	0	1	0	1	0	0	1	1	1	1
0	0	77	1	169	73.5	-2.10	25.6	147	90	160	41	119.00	6.50	1	1.00	1	1	1	0	0	1	0	0	0	1	0
1	1	60	1	163	75.3	1.60	28.4	135	90	186	63	123.00	5.50	1	1.00	1	1	1	0	1	1	1	0	0	1	1
0	0	75	2	153	56.0	5.00	23.8	122	69	203	70	133.00	5.40	1	1.00	1	0	1	0	0	0	0	1	1	0	1
1	1	61	2	144	53.6	2.00	26.0	129	79	205	52	153.00	5.20	1	1.00	1	0	1	0	1	1	1	0	0	1	1
0	0	74	2	142	45.7	-1.30	22.7	106	63	226	55	171.00	5.80	1	1.00	1	1	1	1	0	1	0	1	0	0	1
0	0	64	1	172	83.5	.90	28.1	133	77	301	54	247.00	6.00	0	1.00	1	1	1	1	1	1	1	0	1	0	1
1	1	43	2	156	52.1	1.40	21.4	110	68	192	84	108.00	5.50	0	1.00	1	1	1	0	1	0	1	1	1	1	1
0	0	54	2	151	41.3	1.00	18.0	104	62	214	65	149.00	5.60	1	1.00	0	0	1	1	1	0	0	1	1	1	1
1	1	41	2	158	72.3	-.40	28.9	147	101	185	56	129.00	5.30	1	1.00	0	0	1	1	1	0	1	0	0	1	1
0	1	79	1	156	55.9	.10	22.9	132	70	190	60	130.00	5.90	1	1.00	0	0	0	0	0	0	1	1	1	1	1
1	1	56	2	157	57.9	-.10	23.4	142	83	200	75	125.00	5.70	1	1.00	1	1	1	1	1	0	0	1	0	1	1
1	1	66	2	147	48.0	.20	22.2	118	75	220	80	140.00	5.40	1	1.00	1	1	1	1	1	1	1	1	1	0	1
1	1	52	2	150	61.7	2.80	27.3	140	75	183	57	126.00	6.50	1	1.00	1	1	1	0	1	0	1	0	0	1	0

1	1	63	1	164	62.7	1.60	23.4	103	65	224	57	167.00	5.60	1	1.00	1	1	1	0	1	1	1	1	1	0	1
0	0	69	1	169	67.5	-.80	23.6	147	74	167	68	99.00	5.90	0	1.00	0	1	1	1	1	1	1	1	0	1	1
0	0	38	2	160	53.5	3.60	20.8	114	72	172	86	86.00	5.10	1	1.00	0	1	1	0	0	0	0	1	1	1	1
1	1	64	2	153	49.1	-.60	21.0	139	86	232	101	131.00	5.60	1	1.00	1	1	1	0	0	0	0	1	1	0	1
0	0	69	2	140	45.5	2.10	23.0	144	83	224	52	172.00	5.70	1	1.00	1	1	1	0	1	1	1	1	0	0	1
1	1	52	2	155	48.0	.30	20.0	106	72	217	84	133.00	5.30	1	1.00	1	1	1	0	1	1	1	1	1	1	1
1	1	44	2	153	51.7	.50	21.9	121	68	187	56	131.00	5.40	1	1.00	1	0	1	0	0	0	1	1	1	1	1
1	1	71	1	166	70.5	.50	25.4	128	78	198	52	146.00	4.90	1	1.00	0	1	1	0	1	0	1	0	1	1	1
0	0	72	1	155	55.1	2.50	22.8	100	55	242	59	183.00	6.80	1	1.00	0	1	1	1	1	0	1	1	0	0	0
1	1	48	1	170	66.9	-1.90	23.2	136	81	249	56	193.00	5.50	0	1.00	0	1	1	0	1	1	1	1	1	0	1
1	1	79	2	144	50.5	.30	24.2	98	64	206	73	133.00	5.70	1	1.00	1	1	1	0	1	1	1	1	1	1	1
0	0	39	2	156	51.6	.30	21.3	119	71	160	68	92.00	4.80	1	1.00	1	0	1	0	0	0	1	1	0	0	1
1	1	69	2	153	48.6	.70	20.8	127	71	294	89	205.00	5.50	1	1.00	1	0	1	1	1	0	0	1	1	0	1
1	1	60	1	165	63.8	-3.30	23.4	139	85	200	110	90.00	5.50	0	1.00	0	1	1	1	1	1	1	1	1	1	1
1	1	76	1	157	53.2	-1.50	21.7	124	78	176	88	88.00	5.50	1	1.00	0	1	1	1	1	0	1	1	1	1	1
0	0	65	1	168	67.4	-.90	23.8	121	76	191	50	141.00	5.60	1	1.00	0	1	0	0	0	1	1	1	0	0	1
0	0	63	1	174	61.3	-.60	20.1	131	81	191	85	106.00	6.20	0	1.00	0	1	1	0	1	1	1	1	1	1	0
1	1	71	1	166	57.3	-.80	20.8	155	91	222	51	171.00	5.20	1	1.00	1	1	1	1	1	1	1	1	0	0	1
0	1	62	2	155	47.4	1.30	19.7	123	81	194	83	111.00	5.90	1	1.00	1	1	1	0	1	0	0	1	1	1	1
1	1	70	1	160	60.9	-.20	23.7	123	66	178	41	137.00	5.70	1	1.00	1	0	1	0	1	1	1	1	0	1	1
1	1	69	2	150	60.5	.50	27.0	131	81	205	44	161.00	5.70	1	1.00	1	0	1	1	1	1	1	0	1	1	1
1	1	74	2	152	65.6	2.00	28.2	130	79	163	41	122.00	6.20	1	1.00	0	1	1	1	1	1	0	0	1	1	1
0	0	53	1	166	68.5	4.90	24.7	128	86	237	54	183.00	5.80	1	1.00	0	0	1	1	1	1	0	1	1	0	1
1	1	55	1	170	66.1	-7.00	22.9	119	74	142	56	86.00	6.10	1	1.00	0	1	1	0	1	1	1	1	1	1	0
1	1	46	2	158	60.9	-1.60	24.4	111	61	160	54	106.00	5.10	1	1.00	0	1	1	1	1	1	1	1	1	1	1
1	1	76	1	159	75.1	.00	29.8	136	77	166	43	123.00	6.10	1	1.00	0	1	1	1	1	1	1	0	0	1	1
1	1	70	2	142	49.9	1.60	24.8	132	83	235	90	145.00	5.70	1	1.00	1	1	1	1	1	1	1	1	1	0	1
1	1	61	1	153	53.4	.60	22.6	154	101	237	37	200.00	5.30	1	1.00	1	1	1	0	0	0	0	1	0	0	1
1	1	75	2	141	43.3	-2.60	21.6	113	57	214	60	154.00	6.10	1	1.00	1	0	1	0	1	0	0	1	0	1	1
1	1	73	2	140	44.9	2.60	22.9	161	82	210	54	156.00	5.70	1	1.00	1	0	1	1	1	1	1	1	0	1	1
1	1	41	2	161	54.9	2.10	21.0	119	82	199	81	118.00	4.90	1	1.00	1	0	1	0	0	0	1	1	0	1	1
1	1	33	1	171	87.7	3.50	30.1	134	84	182	50	132.00	5.90	0	1.00	0	1	1	0	1	0	1	0	1	1	0
1	1	23	1	168	72.5	-.30	25.5	129	67	265	50	215.00	5.10	1	1.00	1	1	1	1	1	1	0	0	1	0	1
1	1	61	2	150	44.9	-.20	19.9	178	100	246	100	146.00	5.10	1	1.00	0	1	1	0	1	1	1	1	0	0	1
1	1	74	2	145	56.9	-.20	26.9	122	77	187	58	129.00	5.50	1	1.00	1	1	1	0	0	0	0	0	1	1	1
0	1	54	2	158	79.8	2.70	32.1	137	79	239	65	174.00	5.90	1	1.00	1	0	1	0	0	0	0	0	1	0	1
1	1	55	2	151	39.9	.70	17.4	105	70	191	96	95.00	5.70	1	1.00	0	0	1	0	1	0	1	1	1	1	1
1	1	48	2	152	60.7	1.30	26.2	127	84	234	67	167.00	5.80	1	1.00	1	1	1	0	0	0	1	0	1	0	1
1	1	68	1	167	65.3	-1.10	23.4	106	66	193	44	149.00	5.60	1	1.00	1	0	1	0	1	0	0	1	1	1	1

0	0	70	2	145	41.5	-3.90	19.7	103	63	227	52	175.00	5.90	1	1.00	1	1	1	1	1	1	1	1	0	0	1
1	1	42	2	153	52.4	-2.10	22.3	125	71	248	98	150.00	5.70	1	1.00	0	1	1	1	1	1	1	1	1	0	1
0	0	28	2	149	40.8	-1.50	18.4	93	56	161	71	90.00	4.90	1	1.00	0	1	0	0	1	0	0	1	1	1	1
1	1	62	2	146	38.6	-1.50	18.1	93	54	252	81	171.00	5.40	1	1.00	1	0	1	1	1	0	0	1	1	0	1
1	1	73	1	159	44.5	3.00	17.6	108	68	177	54	123.00	5.30	1	1.00	0	1	1	1	1	1	1	1	1	1	1
0	0	53	1	181	84.9	-.60	25.9	119	86	249	84	165.00	6.20	1	1.00	0	0	1	0	1	1	0	0	0	0	1
1	1	68	1	158	61.9	-1.70	24.9	141	85	271	88	183.00	5.50	1	1.00	0	0	1	1	1	1	1	1	0	0	1
1	1	32	2	153	48.8	.80	20.8	94	56	159	64	95.00	4.90	1	1.00	0	0	1	0	1	0	1	1	1	1	1
1	1	32	2	162	47.0	-1.20	18.0	110	63	172	82	90.00	5.10	1	1.00	1	1	1	0	1	1	1	1	1	1	1
1	1	56	2	161	53.4	.70	20.7	125	66	250	90	160.00	5.70	1	1.00	1	0	1	1	1	1	1	1	1	0	1
1	1	71	1	159	76.5	-1.70	30.1	133	77	128	36	92.00	5.50	1	1.00	1	0	1	0	1	0	1	0	0	0	1
1	1	74	1	153	65.9	1.50	28.3	127	67	191	56	135.00	5.40	1	1.00	1	1	1	0	1	0	0	0	1	1	1
1	1	66	1	169	67.4	1.30	23.6	141	99	226	53	173.00	5.60	1	1.00	0	1	1	1	1	1	1	1	0	0	1
0	0	53	1	168	79.2	-5.10	28.0	127	77	158	43	115.00	5.00	1	1.00	1	0	1	1	1	1	1	0	1	1	1
0	0	69	1	159	66.1	2.20	26.2	114	71	232	60	172.00	5.50	1	1.00	0	0	1	0	1	1	1	0	1	0	1
1	1	64	1	163	53.2	.70	20.0	118	73	183	104	79.00	5.40	1	1.00	0	1	1	1	1	1	1	1	1	1	1
1	1	67	2	154	58.8	1.30	24.7	164	99	222	81	141.00	5.30	1	1.00	1	0	1	0	1	0	0	1	0	0	1
0	0	67	1	155	64.8	1.00	26.9	120	77	205	42	163.00	5.40	0	1.00	0	1	1	1	1	1	1	0	0	1	1
0	0	50	2	163	55.9	1.40	20.9	112	70	195	53	142.00	5.40	1	1.00	1	0	1	0	0	1	1	1	1	1	1
0	0	40	2	161	45.6	.10	17.5	106	65	197	128	69.00	5.50	1	1.00	0	0	1	0	1	1	1	1	1	1	1
0	0	48	2	149	50.8	2.80	22.7	112	67	209	73	136.00	5.50	1	1.00	0	1	1	0	1	0	1	1	1	1	1
1	1	76	1	166	63.9	3.70	23.1	150	80	178	56	122.00	5.60	1	1.00	1	1	1	1	1	0	1	1	0	0	1
0	0	68	2	140	55.3	.60	28.2	96	55	214	58	156.00	5.60	1	1.00	1	1	1	1	1	1	1	0	0	1	1
1	1	43	2	154	62.1	.50	26.0	108	74	257	55	202.00	5.50	1	1.00	1	1	1	1	1	1	1	0	1	0	1
1	1	72	2	143	39.2	-1.40	19.1	98	63	238	81	157.00	5.70	1	1.00	1	1	1	0	1	0	1	1	1	0	1
1	1	64	1	159	71.9	-5.40	28.3	117	74	221	75	146.00	5.40	1	1.00	0	1	1	0	1	1	1	0	1	0	1
1	1	73	2	154	58.9	1.20	24.8	131	82	236	82	154.00	5.60	1	1.00	1	1	1	1	1	1	1	1	0	0	1
1	1	45	2	156	50.5	-1.50	20.6	103	61	198	78	120.00	5.30	1	1.00	1	0	1	0	1	1	1	1	1	1	1
0	1	72	2	155	74.6	-.40	30.9	160	85	210	50	160.00	6.00	1	1.00	1	0	1	0	1	0	0	0	0	1	1
0	0	23	2	163	51.0	1.30	19.2	109	64	294	71	223.00	5.00	0	1.00	0	0	0	1	1	1	0	1	1	0	1
0	1	42	1	167	52.8	.50	18.8	116	82	195	78	117.00	5.40	0	1.00	0	0	1	1	0	0	0	1	1	1	1
0	0	47	1	170	59.6	.80	20.6	130	86	156	53	103.00	5.80	0	1.00	0	1	1	0	1	1	0	1	1	1	1
1	1	31	2	164	51.7	.20	19.2	87	54	158	56	102.00	5.40	1	1.00	0	0	1	1	1	1	1	1	1	1	1
1	1	56	2	152	60.9	-.90	26.4	122	70	176	57	119.00	5.90	1	1.00	1	1	1	1	1	0	1	0	0	1	1
1	1	80	2	144	51.9	-4.50	24.9	96	57	158	41	117.00	5.70	0	1.00	1	0	1	1	1	0	1	1	0	1	1
1	1	59	2	155	54.1	2.30	22.4	120	74	186	56	130.00	5.30	1	1.00	0	0	1	1	1	0	0	1	1	1	1
0	1	73	2	145	52.4	1.30	24.8	119	68	205	53	152.00	5.70	1	1.00	1	0	1	0	1	0	1	1	0	1	1
1	1	57	2	158	49.8	-.50	19.9	158	95	201	89	112.00	5.10	1	1.00	1	0	1	0	1	1	1	1	0	1	1
1	1	68	2	155	42.3	-1.00	17.6	90	53	227	68	159.00	5.10	1	1.00	1	0	1	1	1	1	0	1	1	0	1

0	0	67	1	171	63.1	-1.40	21.6	140	88	170	40	130.00	5.70	1	1.00	0	0	1	1	1	1	1	1	0	1	1
1	1	73	1	168	67.5	-.50	23.8	126	82	265	34	231.00	5.90	1	1.00	1	0	1	1	1	1	1	1	1	0	1
1	1	63	2	147	45.4	-.10	20.9	144	93	232	58	174.00	5.60	1	1.00	1	0	1	1	1	0	0	1	0	0	1
1	1	67	1	161	62.9	-7.70	24.2	149	98	224	50	174.00	5.30	1	1.00	1	0	1	0	0	1	1	1	0	0	1
1	1	61	1	175	74.3	4.90	24.3	150	90	193	52	141.00	5.80	1	1.00	0	0	1	0	1	0	0	1	0	1	1
0	0	65	1	162	70.9	2.90	26.9	132	75	169	53	116.00	5.40	1	1.00	0	0	1	0	1	1	1	0	0	1	1
0	0	62	2	155	48.0	-.60	19.9	139	84	193	48	145.00	5.70	1	1.00	0	1	1	0	0	0	0	1	1	1	1
1	1	45	2	158	46.7	-.60	18.7	99	63	183	65	118.00	5.80	1	1.00	1	0	1	0	1	0	1	1	1	1	1
1	1	64	2	147	65.5	-4.80	30.1	127	75	216	63	153.00	5.70	1	1.00	1	0	1	1	1	1	1	0	1	1	1
1	1	75	2	143	44.8	-1.30	21.8	162	78	227	45	182.00	5.10	1	1.00	1	0	1	1	1	1	1	1	0	0	1
1	1	69	1	156	59.2	2.50	24.4	125	70	170	40	130.00	6.20	1	1.00	1	0	1	1	1	0	0	1	0	1	1
1	1	60	1	164	57.9	-4.80	21.4	134	91	168	36	132.00	5.70	0	1.00	0	0	1	0	1	1	1	1	0	0	1
1	1	80	2	139	39.6	-.20	20.4	142	79	237	85	152.00	5.70	1	1.00	1	0	1	1	1	0	0	1	0	0	1
1	1	50	1	169	67.4	5.40	23.6	119	78	203	44	159.00	5.70	1	1.00	1	1	1	0	1	0	0	1	1	1	1
1	1	60	1	172	73.4	2.20	24.7	106	69	135	29	106.00	5.90	1	1.00	1	0	1	1	1	1	0	1	1	0	1
1	0	56	2	152	59.2	-2.40	25.7	126	78	220	39	181.00	6.60	1	1.00	0	0	1	0	1	0	0	0	1	0	0
1	1	65	1	159	69.5	1.50	27.4	111	58	191	58	133.00	5.50	1	1.00	0	0	1	0	1	1	1	0	0	1	1
1	1	67	2	148	41.7	2.70	18.9	119	68	234	53	181.00	5.80	1	1.00	1	0	1	0	1	0	1	1	1	0	1
1	1	65	2	146	49.6	.30	23.2	144	81	202	60	142.00	5.70	1	1.00	0	0	1	1	1	1	1	1	0	1	1
1	1	46	2	161	59.6	3.00	22.8	98	60	165	71	94.00	5.20	1	1.00	1	0	1	1	1	1	1	1	1	1	1
1	1	33	2	159	52.2	3.70	20.5	117	68	178	47	131.00	5.50	1	1.00	1	0	1	0	0	1	1	1	1	1	1
1	1	78	1	163	64.6	1.60	24.4	105	56	203	42	161.00	6.50	1	1.00	0	0	1	1	1	0	1	1	1	1	0
1	1	68	2	155	56.9	2.70	23.6	128	81	234	54	180.00	5.90	1	1.00	1	0	1	0	1	0	0	1	0	0	1
1	1	57	1	167	76.3	4.10	27.4	147	82	175	42	133.00	4.90	1	1.00	0	0	1	1	1	0	1	0	0	1	1
1	1	68	2	150	45.6	-2.40	20.2	119	78	159	44	115.00	5.50	1	1.00	1	0	1	1	1	1	0	1	1	0	1
1	1	51	1	172	94.9	-.30	32.1	111	72	177	42	135.00	5.30	1	1.00	0	0	1	0	1	0	1	0	1	0	1
1	1	72	1	164	64.5	3.50	24.1	122	73	144	52	92.00	5.70	1	1.00	0	0	1	1	1	0	1	1	0	1	1
1	1	44	2	155	41.6	3.30	17.3	128	82	185	72	113.00	5.50	1	1.00	1	1	1	1	1	1	1	1	1	1	1
1	1	41	1	166	83.2	3.50	30.1	110	73	269	38	231.00	5.70	1	1.00	1	0	1	1	1	0	1	0	1	0	1
1	1	42	2	149	45.1	.30	20.2	96	53	166	72	94.00	5.50	1	1.00	1	0	1	0	1	0	0	1	1	1	1
1	1	35	2	157	47.9	1.10	19.4	104	58	184	73	111.00	5.70	1	1.00	1	0	1	0	1	1	1	1	1	1	1
1	1	38	2	154	51.8	.60	21.7	101	67	154	85	69.00	4.90	1	1.00	0	0	1	1	1	1	1	1	1	1	1
1	1	72	2	149	53.5	.80	24.1	141	78	195	68	127.00	5.40	1	1.00	1	1	1	1	1	1	1	1	0	1	1
1	1	59	2	156	56.5	2.40	23.3	106	66	226	78	148.00	5.50	1	1.00	1	0	1	0	1	0	0	1	1	0	1
1	1	82	1	159	47.1	-1.00	18.6	141	72	184	29	155.00	5.70	0	1.00	1	0	1	1	1	1	1	1	0	0	1
1	1	66	1	166	77.0	-2.60	28.1	136	80	265	64	201.00	6.20	1	1.00	1	0	1	1	1	1	1	0	1	0	1
1	1	48	1	166	72.8	3.40	26.2	115	72	190	29	161.00	5.50	0	1.00	1	1	1	1	1	1	1	0	1	0	1
1	1	51	1	172	63.3	-5.00	21.4	99	63	212	54	158.00	5.80	0	1.00	1	0	1	0	0	0	0	1	1	1	0
1	1	37	2	151	48.8	-2.20	21.3	86	52	174	72	102.00	5.60	1	1.00	1	0	1	0	1	1	1	1	1	1	1

1	1	69	2	155	58.1	-1.60	24.1	105	63	225	69	156.00	6.70	1	1.00	1	1	1	0	1	1	1	1	1	0	0
1	1	73	1	162	77.1	3.20	29.3	107	58	174	46	128.00	5.40	1	1.00	1	0	1	0	1	1	1	0	0	1	1
1	1	81	1	169	60.0	1.30	21.0	116	70	178	62	116.00	5.50	1	1.00	1	0	1	1	1	0	1	1	0	1	1
0	0	44	2	155	46.8	.70	19.4	135	78	175	80	95.00	5.10	1	1.00	0	0	1	0	0	0	0	1	1	1	1
1	1	45	1	172	58.0	-.40	19.7	99	64	336	63	273.00	5.40	1	1.00	1	1	1	0	1	1	0	1	1	0	1
1	1	66	1	167	72.0	-2.00	25.9	104	66	152	42	110.00	6.60	0	1.00	0	1	1	1	1	0	1	0	1	1	0
1	1	26	2	156	38.6	5.90	15.9	94	55	189	72	117.00	5.10	1	1.00	1	0	0	0	0	0	1	1	1	1	1
0	0	33	2	163	63.7	7.20	24.0	100	58	147	75	72.00	5.40	1	1.00	1	0	1	0	1	0	1	1	1	1	1
0	0	42	1	176	75.6	-.60	24.2	118	80	204	34	170.00	5.40	0	1.00	0	0	0	0	0	1	1	1	1	0	1
1	1	34	1	179	72.9	1.40	22.8	110	67	218	59	159.00	5.30	1	1.00	0	0	1	1	0	1	1	1	1	1	1
1	1	45	1	178	78.0	-4.80	24.6	144	86	157	47	110.00	5.20	1	1.00	1	0	1	0	1	0	1	1	0	1	1
1	1	62	2	152	68.3	-.60	29.7	140	82	232	41	191.00	5.70	1	1.00	1	0	1	1	0	0	1	0	0	0	1
0	0	56	2	154	63.4	-2.80	26.6	130	77	224	57	167.00	6.30	1	1.00	0	0	1	0	0	0	0	0	1	0	1
0	0	54	1	160	55.7	-1.50	21.8	140	88	177	81	96.00	5.40	0	1.00	0	0	0	1	1	0	0	1	0	1	1
0	0	78	1	157	61.9	-.50	25.2	129	63	184	34	150.00	5.60	0	1.00	1	0	1	0	1	0	1	0	0	0	1
1	1	53	1	160	84.5	-2.00	33.0	140	92	195	44	151.00	5.60	1	1.00	1	0	1	0	1	0	1	0	0	1	1
0	0	54	2	154	52.2	2.30	21.9	131	73	178	69	109.00	7.10	1	1.00	1	1	1	0	1	1	1	1	0	0	0
1	1	42	2	157	57.0	1.00	23.0	119	82	217	51	166.00	6.00	0	1.00	1	0	1	0	1	1	0	1	1	1	1
1	1	55	2	155	74.6	-.20	31.0	165	101	229	50	179.00	5.80	1	1.00	1	0	1	0	0	0	0	0	0	0	1
1	1	62	1	170	74.6	4.50	25.9	121	71	206	62	144.00	7.40	1	1.00	1	1	1	0	1	1	1	0	1	1	0
1	1	76	1	162	55.0	-.30	20.9	105	55	256	36	220.00	5.80	0	1.00	0	0	1	1	1	1	1	1	1	0	1
0	0	73	1	161	55.5	1.50	21.3	116	67	225	35	190.00	6.00	1	1.00	0	1	1	1	0	1	1	1	1	0	1
1	1	43	2	149	50.4	-1.70	22.7	118	69	222	49	173.00	5.40	1	1.00	1	0	1	1	0	0	0	1	1	0	1
1	1	77	2	149	49.8	1.00	22.3	98	50	212	92	120.00	5.50	1	1.00	1	0	1	0	1	1	1	1	0	1	1
0	0	39	2	155	72.3	-1.90	30.0	90	53	189	44	145.00	5.90	1	1.00	1	0	1	0	0	1	0	0	1	1	1
1	1	84	1	153	48.6	.30	20.7	165	89	122	69	53.00	4.80	1	1.00	1	0	1	1	1	0	1	1	0	1	1
1	1	64	1	161	75.4	.30	29.0	107	67	182	44	138.00	6.00	1	1.00	0	1	1	0	1	0	0	0	1	1	1
0	0	71	1	165	72.3	-1.00	26.4	112	61	229	51	178.00	7.50	1	1.00	0	0	1	1	1	1	1	0	1	0	0
0	0	57	2	153	36.9	1.80	15.7	99	72	164	52	112.00	5.20	1	1.00	1	0	1	0	0	0	0	1	1	1	1
0	0	72	2	150	40.8	.70	18.2	139	82	232	67	165.00	5.90	1	1.00	1	0	1	0	0	1	0	1	1	0	1
1	1	81	2	150	51.7	-1.30	22.8	126	81	250	51	199.00	5.40	1	1.00	1	1	1	1	1	0	0	1	1	0	1
1	1	23	2	167	56.4	-.50	20.2	86	53	118	45	73.00	5.10	1	1.00	1	1	1	0	1	0	0	1	1	1	1
1	1	32	2	156	43.1	.50	17.7	91	58	199	75	124.00	5.40	1	1.00	1	1	1	0	1	1	1	1	1	1	1
1	1	39	1	173	65.6	1.80	21.8	112	69	205	78	127.00	5.60	0	1.00	0	1	1	0	1	1	1	1	1	1	1
1	1	71	2	148	52.1	.40	23.6	139	82	221	88	133.00	5.20	1	1.00	1	0	1	1	1	1	0	1	1	0	1
1	1	82	2	143	38.0	-1.40	18.5	129	76	214	79	135.00	5.90	1	1.00	1	0	1	0	1	0	0	1	1	1	1
1	1	74	2	149	55.5	-.30	24.9	138	86	205	61	144.00	5.90	1	1.00	1	1	1	1	1	0	0	1	0	1	1
1	1	74	1	162	65.9	.40	25.1	136	85	188	34	154.00	5.50	1	1.00	0	0	1	1	1	1	1	0	0	0	1
1	1	71	1	153	60.2	-4.90	25.7	123	58	213	62	151.00	5.30	1	1.00	1	0	1	0	0	1	1	0	0	1	1

1	1	74	1	170	72.8	2.20	25.1	137	83	192	59	133.00	6.00	1	1.00	0	0	1	1	1	0	0	0	1	1	1
1	1	53	2	154	55.4	2.50	23.3	144	85	217	81	136.00	5.90	1	1.00	1	0	1	0	1	0	0	1	0	0	1
1	1	61	2	155	48.8	-1.60	20.3	122	79	221	62	159.00	5.70	1	1.00	1	0	1	0	1	1	1	1	1	0	1
1	1	78	1	162	58.8	.00	22.5	144	83	164	65	99.00	6.70	0	1.00	0	0	1	1	1	1	1	1	0	1	0
1	1	64	2	140	42.2	-.30	21.6	117	76	222	51	171.00	5.50	1	1.00	1	0	1	1	1	0	1	1	1	0	1
1	1	74	1	160	47.4	1.10	18.5	114	76	132	45	87.00	5.30	1	1.00	0	0	1	1	1	1	1	1	1	1	1
1	1	69	2	144	52.2	.90	25.1	119	66	214	72	142.00	5.50	1	1.00	1	0	1	1	1	1	1	0	0	1	1
1	1	61	1	162	59.3	-2.20	22.7	118	80	173	78	95.00	6.00	0	1.00	0	1	1	0	0	1	1	1	1	1	1
0	0	73	2	147	45.0	4.10	20.8	111	72	275	76	199.00	5.70	1	1.00	1	0	1	1	1	0	1	1	1	0	1
1	1	64	1	162	74.9	2.30	28.7	124	84	178	62	116.00	5.50	1	1.00	0	1	1	1	1	1	1	0	0	1	1
0	0	48	2	153	36.0	1.20	15.3	117	81	235	128	107.00	5.90	1	1.00	1	1	1	0	0	0	0	1	1	0	1
0	0	60	1	159	63.4	7.10	24.9	162	91	227	74	153.00	5.80	0	1.00	0	0	1	0	1	1	0	1	0	0	1
1	1	26	2	156	43.1	.70	17.7	109	62	200	76	124.00	5.20	1	1.00	1	0	1	0	1	0	1	1	1	1	1
1	1	63	2	152	50.4	.10	21.7	143	88	194	74	120.00	5.70	1	1.00	1	0	1	1	1	1	1	1	0	1	1
1	1	78	1	165	48.3	-1.10	17.6	103	56	164	67	97.00	6.10	0	1.00	0	0	1	1	1	1	1	1	0	1	1
0	0	60	1	157	55.2	.70	22.5	97	61	180	78	102.00	6.20	1	1.00	0	0	1	0	1	1	1	1	1	1	1
1	1	58	2	158	51.2	.80	20.5	126	68	224	82	142.00	5.70	1	1.00	1	1	1	0	0	1	1	1	1	0	1
1	1	65	2	155	60.5	1.60	25.3	126	77	247	70	177.00	5.70	1	1.00	1	0	1	1	1	1	0	0	1	0	1
1	1	71	1	156	63.9	-1.90	26.3	126	73	206	51	155.00	5.80	1	1.00	1	0	1	1	1	1	1	0	1	1	1
1	1	72	1	154	58.1	-2.50	24.6	117	75	212	68	144.00	5.40	1	1.00	1	0	0	1	1	1	0	1	1	1	1
1	1	73	1	147	52.8	-1.30	24.3	120	66	218	51	167.00	5.80	1	1.00	0	1	1	1	1	1	0	1	0	1	1
1	1	58	2	160	55.1	-1.20	21.4	111	65	198	77	121.00	5.30	1	1.00	1	0	1	0	0	0	1	1	1	1	1
1	1	72	2	152	38.5	-.70	16.5	134	68	206	62	144.00	5.90	1	1.00	1	0	1	0	1	1	0	1	1	1	1
1	1	57	1	165	59.8	2.70	21.8	116	76	235	41	194.00	5.80	0	1.00	1	0	0	1	1	1	1	1	1	0	1
1	1	69	2	148	51.2	.80	23.3	156	81	292	51	241.00	12.20	1	1.00	1	1	1	1	0	1	1	1	0	0	0
1	1	67	2	146	50.8	.00	23.7	146	82	201	49	152.00	5.80	1	1.00	1	0	1	1	1	0	1	1	0	1	1
1	1	85	1	161	52.7	.00	20.3	146	80	131	41	90.00	5.90	1	1.00	1	1	1	1	1	1	1	1	0	1	1
1	1	70	1	160	66.8	1.10	26.2	130	85	205	78	127.00	6.40	1	1.00	0	1	1	1	1	1	1	0	0	1	1
1	1	62	1	162	69.3	-4.00	26.2	141	84	212	50	162.00	5.90	1	1.00	1	0	1	0	1	0	1	0	0	1	1
0	0	46	2	158	68.8	1.70	27.7	115	70	185	54	131.00	5.30	1	1.00	1	0	1	1	1	0	0	0	1	1	1
1	1	76	2	146	46.4	1.20	21.7	127	60	171	76	95.00	5.70	1	1.00	1	1	1	1	1	0	0	1	1	1	1
1	1	43	1	167	80.2	.60	28.8	137	75	214	45	169.00	5.80	0	1.00	1	1	1	0	0	1	1	0	1	1	1
1	1	63	2	149	69.7	2.10	31.6	105	73	243	59	184.00	5.70	1	1.00	1	0	1	1	1	0	0	0	1	0	1
1	1	40	2	154	47.0	-.40	19.9	112	66	212	81	131.00	5.40	1	1.00	1	1	1	0	1	1	1	1	1	1	1
1	1	70	2	145	49.2	-.10	23.4	140	71	211	43	168.00	5.60	1	1.00	1	1	1	1	1	1	1	1	0	1	1
1	1	68	1	162	56.0	1.30	21.2	127	75	149	56	93.00	5.90	1	1.00	1	0	1	1	1	1	1	1	1	1	1
1	1	71	2	159	49.1	.40	19.4	95	59	252	101	151.00	6.20	1	1.00	0	1	1	1	1	1	1	1	1	0	1
1	1	86	2	136	34.6	-.90	18.6	147	73	183	70	113.00	7.30	1	1.00	1	0	1	1	1	1	1	1	0	1	0
1	1	51	1	168	68.8	-1.20	24.4	118	73	170	49	121.00	5.60	0	1.00	1	1	1	1	1	1	1	1	1	1	1

1	1	36	2	156	55.8	-1.60	22.8	100	65	178	96	82.00	5.50	1	1.00	1	1	1	0	1	1	1	1	1	1	1
1	1	73	2	158	53.1	-1.60	21.2	110	61	233	46	187.00	5.20	1	1.00	1	1	1	1	1	1	0	1	0	0	1
1	1	65	2	149	43.5	.60	19.5	123	62	205	41	164.00	5.80	1	1.00	1	1	1	1	0	0	0	1	1	1	1
1	1	57	2	155	52.1	1.90	21.7	115	81	218	81	137.00	5.90	1	1.00	1	0	1	1	1	1	1	1	0	1	1
1	1	67	1	160	68.2	-1.70	26.7	134	92	233	47	186.00	5.40	1	1.00	0	1	1	0	0	1	1	0	0	0	1
1	1	65	2	143	56.8	-7.90	27.9	131	75	191	52	139.00	6.40	1	1.00	1	1	1	0	0	0	0	0	1	1	1
0	0	71	2	142	43.8	.10	21.6	146	92	247	46	201.00	8.50	1	1.00	0	0	1	0	0	1	0	1	0	0	0
1	1	60	1	171	72.8	.10	24.8	142	94	213	50	163.00	5.60	1	1.00	0	0	1	1	1	1	1	1	0	1	1
0	0	75	1	157	53.6	-1.00	21.6	122	67	196	60	136.00	5.20	0	1.00	0	1	1	0	1	0	1	1	0	1	1
1	1	64	2	146	60.5	5.50	28.3	139	83	183	37	146.00	6.00	1	1.00	0	0	1	0	1	0	0	0	0	0	1
1	1	24	2	160	60.4	2.90	23.6	107	65	183	45	138.00	5.50	1	1.00	0	0	0	0	1	0	0	1	1	1	1
1	1	71	2	149	60.4	1.50	27.1	140	87	249	38	211.00	6.30	1	1.00	1	0	1	0	1	0	1	0	0	0	1
1	1	73	1	162	56.1	.60	21.4	163	93	175	51	124.00	5.80	1	1.00	1	1	1	0	0	1	1	1	0	1	1
0	0	68	2	147	40.4	-3.50	18.7	100	56	217	68	149.00	5.30	1	1.00	1	0	1	0	1	0	1	1	1	1	1
0	0	75	2	136	43.1	-.10	23.2	98	60	222	51	171.00	5.60	1	1.00	1	1	1	1	1	0	1	1	0	0	1
1	1	64	1	164	62.9	-.90	23.5	121	76	217	64	153.00	5.30	1	1.00	0	1	1	1	1	1	1	1	1	1	1
0	1	69	2	148	52.3	.60	24.0	115	64	221	75	146.00	5.80	1	1.00	1	1	1	0	0	1	0	1	1	0	1
1	1	70	1	157	49.5	-.90	20.0	123	72	179	54	125.00	5.70	1	1.00	1	1	1	1	1	1	1	1	1	1	1
1	1	65	1	159	64.2	-3.10	25.3	146	78	238	78	160.00	5.40	0	1.00	0	1	1	1	1	1	1	0	0	0	1
1	1	62	2	147	62.1	-2.10	28.5	126	72	209	44	165.00	5.80	1	1.00	1	0	1	1	1	1	1	0	0	1	1
1	1	79	2	141	43.5	-3.30	21.8	118	63	172	54	118.00	5.90	1	1.00	1	1	1	1	1	1	1	1	1	1	1
1	1	59	2	142	38.6	-1.30	19.1	142	85	201	62	139.00	5.30	1	1.00	1	0	1	1	1	1	1	1	0	1	1
1	1	61	1	170	72.2	.80	24.9	134	87	184	57	127.00	7.10	1	1.00	0	0	1	0	1	1	1	1	1	1	0
1	1	59	2	147	48.8	-.70	22.5	123	71	210	44	166.00	5.70	1	1.00	1	1	1	0	0	1	1	1	0	1	1
1	1	58	2	156	53.3	.10	21.9	104	65	292	57	235.00	5.70	1	1.00	1	1	1	1	1	1	1	1	1	0	1
1	1	69	1	160	59.8	2.50	23.4	125	87	183	40	143.00	5.60	0	1.00	0	1	1	0	0	0	1	1	0	1	1
0	0	43	1	162	63.0	6.70	24.0	111	66	252	53	199.00	5.60	0	1.00	0	1	0	1	1	1	1	1	1	0	1
0	0	75	1	161	54.1	2.10	20.9	133	74	184	76	108.00	5.20	1	1.00	0	1	1	0	1	1	1	1	0	1	1
1	1	66	2	154	61.1	1.70	25.7	135	82	209	48	161.00	6.10	1	1.00	1	1	1	0	1	0	0	0	1	1	1
1	1	64	1	165	65.5	-1.30	24.1	124	82	256	47	209.00	5.70	1	1.00	0	0	1	1	1	1	1	1	1	0	1
1	1	40	2	150	44.4	-.40	19.8	112	65	172	66	106.00	5.60	1	1.00	1	1	1	1	1	1	1	1	1	1	1
1	1	48	2	156	38.0	1.40	15.7	125	84	197	125	72.00	5.20	1	1.00	0	1	1	0	0	0	0	1	1	1	1
1	1	71	1	163	71.3	.20	26.8	138	81	300	98	202.00	6.10	0	1.00	0	1	0	1	1	1	1	0	1	0	1
1	1	77	1	164	56.4	1.10	21.0	161	87	186	83	103.00	5.30	1	1.00	0	1	1	1	1	1	1	1	0	1	1
0	0	50	1	172	65.4	.60	22.2	122	77	253	43	210.00	5.60	1	1.00	0	0	1	0	0	0	1	1	1	0	1
0	0	75	2	141	45.1	.90	22.6	136	76	192	61	131.00	5.50	1	1.00	1	0	1	1	1	0	1	1	1	1	1
0	0	67	2	158	46.0	1.50	18.3	130	74	275	100	175.00	5.80	1	1.00	1	1	1	1	1	1	0	1	1	0	1
1	1	75	2	151	53.2	-.20	23.2	117	63	165	64	101.00	5.40	1	1.00	1	1	1	0	1	0	1	1	1	1	1
1	1	55	2	153	53.7	1.20	22.9	101	63	272	53	219.00	5.10	1	1.00	1	1	1	0	1	1	1	1	1	0	1

1	1	59	1	173	73.3	.80	24.4	126	82	185	65	120.00	5.40	0	1.00	0	1	1	0	1	0	1	1	1	1	1
0	0	32	2	161	48.2	.80	18.5	115	61	161	78	83.00	5.00	0	1.00	1	1	1	1	0	1	1	1	1	1	1
1	1	80	1	153	52.2	-.10	22.2	150	70	148	49	99.00	5.30	1	1.00	0	1	1	1	1	1	1	1	0	1	1
1	1	30	1	176	60.8	4.60	19.5	116	64	180	62	118.00	5.10	0	1.00	0	1	1	1	1	1	1	1	1	1	1
0	0	51	1	161	90.8	-2.30	35.2	128	85	214	56	158.00	5.40	1	1.00	0	0	1	0	1	0	0	0	1	1	1
0	0	84	2	142	42.1	-.10	20.9	97	60	243	73	170.00	5.50	1	1.00	1	0	1	1	1	0	1	1	1	0	1
0	0	67	2	147	48.0	-.30	22.2	134	65	287	60	227.00	5.30	1	1.00	1	0	1	0	0	1	0	1	0	0	1
0	0	70	1	161	60.4	-.50	23.3	130	67	187	54	133.00	5.60	1	1.00	0	0	1	1	1	1	1	1	0	1	1
0	0	68	1	156	52.3	-2.00	21.6	139	76	109	51	58.00	5.80	0	1.00	0	0	1	1	1	0	0	1	1	1	0
0	0	33	2	159	55.6	1.70	22.1	86	54	130	62	68.00	5.10	1	1.00	1	0	1	1	1	1	1	1	1	1	1
1	1	79	1	153	63.8	-2.20	27.2	94	55	202	54	148.00	5.30	1	1.00	0	1	1	1	1	1	1	0	0	1	1
0	0	31	2	156	86.4	2.30	35.3	104	57	169	47	122.00	5.70	0	1.00	1	0	0	1	1	0	0	0	1	1	1
1	1	58	2	150	46.7	-.60	20.7	99	59	199	59	140.00	5.50	1	1.00	1	1	1	0	0	1	0	1	1	1	1
0	0	67	2	153	55.1	1.60	23.6	108	59	184	51	133.00	5.10	1	1.00	1	0	1	1	1	1	0	1	1	1	1
0	0	64	1	157	51.6	-.60	20.9	102	62	179	68	111.00	5.70	0	1.00	0	1	1	0	0	1	1	1	1	1	1
0	1	50	2	164	62.9	2.20	23.3	114	85	198	76	122.00	5.30	1	1.00	1	0	0	1	0	0	0	1	0	1	1
1	1	62	1	168	63.1	-1.00	22.4	160	90	191	63	128.00	5.20	1	1.00	0	1	1	1	1	1	0	1	0	1	1
1	1	62	2	153	48.0	.60	20.5	124	72	232	74	158.00	5.60	1	1.00	1	1	1	0	1	1	0	1	1	0	1
1	1	57	1	169	61.4	1.90	21.4	137	92	166	85	81.00	5.40	1	1.00	0	1	1	0	1	1	1	1	0	1	1
1	1	53	2	152	83.5	-2.00	36.0	126	71	203	72	131.00	9.20	1	1.00	1	1	1	0	1	0	0	0	1	1	0
1	1	71	1	156	54.3	-.70	22.3	136	76	149	77	72.00	5.50	1	1.00	0	0	1	1	1	1	1	1	1	1	1
1	1	73	2	155	52.0	-.20	21.7	119	72	166	71	95.00	5.10	1	1.00	1	1	1	1	1	1	1	1	1	1	1
0	0	59	1	172	56.1	8.40	18.8	108	74	160	67	93.00	6.80	0	2.00	1	0	1	1	1	0	0	1	1	1	0
0	0	51	2	157	50.5	1.10	20.5	151	86	259	73	186.00	5.90	0	1.00	1	1	1	1	1	1	1	1	0	0	1
0	0	76	2	147	48.6	-1.00	22.6	116	69	188	72	116.00	5.40	1	1.00	1	0	1	0	1	0	0	1	0	1	1
1	1	68	2	143	48.2	-1.90	23.5	119	65	206	72	134.00	5.50	1	1.00	1	1	1	1	1	0	1	1	1	1	1
0	0	37	1	172	87.8	2.40	29.6	130	77	234	43	191.00	5.50	0	1.00	1	1	1	0	1	0	1	0	1	0	1
1	1	29	2	158	50.9	-4.00	20.4	109	66	176	76	100.00	5.20	1	1.00	1	0	0	1	1	1	1	1	1	1	1
0	0	39	1	167	90.2	-2.20	32.5	151	91	219	39	180.00	5.60	0	1.00	0	0	0	0	1	0	0	0	0	0	1
1	1	71	1	164	67.6	-2.00	25.0	130	69	184	77	107.00	5.20	1	1.00	0	1	1	1	1	1	1	0	0	1	1
1	1	60	1	160	58.8	5.80	23.0	121	89	185	46	139.00	5.90	0	1.00	1	1	1	0	1	0	1	1	0	1	0
1	1	33	2	159	58.6	.10	23.1	104	69	252	90	162.00	5.10	1	1.00	1	0	1	1	1	0	1	1	1	0	1
1	1	68	1	156	63.1	2.00	26.0	169	89	190	47	143.00	4.90	1	1.00	1	0	0	0	1	1	0	0	0	1	1
0	0	68	1	163	65.2	.70	24.6	106	58	205	105	100.00	5.40	0	1.00	0	1	1	1	1	1	1	1	1	1	1
1	1	63	1	157	76.7	-2.50	30.9	134	86	216	44	172.00	5.60	1	1.00	0	0	1	0	1	0	0	0	0	1	1
0	0	75	1	155	60.8	-3.20	25.1	130	74	215	69	146.00	5.50	1	1.00	0	0	1	0	0	0	1	0	0	1	1
1	1	64	2	155	61.8	-1.70	25.7	101	64	184	65	119.00	5.60	1	1.00	1	0	1	1	1	0	1	0	1	0	1
0	0	75	1	157	51.9	.80	20.9	158	84	175	67	108.00	6.50	1	1.00	0	0	1	1	1	1	1	1	0	1	0
1	1	61	1	161	61.3	-.70	23.5	142	96	210	79	131.00	6.30	1	1.00	0	1	1	0	1	0	0	1	0	1	1

1	1	46	2	154	68.1	-.10	28.7	107	65	160	48	112.00	5.40	0	1.00	1	1	1	0	0	0	1	0	1	1	1
0	0	40	1	163	63.0	2.60	23.6	123	79	225	59	166.00	6.10	1	1.00	0	0	1	1	1	0	1	1	1	0	1
0	1	72	1	155	63.5	-2.00	26.2	121	73	178	57	121.00	6.00	1	1.00	1	0	1	0	0	0	0	0	1	1	1
1	1	62	1	166	66.0	-1.00	23.9	125	78	190	52	138.00	5.40	1	1.00	0	1	1	1	1	1	0	1	0	1	1
0	0	60	2	159	53.3	-1.00	21.0	127	71	195	53	142.00	5.70	1	1.00	1	0	1	1	1	1	0	1	0	0	1
1	1	87	2	142	54.0	-1.10	26.7	178	96	254	55	199.00	6.10	1	1.00	1	0	0	1	1	0	0	0	0	0	1
1	1	63	2	157	56.2	-3.90	22.7	121	69	261	43	218.00	5.70	1	1.00	0	0	1	0	1	0	0	1	1	0	1
0	0	59	1	174	85.7	-3.20	28.4	112	72	218	52	166.00	5.70	0	1.00	0	0	1	0	1	0	1	0	0	1	1
1	1	55	2	155	59.6	4.80	24.9	129	82	246	106	140.00	5.30	1	1.00	1	1	1	1	1	1	1	1	0	0	1
1	1	53	2	152	46.2	-3.60	20.0	114	76	156	56	100.00	5.10	0	1.00	0	0	0	0	1	0	1	1	0	1	1
1	1	57	2	158	56.1	.40	22.5	110	70	215	51	164.00	5.80	1	1.00	1	1	1	0	1	0	0	1	1	1	1
1	1	71	2	143	46.4	-.90	22.6	114	72	235	71	164.00	5.60	1	1.00	1	1	1	0	1	1	1	1	0	0	1
1	1	56	1	159	59.6	-1.80	23.4	132	82	338	42	296.00	5.70	0	1.00	0	0	1	1	0	1	0	1	1	0	1
1	1	64	2	147	74.1	2.50	34.4	136	78	199	51	148.00	7.70	1	1.00	1	0	1	1	1	1	1	0	0	0	0
1	1	60	2	152	58.5	-.80	25.2	98	60	181	53	128.00	5.60	1	1.00	1	1	1	0	1	1	1	0	1	0	1
0	0	31	2	165	51.8	1.00	19.0	90	58	165	70	95.00	5.40	0	2.00	0	1	1	1	1	1	1	1	1	1	1
0	0	45	2	148	56.0	3.30	25.4	104	62	242	62	180.00	5.50	1	1.00	1	1	1	0	0	0	0	0	1	0	1
1	1	30	2	159	57.3	.00	22.7	99	66	219	92	127.00	5.30	1	1.00	0	0	1	1	1	1	1	1	1	1	1
1	1	55	1	167	77.9	-2.00	27.7	154	82	197	45	152.00	5.80	0	1.00	0	0	0	1	1	1	1	0	0	1	1
1	1	59	2	143	53.5	-.60	26.0	145	83	243	91	152.00	5.20	1	1.00	0	0	0	0	1	1	0	0	0	0	1
1	1	55	2	147	57.9	3.70	26.7	145	99	237	88	149.00	5.60	1	1.00	0	1	1	0	0	0	1	0	0	0	1
1	1	50	1	172	62.0	-2.60	21.0	120	76	256	75	181.00	5.20	0	1.00	0	1	1	1	1	1	1	1	1	0	1
1	1	47	2	154	63.7	4.50	26.7	113	65	250	98	152.00	5.20	1	1.00	0	0	1	0	1	0	0	0	1	0	1
1	1	56	2	162	61.4	.30	23.2	127	68	288	58	230.00	5.80	1	1.00	0	0	1	0	1	1	0	1	1	0	1
1	1	68	2	147	38.5	-1.70	17.9	116	68	231	87	144.00	5.50	1	1.00	1	0	1	0	1	1	1	1	0	0	1
1	1	57	2	149	59.9	.40	26.8	123	85	303	54	249.00	5.90	0	1.00	1	0	1	0	0	0	0	0	1	0	1
0	0	72	2	152	59.2	.10	25.4	112	81	203	40	163.00	5.60	1	1.00	1	0	1	0	1	1	0	0	0	1	1
1	1	44	2	159	55.0	-1.00	21.6	111	78	174	71	103.00	5.10	0	1.00	0	0	0	0	1	0	1	1	1	1	1
0	0	77	1	159	64.3	2.50	25.4	128	67	163	75	88.00	6.00	1	1.00	0	0	1	1	1	1	1	0	1	1	0
0	0	68	2	159	69.1	1.90	27.3	118	65	219	65	154.00	6.10	1	1.00	1	0	1	0	0	0	0	0	0	1	1
1	1	75	2	142	49.3	-2.00	24.3	140	70	216	51	165.00	5.80	1	1.00	1	0	1	0	1	0	1	1	0	1	0
1	1	61	2	163	54.5	5.30	20.6	129	84	221	70	151.00	6.10	0	1.00	1	0	1	0	0	0	1	1	1	0	1
1	1	63	2	136	44.9	2.40	24.2	138	85	261	63	198.00	5.70	1	1.00	1	0	1	0	1	0	0	1	1	0	1
1	1	69	2	150	54.7	-.50	24.2	117	66	185	49	136.00	5.20	1	1.00	1	0	1	1	1	1	1	1	1	1	1
0	0	66	1	155	51.7	-.40	21.6	127	79	199	55	144.00	6.30	1	1.00	1	0	1	1	0	1	0	1	1	1	0
1	1	62	1	155	58.1	1.60	24.0	142	95	164	53	111.00	6.00	1	1.00	0	0	1	0	1	0	0	1	0	1	1
0	0	68	2	158	50.2	1.70	20.2	101	61	240	57	183.00	5.50	1	1.00	1	0	1	0	1	0	1	1	1	0	1
0	0	83	2	138	42.2	1.20	22.3	118	67	206	67	139.00	5.60	1	1.00	1	1	1	0	1	1	1	1	1	0	1
1	1	51	2	156	51.5	-2.00	21.0	105	63	200	75	125.00	5.60	1	1.00	1	1	1	1	1	1	1	1	1	1	1

1	1	67	2	144	48.4	1.50	23.2	113	65	233	119	114.00	5.90	1	1.00	1	0	1	1	1	1	0	1	1	0	1
0	0	50	1	180	74.4	-.20	22.9	103	63	183	38	145.00	5.60	1	1.00	0	0	1	0	1	0	0	1	1	0	1
1	1	57	1	164	51.8	-1.50	19.1	113	78	151	79	72.00	5.40	1	1.00	0	0	1	1	0	0	0	1	1	1	1
1	1	46	1	175	68.5	-4.50	22.3	135	71	242	118	124.00	5.50	1	1.00	0	1	1	0	1	1	1	1	1	0	1
1	1	64	2	154	51.8	.50	21.7	99	61	223	70	153.00	5.40	1	1.00	1	0	1	0	1	1	1	1	1	0	1
1	1	43	2	162	59.1	3.80	22.6	127	87	207	62	145.00	5.40	1	1.00	0	1	1	0	0	1	1	1	0	0	1
1	1	73	2	143	38.1	-.30	18.6	121	74	208	97	111.00	5.60	1	1.00	1	0	1	1	1	0	0	1	1	0	1
1	1	73	1	171	83.7	6.40	28.5	155	78	132	37	95.00	5.10	1	1.00	1	0	1	0	1	0	1	0	0	0	1
1	0	60	1	159	47.8	2.90	18.9	135	83	134	44	90.00	5.30	0	1.00	0	0	1	0	1	0	1	1	1	1	1
0	0	49	1	171	88.7	-.50	30.4	116	76	197	57	140.00	5.10	1	1.00	0	0	0	1	1	1	1	0	1	1	1
1	1	43	1	166	90.1	6.20	32.8	148	93	255	64	191.00	5.60	1	1.00	0	0	0	1	0	1	0	0	0	0	1
0	0	65	1	162	57.9	-1.40	22.0	118	69	174	42	132.00	5.30	1	1.00	0	0	1	1	1	1	1	1	1	1	1
1	1	57	2	145	46.6	1.20	22.1	132	82	222	84	138.00	5.40	1	1.00	0	0	1	0	1	0	0	1	1	0	1
1	1	60	2	157	67.1	.90	27.3	127	85	218	69	149.00	5.40	1	1.00	1	0	1	1	0	1	1	0	1	1	1
1	1	63	1	169	79.0	-3.00	27.5	139	91	158	55	103.00	4.90	1	1.00	0	1	1	1	1	1	1	0	0	1	1
1	1	43	2	162	54.9	.80	20.8	97	64	205	78	127.00	5.30	1	1.00	0	1	1	0	0	0	0	1	1	1	1
1	1	76	1	166	69.5	.20	25.3	132	74	178	55	123.00	6.00	1	1.00	0	0	1	1	1	1	1	0	0	1	1
1	1	63	2	151	56.5	.30	24.7	128	82	201	89	112.00	5.20	1	1.00	0	0	1	0	1	0	1	1	0	1	1
1	1	62	2	147	54.7	.40	25.3	84	55	195	72	123.00	5.60	1	1.00	1	1	1	0	1	0	0	0	1	1	1
1	1	62	1	169	73.4	1.50	25.6	131	88	224	81	143.00	5.50	1	1.00	0	0	1	1	1	1	1	0	0	0	1
1	1	61	2	153	45.4	2.40	19.2	116	74	196	62	134.00	5.80	0	1.00	0	0	1	0	1	0	1	1	1	1	1
1	1	71	2	145	51.6	.70	24.6	130	87	264	70	194.00	5.90	1	1.00	1	0	1	1	1	1	1	1	1	0	1
1	1	66	2	149	48.4	2.40	21.9	110	78	211	81	130.00	5.70	1	1.00	1	0	1	1	1	1	0	1	1	1	1
1	1	62	1	157	58.6	-2.30	23.6	138	89	179	45	134.00	5.50	1	1.00	0	1	1	0	1	1	1	1	0	1	1
1	1	64	1	162	60.6	2.50	23.0	101	58	163	64	99.00	5.60	0	1.00	1	0	1	0	1	0	0	1	1	1	1
1	1	74	1	162	58.1	-1.20	22.2	119	76	216	57	159.00	6.10	1	1.00	0	0	1	0	1	0	0	1	1	0	1
1	1	29	2	163	56.2	.60	21.2	93	58	190	72	118.00	5.20	1	1.00	0	0	0	0	1	0	0	1	1	1	1
0	0	70	1	164	50.2	4.90	18.6	141	88	197	59	138.00	6.20	1	1.00	1	0	1	0	1	0	0	1	0	1	1
1	1	62	2	147	54.7	-2.40	25.2	143	91	290	118	172.00	5.60	1	1.00	1	1	1	1	1	0	1	0	0	0	1
1	1	73	1	162	57.6	2.20	21.8	142	62	182	47	135.00	5.40	1	1.00	1	0	1	1	1	1	0	1	0	1	1
1	1	57	1	161	68.1	1.40	26.2	125	82	285	44	241.00	5.60	1	1.00	0	0	1	0	1	1	1	0	1	0	1
1	1	64	2	154	52.8	1.00	22.4	120	65	223	82	141.00	5.70	1	1.00	1	0	1	0	1	0	0	1	1	0	1
1	1	25	1	171	64.5	-1.70	21.9	143	82	164	50	114.00	5.70	1	1.00	1	1	1	0	1	1	1	1	0	1	1
0	0	56	1	173	120.1	-.40	40.0	131	90	184	63	121.00	6.20	1	1.00	0	0	1	0	1	1	1	0	0	1	1
0	0	67	1	168	61.3	2.20	21.8	137	78	173	56	117.00	5.20	1	1.00	0	1	1	1	1	0	0	1	1	1	1
1	1	73	1	159	72.0	3.50	28.3	123	68	186	45	141.00	5.80	1	1.00	1	0	1	1	1	1	1	0	0	1	1
1	1	51	2	152	57.3	1.30	24.8	145	82	226	73	153.00	5.70	1	1.00	1	1	1	1	1	0	1	1	0	0	1
0	0	60	2	142	41.3	-.50	20.4	140	85	166	48	118.00	6.00	1	1.00	1	0	1	0	1	0	0	1	0	1	1
1	1	51	2	161	56.0	3.70	21.5	99	59	263	109	154.00	5.90	1	1.00	1	0	1	0	1	0	1	1	1	0	1

0	0	57	2	158	60.4	8.90	24.2	111	71	195	79	116.00	5.60	1	1.00	1	0	1	1	1	1	0	1	0	0	1
1	1	85	1	152	48.5	2.50	21.1	103	53	202	46	156.00	5.80	1	1.00	1	0	1	1	1	1	0	1	1	1	1
1	1	62	2	146	44.1	-.30	20.8	139	91	173	77	96.00	5.50	1	1.00	0	1	1	1	1	1	1	1	0	1	1
0	0	72	1	167	73.8	1.30	26.4	116	65	230	45	185.00	5.40	1	1.00	1	0	1	1	1	1	0	0	0	0	1
1	1	67	1	161	74.9	.20	28.7	143	103	188	63	125.00	5.30	1	1.00	1	1	1	1	1	1	1	0	0	1	1
0	0	52	1	164	71.4	1.00	26.5	106	69	250	51	199.00	5.40	0	1.00	0	0	1	0	1	1	0	0	0	0	1
0	0	69	2	151	42.0	1.10	18.5	125	70	227	74	153.00	5.50	1	1.00	1	1	1	1	0	0	0	1	0	0	1
1	1	75	2	154	50.2	1.00	21.1	81	50	141	53	88.00	5.50	1	1.00	1	0	1	1	1	1	0	1	1	1	1
1	1	51	2	153	54.2	3.10	23.2	120	70	225	79	146.00	5.50	1	1.00	1	0	1	1	1	1	1	1	1	0	1
1	1	63	1	168	71.0	.70	25.2	120	83	207	55	152.00	6.00	1	1.00	1	0	1	1	1	1	1	0	1	1	1
1	1	66	2	152	54.4	-.20	23.5	140	83	223	62	161.00	5.70	1	1.00	1	0	1	1	1	0	0	1	0	0	1
1	1	74	2	148	56.9	-.30	25.9	138	79	234	83	151.00	5.90	1	1.00	1	1	1	1	1	1	1	0	0	0	1
1	1	76	1	175	66.1	-1.40	21.4	133	76	189	51	138.00	6.20	1	1.00	0	0	1	1	1	1	1	1	1	1	0
1	1	65	2	150	60.7	1.50	26.8	111	65	238	44	194.00	5.90	1	1.00	1	0	1	1	0	1	1	0	1	0	1
1	1	58	1	166	66.4	1.20	24.2	128	75	210	56	154.00	6.00	1	1.00	1	0	1	0	1	1	1	1	1	1	1
1	1	80	2	148	40.9	-.10	18.6	112	63	222	93	129.00	5.70	1	1.00	1	0	1	1	1	0	0	1	0	0	1
1	1	66	2	143	42.2	-.40	20.6	131	75	239	76	163.00	5.60	1	1.00	1	1	1	0	0	0	0	1	1	0	1
1	1	69	2	148	50.5	1.20	22.9	107	67	210	88	122.00	5.20	1	1.00	1	0	1	0	1	0	1	1	0	1	1
1	1	63	2	158	60.7	-2.10	24.4	117	64	203	48	155.00	6.50	1	1.00	1	1	1	0	1	0	1	1	1	1	0
1	1	83	2	150	44.9	3.60	20.0	134	62	180	69	111.00	5.60	1	1.00	1	1	0	1	1	1	1	1	1	1	1
0	0	52	2	151	49.8	1.00	21.8	112	76	208	64	144.00	6.20	1	1.00	1	1	1	1	1	1	1	1	1	1	1
0	0	48	2	149	49.8	1.70	22.3	117	67	216	82	134.00	5.40	1	1.00	1	1	1	0	1	0	1	1	1	1	1
0	0	80	2	142	44.4	2.30	22.0	134	64	247	89	158.00	6.00	1	1.00	1	0	1	1	1	1	0	1	0	0	1
1	1	61	2	151	47.2	-.70	20.6	106	58	201	85	116.00	5.80	1	1.00	1	1	1	1	0	1	0	1	1	1	1
1	1	62	2	147	46.5	-.90	21.6	103	57	224	61	163.00	5.50	1	1.00	0	0	1	0	0	0	1	1	1	0	1
1	1	43	2	160	49.6	-1.20	19.4	106	63	202	64	138.00	5.20	1	1.00	1	0	1	0	1	0	1	1	1	1	1
1	1	69	2	153	63.1	.00	27.0	122	75	186	79	107.00	5.80	1	1.00	0	1	1	1	1	1	0	0	1	1	1
1	1	57	2	153	58.4	-2.80	24.8	126	76	263	58	205.00	5.20	1	1.00	1	0	1	1	1	1	1	1	1	0	1
0	0	63	1	168	82.0	-8.30	29.1	121	75	178	40	138.00	7.60	1	1.00	1	0	1	0	0	0	1	0	0	1	0
1	1	53	2	164	58.9	3.30	22.0	132	84	281	131	150.00	5.70	1	1.00	1	0	1	0	0	1	0	1	1	0	1
1	1	39	2	165	51.5	1.10	19.0	129	74	156	83	73.00	5.20	0	1.00	1	1	1	0	1	0	1	1	1	1	1
0	0	80	1	163	62.1	-2.80	23.2	117	72	186	35	151.00	5.60	0	1.00	1	1	1	1	1	1	1	1	1	0	1
1	1	68	2	145	47.0	.20	22.2	131	79	176	65	111.00	5.60	1	1.00	1	0	1	0	1	0	0	1	1	0	1
1	1	47	2	156	47.2	.20	19.4	100	65	202	134	68.00	5.00	1	1.00	0	1	0	1	1	1	1	1	1	1	1
1	1	68	2	150	56.6	1.90	25.2	102	58	189	55	134.00	5.70	1	1.00	0	1	1	1	1	1	1	0	1	1	1
1	1	68	2	146	53.8	-1.30	25.1	118	73	215	46	169.00	5.90	1	1.00	1	0	1	1	1	1	1	0	1	1	1
1	1	48	2	161	64.3	1.80	24.8	118	83	164	59	105.00	5.60	1	1.00	1	0	1	0	1	1	1	1	1	1	1
1	1	67	1	166	69.4	.40	25.1	141	91	185	44	141.00	5.00	1	1.00	0	1	1	0	1	1	1	0	0	1	1
1	1	69	2	150	54.9	.00	24.5	121	67	196	66	130.00	5.70	1	1.00	1	0	1	1	1	0	1	1	0	1	1

1	1	67	2	142	45.7	.80	22.8	124	71	243	83	160.00	5.20	1	1.00	0	0	1	1	1	1	1	1	0	0	1
1	1	69	1	157	58.8	.40	23.9	130	80	201	55	146.00	5.50	1	1.00	0	0	1	0	1	0	1	1	0	1	1
1	1	69	2	152	59.6	.90	25.7	150	78	214	67	147.00	5.50	1	1.00	1	1	1	1	1	1	1	0	0	1	1
1	1	71	1	158	60.1	4.00	23.9	156	82	232	106	126.00	5.40	1	1.00	0	1	1	1	1	1	1	1	0	0	1
0	0	55	1	167	76.6	-1.40	27.4	123	86	221	37	184.00	5.60	1	1.00	1	0	1	0	0	0	0	0	0	0	1
1	1	62	2	156	41.5	-2.20	17.1	101	56	207	49	158.00	6.20	1	1.00	1	0	1	0	0	1	1	1	0	1	1
1	1	78	1	161	56.3	-1.00	21.8	122	79	193	71	122.00	5.70	1	1.00	1	1	1	0	1	1	1	1	0	1	1
1	1	63	2	150	58.3	1.40	25.9	139	90	159	52	107.00	5.80	1	1.00	1	0	1	1	1	1	1	0	0	0	1
1	1	68	2	152	52.7	-.60	22.7	128	82	259	62	197.00	5.50	1	1.00	1	1	1	1	1	1	1	1	1	0	1
1	1	73	2	151	62.9	.60	27.6	159	94	295	59	236.00	6.00	1	1.00	1	0	1	1	1	1	1	0	0	0	1
0	1	39	2	149	47.7	-1.50	21.4	130	84	249	55	194.00	5.30	1	1.00	0	0	1	0	0	0	1	1	1	0	1
0	1	57	1	169	70.3	-.30	24.4	121	68	178	49	129.00	5.90	0	1.00	1	0	1	1	1	1	1	1	1	1	1
1	1	57	2	144	52.3	1.30	25.2	102	61	247	50	197.00	5.60	1	1.00	0	0	1	0	0	1	1	0	1	0	1
1	1	54	1	157	63.6	-3.00	25.8	151	81	190	51	139.00	6.40	1	1.00	0	1	1	1	1	1	0	0	0	1	1
0	1	74	2	149	58.1	3.30	26.3	121	63	193	63	130.00	5.50	1	1.00	1	1	1	0	1	0	0	0	0	1	1
1	1	70	2	141	65.1	-1.70	32.6	124	73	196	52	144.00	6.20	1	1.00	1	1	1	1	1	0	1	0	0	1	1
1	1	61	2	151	57.8	-.10	25.2	127	79	226	51	175.00	5.30	1	1.00	1	0	1	0	0	0	0	0	0	0	1
1	1	87	2	144	57.8	-1.70	27.9	118	67	228	71	157.00	5.80	1	1.00	1	0	1	1	1	0	1	0	0	0	1
0	0	80	1	158	48.7	.30	19.5	113	66	224	73	151.00	5.30	1	1.00	1	0	1	1	1	0	1	1	0	0	1
0	0	42	1	168	63.7	5.40	22.4	132	79	210	57	153.00	5.60	0	1.00	0	0	0	0	1	0	1	1	1	1	1
1	1	81	1	160	45.0	.10	17.4	106	68	188	76	112.00	5.90	1	1.00	1	0	1	0	1	0	1	1	1	1	1
1	1	63	2	153	47.2	3.30	20.0	141	94	222	83	139.00	5.70	1	1.00	1	1	1	1	1	1	1	1	0	0	1
0	0	61	1	167	68.0	-3.20	24.3	135	73	235	78	157.00	5.30	1	1.00	0	0	0	0	1	0	0	1	1	0	1
1	1	69	2	144	52.0	.50	24.9	109	63	200	44	156.00	5.90	1	1.00	0	1	1	1	1	1	0	1	0	1	1
0	0	71	2	150	45.8	.20	20.4	126	69	167	60	107.00	5.80	1	1.00	1	0	1	0	1	0	0	1	0	0	1
0	0	48	2	153	54.9	-1.30	23.4	138	88	208	50	158.00	5.20	1	1.00	1	0	1	1	1	1	1	1	1	1	1
0	0	67	2	142	49.5	1.30	24.6	131	80	258	63	195.00	5.30	1	1.00	1	0	1	0	0	0	0	1	1	0	1
0	0	74	1	163	57.6	-.80	21.5	139	78	192	63	129.00	6.30	1	1.00	0	0	0	0	0	1	1	1	1	1	0
0	0	56	2	157	63.5	3.70	25.6	136	80	222	65	157.00	5.60	1	1.00	1	0	1	0	0	0	0	0	0	0	1
1	1	73	1	168	71.0	-1.30	25.1	114	68	185	58	127.00	6.40	1	1.00	0	1	1	0	1	1	0	0	0	1	1
1	1	40	2	167	53.8	-4.30	19.3	95	53	137	71	66.00	5.60	1	1.00	0	1	1	1	1	1	1	1	1	1	1
1	1	68	1	158	59.1	-10.20	23.7	140	97	204	51	153.00	5.60	1	1.00	0	0	1	1	1	1	1	1	0	1	1
1	1	63	2	147	53.4	.90	24.7	120	71	227	84	143.00	5.80	1	1.00	0	0	1	0	1	0	0	1	1	0	1
1	1	60	2	145	44.8	2.70	21.4	151	74	251	79	172.00	5.50	1	1.00	1	1	1	0	1	1	1	1	0	0	1
1	1	74	2	143	47.2	3.80	23.0	106	62	214	60	154.00	5.40	1	1.00	1	0	1	1	1	0	0	1	0	1	1
1	1	39	2	154	42.6	-1.20	17.8	104	61	222	104	118.00	5.50	0	1.00	1	0	1	1	1	1	1	1	1	0	1
1	1	60	2	154	52.2	-.10	22.0	113	79	189	80	109.00	6.10	1	1.00	1	1	1	1	1	1	1	1	1	1	1
1	1	67	2	147	42.7	1.00	19.6	109	63	248	124	124.00	5.20	1	1.00	1	1	1	1	0	1	0	1	1	0	1
0	0	71	2	154	56.3	-.50	23.8	128	76	177	57	120.00	5.10	1	1.00	1	1	1	1	1	0	1	1	1	1	1

0	0	73	2	149	61.8	4.60	27.7	131	75	207	61	146.00	5.90	1	1.00	0	0	1	0	1	0	0	0	1	1	1
1	1	73	2	151	51.8	-.20	22.8	154	84	220	63	157.00	5.50	1	1.00	1	1	1	1	1	1	1	1	0	0	1
0	0	55	1	162	57.1	1.70	21.7	127	82	256	91	165.00	5.50	0	2.00	0	1	1	1	0	0	0	1	1	0	1
1	1	59	2	155	47.3	3.40	19.7	104	69	290	76	214.00	5.10	1	1.00	0	1	1	0	0	0	1	1	1	0	1
1	1	74	1	168	80.7	-3.40	28.4	106	59	194	77	117.00	5.80	0	1.00	0	0	1	0	1	1	1	0	0	1	1
1	1	69	2	154	61.9	-.20	26.0	114	71	162	67	95.00	5.40	1	1.00	1	1	1	1	1	1	1	0	0	1	1
0	0	69	2	141	56.8	2.20	28.6	133	78	198	62	136.00	5.50	1	1.00	1	0	1	1	1	1	1	0	0	0	1
0	0	40	2	140	50.4	-1.40	25.8	98	58	138	60	78.00	4.10	1	1.00	1	0	1	0	0	1	0	0	1	1	1
0	1	79	1	159	57.3	-.10	22.8	100	62	182	75	107.00	5.30	1	1.00	0	0	1	1	1	0	1	1	0	1	1
1	1	52	2	158	60.4	3.30	24.2	143	89	183	60	123.00	5.30	1	1.00	1	0	1	1	1	0	0	1	0	1	1
1	1	62	2	148	45.1	3.70	20.6	160	85	177	79	98.00	5.60	1	1.00	1	1	1	1	1	0	1	1	0	1	1
0	0	80	2	136	52.5	.10	28.3	160	86	160	57	103.00	5.90	1	1.00	1	1	1	0	1	0	1	0	0	1	1
0	0	37	2	161	65.6	4.50	25.4	102	62	162	63	99.00	5.30	1	1.00	1	0	1	0	0	0	0	0	1	1	1
0	0	63	2	145	69.4	-4.00	33.0	139	78	240	61	179.00	5.90	1	1.00	1	0	1	1	1	1	1	0	0	0	1
1	1	74	2	153	58.7	-1.30	25.0	135	76	169	46	123.00	7.30	1	1.00	1	1	1	0	1	0	0	0	0	1	0
1	1	79	1	163	63.7	-.20	23.8	115	60	224	63	161.00	7.40	1	1.00	1	0	1	1	1	1	1	1	0	0	0
1	1	79	2	141	37.5	-.30	18.8	113	60	201	77	124.00	5.00	1	1.00	1	1	1	1	1	1	1	1	0	0	1
0	0	47	2	155	47.4	-.70	19.7	92	56	179	65	114.00	5.10	1	1.00	0	1	0	1	1	1	1	1	1	1	1
0	0	57	1	165	57.7	-.80	21.1	123	72	200	40	160.00	7.60	0	1.00	1	1	1	0	1	0	0	1	0	1	0
0	0	62	1	164	70.2	4.10	26.2	151	83	217	46	171.00	6.30	0	1.00	1	0	1	0	1	1	1	0	0	1	1
1	1	52	1	154	59.5	.50	25.2	146	90	195	95	100.00	5.20	0	1.00	0	1	1	1	1	1	1	0	0	1	1
0	0	45	2	153	60.3	.90	25.6	99	65	246	92	154.00	5.10	1	1.00	1	1	1	0	0	0	0	0	1	0	1
1	1	75	1	163	47.2	2.30	17.8	123	76	208	70	138.00	5.60	1	1.00	1	1	1	1	0	0	1	1	1	1	1
1	1	65	1	170	66.6	-1.60	23.0	146	98	218	53	165.00	6.00	1	1.00	1	0	1	1	1	1	1	1	0	1	0
1	1	70	2	157	65.2	-.10	26.4	113	65	210	51	159.00	5.90	1	1.00	1	0	1	1	1	0	0	0	0	1	1
1	1	45	2	151	50.8	.00	22.3	150	88	191	48	143.00	5.10	1	1.00	0	1	1	1	1	1	1	1	0	1	1
0	0	36	2	161	54.4	1.80	20.9	81	45	183	64	119.00	5.60	1	1.00	1	1	1	1	1	1	0	1	1	1	1
1	1	27	1	168	78.2	4.50	27.8	106	55	167	61	106.00	5.20	0	1.00	0	0	1	1	1	0	0	0	1	1	1
0	0	56	1	166	69.6	-1.40	25.3	138	83	237	51	186.00	5.30	1	1.00	0	0	1	0	0	1	1	0	1	0	1
1	1	60	2	157	46.4	-1.90	18.9	99	54	227	57	170.00	5.70	1	1.00	1	0	1	1	1	1	1	1	1	0	1
1	1	61	1	162	61.3	-1.90	23.4	115	70	183	64	119.00	5.40	1	1.00	0	1	1	1	1	1	1	1	1	1	1
1	1	64	1	156	51.9	.70	21.4	125	71	220	86	134.00	5.50	1	1.00	0	0	1	0	0	1	1	1	0	0	1
1	1	75	1	157	55.1	1.30	22.2	123	70	177	54	123.00	5.40	0	1.00	0	0	1	1	1	0	0	1	1	1	1
1	0	33	1	171	78.3	-2.50	26.8	144	78	225	65	160.00	4.90	1	1.00	0	0	1	0	0	1	0	0	0	0	1
1	1	56	2	146	59.5	-.50	27.7	102	63	251	54	197.00	6.30	1	1.00	1	0	1	1	1	1	0	0	1	0	1
1	1	72	1	166	60.3	-1.60	21.9	147	78	185	90	95.00	6.90	0	1.00	0	1	1	1	1	1	1	1	0	1	0
0	0	74	1	152	61.7	2.70	26.7	129	85	193	56	137.00	5.40	1	1.00	0	0	1	1	0	1	1	0	0	1	1
0	0	68	2	154	44.9	3.80	18.9	156	84	194	68	126.00	5.10	1	1.00	1	1	1	1	0	1	1	1	0	1	1
1	1	70	2	142	47.5	-2.40	23.6	137	73	225	101	124.00	5.80	1	1.00	1	1	1	1	1	1	1	1	0	0	1

1	1	32	1	173	63.4	-4.20	21.1	114	71	197	60	137.00	5.30	0	1.00	0	0	1	0	0	0	1	1	1	1	1
1	1	45	2	160	58.4	-3.40	22.7	124	66	203	51	152.00	5.40	1	1.00	0	1	1	0	1	1	1	1	1	1	1
0	0	41	2	161	55.1	1.60	21.2	99	54	183	47	136.00	5.30	0	1.00	1	0	0	0	1	0	1	1	1	1	1
0	0	72	2	146	43.2	3.10	20.1	142	78	249	77	172.00	5.90	1	1.00	1	1	1	0	1	1	1	1	0	0	1
0	0	73	2	150	42.4	.10	18.8	84	56	182	43	139.00	5.90	1	1.00	1	0	1	0	1	0	0	1	0	1	1
1	1	57	2	155	62.8	6.50	26.2	99	63	218	55	163.00	5.40	1	1.00	0	1	1	0	1	0	1	0	1	1	1
1	1	33	2	162	53.9	3.70	20.5	111	60	148	92	56.00	4.60	1	1.00	1	1	1	1	1	1	1	1	1	1	1
1	1	65	1	167	65.4	-.80	23.5	102	68	214	50	164.00	5.30	0	1.00	1	0	1	1	1	1	1	1	1	1	1
0	0	70	1	158	62.9	.40	25.1	127	80	144	51	93.00	7.10	1	1.00	1	0	1	1	1	1	1	0	1	1	0
0	0	54	2	153	67.0	1.80	28.7	123	74	215	45	170.00	5.10	1	1.00	0	0	1	1	0	1	0	0	1	1	1
0	0	32	2	158	56.7	-1.70	22.6	110	67	202	53	149.00	4.90	1	1.00	1	1	1	0	1	1	0	1	1	1	1
1	1	70	2	150	56.5	2.00	25.2	111	63	186	87	99.00	5.80	1	1.00	1	0	1	1	1	1	0	0	1	0	1
1	1	72	1	153	59.3	-.60	25.4	140	90	219	79	140.00	5.40	0	1.00	0	1	1	0	1	1	1	0	0	1	1
0	0	48	1	160	59.2	-.40	23.2	114	64	258	47	211.00	6.20	0	1.00	0	1	0	1	1	1	1	1	1	0	1
0	0	34	1	181	78.8	-.40	23.9	137	83	268	66	202.00	5.70	0	1.00	0	1	1	0	1	1	1	1	1	0	1
0	0	62	1	163	48.4	-3.30	18.2	98	62	194	75	119.00	5.40	0	1.00	1	0	1	1	1	1	0	1	1	1	1
1	1	64	1	170	62.5	-1.90	21.5	154	86	172	62	110.00	5.20	0	1.00	0	1	1	0	1	0	1	1	0	1	1
0	0	64	1	164	66.8	2.50	24.7	121	72	217	56	161.00	6.60	1	1.00	0	0	1	0	0	1	0	1	0	1	0
0	0	62	1	160	61.6	-.20	24.2	129	72	213	72	141.00	7.00	1	1.00	0	0	0	1	1	1	1	1	1	1	0
1	1	81	2	137	48.8	-1.30	25.8	129	70	282	76	206.00	5.90	1	1.00	1	0	1	1	1	1	1	0	1	0	1
0	0	68	2	147	49.5	.40	22.8	113	62	218	78	140.00	5.40	1	1.00	1	0	1	1	1	1	1	1	0	1	1
1	1	67	2	151	57.1	5.10	25.1	113	66	194	66	128.00	5.50	1	1.00	1	1	1	1	0	1	1	0	1	1	1
0	0	67	1	164	51.9	1.80	19.2	93	59	232	83	149.00	5.60	1	1.00	0	1	1	0	1	1	1	1	1	0	1
0	0	43	2	158	78.2	-5.10	31.2	109	72	203	43	160.00	6.20	1	1.00	0	0	1	0	1	0	0	0	1	1	1
1	1	73	2	147	51.8	1.90	24.0	120	68	230	80	150.00	5.60	1	1.00	1	0	1	0	1	0	0	1	0	0	1
1	1	59	1	162	77.8	-1.20	29.5	159	94	210	68	142.00	5.60	0	1.00	0	1	1	0	1	1	1	0	0	1	1
1	1	45	2	155	64.3	3.00	26.9	110	64	164	53	111.00	6.60	1	1.00	1	0	1	0	1	1	1	0	1	1	0
1	1	77	2	148	48.5	.40	22.0	144	79	224	69	155.00	5.40	1	1.00	1	0	1	0	1	0	1	1	0	0	1
1	1	76	2	152	58.6	1.70	25.2	125	69	265	108	157.00	5.40	1	1.00	1	0	1	1	1	1	1	0	1	0	1
1	1	72	2	152	49.3	2.70	21.3	130	69	224	86	138.00	5.30	1	1.00	1	0	1	1	1	1	1	1	1	0	1
1	1	72	2	158	48.3	.50	19.3	126	77	205	118	87.00	5.60	1	1.00	1	0	1	1	1	1	1	1	0	1	1
1	1	57	1	173	73.7	2.30	24.5	119	72	213	47	166.00	6.20	1	1.00	0	0	1	0	1	0	1	1	1	1	0
1	1	82	2	149	54.2	.30	24.3	146	68	166	60	106.00	6.10	1	1.00	1	0	1	1	1	1	1	1	0	0	1
1	1	43	1	157	52.9	-4.40	21.5	137	84	200	54	146.00	5.30	1	1.00	0	1	1	1	1	1	1	1	1	1	1
1	1	77	2	140	53.3	-.10	27.1	137	74	187	63	124.00	5.50	1	1.00	1	0	1	0	1	0	0	0	0	1	1
1	1	83	1	171	52.7	2.50	18.0	116	64	165	60	105.00	5.50	0	1.00	0	0	1	0	1	0	1	1	1	1	1
1	1	65	1	175	88.0	-.90	28.6	145	95	184	37	147.00	6.00	1	1.00	1	0	1	1	1	0	0	0	0	0	1
1	1	69	2	147	41.7	1.00	19.4	105	70	215	105	110.00	6.30	1	1.00	1	0	1	0	1	0	0	1	1	1	1
1	1	75	2	146	57.7	-1.60	27.2	132	67	215	63	152.00	5.20	1	1.00	1	0	1	1	1	0	1	0	0	1	1

1	1	82	2	146	52.5	-.80	24.5	147	80	189	69	120.00	6.60	1	1.00	1	1	1	1	1	1	1	1	0	0	0
1	1	62	1	168	111.0	22.40	39.5	166	100	210	72	138.00	5.30	1	1.00	0	1	1	1	1	1	0	0	0	1	1
0	0	83	1	156	58.6	.80	24.1	191	91	172	87	85.00	5.00	1	1.00	0	0	1	0	1	0	1	1	0	1	1
1	1	86	2	141	43.5	-.20	21.8	116	72	163	45	118.00	5.60	1	1.00	1	0	1	1	1	1	1	1	0	0	1
1	1	56	1	178	91.2	-.90	28.8	128	93	216	54	162.00	5.40	1	1.00	1	0	0	1	1	0	1	0	0	1	1
0	0	60	2	156	45.1	1.50	18.6	117	74	187	65	122.00	5.50	0	1.00	1	0	1	1	1	0	1	1	0	1	1
1	1	44	2	159	62.0	.20	24.6	122	71	203	50	153.00	5.20	1	1.00	1	1	1	0	1	1	1	1	1	1	1
1	1	65	1	157	74.2	-1.90	30.0	130	82	203	58	145.00	6.00	0	1.00	0	0	1	0	1	1	1	0	0	1	1
0	0	31	2	169	67.9	2.00	23.8	99	57	183	74	109.00	4.70	1	1.00	0	0	0	1	1	1	1	1	1	1	1
0	0	62	1	158	62.4	1.50	24.9	156	91	207	48	159.00	5.30	1	1.00	0	0	1	1	1	1	1	1	0	1	1
0	0	71	2	143	43.7	-.50	21.2	146	86	176	75	101.00	5.80	1	1.00	1	0	1	0	1	0	0	1	0	1	1
0	0	84	2	151	57.5	2.00	25.3	116	62	182	64	118.00	5.50	1	1.00	1	0	1	0	1	0	1	0	0	1	1
0	0	52	2	147	56.8	2.10	26.3	112	64	196	64	132.00	5.40	1	1.00	1	0	1	1	0	0	1	0	1	1	1
0	0	42	2	163	62.5	3.60	23.4	124	74	154	60	94.00	5.40	1	1.00	1	0	1	0	1	0	0	1	1	1	1
0	0	79	2	149	47.3	.70	21.4	120	60	208	61	147.00	5.50	1	1.00	1	0	1	0	1	0	0	1	0	1	1
0	0	67	1	155	52.8	.80	21.9	120	74	279	67	212.00	5.40	1	1.00	0	1	1	0	1	1	0	1	1	0	1
1	1	57	1	159	64.7	-1.20	25.5	122	72	187	77	110.00	5.00	1	1.00	0	1	1	0	1	0	1	0	1	1	1
1	1	32	2	154	56.8	-.90	23.9	138	81	240	96	144.00	5.60	1	1.00	1	1	1	1	1	0	1	1	1	0	1
1	1	62	1	165	73.9	-2.50	27.0	123	74	201	62	139.00	5.40	1	1.00	0	1	1	0	1	1	1	0	0	1	1
1	1	63	2	154	55.7	-3.40	23.6	141	86	249	52	197.00	4.90	1	1.00	1	0	1	1	1	1	1	1	0	0	1
0	0	76	1	154	61.6	-2.00	26.0	139	74	273	55	218.00	6.40	1	1.00	1	0	1	0	1	1	1	0	0	0	1
1	1	68	1	161	69.6	.70	26.7	140	83	205	91	114.00	5.70	1	1.00	1	1	1	1	1	1	1	0	0	1	1
0	0	21	2	154	51.1	-.70	21.5	89	49	193	79	114.00	5.10	1	1.00	0	0	1	1	1	0	1	1	1	1	1
0	0	30	2	158	48.0	-.10	19.1	98	59	226	72	154.00	5.20	1	1.00	1	0	0	0	0	1	1	1	1	0	1
0	0	60	1	169	62.4	-1.70	21.9	107	65	148	66	82.00	5.40	1	1.00	0	0	1	0	1	0	1	1	1	1	1
1	1	69	2	144	47.8	.00	23.0	121	66	180	57	123.00	5.40	1	1.00	1	0	1	0	1	1	0	1	1	0	1
0	0	51	2	155	52.2	-1.10	21.6	95	56	214	67	147.00	6.00	1	1.00	1	0	1	1	1	1	1	1	1	1	1
1	1	73	1	162	68.8	-.30	26.3	109	71	181	65	116.00	5.70	1	1.00	1	1	1	0	0	1	1	0	0	1	1
0	0	58	2	157	41.3	-.80	16.8	93	51	212	54	158.00	5.70	1	1.00	0	0	1	0	0	0	0	1	1	1	1
1	1	36	1	168	71.0	-2.60	25.0	152	89	158	55	103.00	5.40	1	1.00	1	1	1	1	1	0	0	0	0	1	1
0	0	62	1	162	68.1	-3.00	26.0	130	91	204	73	131.00	11.30	0	1.00	0	0	1	1	0	1	1	0	0	1	0
1	1	61	2	152	66.3	1.70	28.6	127	84	196	72	124.00	6.10	1	1.00	1	0	1	1	0	1	0	0	0	0	1
1	1	70	1	159	71.4	1.30	28.2	137	82	169	46	123.00	5.70	1	1.00	1	1	1	0	1	1	1	0	0	1	1
0	0	61	1	179	111.2	4.50	34.7	123	80	223	66	157.00	5.80	0	1.00	0	0	1	0	0	0	1	0	0	0	0
1	1	71	2	146	56.9	1.70	26.5	105	72	175	49	126.00	5.60	1	1.00	1	1	1	1	1	1	1	0	0	0	1
1	1	55	2	150	57.1	1.70	25.3	106	73	238	74	164.00	5.40	1	1.00	1	0	1	1	1	1	1	0	1	0	1
1	1	72	2	150	57.9	.60	25.6	124	67	142	52	90.00	5.60	1	1.00	1	1	1	1	1	1	1	0	1	1	1
1	1	60	2	151	58.1	-2.80	25.4	149	84	257	55	202.00	5.30	1	1.00	1	1	1	0	0	1	0	0	0	0	1
1	1	54	2	157	50.8	.80	20.6	113	72	173	55	118.00	5.00	1	1.00	1	1	1	1	0	0	0	1	1	1	1

1	1	65	1	167	72.5	-3.50	25.8	129	74	166	43	123.00	5.40	1	1.00	0	0	1	0	1	1	1	0	1	1	1
1	1	56	1	165	59.8	-.10	21.8	130	83	170	39	131.00	5.10	1	1.00	1	1	1	0	0	0	0	1	1	0	1
1	1	69	2	140	58.4	-1.40	30.0	112	79	162	66	96.00	5.20	1	1.00	1	0	1	1	1	1	1	0	1	1	1
1	1	79	1	157	67.8	-.20	27.6	161	89	151	67	84.00	5.10	1	1.00	0	0	1	1	1	0	1	0	0	1	1
0	0	65	1	165	77.1	-3.20	28.1	135	81	134	43	91.00	5.30	1	1.00	1	0	1	0	0	1	1	0	0	1	1
1	1	60	2	155	67.4	.60	27.9	106	64	211	79	132.00	5.40	1	1.00	1	0	1	1	1	1	1	0	1	1	1
1	1	64	2	154	52.4	.80	22.2	109	72	248	101	147.00	5.70	1	1.00	1	0	1	0	1	0	1	1	1	0	1
1	1	34	2	161	53.4	1.20	20.6	132	85	196	71	125.00	5.30	1	1.00	1	0	1	1	1	1	1	1	1	1	1
1	1	76	2	149	64.8	-1.50	29.0	112	59	154	81	73.00	5.00	1	1.00	1	0	1	1	1	1	1	0	1	1	1
0	0	72	1	157	73.0	-2.40	29.4	137	91	242	43	199.00	5.50	0	1.00	0	1	1	0	0	0	1	0	0	0	1
0	0	65	1	174	71.4	-1.10	23.6	134	78	208	54	154.00	5.70	1	1.00	1	1	1	1	1	1	1	1	0	1	1
0	0	76	1	164	58.1	-2.10	21.5	97	54	212	97	115.00	5.40	1	1.00	1	1	1	0	1	1	1	1	1	1	1
0	0	76	1	164	62.7	-1.50	23.4	120	65	224	61	163.00	6.30	1	1.00	0	1	1	0	1	0	1	1	1	0	0
1	1	69	2	154	55.1	-1.40	23.2	143	72	238	74	164.00	5.80	1	1.00	1	1	1	0	1	0	1	1	0	0	1
0	0	60	2	155	49.1	.60	20.4	116	70	176	69	107.00	5.40	1	1.00	1	0	1	1	1	0	1	1	1	1	1
1	1	64	1	167	66.7	-4.60	24.0	169	115	155	71	84.00	5.20	1	1.00	0	0	1	1	0	1	1	1	0	1	1
0	0	69	2	150	46.4	2.00	20.7	124	67	204	68	136.00	5.40	1	1.00	1	1	1	0	1	1	0	1	0	1	1
0	0	70	2	149	52.5	.70	23.5	111	74	190	93	97.00	5.40	1	1.00	1	0	1	0	1	1	1	1	0	1	1
0	0	71	1	160	58.1	1.90	22.7	149	83	219	52	167.00	5.60	1	1.00	1	0	1	0	1	0	0	1	0	1	1
0	0	86	1	168	66.9	.50	23.6	134	74	182	63	119.00	5.30	1	1.00	0	1	1	1	1	1	0	1	1	1	1
0	0	66	2	154	65.7	.00	27.7	126	74	288	98	190.00	5.80	1	1.00	1	0	1	0	1	0	1	0	1	0	1
1	1	72	2	144	56.2	-.10	27.2	140	78	247	61	186.00	5.60	1	1.00	1	0	1	1	1	0	1	0	0	0	1
0	0	70	2	150	47.3	5.10	21.1	138	74	245	101	144.00	5.30	1	1.00	1	0	1	1	1	0	0	1	1	0	1
0	0	77	1	150	58.4	-2.20	25.8	139	76	229	55	174.00	5.30	1	1.00	1	1	1	0	1	1	1	0	1	0	1
1	1	83	2	148	61.5	.50	28.0	162	88	183	46	137.00	5.80	1	1.00	1	0	1	1	1	1	1	0	0	1	1
0	0	86	2	143	53.5	-.30	26.1	111	69	193	65	128.00	5.20	1	1.00	1	0	1	0	1	0	1	0	0	1	1
0	0	63	2	155	73.4	4.00	30.3	131	80	209	83	126.00	5.70	1	1.00	0	0	1	1	1	0	0	0	0	0	1
0	0	70	1	165	63.9	3.00	23.4	183	104	182	68	114.00	5.80	1	1.00	0	1	1	1	1	1	1	1	0	1	1
0	0	73	2	144	47.0	.80	22.6	115	64	198	77	121.00	5.20	1	1.00	1	0	1	1	1	1	1	1	1	0	1
1	1	74	1	164	58.5	-1.50	21.7	139	73	180	39	141.00	6.50	0	1.00	1	1	0	1	1	1	1	1	1	0	0
1	1	58	2	168	93.0	.00	33.1	140	77	186	43	143.00	5.50	1	1.00	1	0	1	1	1	1	1	0	0	0	1
0	0	49	2	166	56.4	-.60	20.5	103	57	187	84	103.00	5.10	1	1.00	0	0	1	0	1	1	0	1	1	1	1
1	1	60	2	146	42.4	-.80	20.0	101	54	228	74	154.00	5.10	1	1.00	1	0	1	0	1	1	0	1	1	0	1
0	0	86	2	146	41.3	-1.90	19.4	132	75	202	68	134.00	5.80	1	1.00	1	1	1	1	1	1	0	1	0	1	1
0	0	41	1	169	60.0	2.50	21.0	120	82	184	65	119.00	5.20	0	1.00	0	0	1	0	1	1	1	1	1	1	1
1	1	28	2	167	67.6	1.70	24.2	117	73	179	59	120.00	4.90	1	1.00	1	0	1	1	1	1	1	1	1	1	1
1	1	86	1	158	57.6	-1.90	23.0	134	80	230	76	154.00	5.50	1	1.00	1	0	1	0	1	0	1	1	0	0	1
1	1	59	2	155	56.1	-.10	23.5	110	63	228	73	155.00	5.60	1	1.00	1	0	1	0	0	0	0	1	1	0	1
1	1	69	2	147	61.5	-.40	28.3	133	72	190	55	135.00	5.40	0	1.00	1	0	1	1	1	0	0	0	1	0	1

0	0	48	2	157	50.8	-4.00	20.6	123	84	228	84	144.00	5.60	0	1.00	0	0	1	0	1	1	1	1	1	0	1
1	1	76	2	143	49.1	-1.00	24.0	141	88	292	83	209.00	5.90	1	1.00	0	0	1	1	1	1	1	1	0	0	1
0	0	51	2	161	55.5	-.90	21.4	132	80	230	45	185.00	5.50	0	1.00	1	0	0	0	0	0	0	1	1	0	1
0	0	46	2	168	62.3	.30	22.2	102	62	210	65	145.00	5.50	1	1.00	1	1	1	1	0	0	1	1	1	1	1
1	1	56	2	154	56.7	-3.40	24.0	124	75	229	69	160.00	5.60	1	1.00	1	0	1	0	0	0	1	1	1	0	1
1	1	70	1	164	61.9	-1.90	23.1	138	83	214	53	161.00	6.20	1	1.00	0	1	1	1	1	1	1	1	1	1	1
1	1	79	2	159	60.7	2.90	24.1	137	89	218	64	154.00	5.60	1	1.00	0	1	1	1	1	1	1	1	1	0	1
1	1	70	2	148	57.7	.30	26.2	183	90	192	68	124.00	6.00	1	1.00	1	0	1	1	1	1	1	0	0	1	1
0	0	70	1	167	81.7	3.50	29.2	121	85	202	72	130.00	6.40	1	1.00	0	0	1	1	1	1	0	0	0	1	0
1	1	52	2	146	63.1	.40	29.4	151	87	250	49	201.00	5.80	1	1.00	1	0	1	0	1	1	1	0	0	0	1
1	1	67	2	158	61.9	.70	24.8	135	85	200	46	154.00	5.40	1	1.00	1	0	1	1	1	1	0	1	1	1	1
1	1	63	1	170	67.1	-.50	23.2	126	82	182	49	133.00	5.30	0	1.00	0	1	1	1	1	0	1	1	1	1	1
0	0	62	2	157	44.3	1.00	17.9	99	57	237	66	171.00	5.50	1	1.00	1	0	1	1	0	0	0	1	1	0	1
1	1	68	1	165	66.4	-1.50	24.5	166	103	219	70	149.00	6.20	1	1.00	1	0	1	1	1	1	1	1	0	1	1
1	1	70	1	156	53.6	-4.50	22.1	130	72	152	43	109.00	5.70	0	1.00	1	1	1	0	1	0	1	1	0	1	1
1	1	82	1	164	73.1	2.10	27.3	143	72	235	66	169.00	5.90	1	1.00	0	0	1	1	1	1	1	0	0	0	1
1	1	65	2	143	48.2	1.70	23.5	115	67	182	71	111.00	9.70	1	1.00	1	0	1	1	1	0	1	1	1	1	0
0	0	70	1	159	64.4	-1.60	25.5	116	80	175	57	118.00	5.70	1	1.00	0	0	1	1	0	0	1	0	1	1	1
0	0	64	1	161	53.2	-1.70	20.6	140	83	149	38	111.00	5.30	0	1.00	0	1	1	0	1	1	1	1	0	0	1
0	0	71	1	163	75.9	-1.40	28.5	105	71	222	53	169.00	5.50	1	1.00	0	1	1	0	1	0	1	0	1	0	1
0	0	63	2	155	71.4	-.80	29.6	141	83	273	50	223.00	5.90	1	1.00	1	0	1	0	0	0	0	0	0	0	1
1	1	64	1	160	63.8	-3.30	24.8	127	94	284	105	179.00	5.50	1	1.00	0	0	1	1	1	1	1	1	0	0	1
0	0	71	2	154	67.3	-.20	28.4	137	78	240	46	194.00	5.70	1	1.00	1	1	0	0	1	1	0	0	1	0	1
1	1	78	1	164	57.2	1.10	21.2	149	73	175	34	141.00	6.10	1	1.00	0	0	1	0	1	0	0	1	0	0	1
0	0	80	1	157	65.0	1.90	26.4	174	93	171	46	125.00	5.30	1	1.00	1	0	1	0	1	1	1	0	0	1	1
1	1	64	1	164	57.1	-4.40	21.1	135	72	187	88	99.00	7.50	0	1.00	0	1	1	0	1	1	1	1	1	1	0
1	1	74	2	149	45.4	-.50	20.3	126	75	254	119	135.00	6.20	1	1.00	1	0	1	1	1	1	1	1	0	0	1
1	1	51	1	171	69.7	-.70	23.9	103	68	297	46	251.00	5.70	0	1.00	1	1	1	0	0	1	1	1	1	0	1
0	1	65	2	147	51.3	-1.60	23.8	175	104	244	82	162.00	5.60	1	1.00	1	0	1	1	1	1	1	1	0	0	1
0	0	75	1	159	57.5	-.80	22.6	155	89	196	47	149.00	7.20	1	1.00	1	1	0	1	1	0	1	1	0	1	0
0	0	73	2	148	46.0	1.10	21.1	119	63	174	53	121.00	5.00	1	1.00	1	1	1	0	1	0	1	1	1	1	1
0	0	40	1	178	81.4	1.00	25.8	127	87	160	42	118.00	8.90	0	1.00	0	1	0	1	1	0	0	0	1	1	0
0	0	37	1	171	64.3	-.70	21.8	125	73	238	76	162.00	5.40	0	1.00	1	1	0	1	1	1	1	1	1	0	1
0	0	71	2	155	51.2	-.30	21.3	114	64	239	73	166.00	5.40	1	1.00	1	0	1	0	1	0	0	1	1	0	1
0	0	50	1	161	59.9	.50	22.9	124	71	236	63	173.00	5.20	0	1.00	0	1	0	0	0	1	0	1	1	0	1
0	0	46	2	150	58.6	2.20	25.9	134	93	260	36	224.00	5.80	0	1.00	0	0	0	1	1	1	1	0	0	0	1
1	1	25	2	159	46.5	2.00	18.3	103	62	195	70	125.00	5.10	0	1.00	1	0	0	0	1	0	1	1	1	1	1
0	0	47	1	174	61.5	.60	20.3	131	85	209	73	136.00	5.10	0	1.00	0	1	1	0	1	1	1	1	1	1	1
1	1	64	1	161	69.8	-2.10	27.0	135	80	185	62	123.00	5.40	1	1.00	1	1	1	0	1	1	1	0	0	1	1

1	1	48	2	155	63.2	-.30	26.2	133	87	206	93	113.00	5.30	1	1.00	1	1	1	0	1	0	0	0	1	1	1
1	1	74	2	142	48.7	-.90	24.1	145	70	231	60	171.00	5.40	1	1.00	1	1	1	1	1	1	1	1	0	0	1
1	1	65	2	147	62.2	-.10	28.7	185	97	229	68	161.00	5.40	1	1.00	1	0	1	0	1	1	1	0	0	0	1
1	1	65	1	167	82.4	-.50	29.6	143	81	165	42	123.00	6.00	1	1.00	0	0	1	1	1	1	1	0	0	1	1
1	1	64	2	163	65.0	2.70	24.4	117	66	194	59	135.00	5.30	1	1.00	1	0	1	1	1	0	0	1	1	1	1
1	1	81	1	161	56.6	-.10	21.9	153	77	181	48	133.00	5.50	1	1.00	1	0	1	1	1	0	0	1	0	1	1
1	1	68	2	158	64.0	1.60	25.5	148	79	226	65	161.00	5.30	1	1.00	1	0	1	1	1	1	1	0	0	0	1
1	1	69	1	170	78.8	.40	27.1	111	78	177	51	126.00	5.70	0	1.00	0	1	1	1	1	1	1	0	1	1	1
1	1	77	2	143	50.8	1.20	24.7	117	63	184	73	111.00	5.80	1	1.00	1	0	1	1	1	1	1	1	1	1	1
1	1	73	1	159	57.3	-.60	22.6	119	71	199	52	147.00	7.20	1	1.00	1	1	1	0	1	0	1	1	1	1	0
1	1	67	1	166	68.0	-.40	24.5	126	73	191	67	124.00	5.50	1	1.00	1	0	1	1	1	1	1	1	0	1	1
0	0	80	2	142	48.3	-.40	23.8	117	61	192	75	117.00	5.40	1	1.00	1	0	1	1	1	0	0	1	1	1	1
1	1	83	2	144	50.8	-1.70	24.4	110	57	196	83	113.00	5.50	1	1.00	1	1	1	1	1	1	1	1	0	1	1
1	1	81	2	160	55.5	-.40	21.5	115	61	167	72	95.00	5.40	1	1.00	1	0	1	0	1	0	1	1	0	1	1
1	1	76	2	147	38.1	2.10	17.7	120	73	206	57	149.00	5.80	1	1.00	1	0	1	1	1	1	0	1	1	1	1
1	1	75	2	146	62.6	2.30	29.4	152	83	238	93	145.00	5.20	1	1.00	1	0	1	0	1	0	1	0	0	0	1
1	1	67	2	157	60.8	1.60	24.6	107	69	183	45	138.00	5.40	1	1.00	1	0	1	1	1	1	1	1	0	1	1
1	1	74	1	161	71.1	-.70	27.4	128	75	190	40	150.00	5.80	1	1.00	1	0	1	1	1	1	0	0	0	1	1
0	0	70	2	154	58.8	-.80	24.7	141	79	178	51	127.00	6.10	1	1.00	1	0	1	1	1	1	0	1	0	1	0
0	0	56	1	174	71.1	-2.00	23.5	130	76	230	50	180.00	5.20	0	2.00	0	1	1	0	1	0	0	1	1	0	1
1	1	44	2	159	53.1	-1.20	20.9	113	69	254	103	151.00	4.90	1	1.00	1	1	1	0	1	1	1	1	1	0	1
1	1	61	2	158	46.0	-1.20	18.3	112	82	157	80	77.00	5.50	1	1.00	1	1	0	1	1	1	1	1	1	1	1
1	1	68	2	144	52.4	-1.20	25.4	148	77	253	85	168.00	5.00	1	1.00	1	0	1	1	0	0	1	0	0	0	1
0	0	33	2	156	57.4	-.20	23.6	88	57	170	75	95.00	5.30	1	1.00	1	1	1	1	1	1	1	1	1	1	1
0	0	68	2	147	46.6	.50	21.4	121	82	151	80	71.00	5.50	1	1.00	1	0	1	0	1	0	0	1	0	1	1
1	1	60	2	146	56.3	.60	26.5	103	68	213	63	150.00	5.40	1	1.00	1	0	1	0	1	0	0	0	1	1	1
1	1	57	1	171	68.6	-2.70	23.4	126	80	191	42	149.00	5.00	0	1.00	0	0	1	0	1	1	1	1	0	1	1
0	0	60	2	154	55.0	1.10	23.2	125	71	246	100	146.00	5.50	1	1.00	0	0	1	1	0	1	1	1	0	0	1
0	0	61	1	167	79.9	-1.90	28.7	125	63	162	38	124.00	6.20	0	2.00	0	1	1	1	1	1	1	0	0	0	1
0	0	76	1	155	56.3	-.40	23.5	128	71	165	46	119.00	6.70	1	1.00	0	0	1	0	1	1	1	1	0	1	0
1	1	60	2	156	50.7	1.30	20.9	139	76	158	70	88.00	7.40	1	1.00	1	1	1	1	1	0	0	1	1	1	0
0	0	67	1	167	54.1	-2.50	19.3	127	73	130	55	75.00	6.30	1	1.00	1	1	1	0	0	1	1	1	1	1	0
0	0	73	1	164	56.0	-1.30	20.9	122	69	193	49	144.00	5.50	0	1.00	1	1	0	0	1	1	0	1	0	1	1
0	0	37	1	171	84.6	-1.90	29.0	131	78	182	50	132.00	5.20	1	1.00	1	0	1	0	1	0	0	0	1	0	1
0	0	74	2	149	58.4	.60	26.3	132	81	211	74	137.00	5.00	1	1.00	1	0	1	1	1	1	1	0	1	1	1
0	0	74	1	161	61.7	-2.00	23.8	130	69	174	52	122.00	4.90	1	1.00	0	0	1	1	1	1	1	1	0	1	1
1	1	61	1	157	52.3	-1.10	21.1	146	81	193	61	132.00	5.10	0	1.00	0	1	1	0	1	1	1	1	0	1	1
0	0	73	1	160	83.0	1.30	32.4	128	79	219	72	147.00	5.30	1	1.00	0	0	1	1	1	1	1	0	0	1	1
1	1	73	2	147	46.6	-.70	21.5	152	84	170	58	112.00	5.40	1	1.00	1	0	1	1	1	1	1	1	0	1	1

1	1	70	1	159	63.0	.00	24.8	113	62	184	102	82.00	7.30	1	1.00	1	0	1	1	1	1	1	1	0	0	0
1	1	72	2	147	41.3	-1.30	19.1	97	54	172	57	115.00	6.00	1	1.00	1	0	1	0	1	0	0	1	1	0	1
1	1	45	2	141	64.5	2.70	32.2	109	66	189	72	117.00	5.30	1	1.00	1	0	1	0	1	0	1	0	1	1	1
0	0	56	2	148	66.0	2.70	29.9	112	62	228	85	143.00	6.80	1	1.00	1	0	1	0	1	0	0	0	1	0	0
0	0	81	2	152	59.7	2.80	25.8	163	78	213	51	162.00	5.50	1	1.00	1	0	1	1	1	0	1	0	0	1	1
1	1	28	2	164	67.5	-.90	25.2	110	72	175	52	123.00	5.70	1	1.00	1	1	1	1	1	1	1	0	1	1	1
0	0	43	2	162	48.3	-.60	18.3	88	57	160	83	77.00	5.10	0	1.00	0	1	0	0	1	0	0	1	1	1	1
1	1	48	2	152	57.0	-.10	24.6	153	84	221	83	138.00	5.60	1	1.00	1	1	1	0	1	0	0	1	0	0	1
1	1	46	2	165	55.4	1.30	20.2	114	76	226	57	169.00	5.50	0	1.00	0	1	1	1	1	0	1	1	0	0	1
1	1	36	2	158	46.1	-.50	18.5	98	60	175	76	99.00	5.40	1	1.00	1	1	1	1	1	1	1	1	1	1	1
1	1	33	1	164	64.1	.50	23.7	112	72	160	81	79.00	5.40	0	1.00	0	0	1	0	0	1	1	1	1	1	1
1	1	72	2	146	45.0	3.00	21.1	124	69	212	86	126.00	5.70	1	1.00	1	0	1	1	1	0	1	1	1	1	1
0	0	43	1	169	90.9	1.60	31.7	122	67	218	40	178.00	5.70	0	1.00	0	0	0	0	0	0	0	0	1	1	1
1	1	67	1	163	58.1	.20	21.8	110	73	222	56	166.00	6.10	1	1.00	0	0	1	0	1	0	1	1	1	0	1
0	0	57	2	160	42.4	-.90	16.6	135	79	186	74	112.00	5.20	1	1.00	1	0	1	1	1	1	1	1	1	1	1
1	1	63	2	152	65.7	-1.10	28.3	108	62	207	58	149.00	5.80	1	1.00	0	0	1	0	1	0	0	0	0	1	1
1	1	62	1	163	69.6	.70	26.1	129	72	170	34	136.00	7.30	0	1.00	1	0	1	0	1	1	1	0	0	0	0
1	1	59	2	159	48.2	-1.50	19.1	105	65	332	108	224.00	5.50	1	1.00	1	1	1	1	0	1	0	1	1	0	1
1	1	67	1	169	62.6	.60	22.0	111	66	175	56	119.00	5.60	1	1.00	1	0	1	0	1	0	0	1	1	1	1
1	1	40	1	172	66.2	5.90	22.4	118	66	216	62	154.00	5.00	1	1.00	1	0	1	0	0	0	0	1	1	1	1
1	1	63	2	155	71.8	-1.10	29.9	156	86	180	56	124.00	6.60	1	1.00	1	0	1	0	1	0	1	0	0	0	0
1	1	71	1	146	50.6	-.70	23.7	148	78	63	28	35.00	5.20	1	1.00	0	0	0	1	1	1	0	1	0	0	1
1	1	78	1	165	67.9	1.80	25.0	107	56	184	88	96.00	5.30	1	1.00	0	1	1	1	1	1	1	0	0	1	1
1	1	77	1	154	60.5	2.80	25.4	138	77	210	75	135.00	5.20	1	1.00	0	1	1	0	1	1	0	0	0	1	0
1	1	42	2	170	57.3	.70	19.8	98	63	207	78	129.00	5.30	1	1.00	1	0	1	0	1	1	1	1	1	1	1
1	1	38	2	151	48.6	.10	21.2	114	75	187	92	95.00	5.20	0	1.00	0	1	1	0	1	0	1	1	1	1	1
1	1	64	2	152	52.1	-.60	22.6	130	73	147	74	73.00	5.10	1	1.00	1	1	1	0	0	1	0	1	0	1	1
1	1	86	1	158	44.4	.00	17.7	106	55	146	74	72.00	5.90	1	1.00	1	0	1	1	1	1	1	1	1	1	0
0	0	56	2	158	59.2	.10	23.6	111	74	178	63	115.00	5.40	1	1.00	1	1	1	1	1	1	1	1	0	1	1
0	0	66	1	162	71.9	.20	27.4	146	79	174	59	115.00	6.10	1	1.00	0	0	1	1	1	1	0	0	0	1	1
1	1	72	2	151	67.7	.00	29.6	159	85	198	95	103.00	5.40	1	1.00	1	1	1	0	1	0	1	0	0	1	1
1	1	81	1	165	65.8	.40	24.1	152	78	167	43	124.00	5.30	1	1.00	1	0	1	1	1	1	1	1	0	1	1
1	1	66	2	148	65.8	-.80	29.8	121	79	219	54	165.00	5.90	1	1.00	1	0	1	1	1	1	1	0	0	1	1
0	0	28	2	157	53.9	-1.70	21.8	101	62	172	83	89.00	5.30	1	1.00	1	0	1	1	1	0	1	1	1	1	1
1	1	58	2	155	53.1	-.70	22.1	115	69	233	60	173.00	4.90	1	1.00	1	0	1	0	0	0	0	1	1	0	1
1	1	86	1	155	43.2	2.10	17.9	144	79	234	114	120.00	5.80	0	1.00	0	1	1	1	1	1	1	1	0	0	1
0	0	69	2	150	54.5	.20	24.1	158	94	211	53	158.00	6.10	1	1.00	1	0	1	1	1	0	1	1	0	1	1
1	1	54	1	169	70.5	-1.30	24.8	124	82	299	36	263.00	7.50	1	1.00	0	0	1	1	1	1	1	1	0	0	0
1	1	61	1	165	63.8	-.60	23.4	112	77	213	66	147.00	5.70	0	2.00	0	0	1	0	1	1	1	1	1	1	1

1	1	53	2	153	53.4	-1.50	22.7	99	63	218	85	133.00	5.60	1	1.00	0	1	1	1	1	1	1	1	1	1	1
0	0	70	2	138	37.0	1.30	19.3	114	71	180	55	125.00	5.30	1	1.00	1	0	1	1	1	1	1	1	0	1	1
1	1	60	2	155	61.4	1.50	25.4	137	89	217	52	165.00	5.50	1	1.00	1	1	1	1	1	1	1	0	0	1	1
0	0	68	1	164	75.2	-.80	28.0	149	96	238	54	184.00	5.20	1	1.00	1	0	1	0	1	0	0	0	0	0	1
0	0	47	1	175	61.7	5.30	20.1	120	80	267	57	210.00	5.40	0	1.00	1	0	0	0	1	1	1	1	1	0	1
0	0	84	1	157	63.3	.20	25.8	136	70	187	46	141.00	5.40	1	1.00	0	0	1	0	1	1	1	0	0	1	1
1	1	66	2	153	51.3	.00	21.9	114	63	265	84	181.00	5.50	1	1.00	1	0	1	0	1	1	1	1	1	0	1
1	1	74	2	149	53.2	1.00	23.8	143	84	193	52	141.00	5.20	1	1.00	1	1	1	1	1	1	1	1	0	1	1
1	1	72	2	135	44.1	-.20	24.3	143	77	205	46	159.00	5.80	1	1.00	1	1	1	0	1	1	1	1	0	1	1
0	0	62	1	162	68.5	-1.50	26.0	119	70	209	51	158.00	5.40	0	1.00	1	0	1	1	1	1	1	0	1	1	1
1	1	66	1	160	63.0	-2.90	24.6	118	77	258	66	192.00	5.50	1	1.00	0	1	1	0	0	1	1	1	1	0	1
1	1	31	2	162	70.8	-.70	27.1	112	65	168	62	106.00	5.60	1	1.00	1	0	1	1	1	0	0	0	1	1	1
1	1	55	2	146	52.9	-.90	24.9	102	61	222	72	150.00	6.10	1	1.00	0	1	1	1	1	1	1	1	1	0	1
0	0	64	2	149	54.3	2.40	24.3	125	88	293	89	204.00	5.40	1	1.00	0	0	1	1	1	0	1	1	1	0	1
1	1	57	2	153	54.1	.70	23.0	142	85	236	54	182.00	5.70	1	1.00	1	1	1	1	0	1	1	1	0	0	1
1	1	45	1	171	56.4	-.70	19.2	105	57	163	46	117.00	6.10	1	1.00	0	1	1	1	1	1	1	1	1	1	1
1	1	62	2	155	60.7	1.50	25.1	122	74	220	44	176.00	5.50	1	1.00	1	0	1	0	0	1	1	0	1	0	1
1	1	29	2	162	56.0	-4.00	21.2	116	70	134	73	61.00	5.20	0	1.00	0	0	0	0	1	0	0	1	1	1	1
1	1	61	2	156	54.6	-.30	22.3	109	64	264	51	213.00	5.30	1	1.00	1	0	1	1	1	1	1	1	1	0	1
1	1	71	2	148	47.7	.30	21.6	121	72	193	87	106.00	5.50	1	1.00	1	0	1	1	1	1	1	1	1	0	1
1	1	75	2	144	37.6	.20	18.0	144	79	238	85	153.00	5.40	1	1.00	1	0	1	0	1	1	0	1	0	0	1
0	0	58	2	152	66.7	-.10	29.0	122	73	193	45	148.00	5.90	1	1.00	1	0	1	0	0	1	1	0	1	0	1
0	0	77	2	145	35.9	-.30	17.1	158	88	213	92	121.00	5.50	0	1.00	1	1	1	0	1	1	1	1	0	1	1
1	1	66	2	153	54.7	-1.90	23.4	106	72	223	32	191.00	5.80	1	1.00	0	0	1	1	1	1	0	1	1	0	1
1	1	82	2	161	80.8	-.80	31.0	143	68	259	53	206.00	6.10	1	1.00	1	0	1	1	1	1	0	0	0	0	1
1	1	72	1	156	63.0	-1.90	25.7	156	85	221	67	154.00	5.80	1	1.00	0	1	1	1	1	1	1	0	0	0	1
1	1	76	2	151	50.4	-.90	22.1	125	75	152	38	114.00	5.70	1	1.00	1	0	1	0	1	0	0	1	1	0	1
1	1	68	1	165	70.8	-3.00	25.9	145	84	289	42	247.00	6.00	1	1.00	0	1	1	1	0	1	1	0	0	0	1
1	1	77	2	143	53.6	1.30	26.1	138	78	216	54	162.00	5.80	1	1.00	1	0	1	1	1	1	1	0	0	1	1
1	1	63	2	157	54.9	-1.80	22.3	116	78	245	78	167.00	5.40	1	1.00	0	0	1	0	1	0	0	1	1	0	1
0	0	75	2	150	44.7	.30	19.9	108	65	229	113	116.00	5.60	1	1.00	1	0	1	0	1	0	0	1	0	0	1
0	0	85	2	150	57.3	-1.80	25.4	158	78	208	47	161.00	5.60	1	1.00	1	0	1	0	1	1	1	0	0	0	1
1	1	48	2	161	82.2	-.30	31.7	140	81	222	72	150.00	5.70	1	1.00	0	0	1	0	1	1	1	0	0	0	1
1	1	76	1	157	67.4	1.50	27.2	135	80	206	58	148.00	5.90	1	1.00	0	1	1	1	1	1	1	0	0	1	1
0	0	67	1	170	55.2	2.60	19.1	123	79	268	56	212.00	5.90	0	1.00	0	1	1	1	1	1	1	1	1	0	1
1	1	77	2	153	45.5	.00	19.5	102	59	204	80	124.00	5.30	1	1.00	1	1	1	0	1	0	0	1	1	1	1
1	1	60	2	158	62.3	-1.00	25.1	119	76	209	60	149.00	5.60	1	1.00	1	1	1	1	1	1	0	0	1	1	1
1	1	64	1	172	84.3	-4.80	28.4	131	78	204	42	162.00	6.10	0	1.00	0	1	1	0	1	1	1	0	1	1	1
1	1	41	2	153	44.9	.10	19.1	99	59	248	92	156.00	5.20	1	1.00	1	1	1	0	1	0	1	1	1	0	1

0	0	53	2	155	56.8	-3.40	23.7	112	76	197	68	129.00	5.70	1	1.00	1	0	1	1	0	0	1	1	0	1	1
0	0	54	2	153	64.3	-2.30	27.3	142	81	326	91	235.00	5.20	1	1.00	1	0	0	0	0	0	0	0	0	0	1
0	0	43	2	165	66.8	1.20	24.6	107	69	245	90	155.00	5.40	1	1.00	1	1	1	0	1	0	0	1	1	0	1
0	0	64	2	145	46.9	1.10	22.3	131	84	242	87	155.00	5.00	1	1.00	1	1	1	1	1	1	1	1	1	0	1
0	0	75	1	167	48.3	-.80	17.2	129	81	173	65	108.00	5.70	0	1.00	1	0	1	0	1	0	1	1	1	1	1
1	1	68	2	152	60.2	.70	26.0	156	98	209	47	162.00	5.30	1	1.00	1	1	1	0	1	1	1	0	0	1	1
1	1	59	2	156	65.4	-2.40	27.0	136	86	235	65	170.00	5.30	1	1.00	1	1	1	1	1	0	0	0	1	0	1
1	1	67	1	165	49.1	-.40	18.1	117	74	226	111	115.00	5.20	1	1.00	0	1	1	1	1	0	0	1	1	0	1
1	1	77	2	152	44.0	-.70	18.9	141	76	216	93	123.00	4.80	1	1.00	1	1	1	1	1	1	1	1	0	1	1
0	0	59	1	165	58.3	.90	21.5	125	79	196	70	126.00	5.80	1	1.00	1	0	1	0	1	0	1	1	1	1	1
1	1	58	2	145	48.3	-.70	22.9	114	69	253	65	188.00	5.30	1	1.00	1	0	1	0	0	0	0	1	1	0	1
1	1	77	2	140	51.3	-2.30	26.1	148	77	225	57	168.00	5.00	1	1.00	1	1	1	0	1	1	1	0	0	0	1
1	1	76	1	167	71.5	-1.30	25.6	127	55	177	46	131.00	8.50	1	1.00	0	0	1	0	1	1	1	0	1	1	0
1	1	67	1	169	68.6	.10	23.9	114	77	198	53	145.00	7.70	1	1.00	0	1	1	0	1	1	1	1	0	0	0
1	1	60	1	160	61.5	2.10	23.9	104	58	120	40	80.00	6.30	1	1.00	1	0	1	0	1	1	1	1	0	0	0
1	1	72	1	162	65.5	-1.20	25.0	104	60	158	50	108.00	5.40	1	1.00	0	0	1	1	1	1	1	0	1	1	1
1	1	80	2	149	54.4	2.30	24.3	125	81	230	61	169.00	5.70	1	1.00	1	0	1	0	1	1	1	1	0	0	1
1	1	69	2	146	54.1	.30	25.5	137	72	203	45	158.00	5.50	1	1.00	1	0	1	0	1	0	0	0	0	1	1
1	1	74	1	167	64.7	-.10	23.1	121	76	176	60	116.00	5.40	1	1.00	1	0	1	1	1	1	1	1	0	1	1
1	1	67	1	164	82.7	-.50	30.6	155	102	146	38	108.00	6.00	1	1.00	0	0	1	0	0	0	0	0	0	0	1
1	1	63	2	149	54.8	2.80	24.6	129	86	185	71	114.00	5.80	1	1.00	1	0	1	1	1	0	1	1	0	0	1
1	1	75	2	142	56.3	3.70	27.7	146	79	148	42	106.00	5.40	1	1.00	1	0	1	1	1	1	0	0	0	1	1
1	1	69	2	160	54.8	.20	21.2	93	51	236	78	158.00	5.80	1	1.00	1	1	1	1	1	1	1	1	1	0	1
1	1	76	2	144	53.0	-1.20	25.4	174	107	219	88	131.00	5.10	1	1.00	0	0	1	1	0	1	1	0	0	1	1
1	1	77	1	165	62.5	3.60	22.9	138	77	200	64	136.00	5.50	1	1.00	1	0	1	1	1	1	1	1	0	1	1
0	0	69	1	161	69.0	-3.20	26.5	128	79	292	50	242.00	5.80	1	1.00	1	0	1	0	1	0	1	0	1	0	1
1	1	70	2	150	65.6	.80	29.0	149	93	333	51	282.00	5.50	1	1.00	1	1	1	0	1	1	0	0	0	0	1
0	0	76	2	149	45.7	-2.40	20.6	120	65	211	80	131.00	5.10	1	1.00	1	0	1	0	1	1	1	1	1	1	1
0	0	57	2	152	58.1	4.40	25.2	120	61	249	93	156.00	5.40	1	1.00	1	1	1	1	1	1	1	0	1	0	1
1	1	65	1	166	70.3	-4.80	25.5	163	100	197	64	133.00	5.40	0	1.00	0	1	1	1	1	1	1	0	0	1	1
0	0	73	2	156	53.1	1.40	21.7	143	88	229	102	127.00	5.40	1	1.00	1	0	1	0	1	0	0	1	0	0	1
0	1	54	2	157	59.2	-2.30	24.0	112	78	242	55	187.00	5.50	1	1.00	0	0	1	1	0	1	1	1	1	0	1
0	0	66	1	156	56.4	.10	23.0	137	83	140	52	88.00	5.20	1	1.00	0	0	1	0	0	0	0	1	0	1	1
0	0	59	2	148	60.4	2.50	27.6	162	94	266	71	195.00	6.00	1	1.00	1	1	1	0	1	1	1	0	0	0	1
0	0	81	1	158	59.7	-3.30	23.8	148	80	201	70	131.00	5.70	1	1.00	1	0	1	1	1	1	1	1	0	1	1
0	0	62	2	147	56.9	-2.10	26.1	142	83	170	41	129.00	4.90	1	1.00	1	0	0	1	0	1	1	0	0	1	1
1	1	66	1	165	55.9	1.10	20.4	117	81	196	59	137.00	6.00	1	1.00	0	1	1	0	1	1	0	1	1	1	1
1	1	66	2	156	69.8	1.40	28.7	132	77	203	46	157.00	5.90	1	1.00	1	0	1	1	1	0	1	0	0	0	1
0	0	62	1	162	62.7	-1.60	23.8	144	93	219	43	176.00	5.20	1	1.00	0	0	1	0	1	0	0	1	0	1	1

0	0	65	2	153	60.3	2.40	25.6	106	60	221	56	165.00	5.70	1	1.00	0	0	0	1	1	0	0	0	1	0	1
0	0	62	1	164	70.1	3.60	26.1	135	82	163	41	122.00	5.40	0	1.00	0	1	1	0	1	1	0	0	0	1	1
1	1	42	2	161	62.7	-3.00	24.3	95	57	192	69	123.00	5.00	1	1.00	1	1	1	0	1	0	0	1	1	1	1
1	1	64	2	142	49.9	-4.20	24.6	125	79	231	56	175.00	5.50	0	1.00	1	0	0	0	1	0	1	1	0	0	1
0	0	76	2	150	41.6	-.80	18.5	133	68	195	98	97.00	6.10	1	1.00	1	1	1	1	1	1	1	1	1	0	1
0	0	63	2	143	61.2	1.50	29.9	142	92	163	74	89.00	5.50	1	1.00	1	0	1	1	0	1	0	0	0	1	1
0	0	64	2	166	64.6	3.60	23.5	99	68	222	49	173.00	5.10	1	1.00	1	0	1	1	1	0	1	1	1	0	1
1	1	62	1	161	64.7	-1.40	24.8	120	74	195	73	122.00	5.50	1	1.00	0	1	1	1	1	1	1	1	0	1	1
1	1	77	2	143	52.0	-.40	25.3	142	86	172	57	115.00	5.80	1	1.00	1	0	1	1	1	0	0	0	0	0	1
1	1	25	2	159	56.4	.60	22.4	110	76	206	69	137.00	5.30	1	1.00	1	0	1	1	0	0	1	1	1	1	1
1	1	36	2	149	74.2	-.70	33.4	114	69	192	79	113.00	5.00	0	1.00	0	0	1	1	0	0	0	0	1	1	1
1	1	85	1	159	52.7	-1.30	20.9	138	74	188	83	105.00	5.10	1	1.00	0	1	1	0	1	1	1	1	1	1	1
0	0	57	2	159	48.8	.70	19.2	108	62	222	63	159.00	5.30	1	1.00	1	0	1	1	1	0	0	1	1	0	1
1	1	74	1	162	63.3	1.30	24.1	111	62	177	44	133.00	5.70	1	1.00	0	0	1	0	1	0	1	1	1	1	1
1	1	77	2	147	43.9	2.10	20.2	136	80	222	62	160.00	5.60	1	1.00	1	1	1	1	1	0	1	1	0	0	1
1	1	64	2	147	59.5	-.40	27.6	98	70	201	61	140.00	5.50	1	1.00	1	0	1	1	1	0	0	0	1	1	1
0	0	60	1	166	58.8	-.50	21.2	122	73	154	65	89.00	5.50	0	1.00	0	0	1	1	1	0	0	1	1	1	1
1	1	67	2	158	53.7	.20	21.6	153	84	222	68	154.00	5.60	1	1.00	1	0	1	1	1	1	1	1	0	0	1
1	1	56	2	157	66.3	-.40	26.9	165	100	225	68	157.00	5.50	0	1.00	0	1	1	0	0	0	0	0	0	0	1
0	0	64	2	151	48.8	2.60	21.4	103	70	232	59	173.00	5.70	1	1.00	1	0	1	0	1	0	0	1	1	0	1
0	0	64	2	155	52.8	-1.50	21.9	99	55	189	65	124.00	5.50	1	1.00	1	0	1	1	1	1	1	1	1	1	1
1	1	45	2	156	45.7	.20	18.8	105	62	187	78	109.00	5.60	0	1.00	1	1	1	0	1	0	0	1	1	1	1
0	0	53	1	160	90.3	4.10	35.4	130	105	207	46	161.00	6.50	1	1.00	0	0	0	1	1	1	1	0	0	1	0
1	1	31	2	162	63.5	-4.30	24.1	111	58	307	59	248.00	4.80	0	1.00	1	0	0	0	1	0	0	1	1	0	1
1	1	59	2	154	52.9	.20	22.2	146	97	270	84	186.00	5.50	1	1.00	1	1	1	0	1	0	1	1	0	0	1
1	1	75	1	142	47.2	-2.00	23.3	158	98	149	82	67.00	5.10	1	1.00	0	0	1	1	1	1	1	1	0	1	1
1	1	60	2	153	61.5	1.60	26.3	106	69	265	61	204.00	5.60	1	1.00	0	0	1	0	1	1	1	0	1	0	1
1	1	39	1	171	61.3	-3.50	20.8	104	59	213	57	156.00	5.80	1	1.00	1	0	1	0	0	0	0	1	1	1	1
1	1	68	1	153	57.3	.50	24.5	115	65	218	46	172.00	5.30	0	1.00	1	1	1	1	1	1	1	1	1	1	1
1	1	52	2	152	44.8	-.20	19.4	108	63	280	64	216.00	6.30	1	1.00	1	0	1	0	1	0	0	1	1	0	1
1	1	51	2	155	63.9	-2.70	26.5	127	80	203	73	130.00	5.70	1	1.00	1	0	1	0	1	1	1	0	1	1	1
1	1	72	1	167	75.7	.30	27.3	151	81	214	36	178.00	5.80	1	1.00	0	0	1	0	0	1	1	0	0	0	1
1	1	81	2	149	71.5	1.90	32.2	136	79	192	72	120.00	5.50	1	1.00	1	0	1	1	1	0	1	0	0	0	1
0	0	74	2	148	57.8	.80	26.2	108	57	157	75	82.00	5.30	1	1.00	1	0	1	0	1	0	1	0	0	1	1
1	1	72	1	160	65.6	-2.50	25.6	126	81	298	68	230.00	5.60	1	1.00	1	1	1	1	1	1	1	0	0	0	1
1	1	75	2	146	52.7	-.90	24.6	139	83	146	47	99.00	5.40	1	1.00	1	0	1	1	1	1	1	1	0	1	1
1	1	65	2	155	65.6	-.80	27.4	128	70	171	53	118.00	5.70	1	1.00	1	0	1	0	1	1	1	0	0	1	1
1	1	31	2	155	51.1	.60	21.2	106	64	176	88	88.00	5.10	1	1.00	0	0	1	0	1	0	0	1	1	1	1
1	1	70	1	167	64.4	-.80	23.0	111	69	172	90	82.00	6.30	1	1.00	0	0	1	1	1	0	1	1	1	0	0

1	1	69	1	163	69.1	-1.10	25.9	116	64	201	96	105.00	7.20	1	1.00	0	0	1	1	1	0	1	0	1	1	0
1	1	68	1	164	62.7	-1.10	23.3	159	84	185	82	103.00	6.70	0	1.00	0	0	1	0	1	1	1	1	0	1	0
1	1	60	2	145	48.6	-1.00	23.2	122	64	191	60	131.00	5.40	1	1.00	1	1	1	1	1	1	1	1	1	1	1
0	0	42	2	158	50.0	2.00	19.9	106	68	195	83	112.00	5.40	1	1.00	0	0	1	1	1	1	1	1	1	1	1
1	1	57	2	154	63.0	-1.20	26.7	157	97	250	40	210.00	6.70	1	1.00	0	0	1	1	0	1	0	0	0	0	0
1	1	72	1	160	68.5	-2.20	26.9	92	54	182	54	128.00	5.70	1	1.00	1	1	1	0	1	1	1	0	0	1	1
1	1	74	2	149	42.2	-.10	19.0	128	79	227	72	155.00	5.50	1	1.00	1	1	1	1	1	0	1	1	1	0	1
1	1	74	2	144	57.4	-2.40	27.6	126	74	212	49	163.00	6.40	1	1.00	1	0	1	0	1	0	0	0	0	1	1
0	0	77	1	153	63.0	-.50	26.9	159	86	213	65	148.00	5.90	0	2.00	0	0	1	0	1	0	0	0	0	1	0
0	0	70	2	153	62.4	.70	26.5	162	99	269	48	221.00	5.80	1	1.00	1	0	1	1	0	1	1	0	0	0	1
1	1	58	1	168	65.7	-.80	23.2	153	87	189	55	134.00	4.90	0	1.00	0	0	1	0	0	0	0	1	0	1	1
1	1	46	2	157	66.9	.60	27.3	115	75	193	74	119.00	5.60	1	1.00	0	1	1	1	1	1	1	0	1	1	1
1	1	64	2	147	47.9	.50	22.1	135	78	234	52	182.00	5.60	1	1.00	1	0	1	1	1	1	1	1	1	0	1
1	1	53	2	158	59.5	-9.50	23.8	102	61	230	85	145.00	5.30	1	1.00	0	1	1	1	1	1	1	1	1	0	1
1	1	62	2	149	54.0	1.40	24.2	149	86	210	59	151.00	5.40	1	1.00	1	0	1	0	1	0	0	1	0	1	1
1	1	84	2	149	59.8	.50	27.0	116	70	234	74	160.00	6.20	1	1.00	1	0	1	1	1	1	1	0	0	0	1
0	0	47	2	158	50.7	-.20	20.3	111	59	153	61	92.00	5.20	1	1.00	1	0	1	0	0	1	1	1	1	1	1
0	0	82	2	149	42.2	.00	18.9	154	81	250	103	147.00	5.40	1	1.00	1	0	1	0	1	1	1	1	0	0	1
1	1	65	2	147	56.2	-.30	25.8	157	86	241	65	176.00	5.20	1	1.00	1	1	1	1	1	0	0	0	0	0	1
1	1	73	2	146	36.2	-1.50	16.9	211	96	190	89	101.00	5.60	1	1.00	1	0	1	1	1	0	1	1	0	1	1
1	1	49	2	155	43.2	1.30	17.9	104	58	184	45	139.00	5.80	0	1.00	1	1	1	1	1	1	1	1	1	1	1
1	1	38	2	152	44.1	.40	19.0	96	63	218	88	130.00	5.20	0	1.00	1	0	0	0	1	1	1	1	1	1	1
0	0	26	2	163	57.4	-2.30	21.7	95	52	189	83	106.00	5.40	1	1.00	1	1	1	0	1	0	1	1	1	1	1
1	1	90	1	158	60.2	-.30	24.1	110	63	164	57	107.00	5.40	1	1.00	1	1	1	1	1	1	1	1	0	1	1
0	0	59	2	161	55.2	1.50	21.3	157	98	293	94	199.00	6.20	1	1.00	0	0	1	0	0	0	0	1	0	0	1
0	0	81	2	136	53.6	-.40	29.0	133	74	208	50	158.00	5.50	1	1.00	1	0	1	0	1	0	0	0	0	1	1
1	1	52	2	152	66.1	1.10	28.6	140	74	247	54	193.00	6.20	1	1.00	1	0	1	1	1	0	0	0	0	0	1
0	0	62	1	164	57.3	-1.20	21.2	158	96	209	94	115.00	5.60	0	1.00	0	0	1	0	1	1	1	1	0	1	1
1	1	46	2	156	51.6	.70	21.2	170	103	193	91	102.00	4.70	0	1.00	0	0	0	0	0	0	1	1	0	1	1
0	0	49	2	157	49.1	3.70	19.8	118	72	292	86	206.00	5.50	1	1.00	1	1	0	1	1	1	1	1	1	0	1
1	1	28	1	176	85.4	-5.30	27.6	133	77	201	54	147.00	5.40	0	1.00	1	1	1	0	1	0	1	0	1	1	1
0	0	61	2	156	59.5	3.60	24.5	121	66	254	73	181.00	5.60	1	1.00	1	1	1	0	0	0	1	1	1	0	1
0	0	51	2	148	47.7	-.30	21.7	84	60	200	66	134.00	5.50	1	1.00	1	0	1	1	1	0	0	1	1	1	1
1	1	62	2	146	64.0	2.70	30.0	119	61	342	66	276.00	5.60	1	1.00	1	0	1	1	0	0	1	0	1	0	1
0	0	73	1	163	77.8	-4.10	29.3	121	75	190	44	146.00	5.40	0	1.00	1	0	1	0	0	1	1	0	0	1	1
1	1	72	1	154	63.3	-1.10	26.8	145	84	214	68	146.00	5.80	0	1.00	0	1	1	1	1	1	1	0	0	1	1
1	1	51	2	156	51.5	2.40	21.2	110	73	257	83	174.00	5.50	1	1.00	1	0	1	1	0	1	1	1	1	0	1
1	1	69	1	173	89.0	-.70	29.7	138	73	214	57	157.00	5.40	1	1.00	0	0	1	1	1	1	1	0	0	1	1
1	1	64	2	142	46.0	-.20	22.8	132	76	263	101	162.00	5.20	1	1.00	1	0	1	0	1	0	0	1	0	0	1

1	1	42	2	151	53.6	-1.30	23.4	98	66	189	91	98.00	5.40	1	1.00	1	1	0	0	1	1	1	1	1	1	1
0	0	71	2	147	74.4	-.10	34.2	150	78	184	62	122.00	6.50	1	1.00	1	1	1	1	1	1	1	0	0	1	0
1	1	74	2	143	39.9	-.40	19.5	157	81	231	43	188.00	5.50	1	1.00	1	1	1	1	1	0	1	1	0	0	1
0	0	68	2	148	68.5	-.70	31.3	127	77	153	46	107.00	4.90	1	1.00	1	0	1	0	1	1	1	0	0	0	1
0	0	63	2	149	56.3	1.50	25.5	151	99	202	54	148.00	6.00	1	1.00	1	0	1	0	0	1	1	0	0	1	1
0	0	25	2	161	72.2	1.60	27.8	110	61	194	52	142.00	5.30	0	1.00	1	1	0	0	1	1	1	0	1	1	1
1	1	79	1	150	64.0	.40	28.4	130	77	146	75	71.00	5.40	1	1.00	0	1	1	1	1	1	1	0	0	1	1
1	1	64	2	159	66.3	-.30	26.2	126	78	148	65	83.00	5.80	1	1.00	1	0	1	0	1	0	0	0	0	1	1
1	1	59	1	174	74.7	-1.60	24.7	138	78	205	57	148.00	5.80	0	1.00	0	1	1	0	0	1	1	1	0	1	1
1	1	67	2	142	54.5	-.70	26.8	134	70	254	51	203.00	6.00	1	1.00	1	0	1	1	1	1	1	0	1	0	1
1	1	60	2	149	51.3	.10	23.1	140	86	229	76	153.00	5.40	1	1.00	1	1	1	0	1	0	1	1	0	0	1
0	0	65	1	170	61.6	3.10	21.3	113	80	239	74	165.00	5.70	1	1.00	1	0	1	0	1	0	0	1	1	0	1
0	0	35	1	165	63.5	1.90	23.4	127	76	228	54	174.00	5.50	0	1.00	0	1	1	1	1	0	1	1	1	0	1
0	0	49	2	158	49.9	2.60	19.9	113	66	171	76	95.00	4.70	1	1.00	1	1	1	0	0	1	0	1	1	1	1
0	0	33	1	161	108.2	-4.90	41.6	153	93	238	60	178.00	8.00	0	1.00	1	0	0	0	1	1	1	0	0	0	0
0	0	58	2	154	62.0	1.70	26.2	106	69	215	66	149.00	5.50	1	1.00	1	0	1	1	1	0	1	0	0	1	1
0	0	67	1	159	65.0	1.00	25.7	132	76	208	51	157.00	7.30	1	1.00	0	1	1	0	1	0	0	0	0	1	0
0	0	84	2	145	47.2	-.10	22.3	110	59	214	79	135.00	5.50	0	1.00	1	0	1	1	1	1	1	1	0	1	1
1	1	88	2	140	40.0	-1.70	20.4	151	78	150	42	108.00	5.20	1	1.00	1	0	1	1	1	1	1	1	0	1	1
1	1	74	1	167	66.2	.40	23.7	175	93	197	124	73.00	6.60	1	1.00	0	1	1	1	1	0	0	1	0	0	0
1	1	65	1	159	61.9	-1.70	24.3	132	75	240	53	187.00	5.20	0	1.00	0	1	1	1	1	1	1	1	0	0	1
1	1	76	1	150	61.3	1.40	27.0	137	65	177	66	111.00	5.60	1	1.00	1	0	1	1	1	1	1	0	0	1	1
1	1	57	2	156	64.9	.40	26.8	104	69	209	65	144.00	5.80	1	1.00	1	0	1	0	1	0	0	0	1	1	1
1	1	81	1	161	61.4	.70	23.7	161	84	221	73	148.00	5.70	1	1.00	0	0	1	0	1	1	1	1	0	0	1
1	1	61	2	151	57.8	2.70	25.3	116	70	185	56	129.00	5.10	1	1.00	1	0	1	1	0	1	1	0	1	1	1
1	1	87	2	142	43.5	2.00	21.4	158	73	166	66	100.00	5.60	1	1.00	1	0	1	1	1	1	1	1	0	1	1
1	1	37	1	179	73.0	3.20	22.7	111	66	198	47	151.00	5.30	0	1.00	0	1	1	0	1	0	1	1	1	1	1
1	1	74	1	171	76.9	-.40	26.3	129	72	201	56	145.00	5.50	1	1.00	0	1	1	1	1	1	1	0	0	1	1
0	0	82	1	162	60.5	-1.40	22.9	141	72	196	48	148.00	7.00	1	1.00	0	0	1	0	1	1	1	1	0	1	0
1	1	71	1	166	54.0	-1.50	19.5	147	82	156	68	88.00	5.00	1	1.00	0	0	1	1	1	1	1	1	0	1	1
1	1	62	1	175	93.0	-4.80	30.2	145	81	152	37	115.00	8.20	1	1.00	1	0	1	0	1	0	1	0	0	0	0
1	1	56	2	159	43.9	-.10	17.3	97	53	205	81	124.00	5.10	1	1.00	1	0	1	0	1	0	0	1	1	1	1
1	1	71	2	152	53.7	1.00	23.3	129	70	171	110	61.00	6.00	1	1.00	0	1	1	1	1	0	1	1	0	1	1
1	1	63	2	151	59.4	1.70	26.0	146	87	220	67	153.00	5.30	1	1.00	1	0	1	0	1	0	1	0	0	0	1
0	0	64	2	151	71.5	1.00	31.2	127	80	246	87	159.00	5.60	1	1.00	1	0	1	1	1	1	0	0	1	0	1
0	0	66	2	146	33.5	-.40	15.7	116	67	203	96	107.00	5.80	1	1.00	1	0	1	0	1	1	0	1	1	1	1
0	1	85	2	139	59.3	-2.30	30.6	142	75	206	55	151.00	5.50	1	1.00	1	0	1	0	1	0	1	0	0	1	1
1	1	66	1	161	63.5	1.40	24.5	144	86	173	61	112.00	5.30	0	1.00	1	0	1	1	1	1	1	1	0	1	1
1	1	78	1	153	46.2	.80	19.7	148	77	184	66	118.00	5.20	1	1.00	1	0	1	1	1	1	1	1	0	1	1

1	1	67	1	163	66.4	-1.50	24.9	146	85	155	34	121.00	5.20	0	1.00	0	0	1	1	1	1	1	1	0	0	1
1	1	58	2	153	63.4	-1.30	27.0	107	73	250	58	192.00	5.20	1	1.00	1	1	1	0	0	1	1	0	1	0	1
0	0	39	1	168	118.8	.10	42.1	145	85	173	38	135.00	5.10	1	1.00	0	0	1	0	1	0	0	0	0	0	1
0	0	43	1	165	60.4	-2.10	22.0	99	70	213	51	162.00	5.50	1	1.00	1	0	1	0	0	0	0	1	1	1	1
1	1	63	2	148	44.0	-1.10	19.9	163	95	215	71	144.00	5.20	1	1.00	1	0	1	1	1	0	0	1	0	1	1
1	1	71	2	146	38.2	-.90	17.9	161	93	245	52	193.00	5.90	1	1.00	1	1	1	1	1	0	1	1	0	0	1
1	1	77	2	155	54.4	.90	22.7	162	101	167	69	98.00	5.60	1	1.00	1	0	1	1	1	0	1	1	0	1	1
0	0	62	2	150	53.4	-2.50	23.8	116	71	288	101	187.00	5.80	1	1.00	1	0	1	0	0	0	1	1	1	0	1
0	0	71	2	152	37.8	-.80	16.2	102	68	216	97	119.00	5.80	1	1.00	1	0	1	0	1	0	0	1	1	1	1
0	0	61	2	151	63.7	2.50	28.0	124	76	237	64	173.00	5.50	1	1.00	1	0	1	1	0	1	0	0	1	0	1
0	0	44	1	181	73.5	-1.50	22.5	152	84	224	76	148.00	5.10	1	1.00	0	0	1	0	1	1	1	1	0	0	1
0	0	72	2	145	50.2	-1.00	23.9	118	66	189	36	153.00	5.90	1	1.00	1	0	1	1	1	0	0	1	0	0	0
0	0	48	1	167	63.9	1.30	22.9	145	97	229	58	171.00	6.40	0	1.00	0	0	1	1	1	0	0	1	0	0	1
0	0	27	2	159	54.3	-.10	21.5	95	52	188	72	116.00	4.90	1	1.00	1	1	1	0	1	0	0	1	1	1	1
0	0	42	2	156	62.1	-1.90	25.5	150	86	233	48	185.00	5.20	1	1.00	1	1	1	0	0	1	1	0	0	0	1
1	1	76	2	150	42.5	1.30	18.9	102	51	198	42	156.00	5.90	1	1.00	1	0	1	1	1	1	1	1	0	1	1
1	1	33	1	165	60.3	-1.50	22.0	99	65	195	65	130.00	5.40	1	1.00	1	1	1	0	1	1	1	1	1	1	1
1	1	74	2	149	42.9	-.50	19.2	127	76	235	111	124.00	5.80	1	1.00	1	1	1	1	1	1	1	1	0	0	1
1	1	59	2	150	49.7	-.20	22.0	113	58	253	80	173.00	5.30	0	2.00	1	0	1	0	0	0	0	1	1	0	1
0	0	60	1	160	67.3	-2.40	26.2	137	87	216	40	176.00	5.70	0	1.00	0	1	1	1	1	1	1	0	0	1	1
1	1	58	2	167	48.1	.80	17.2	108	81	249	66	183.00	5.40	0	1.00	0	0	0	0	0	1	0	1	1	0	1
1	1	73	1	165	69.9	-.10	25.8	134	68	204	36	168.00	5.20	0	1.00	1	0	1	0	1	1	1	0	0	0	1
1	1	67	2	149	61.5	-.60	27.7	119	65	304	72	232.00	5.70	1	1.00	1	0	1	1	1	1	1	0	1	0	1
1	1	62	2	147	53.2	-1.60	24.7	185	96	139	53	86.00	5.50	1	1.00	1	0	1	1	0	1	1	1	0	1	1
1	1	66	2	144	63.9	1.40	30.7	125	71	167	52	115.00	5.70	1	1.00	1	1	1	1	1	1	1	0	0	1	1
1	1	32	2	156	68.4	-.80	28.1	115	69	189	62	127.00	5.80	1	1.00	0	0	1	0	0	0	1	0	1	0	1
0	0	77	2	147	57.9	-.60	26.7	119	62	202	52	150.00	5.90	1	1.00	1	0	1	1	1	1	1	0	0	1	1
1	1	58	2	154	55.4	2.30	23.2	133	85	200	50	150.00	5.70	1	1.00	1	1	1	1	1	1	1	1	1	1	1
1	1	68	1	158	65.1	-.40	26.2	119	61	267	95	172.00	5.70	1	1.00	0	1	1	1	1	1	1	0	0	0	1
1	1	77	2	137	37.6	.20	19.9	121	65	160	51	109.00	5.20	1	1.00	1	1	1	1	1	1	1	1	0	1	1
1	1	61	1	173	64.2	-.80	21.5	114	72	190	43	147.00	5.60	0	1.00	1	0	1	1	0	0	0	1	1	1	1
1	1	67	2	151	53.5	-.10	23.3	123	71	232	37	195.00	5.40	1	1.00	1	0	1	0	1	0	1	1	1	0	1
1	1	82	1	159	66.3	-1.40	26.2	105	66	119	44	75.00	6.90	1	1.00	1	0	1	0	1	1	1	0	0	1	0
1	1	61	2	144	51.6	.90	24.9	126	75	256	52	204.00	5.80	1	1.00	1	1	1	1	1	1	1	1	1	0	1
1	1	76	2	154	60.6	-4.50	25.7	122	71	201	49	152.00	5.40	1	1.00	1	1	1	0	1	0	0	0	1	1	1
1	1	75	1	162	65.4	3.90	25.0	138	84	165	35	130.00	5.40	0	2.00	1	1	1	1	1	1	0	0	0	0	1
1	1	79	2	146	46.4	-1.00	21.9	117	72	215	87	128.00	5.20	1	1.00	1	0	1	0	0	1	1	1	1	1	1
1	1	68	2	143	53.5	3.40	26.0	135	79	212	84	128.00	5.40	1	1.00	1	0	1	0	1	0	0	0	1	0	1
1	1	62	2	154	63.1	.70	26.6	120	70	201	58	143.00	5.60	1	1.00	1	0	1	1	1	1	0	0	0	1	1

1	1	75	2	149	59.7	.30	26.8	163	82	199	91	108.00	5.70	1	1.00	1	1	1	1	1	0	0	0	0	1	1
1	1	62	2	150	58.8	1.00	26.3	123	72	189	68	121.00	5.80	1	1.00	1	0	1	1	1	0	1	0	1	1	1
1	1	25	1	159	55.8	-1.60	22.0	115	57	168	69	99.00	5.50	1	1.00	0	0	0	0	1	1	1	1	1	1	1
0	0	61	2	153	74.8	.50	31.8	136	80	134	53	81.00	5.70	1	1.00	1	1	1	0	0	0	0	0	0	1	1
1	1	80	2	154	59.7	1.60	25.1	128	67	188	61	127.00	5.70	1	1.00	1	0	0	0	1	0	0	0	1	1	1
1	1	76	1	162	76.8	.70	29.4	111	64	131	32	99.00	5.80	1	1.00	1	1	1	0	1	1	1	0	1	0	1
0	0	72	1	151	54.7	-.30	23.9	125	71	218	43	175.00	6.00	1	1.00	0	0	1	0	1	1	1	1	0	1	0
1	1	48	2	160	48.5	-.50	19.0	101	60	173	41	132.00	5.10	1	1.00	0	0	1	1	1	1	1	1	1	1	1
0	0	43	2	148	63.2	-.10	28.8	154	102	205	37	168.00	5.50	1	1.00	1	1	1	1	1	1	0	0	0	0	1
1	1	81	2	143	57.1	-2.10	28.1	142	76	170	67	103.00	5.70	1	1.00	1	0	1	1	1	0	1	0	0	0	0
1	1	61	2	148	50.9	-1.70	23.1	133	68	201	72	129.00	5.50	1	1.00	1	0	1	1	0	0	0	1	1	1	1
1	1	64	2	146	58.3	1.10	27.3	138	61	203	52	151.00	5.60	1	1.00	1	1	1	0	1	0	1	0	0	0	1
1	1	55	2	148	66.7	1.90	30.3	145	90	294	77	217.00	6.20	1	1.00	1	1	1	1	0	1	1	0	0	0	1
0	1	71	1	156	49.1	-2.10	20.3	129	76	162	73	89.00	5.20	1	1.00	0	0	1	0	0	1	0	1	1	1	1
1	1	52	2	162	66.1	1.00	25.1	126	80	245	54	191.00	5.50	1	1.00	1	1	1	1	1	1	1	0	0	0	1
1	1	65	2	154	60.0	1.30	25.2	142	89	216	59	157.00	5.60	1	1.00	1	0	1	1	1	1	1	0	0	0	1
0	0	72	2	154	60.0	1.20	25.3	137	84	177	50	127.00	5.40	1	1.00	1	1	1	0	1	1	1	0	1	0	1
1	1	60	2	162	46.0	.20	17.5	140	92	170	84	86.00	5.20	0	1.00	0	0	0	1	1	0	0	1	0	1	0
1	1	66	1	175	66.2	-.20	21.6	104	62	217	55	162.00	8.00	1	1.00	0	1	1	1	1	1	0	1	1	1	0
1	1	57	2	144	38.6	1.00	18.6	110	59	183	67	116.00	5.30	1	1.00	1	1	1	0	1	1	1	1	1	1	1
1	1	75	2	156	71.0	.00	29.0	112	70	232	39	193.00	5.90	1	1.00	1	1	1	1	1	1	1	0	0	0	1
1	1	79	2	147	64.2	-1.40	29.7	169	89	186	56	130.00	5.80	1	1.00	1	0	1	0	1	0	1	0	0	0	1
0	0	76	2	151	54.8	-1.00	23.9	134	75	161	53	108.00	6.00	1	1.00	1	1	1	0	1	0	0	1	0	0	1
0	0	41	2	164	70.5	1.40	26.3	117	75	241	89	152.00	5.30	1	1.00	1	0	1	0	1	1	0	0	1	0	1
0	0	58	2	147	52.1	3.00	24.0	128	69	246	55	191.00	5.60	1	1.00	1	1	1	0	1	0	0	1	1	0	1
0	0	43	2	162	58.9	.10	22.4	107	68	171	63	108.00	5.10	1	1.00	1	0	1	0	1	1	1	1	1	1	1
0	0	73	1	166	72.1	.10	26.0	152	87	189	48	141.00	5.00	1	1.00	1	1	1	1	1	1	1	0	0	1	1
1	1	64	2	162	55.2	1.60	21.1	122	85	260	63	197.00	5.50	0	1.00	1	0	1	0	1	1	1	1	1	0	1
1	1	63	2	153	68.6	1.50	29.1	134	87	211	38	173.00	5.70	1	1.00	1	1	1	0	0	1	1	0	1	0	1
1	1	64	2	164	57.8	-.10	21.4	104	62	214	42	172.00	5.80	1	1.00	1	0	1	1	0	1	1	1	1	1	1
1	1	78	1	157	60.3	1.70	24.3	182	89	193	57	136.00	5.40	1	1.00	0	0	1	1	0	1	1	1	0	1	1
1	1	78	1	152	55.3	-1.60	23.8	134	82	230	50	180.00	6.00	1	1.00	1	0	1	1	1	1	1	1	1	0	1
1	1	55	2	153	62.7	.00	26.8	116	71	230	80	150.00	5.50	1	1.00	0	0	1	0	0	0	0	0	0	0	1
0	0	80	1	159	60.9	.70	24.0	141	77	181	61	120.00	5.60	0	1.00	0	0	1	0	1	0	1	1	0	1	1
0	0	63	1	162	53.4	.00	20.2	135	86	238	91	147.00	5.20	1	1.00	0	0	1	0	0	0	0	1	1	0	1
0	0	65	1	160	62.6	.80	24.3	114	65	208	35	173.00	5.60	1	1.00	1	1	1	0	1	0	0	1	0	0	1
0	0	67	2	152	51.9	-.80	22.5	124	78	239	70	169.00	5.70	1	1.00	1	1	1	0	0	1	1	1	1	0	1
0	0	61	2	151	59.5	1.10	26.1	171	92	225	37	188.00	5.00	1	1.00	1	0	1	1	0	1	1	0	0	0	1
1	1	66	2	144	64.3	.90	31.1	146	76	258	58	200.00	7.50	1	1.00	0	0	1	1	1	0	1	0	0	0	0

1	1	46	2	150	73.0	-4.80	32.4	152	98	235	38	197.00	7.20	1	1.00	1	0	1	1	0	1	0	0	0	0	0
1	1	69	1	159	49.3	2.30	19.4	156	93	245	109	136.00	5.40	1	1.00	0	0	1	0	1	0	1	1	0	0	1
1	1	70	1	165	59.3	-1.80	21.8	128	83	212	58	154.00	5.50	1	1.00	1	1	1	1	1	1	1	1	0	1	1
1	1	85	1	170	64.0	-3.60	22.1	146	84	169	63	106.00	5.00	1	1.00	0	0	1	1	1	0	1	1	0	1	1
0	0	72	2	148	50.4	-3.20	22.9	102	58	163	53	110.00	5.50	0	1.00	1	1	1	1	1	1	1	1	1	1	1
0	0	65	2	148	42.7	-.60	19.4	110	65	243	83	160.00	7.10	1	1.00	1	0	1	0	1	0	1	1	1	0	0
0	0	71	2	151	48.3	2.40	21.0	125	82	221	60	161.00	5.50	1	1.00	1	0	1	1	1	1	1	1	1	0	1
1	1	75	2	143	43.6	-.60	21.2	125	73	222	67	155.00	5.30	1	1.00	1	0	1	0	1	0	0	1	0	0	1
1	1	72	1	155	56.8	-1.20	23.6	113	73	230	44	186.00	5.70	1	1.00	1	1	1	1	1	1	1	1	1	0	1
1	1	83	2	147	52.0	-.40	24.1	156	79	217	67	150.00	5.60	1	1.00	1	0	1	1	1	0	0	1	0	1	1
1	1	70	2	151	45.9	-.10	20.1	105	68	251	95	156.00	5.40	1	1.00	1	0	1	1	1	1	1	1	0	0	1
1	1	57	1	162	60.7	1.80	23.0	162	91	151	63	88.00	4.60	1	1.00	0	0	1	1	0	0	0	1	0	1	1
0	0	67	1	162	60.1	-2.10	22.8	157	89	199	66	133.00	5.70	1	1.00	0	1	1	0	1	1	1	1	0	1	1
1	1	54	2	161	70.3	.20	27.0	146	85	242	74	168.00	5.40	1	1.00	0	0	1	0	0	0	0	0	0	0	1
0	0	76	2	140	48.1	.10	24.5	122	72	170	82	88.00	6.10	1	1.00	1	0	1	0	1	0	0	1	0	1	1
0	0	57	2	149	60.3	-1.70	27.2	104	71	273	62	211.00	6.30	1	1.00	0	0	1	1	0	0	1	0	1	0	1
0	0	54	2	152	56.4	.80	24.3	105	57	234	52	182.00	5.20	1	1.00	1	1	1	1	1	0	0	1	1	0	1
0	0	61	1	168	74.6	2.70	26.5	155	97	199	51	148.00	6.60	0	1.00	0	1	0	1	1	0	1	0	0	1	0
1	1	75	1	148	45.9	-1.00	21.0	110	60	263	95	168.00	6.80	1	1.00	1	1	1	1	1	1	1	1	1	0	0
0	0	73	1	161	76.9	.10	29.5	144	82	195	47	148.00	6.00	1	1.00	0	0	1	1	1	1	1	0	0	1	0
0	0	83	2	146	50.8	-.10	23.9	149	90	188	64	124.00	4.80	1	1.00	1	0	1	0	1	0	0	1	0	1	1
0	0	66	2	150	71.2	.40	31.5	162	88	215	90	125.00	5.70	1	1.00	1	0	1	1	1	1	1	0	0	1	1
0	0	60	2	143	56.4	-3.90	27.6	135	76	255	60	195.00	5.40	0	1.00	1	1	0	1	0	0	1	0	0	0	1
0	0	31	2	161	51.7	.10	20.0	95	58	274	112	162.00	5.50	1	1.00	1	0	0	1	0	0	0	1	1	0	1
0	0	79	1	160	49.5	.90	19.4	169	88	184	45	139.00	5.50	1	1.00	0	0	1	0	1	0	0	1	0	1	1
0	0	42	1	158	81.5	1.30	32.7	133	85	213	38	175.00	5.60	0	1.00	0	0	1	0	1	1	1	0	1	0	1
0	0	60	2	154	69.8	-2.00	29.3	164	91	303	75	228.00	5.80	1	1.00	1	0	1	1	0	1	1	0	0	0	1
1	1	38	1	173	78.2	-2.80	26.0	144	94	219	41	178.00	5.10	0	1.00	0	1	1	0	0	1	1	0	0	1	1
1	1	74	2	152	55.6	.00	24.0	140	66	206	44	162.00	5.70	1	1.00	1	0	1	0	1	1	1	1	0	1	1
0	0	36	2	152	47.7	-.40	20.5	121	81	198	80	118.00	4.90	0	1.00	1	1	1	1	1	1	1	1	1	1	1
0	0	68	1	161	81.7	-.10	31.6	120	72	242	53	189.00	5.70	1	1.00	0	0	1	1	0	0	0	0	0	0	1
1	1	59	2	160	66.7	3.80	26.0	132	77	260	67	193.00	5.20	1	1.00	1	1	1	1	1	0	0	0	1	0	1
1	1	36	2	162	53.8	-.20	20.4	108	70	164	49	115.00	4.60	0	1.00	1	1	0	0	1	0	0	1	1	1	1
1	1	64	1	172	78.7	-2.30	26.6	154	80	202	66	136.00	5.30	1	1.00	0	1	1	1	1	1	1	0	0	1	1
1	1	83	2	144	46.8	1.10	22.5	140	74	175	64	111.00	5.30	1	1.00	1	1	1	1	1	1	1	1	0	0	1
1	1	63	2	157	62.4	-.40	25.3	110	70	160	64	96.00	5.40	1	1.00	1	0	1	1	1	1	1	0	1	0	1
1	1	64	2	159	61.6	-.20	24.4	102	72	266	52	214.00	5.30	1	1.00	1	1	1	1	1	1	0	1	1	0	1
1	1	76	2	147	62.4	-1.40	28.9	113	68	236	68	168.00	5.70	1	1.00	1	0	1	1	1	1	1	0	0	0	1
1	1	74	1	162	72.1	-1.90	27.6	133	74	121	52	69.00	6.20	1	1.00	0	0	1	1	1	1	1	0	0	1	1

1	1	66	1	166	82.1	-2.70	29.8	151	87	165	52	113.00	5.80	0	1.00	0	0	1	1	1	1	0	0	0	0	1
1	1	80	1	156	55.9	1.70	22.9	153	81	163	38	125.00	4.90	1	1.00	1	0	1	1	1	0	1	1	0	0	1
1	1	71	2	147	38.2	-.10	17.7	149	79	190	75	115.00	6.20	1	1.00	1	0	1	1	1	1	1	1	0	0	1
1	1	64	2	155	49.8	.00	20.7	123	67	248	71	177.00	5.60	1	1.00	1	0	1	1	1	1	1	1	1	0	1
1	1	73	2	148	45.8	-.80	20.8	125	60	170	54	116.00	5.70	1	1.00	1	0	1	0	1	0	0	1	0	0	1
1	1	69	1	176	75.2	2.80	24.2	135	83	165	60	105.00	5.60	1	1.00	0	0	1	1	1	1	1	1	1	1	1
0	0	89	2	133	40.2	.90	22.6	134	74	273	67	206.00	5.60	1	1.00	1	0	1	0	1	0	0	1	0	0	1
0	1	86	1	157	58.5	-1.90	23.6	130	75	205	65	140.00	5.50	1	1.00	1	0	1	0	1	0	1	1	1	1	1
1	1	62	2	158	49.9	-1.70	19.9	168	84	259	92	167.00	5.90	1	1.00	1	0	1	0	1	0	1	1	0	0	1
0	0	52	2	156	54.3	-1.10	22.3	138	89	223	87	136.00	4.90	1	1.00	1	0	1	0	1	0	0	1	1	0	1
0	0	61	2	154	65.7	.60	27.5	120	73	322	55	267.00	5.50	1	1.00	1	0	1	1	0	1	1	0	1	0	1
1	1	74	2	143	57.2	-1.40	28.1	149	82	164	84	80.00	5.20	1	1.00	1	1	1	1	1	1	1	0	0	1	1
0	0	70	1	159	53.3	-.70	21.1	117	66	169	43	126.00	6.60	0	1.00	1	1	1	1	0	1	1	1	1	1	0
0	0	69	2	149	55.4	-4.00	24.9	135	79	175	42	133.00	5.10	1	1.00	1	1	1	0	1	1	1	1	1	1	1
1	1	62	2	153	53.0	-.90	22.7	103	61	160	63	97.00	6.00	1	1.00	1	0	1	1	1	1	1	1	1	1	1
1	1	75	1	169	82.2	-2.90	28.9	152	85	163	65	98.00	7.50	1	1.00	0	0	1	1	1	0	1	0	0	1	0
0	0	69	1	167	84.2	.30	30.3	134	86	180	67	113.00	5.60	1	1.00	0	0	1	0	0	1	1	0	1	1	1
1	1	70	2	141	54.5	3.40	27.3	149	83	184	95	89.00	5.20	1	1.00	1	0	1	0	1	0	1	0	0	1	1
0	0	30	2	160	45.5	.30	17.8	106	69	155	90	65.00	4.80	1	1.00	1	0	1	1	1	1	1	1	1	1	1
0	0	66	1	164	57.6	-2.70	21.3	128	71	142	53	89.00	6.20	1	1.00	1	1	1	0	1	0	1	1	1	0	0
1	1	63	1	165	48.5	-2.40	17.7	142	91	209	54	155.00	5.60	0	1.00	1	1	1	0	1	0	0	1	0	1	1
1	1	57	1	163	73.0	1.90	27.4	123	82	182	30	152.00	7.80	0	1.00	1	0	1	1	1	1	1	0	1	0	0
1	1	44	1	162	59.0	.20	22.3	125	81	260	84	176.00	5.20	1	1.00	0	0	1	0	1	1	1	1	1	0	1
0	0	78	2	143	51.9	3.40	25.3	114	76	259	99	160.00	4.80	1	1.00	1	0	1	1	1	0	1	0	0	0	1
0	0	69	2	151	41.2	.80	18.1	135	80	242	101	141.00	5.20	1	1.00	1	0	1	0	1	0	1	1	1	0	1
0	0	71	2	157	54.3	-1.20	21.9	130	74	259	37	222.00	5.00	1	1.00	1	0	1	1	1	1	1	1	1	0	1
1	1	54	2	157	53.3	1.10	21.5	133	88	219	88	131.00	5.30	1	1.00	1	0	1	1	1	0	1	1	1	1	1
1	1	39	2	151	47.2	-.50	20.5	117	73	136	58	78.00	5.30	1	1.00	1	0	0	0	1	0	0	1	1	1	1
0	0	34	2	161	53.1	1.40	20.3	107	63	191	64	127.00	5.00	1	1.00	1	1	1	1	1	0	0	1	1	1	1
1	1	32	1	174	64.2	-.40	21.2	122	53	148	46	102.00	5.10	0	1.00	1	1	0	1	1	0	1	1	1	1	1
0	0	65	1	167	56.6	.70	20.3	153	93	188	65	123.00	5.40	0	1.00	1	0	1	0	1	1	1	1	0	1	1
0	0	53	1	162	58.1	-1.00	22.2	115	78	194	105	89.00	5.60	0	1.00	0	0	1	1	1	1	1	1	1	1	1
0	0	20	2	156	48.8	-1.60	20.0	113	65	156	79	77.00	5.20	1	1.00	0	1	1	1	1	0	1	1	1	1	1
0	0	54	1	172	67.7	.10	22.8	140	81	209	60	149.00	5.90	1	1.00	0	0	1	0	1	1	0	1	0	1	1
1	1	67	1	170	86.6	.30	29.8	163	101	221	46	175.00	6.10	1	1.00	1	1	1	1	1	1	1	0	0	0	1
1	1	59	1	165	58.9	-.30	21.5	130	73	235	36	199.00	6.00	0	1.00	1	0	1	0	1	0	1	1	1	0	1
1	1	68	1	169	62.4	-.60	21.7	121	61	186	39	147.00	5.80	1	1.00	1	0	1	1	0	1	0	1	0	0	1
1	1	61	1	167	71.6	-1.60	25.5	151	87	234	70	164.00	5.30	1	1.00	0	0	1	1	1	1	1	0	0	0	1
1	1	61	1	166	71.5	.00	26.0	119	79	166	44	122.00	5.60	1	1.00	0	0	1	0	0	0	1	0	1	1	1

1	1	32	2	159	49.5	-1.20	19.6	108	58	171	72	99.00	5.10	1	1.00	1	0	1	1	1	1	1	1	1	1	1
0	0	60	2	159	58.3	-2.10	23.1	129	78	235	57	178.00	5.30	1	1.00	0	1	1	0	1	1	1	1	0	0	1
1	1	53	2	164	56.0	-1.80	20.8	132	82	232	104	128.00	5.30	1	1.00	0	0	1	1	0	1	1	1	1	0	1
1	1	58	2	148	45.8	.20	20.9	121	77	261	87	174.00	5.30	1	1.00	1	0	1	1	1	1	1	1	1	0	1
1	1	62	1	166	58.7	-.20	21.4	143	83	227	77	150.00	5.40	1	1.00	0	1	1	1	1	1	1	1	0	0	1
1	1	60	2	159	55.7	1.30	21.9	121	72	232	55	177.00	6.00	1	1.00	1	1	1	1	1	0	1	1	1	0	1
1	1	26	2	160	44.3	-1.80	17.3	99	50	153	87	66.00	5.50	1	1.00	1	0	1	1	1	0	0	1	1	1	1
1	1	79	1	162	62.3	.40	23.8	151	86	174	60	114.00	5.90	1	1.00	0	1	1	0	1	1	1	1	0	1	1
1	1	70	2	147	56.5	1.10	26.2	160	89	219	47	172.00	5.50	1	1.00	1	1	1	1	1	1	1	0	0	1	1
1	1	63	1	168	58.0	.90	20.5	118	78	195	47	148.00	5.10	0	1.00	0	0	1	0	1	1	1	1	1	1	1
1	1	74	2	148	46.1	.00	21.0	139	79	167	74	93.00	6.20	1	1.00	1	1	1	1	1	1	1	1	1	1	1
1	1	65	2	152	48.8	.60	21.0	121	64	170	57	113.00	5.70	1	1.00	1	1	1	1	1	1	1	1	1	1	1
1	1	83	2	146	62.7	-.30	29.4	119	63	183	55	128.00	7.20	1	1.00	1	1	1	1	1	0	1	0	0	1	0
1	1	66	2	155	74.3	.20	31.1	117	66	172	64	108.00	5.90	1	1.00	1	0	1	1	1	0	0	0	0	0	1
0	0	57	2	153	63.5	1.10	27.1	127	74	210	60	150.00	6.00	1	1.00	1	0	1	1	0	0	0	0	0	1	1
0	0	59	1	164	76.3	.30	28.2	127	87	180	70	110.00	6.50	1	1.00	0	1	1	0	0	1	0	0	0	1	0
1	1	76	2	142	51.1	.40	25.3	143	87	229	56	173.00	5.70	1	1.00	1	0	1	1	0	0	1	0	0	0	1
1	1	69	1	140	49.1	.50	24.9	121	63	189	68	121.00	5.70	1	1.00	1	0	1	1	1	1	1	1	0	1	1
1	1	61	2	158	72.3	-2.10	29.1	98	51	208	43	165.00	6.60	1	1.00	1	1	1	0	0	0	0	0	0	1	0
1	1	73	1	166	60.8	-1.60	21.9	116	65	174	53	121.00	5.90	0	1.00	0	0	1	0	1	1	1	1	1	1	1
1	1	68	2	153	50.3	-1.10	21.4	135	80	259	107	152.00	5.30	1	1.00	0	1	1	0	1	1	1	1	0	0	1
0	0	68	1	162	68.6	-2.20	26.0	98	59	207	78	129.00	5.30	1	1.00	1	1	1	0	0	0	0	0	1	1	1
1	1	65	1	175	79.8	-3.50	26.0	157	81	218	66	152.00	5.60	0	1.00	0	0	1	1	1	1	1	0	0	1	1
0	0	69	2	149	47.8	2.60	21.5	156	97	213	50	163.00	5.60	1	1.00	1	0	1	0	0	0	0	1	0	1	1
1	1	80	2	141	48.0	-2.20	24.3	130	83	195	91	104.00	5.70	1	1.00	1	0	1	0	1	1	1	1	0	0	1
1	1	70	2	159	56.4	-.10	22.4	148	74	198	74	124.00	5.00	1	1.00	1	0	1	0	1	0	1	1	0	1	1
1	1	66	2	152	58.2	-1.60	25.2	149	83	164	61	103.00	5.40	1	1.00	1	1	1	1	1	0	1	0	0	0	1
0	0	72	2	152	52.0	.40	22.5	126	71	266	71	195.00	5.10	1	1.00	1	1	1	0	1	1	1	1	1	0	1
0	0	32	2	160	51.4	.60	20.0	96	65	183	75	108.00	5.00	1	1.00	0	0	1	0	0	0	1	1	1	1	1
1	1	63	2	155	51.9	-.40	21.6	129	83	129	54	75.00	5.70	1	1.00	1	1	1	0	1	1	1	1	1	1	1
1	1	61	2	152	53.9	.10	23.3	123	78	192	72	120.00	5.60	1	1.00	1	0	1	0	0	0	0	1	1	0	1
1	1	64	2	159	53.4	2.90	21.1	147	81	192	87	105.00	5.70	0	2.00	1	0	1	1	1	0	0	1	0	1	1
0	0	62	2	154	53.4	1.80	22.4	105	62	175	64	111.00	5.50	1	1.00	1	0	1	0	1	0	0	1	0	1	1
0	0	73	2	146	53.4	1.70	25.0	141	80	179	68	111.00	5.30	1	1.00	1	1	1	0	1	0	1	0	0	0	1
0	0	46	2	151	42.8	.90	18.7	118	59	247	97	150.00	5.20	1	1.00	0	1	1	0	1	0	1	1	1	0	1
0	0	43	1	168	59.7	-1.30	21.2	126	76	208	73	135.00	5.40	1	1.00	0	1	1	0	1	1	1	1	1	1	1
0	0	65	1	170	78.8	-1.00	27.2	141	95	173	61	112.00	5.00	1	1.00	0	0	1	1	0	0	1	0	0	0	1
0	0	68	2	151	58.8	1.00	25.8	177	96	204	87	117.00	5.30	1	1.00	0	0	1	0	1	0	0	0	0	1	1
0	0	66	1	154	57.9	.50	24.3	148	67	181	36	145.00	5.20	0	1.00	1	0	0	0	1	0	0	1	0	0	1

1	1	55	1	163	65.2	-1.20	24.6	121	69	233	51	182.00	5.50	1	1.00	0	0	1	0	1	0	1	1	1	0	1
1	1	69	1	163	67.0	2.20	25.3	144	93	210	72	138.00	5.80	1	1.00	1	0	1	1	0	1	1	0	0	1	1
1	1	44	2	165	77.8	4.10	28.7	159	110	213	62	151.00	5.00	0	1.00	0	0	1	0	0	0	1	0	0	1	1
1	1	62	1	160	64.1	-1.90	25.1	142	78	215	57	158.00	6.20	1	1.00	1	1	1	1	1	1	1	0	0	0	1
0	0	37	1	173	76.2	-.40	25.3	111	66	187	42	145.00	5.30	0	1.00	0	1	1	0	1	1	1	0	1	1	1
0	0	40	2	161	51.9	-.50	19.9	106	66	190	50	140.00	5.10	1	1.00	1	1	1	1	1	1	0	1	1	1	1
1	1	42	1	159	68.7	-1.90	27.3	127	90	183	79	104.00	6.40	1	1.00	0	1	1	1	1	0	1	0	0	1	1
1	1	68	1	164	67.7	-2.80	25.0	129	77	251	59	192.00	6.10	1	1.00	1	1	1	0	1	0	0	0	1	0	1
1	1	35	2	157	56.5	2.70	22.8	124	70	171	74	97.00	5.10	1	1.00	1	0	1	1	1	1	1	1	1	1	1
1	1	67	2	145	41.8	-1.30	20.0	118	66	250	80	170.00	5.40	1	1.00	1	0	1	1	1	1	1	1	1	0	1
1	1	63	2	151	52.4	-.50	23.1	105	66	225	67	158.00	4.90	1	1.00	1	1	1	1	0	1	1	1	0	0	1
0	0	67	1	167	64.7	-.20	23.1	153	82	188	71	117.00	5.80	1	1.00	0	0	1	0	1	1	1	1	0	1	0
1	1	73	1	162	75.5	-.20	28.9	137	73	227	58	169.00	5.60	1	1.00	1	1	1	1	1	1	1	0	1	0	1
1	1	66	2	143	43.1	.10	21.0	121	66	206	59	147.00	5.50	1	1.00	0	0	1	1	1	1	0	1	1	1	1
1	1	54	2	158	57.5	-.70	23.0	115	72	192	79	113.00	5.20	1	1.00	1	1	1	1	1	1	1	1	1	1	1
1	1	44	1	167	55.5	-.40	19.9	149	79	176	90	86.00	5.60	0	1.00	0	0	1	1	1	0	1	1	0	1	1
1	1	66	1	174	86.6	-6.30	28.5	134	90	257	31	226.00	5.60	1	1.00	1	0	1	1	1	1	1	0	0	0	1
1	1	70	1	159	63.1	-.40	24.8	112	63	205	59	146.00	6.00	1	1.00	1	0	1	0	1	1	1	1	1	1	1
1	1	43	2	156	51.5	1.20	21.1	103	61	220	59	161.00	5.00	1	1.00	1	1	1	1	1	1	1	1	1	0	1
0	0	44	2	151	57.0	3.50	25.0	115	64	180	73	107.00	5.60	1	1.00	1	1	1	1	1	0	1	0	1	1	1
1	1	44	1	162	60.3	-1.30	22.8	126	76	167	51	116.00	5.00	1	1.00	0	1	1	1	1	0	1	1	1	1	1
0	0	71	1	171	58.3	1.20	19.9	155	83	184	75	109.00	5.00	1	1.00	0	0	1	1	1	1	1	1	0	1	1
0	0	61	2	150	59.8	.90	26.7	152	89	281	57	224.00	5.60	1	1.00	1	0	1	0	1	1	1	0	0	0	1
0	0	65	2	150	54.0	.70	24.0	148	94	233	91	142.00	5.60	1	1.00	1	0	1	1	1	0	0	1	0	0	1
0	0	68	2	145	49.6	-1.10	23.5	118	79	244	52	192.00	5.80	1	1.00	0	1	1	0	1	0	0	1	0	0	1
0	0	71	2	147	38.7	.60	17.8	91	50	226	131	95.00	6.00	1	1.00	1	0	1	0	1	1	0	1	1	0	1
0	0	70	1	159	62.0	-1.10	24.4	165	90	222	50	172.00	6.00	1	1.00	0	0	1	0	1	1	1	1	0	0	1
0	0	67	2	149	53.3	-4.80	24.0	137	87	216	62	154.00	5.80	1	1.00	1	0	1	0	0	0	0	1	1	1	1
1	1	73	2	143	46.0	.20	22.3	136	70	218	48	170.00	5.40	1	1.00	1	1	1	1	1	1	0	1	0	1	1
1	1	53	1	172	73.8	-.70	25.0	140	85	266	48	218.00	6.00	1	1.00	0	1	1	0	1	1	1	0	0	0	1
1	1	82	2	149	54.4	.70	24.3	151	82	218	86	132.00	5.30	1	1.00	1	0	1	1	1	1	1	1	0	0	1
1	1	55	2	148	47.5	.60	21.6	127	68	270	111	159.00	5.10	1	1.00	1	1	1	1	1	1	1	1	1	0	1
1	1	57	2	152	56.3	-.90	24.2	156	84	246	70	176.00	5.60	1	1.00	0	1	1	1	0	1	1	1	0	0	1
1	1	81	1	155	52.1	-1.60	21.7	195	106	191	77	114.00	4.80	1	1.00	1	0	1	0	1	1	1	1	0	1	1
1	1	70	1	165	81.8	.00	30.1	140	78	153	53	100.00	5.80	1	1.00	0	1	1	0	1	0	1	0	0	1	1
1	1	65	2	145	49.3	1.00	23.5	122	71	240	41	199.00	6.10	1	1.00	1	0	1	1	1	0	1	1	1	0	1
0	0	77	2	137	44.6	-2.30	23.7	138	84	122	37	85.00	5.50	1	1.00	1	0	1	1	1	0	1	1	1	0	1
0	0	62	1	167	80.1	-3.90	28.8	109	70	161	46	115.00	5.70	0	1.00	1	0	0	1	1	1	1	0	1	1	1
1	1	65	2	144	52.7	.10	25.2	125	85	196	52	144.00	5.30	1	1.00	1	0	1	1	1	1	1	0	0	1	1

1	1	74	1	164	65.3	-.60	24.3	130	76	207	38	169.00	6.20	0	1.00	0	0	1	1	1	1	1	1	0	0	1
1	1	55	2	157	66.5	7.70	27.0	130	81	232	57	175.00	5.70	1	1.00	1	0	1	0	1	0	1	0	1	0	1
1	1	65	1	174	78.0	3.30	25.8	164	103	207	75	132.00	5.60	1	1.00	0	1	1	0	1	1	1	0	0	1	1
1	1	31	2	158	52.0	.80	20.7	99	51	184	48	136.00	5.10	0	1.00	1	0	1	0	0	0	0	1	1	1	1
1	1	71	2	148	47.5	-1.70	21.6	124	69	186	90	96.00	5.20	1	1.00	0	0	1	1	1	0	0	1	1	1	1
1	1	66	2	152	57.0	2.10	24.8	143	77	174	73	101.00	5.10	1	1.00	1	0	1	0	1	1	1	1	0	1	1
1	1	74	1	165	55.2	-.80	20.2	161	81	228	93	135.00	5.90	1	1.00	1	0	1	1	1	1	1	1	0	0	1
1	1	65	1	172	71.3	-1.40	23.9	130	78	205	57	148.00	5.30	1	1.00	1	0	1	1	1	1	1	1	0	1	1
1	1	53	2	153	49.5	1.60	21.1	126	69	157	50	107.00	5.40	1	1.00	1	0	1	0	1	0	0	1	1	0	1
1	1	81	1	161	55.4	-1.20	21.4	147	75	136	45	91.00	5.90	0	1.00	0	1	1	0	1	0	1	1	0	0	1
0	0	68	1	169	60.6	-.80	21.1	144	88	136	52	84.00	6.20	0	1.00	0	0	1	1	1	1	1	1	0	1	1
0	1	56	2	143	43.2	-1.10	21.1	126	75	229	96	133.00	5.20	1	1.00	1	0	1	0	1	0	0	1	1	0	1
0	0	68	2	142	42.9	-1.00	21.2	111	68	153	67	86.00	5.30	1	1.00	1	0	1	1	1	1	1	1	1	1	1
1	1	76	2	147	65.2	-.80	30.2	136	63	178	52	126.00	6.10	1	1.00	1	1	1	0	1	1	1	0	0	1	1
0	0	73	2	149	51.6	.40	23.1	126	67	204	64	140.00	5.60	1	1.00	1	1	1	1	1	0	1	1	0	1	1
1	1	80	2	151	51.2	-2.70	22.5	136	74	180	44	136.00	6.10	1	1.00	1	0	1	1	1	0	0	1	0	1	1
1	1	62	1	168	57.1	1.40	20.1	127	85	164	70	94.00	4.70	1	1.00	0	1	1	0	0	0	0	1	1	1	1
0	0	72	2	148	76.4	-1.10	34.7	149	81	203	83	120.00	8.10	1	1.00	1	0	1	0	0	1	0	0	0	0	0
1	1	62	1	166	74.2	-3.40	26.9	153	98	190	40	150.00	5.80	1	1.00	0	0	1	1	0	0	0	0	0	1	1
0	0	54	1	163	67.0	.70	25.3	128	89	182	52	130.00	5.20	1	1.00	0	0	1	0	1	1	1	0	1	1	1
0	0	53	2	157	48.5	.80	19.8	137	88	236	64	172.00	5.60	0	1.00	1	1	0	1	0	0	1	1	1	0	1
0	0	58	2	148	57.0	1.20	26.0	144	79	172	64	108.00	5.20	1	1.00	1	0	1	1	1	1	1	0	0	1	1
0	0	76	2	145	48.4	1.30	22.8	144	63	215	67	148.00	6.40	1	1.00	1	1	1	1	1	1	1	1	0	1	1
0	0	69	1	167	66.0	-.80	23.5	150	84	149	61	88.00	5.10	0	1.00	0	0	1	1	1	1	1	1	0	1	1
0	0	59	2	156	67.4	1.10	27.8	128	73	227	49	178.00	5.20	0	1.00	1	0	0	0	0	0	1	0	0	0	1
0	0	76	2	146	52.4	.60	24.4	124	67	270	84	186.00	5.40	1	1.00	1	0	1	1	1	0	0	1	1	0	1
0	0	74	2	149	58.2	.70	26.1	153	79	195	61	134.00	5.60	1	1.00	1	0	1	0	1	0	1	0	0	1	1
0	0	31	2	165	55.1	-1.70	20.1	96	54	175	87	88.00	5.30	1	1.00	1	0	1	1	0	0	1	1	1	1	1
0	1	61	2	150	62.7	-.40	28.0	182	98	243	68	175.00	5.70	1	1.00	0	1	1	0	1	1	1	0	0	0	1
1	1	39	2	166	55.9	-1.30	20.2	125	74	226	68	158.00	5.10	1	1.00	1	1	1	1	1	1	1	1	1	0	1
0	0	67	2	154	60.6	1.70	25.5	132	87	210	69	141.00	5.50	1	1.00	0	1	1	1	1	0	0	0	1	1	1
1	1	32	2	165	58.5	1.10	21.5	106	62	212	90	122.00	5.20	1	1.00	1	0	1	1	1	1	1	1	1	1	1
1	1	76	2	147	61.1	1.40	28.2	142	74	145	60	85.00	5.60	1	1.00	1	1	1	1	1	1	0	0	0	1	1
1	1	67	1	167	70.8	2.90	25.2	142	87	215	50	165.00	5.60	0	1.00	1	0	1	1	1	1	1	0	0	1	1
1	1	61	2	147	61.0	-3.70	28.1	106	64	177	62	115.00	5.30	0	1.00	1	0	1	0	0	0	1	0	1	1	1
1	1	48	2	168	69.2	1.40	24.6	114	65	183	71	112.00	5.50	1	1.00	1	1	1	1	1	1	0	1	1	1	1
1	1	68	1	170	71.6	-.50	24.8	146	99	221	58	163.00	7.70	1	1.00	0	0	1	1	1	1	1	1	0	0	0
1	1	53	2	152	75.3	-1.00	32.5	127	75	133	50	83.00	5.30	1	1.00	1	1	1	0	1	0	0	0	1	1	1
1	1	26	2	153	61.1	.90	26.1	104	62	160	51	109.00	5.10	1	1.00	1	1	1	1	1	0	1	0	1	1	1

1	1	64	2	140	42.1	-.70	21.4	113	73	181	58	123.00	6.00	1	1.00	1	1	1	1	1	1	1	1	1	1	1
1	1	32	1	173	70.0	2.50	23.4	118	69	211	68	143.00	5.10	1	1.00	1	0	0	0	1	0	0	1	1	1	1
0	0	63	2	152	52.4	2.50	22.7	140	79	209	73	136.00	5.10	1	1.00	1	0	1	1	0	1	0	1	0	1	1
1	1	54	1	181	65.9	.60	20.1	127	89	159	49	110.00	4.80	0	1.00	1	0	1	0	1	0	0	1	0	1	1
1	1	79	1	163	75.6	-.80	28.4	138	67	176	50	126.00	5.30	0	1.00	0	0	1	1	1	1	1	0	0	1	1
1	1	74	2	146	53.8	-.60	25.1	107	61	179	49	130.00	5.20	1	1.00	1	0	1	1	1	0	0	0	0	1	1
0	0	78	2	145	50.9	.40	24.2	92	54	205	77	128.00	6.00	1	1.00	1	1	1	0	1	0	1	1	1	1	1
1	1	78	1	163	65.3	.10	24.5	137	79	163	53	110.00	5.30	1	1.00	0	0	1	1	1	1	1	1	0	1	1
0	0	66	1	161	56.4	-.60	21.6	103	59	146	53	93.00	7.40	0	1.00	1	0	0	0	1	1	1	1	0	0	0
1	1	69	2	149	46.8	-.90	21.0	122	70	216	62	154.00	5.70	1	1.00	1	0	1	1	0	1	0	1	1	1	1
0	0	65	2	145	46.9	.80	22.2	106	71	212	66	146.00	5.40	1	1.00	1	1	1	0	1	1	0	1	1	1	1
0	0	75	2	150	70.3	.00	31.2	152	84	166	30	136.00	5.30	1	1.00	1	1	1	1	1	1	1	0	0	0	1
1	1	59	2	149	73.9	1.40	33.3	121	67	228	46	182.00	5.50	1	1.00	1	0	1	1	0	1	1	0	1	0	1
1	1	68	1	169	67.9	-.40	23.8	142	78	166	66	100.00	6.30	1	1.00	0	1	1	1	1	1	1	1	0	1	0
0	0	60	2	153	59.9	1.50	25.5	109	71	174	43	131.00	5.20	1	1.00	1	0	1	0	1	0	0	0	1	1	1
1	1	61	2	161	50.4	.20	19.4	147	80	169	56	113.00	7.10	1	1.00	1	1	1	0	1	0	0	1	0	1	0
1	1	63	2	146	49.1	1.60	23.0	115	82	214	65	149.00	5.90	1	1.00	1	0	1	1	1	1	1	1	1	1	1
1	1	77	2	147	58.2	1.60	27.0	135	51	167	66	101.00	5.60	1	1.00	1	0	1	1	1	0	1	0	0	1	1
0	0	78	1	156	55.2	-.80	22.7	146	85	181	67	114.00	5.80	1	1.00	0	1	1	0	0	0	1	1	0	1	1
1	1	53	2	151	71.7	.70	31.6	121	79	207	60	147.00	6.20	1	1.00	1	0	1	1	1	1	0	0	1	1	1
0	0	72	1	163	63.1	.50	23.7	133	81	180	74	106.00	5.20	0	1.00	0	0	1	0	1	1	1	1	0	1	1
1	1	64	1	156	55.6	-.10	22.7	139	75	261	113	148.00	6.10	0	1.00	0	1	1	0	1	1	1	1	0	0	1
1	1	49	1	165	75.9	-1.60	27.8	120	80	194	85	109.00	5.60	1	1.00	0	0	1	0	1	1	1	0	0	1	1
0	0	66	1	166	55.7	2.30	20.2	119	64	226	54	172.00	5.70	1	1.00	0	0	1	1	1	1	1	1	0	0	1
1	1	72	1	165	59.2	-.10	21.6	136	79	175	64	111.00	5.40	1	1.00	1	1	1	1	1	0	0	1	0	1	1
1	1	60	2	157	59.9	-.30	24.3	108	75	195	89	106.00	5.30	1	1.00	1	0	0	1	1	0	0	1	0	1	1
0	0	63	2	149	52.3	.70	23.6	127	84	254	56	198.00	5.40	1	1.00	1	1	1	0	0	0	1	1	0	0	1
1	1	68	1	162	61.6	.00	23.3	134	64	217	55	162.00	5.20	0	1.00	0	1	1	0	1	0	0	1	0	1	1
1	1	41	2	160	54.3	-1.20	21.2	124	76	226	109	117.00	5.50	1	1.00	0	1	1	1	1	1	1	1	1	0	1
1	1	40	1	169	78.5	-1.30	27.4	143	93	213	35	178.00	5.20	1	1.00	0	0	1	0	1	1	1	0	0	0	1
0	0	70	2	149	49.2	1.10	22.2	127	68	199	68	131.00	5.40	1	1.00	1	1	1	0	1	1	1	1	1	1	1
1	1	71	1	160	58.5	.20	22.8	132	61	198	52	146.00	5.00	1	1.00	0	1	1	1	0	0	1	1	0	0	1
1	1	77	2	154	74.7	-1.30	31.4	147	79	132	48	84.00	5.60	1	1.00	1	0	1	0	1	1	1	0	0	1	1
1	1	76	1	168	48.7	1.20	17.3	99	58	183	60	123.00	6.00	1	1.00	0	0	1	0	1	0	0	1	1	1	1
0	0	65	1	172	65.4	3.20	22.0	144	80	190	62	128.00	5.90	1	1.00	0	1	1	1	1	1	1	1	0	1	1
1	1	36	2	155	51.8	1.70	21.4	124	88	201	95	106.00	5.20	1	1.00	1	0	1	1	1	1	1	1	1	1	1
1	1	64	2	159	66.1	-1.00	26.1	124	73	227	66	161.00	5.40	1	1.00	1	0	1	0	1	1	0	0	1	0	1
1	1	47	2	161	58.2	2.50	22.4	106	68	211	69	142.00	5.70	1	1.00	1	1	1	0	1	1	1	1	1	1	1
0	0	38	2	163	72.3	-.10	27.3	123	85	272	67	205.00	6.80	1	1.00	1	1	1	0	1	0	0	0	1	0	0

0	0	62	1	163	65.3	1.50	24.6	149	91	253	98	155.00	5.30	1	1.00	0	0	1	0	1	1	1	1	0	0	1
1	1	71	1	162	60.6	1.10	23.0	151	83	185	45	140.00	5.90	1	1.00	0	1	1	1	1	1	1	1	0	1	1
1	1	41	2	153	45.2	-1.20	19.3	112	80	220	111	109.00	5.40	0	1.00	0	0	0	0	1	0	0	1	1	0	1
0	0	64	2	147	55.3	.40	25.4	94	54	194	77	117.00	5.40	1	1.00	1	0	1	0	0	1	0	0	1	1	1
1	1	68	2	154	47.0	-.90	19.8	159	90	174	52	122.00	5.50	1	1.00	1	0	1	0	1	0	0	1	0	1	1
1	1	79	2	147	50.1	.30	23.3	171	92	127	52	75.00	6.10	1	1.00	1	0	1	1	1	1	1	1	0	1	1
1	1	36	2	166	65.2	-3.50	23.7	107	65	183	40	143.00	5.50	1	1.00	1	0	1	0	0	0	1	1	1	1	1
1	1	63	2	156	46.4	.00	19.0	133	73	204	78	126.00	5.60	1	1.00	1	0	1	0	1	1	1	1	1	1	1
0	0	46	1	171	71.2	2.30	24.3	118	68	167	52	115.00	5.00	1	1.00	1	1	1	0	1	1	0	1	1	1	1
1	1	41	1	175	75.3	.80	24.5	139	87	264	57	207.00	5.20	1	1.00	0	0	1	0	1	0	1	1	1	0	1
1	1	56	2	151	47.7	-1.20	20.8	104	69	187	80	107.00	5.10	1	1.00	0	0	1	1	1	1	1	1	1	1	1
1	1	76	2	150	67.5	.50	30.1	124	63	225	66	159.00	6.50	1	1.00	1	0	1	1	1	0	0	0	0	0	0
1	1	68	2	159	58.2	.20	22.9	144	73	184	75	109.00	5.80	0	1.00	1	0	0	1	1	1	1	1	0	1	1
1	1	73	1	163	67.7	-2.00	25.6	134	79	223	54	169.00	5.90	1	1.00	1	0	1	0	1	1	1	0	1	0	0
1	1	69	1	161	59.9	.20	23.1	113	67	215	85	130.00	5.40	1	1.00	0	1	1	1	1	1	1	1	1	1	1
0	0	70	2	150	60.5	-1.30	26.8	113	53	205	55	150.00	5.60	1	1.00	1	0	1	1	1	0	1	0	0	1	1
1	1	72	1	163	72.5	.80	27.3	146	92	252	38	214.00	5.50	1	1.00	1	1	1	1	1	1	0	0	0	0	1
0	0	71	2	150	55.2	-1.90	24.6	106	64	174	64	110.00	5.80	1	1.00	1	0	1	0	1	1	1	1	1	0	1
1	1	75	1	166	67.9	-1.20	24.7	130	69	202	58	144.00	9.60	1	1.00	0	0	1	1	1	1	1	1	0	1	0
1	1	80	2	142	62.3	-.90	31.1	159	84	177	56	121.00	5.00	1	1.00	0	0	1	1	1	0	0	0	0	1	1
1	1	70	1	154	51.2	-3.10	21.7	140	67	232	82	150.00	6.20	1	1.00	0	0	0	1	1	1	1	1	0	0	1
1	1	40	2	161	55.8	.50	21.4	116	64	155	70	85.00	5.30	1	1.00	1	1	1	0	0	1	1	1	1	1	1
1	1	77	1	164	64.4	2.20	24.0	132	63	127	62	65.00	5.80	1	1.00	1	0	1	1	1	0	1	1	0	1	0
1	1	75	1	152	61.1	-.40	26.4	122	67	258	52	206.00	6.00	1	1.00	0	1	1	1	1	0	0	0	1	0	1
1	1	64	2	159	59.2	1.40	23.2	201	101	141	51	90.00	5.60	1	1.00	1	0	1	1	1	1	1	1	0	0	1
1	1	74	1	162	77.7	.00	29.6	160	89	229	40	189.00	5.50	1	1.00	0	0	1	1	0	1	1	0	0	0	1
0	0	65	2	148	65.4	-1.80	29.9	127	79	196	54	142.00	6.00	1	1.00	1	0	1	0	1	0	0	0	0	1	1
0	0	83	1	153	59.9	1.70	25.7	129	72	189	67	122.00	5.60	1	1.00	1	0	1	1	1	1	1	0	0	1	1
1	1	73	1	151	51.8	-.90	22.6	104	63	162	30	132.00	6.30	0	1.00	1	0	0	0	1	1	0	1	1	0	0
1	1	72	2	154	47.8	-1.60	20.2	97	55	108	36	72.00	5.80	1	1.00	1	0	1	1	1	1	1	1	0	0	1
1	1	73	2	147	62.2	-3.60	28.9	114	57	153	72	81.00	6.80	1	1.00	1	1	1	0	1	1	1	0	1	1	0
0	0	67	2	152	52.7	.40	22.9	137	94	224	83	141.00	5.60	1	1.00	0	0	0	0	1	0	0	1	0	0	1
1	1	73	1	172	64.7	-.20	21.7	133	80	174	49	125.00	5.30	1	1.00	1	1	1	1	1	1	1	1	1	1	1
0	0	71	1	160	67.4	.30	26.4	153	91	213	55	158.00	6.00	1	1.00	1	1	1	1	1	1	1	0	0	1	1
1	1	57	2	161	71.7	1.70	27.7	118	76	230	59	171.00	5.40	1	1.00	1	1	1	0	0	1	1	0	1	0	1
0	0	61	1	165	66.6	-.50	24.3	127	80	167	36	131.00	6.30	1	1.00	1	1	1	0	1	0	1	1	1	0	0
0	0	64	1	163	56.4	.30	21.3	145	81	222	86	136.00	5.50	1	1.00	0	0	1	1	1	1	1	1	0	0	0
1	1	68	1	164	76.9	-.30	28.4	157	72	177	46	131.00	5.20	1	1.00	0	1	1	1	1	1	1	0	0	1	1
1	1	64	2	146	49.0	-1.80	22.8	133	77	244	77	167.00	5.60	1	1.00	1	1	1	0	1	1	1	1	0	0	1

1	1	68	1	171	58.6	-1.90	19.9	152	70	172	77	95.00	6.10	1	1.00	1	1	1	0	1	1	1	1	0	1	0
0	0	51	1	176	84.1	1.10	27.0	141	90	199	51	148.00	5.00	0	1.00	1	0	1	1	1	0	1	0	0	1	1
1	1	43	2	153	57.9	-.60	24.7	112	64	248	75	173.00	5.10	1	1.00	1	0	1	0	1	0	1	1	1	0	1
1	1	81	2	145	42.5	-.40	20.2	115	64	163	101	62.00	5.30	1	1.00	1	0	1	0	1	1	1	1	0	0	1
1	1	74	2	148	53.8	.60	24.4	116	75	240	53	187.00	5.80	1	1.00	1	0	1	1	1	0	1	1	1	0	1
1	1	77	1	163	62.7	-1.90	23.5	141	73	202	53	149.00	7.00	1	1.00	0	1	1	1	1	1	1	1	0	1	0
1	1	69	2	145	63.1	1.50	30.0	155	79	219	52	167.00	6.10	1	1.00	1	0	0	1	1	1	1	0	0	1	0
1	1	72	1	157	51.7	1.30	20.9	134	96	187	56	131.00	5.40	0	1.00	0	0	0	1	1	1	1	1	0	1	1
1	1	61	2	150	64.4	.70	28.6	116	70	175	69	106.00	5.70	1	1.00	1	0	1	0	1	1	1	0	1	1	1
1	1	61	2	154	47.5	-.50	19.9	124	76	264	46	218.00	5.50	1	1.00	1	0	1	0	0	0	1	1	1	0	1
1	1	66	1	159	58.8	-1.30	23.2	137	86	153	49	104.00	5.50	0	1.00	1	0	1	0	1	1	1	1	1	1	1
1	1	83	2	150	70.3	-1.20	31.2	159	81	195	59	136.00	5.30	1	1.00	1	0	1	1	1	0	0	0	0	1	1
1	1	68	2	153	37.8	1.30	16.0	106	68	182	68	114.00	5.50	1	1.00	1	0	1	1	1	0	0	1	1	1	1
1	1	63	1	162	66.6	.00	25.3	117	77	220	79	141.00	5.50	1	1.00	1	0	1	0	0	0	0	0	1	0	1
1	1	62	1	178	82.9	1.20	26.3	134	80	241	86	155.00	5.60	0	1.00	0	1	1	1	1	1	1	0	1	0	1
1	1	69	1	164	60.0	2.90	22.2	103	68	165	63	102.00	5.80	0	1.00	0	0	1	0	1	0	0	1	1	1	1
1	1	82	1	154	57.8	-.80	24.3	121	61	219	95	124.00	5.50	1	1.00	0	0	1	1	1	1	1	1	0	1	1
1	1	64	2	159	46.0	-1.30	18.2	124	78	206	69	137.00	4.90	1	1.00	1	0	1	1	1	1	1	1	1	1	1
0	0	75	2	137	59.0	3.00	31.3	151	82	186	72	114.00	5.60	1	1.00	1	1	1	0	1	1	1	0	0	1	1
1	1	66	1	167	61.9	-.90	22.1	133	86	204	63	141.00	5.60	1	1.00	0	0	1	1	1	0	1	1	0	1	1
1	1	75	1	163	80.1	-.30	30.1	163	99	228	54	174.00	4.80	1	1.00	0	1	1	0	1	1	1	0	0	0	1
0	0	58	2	149	54.0	-.90	24.2	102	64	212	106	106.00	5.70	1	1.00	0	1	1	1	0	1	1	1	1	1	1
0	0	63	2	148	57.3	-1.40	26.1	127	74	150	58	92.00	5.10	0	1.00	1	0	1	1	1	1	1	0	1	1	1
0	0	56	2	147	60.5	-2.40	27.9	131	84	234	80	154.00	5.40	1	1.00	1	0	1	0	0	1	1	0	1	0	1
0	0	76	1	154	59.7	-.80	25.0	137	80	202	44	158.00	5.70	1	1.00	0	0	1	1	1	1	1	0	1	1	1
1	1	66	2	154	52.8	1.60	22.2	130	80	286	130	156.00	5.70	1	1.00	1	1	1	1	1	1	1	1	1	0	1
1	1	62	2	140	60.8	-2.30	31.1	129	81	183	45	138.00	5.90	1	1.00	1	0	1	0	0	1	1	0	1	1	1
0	0	73	1	164	56.8	.50	21.1	137	71	186	77	109.00	6.80	1	1.00	0	0	1	1	1	1	1	1	1	1	0
0	0	42	2	154	64.5	-1.80	27.2	103	57	174	73	101.00	4.70	0	1.00	1	1	1	0	1	0	1	0	1	1	1
0	0	51	2	168	60.2	-1.30	21.3	123	70	184	77	107.00	5.20	1	1.00	0	0	1	1	1	1	1	1	1	1	1
1	1	79	2	140	54.0	.30	27.6	119	69	167	51	116.00	5.80	1	1.00	1	1	1	1	1	0	0	0	0	1	0
0	0	47	1	168	73.0	-1.50	25.8	125	82	191	44	147.00	5.60	0	1.00	0	0	0	0	0	1	0	0	1	1	1
0	0	35	1	165	72.4	-2.00	26.4	122	69	182	47	135.00	5.40	0	1.00	0	1	1	0	1	0	0	0	1	1	1
1	1	26	1	168	69.0	-.30	24.4	119	68	178	45	133.00	5.10	0	1.00	1	1	1	0	0	0	1	1	1	1	1
0	0	25	2	152	74.9	-5.90	32.3	116	70	166	46	120.00	4.80	1	1.00	1	0	1	1	1	0	1	0	1	1	1
0	0	72	2	150	49.5	.00	22.1	131	76	194	58	136.00	5.30	1	1.00	1	0	1	0	1	0	0	1	1	1	1
0	0	51	1	166	79.1	-.20	28.5	133	80	162	36	126.00	5.90	0	1.00	0	1	1	1	1	1	0	0	1	0	1
0	0	68	2	157	58.3	-1.00	23.6	137	68	175	53	122.00	5.50	1	1.00	1	1	1	1	1	0	0	1	0	1	1
1	1	84	1	165	69.2	-.30	25.3	134	80	172	46	126.00	5.50	1	1.00	1	0	1	1	1	0	1	0	1	1	1

0	0	50	1	166	75.9	2.30	27.5	116	80	192	34	158.00	6.10	0	1.00	1	1	1	1	1	1	1	0	0	0	1
0	0	64	1	167	64.3	-.60	23.1	122	86	244	45	199.00	5.60	1	1.00	0	0	1	0	1	0	1	1	1	0	1
1	1	84	1	159	68.8	-1.90	27.1	101	58	156	59	97.00	5.40	1	1.00	0	0	1	1	1	1	1	0	0	1	1
0	0	50	2	157	48.4	.40	19.7	119	75	284	69	215.00	5.90	0	1.00	1	1	1	0	1	1	1	1	1	0	1
1	1	65	1	179	57.3	-.50	17.9	109	74	181	57	124.00	5.30	0	1.00	1	1	1	0	0	1	0	1	1	1	1
0	0	35	2	160	65.3	-1.20	25.5	113	73	177	76	101.00	5.40	1	1.00	0	0	0	0	1	0	0	0	1	1	1
1	1	58	2	146	47.8	.70	22.5	104	67	177	64	113.00	5.40	1	1.00	1	0	1	0	1	1	1	1	1	1	1
1	1	61	2	156	60.6	2.00	24.9	150	81	218	61	157.00	5.50	1	1.00	1	1	1	0	1	1	1	1	0	0	1
1	1	69	2	150	59.6	1.00	26.4	109	58	153	43	110.00	5.40	1	1.00	1	1	1	1	1	1	1	0	1	1	1
0	0	43	2	163	66.4	4.10	24.9	155	105	222	94	128.00	4.90	0	1.00	0	0	0	1	1	1	1	1	0	0	1
0	0	35	1	168	76.8	2.30	27.3	151	97	215	49	166.00	5.90	1	1.00	0	0	1	0	1	0	1	0	0	1	1
1	1	29	1	174	53.4	3.10	17.6	126	70	198	54	144.00	5.40	1	1.00	1	0	1	1	1	1	1	1	1	1	1
1	1	61	2	158	53.6	.20	21.5	144	80	187	74	113.00	5.40	1	1.00	1	0	0	1	0	1	1	1	0	1	1
1	1	54	2	151	55.5	-1.70	24.3	174	109	192	67	125.00	5.60	1	1.00	0	1	1	0	1	1	1	1	0	1	1
0	0	64	1	162	61.7	2.30	23.3	146	83	157	39	118.00	5.30	1	1.00	0	1	1	1	1	1	1	1	0	0	1
1	1	75	2	144	46.8	.30	22.4	126	78	189	60	129.00	5.80	1	1.00	1	0	1	1	1	0	0	1	1	1	1
1	1	78	1	161	54.1	-.50	20.7	117	74	174	48	126.00	5.20	1	1.00	1	1	1	1	1	1	1	1	1	1	1
1	1	69	2	136	51.6	.40	27.8	156	88	205	53	152.00	5.90	1	1.00	1	1	1	1	1	1	1	0	0	1	1
1	1	61	2	156	41.9	.60	17.2	138	83	174	57	117.00	5.30	1	1.00	1	0	1	0	0	0	0	1	1	1	1
1	1	65	2	150	52.4	-.30	23.3	123	78	206	54	152.00	5.80	0	1.00	1	0	1	0	1	0	0	1	0	0	1
1	1	48	2	152	62.1	-.70	26.7	100	61	268	52	216.00	5.10	1	1.00	1	0	1	1	1	1	1	0	1	0	1
1	1	51	2	150	69.0	.50	30.5	123	69	185	73	112.00	5.20	1	1.00	1	0	1	1	1	0	1	0	1	0	1
1	1	46	2	151	50.4	-1.20	22.0	109	70	205	71	134.00	4.80	1	1.00	1	1	1	1	0	1	1	1	1	1	1
1	1	59	2	165	66.5	2.60	24.5	106	68	171	68	103.00	5.20	1	1.00	0	1	1	1	1	1	0	1	1	1	1
1	1	72	2	150	55.4	-.10	24.6	111	74	228	82	146.00	5.90	1	1.00	1	0	1	1	1	1	1	1	1	0	1
1	1	74	2	149	57.4	.40	25.7	116	71	176	60	116.00	5.50	1	1.00	1	0	1	1	1	0	0	0	1	1	1
1	1	59	2	151	54.9	-.50	24.0	153	83	241	118	123.00	6.40	1	1.00	1	1	1	1	1	1	1	1	0	0	1
1	1	48	1	167	73.5	-2.20	26.4	140	77	217	57	160.00	5.20	1	1.00	0	0	1	0	1	0	0	0	0	1	1
1	1	56	2	149	43.9	.20	19.6	119	73	130	75	55.00	5.20	1	1.00	1	1	1	0	1	1	0	1	1	1	1
0	0	72	1	166	73.0	-2.20	26.6	167	86	234	41	193.00	5.50	1	1.00	0	1	1	1	1	1	1	0	0	0	1
1	1	63	2	153	59.3	.90	25.4	123	79	189	76	113.00	5.60	1	1.00	1	1	1	1	1	1	1	0	0	1	1
0	0	66	2	145	51.6	.00	24.7	134	80	173	46	127.00	5.60	1	1.00	1	1	1	0	0	1	1	1	1	1	1
1	1	61	2	151	61.3	.40	26.7	116	79	180	51	129.00	5.50	1	1.00	1	1	1	1	1	1	1	0	0	1	1
1	1	53	2	167	68.0	-.20	24.3	123	69	241	68	173.00	5.30	1	1.00	1	0	1	0	1	0	0	1	1	0	1
1	1	83	1	150	57.3	1.30	25.4	124	54	169	27	142.00	5.30	1	1.00	1	1	0	0	0	0	0	0	0	0	1
1	1	63	1	167	58.0	.10	20.7	136	76	253	80	173.00	5.80	0	2.00	0	1	1	1	1	1	1	1	1	0	1
1	1	45	2	152	58.8	1.90	25.4	115	77	223	60	163.00	5.10	1	1.00	1	0	1	0	0	0	0	0	1	0	1
1	1	69	2	154	58.5	-2.60	24.5	126	79	224	60	164.00	5.80	1	1.00	0	0	1	0	1	1	0	1	0	0	1
1	1	39	1	176	84.9	-.60	27.5	106	59	246	90	156.00	5.40	1	1.00	0	1	1	0	1	1	1	0	1	0	1

0	0	61	2	147	36.9	1.20	17.1	88	60	220	52	168.00	5.60	1	1.00	1	1	1	1	1	0	1	1	1	0	1
1	1	48	2	157	52.0	.50	21.1	111	66	216	100	116.00	5.70	1	1.00	1	0	1	1	1	0	0	1	0	1	1
0	0	74	1	162	67.4	-3.90	25.8	141	84	194	65	129.00	6.10	1	1.00	1	0	1	0	1	1	1	0	0	1	1
0	0	52	1	173	74.6	-1.30	24.8	121	90	209	64	145.00	5.30	1	1.00	0	0	1	0	1	1	1	1	0	1	1
0	0	75	1	160	57.9	-4.00	22.5	119	68	184	53	131.00	5.70	1	1.00	1	1	1	1	1	1	1	1	0	0	1
0	0	82	2	153	62.8	1.70	26.6	132	71	239	38	201.00	5.40	1	1.00	1	0	1	0	0	0	0	0	0	0	1
0	0	64	2	150	50.3	.00	22.2	149	95	276	112	164.00	5.10	1	1.00	1	1	1	0	0	0	1	1	0	0	1
0	0	69	1	162	63.6	-2.10	24.1	120	82	250	41	209.00	5.50	1	1.00	1	0	1	0	1	1	1	1	1	0	1
1	1	48	2	156	76.6	.90	31.4	142	93	148	50	98.00	5.50	1	1.00	1	0	1	1	1	1	1	0	0	1	1
1	1	68	1	162	58.1	-3.50	22.2	119	69	245	60	185.00	5.40	0	1.00	0	0	1	1	1	1	1	1	1	0	1
0	0	74	1	163	65.4	-1.10	24.5	137	83	244	59	185.00	5.50	1	1.00	0	0	1	0	1	0	0	1	1	0	1
1	1	70	2	148	44.1	-2.00	20.0	193	109	214	87	127.00	5.20	1	1.00	1	0	1	1	1	1	1	1	0	1	1
0	0	42	2	152	49.7	1.10	21.6	107	60	190	66	124.00	5.40	1	1.00	0	0	1	1	1	1	1	1	1	1	1
0	0	54	1	175	113.9	-.20	37.0	137	89	226	45	181.00	6.20	1	1.00	1	0	1	1	0	0	0	0	1	0	0
0	0	56	2	160	42.3	1.80	16.5	166	84	195	76	119.00	6.20	1	1.00	1	1	1	0	1	0	0	1	0	1	0
1	1	66	1	160	62.8	.50	24.4	124	84	222	59	163.00	5.80	1	1.00	0	0	1	0	1	1	1	1	0	0	1
1	1	52	2	150	55.9	.00	24.8	112	75	235	38	197.00	5.40	1	1.00	1	1	1	1	1	0	0	1	1	0	1
1	1	48	1	166	64.4	-3.50	23.4	152	99	251	99	152.00	5.30	0	1.00	0	0	0	0	0	1	1	1	0	0	1
0	0	40	2	159	47.1	.10	18.5	120	61	214	63	151.00	5.50	1	1.00	1	1	1	0	0	1	0	1	1	1	1
0	0	59	2	152	67.8	-1.60	29.4	121	69	213	47	166.00	5.90	1	1.00	1	0	1	0	0	0	1	0	0	1	1
1	1	56	1	175	67.3	-1.20	22.0	116	69	201	78	123.00	5.30	0	1.00	1	1	1	0	1	1	1	1	1	1	1
1	1	69	1	152	49.3	.20	21.3	134	83	198	59	139.00	5.30	1	1.00	0	0	1	1	1	1	1	1	0	1	1
0	0	49	2	150	45.5	1.60	20.2	118	73	232	82	150.00	5.60	1	1.00	1	1	1	1	1	1	1	1	1	0	1
1	1	41	1	169	64.1	1.80	22.3	108	61	218	76	142.00	5.80	1	1.00	1	0	1	0	0	0	1	1	1	1	1
1	1	64	2	156	49.1	-.20	20.1	115	74	217	59	158.00	5.60	1	1.00	1	1	1	1	1	1	0	1	1	1	1
1	1	72	2	148	52.0	.10	23.7	142	77	237	56	181.00	5.80	1	1.00	1	0	1	0	1	0	0	1	0	0	1
0	0	47	2	164	71.6	-.40	26.6	134	87	203	38	165.00	6.10	1	1.00	1	0	1	0	1	0	1	0	0	0	1
1	1	75	2	140	55.1	-.20	28.1	161	94	212	50	162.00	5.70	1	1.00	1	1	1	0	1	1	1	0	0	1	1
1	1	42	2	155	69.4	-1.30	28.9	101	67	220	105	115.00	5.80	1	1.00	0	0	0	0	0	0	0	0	1	0	1
1	1	79	1	152	56.7	2.70	24.4	108	72	240	56	184.00	5.40	1	1.00	0	1	1	0	1	1	1	1	1	0	1
1	1	79	2	152	54.4	-.10	23.5	155	78	215	66	149.00	6.30	1	1.00	1	0	1	0	1	1	1	1	0	1	1
1	1	71	2	151	54.1	-2.90	23.6	104	55	272	58	214.00	6.40	1	1.00	1	1	1	1	1	1	0	1	0	0	1
0	0	82	2	154	48.5	-2.10	20.4	99	63	137	53	84.00	6.00	1	1.00	1	0	1	1	1	0	1	1	0	1	1
1	1	53	2	146	54.1	.90	25.4	123	75	223	67	156.00	5.70	1	1.00	1	0	1	0	1	0	0	0	1	0	1
1	1	57	2	153	40.8	-.80	17.4	122	68	136	46	90.00	5.10	1	1.00	1	1	1	0	1	0	0	1	1	1	1
1	1	70	1	176	74.4	-1.60	24.0	140	82	237	62	175.00	5.90	1	1.00	0	1	1	1	1	1	1	1	0	0	1
1	1	66	2	140	40.4	-.90	20.7	127	69	290	24	266.00	5.70	1	1.00	1	0	1	0	1	0	0	1	0	0	0
1	1	65	2	152	68.0	1.10	29.4	122	72	195	49	146.00	5.50	1	1.00	0	1	1	1	1	1	1	0	0	1	1
1	1	85	1	163	66.3	-2.40	25.1	130	80	181	37	144.00	5.60	1	1.00	0	1	1	1	1	1	1	0	0	0	1

1	1	59	1	174	68.9	1.30	22.8	112	63	219	73	146.00	5.30	0	1.00	0	0	0	1	1	1	1	1	1	1	1
0	0	71	1	170	85.7	-1.70	29.6	162	82	171	64	107.00	5.10	1	1.00	0	0	1	0	1	0	0	0	0	1	1
0	0	50	1	174	72.4	3.80	23.8	120	74	247	53	194.00	5.70	0	1.00	0	1	1	0	1	0	0	1	1	0	1
1	1	65	2	154	43.5	-.30	18.3	122	80	143	38	105.00	5.90	1	1.00	1	0	1	1	1	1	1	1	1	0	1
0	0	60	2	141	58.6	1.80	29.6	123	70	180	57	123.00	5.60	1	1.00	1	0	1	1	1	0	0	0	0	0	1
1	1	50	2	158	55.5	2.50	22.3	115	61	195	73	122.00	5.40	1	1.00	1	0	1	1	1	1	0	1	1	1	1
0	0	62	2	153	71.6	-.60	30.5	144	82	182	62	120.00	6.60	1	1.00	0	0	1	0	1	0	1	0	0	1	0
1	1	80	1	159	65.0	-1.80	25.8	137	58	155	53	102.00	5.90	1	1.00	1	1	1	0	1	1	1	0	1	1	1
0	0	63	2	148	43.4	.50	19.8	129	75	152	58	94.00	4.80	1	1.00	1	0	1	0	0	0	1	1	0	1	1
0	0	76	2	158	51.4	2.00	20.5	135	67	244	66	178.00	5.50	1	1.00	1	0	1	1	1	1	0	1	0	0	1
0	0	51	2	152	71.6	.80	31.0	153	96	207	96	111.00	5.30	1	1.00	1	1	1	0	1	0	1	0	0	1	1
0	1	68	1	165	60.1	-1.50	22.1	157	83	172	40	132.00	6.30	0	1.00	0	0	1	1	1	0	1	1	0	1	0
0	0	69	1	167	73.9	-1.60	26.4	106	69	212	49	163.00	7.30	1	1.00	0	0	1	0	1	0	0	0	0	1	0
0	0	77	2	149	36.2	.40	16.3	129	79	166	117	49.00	5.80	1	1.00	0	1	1	0	1	0	0	1	1	1	1
0	0	74	1	165	73.8	-.70	27.1	147	70	171	56	115.00	7.70	1	1.00	1	1	1	1	1	1	1	0	0	1	0
1	1	66	2	145	56.5	1.20	27.0	184	92	195	66	129.00	5.20	1	1.00	1	1	1	0	1	1	1	0	0	0	1
1	1	62	2	142	46.2	.40	22.8	114	65	247	74	173.00	5.50	1	1.00	1	1	1	1	1	1	1	1	1	0	1
1	1	75	2	150	56.9	-.70	25.4	143	73	213	67	146.00	4.70	1	1.00	1	1	1	0	1	1	0	0	0	1	1
0	0	59	1	167	68.8	1.90	24.6	161	93	225	76	149.00	5.20	1	1.00	1	0	1	1	0	0	0	1	0	0	1
1	1	61	2	145	51.4	-1.20	24.3	143	79	245	83	162.00	5.10	1	1.00	1	1	1	0	1	1	1	1	0	0	1
1	1	75	2	150	59.0	-2.20	26.1	113	66	248	72	176.00	5.80	1	1.00	1	1	1	0	1	1	1	0	1	0	1
1	1	78	1	165	59.8	.80	21.8	136	71	183	44	139.00	6.20	1	1.00	1	0	1	1	1	1	1	1	1	0	1
1	1	69	1	180	74.9	-3.20	23.1	110	68	159	57	102.00	5.70	0	1.00	0	1	1	0	1	1	1	1	1	1	1
1	1	77	2	143	59.2	1.60	28.7	144	81	184	94	90.00	5.10	1	1.00	1	0	1	1	1	1	1	0	0	0	1
1	1	72	2	150	58.8	-1.40	26.2	125	75	144	61	83.00	5.50	1	1.00	1	1	1	1	1	1	1	0	0	1	1
1	1	76	1	157	58.4	.40	23.7	140	81	215	93	122.00	5.50	1	1.00	0	0	1	1	1	1	1	1	0	1	1
1	1	72	2	154	56.1	.80	23.8	101	61	175	43	132.00	5.20	1	1.00	0	0	1	1	1	1	0	1	0	1	1
1	1	69	2	158	51.5	.10	20.5	117	75	203	83	120.00	5.80	1	1.00	0	0	1	1	1	1	0	1	1	1	1
1	1	61	2	151	57.3	-.80	25.1	102	59	217	74	143.00	5.20	1	1.00	1	0	1	1	1	1	1	0	1	1	1
1	1	67	2	155	67.1	.10	28.0	148	80	236	85	151.00	5.50	1	1.00	1	1	1	1	1	1	1	0	0	0	1
1	1	55	2	162	58.4	.10	22.1	125	69	239	56	183.00	5.50	1	1.00	1	0	1	0	0	0	0	1	1	0	1
1	1	82	1	150	63.2	-.60	28.1	126	61	182	69	113.00	5.60	1	1.00	0	0	1	1	1	1	1	0	0	1	1
1	1	60	2	154	46.0	.80	19.2	122	60	252	44	208.00	5.50	1	1.00	1	0	1	1	1	1	0	1	1	0	1
0	0	85	1	157	68.6	-.70	27.7	153	88	190	95	95.00	5.60	1	1.00	0	0	1	0	1	0	0	0	0	1	1
1	1	74	2	149	54.9	1.10	24.7	128	73	176	53	123.00	5.50	1	1.00	1	0	1	1	1	0	0	1	1	1	1
1	1	83	2	158	56.2	-3.60	22.4	133	79	187	47	140.00	5.40	1	1.00	1	0	1	1	1	0	1	1	1	1	1
1	1	60	2	149	47.2	-.60	21.3	115	77	183	83	100.00	5.10	1	1.00	1	0	1	0	0	0	0	1	1	1	1
0	0	28	2	153	43.8	.30	18.7	97	52	193	81	112.00	5.30	1	1.00	1	1	0	1	0	1	0	1	1	1	1
0	1	51	2	143	46.9	-.60	22.8	128	79	243	101	142.00	5.10	1	1.00	0	0	1	0	1	0	1	1	1	0	1

1	1	80	1	159	62.6	-.30	24.7	116	58	186	52	134.00	5.10	1	1.00	0	0	1	0	1	0	0	1	1	1	1
0	0	77	2	152	43.7	.70	18.8	129	68	196	53	143.00	5.20	1	1.00	1	0	1	0	1	0	0	1	1	1	0
1	1	59	2	158	78.3	.40	31.3	121	70	200	52	148.00	5.30	0	1.00	1	0	1	0	0	0	0	0	1	1	1
0	0	67	1	169	72.9	1.60	25.5	104	66	160	33	127.00	6.30	0	1.00	1	0	1	0	1	1	1	0	1	0	0
0	0	79	1	154	50.4	-.70	21.1	124	69	166	68	98.00	5.30	1	1.00	0	1	1	0	1	1	1	1	0	1	1
1	1	70	1	154	49.7	.90	20.9	130	68	240	118	122.00	5.60	1	1.00	1	0	1	1	1	1	1	1	1	0	1
1	1	63	2	147	56.1	-.60	25.9	124	58	256	78	178.00	5.30	1	1.00	1	0	1	0	1	0	0	0	1	0	1
0	1	53	2	159	48.9	-.70	19.4	127	88	172	67	105.00	5.40	1	1.00	0	0	0	0	0	1	0	1	1	0	1
0	0	70	1	171	63.0	3.10	21.4	127	72	144	43	101.00	5.10	1	1.00	1	0	1	1	1	0	1	1	1	1	1
0	0	70	1	158	64.6	-.50	25.7	135	84	248	34	214.00	4.80	0	1.00	0	0	0	0	1	0	0	0	1	0	1
0	0	43	2	163	78.5	-1.60	29.6	117	75	238	49	189.00	7.00	1	1.00	1	0	1	0	1	0	0	0	1	0	0
1	1	65	2	150	83.9	-.30	37.2	168	74	201	45	156.00	5.50	1	1.00	1	0	1	1	0	0	1	0	0	1	1
1	1	61	1	166	82.7	2.20	30.1	130	83	181	58	123.00	6.10	1	1.00	0	1	1	0	0	0	0	0	0	1	1
1	1	53	1	165	70.4	.70	25.7	144	90	196	95	101.00	5.00	1	1.00	0	1	1	0	1	1	0	0	0	1	1
0	0	29	2	166	59.4	-.30	21.4	100	52	173	71	102.00	5.00	1	1.00	1	1	1	0	1	0	1	1	1	1	1
1	1	48	2	162	52.2	-.40	19.8	115	65	280	70	210.00	5.20	1	1.00	1	1	1	0	1	1	0	1	1	0	1
1	1	68	2	152	59.2	-.80	25.6	147	83	211	56	155.00	5.60	1	1.00	1	1	1	0	1	0	1	0	0	1	1
0	0	63	2	143	49.5	2.40	24.2	147	74	226	58	168.00	5.90	1	1.00	1	0	0	0	1	0	0	1	0	0	1
1	1	58	2	153	50.3	.70	21.5	154	86	250	68	182.00	5.60	1	1.00	0	1	1	0	1	0	1	1	0	0	1
0	0	39	2	154	49.0	-.50	20.5	98	54	196	85	111.00	5.30	1	1.00	1	0	1	1	1	0	0	1	1	1	1
0	0	36	1	168	77.9	-14.20	27.5	148	90	247	51	196.00	5.70	0	1.00	1	1	1	0	0	0	0	0	0	0	1
0	0	49	1	172	95.0	3.30	32.1	142	84	254	40	214.00	5.20	1	1.00	0	0	1	0	0	1	1	0	0	0	1
1	1	65	1	165	77.6	-.40	28.3	179	95	215	40	175.00	6.00	0	1.00	1	1	1	1	0	1	1	0	0	1	1
1	1	60	2	157	50.1	-1.60	20.4	109	61	229	65	164.00	5.70	1	1.00	1	0	0	0	0	1	1	1	1	0	1
1	1	68	2	139	51.3	-.80	26.7	118	68	151	57	94.00	7.30	1	1.00	1	0	1	0	1	0	0	0	0	1	0
1	1	59	1	161	64.3	.40	24.7	129	79	191	44	147.00	5.30	0	1.00	0	1	1	1	1	1	1	1	1	1	1
1	1	32	2	157	55.2	.60	22.2	103	56	184	77	107.00	5.00	1	1.00	1	0	1	1	1	0	1	1	1	1	1
1	1	40	2	163	50.9	.40	19.1	100	60	176	79	97.00	5.00	0	1.00	1	1	1	0	1	1	1	1	1	1	1
1	1	54	1	174	74.9	-.20	24.7	163	105	300	86	214.00	5.40	0	1.00	0	0	1	1	1	0	0	1	0	0	1
1	1	61	1	165	58.2	-1.40	21.2	126	72	211	68	143.00	5.70	0	1.00	1	1	1	0	1	1	1	1	1	1	1
1	1	38	2	161	66.8	-1.40	25.8	115	67	249	58	191.00	5.30	1	1.00	1	0	1	0	0	0	0	0	1	0	1
1	1	30	1	164	67.0	-1.60	24.9	120	68	137	46	91.00	5.40	0	1.00	1	0	1	0	0	1	1	1	1	1	1
1	1	56	1	167	50.4	-.60	18.1	154	85	193	67	126.00	5.40	0	1.00	0	1	1	0	1	0	1	1	0	1	1
1	1	64	2	152	52.1	-.20	22.6	136	75	295	121	174.00	5.40	1	1.00	1	1	1	0	1	1	1	1	0	0	1
1	1	59	2	149	54.4	.00	24.6	135	80	181	60	121.00	6.40	1	1.00	1	0	1	0	1	1	1	1	1	0	0
0	0	56	2	148	54.5	-3.10	24.7	128	76	166	64	102.00	5.70	1	1.00	1	0	1	0	1	0	0	1	1	0	1
1	1	71	1	168	61.2	1.10	21.6	119	78	175	51	124.00	5.60	1	1.00	0	0	1	1	1	1	1	1	1	1	1
1	1	58	2	148	57.1	.10	25.9	107	64	209	60	149.00	5.40	1	1.00	1	1	1	1	1	0	0	0	1	0	1
1	1	76	2	136	44.0	.80	23.7	127	73	269	94	175.00	5.20	1	1.00	1	1	1	1	1	1	1	1	1	0	1

0	0	70	2	153	51.8	-1.90	22.1	126	72	159	65	94.00	5.70	1	1.00	1	0	1	0	1	1	1	1	1	1	1
1	1	79	1	157	63.7	-.50	25.8	123	68	179	36	143.00	5.30	1	1.00	0	1	1	1	1	0	0	0	1	0	1
1	1	54	2	153	50.9	.80	21.8	89	68	184	79	105.00	5.90	1	1.00	1	0	1	1	1	1	1	1	1	1	1
1	1	74	2	152	54.8	-.40	23.6	135	76	143	63	80.00	5.10	1	1.00	1	0	1	0	1	0	1	1	0	1	1
1	1	73	1	167	71.9	-1.30	25.6	150	95	140	36	104.00	4.80	1	1.00	0	0	1	1	1	1	1	0	0	0	1
1	1	71	2	157	50.6	-.90	20.5	123	73	204	77	127.00	5.20	1	1.00	1	1	1	0	1	1	1	1	1	1	1
0	0	75	2	152	59.8	.70	25.8	126	68	204	88	116.00	5.60	1	1.00	1	1	1	0	1	0	1	0	0	1	1
1	1	60	2	156	54.1	1.60	22.1	132	82	291	54	237.00	5.30	1	1.00	1	0	1	1	1	1	0	1	1	0	1
1	1	60	1	164	63.4	-5.30	23.7	147	80	186	58	128.00	5.50	1	1.00	0	1	0	0	1	0	0	1	0	1	1
0	0	63	2	157	52.7	-2.60	21.2	140	83	200	58	142.00	6.10	1	1.00	1	0	1	1	1	1	1	1	0	1	1
1	1	58	1	167	70.2	-.80	25.1	128	85	166	78	88.00	5.20	1	1.00	0	0	0	0	1	0	0	0	0	0	1
1	1	61	2	161	66.6	.90	25.6	133	87	206	70	136.00	5.40	1	1.00	1	0	1	1	1	0	1	0	1	1	1
0	0	62	2	157	48.9	-1.90	19.7	131	77	204	77	127.00	5.40	1	1.00	1	1	1	1	1	1	1	1	1	1	1
1	1	68	1	171	81.9	-4.90	28.0	134	85	139	49	90.00	6.50	0	1.00	0	1	1	0	0	1	0	0	0	1	0
0	0	59	2	155	49.7	-.70	20.7	133	84	212	69	143.00	5.50	1	1.00	1	0	0	0	0	0	1	1	1	1	1
1	1	59	1	168	67.8	-1.10	24.1	115	67	230	55	175.00	5.60	1	1.00	0	0	1	0	1	0	0	1	1	0	1
1	1	33	2	159	55.1	-.10	21.8	102	57	211	80	131.00	5.00	1	1.00	1	0	1	0	1	1	1	1	1	1	1
1	1	62	2	152	44.8	.90	19.5	93	57	317	57	260.00	5.70	1	1.00	1	1	0	1	1	0	0	1	1	0	1
1	1	60	2	153	61.0	-1.60	26.0	109	72	210	62	148.00	5.40	1	1.00	1	1	0	0	1	0	1	0	0	1	1
1	1	57	2	155	60.1	-.40	24.8	127	69	196	79	117.00	5.30	1	1.00	0	1	1	1	1	1	1	1	1	1	1
0	0	61	1	176	75.7	-2.40	24.3	118	78	172	58	114.00	5.30	1	1.00	0	0	0	0	1	0	0	1	0	1	1
1	1	59	2	166	52.6	-.50	19.1	107	62	204	63	141.00	5.50	1	1.00	1	1	1	0	0	0	1	1	1	1	1
1	1	56	1	173	94.0	-1.30	31.2	167	93	188	46	142.00	6.70	0	1.00	0	0	1	0	1	1	1	0	0	1	0
0	1	82	1	160	63.1	-2.40	24.7	155	80	225	44	181.00	5.90	1	1.00	1	1	1	1	1	1	1	1	0	0	1
0	0	63	2	149	49.2	.50	22.3	118	69	255	75	180.00	5.80	1	1.00	0	0	1	0	1	0	1	1	1	0	1
0	0	63	1	172	86.7	-5.60	29.4	130	86	159	39	120.00	5.60	1	1.00	1	0	1	0	1	0	0	0	1	0	1
1	1	54	2	161	69.2	-1.10	26.5	142	86	211	64	147.00	5.30	1	1.00	1	1	1	1	1	1	1	0	0	1	1
0	0	56	2	158	56.2	-.60	22.4	118	73	282	86	196.00	5.20	1	1.00	1	0	1	0	0	1	1	1	0	0	1
0	0	68	2	149	55.9	-.10	25.2	146	92	291	63	228.00	5.40	1	1.00	1	0	1	1	1	1	1	0	0	0	1
1	1	59	1	170	57.7	.70	19.9	157	93	222	75	147.00	5.60	1	1.00	0	1	1	0	1	1	1	1	0	0	1
0	0	38	2	161	47.1	.10	18.1	111	54	163	78	85.00	5.00	1	1.00	1	0	1	1	1	1	1	1	1	1	1
0	0	57	2	152	43.8	-.70	19.0	103	61	199	97	102.00	5.30	1	1.00	1	0	1	0	1	0	1	1	1	1	1
0	0	77	1	160	61.2	-.30	23.9	122	84	230	55	175.00	5.10	1	1.00	1	0	1	0	1	0	0	1	1	0	1
0	0	70	1	164	72.9	1.40	26.9	141	88	203	67	136.00	5.20	1	1.00	0	0	1	0	1	0	0	0	0	1	1
1	1	30	1	173	66.8	5.60	22.3	120	70	177	63	114.00	5.10	1	1.00	1	0	1	1	1	0	1	1	1	1	1
1	1	55	1	171	66.5	-1.80	22.6	122	83	186	60	126.00	4.90	1	1.00	0	1	1	1	1	0	1	1	1	1	1
0	0	63	2	156	67.4	2.70	27.6	149	79	286	82	204.00	5.60	1	1.00	1	0	1	1	0	0	0	0	0	0	1
1	1	68	2	154	43.4	.40	18.2	133	69	255	81	174.00	5.40	1	1.00	1	1	1	1	1	1	1	1	1	0	1
0	0	73	2	141	47.0	.80	23.7	121	70	125	36	89.00	4.60	1	1.00	1	0	0	0	1	1	1	1	0	0	1

1	1	66	1	165	60.1	-.90	22.1	135	76	225	85	140.00	5.60	0	1.00	0	1	1	0	1	0	1	1	1	0	1
1	1	44	2	162	51.9	2.60	19.8	97	66	234	54	180.00	5.30	0	2.00	1	0	0	0	1	0	0	1	1	0	1
1	1	39	1	168	100.7	1.80	35.8	138	90	279	50	229.00	8.70	1	1.00	0	0	1	0	1	0	1	0	0	0	0
1	1	69	2	150	48.6	-.10	21.5	88	49	194	75	119.00	5.40	1	1.00	1	0	1	0	1	0	0	1	0	0	1
1	1	60	2	142	38.5	-.40	19.1	122	75	253	71	182.00	5.40	1	1.00	1	1	1	0	1	0	1	1	1	0	1
1	1	38	1	167	58.8	-2.40	21.1	109	72	202	86	116.00	4.90	1	1.00	0	1	1	0	0	0	1	1	1	1	1
0	0	71	1	166	61.5	-.60	22.4	88	55	203	52	151.00	6.30	1	1.00	1	0	1	0	1	1	1	1	1	1	0
1	1	76	1	157	57.4	-3.80	23.1	133	75	182	57	125.00	5.30	0	1.00	0	1	1	1	1	0	1	1	1	1	1
1	1	64	2	150	50.0	-.60	22.3	132	82	206	84	122.00	5.50	1	1.00	1	1	1	0	1	1	1	1	1	1	1
1	1	63	2	154	45.2	2.30	19.0	101	59	218	90	128.00	5.90	1	1.00	1	0	1	1	1	1	1	1	1	1	1
0	0	71	2	144	52.6	-1.10	25.3	134	86	226	60	166.00	5.90	1	1.00	1	1	1	1	0	1	1	0	0	0	1
1	1	62	1	163	63.1	-.30	23.7	113	73	175	67	108.00	5.40	1	1.00	0	0	1	0	1	0	0	1	1	1	1
1	1	59	2	153	52.6	.90	22.5	132	80	259	54	205.00	5.40	1	1.00	1	0	1	0	0	1	0	1	1	0	1
1	1	66	2	150	51.1	1.30	22.5	124	72	287	90	197.00	6.00	1	1.00	1	0	1	0	1	1	1	1	1	0	1
1	1	68	2	154	60.1	1.30	25.4	110	67	207	43	164.00	5.40	1	1.00	1	1	1	1	1	1	1	0	0	1	1
1	1	41	2	153	52.5	.10	22.4	95	63	185	66	119.00	5.10	0	1.00	1	1	1	1	1	1	1	1	1	1	1
0	0	52	1	162	64.9	1.10	24.7	137	78	278	39	239.00	6.40	1	1.00	1	1	1	0	1	0	0	1	1	0	1
0	0	59	2	155	68.4	1.40	28.4	116	74	257	61	196.00	5.40	1	1.00	1	1	1	0	0	0	1	0	0	0	1
0	0	80	2	128	45.2	2.00	27.5	122	57	186	64	122.00	5.60	1	1.00	1	0	1	0	1	0	0	0	0	1	1
1	1	63	2	157	59.2	-2.00	23.8	131	79	232	84	148.00	6.10	1	1.00	1	1	1	0	1	0	0	1	1	0	1
1	1	83	2	142	66.2	1.10	32.7	145	70	169	60	109.00	6.20	1	1.00	1	0	1	1	1	1	1	0	0	1	1
0	0	63	2	154	43.7	-.30	18.5	116	66	225	67	158.00	5.50	1	1.00	1	0	1	0	1	0	0	1	1	0	1
1	1	82	1	162	58.9	-1.80	22.5	157	83	183	44	139.00	5.70	1	1.00	1	1	1	1	1	1	1	1	0	1	1
0	0	79	1	160	67.6	.50	26.2	148	82	216	65	151.00	5.60	1	1.00	0	0	1	1	1	1	1	0	0	1	1
0	0	62	2	129	38.3	-.50	23.1	109	67	238	71	167.00	5.40	1	1.00	0	1	1	1	1	1	1	1	0	0	1
0	0	68	2	164	58.8	.50	21.9	144	79	203	62	141.00	5.50	1	1.00	1	0	1	1	1	1	1	1	0	1	1
0	0	95	2	120	32.5	.10	22.5	134	65	181	61	120.00	5.60	1	1.00	1	0	1	0	1	0	1	1	0	1	1
1	1	33	2	155	54.3	-.40	22.6	99	60	236	62	174.00	4.90	1	1.00	1	0	1	0	1	0	1	1	1	0	1
0	0	72	2	147	49.9	1.90	23.0	124	84	182	65	117.00	5.20	1	1.00	1	1	1	1	1	1	1	1	1	1	1
1	1	62	2	154	60.5	-1.50	25.6	153	91	206	41	165.00	5.30	1	1.00	1	0	1	0	1	1	1	0	0	1	1
0	0	35	2	150	47.4	.50	21.0	107	60	245	110	135.00	5.00	1	1.00	0	0	1	1	1	0	1	1	1	0	1
1	1	52	1	171	89.3	-1.60	30.5	123	78	270	59	211.00	5.40	0	1.00	0	1	1	0	1	1	1	0	1	0	1
1	1	67	1	165	64.6	3.20	23.6	130	70	185	72	113.00	5.90	1	1.00	0	0	1	0	1	0	1	1	0	1	1
1	1	74	2	145	55.2	-1.80	26.2	131	90	197	39	158.00	5.00	1	1.00	1	1	1	0	1	1	1	0	0	0	1
1	1	42	2	162	57.7	-3.70	21.9	175	94	208	77	131.00	4.90	1	1.00	1	1	1	1	1	1	1	1	0	1	1
1	1	67	1	168	61.9	-1.50	21.9	133	82	239	61	178.00	5.60	1	1.00	0	1	1	1	1	1	1	1	1	0	1
0	0	46	2	157	56.1	-1.50	22.7	127	77	260	56	204.00	5.00	0	1.00	1	0	0	1	1	1	1	1	1	0	1
1	1	62	1	168	78.4	-3.60	27.9	126	63	180	54	126.00	5.00	1	1.00	0	0	1	1	1	0	0	0	1	1	1
1	1	68	2	158	61.5	.50	24.6	109	59	221	100	121.00	5.60	1	1.00	1	0	1	1	1	1	1	1	1	0	1

1	1	66	2	157	84.8	3.70	34.3	127	69	213	52	161.00	6.40	1	1.00	1	0	1	1	1	0	1	0	0	1	1
1	1	68	2	153	55.7	1.20	23.8	116	66	248	88	160.00	5.50	1	1.00	1	0	1	0	1	1	0	1	1	0	1
1	1	72	1	167	69.9	2.40	25.1	118	70	214	73	141.00	5.30	1	1.00	0	0	1	1	1	1	1	0	1	0	1
1	1	78	1	168	64.5	2.30	22.9	140	77	136	59	77.00	6.80	1	1.00	0	0	1	1	1	1	1	1	0	1	0
1	1	71	1	168	81.8	-1.70	29.0	130	84	154	62	92.00	5.70	1	1.00	0	0	1	0	1	1	0	0	0	1	1
1	1	83	1	167	68.9	-4.00	24.5	128	75	169	53	116.00	5.70	1	1.00	0	1	1	1	1	1	0	1	0	1	0
1	1	75	2	149	54.7	.30	24.5	169	87	172	63	109.00	5.60	1	1.00	1	0	1	1	1	1	1	1	0	1	1
1	1	74	2	149	55.0	1.30	24.8	146	84	214	42	172.00	5.10	1	1.00	1	1	1	1	1	1	1	1	0	1	1
1	1	69	2	152	50.8	1.00	21.8	113	62	155	46	109.00	6.30	1	1.00	1	0	1	1	1	0	1	1	1	1	1
0	0	63	2	161	55.6	2.00	21.3	143	77	314	54	260.00	5.20	1	1.00	1	1	1	1	1	1	1	1	0	0	1
0	0	70	2	150	47.5	-2.30	21.2	113	65	206	54	152.00	5.80	1	1.00	1	0	1	0	1	1	1	1	1	0	1
1	1	62	2	152	49.6	-.10	21.5	138	71	275	80	195.00	6.00	1	1.00	0	0	1	1	1	1	1	1	1	0	1
1	1	60	1	179	84.8	-1.30	26.4	146	78	181	34	147.00	5.70	1	1.00	0	0	1	0	0	0	0	0	0	0	1
0	0	75	1	163	72.9	-.60	27.4	131	81	169	43	126.00	6.80	1	1.00	0	0	1	0	1	1	1	0	0	1	0
1	1	69	1	158	54.5	2.00	21.9	163	80	260	41	219.00	5.70	0	1.00	0	0	1	1	1	0	1	1	0	0	1
1	1	66	1	169	62.3	-.20	21.7	104	78	200	55	145.00	5.30	1	1.00	1	0	1	0	1	1	1	1	1	1	1
0	0	44	2	148	60.6	.50	27.5	123	82	251	61	190.00	5.20	1	1.00	0	0	1	0	1	1	1	0	1	0	1
0	0	76	2	149	51.7	1.00	23.2	146	69	211	64	147.00	5.30	1	1.00	1	1	1	0	1	0	1	1	0	1	1
0	0	52	1	161	62.0	2.00	23.8	98	66	248	63	185.00	4.80	1	1.00	0	0	1	0	0	0	1	1	0	0	1
0	0	55	1	173	85.1	4.90	28.3	148	86	190	57	133.00	6.10	1	1.00	1	0	1	1	1	1	1	0	0	1	1
1	1	55	2	154	51.0	-.70	21.4	150	97	207	85	122.00	5.40	1	1.00	1	1	1	0	1	0	1	1	0	1	1
0	0	62	2	154	46.5	1.30	19.6	91	57	224	90	134.00	6.10	1	1.00	1	0	1	0	0	0	0	1	1	0	1
0	0	69	2	143	40.8	1.70	19.8	113	68	249	91	158.00	6.00	1	1.00	1	0	1	0	1	0	1	1	1	0	1
0	0	42	2	159	63.5	.30	25.0	95	55	234	86	148.00	5.30	1	1.00	1	1	1	0	1	1	0	0	1	0	1
1	1	71	2	151	51.3	-1.20	22.4	117	73	218	75	143.00	5.30	1	1.00	1	1	1	1	0	0	1	1	1	1	1
1	1	60	1	167	60.6	-1.40	21.7	108	64	221	71	150.00	4.80	1	1.00	1	0	1	0	0	0	0	1	1	0	1
0	0	75	1	161	57.2	1.50	22.0	144	92	199	54	145.00	5.30	1	1.00	1	0	1	1	0	1	0	1	0	1	1
1	1	39	1	181	69.1	4.30	21.1	107	64	156	46	110.00	5.30	1	1.00	1	0	1	1	1	1	1	1	1	0	1
1	1	59	2	155	54.7	1.60	22.7	131	73	192	70	122.00	5.10	1	1.00	0	1	1	0	1	0	0	1	1	1	1
1	1	46	1	174	62.3	-1.90	20.6	100	61	186	44	142.00	5.40	0	1.00	0	1	1	0	1	0	0	1	1	1	1
1	1	45	2	176	59.2	-1.20	19.1	103	55	139	61	78.00	5.50	1	1.00	1	1	1	0	1	1	1	1	1	1	1
1	1	31	1	167	81.0	-7.30	29.0	104	65	187	46	141.00	5.20	1	1.00	1	1	1	0	1	1	1	0	1	1	1
1	1	77	2	154	46.2	-.40	19.3	114	55	198	40	158.00	5.30	1	1.00	1	0	1	0	1	0	0	1	0	1	1
0	0	74	2	142	51.9	-.90	25.8	137	78	187	62	125.00	5.10	1	1.00	1	0	1	0	1	0	0	0	1	1	1
1	1	74	1	167	62.0	.00	22.1	139	80	175	36	139.00	6.30	1	1.00	0	1	1	0	1	0	0	1	0	0	1
0	0	26	2	165	52.7	-.50	19.3	96	54	154	71	83.00	5.40	1	1.00	1	0	1	0	0	0	1	1	1	1	1
1	1	59	1	174	71.9	-1.20	23.7	126	84	254	58	196.00	5.90	1	1.00	0	1	1	0	1	0	1	1	1	0	0
0	0	64	1	169	67.7	-1.20	23.7	131	74	169	63	106.00	5.50	0	1.00	0	1	1	0	1	1	1	1	0	1	1
1	1	74	1	171	76.4	3.00	26.0	130	67	193	50	143.00	5.40	1	1.00	1	0	1	1	1	1	1	0	0	1	1

1	1	75	1	160	64.2	-1.00	24.9	110	59	120	35	85.00	5.40	1	1.00	1	1	1	0	1	1	1	1	0	0	1
1	1	71	2	148	54.2	.60	24.8	118	68	151	73	78.00	6.40	1	1.00	1	0	1	0	1	0	0	1	0	1	1
1	1	76	2	151	59.8	-4.00	26.2	155	94	206	81	125.00	5.40	1	1.00	1	0	1	1	1	1	1	0	0	1	1
0	0	64	2	158	64.4	-2.30	25.7	121	69	255	100	155.00	5.80	1	1.00	1	0	1	1	0	0	0	0	1	0	1
1	1	63	2	155	44.6	-.30	18.6	124	72	145	51	94.00	5.70	1	1.00	1	1	1	0	1	0	0	1	1	1	1
1	1	71	2	146	53.0	-.70	24.8	124	71	179	69	110.00	5.40	1	1.00	1	0	1	0	1	1	0	1	0	1	1
1	1	62	2	151	79.4	-1.70	34.8	132	71	218	64	154.00	5.50	1	1.00	1	0	1	0	0	0	0	0	0	1	1
0	0	69	1	165	73.2	-1.20	26.7	117	85	237	50	187.00	5.30	1	1.00	0	0	1	1	1	1	1	0	1	0	1
0	0	36	2	152	74.1	-2.60	31.9	114	72	145	75	70.00	4.70	1	1.00	1	1	1	0	1	0	1	0	1	1	1
1	1	60	2	158	61.3	-1.30	24.4	127	75	170	68	102.00	5.50	1	1.00	1	1	1	1	1	1	1	1	1	1	1
1	1	81	2	136	52.6	.10	28.5	111	66	207	66	141.00	6.30	1	1.00	1	0	1	1	1	1	1	0	0	1	0
1	1	77	1	158	56.9	-.60	22.6	127	78	192	58	134.00	6.20	1	1.00	0	1	1	1	1	1	1	1	1	1	1
1	1	72	1	151	58.5	-1.00	25.5	121	77	180	76	104.00	6.20	1	1.00	0	1	1	1	0	1	0	0	0	1	0
0	0	61	2	153	51.1	.70	21.8	121	61	286	71	215.00	6.40	1	1.00	0	0	1	0	0	1	1	1	1	0	1
1	1	59	2	170	69.6	7.30	24.0	131	72	255	69	186.00	5.80	1	1.00	1	1	1	0	1	0	1	1	0	0	1
1	1	60	2	154	51.6	-.10	21.7	134	73	248	85	163.00	5.40	1	1.00	1	1	1	1	0	0	1	1	1	0	1
1	1	66	2	151	51.7	-.70	22.7	132	81	215	65	150.00	5.40	1	1.00	0	0	1	1	1	1	0	1	1	1	1
1	1	76	2	150	68.7	.10	30.5	128	68	218	64	154.00	5.60	1	1.00	1	1	1	1	1	1	1	0	0	1	1
1	1	61	2	162	66.7	1.20	25.3	138	78	193	58	135.00	5.90	1	1.00	1	0	1	0	1	0	0	0	0	1	1
1	1	60	1	164	73.1	2.00	27.2	153	93	189	48	141.00	5.20	0	1.00	0	0	1	0	1	0	1	0	0	1	1
1	1	45	1	169	62.0	-.60	21.6	97	55	203	108	95.00	5.10	0	1.00	0	0	1	0	1	1	1	1	1	1	1
1	1	60	2	149	43.5	-1.00	19.6	111	62	168	68	100.00	5.40	1	1.00	1	0	1	0	1	0	1	1	1	1	1
0	0	71	1	163	67.0	-.80	25.2	162	80	163	50	113.00	5.50	0	1.00	0	0	1	0	0	0	0	0	0	1	0
1	1	61	2	153	56.5	-.80	24.1	107	70	201	70	131.00	5.80	1	1.00	1	1	1	1	0	0	0	1	1	1	1
1	1	71	2	148	55.5	-2.00	25.3	169	93	181	66	115.00	5.50	1	1.00	1	0	1	1	1	1	1	0	0	1	1
0	0	59	2	150	61.3	-1.20	27.1	107	72	271	62	209.00	5.70	1	1.00	1	0	1	1	1	1	1	0	0	0	1
1	1	54	2	159	51.2	-2.20	20.1	108	62	272	93	179.00	5.10	1	1.00	1	0	1	0	1	0	1	1	1	0	1
0	0	60	2	156	56.6	-3.40	23.1	109	67	219	103	116.00	5.80	1	1.00	1	1	0	0	0	0	0	1	1	1	1
0	0	72	2	147	52.2	-.60	24.1	135	65	245	54	191.00	5.50	0	1.00	1	1	1	1	1	0	0	1	0	0	1
1	1	67	1	170	84.3	-1.50	29.2	151	96	189	50	139.00	5.60	1	1.00	1	1	1	0	1	0	1	0	0	1	1
1	1	32	2	158	55.1	.30	21.9	117	68	230	73	157.00	5.10	1	1.00	1	1	1	1	1	1	1	1	1	0	1
1	1	75	2	152	50.0	.10	21.7	155	79	199	79	120.00	5.20	1	1.00	1	0	1	1	1	1	1	1	0	1	1
0	0	35	2	169	95.1	2.70	33.2	134	81	249	64	185.00	5.50	1	1.00	0	1	1	0	1	1	1	0	1	0	1
0	0	62	1	151	60.0	-.90	26.4	121	74	241	46	195.00	5.70	0	1.00	1	1	1	1	1	1	1	0	0	0	1
1	1	35	2	161	77.2	.70	29.7	121	69	198	72	126.00	6.20	1	1.00	1	0	1	1	1	1	1	0	1	1	1
0	0	70	1	165	67.6	2.10	24.7	150	80	428	37	391.00	5.20	1	1.00	1	0	1	1	1	1	1	1	0	0	1
1	1	74	2	145	48.9	2.00	23.2	148	81	193	72	121.00	5.60	1	1.00	1	0	1	1	1	0	1	1	0	1	1
1	1	45	1	172	66.8	.90	22.5	110	63	217	58	159.00	5.20	1	1.00	0	1	1	1	1	0	0	1	1	1	1
0	0	44	1	169	71.5	2.10	25.0	120	76	225	76	149.00	5.90	1	1.00	0	0	0	0	1	0	0	0	1	0	1

1	1	77	2	144	49.2	-.20	23.8	154	87	179	57	122.00	5.60	1	1.00	1	1	1	1	1	1	1	1	0	1	1
1	1	37	2	156	50.2	-1.30	20.7	114	76	213	64	149.00	5.40	1	1.00	1	0	1	0	1	1	1	1	1	1	1
0	0	59	2	148	47.5	1.70	21.7	131	81	240	76	164.00	5.10	1	1.00	1	1	0	1	0	0	1	1	0	0	1
0	0	72	1	158	66.2	2.20	26.5	119	75	240	60	180.00	5.30	1	1.00	1	0	1	1	1	1	1	0	1	0	1
0	0	37	2	160	46.4	1.70	18.0	94	64	226	82	144.00	5.10	1	1.00	1	0	0	0	0	0	1	1	1	0	1
1	1	68	1	164	59.8	-.60	22.3	109	70	222	73	149.00	5.10	1	1.00	0	0	1	1	0	1	1	1	0	0	1
1	1	24	2	159	49.6	-.50	19.6	110	64	162	65	97.00	4.90	1	1.00	1	0	1	1	1	0	0	1	1	1	1
0	0	76	2	151	61.2	-2.40	27.0	129	67	225	41	184.00	6.00	1	1.00	1	1	1	1	1	1	1	0	1	0	1
1	1	64	2	151	53.0	-1.50	23.1	141	71	214	51	163.00	6.30	1	1.00	1	0	1	1	1	1	1	1	0	1	1
1	1	77	2	154	55.0	.10	23.2	159	87	230	91	139.00	5.40	1	1.00	1	1	1	1	1	1	0	1	0	0	1
1	1	50	2	169	75.8	-4.50	26.6	111	71	229	72	157.00	5.00	1	1.00	1	1	1	1	1	0	1	0	1	0	1
1	1	70	2	139	48.2	-.80	24.9	118	78	221	62	159.00	5.90	1	1.00	1	0	1	1	1	0	0	1	1	0	1
1	1	62	2	158	65.6	-3.30	26.2	124	69	239	61	178.00	5.60	1	1.00	1	0	1	1	0	1	1	0	1	0	1
1	1	69	2	147	59.8	-2.10	27.7	193	102	260	59	201.00	6.10	1	1.00	1	0	1	1	1	1	1	0	0	0	1
0	0	53	2	151	60.3	-1.60	26.3	114	67	255	74	181.00	5.40	1	1.00	1	1	1	0	1	1	0	0	1	0	1
0	0	65	2	152	65.2	-1.10	28.1	148	92	217	107	110.00	5.60	1	1.00	0	0	0	1	0	0	0	0	0	1	1
1	1	70	2	159	58.7	-.80	23.1	155	82	218	88	130.00	5.50	1	1.00	0	1	1	1	1	1	1	1	0	1	1
0	0	66	2	146	48.6	.30	22.7	125	84	275	71	204.00	5.20	1	1.00	0	1	1	0	1	0	1	1	0	0	1
1	1	62	1	165	75.4	2.30	27.5	156	83	155	49	106.00	5.50	0	1.00	0	0	1	1	1	1	1	0	0	1	1
1	1	52	2	152	42.1	-.50	18.1	97	66	152	55	97.00	5.50	1	1.00	1	1	1	0	1	0	0	1	1	1	1
1	1	67	1	170	67.9	-.20	23.5	119	73	201	41	160.00	8.40	1	1.00	1	0	1	1	1	1	1	1	1	1	0
0	0	70	1	168	69.8	.40	24.7	152	80	186	33	153.00	5.30	1	1.00	1	0	1	0	1	0	1	1	0	0	1
0	0	68	2	156	54.1	1.10	22.3	115	66	183	74	109.00	5.60	1	1.00	1	0	1	1	1	1	1	1	1	1	1
0	0	57	1	170	66.5	-3.40	23.0	125	76	197	71	126.00	5.40	0	1.00	0	1	0	0	1	1	1	1	1	1	1
0	0	61	1	169	66.9	-.20	23.3	146	93	225	80	145.00	6.10	0	1.00	0	0	1	0	1	1	1	1	0	0	1
0	0	77	1	157	50.4	1.50	20.5	138	69	197	56	141.00	6.60	1	1.00	1	0	1	0	1	1	0	1	1	1	0
0	0	76	1	169	61.7	.10	21.6	133	73	257	102	155.00	4.90	1	1.00	0	0	1	1	1	0	0	1	0	0	1
0	0	64	1	160	59.8	-.80	23.3	130	78	254	50	204.00	5.70	1	1.00	1	1	1	0	1	0	1	1	1	0	1
1	1	64	2	160	58.2	-.60	22.6	143	85	202	71	131.00	5.30	1	1.00	1	0	1	1	1	1	0	1	0	1	1
0	0	62	2	146	53.4	.40	24.9	125	69	229	61	168.00	5.10	1	1.00	1	0	1	1	1	1	1	1	1	0	1
1	1	53	1	171	55.2	-.10	18.8	153	83	204	63	141.00	5.30	0	1.00	1	1	1	1	0	1	1	1	0	1	1
1	1	77	2	152	75.9	1.80	32.8	152	79	211	52	159.00	5.60	1	1.00	1	0	1	0	1	1	0	0	0	1	1
1	1	60	2	149	62.4	-1.40	28.2	106	73	278	65	213.00	5.60	1	1.00	0	1	1	1	1	1	1	0	1	0	1
1	1	71	2	153	73.2	-1.20	31.4	145	81	177	77	100.00	5.30	1	1.00	1	1	1	0	1	0	0	0	0	1	1
1	1	66	2	149	50.8	-1.80	22.8	145	88	229	82	147.00	5.50	1	1.00	1	0	1	0	1	1	1	1	0	0	1
1	1	69	2	151	49.3	3.20	21.7	142	86	216	69	147.00	5.40	1	1.00	1	0	1	1	1	0	0	1	0	1	1
1	1	80	2	146	47.0	-.90	22.1	149	74	206	46	160.00	5.70	1	1.00	1	1	1	1	1	0	1	1	0	1	1
1	1	65	2	156	62.7	.90	25.8	114	70	246	66	180.00	5.60	1	1.00	0	1	1	1	1	1	1	0	1	0	1
1	1	51	2	147	55.1	1.30	25.3	110	79	184	71	113.00	4.70	1	1.00	1	0	1	0	1	1	1	0	1	1	1

1	1	75	2	144	35.8	.90	17.1	149	87	239	61	178.00	5.60	1	1.00	1	1	1	1	1	1	1	1	0	0	1
1	1	67	1	167	65.1	-3.50	23.3	132	81	223	55	168.00	11.10	1	1.00	0	1	1	0	1	1	1	1	0	0	0
1	1	66	2	149	56.8	-.50	25.7	140	76	145	60	85.00	5.30	1	1.00	1	1	1	0	1	1	1	0	0	1	1
1	1	53	2	151	52.5	-1.00	23.0	149	82	257	116	141.00	5.10	1	1.00	1	1	1	0	0	1	1	1	0	0	1
1	1	58	2	163	78.8	3.80	29.6	118	70	205	55	150.00	5.30	1	1.00	1	0	1	1	1	1	1	0	1	1	1
0	0	66	2	150	59.1	-3.90	26.2	184	100	266	98	168.00	5.40	1	1.00	1	0	1	1	1	0	0	0	0	0	1
0	0	71	2	152	64.0	.90	27.7	130	69	195	75	120.00	4.90	1	1.00	1	0	1	1	1	0	0	0	1	1	1
0	0	56	1	168	67.0	3.70	23.8	154	98	218	49	169.00	5.20	1	1.00	0	1	0	0	1	1	1	1	0	1	1
1	1	76	1	156	58.3	.30	23.8	176	91	200	73	127.00	5.50	1	1.00	0	1	1	1	1	0	1	1	0	1	1
1	1	81	2	146	48.3	-.70	22.7	113	72	204	72	132.00	5.50	1	1.00	1	0	1	0	1	0	1	1	0	1	1
1	1	59	2	154	58.4	.30	24.5	159	93	221	78	143.00	5.10	1	1.00	1	1	1	1	0	1	1	1	0	0	1
1	1	63	2	144	52.3	-1.20	25.1	141	87	288	43	245.00	5.80	1	1.00	1	0	1	1	0	1	1	0	0	0	1
1	1	32	2	153	61.3	-.10	26.1	117	73	210	72	138.00	5.20	1	1.00	1	0	1	0	1	1	1	0	1	1	1
0	0	64	2	158	61.7	-2.10	24.8	163	100	198	73	125.00	5.60	1	1.00	0	1	1	1	0	0	0	1	0	1	1
0	0	79	1	159	65.9	.00	25.9	133	70	225	93	132.00	4.80	1	1.00	1	0	1	0	1	0	1	0	1	0	1
0	0	71	2	147	47.1	.30	21.8	141	86	190	102	88.00	5.80	1	1.00	1	0	1	0	1	1	1	1	0	0	1
0	0	70	1	156	50.8	.10	20.8	157	90	182	58	124.00	5.40	0	1.00	0	0	1	1	1	1	1	1	0	1	1
0	0	61	1	168	87.8	1.70	31.2	151	97	200	51	149.00	5.50	1	1.00	0	1	1	1	1	1	1	0	0	1	1
1	1	54	2	155	46.8	-.20	19.5	103	58	188	71	117.00	5.10	1	1.00	0	1	1	0	0	1	1	1	1	1	1
1	1	58	2	155	68.7	-2.90	28.7	121	76	173	64	109.00	5.70	1	1.00	1	1	1	0	0	0	1	0	1	1	1
1	1	57	1	162	47.0	.30	17.9	119	74	189	58	131.00	5.10	1	1.00	0	0	1	0	1	0	1	1	1	1	1
1	1	65	1	165	73.2	-1.80	27.0	130	79	213	37	176.00	5.50	1	1.00	1	0	1	0	1	0	1	0	0	0	1
1	1	32	1	160	66.1	-3.30	25.8	119	63	203	59	144.00	5.20	0	1.00	0	0	1	1	0	1	1	0	1	1	1
0	0	71	2	154	43.2	-1.80	18.2	103	61	167	64	103.00	5.20	0	1.00	1	0	1	0	1	0	0	1	0	1	1
0	0	66	2	150	56.3	.70	25.1	122	68	192	78	114.00	5.50	1	1.00	1	0	1	0	1	1	1	0	0	1	1
0	0	41	1	157	63.5	2.00	25.7	112	73	197	60	137.00	5.00	1	1.00	0	0	1	0	1	1	1	0	1	1	1
1	1	78	1	158	63.5	-.70	25.4	112	67	115	54	61.00	5.50	1	1.00	0	0	1	0	1	0	1	0	0	1	1
1	1	47	2	165	54.8	1.20	20.0	115	65	175	67	108.00	5.70	1	1.00	1	1	1	1	1	0	1	1	1	1	1
1	1	53	2	156	54.3	1.20	22.4	151	89	216	59	157.00	5.30	1	1.00	0	0	1	0	1	0	1	1	0	1	1
1	1	35	2	164	50.7	.30	18.8	100	59	189	64	125.00	5.30	1	1.00	1	1	1	1	1	1	1	1	1	1	1
0	0	62	1	167	69.8	1.50	25.1	127	84	207	62	145.00	5.70	0	1.00	0	1	1	0	1	1	1	0	0	1	1
1	1	61	2	150	53.3	-.60	23.5	108	61	227	56	171.00	5.90	1	1.00	1	1	1	1	1	0	1	1	1	0	1
1	1	46	2	157	69.4	-1.20	28.2	168	91	218	86	132.00	5.60	1	1.00	1	1	1	1	1	1	1	0	0	1	1
1	1	51	1	169	74.6	-1.90	26.0	132	87	198	66	132.00	5.90	1	1.00	0	1	1	0	1	1	1	0	1	1	1
0	0	42	2	154	63.7	1.60	26.7	152	86	224	75	149.00	5.20	1	1.00	0	1	1	0	0	0	0	0	0	0	1
1	1	66	1	169	62.5	-4.00	21.9	114	63	198	43	155.00	5.30	0	1.00	1	0	1	1	1	1	1	1	0	1	1
1	1	29	2	166	44.3	.70	15.9	110	64	181	87	94.00	4.80	0	1.00	1	0	1	0	0	0	1	1	1	1	1
0	0	65	2	159	52.5	-1.00	20.8	130	80	230	64	166.00	5.60	1	1.00	1	0	1	1	1	0	1	1	1	0	1
1	1	61	2	157	58.1	-7.50	23.5	112	66	197	61	136.00	5.60	1	1.00	1	0	1	0	0	0	1	1	1	1	1

1	1	61	2	148	51.2	-2.30	23.2	113	77	175	64	111.00	5.30	1	1.00	1	0	1	0	0	0	0	1	0	1	1
1	1	72	2	146	44.0	1.30	20.5	123	62	146	41	105.00	5.80	1	1.00	1	1	1	1	1	1	1	1	1	1	1
1	1	40	2	157	60.9	-2.70	24.8	114	71	180	41	139.00	5.00	0	1.00	1	0	1	1	1	0	1	1	1	1	1
1	1	39	1	163	65.0	-.50	24.5	113	69	172	62	110.00	5.10	0	1.00	0	0	1	0	1	0	0	1	1	1	1
0	0	37	2	154	48.1	-.20	20.2	92	61	166	59	107.00	5.60	1	1.00	0	0	1	1	0	1	1	1	1	1	1
1	1	60	1	160	60.0	-1.80	23.3	155	92	264	52	212.00	5.40	1	1.00	0	0	1	0	1	1	1	1	0	0	1
0	0	27	2	158	44.5	-.30	17.8	105	54	209	101	108.00	5.10	1	1.00	1	0	1	1	1	0	0	1	1	1	1
1	1	54	2	160	52.4	-.10	20.5	103	60	189	94	95.00	5.10	1	1.00	0	1	1	1	1	0	1	1	1	1	1
1	1	55	2	156	71.8	1.70	29.5	119	74	162	36	126.00	5.50	1	1.00	1	0	1	1	1	1	1	0	1	0	1
1	1	51	2	154	51.6	.50	21.7	101	67	248	71	177.00	5.70	1	1.00	1	0	1	1	1	1	1	1	1	0	1
1	1	73	2	143	56.5	-1.00	27.7	113	62	173	63	110.00	5.60	1	1.00	1	1	1	1	1	1	1	0	0	1	1
1	1	45	2	161	69.0	4.90	26.6	117	70	211	62	149.00	5.30	1	1.00	1	1	1	1	1	1	1	0	1	1	1
1	1	79	1	167	73.0	.30	26.1	106	64	246	68	178.00	5.50	1	1.00	1	1	1	1	1	1	1	0	0	0	1
1	1	72	2	151	43.7	-.70	19.2	102	59	142	38	104.00	5.70	1	1.00	1	1	1	0	1	1	1	1	1	0	1
1	1	69	1	160	65.8	-4.60	25.7	157	85	188	76	112.00	5.40	1	1.00	0	1	1	0	1	1	0	0	0	1	0
1	1	75	2	157	55.6	.50	22.5	155	87	138	54	84.00	5.70	1	1.00	1	0	1	1	1	0	1	1	0	1	0
1	1	67	1	158	68.1	-2.20	27.1	146	92	142	59	83.00	5.60	1	1.00	1	1	1	0	1	1	1	0	0	1	1
1	1	46	2	156	49.0	-.10	20.1	112	58	199	83	116.00	5.10	0	1.00	0	0	0	1	1	0	0	1	1	1	1
1	1	81	1	163	61.8	-1.20	23.3	123	81	205	86	119.00	5.10	1	1.00	0	1	1	1	1	0	1	1	0	1	1
1	1	68	2	149	60.4	1.70	27.2	136	78	164	76	88.00	5.50	1	1.00	1	0	1	0	1	1	1	0	0	1	1
1	1	63	2	142	47.8	-.80	23.8	123	77	228	52	176.00	6.10	1	1.00	1	1	1	1	1	0	1	1	1	0	1
0	0	25	1	173	78.2	-1.10	26.1	123	67	154	72	82.00	5.00	1	1.00	0	1	0	0	0	1	1	0	1	1	1
1	1	63	1	178	72.9	-.40	23.1	134	89	237	44	193.00	4.70	1	1.00	1	0	1	1	1	1	0	1	1	0	1
1	1	38	2	164	58.1	-2.60	21.6	101	54	173	68	105.00	5.40	1	1.00	1	0	1	1	1	1	1	1	1	1	1
1	1	65	2	159	85.2	1.50	33.7	137	70	224	64	160.00	5.80	1	1.00	1	1	1	0	1	1	1	0	1	0	1
1	1	74	2	150	55.7	1.20	24.7	109	63	163	60	103.00	5.90	1	1.00	1	0	1	0	0	1	1	1	1	1	1
1	1	65	1	157	62.3	.20	25.2	118	71	156	49	107.00	5.00	1	1.00	0	1	1	0	1	1	1	0	0	1	1
1	1	82	1	162	62.1	-2.00	23.7	121	66	195	59	136.00	5.80	1	1.00	0	0	1	1	1	1	1	1	1	1	1
1	1	53	1	161	67.2	.80	25.9	136	91	201	39	162.00	7.60	0	1.00	0	1	1	0	1	1	0	0	0	0	0
1	1	74	2	151	56.0	1.00	24.5	127	69	244	47	197.00	5.80	1	1.00	1	0	1	0	1	0	1	1	0	0	1
1	1	65	2	151	56.8	.20	24.9	121	74	162	71	91.00	5.70	1	1.00	1	1	1	1	1	1	1	1	0	1	1
1	1	69	2	146	47.7	-.20	22.4	124	76	238	81	157.00	5.60	1	1.00	1	0	1	1	1	1	1	1	1	0	1
1	1	43	1	177	80.2	-5.00	25.7	124	86	200	43	157.00	5.20	1	1.00	0	0	1	0	1	0	0	0	1	1	1
0	0	31	2	161	43.8	2.80	17.0	111	68	167	92	75.00	5.00	1	1.00	1	0	1	0	1	1	1	1	1	1	1
1	1	52	2	155	68.2	-7.60	28.4	151	84	295	78	217.00	5.90	1	1.00	0	0	1	0	1	0	0	0	0	0	1
0	0	64	1	162	67.9	-4.80	25.9	125	76	241	79	162.00	5.10	1	1.00	0	0	0	0	1	0	0	0	1	0	1
0	0	53	2	159	57.6	-1.60	22.8	128	73	245	54	191.00	5.40	1	1.00	1	1	1	1	1	1	1	1	1	0	1
0	0	87	1	165	63.1	-1.80	23.0	171	83	111	61	50.00	6.70	1	1.00	1	0	1	0	1	0	1	1	0	1	0
1	1	85	1	161	60.7	.30	23.4	112	64	184	56	128.00	5.80	1	1.00	1	0	1	1	1	1	1	1	0	1	0

1	1	61	2	152	48.7	-.10	21.2	88	58	195	86	109.00	6.00	1	1.00	1	1	1	1	1	0	0	1	1	1	1
1	1	67	1	159	62.3	1.40	24.6	156	101	215	50	165.00	5.20	1	1.00	1	1	1	0	1	1	1	1	0	1	1
0	0	75	2	144	40.5	.00	19.5	136	60	190	66	124.00	5.10	1	1.00	1	0	1	1	1	1	1	1	1	1	1
0	0	66	2	148	55.0	-.50	25.2	113	73	203	42	161.00	6.00	1	1.00	0	0	1	1	0	0	0	0	1	0	0
1	1	68	2	151	61.6	-1.10	27.0	134	79	150	31	119.00	5.60	1	1.00	0	0	1	0	1	0	0	0	0	0	1
1	1	62	2	156	49.8	.90	20.4	106	60	222	85	137.00	5.90	1	1.00	1	0	1	1	1	1	1	1	1	0	1
1	1	69	2	139	37.3	1.30	19.3	114	67	217	51	166.00	5.50	1	1.00	1	1	1	1	1	0	1	1	1	1	1
1	1	44	2	161	57.4	1.60	22.2	114	74	206	67	139.00	5.40	1	1.00	1	1	1	0	0	1	1	1	1	1	1
0	0	60	2	152	48.6	-4.60	21.1	113	64	232	79	153.00	5.20	1	1.00	1	1	1	0	0	0	0	1	1	0	1
1	1	42	2	156	68.0	-2.40	27.7	138	79	230	50	180.00	5.10	1	1.00	1	1	1	0	1	0	1	0	1	0	1
1	1	45	2	152	55.4	.90	23.9	134	70	178	51	127.00	5.20	1	1.00	1	0	1	0	1	1	1	1	1	1	1
1	1	51	2	157	49.9	-3.00	20.3	133	79	222	82	140.00	5.10	1	1.00	1	1	1	0	1	1	1	1	1	0	1
0	0	68	2	148	62.3	.90	28.5	123	80	193	60	133.00	5.40	1	1.00	1	0	1	1	1	1	1	0	1	1	1
1	1	56	2	159	52.8	-4.10	20.8	160	86	256	65	191.00	5.60	1	1.00	1	0	1	0	0	0	0	1	0	0	1
1	1	65	1	162	63.4	-2.50	24.1	127	80	206	56	150.00	5.20	1	1.00	1	0	1	1	1	1	1	1	0	1	1
1	1	73	1	160	58.9	-.70	23.0	110	72	181	74	107.00	6.50	1	1.00	1	0	1	1	1	1	1	1	1	1	0
1	1	82	2	152	64.8	1.10	27.9	103	59	217	57	160.00	5.90	1	1.00	1	0	1	1	1	1	1	0	0	1	1
1	1	79	1	153	51.3	-.80	22.0	130	69	178	43	135.00	5.90	1	1.00	1	0	1	0	1	1	1	1	1	1	1
0	0	70	2	154	49.7	-2.50	20.8	143	79	168	67	101.00	5.60	1	1.00	1	0	1	1	0	0	0	1	0	1	1
1	1	48	2	157	46.8	1.50	19.0	117	71	174	69	105.00	5.80	1	1.00	1	1	1	1	1	1	1	1	1	1	1
0	0	62	2	142	42.2	.50	21.0	131	75	246	74	172.00	5.60	1	1.00	1	0	1	0	1	1	0	1	1	0	1
1	1	73	1	161	75.8	-.30	29.3	159	99	190	116	74.00	4.70	1	1.00	0	1	1	1	1	1	1	0	0	1	1
1	1	62	2	158	56.6	.00	22.7	126	80	222	77	145.00	5.40	0	2.00	1	0	1	1	1	1	1	1	1	0	1
1	1	81	1	160	74.0	-.80	28.7	138	75	167	39	128.00	5.40	1	1.00	0	0	1	1	1	1	1	0	0	0	1
1	1	63	2	149	41.4	-.50	18.5	113	78	235	62	173.00	5.20	1	1.00	0	0	1	1	1	0	1	1	1	0	1
0	0	60	2	156	46.8	.60	19.2	121	82	178	71	107.00	5.60	1	1.00	1	1	1	1	0	0	0	1	1	1	1
0	0	73	2	153	54.2	-1.40	23.0	149	98	250	70	180.00	5.20	1	1.00	1	0	1	1	1	1	0	1	0	0	1
0	0	58	2	144	55.3	-.70	26.8	123	73	278	64	214.00	5.50	1	1.00	0	0	1	0	0	0	0	0	1	0	1
1	1	66	2	148	59.2	-1.50	27.1	113	63	163	43	120.00	5.50	1	1.00	1	1	1	1	1	0	1	0	1	1	1
0	0	61	2	152	37.0	.40	15.9	104	71	188	56	132.00	5.60	1	1.00	1	1	1	0	0	1	1	1	1	1	1
0	0	30	2	161	63.0	3.20	24.2	123	77	226	70	156.00	5.10	1	1.00	1	1	1	1	1	1	1	1	1	0	1
1	1	64	2	150	50.4	.60	22.3	149	93	194	46	148.00	5.30	1	1.00	1	0	1	1	0	1	1	1	0	1	1
1	1	81	2	147	63.9	-1.10	29.5	124	68	219	42	177.00	6.10	1	1.00	1	0	1	0	1	1	1	0	0	1	1
1	1	75	1	160	65.3	-2.40	25.6	124	72	192	49	143.00	5.80	1	1.00	1	1	1	0	1	1	1	0	1	1	0
0	0	44	2	157	66.7	-1.70	27.0	120	74	266	60	206.00	5.10	0	1.00	1	0	1	0	1	0	0	0	1	0	1
0	0	65	1	165	68.6	-1.10	25.0	139	98	237	78	159.00	5.70	1	1.00	0	1	1	1	1	1	1	0	0	0	1
0	0	48	1	166	54.2	1.30	19.6	110	74	192	52	140.00	5.50	0	1.00	0	1	1	0	1	1	0	1	0	1	1
0	0	30	2	169	65.4	-1.80	22.8	104	64	201	69	132.00	5.30	0	1.00	0	0	0	0	1	0	0	1	1	1	1
1	1	33	1	174	65.7	-.30	21.6	135	83	197	49	148.00	5.20	1	1.00	1	0	1	0	1	0	0	1	1	1	1

0	0	67	1	174	79.0	-4.60	26.1	137	85	226	48	178.00	8.60	1	1.00	1	1	1	0	1	1	1	0	1	0	0
1	1	43	2	155	61.2	-.30	25.6	117	61	177	72	105.00	5.10	1	1.00	1	0	1	1	1	1	1	0	1	1	1
1	1	65	2	152	60.1	-.10	26.0	118	72	199	44	155.00	5.80	1	1.00	1	1	1	0	1	0	1	0	1	1	1
1	1	77	1	159	59.1	-.20	23.4	110	64	204	37	167.00	5.10	1	1.00	1	0	1	1	1	1	1	1	1	0	1
1	1	74	2	139	45.8	-1.80	23.7	108	60	264	86	178.00	6.20	1	1.00	1	1	1	1	1	1	1	1	0	0	0
1	1	63	2	150	59.0	-2.20	26.3	115	62	269	43	226.00	5.70	1	1.00	1	1	1	1	1	1	1	0	1	0	1
1	1	55	2	151	47.8	-1.90	21.0	172	98	237	103	134.00	5.10	1	1.00	1	1	1	1	1	1	1	1	0	0	1
1	1	49	2	168	85.7	4.70	30.4	160	90	318	108	210.00	5.90	1	1.00	0	0	1	1	1	0	0	0	0	0	1
1	1	80	2	140	48.9	1.20	24.8	168	85	246	53	193.00	5.70	1	1.00	1	0	1	1	1	1	1	1	0	0	1
0	0	62	2	142	37.7	-1.00	18.6	150	89	260	68	192.00	5.70	1	1.00	1	1	1	1	0	1	1	1	0	0	1
1	1	74	2	149	45.9	3.00	20.7	109	68	178	63	115.00	5.70	1	1.00	1	0	1	1	1	0	0	1	0	0	1
1	1	68	2	150	52.4	-.60	23.2	132	72	218	52	166.00	5.70	1	1.00	1	1	1	1	1	1	0	1	1	1	1
1	1	59	2	153	60.5	-1.20	25.9	132	76	194	49	145.00	5.60	0	1.00	1	0	1	0	0	0	0	0	1	1	1
1	1	40	2	156	43.9	6.60	17.9	129	83	164	54	110.00	5.10	0	1.00	0	0	1	0	1	0	0	1	1	1	1
0	0	80	2	147	46.7	-1.30	21.7	107	62	238	59	179.00	5.60	1	1.00	1	0	1	0	1	1	1	1	0	0	1
1	1	58	2	164	71.6	-.10	26.5	139	76	196	57	139.00	5.30	1	1.00	1	0	1	1	1	1	1	0	1	1	1
0	0	67	2	150	63.8	-2.20	28.4	107	66	165	33	132.00	6.30	1	1.00	1	0	1	1	1	1	1	0	0	0	1
1	1	66	2	153	63.0	.50	26.9	135	88	230	73	157.00	5.20	1	1.00	1	0	1	0	1	1	1	0	0	0	1
1	1	66	2	147	42.1	2.00	19.5	139	82	252	59	193.00	5.90	1	1.00	1	1	1	0	1	1	0	1	1	0	1
1	1	78	1	165	57.2	.70	21.0	156	77	214	71	143.00	7.20	1	1.00	0	1	1	0	1	1	1	1	0	1	0
1	1	63	2	148	41.2	-1.80	18.8	128	77	223	88	135.00	5.80	1	1.00	0	0	1	1	1	0	0	1	1	0	1
0	0	67	2	138	38.5	-1.30	20.2	136	76	217	109	108.00	5.50	1	1.00	1	0	1	1	1	1	1	1	1	1	1
1	1	74	2	138	40.5	.50	21.2	153	82	209	71	138.00	5.60	1	1.00	1	0	1	1	1	0	0	1	0	1	1
0	0	66	1	171	65.6	2.70	22.3	156	91	175	41	134.00	5.40	0	2.00	1	0	1	1	1	1	1	1	0	1	1
0	0	63	1	167	69.2	-3.10	24.9	110	75	204	53	151.00	6.30	0	1.00	0	0	1	0	1	1	1	1	1	1	1
1	1	62	1	161	53.4	.30	20.5	179	98	237	71	166.00	5.50	1	1.00	0	1	1	1	1	0	0	1	0	0	1
0	0	80	1	158	59.1	-4.50	23.7	130	67	185	35	150.00	7.90	1	1.00	1	0	1	1	1	1	1	1	1	0	0
1	1	68	2	155	65.1	.80	27.0	122	67	213	60	153.00	5.30	1	1.00	0	0	1	1	1	1	1	0	1	1	1
1	1	67	2	153	43.8	-.10	18.7	142	92	223	65	158.00	5.50	1	1.00	1	0	1	1	1	1	1	1	0	0	1
1	1	70	1	166	60.6	-1.40	21.9	150	81	215	96	119.00	6.20	1	1.00	0	1	1	1	1	1	1	1	0	1	0
1	1	77	2	150	50.0	-2.40	22.2	130	86	207	66	141.00	5.40	1	1.00	1	0	1	1	1	1	1	1	0	1	1
1	1	61	2	142	40.7	.50	20.0	82	50	240	59	181.00	5.20	1	1.00	1	1	1	1	1	1	1	1	1	0	1
1	1	60	2	151	54.4	3.20	23.8	115	71	279	92	187.00	6.10	1	1.00	1	0	1	1	1	0	0	1	0	0	1
0	0	75	2	137	46.7	-2.80	24.7	143	81	153	60	93.00	5.50	1	1.00	1	0	1	1	1	0	1	1	0	1	1
0	0	67	2	147	45.4	-1.00	20.9	137	72	206	79	127.00	5.30	1	1.00	1	1	1	0	0	0	0	1	1	1	1
0	0	68	1	158	60.4	2.70	24.1	128	67	178	64	114.00	5.50	1	1.00	0	1	1	0	1	1	1	1	1	1	1
1	1	70	2	157	84.3	.50	34.2	102	62	159	39	120.00	6.10	1	1.00	1	0	1	1	1	1	0	0	0	0	1
1	1	64	1	165	66.7	1.50	24.4	116	75	161	53	108.00	5.90	1	1.00	0	0	1	1	1	1	1	1	0	1	0
1	1	72	2	150	52.5	.90	23.4	134	77	224	50	174.00	6.10	1	1.00	1	0	1	0	1	1	0	1	0	0	1

0	0	71	1	161	64.7	.10	24.9	106	66	189	39	150.00	6.40	1	1.00	1	0	1	0	0	0	1	1	1	0	1
0	0	64	2	157	52.1	4.90	21.0	143	93	212	67	145.00	5.60	1	1.00	1	0	1	0	1	0	0	1	0	1	1
1	1	71	1	167	73.7	.40	26.3	143	84	232	65	167.00	5.80	1	1.00	1	0	1	1	1	1	1	0	0	0	1
1	1	72	2	152	47.7	.20	20.7	109	61	239	95	144.00	5.60	1	1.00	1	0	1	0	1	0	0	1	1	0	1
0	0	45	2	158	56.2	3.80	22.6	115	72	224	65	159.00	5.30	1	1.00	0	0	1	0	1	0	1	1	1	0	1
0	0	65	1	165	65.7	.30	24.1	138	81	172	61	111.00	7.30	1	1.00	0	1	1	0	0	1	1	1	0	1	0
1	1	63	2	153	51.3	-.40	21.8	101	63	226	95	131.00	5.60	1	1.00	1	1	1	1	1	1	0	1	1	0	1
0	0	38	2	157	51.2	-1.40	20.6	92	57	188	77	111.00	5.60	1	1.00	0	0	1	1	1	0	1	1	1	1	1
0	0	54	2	158	68.1	2.30	27.3	130	86	226	63	163.00	5.80	1	1.00	0	0	1	0	0	0	0	0	1	0	1
0	0	72	2	147	43.2	.30	19.9	146	82	174	39	135.00	6.20	1	1.00	1	0	1	0	1	1	1	1	0	0	1
1	1	72	2	150	40.0	-1.10	17.8	174	94	206	65	141.00	5.30	1	1.00	1	0	1	1	1	1	1	1	0	1	1
1	1	75	2	148	56.0	.80	25.4	166	78	199	47	152.00	5.50	1	1.00	1	0	1	1	1	1	0	0	0	1	1
1	1	68	2	154	52.2	2.10	22.1	107	69	203	84	119.00	5.50	1	1.00	1	0	1	1	1	1	0	1	0	0	1
1	1	74	2	150	59.4	-1.70	26.4	129	69	202	54	148.00	5.80	1	1.00	1	1	1	1	1	1	1	0	0	1	1
1	1	81	2	147	69.5	-1.80	32.3	153	84	168	55	113.00	5.30	1	1.00	1	0	1	1	1	1	1	0	0	1	1
0	0	52	2	154	50.8	3.40	21.5	117	67	187	70	117.00	5.40	1	1.00	1	1	1	1	1	1	1	1	1	1	1
1	1	85	1	154	55.3	-1.90	23.3	131	79	155	50	105.00	6.30	1	1.00	1	0	1	1	1	0	1	1	1	1	0
1	1	73	1	158	52.1	-.90	20.7	154	85	166	56	110.00	5.50	0	1.00	0	1	1	1	1	1	1	1	0	1	0
0	0	73	2	156	64.4	.00	26.6	128	72	242	51	191.00	5.90	1	1.00	1	0	1	1	1	1	1	0	0	0	1
1	1	80	1	150	50.5	.10	22.5	142	69	196	67	129.00	5.70	1	1.00	0	0	1	1	1	1	1	1	0	1	0
1	1	70	2	159	56.9	.70	22.6	126	74	186	40	146.00	5.00	1	1.00	1	0	1	1	1	1	0	1	1	1	1
1	1	69	2	146	51.5	1.90	24.0	159	89	256	55	201.00	5.10	1	1.00	1	1	1	1	1	1	1	1	0	0	1
1	1	68	1	172	73.4	-.90	24.8	178	95	237	63	174.00	5.20	1	1.00	0	0	1	1	1	1	1	1	0	0	1
1	1	78	2	151	53.6	1.90	23.5	124	61	197	64	133.00	5.70	1	1.00	1	0	1	1	1	1	1	1	1	1	1
1	1	68	2	141	46.0	-1.00	23.0	119	69	190	63	127.00	6.10	1	1.00	1	0	1	1	1	1	1	1	0	1	1
1	1	87	1	162	56.1	.90	21.4	142	81	180	65	115.00	5.60	1	1.00	0	0	1	1	1	1	1	1	0	1	1
1	1	49	1	179	70.7	-.30	22.1	115	71	236	59	177.00	5.70	0	1.00	0	0	1	0	1	1	1	1	1	0	1
1	1	56	2	163	55.7	-.10	20.8	120	81	188	64	124.00	5.50	1	1.00	1	0	1	0	1	0	0	1	1	1	1
0	0	67	1	170	66.2	1.30	22.9	139	70	177	66	111.00	5.60	0	1.00	0	0	1	0	1	0	1	1	0	1	1
1	1	49	1	169	59.2	-4.60	20.8	106	63	286	36	250.00	4.90	0	1.00	1	0	1	0	0	0	0	1	1	0	1
1	1	72	2	152	56.0	3.00	24.2	90	55	171	66	105.00	5.70	1	1.00	1	0	1	0	1	1	0	1	1	1	1
0	0	34	2	142	49.7	-2.20	24.5	110	62	174	58	116.00	5.20	1	1.00	1	0	1	0	1	0	0	1	1	1	1
1	1	57	2	150	46.3	1.30	20.6	143	79	270	79	191.00	5.60	1	1.00	0	1	1	0	1	1	1	1	0	0	1
1	1	75	2	147	49.6	.20	23.0	108	65	168	56	112.00	6.50	1	1.00	1	1	1	0	1	0	0	1	1	0	0
1	1	72	2	154	47.6	-1.80	20.0	136	80	233	104	129.00	6.20	1	1.00	0	1	1	1	1	0	1	1	1	0	1
1	1	75	1	159	52.4	.40	20.6	129	78	188	72	116.00	5.50	1	1.00	0	1	1	0	1	1	1	1	1	1	1
1	1	72	2	146	53.2	.10	25.0	111	63	194	64	130.00	7.10	1	1.00	1	0	1	0	1	0	0	0	0	1	0
1	1	76	1	161	65.3	.30	25.3	96	69	199	37	162.00	5.60	1	1.00	1	1	1	0	1	0	0	0	0	0	1
1	1	38	2	163	61.6	-.50	23.2	106	66	185	54	131.00	5.60	1	1.00	1	0	1	0	0	1	1	1	1	1	1

0	0	77	2	144	42.2	1.40	20.3	89	58	192	70	122.00	5.60	1	1.00	1	0	1	0	1	0	0	1	1	1	1
1	1	52	2	162	58.0	1.40	22.2	120	76	294	61	233.00	5.70	1	1.00	1	1	1	1	1	1	1	1	1	0	1
0	0	75	2	140	44.3	-.70	22.7	134	69	255	52	203.00	6.80	1	1.00	1	0	1	0	1	1	1	1	0	0	0
1	1	39	2	165	57.0	5.80	20.8	96	61	210	93	117.00	5.30	1	1.00	1	0	1	0	1	1	1	1	1	1	1
0	0	50	2	159	51.7	1.70	20.4	116	67	237	71	166.00	5.70	1	1.00	1	0	1	0	0	0	0	1	1	0	1
0	0	43	2	161	51.0	6.40	19.7	96	55	188	79	109.00	5.50	1	1.00	1	0	1	0	0	0	1	1	1	1	1
1	1	78	1	153	59.9	1.40	25.5	137	70	196	41	155.00	5.30	1	1.00	1	1	1	1	1	1	1	0	1	1	1
0	0	50	2	159	53.3	.80	21.1	99	59	200	85	115.00	5.70	1	1.00	0	1	1	0	1	0	0	1	1	1	1
1	1	63	1	164	77.5	-1.30	28.7	123	91	219	44	175.00	5.20	1	1.00	0	0	1	0	0	0	0	0	0	1	1
1	1	75	2	140	54.1	1.50	27.8	101	62	214	70	144.00	5.60	1	1.00	1	0	1	0	1	1	1	0	0	1	1
0	1	54	2	153	56.0	2.10	24.0	111	63	268	92	176.00	5.70	1	1.00	1	0	1	1	1	1	1	1	1	0	1
0	0	55	2	148	54.0	.50	24.6	128	80	214	40	174.00	6.20	1	1.00	1	0	1	0	0	1	0	1	0	1	1
1	1	61	2	150	60.4	4.60	26.9	135	80	222	89	133.00	5.40	1	1.00	1	0	1	0	0	1	0	0	1	0	1
0	0	64	2	147	49.0	2.60	22.7	135	76	219	71	148.00	5.40	1	1.00	1	1	1	1	1	1	1	1	1	1	1
0	0	76	1	162	69.5	1.80	26.4	123	77	192	33	159.00	6.20	1	1.00	0	0	1	1	1	0	1	0	0	0	1
0	0	62	1	167	76.5	-3.80	27.3	133	102	176	38	138.00	6.50	1	1.00	1	0	1	0	0	1	1	0	0	0	0
0	0	83	1	161	49.0	5.40	18.9	121	65	216	57	159.00	6.50	1	1.00	1	0	1	1	0	0	0	1	0	1	0
0	0	59	1	157	61.0	1.50	24.7	134	87	214	63	151.00	5.30	1	1.00	0	1	1	0	1	0	1	1	0	1	1
0	0	75	2	145	51.9	1.80	24.6	123	62	180	39	141.00	5.80	1	1.00	1	0	1	0	1	1	0	1	1	0	1
0	0	56	2	154	47.7	1.70	20.2	137	88	258	103	155.00	5.50	1	1.00	1	1	1	0	1	0	0	1	1	0	1
1	1	60	2	150	44.5	1.40	19.8	126	87	213	65	148.00	6.00	1	1.00	1	0	1	0	1	1	0	1	1	1	1
1	1	62	1	163	73.1	.70	27.6	118	72	186	79	107.00	5.40	1	1.00	0	0	1	1	1	0	1	0	0	1	1
1	1	41	1	176	80.0	1.70	25.7	112	69	201	61	140.00	5.60	1	1.00	0	1	1	0	1	1	0	0	1	1	1
1	1	52	2	159	47.5	.30	18.9	105	56	208	87	121.00	5.50	1	1.00	1	1	1	1	1	1	1	1	1	1	1
1	0	81	2	142	51.9	2.50	25.8	125	58	177	36	141.00	6.40	1	1.00	1	0	1	1	1	1	1	0	0	0	0
1	1	43	2	151	50.5	.50	22.0	114	74	232	86	146.00	5.00	1	1.00	1	1	1	0	1	1	1	1	1	0	1
0	0	63	2	153	47.0	1.00	20.1	124	68	214	75	139.00	6.50	1	1.00	1	0	1	0	1	0	1	1	1	1	0
1	1	64	2	154	45.9	4.80	19.2	110	61	230	50	180.00	5.50	1	1.00	1	1	1	0	1	1	1	1	1	0	1
1	1	68	2	146	55.4	.80	26.0	130	77	219	41	178.00	6.80	1	1.00	1	1	1	1	1	0	1	0	0	1	0
1	1	74	1	160	51.2	.30	19.9	130	67	220	70	150.00	4.90	1	1.00	1	1	1	1	1	1	1	1	0	0	1
1	1	73	2	153	54.9	-1.00	23.5	145	72	283	43	240.00	5.20	1	1.00	1	0	1	0	0	1	1	1	0	0	1
0	0	70	2	150	59.5	-4.20	26.6	113	66	187	69	118.00	5.20	1	1.00	0	0	1	1	1	0	0	0	0	1	1
1	1	82	2	150	50.0	-.10	22.2	123	62	198	67	131.00	5.90	1	1.00	1	0	1	1	1	0	1	1	0	1	1
1	1	63	2	151	50.8	.20	22.2	151	86	192	60	132.00	6.00	1	1.00	1	0	1	0	1	0	1	1	0	0	1
1	1	72	2	153	53.0	1.40	22.7	123	76	204	53	151.00	5.00	1	1.00	1	0	1	0	0	0	0	1	0	1	1
1	1	58	1	169	75.7	.40	26.5	132	85	192	52	140.00	5.20	1	1.00	0	0	1	1	1	1	1	0	0	1	1
1	1	60	2	159	57.6	-2.50	22.8	122	76	169	57	112.00	5.50	1	1.00	1	0	1	0	0	1	1	1	0	1	1
1	1	59	1	168	60.2	.30	21.4	123	68	227	95	132.00	6.40	0	1.00	1	1	1	0	1	0	0	1	1	0	1
0	0	72	2	145	41.1	1.20	19.4	129	66	208	66	142.00	5.60	1	1.00	1	0	1	1	1	0	0	1	0	1	1

1	1	73	2	148	51.1	-1.20	23.2	142	80	201	68	133.00	5.70	1	1.00	1	1	1	1	1	1	1	1	0	1	1
1	1	73	2	147	47.9	-2.70	22.2	147	78	227	68	159.00	5.40	1	1.00	1	1	1	1	1	0	1	1	0	0	1
1	1	63	2	163	63.0	1.00	23.6	128	75	177	73	104.00	5.80	1	1.00	0	0	1	1	1	1	1	1	1	1	1
1	1	80	2	143	55.6	.60	27.1	142	66	198	73	125.00	5.50	1	1.00	1	0	1	0	1	1	1	0	0	1	1
1	1	55	1	163	63.0	-2.90	23.7	138	80	229	107	122.00	5.50	1	1.00	0	1	1	1	1	1	1	1	1	0	1
1	1	41	2	155	49.5	1.80	20.6	91	55	193	46	147.00	5.50	1	1.00	1	0	1	0	1	1	1	1	1	1	1
0	1	39	1	171	70.8	1.00	24.1	113	64	173	79	94.00	5.80	1	1.00	0	1	1	0	1	1	0	1	1	1	1
1	1	64	1	167	62.2	2.30	22.1	116	62	246	87	159.00	5.70	1	1.00	0	0	1	0	1	1	1	1	1	0	1
1	1	89	2	135	35.1	.10	19.2	156	66	220	67	153.00	5.50	1	1.00	1	1	1	1	1	1	1	1	0	0	1
0	0	74	2	148	37.6	2.00	17.2	126	79	197	87	110.00	5.90	1	1.00	1	0	1	0	1	0	0	1	0	0	1
1	1	67	1	168	55.2	-2.80	19.6	141	87	167	68	99.00	6.30	1	1.00	0	1	1	1	1	1	1	1	0	1	1
1	1	45	2	165	72.0	3.50	26.3	120	66	182	60	122.00	5.50	1	1.00	1	0	1	0	1	1	0	0	1	1	1
1	1	70	2	143	47.2	1.50	23.0	214	118	251	107	144.00	5.80	1	1.00	1	0	1	1	1	1	1	1	0	0	1
1	1	66	2	150	49.1	.40	21.8	133	82	209	52	157.00	5.70	1	1.00	1	1	1	1	1	1	1	1	1	1	1
1	1	71	1	163	71.5	-.30	26.7	112	70	269	55	214.00	6.50	1	1.00	0	0	1	1	1	1	1	0	0	0	0
1	1	59	2	153	78.0	-2.30	33.1	150	91	265	98	167.00	5.40	0	1.00	0	1	1	0	0	1	1	0	0	0	1
1	1	76	1	174	82.6	1.20	27.2	113	71	165	64	101.00	5.90	1	1.00	1	0	1	0	1	0	1	0	0	1	1
1	1	72	2	153	57.4	-2.00	24.3	132	72	158	49	109.00	6.10	1	1.00	1	0	1	1	1	1	1	1	0	1	1
1	1	63	2	150	49.3	2.50	21.8	103	69	220	76	144.00	5.80	1	1.00	1	0	1	0	1	1	1	1	1	0	1
0	0	76	2	151	43.7	.10	19.1	121	78	221	80	141.00	5.30	1	1.00	1	0	1	0	1	1	1	1	0	0	1
1	1	60	1	168	60.8	.90	21.5	184	105	301	65	236.00	5.40	1	1.00	1	1	1	1	1	1	1	1	0	0	1
1	1	62	2	151	56.8	.20	24.8	135	82	176	74	102.00	5.80	1	1.00	0	0	1	1	1	1	1	1	0	1	1
1	1	61	2	157	49.7	.50	20.2	129	72	206	71	135.00	5.50	1	1.00	1	1	1	0	1	1	1	1	1	1	1
1	1	75	1	161	72.6	-1.00	28.1	140	82	229	35	194.00	5.70	1	1.00	1	0	1	1	1	1	0	0	0	0	1
0	0	61	2	151	42.8	.40	18.7	118	62	169	55	114.00	5.60	1	1.00	0	0	1	1	1	0	0	1	1	1	1
1	1	75	2	141	41.2	-.10	20.8	110	60	171	61	110.00	6.30	1	1.00	1	0	1	0	1	0	0	1	1	1	1
1	1	49	1	161	59.1	-.10	22.6	138	85	170	85	85.00	5.60	1	1.00	0	0	1	1	1	1	1	1	0	0	1
1	1	69	1	157	59.9	1.70	24.3	127	79	213	49	164.00	6.10	1	1.00	1	1	1	1	1	1	1	1	0	1	1
0	0	70	2	152	73.8	1.20	31.9	115	75	217	47	170.00	6.00	1	1.00	1	0	1	0	1	0	0	0	1	1	1
1	1	67	1	158	59.2	-2.20	23.8	118	65	186	81	105.00	5.60	1	1.00	0	0	1	1	1	0	0	1	0	1	1
1	1	63	1	170	66.3	-1.00	22.9	137	79	200	59	141.00	5.50	1	1.00	1	0	1	1	0	1	1	1	1	1	1
0	0	66	2	148	48.7	.80	22.1	194	99	241	51	190.00	5.80	1	1.00	1	1	1	1	1	1	1	1	0	0	1
0	0	75	2	141	39.7	2.50	19.9	129	86	220	75	145.00	5.30	1	1.00	1	0	1	1	1	1	0	1	1	0	1
1	1	86	1	158	56.2	-1.90	22.5	134	74	242	71	171.00	5.50	1	1.00	1	0	1	1	1	1	1	1	1	0	1
1	1	60	2	147	47.7	.70	21.9	118	83	210	38	172.00	5.40	1	1.00	1	0	1	0	1	1	1	1	0	0	1
1	1	68	2	147	46.8	-.10	21.7	121	68	220	71	149.00	6.20	1	1.00	1	1	1	1	1	1	1	1	1	0	1
1	1	67	1	168	70.5	.00	24.9	115	76	163	57	106.00	5.90	1	1.00	1	0	1	0	1	1	1	1	1	1	1
0	0	42	1	166	64.2	1.50	23.2	122	74	237	57	180.00	5.80	0	1.00	0	1	1	0	1	0	0	1	1	0	1
1	1	29	1	186	83.1	6.20	24.1	114	59	194	44	150.00	5.30	0	1.00	0	1	1	0	1	1	1	1	1	1	1

1	1	44	1	165	99.7	-5.80	36.4	140	92	162	44	118.00	6.10	1	1.00	0	0	1	0	1	1	0	0	0	1	1
1	1	62	2	155	52.5	-.80	21.9	130	73	248	101	147.00	6.10	1	1.00	1	1	1	1	1	1	1	1	1	0	1
0	0	59	2	158	59.9	1.00	24.0	110	64	231	85	146.00	5.80	1	1.00	0	1	1	0	1	0	1	1	0	0	1
0	0	54	2	157	49.8	1.30	20.2	112	67	239	101	138.00	5.50	1	1.00	0	1	1	1	1	1	1	1	1	0	1
0	0	76	1	161	93.0	3.30	35.9	138	69	167	41	126.00	7.00	1	1.00	0	1	1	0	1	1	1	0	0	1	0
0	0	61	2	153	54.6	-.40	23.2	110	73	216	104	112.00	6.10	1	1.00	1	1	1	0	1	1	1	1	1	1	1
1	1	74	2	154	49.7	-1.20	21.0	140	73	263	32	231.00	5.20	1	1.00	1	0	1	0	1	0	0	1	0	0	1
0	0	44	1	182	69.2	-2.00	20.8	101	69	191	57	134.00	6.00	0	1.00	0	0	1	1	1	0	1	1	1	1	1
1	1	63	1	177	78.0	1.70	25.0	120	70	168	41	127.00	5.80	0	1.00	0	0	1	1	1	1	1	0	1	1	1
0	0	44	1	175	76.2	-.70	24.7	113	73	238	39	199.00	5.40	0	1.00	0	1	1	0	1	0	1	1	1	0	1
0	0	50	1	176	96.0	-3.10	31.1	124	77	184	56	128.00	5.70	1	1.00	0	0	1	0	0	1	0	0	0	0	1
0	0	61	2	159	51.1	-2.10	20.3	126	77	255	92	163.00	5.50	1	1.00	1	0	1	1	1	1	1	1	1	0	1
1	1	49	2	152	49.0	-2.00	21.1	94	56	199	73	126.00	5.70	1	1.00	0	0	1	1	1	1	1	1	1	1	1
0	0	69	2	145	48.2	2.00	22.9	142	83	223	45	178.00	5.60	1	1.00	1	0	1	0	1	1	0	1	0	0	1
0	0	81	2	152	50.9	-2.00	22.1	133	65	180	82	98.00	6.00	1	1.00	1	0	1	1	1	0	1	1	1	1	0
1	1	64	2	164	45.6	-2.40	16.9	112	60	247	55	192.00	5.40	1	1.00	1	0	1	1	1	1	1	1	1	0	1
1	1	70	1	169	79.1	.20	27.8	138	83	178	29	149.00	5.60	0	1.00	1	1	1	1	1	1	1	0	1	0	1
1	1	85	1	147	47.0	-3.90	21.7	137	66	182	86	96.00	7.20	1	1.00	0	0	1	1	1	1	1	1	1	1	0
0	0	57	2	151	54.9	.00	24.2	124	75	216	37	179.00	6.00	1	1.00	1	0	1	0	1	0	0	1	1	0	1
0	0	50	2	152	69.0	-2.60	29.9	148	84	262	68	194.00	4.40	1	1.00	1	0	1	0	1	1	1	0	0	0	1
1	1	73	2	150	51.1	-1.70	22.7	113	72	164	54	110.00	5.80	1	1.00	1	0	1	1	1	1	1	1	0	1	1
1	1	73	2	154	49.7	2.10	21.0	148	82	181	81	100.00	5.70	1	1.00	1	0	1	1	1	1	0	1	0	0	1
1	1	75	1	163	59.2	-1.30	22.2	144	79	153	59	94.00	6.40	1	1.00	0	1	1	0	1	1	1	1	0	1	1
1	1	75	1	160	56.3	2.60	22.0	139	78	231	162	69.00	6.70	0	1.00	0	0	1	0	0	1	1	1	0	0	0
1	1	77	1	168	63.2	1.90	22.4	118	74	182	55	127.00	5.30	1	1.00	0	0	1	0	1	0	1	1	1	1	1
1	1	55	1	162	61.3	.60	23.4	131	74	228	50	178.00	9.60	1	1.00	1	0	1	0	1	1	1	1	1	0	0
1	1	76	2	153	60.1	-.50	25.5	166	91	192	70	122.00	5.70	1	1.00	1	0	1	0	1	1	1	0	0	1	1
1	1	71	1	167	65.3	-1.50	23.4	142	84	227	45	182.00	9.70	0	1.00	0	0	1	1	1	1	1	1	0	0	0
1	1	75	2	143	42.5	1.20	20.6	121	73	262	119	143.00	5.10	1	1.00	1	0	1	0	1	0	1	1	1	0	1
1	1	61	1	168	59.3	2.40	21.0	115	75	232	61	171.00	4.80	1	1.00	0	0	1	0	1	0	0	1	1	0	1
1	1	64	1	160	61.5	.10	23.9	110	67	177	33	144.00	5.10	1	1.00	1	0	1	1	1	1	1	1	1	0	1
1	1	85	2	143	50.9	.20	24.9	137	74	204	69	135.00	5.80	1	1.00	1	1	1	1	1	1	1	1	1	1	1
1	1	59	2	151	51.6	.00	22.6	125	75	250	44	206.00	6.20	1	1.00	1	0	1	0	0	1	0	1	0	0	1
1	1	75	1	164	63.8	-1.60	23.7	114	75	157	68	89.00	5.20	1	1.00	1	1	1	0	0	1	1	1	1	1	1
1	1	76	2	148	42.4	.60	19.4	116	57	210	54	156.00	5.70	1	1.00	1	0	1	0	1	0	1	1	0	1	1
1	1	36	2	161	60.9	-5.20	23.4	119	67	160	64	96.00	5.20	1	1.00	1	1	1	1	1	1	1	1	1	1	1
1	1	69	1	146	50.9	.50	23.8	110	62	180	46	134.00	5.10	1	1.00	1	1	1	1	1	1	1	1	1	1	1
1	1	60	1	166	64.6	-1.50	23.4	153	104	229	70	159.00	5.60	1	1.00	0	1	1	0	1	1	1	1	0	0	1
1	1	75	2	136	35.8	-.90	19.3	114	75	203	69	134.00	5.80	1	1.00	1	0	1	1	1	1	1	1	1	1	1

1	1	61	2	148	49.2	-2.90	22.5	141	79	162	89	73.00	6.40	1	1.00	1	1	1	0	0	0	1	1	0	0	0
1	1	62	2	156	60.6	1.00	24.8	128	71	248	80	168.00	5.60	1	1.00	0	1	1	0	1	1	1	1	1	0	1
1	1	36	2	153	49.6	3.70	21.0	109	68	159	62	97.00	4.90	1	1.00	1	0	1	1	1	1	0	1	1	1	1
1	1	69	2	155	64.1	-3.60	26.6	156	75	233	54	179.00	5.50	1	1.00	1	1	1	1	1	1	1	0	0	0	1
0	0	72	1	169	68.6	.30	23.9	120	70	147	38	109.00	5.70	0	1.00	0	1	1	0	0	1	1	1	0	0	1
0	0	62	1	162	84.2	4.20	32.1	121	79	203	50	153.00	6.70	0	1.00	0	0	1	0	0	1	1	0	1	1	0
1	1	75	2	146	42.5	2.00	20.0	101	52	183	51	132.00	5.60	1	1.00	1	1	1	0	1	1	1	1	0	1	1
1	1	64	1	163	59.6	-1.90	22.5	163	89	194	59	135.00	5.30	0	1.00	0	0	1	1	1	1	1	1	0	1	1
0	0	72	2	155	76.1	2.60	31.5	107	63	193	82	111.00	6.10	1	1.00	1	0	1	1	1	0	1	0	0	1	1
1	1	62	2	143	60.9	-.90	29.6	123	82	182	61	121.00	5.50	1	1.00	1	0	1	0	1	0	1	0	1	1	1
1	1	50	1	171	68.6	.30	23.4	107	71	163	51	112.00	5.40	0	1.00	0	0	1	0	1	0	0	1	1	1	1
0	0	49	2	153	65.5	1.80	27.9	110	67	135	46	89.00	5.40	1	1.00	0	0	1	0	0	0	1	0	1	1	1
1	1	62	2	156	67.7	-1.30	27.9	140	84	184	33	151.00	5.80	1	1.00	1	0	1	0	0	1	0	0	0	0	1
0	0	77	2	152	45.2	4.00	19.5	129	71	194	82	112.00	6.30	1	1.00	1	0	1	1	1	0	0	1	1	1	0
0	0	51	2	153	82.7	-4.30	35.3	164	103	189	54	135.00	6.20	1	1.00	0	1	1	0	0	0	0	0	0	1	1
0	0	60	2	148	41.5	.00	18.9	106	65	234	85	149.00	5.10	1	1.00	0	0	1	0	1	1	0	1	1	0	1
1	1	60	2	153	69.7	.00	29.8	132	77	187	50	137.00	5.90	1	1.00	1	1	1	0	1	0	1	0	1	1	1
1	1	60	2	152	49.7	1.10	21.5	126	82	252	59	193.00	5.40	1	1.00	1	0	1	0	1	0	0	1	0	0	1
1	1	62	2	156	50.7	.60	20.9	113	64	178	67	111.00	5.70	1	1.00	1	0	1	1	1	0	0	1	1	1	1
1	1	63	2	156	57.7	-2.30	23.8	148	73	173	46	127.00	5.40	1	1.00	1	1	1	1	1	1	1	1	0	1	1
1	1	74	2	150	49.1	.70	21.9	143	80	291	49	242.00	6.60	1	1.00	1	0	1	1	1	1	0	1	0	0	0
0	0	41	1	170	65.1	-2.80	22.4	131	96	246	103	143.00	5.20	0	1.00	0	1	1	0	0	1	1	1	0	0	1
0	0	73	2	149	50.5	-.40	22.8	114	61	164	73	91.00	5.40	1	1.00	1	0	1	1	1	1	1	1	1	1	1
0	0	53	2	159	55.2	.00	21.8	104	59	178	91	87.00	7.30	1	1.00	1	0	1	0	1	1	1	1	1	1	0
1	1	70	2	152	68.0	1.50	29.4	112	64	154	78	76.00	5.10	1	1.00	1	0	1	1	1	1	1	0	0	1	1
1	1	66	2	148	54.6	-2.20	25.0	108	66	228	56	172.00	4.90	1	1.00	1	0	1	1	1	0	0	0	1	0	1
1	1	75	2	150	50.4	.00	22.3	130	67	232	54	178.00	5.90	1	1.00	1	1	1	1	1	1	1	1	1	0	1
1	1	61	2	146	45.2	2.80	21.1	143	80	179	56	123.00	5.40	1	1.00	1	0	1	1	1	1	0	1	0	1	1
1	1	77	2	137	53.8	-4.40	28.7	148	79	182	37	145.00	6.40	1	1.00	1	0	1	1	1	1	1	0	0	0	1
1	1	63	2	153	55.1	-2.60	23.6	120	70	213	69	144.00	5.20	1	1.00	1	0	1	1	1	0	0	1	1	1	1
1	1	72	2	147	47.8	-1.70	22.1	143	78	234	49	185.00	6.60	1	1.00	1	0	1	1	1	0	1	1	0	0	0
1	1	71	1	157	56.7	.90	23.0	101	56	183	72	111.00	5.20	1	1.00	0	1	1	1	1	1	1	1	0	1	1
1	1	62	2	147	53.3	3.00	24.6	110	58	161	49	112.00	5.50	1	1.00	1	0	1	1	1	0	0	1	1	0	1
1	1	77	2	145	67.5	-.60	32.2	128	72	200	65	135.00	6.10	1	1.00	0	0	1	1	1	1	1	0	0	1	1
1	1	45	2	154	61.6	.90	25.8	126	76	202	52	150.00	4.90	1	1.00	1	1	1	1	1	1	1	0	1	1	1
1	1	57	1	164	61.0	.60	22.6	117	71	177	45	132.00	5.30	0	1.00	1	0	0	1	1	1	1	1	1	1	1
1	1	34	1	165	77.2	2.70	28.3	153	88	149	47	102.00	5.60	1	1.00	1	0	1	0	1	1	1	0	0	1	1
1	1	59	2	149	44.2	-.50	19.8	128	83	235	93	142.00	6.40	1	1.00	1	0	1	0	1	0	0	1	1	0	0
1	1	79	2	144	43.8	-1.60	21.0	165	84	188	73	115.00	5.50	1	1.00	1	0	1	1	1	0	1	1	0	1	1

1	1	49	2	153	41.8	-.80	17.8	120	67	204	89	115.00	5.20	0	1.00	1	0	0	1	1	1	1	1	1	1	1
1	1	70	2	148	73.5	-4.40	33.6	139	64	156	58	98.00	8.50	1	1.00	1	0	1	0	1	1	1	0	0	1	0
1	1	80	2	150	56.9	2.00	25.2	127	74	249	101	148.00	5.50	1	1.00	1	0	1	0	1	1	1	0	0	0	1
1	1	63	1	160	71.6	-2.20	28.0	101	63	182	46	136.00	5.50	0	1.00	1	0	1	1	1	1	1	0	1	1	1
1	1	66	1	167	74.3	.00	26.8	122	77	232	61	171.00	5.80	1	1.00	0	0	1	1	1	1	1	0	1	0	1
0	0	40	2	162	57.1	.30	21.7	107	65	235	117	118.00	5.30	1	1.00	0	0	1	0	1	1	1	1	1	0	1
0	0	70	2	152	64.9	-2.70	28.1	145	81	155	56	99.00	5.50	1	1.00	1	1	1	1	1	1	1	0	0	1	1
1	1	78	1	160	67.1	-4.30	26.2	143	73	145	48	97.00	5.70	1	1.00	0	0	1	1	0	0	1	0	0	1	1
0	0	66	2	151	47.8	.80	20.8	144	73	194	82	112.00	5.30	1	1.00	1	0	1	1	1	1	1	1	0	1	1
1	1	45	2	161	56.8	5.60	21.8	132	72	216	58	158.00	5.10	1	1.00	1	1	1	0	1	1	1	1	1	1	1
1	1	70	2	143	55.9	-1.30	27.4	151	87	177	51	126.00	5.60	1	1.00	1	1	1	0	0	0	0	0	0	1	1
1	1	44	2	156	57.3	.90	23.6	105	61	236	74	162.00	5.10	1	1.00	1	0	1	0	1	0	1	1	1	0	1
0	0	55	1	170	69.6	-1.20	23.9	124	77	226	55	171.00	5.40	0	1.00	1	1	1	0	0	1	1	1	1	0	1
1	1	52	2	161	52.0	-1.10	19.9	125	70	162	64	98.00	4.60	1	1.00	1	0	1	0	1	1	1	1	1	1	1
1	1	73	2	153	56.4	2.70	24.1	150	73	222	64	158.00	5.20	1	1.00	1	0	1	1	1	1	0	1	0	0	1
0	0	34	2	155	50.0	-3.60	20.9	106	67	202	72	130.00	4.80	1	1.00	1	0	1	0	0	1	1	1	1	1	1
1	1	59	2	150	46.5	.50	20.8	146	82	270	113	157.00	5.20	1	1.00	1	0	1	0	1	0	1	1	0	0	1
0	0	66	2	156	53.3	.20	21.8	141	87	197	50	147.00	5.30	1	1.00	1	0	1	1	0	1	0	1	0	1	1
0	0	69	1	160	55.7	1.10	21.7	140	78	161	80	81.00	5.50	0	2.00	0	0	1	1	1	1	1	1	0	1	1
0	0	69	2	160	60.8	-4.40	23.8	148	74	185	68	117.00	5.90	1	1.00	1	0	1	0	1	0	1	1	0	1	1
1	1	28	2	156	55.0	-.90	22.7	132	80	205	80	125.00	5.00	0	1.00	0	1	0	0	0	0	0	1	1	1	1
1	1	75	2	157	80.0	-2.10	32.6	152	80	162	58	104.00	5.40	1	1.00	1	0	1	0	1	0	0	0	0	1	1
0	0	62	2	160	56.0	.30	21.8	105	66	198	47	151.00	4.80	1	1.00	1	0	1	1	0	0	0	1	1	1	1
1	1	72	1	158	50.7	-.90	20.4	113	76	150	66	84.00	5.10	1	1.00	0	0	1	1	1	0	1	1	1	1	1
1	1	59	1	169	64.9	-.20	22.8	127	75	206	70	136.00	7.20	1	1.00	1	0	1	1	1	1	1	1	1	1	0
0	0	64	1	169	65.8	-3.10	23.0	148	86	185	80	105.00	5.60	0	1.00	0	1	0	0	1	0	1	1	0	1	1
0	0	71	2	149	49.1	-2.60	22.2	135	76	200	33	167.00	6.00	1	1.00	1	1	1	1	1	1	1	1	0	0	1
0	1	87	1	160	63.0	-.90	24.5	160	87	178	64	114.00	5.10	1	1.00	0	1	1	0	1	1	1	1	0	1	1
0	0	70	2	150	55.6	-.50	24.8	145	76	187	32	155.00	6.50	1	1.00	1	0	1	0	1	0	0	1	0	0	0
1	1	49	1	175	79.1	.20	25.7	132	84	192	52	140.00	6.10	0	1.00	0	0	1	1	1	1	1	0	0	1	1
1	1	67	1	166	58.9	-1.90	21.5	117	69	217	69	148.00	6.50	1	1.00	1	1	1	1	1	0	1	1	0	1	0
1	1	63	1	168	75.2	-2.50	26.6	124	79	153	49	104.00	5.80	1	1.00	0	0	1	1	1	1	1	0	0	1	1
0	0	59	2	153	49.5	-3.20	21.0	106	64	207	66	141.00	5.30	1	1.00	1	0	1	0	0	1	1	1	1	1	1
1	1	77	2	145	49.1	1.20	23.3	131	70	223	63	160.00	5.60	1	1.00	1	0	1	0	1	0	1	1	1	0	1
1	1	70	2	147	56.8	1.10	26.4	140	77	204	53	151.00	5.60	1	1.00	1	0	1	0	1	0	1	0	0	1	1
0	0	56	2	154	68.0	1.00	28.5	143	86	194	49	145.00	5.60	1	1.00	1	0	1	0	0	1	1	0	0	1	1
1	1	58	2	152	64.3	-1.90	28.0	129	80	219	90	129.00	5.90	1	1.00	1	0	1	0	0	0	1	0	1	1	1
0	0	78	1	155	71.6	1.50	29.6	125	72	174	65	109.00	5.50	1	1.00	0	0	1	0	1	0	1	0	1	1	1
1	1	83	2	149	57.5	2.30	25.7	152	72	206	59	147.00	5.80	1	1.00	1	0	1	0	0	1	1	0	0	1	1

0	0	50	1	172	61.4	3.10	20.8	162	100	162	66	96.00	5.80	0	1.00	1	1	1	0	1	1	0	1	0	1	1
0	0	63	2	153	54.3	.50	23.1	125	63	180	72	108.00	5.20	1	1.00	1	0	1	0	1	0	0	1	1	0	1
1	1	68	2	150	63.3	.50	28.1	115	65	170	42	128.00	5.90	1	1.00	1	0	1	1	1	1	1	0	1	1	1
0	0	74	1	155	64.3	1.90	26.8	133	70	181	44	137.00	5.60	0	1.00	1	0	1	0	1	0	1	0	0	1	1
0	0	53	2	151	46.4	-.80	20.3	115	74	223	86	137.00	5.50	1	1.00	1	1	1	0	0	0	1	1	1	0	1
0	0	53	2	152	53.7	-7.10	23.2	126	85	199	66	133.00	6.40	1	1.00	1	0	1	1	0	0	1	1	1	1	1
1	1	49	2	161	70.0	2.60	27.1	117	64	230	87	143.00	5.40	1	1.00	1	1	1	0	1	1	1	0	1	0	1
1	1	80	2	144	44.5	-.90	21.4	105	55	202	57	145.00	5.60	1	1.00	1	0	1	0	1	0	1	1	1	1	1
0	0	43	2	157	57.7	1.80	23.4	126	67	136	50	86.00	5.00	1	1.00	1	0	1	1	0	1	1	1	1	1	1
0	0	36	1	171	75.0	-2.30	25.7	138	81	214	43	171.00	5.40	0	1.00	0	0	0	1	0	0	0	0	1	1	1
1	1	70	2	147	48.2	-.80	22.3	158	90	228	70	158.00	5.20	1	1.00	1	0	1	0	1	1	1	1	0	0	1
1	1	42	2	154	44.2	-.50	18.6	97	57	199	92	107.00	5.30	1	1.00	1	0	1	1	1	0	0	1	1	1	1
1	1	71	1	167	55.5	3.40	19.8	135	77	166	52	114.00	5.60	0	2.00	0	0	1	1	1	1	1	1	1	1	1
0	0	79	2	147	55.3	-1.50	25.7	108	57	186	70	116.00	6.10	1	1.00	1	0	1	1	1	0	0	0	0	1	1
1	1	58	2	156	53.0	-1.00	21.6	132	71	193	69	124.00	6.00	1	1.00	1	0	1	0	1	0	1	1	1	1	1
1	1	71	2	154	57.4	-.40	24.1	127	72	173	63	110.00	5.40	1	1.00	1	1	1	1	1	1	0	1	0	1	1
1	1	47	2	164	79.4	1.30	29.3	131	75	186	50	136.00	5.30	1	1.00	1	0	1	0	1	0	0	0	1	1	1
1	1	60	1	165	64.0	-1.90	23.5	110	63	229	64	165.00	5.30	1	1.00	0	0	1	1	1	1	1	1	1	0	1
0	0	28	2	165	61.6	-1.20	22.5	114	66	195	56	139.00	4.90	1	1.00	1	0	0	1	0	0	1	1	1	1	1
0	0	63	2	150	50.9	.20	22.7	113	69	230	86	144.00	5.70	1	1.00	1	1	1	0	1	1	1	1	1	0	1
1	1	72	2	147	57.3	-1.60	26.4	167	88	171	45	126.00	5.90	1	1.00	1	0	1	0	1	0	1	0	0	1	1
0	0	68	2	156	53.7	.60	22.0	155	83	214	71	143.00	5.60	1	1.00	1	0	1	1	0	0	1	1	0	1	1
1	1	72	1	158	60.8	-.80	24.3	142	80	193	42	151.00	5.80	1	1.00	1	0	1	1	1	1	1	1	0	1	1
1	1	74	1	159	60.7	-1.70	23.9	139	84	215	79	136.00	5.50	1	1.00	1	0	1	1	1	1	1	1	1	1	1
1	1	59	2	158	49.8	-3.50	20.0	150	77	256	90	166.00	5.40	1	1.00	0	0	1	0	0	1	1	1	0	0	1
0	0	61	1	155	45.2	-.30	18.8	127	84	264	93	171.00	5.80	0	1.00	1	1	1	1	1	0	1	1	0	0	1
1	1	58	2	157	59.1	-2.20	24.1	114	74	176	79	97.00	5.70	1	1.00	0	0	1	1	0	0	0	1	1	1	1
1	1	65	1	172	66.4	-.40	22.4	121	73	198	60	138.00	6.60	1	1.00	1	0	1	0	1	0	1	1	1	1	0
0	0	79	1	157	59.5	.80	24.1	146	80	191	76	115.00	5.40	1	1.00	0	0	1	1	1	1	1	1	0	1	1
1	1	71	1	160	68.2	.00	26.6	156	86	211	59	152.00	6.20	1	1.00	1	0	1	0	1	0	0	0	0	1	0
0	0	43	2	154	65.4	2.90	27.5	123	79	231	42	189.00	5.70	1	1.00	1	0	1	1	1	1	1	0	1	0	1
1	1	60	2	159	69.6	1.90	27.6	142	80	289	65	224.00	6.50	1	1.00	0	1	1	0	1	0	0	0	0	0	0
1	1	68	2	154	54.1	-.20	22.7	101	55	215	48	167.00	5.20	1	1.00	1	0	1	0	1	0	0	1	1	1	1
0	0	57	2	150	58.8	1.40	26.2	115	72	188	61	127.00	5.80	1	1.00	1	1	1	0	1	0	1	0	1	1	1
0	0	63	1	163	68.4	-.40	25.6	134	78	156	41	115.00	5.20	0	1.00	0	0	1	0	0	0	1	0	1	1	1
0	0	42	2	162	56.2	.70	21.4	109	64	182	70	112.00	5.50	1	1.00	1	0	1	1	1	1	1	1	1	1	1
1	1	71	1	159	60.3	-2.80	23.9	126	80	231	62	169.00	5.80	1	1.00	0	0	1	0	0	0	0	1	1	0	1
0	0	43	2	156	90.3	-.80	37.0	143	92	283	55	228.00	6.10	0	1.00	1	0	1	0	1	0	0	0	0	0	1
1	1	57	2	148	48.9	-2.00	22.4	106	59	240	72	168.00	5.60	1	1.00	1	0	1	1	0	1	0	1	1	0	1

1	1	41	1	170	80.0	-1.30	27.8	114	78	163	41	122.00	5.00	0	1.00	1	0	1	0	1	1	1	0	1	1	1
0	1	35	2	169	50.6	.10	17.6	110	70	198	104	94.00	5.30	1	1.00	0	0	1	1	1	0	0	1	1	1	1
0	0	35	2	160	79.8	-.10	31.2	128	83	183	55	128.00	5.10	0	1.00	0	1	1	0	0	0	1	0	1	1	1
1	1	60	2	150	47.6	-.90	21.0	126	72	242	88	154.00	5.50	1	1.00	1	0	1	0	1	0	1	1	1	0	1
0	0	72	1	173	68.2	-4.60	22.8	157	90	243	71	172.00	4.90	1	1.00	0	0	0	1	1	0	0	1	0	0	1
0	0	59	2	146	46.5	-1.50	21.7	110	72	265	62	203.00	5.80	1	1.00	1	0	1	0	0	0	0	1	1	0	1
1	1	36	2	162	51.3	1.20	19.5	123	72	201	67	134.00	5.30	0	1.00	0	0	0	1	1	1	1	1	1	1	1
0	0	63	1	167	73.8	6.80	26.5	123	86	182	51	131.00	5.40	1	1.00	1	0	1	1	1	1	1	0	1	1	1
1	1	79	1	163	57.3	2.00	21.5	110	61	165	61	104.00	6.00	1	1.00	1	0	1	1	1	0	0	1	1	0	0
1	1	71	1	159	60.5	.50	23.9	125	74	196	59	137.00	5.20	1	1.00	0	1	1	0	1	1	1	1	1	1	1
1	1	68	2	147	46.4	.10	21.5	136	76	239	81	158.00	5.90	1	1.00	1	1	1	1	1	1	1	1	1	0	1
0	0	70	2	153	53.2	-.40	22.7	125	79	210	64	146.00	5.80	1	1.00	1	0	1	1	1	1	1	1	0	1	1
1	1	58	1	175	74.4	3.20	24.2	115	64	234	75	159.00	6.10	1	1.00	0	0	1	0	1	1	1	1	0	0	1
1	1	62	1	166	81.3	2.50	29.6	121	71	206	70	136.00	5.80	1	1.00	0	0	1	0	0	0	1	0	0	1	1
1	1	76	1	160	64.1	.90	25.1	129	74	197	49	148.00	5.20	1	1.00	0	1	1	0	1	0	1	0	1	1	1
1	1	60	2	158	65.4	1.00	26.0	146	79	246	50	196.00	5.70	1	1.00	1	0	1	0	0	0	1	0	0	0	1
0	0	68	1	165	53.2	-.70	19.5	134	76	202	81	121.00	5.50	0	1.00	0	1	1	0	1	1	0	1	1	1	1
1	1	59	2	155	63.5	.50	26.2	137	82	221	48	173.00	5.80	1	1.00	1	1	1	1	1	1	1	0	1	0	1
0	0	62	2	146	56.8	3.10	26.7	116	86	262	94	168.00	5.70	1	1.00	0	1	1	0	0	0	0	0	1	0	1
1	1	61	2	148	52.2	.70	23.9	135	76	164	59	105.00	6.00	1	1.00	1	0	1	0	1	1	0	1	0	0	1
1	1	87	2	144	51.1	-1.80	24.5	159	88	213	61	152.00	5.70	1	1.00	1	1	1	1	1	1	1	1	0	1	1
1	1	62	1	169	69.5	.40	24.4	119	79	208	54	154.00	5.60	1	1.00	1	0	1	1	1	0	1	1	1	1	1
0	0	64	2	155	68.7	.90	28.5	169	93	240	47	193.00	6.20	1	1.00	1	0	1	1	1	1	1	0	0	0	1
0	0	69	1	158	61.7	-.60	24.7	137	81	276	87	189.00	5.80	1	1.00	0	0	1	1	1	1	1	1	0	0	1
0	0	67	2	150	56.6	1.60	25.2	141	72	226	102	124.00	5.50	0	1.00	0	0	1	0	1	1	0	0	0	0	1
1	1	58	2	156	50.4	-1.30	20.7	131	65	205	90	115.00	5.80	1	1.00	1	1	1	0	1	0	0	1	1	1	1
0	0	74	1	168	73.5	1.90	26.0	122	74	180	56	124.00	5.90	1	1.00	0	0	1	1	1	1	0	0	0	1	1
1	1	78	1	165	69.2	5.40	25.5	152	80	204	46	158.00	5.20	1	1.00	1	0	1	1	1	1	1	0	0	1	1
0	0	60	2	153	46.9	-1.30	20.0	103	56	253	69	184.00	5.90	1	1.00	1	0	1	1	1	0	0	1	1	0	1
1	1	55	2	157	45.2	-2.20	18.2	116	64	217	79	138.00	5.30	1	1.00	1	0	1	0	0	1	1	1	0	0	1
1	1	66	2	150	49.3	-1.80	21.8	132	76	206	47	159.00	5.60	1	1.00	1	0	1	0	1	0	0	1	0	1	1
1	1	61	1	158	69.8	-1.50	27.8	124	89	237	42	195.00	5.40	1	1.00	0	1	1	0	1	1	1	0	1	0	1
1	1	78	2	143	45.1	1.10	21.9	121	75	195	74	121.00	5.70	1	1.00	1	0	1	1	1	1	1	1	1	1	1
0	0	66	2	146	56.0	-.40	26.1	126	87	253	69	184.00	6.00	1	1.00	1	0	1	0	1	1	0	0	1	0	1
1	1	74	2	142	56.4	1.00	27.8	114	71	214	57	157.00	6.30	1	1.00	1	1	1	0	1	0	1	0	1	1	1
0	0	71	1	164	65.5	.30	24.4	116	66	224	52	172.00	6.00	1	1.00	1	1	1	0	1	0	1	1	1	0	1
1	1	46	1	171	61.2	1.30	20.9	112	74	138	79	59.00	5.30	1	1.00	0	1	1	1	1	1	1	1	1	1	1
0	0	78	2	150	73.2	1.30	32.3	126	77	223	72	151.00	5.30	1	1.00	1	0	1	1	1	1	1	0	0	0	1
1	1	70	1	166	58.3	-2.10	21.1	121	74	247	113	134.00	5.40	1	1.00	0	0	1	1	1	1	1	1	1	0	1

0	0	69	1	158	48.3	.60	19.3	133	86	205	72	133.00	5.70	0	1.00	1	0	1	0	1	1	1	1	1	1	1
1	1	78	2	150	44.3	-1.40	19.8	127	67	246	90	156.00	6.70	1	1.00	1	0	1	1	1	0	0	1	0	0	0
0	0	79	2	134	38.4	1.10	21.5	143	69	165	38	127.00	5.90	1	1.00	1	1	1	0	1	0	1	1	0	0	0
0	0	43	2	157	57.8	2.80	23.4	115	68	229	77	152.00	5.20	1	1.00	1	0	1	0	0	0	0	1	1	0	1
1	1	72	2	149	49.1	.00	21.9	122	69	192	79	113.00	5.50	1	1.00	1	0	1	1	1	0	0	1	0	1	1
0	0	84	2	150	48.4	2.70	21.4	172	106	233	73	160.00	5.20	1	1.00	1	1	1	0	0	0	0	1	0	0	1
1	1	67	2	141	52.2	-1.60	26.4	127	77	195	54	141.00	5.60	1	1.00	1	0	1	1	1	1	0	0	1	1	1
1	1	56	2	155	65.3	.20	27.1	123	72	202	40	162.00	5.60	1	1.00	1	0	1	0	1	0	0	0	1	1	1
0	0	73	2	148	49.5	1.70	22.5	140	76	192	54	138.00	5.50	1	1.00	1	0	1	1	1	1	1	1	0	1	1
1	1	68	1	167	72.2	-.50	25.8	142	82	248	67	181.00	5.40	1	1.00	0	0	1	1	1	0	1	0	0	0	1
1	1	78	2	147	41.7	.00	19.2	117	72	168	60	108.00	5.10	1	1.00	1	1	1	0	1	0	1	1	1	1	1
1	1	62	2	154	42.1	-2.70	17.7	122	80	225	89	136.00	5.70	0	2.00	1	0	1	0	1	0	0	1	0	0	1
1	1	73	1	157	50.7	-2.40	20.7	83	48	186	61	125.00	5.80	1	1.00	1	0	1	0	1	1	1	1	0	1	1
1	1	60	2	150	44.9	-1.80	20.0	104	62	183	79	104.00	5.70	1	1.00	1	0	1	1	1	0	1	1	1	1	0
1	1	78	2	156	59.7	-2.80	24.6	135	77	183	31	152.00	5.50	1	1.00	1	0	1	1	1	1	1	1	1	0	1
1	1	61	1	154	56.3	.40	23.6	112	71	219	49	170.00	5.80	0	1.00	1	0	1	0	1	0	1	1	1	1	1
1	1	70	2	144	54.6	4.40	26.5	103	63	237	74	163.00	5.90	1	1.00	1	0	1	1	1	1	0	0	0	0	1
1	1	66	2	154	55.1	-3.30	23.2	145	85	217	74	143.00	5.90	1	1.00	1	0	1	0	1	1	1	1	0	1	1
1	1	78	2	145	41.9	1.50	19.9	165	86	212	54	158.00	6.00	1	1.00	1	0	1	1	1	1	1	1	0	1	1
1	1	50	1	163	68.1	-.60	25.5	137	94	242	56	186.00	5.50	1	1.00	0	1	1	1	1	1	1	0	0	0	1
1	1	81	2	138	42.0	3.80	22.0	149	70	232	54	178.00	5.90	1	1.00	1	0	1	1	1	0	1	1	0	0	0
1	1	75	1	171	68.7	1.20	23.5	121	75	226	60	166.00	6.30	1	1.00	1	1	1	1	1	1	1	1	1	0	1
0	0	68	2	141	57.7	-.50	29.1	125	68	223	102	121.00	5.90	1	1.00	1	0	1	0	0	0	0	0	0	0	1
1	1	71	2	148	48.7	.90	22.3	115	81	219	67	152.00	4.90	1	1.00	1	0	1	0	1	0	1	1	1	1	1
1	1	68	2	148	37.4	-1.90	17.1	132	78	177	97	80.00	6.20	1	1.00	1	0	1	1	1	1	1	1	1	1	0
1	1	76	2	150	50.8	.70	22.4	162	88	195	73	122.00	5.20	1	1.00	1	0	1	1	1	1	1	1	0	1	1
1	1	72	2	143	46.8	-.40	22.8	135	68	232	67	165.00	5.50	1	1.00	1	0	1	0	1	1	1	1	1	0	1
1	1	51	2	159	57.6	1.00	22.7	127	70	203	64	139.00	5.60	1	1.00	1	0	1	1	0	0	1	1	1	1	1
1	1	57	2	141	42.1	-1.50	21.1	132	71	237	63	174.00	5.30	1	1.00	1	0	1	0	0	1	0	1	1	0	1
1	1	84	1	150	69.3	-.80	30.8	128	79	191	47	144.00	5.20	1	1.00	0	0	1	1	1	1	1	0	1	1	1
1	1	74	2	141	54.9	-.50	27.6	131	69	161	60	101.00	6.10	1	1.00	1	1	1	1	1	1	1	0	0	1	1
0	0	67	2	155	45.1	5.90	18.7	106	69	253	95	158.00	5.40	1	1.00	1	0	0	0	1	0	0	1	1	0	1
1	1	68	2	156	63.6	-1.10	26.2	112	58	214	81	133.00	5.30	1	1.00	1	0	1	1	1	1	1	0	1	1	1
1	1	47	2	149	46.7	-.40	20.9	99	57	175	62	113.00	5.00	1	1.00	1	0	1	0	1	1	1	1	1	1	1
1	1	71	1	168	61.7	3.00	21.8	138	77	181	62	119.00	5.40	1	1.00	0	1	1	1	1	0	1	1	1	1	1
1	1	63	2	149	54.4	-.90	24.5	120	70	230	51	179.00	5.60	1	1.00	1	0	1	0	1	0	1	1	0	0	1
1	1	64	2	149	57.3	1.30	25.8	128	75	210	52	158.00	5.50	1	1.00	1	0	1	0	1	1	0	0	1	1	1
1	1	76	1	165	60.9	-3.10	22.4	177	106	150	34	116.00	5.60	1	1.00	0	0	1	0	1	1	1	1	0	0	1
1	1	72	1	165	67.4	-.50	24.8	96	58	141	40	101.00	5.80	1	1.00	1	1	1	0	1	0	0	1	1	1	0

1	1	69	2	151	43.2	-.40	19.0	140	73	207	64	143.00	5.40	1	1.00	1	0	1	1	1	0	1	1	0	1	1
1	1	74	2	151	48.1	2.50	21.0	146	90	225	71	154.00	6.00	1	1.00	0	0	1	0	1	0	0	1	0	0	1
0	0	87	2	147	53.7	.10	24.8	107	53	222	55	167.00	6.20	1	1.00	1	0	1	1	1	1	1	1	0	0	0
0	0	19	2	160	64.8	-4.50	25.3	107	56	238	80	158.00	5.60	1	1.00	1	0	1	0	1	0	0	0	1	0	1
1	1	82	1	152	41.5	-.30	18.0	118	71	181	72	109.00	6.10	1	1.00	0	0	1	0	1	0	1	1	1	1	1
0	1	78	2	146	56.4	4.10	26.4	111	77	177	49	128.00	5.70	1	1.00	1	0	1	0	1	0	0	0	0	1	1
1	1	76	1	162	66.8	.50	25.5	124	70	229	36	193.00	5.60	1	1.00	1	0	1	1	1	1	1	0	1	0	1
1	1	63	2	147	60.9	-1.70	28.2	132	86	200	60	140.00	7.40	1	1.00	1	0	1	1	1	1	0	0	0	1	0
0	0	78	2	138	30.4	-.60	15.9	107	58	212	66	146.00	5.80	1	1.00	1	0	1	1	1	0	0	1	0	1	1
1	1	69	1	167	69.8	1.70	24.9	138	87	268	57	211.00	6.00	1	1.00	0	0	1	0	1	1	1	1	0	0	1
0	0	67	1	171	70.0	-.90	23.8	131	91	131	50	81.00	5.30	1	1.00	0	0	1	0	1	0	1	1	0	1	1
1	1	63	2	147	58.2	3.60	26.7	128	85	180	73	107.00	5.90	1	1.00	1	0	1	1	1	0	0	0	1	0	1
0	0	72	1	160	61.9	1.70	24.3	103	57	143	43	100.00	6.70	1	1.00	1	0	1	0	1	0	0	1	0	1	0
0	0	76	2	152	63.3	.60	27.5	151	83	229	75	154.00	5.10	1	1.00	1	0	1	0	1	1	1	0	0	0	1
0	0	54	1	160	76.0	-5.50	29.5	128	70	205	67	138.00	5.70	1	1.00	0	0	0	0	1	0	0	0	0	1	1
0	0	51	2	154	51.3	-.70	21.5	135	74	229	61	168.00	5.40	1	1.00	1	1	1	1	1	1	0	1	1	0	1
1	1	67	2	145	51.7	1.90	24.5	109	57	226	60	166.00	5.70	1	1.00	1	1	1	1	1	1	1	1	1	0	1
0	0	71	2	154	65.9	4.60	27.8	127	67	184	72	112.00	6.00	1	1.00	1	0	1	0	1	0	0	0	0	1	0
1	1	79	2	141	37.1	1.00	18.6	136	82	216	61	155.00	6.00	1	1.00	1	1	1	1	1	0	0	1	1	1	1
0	0	44	2	152	61.1	-.60	26.4	89	56	259	76	183.00	5.50	1	1.00	1	0	1	0	1	1	1	0	1	0	1
1	1	62	2	151	59.8	1.40	26.2	121	69	314	47	267.00	5.90	0	1.00	1	0	1	0	1	1	1	0	1	0	1
1	1	51	2	157	46.7	.00	18.9	134	81	300	62	238.00	5.30	1	1.00	1	1	1	0	0	0	1	1	1	0	1
1	1	61	2	161	55.2	2.60	21.2	99	67	199	72	127.00	5.50	1	1.00	1	0	1	1	1	1	1	1	1	1	1
0	0	80	2	138	35.9	3.10	18.9	138	81	228	66	162.00	5.70	1	1.00	1	0	1	1	1	1	0	1	0	0	1
1	1	61	2	150	48.0	2.40	21.4	158	92	277	76	201.00	8.20	1	1.00	1	0	1	0	1	1	1	1	0	0	0
1	1	41	2	160	58.8	-5.40	23.0	124	86	176	62	114.00	5.60	1	1.00	1	0	1	0	1	1	1	1	1	1	1
1	1	70	2	164	65.5	.60	24.2	140	83	177	51	126.00	5.60	1	1.00	0	0	1	1	1	1	1	1	0	1	1
1	1	73	1	166	84.7	1.30	30.8	105	70	185	52	133.00	5.90	1	1.00	0	1	1	1	1	1	1	0	0	1	1
0	0	74	1	171	66.3	5.70	22.7	115	82	168	57	111.00	6.00	0	1.00	0	1	1	0	0	0	1	1	1	1	1
0	0	59	2	157	55.9	-4.10	22.7	153	83	220	56	164.00	6.80	1	1.00	1	0	1	0	1	0	0	1	0	0	0
0	0	69	1	160	71.0	1.10	27.5	144	77	135	55	80.00	5.90	1	1.00	0	0	1	0	1	1	1	0	0	1	1
0	0	55	2	163	74.3	.20	28.0	118	72	237	49	188.00	6.30	1	1.00	0	0	1	1	0	1	0	0	1	0	1
1	1	66	2	158	63.5	1.80	25.4	97	63	236	75	161.00	5.60	1	1.00	1	1	1	1	1	1	1	0	1	0	1
1	1	71	2	151	49.9	-.50	21.7	155	78	255	87	168.00	6.30	1	1.00	1	0	1	1	1	0	0	1	0	0	1
1	1	65	1	162	66.2	2.20	25.2	104	65	210	61	149.00	6.40	0	1.00	0	1	1	1	1	1	0	0	1	1	1
0	1	40	1	162	53.8	3.10	20.6	119	76	248	82	166.00	5.60	0	2.00	0	0	1	1	1	0	0	1	1	0	1
1	1	80	1	165	76.0	-2.30	28.0	125	71	208	63	145.00	5.60	1	1.00	1	1	1	1	1	1	1	0	0	1	1
1	1	58	2	157	50.6	-3.40	20.4	147	82	187	80	107.00	5.40	1	1.00	1	0	1	0	0	0	0	1	0	0	1
1	1	65	1	160	63.3	-1.80	24.7	128	79	198	88	110.00	7.30	0	2.00	0	0	1	0	1	1	1	1	1	1	0

1	1	80	1	159	48.8	-.80	19.3	95	57	172	69	103.00	5.80	1	1.00	0	0	1	0	1	0	1	1	1	1	1
1	1	62	2	147	49.8	1.30	23.0	139	76	268	54	214.00	5.30	1	1.00	1	0	1	0	1	1	0	1	1	0	1
1	1	69	1	160	61.5	.50	24.1	125	74	177	69	108.00	6.30	1	1.00	0	0	1	1	1	0	1	1	0	1	1
1	1	24	1	179	106.1	1.40	33.0	163	86	209	44	165.00	5.60	1	1.00	1	0	1	1	1	1	1	0	0	1	1
0	0	50	1	176	64.4	5.20	20.7	115	75	220	78	142.00	5.00	1	1.00	0	0	1	0	1	0	1	1	1	0	1
0	0	39	2	164	56.5	1.50	20.9	98	55	202	84	118.00	5.00	1	1.00	1	0	1	1	1	1	1	1	1	1	1
1	1	57	2	156	60.2	-1.00	24.8	103	58	201	50	151.00	5.50	1	1.00	1	0	1	0	0	1	0	1	1	1	1
1	1	73	1	155	56.8	.10	23.7	114	65	152	63	89.00	5.70	0	1.00	0	0	1	1	1	0	0	1	0	1	1
0	0	79	2	151	43.8	5.30	19.1	125	68	221	74	147.00	5.50	1	1.00	1	0	1	1	1	1	0	1	1	0	1
1	1	75	1	158	55.7	-2.10	22.3	128	77	196	52	144.00	5.70	1	1.00	1	0	1	0	1	0	1	1	0	1	1
1	1	81	2	142	42.3	.30	20.8	140	71	167	66	101.00	5.20	1	1.00	1	1	1	1	1	0	1	1	0	1	1
1	1	81	2	142	52.5	-.20	25.9	136	78	173	48	125.00	5.80	1	1.00	1	0	1	1	1	1	1	0	0	1	1
1	1	81	1	154	58.4	-.90	24.5	102	57	223	57	166.00	5.70	1	1.00	1	0	1	1	1	1	1	1	1	0	1
1	1	72	1	167	63.2	4.80	22.6	125	71	147	42	105.00	6.10	1	1.00	1	0	1	1	0	1	1	1	1	1	1
1	1	59	1	164	65.5	.80	24.2	132	79	185	44	141.00	5.30	0	1.00	0	1	1	0	1	1	1	1	1	1	1
0	0	58	2	154	51.6	-.40	21.7	145	78	213	88	125.00	6.20	1	1.00	0	1	1	0	1	1	1	1	0	1	0
0	0	56	1	167	66.2	-10.30	23.7	121	86	274	61	213.00	7.40	1	1.00	0	1	1	0	1	1	1	1	0	0	0
1	1	69	1	160	56.7	-.80	22.1	108	67	150	47	103.00	5.60	1	1.00	1	0	1	0	1	1	0	1	1	1	1
1	1	65	1	168	71.8	1.00	25.3	119	63	177	69	108.00	5.50	1	1.00	0	0	1	0	1	0	0	0	1	1	1
1	1	76	1	168	75.5	-.10	26.7	159	88	146	46	100.00	5.90	1	1.00	1	0	0	0	1	1	1	0	0	1	1
1	1	69	2	160	62.6	-1.40	24.3	153	82	210	54	156.00	5.80	1	1.00	1	1	1	1	1	0	0	1	0	1	1
1	1	73	1	155	62.8	-.10	26.3	144	82	176	48	128.00	6.60	1	1.00	0	0	1	1	1	1	1	0	0	1	0
1	1	64	1	165	66.2	.50	24.4	125	74	193	52	141.00	5.40	1	1.00	1	1	1	1	1	1	0	1	0	1	1
1	1	63	1	157	60.4	1.20	24.5	157	78	210	76	134.00	5.90	0	1.00	0	0	1	1	1	0	0	1	0	1	0
1	1	68	2	144	45.5	-4.10	22.0	159	90	199	61	138.00	5.30	1	1.00	1	0	1	1	1	1	1	1	0	1	1
1	1	67	2	143	45.0	-.20	21.9	134	73	202	71	131.00	5.80	1	1.00	1	0	1	1	1	1	1	1	1	1	1
1	1	78	1	155	63.3	.20	26.3	145	76	168	37	131.00	5.70	0	1.00	1	1	1	1	1	1	1	0	0	0	1
1	1	75	2	143	65.5	.10	31.9	129	70	208	57	151.00	6.00	1	1.00	1	1	1	1	1	1	1	0	0	1	1
1	1	75	2	145	51.7	1.70	24.6	132	74	210	72	138.00	5.50	1	1.00	1	1	1	1	1	1	1	1	0	1	1
1	1	71	1	159	61.3	-1.60	24.3	91	57	175	65	110.00	5.40	1	1.00	0	0	1	0	1	0	1	1	1	1	1
1	1	70	1	161	54.3	2.00	21.0	119	74	238	53	185.00	5.60	1	1.00	0	0	1	1	1	1	1	1	1	0	1
1	1	51	2	160	49.6	1.30	19.3	110	70	228	98	130.00	5.20	1	1.00	1	0	1	0	0	0	1	1	1	0	1
1	0	77	2	157	56.1	1.20	22.8	133	75	188	63	125.00	5.80	1	1.00	1	0	1	1	1	0	1	1	1	0	1
1	1	77	2	135	54.4	2.10	29.8	153	86	178	38	140.00	6.40	1	1.00	1	0	1	1	1	1	1	0	0	0	1
1	1	75	1	164	66.1	-.90	24.6	128	80	179	66	113.00	6.30	1	1.00	0	0	1	0	1	1	1	1	0	1	1
0	0	65	1	169	63.4	.20	22.0	153	86	226	80	146.00	5.60	1	1.00	0	1	1	1	1	0	1	1	0	0	1
1	1	35	1	171	66.4	.10	22.6	91	54	204	47	157.00	5.50	1	1.00	1	0	1	0	1	1	1	1	1	1	1
1	1	74	2	140	47.2	-.50	24.1	127	78	191	33	158.00	5.70	1	1.00	1	0	1	1	1	1	1	1	0	0	1
1	1	62	2	149	68.0	.90	30.4	97	63	165	80	85.00	5.70	1	1.00	0	1	0	0	1	1	1	0	0	1	1

1	1	76	2	149	41.6	-1.70	18.7	140	73	219	58	161.00	5.70	1	1.00	1	0	1	0	1	0	1	1	0	1	1
1	1	64	2	157	52.8	-3.70	21.5	142	77	257	78	179.00	5.40	1	1.00	1	1	1	0	0	0	0	1	0	0	1
1	1	76	2	149	49.0	-.80	22.1	122	81	182	59	123.00	6.80	1	1.00	1	0	1	1	1	0	1	1	0	1	0
0	0	68	2	154	54.0	.30	22.6	122	69	291	78	213.00	5.80	1	1.00	1	1	1	1	1	1	0	1	1	0	1
1	1	66	1	164	61.0	.20	22.6	129	68	203	90	113.00	5.80	1	1.00	0	0	1	0	1	0	1	1	1	1	1
0	0	45	1	159	51.0	-.20	20.0	108	62	167	49	118.00	5.20	0	1.00	0	0	1	0	1	0	1	1	1	1	1
1	1	69	1	150	50.4	.30	22.2	144	80	195	88	107.00	5.70	1	1.00	0	1	1	1	1	1	1	1	0	1	0
1	1	75	1	159	56.4	-.60	22.2	103	56	230	67	163.00	5.70	0	1.00	0	0	1	1	1	0	1	1	0	0	1
0	0	56	1	185	77.0	5.30	22.4	109	72	173	84	89.00	5.80	0	1.00	0	1	1	0	1	1	0	1	1	1	1
1	1	80	1	164	61.1	1.90	22.7	103	65	164	55	109.00	5.00	1	1.00	0	0	1	0	1	1	1	1	0	1	1
0	1	75	2	140	36.4	-3.40	18.5	127	73	211	75	136.00	5.70	1	1.00	1	0	1	0	1	0	1	1	1	1	1
0	1	74	1	160	55.6	.50	21.7	117	59	263	62	201.00	5.60	1	1.00	0	0	1	0	1	1	1	1	1	0	1
1	1	64	2	153	58.4	3.10	24.9	140	79	221	57	164.00	6.20	1	1.00	1	0	1	1	1	1	1	1	0	0	0
0	0	68	1	163	60.7	-.70	22.9	127	86	205	72	133.00	5.30	1	1.00	1	0	1	1	1	1	1	1	1	0	1
1	1	37	2	159	51.0	-2.20	20.1	109	64	165	69	96.00	5.50	1	1.00	1	0	1	1	1	1	1	1	1	1	1
0	0	73	1	157	60.0	-.10	24.2	142	71	219	61	158.00	5.90	1	1.00	0	0	1	0	1	0	0	1	0	1	1
0	0	37	1	159	55.4	-.60	21.9	105	61	212	59	153.00	5.30	0	1.00	1	1	0	0	0	0	1	1	1	1	1
0	0	93	2	142	52.0	-.90	25.8	90	56	287	40	247.00	7.80	1	1.00	1	0	0	1	1	0	1	0	1	0	0
0	0	70	1	167	66.7	-.90	23.8	119	66	194	45	149.00	5.80	1	1.00	0	0	1	0	1	1	0	1	1	1	1
0	0	37	2	157	69.6	6.10	28.1	122	68	149	34	115.00	5.40	1	1.00	1	1	1	1	1	1	1	0	1	0	1
0	0	38	2	154	50.7	.60	21.4	104	63	127	52	75.00	5.50	1	1.00	0	0	1	1	1	1	1	1	1	1	1
1	1	70	2	150	43.6	-.30	19.3	108	62	221	56	165.00	5.60	1	1.00	1	1	1	0	1	1	1	1	1	0	1
0	0	66	2	152	51.3	.60	22.1	110	67	221	84	137.00	5.30	1	1.00	1	0	1	0	1	1	0	1	1	0	1
1	1	67	1	167	63.4	-1.90	22.7	122	73	189	74	115.00	5.80	0	1.00	1	1	1	0	1	1	1	1	1	1	1
1	1	74	2	149	55.3	-.50	24.8	142	80	177	59	118.00	5.60	1	1.00	1	1	1	1	1	1	1	1	0	1	1
0	0	51	1	178	79.3	.20	24.9	128	69	176	82	94.00	6.10	1	1.00	1	0	1	0	1	0	1	1	1	0	0
1	1	64	1	163	72.4	.20	27.2	108	64	254	60	194.00	6.50	0	1.00	1	1	1	1	1	1	1	0	1	0	0
1	1	63	2	157	64.1	-.50	26.0	135	78	211	71	140.00	5.80	1	1.00	1	0	1	1	1	0	1	0	1	1	1
1	1	57	2	157	61.9	.00	24.9	143	82	224	58	166.00	6.40	1	1.00	1	1	1	0	0	1	1	1	0	0	1
1	1	31	1	181	123.8	-.80	37.7	131	66	148	47	101.00	6.70	1	1.00	1	0	1	0	1	0	1	0	1	1	0
1	1	64	2	151	55.4	-4.40	24.4	119	70	207	91	116.00	5.40	0	2.00	0	1	1	0	1	0	0	1	0	1	1
1	1	74	1	156	64.2	-3.20	26.2	110	72	240	48	192.00	5.70	1	1.00	0	1	1	1	0	1	1	0	1	0	1
1	1	65	1	173	68.3	.80	22.8	114	70	193	69	124.00	5.60	1	1.00	0	0	1	0	1	1	1	1	0	1	1
1	1	80	1	157	60.4	-.10	24.4	122	62	186	65	121.00	5.90	1	1.00	0	0	1	0	1	0	0	1	0	1	1
1	1	80	2	149	58.8	.10	26.4	127	63	154	41	113.00	6.00	1	1.00	1	0	1	1	1	0	0	0	0	1	1
1	1	84	1	155	53.2	-.80	22.2	138	69	170	49	121.00	6.10	1	1.00	1	0	1	1	1	1	1	1	1	1	1
1	1	76	1	165	59.4	-2.50	21.9	127	72	235	82	153.00	5.40	1	1.00	0	0	1	1	1	1	0	1	1	0	1
1	1	69	2	148	36.1	.50	16.4	145	81	246	74	172.00	5.40	1	1.00	1	0	1	0	1	0	0	1	0	0	1
1	1	70	1	167	73.8	-1.50	26.3	133	85	187	42	145.00	5.50	1	1.00	0	0	1	1	0	1	1	0	1	1	1

0	0	49	2	165	56.8	-.70	20.8	138	79	160	73	87.00	4.90	1	1.00	1	1	1	1	1	1	1	1	1	1	1
1	1	74	1	160	85.3	-3.80	33.1	149	77	194	62	132.00	6.10	1	1.00	0	1	1	1	1	1	1	0	0	1	1
0	0	71	2	157	57.2	.80	23.2	116	73	197	78	119.00	5.50	1	1.00	1	0	1	1	1	1	1	1	1	0	1
1	1	69	1	156	50.4	3.60	20.7	137	83	152	67	85.00	5.70	1	1.00	0	0	1	0	1	1	0	1	0	1	1
1	1	69	2	146	39.3	.60	18.5	123	71	285	57	228.00	5.90	1	1.00	1	0	1	1	1	0	1	1	0	0	1
1	1	56	2	157	40.7	.20	16.5	114	73	206	101	105.00	5.10	1	1.00	1	0	1	0	1	1	0	1	1	1	1
1	1	76	1	161	60.6	-3.60	23.2	137	78	143	58	85.00	8.70	1	1.00	1	0	1	1	1	1	1	1	1	1	0
1	1	70	1	162	58.3	2.00	22.3	133	87	218	81	137.00	5.70	1	1.00	0	1	1	0	1	1	1	1	0	1	1
0	0	36	2	154	62.3	.40	26.3	125	73	230	89	141.00	5.20	1	1.00	1	0	1	0	1	0	0	0	1	0	1
0	0	55	2	163	63.5	1.10	24.0	116	69	205	71	134.00	5.30	1	1.00	1	1	1	1	0	1	1	1	1	1	1
1	1	60	2	150	43.5	-.20	19.3	134	67	209	76	133.00	5.70	1	1.00	0	0	1	1	1	1	0	1	1	0	1
1	1	74	2	150	60.0	.20	26.6	143	82	205	45	160.00	5.60	1	1.00	1	1	1	0	0	1	1	0	0	1	1
1	1	72	1	158	57.5	3.50	23.0	106	63	232	50	182.00	5.70	1	1.00	1	0	1	1	1	1	1	1	0	0	1
1	1	71	2	143	56.0	1.00	27.2	142	84	239	58	181.00	5.10	1	1.00	1	0	1	1	1	1	1	0	0	0	1
1	1	43	2	156	64.1	-4.70	26.2	128	77	186	50	136.00	5.20	1	1.00	0	1	1	0	1	1	1	0	1	1	1
0	0	41	1	169	79.2	2.10	27.8	123	77	195	59	136.00	5.70	0	1.00	0	1	0	0	1	0	0	0	1	1	1
1	1	55	2	154	56.3	-1.50	23.6	120	83	198	61	137.00	5.60	1	1.00	1	0	1	0	1	0	1	1	1	1	1
1	1	54	2	155	52.0	1.80	21.7	104	71	215	62	153.00	5.40	1	1.00	1	0	1	0	1	0	0	1	1	1	1
1	1	71	1	153	58.3	-1.90	24.9	133	71	238	45	193.00	5.90	1	1.00	1	1	1	1	1	1	1	1	0	0	1
1	1	80	1	164	70.7	-1.70	26.4	126	73	185	52	133.00	5.60	1	1.00	0	0	1	1	1	1	1	0	0	0	1
0	0	60	2	157	50.5	.20	20.4	94	55	228	94	134.00	5.80	1	1.00	1	0	1	0	0	0	1	1	1	0	1
1	1	59	2	157	49.3	1.50	19.9	146	80	228	62	166.00	5.10	1	1.00	1	0	1	1	1	1	1	1	0	0	1
0	0	46	2	172	87.5	7.90	29.7	113	71	217	66	151.00	5.50	1	1.00	1	0	1	0	0	1	1	0	1	1	1
1	1	70	2	160	51.4	2.30	19.9	142	79	162	64	98.00	5.90	1	1.00	1	1	1	1	1	1	1	1	0	1	1
1	1	86	1	161	49.0	-.90	18.9	153	79	149	70	79.00	5.50	1	1.00	1	0	1	1	1	1	1	1	0	1	1
1	1	67	2	142	46.3	2.50	23.0	114	66	190	61	129.00	5.70	1	1.00	1	1	1	1	1	0	0	1	1	1	1
1	1	57	1	168	72.8	-1.40	25.7	151	95	194	60	134.00	4.80	0	1.00	0	0	1	1	1	1	1	0	0	1	1
0	0	75	2	150	51.3	1.10	22.8	143	72	200	58	142.00	5.20	1	1.00	1	0	1	1	0	1	0	1	0	1	1
0	0	78	2	140	45.6	-1.20	23.2	148	83	201	78	123.00	5.20	1	1.00	1	0	1	0	1	0	1	1	0	1	1
0	0	71	2	139	41.2	-1.30	21.3	150	74	226	70	156.00	6.00	1	1.00	1	0	1	0	1	1	1	1	0	0	1
0	0	48	2	171	59.4	-3.40	20.3	131	83	191	53	138.00	5.30	1	1.00	1	0	1	0	1	0	1	1	1	1	1
0	0	74	2	154	45.7	.30	19.2	169	91	205	82	123.00	5.20	1	1.00	1	0	1	1	0	1	1	1	0	1	1
0	1	64	2	156	50.3	-1.00	20.6	124	76	205	96	109.00	5.60	1	1.00	1	1	1	1	1	0	1	1	1	0	1
1	1	72	2	147	66.0	-3.40	30.3	133	76	206	53	153.00	6.50	1	1.00	1	0	1	0	1	1	1	0	0	0	0
1	1	64	2	152	59.6	2.40	25.9	140	77	185	50	135.00	5.60	1	1.00	1	0	1	1	1	0	1	0	0	1	1
1	1	72	2	150	50.3	2.10	22.5	115	69	221	65	156.00	6.40	1	1.00	1	0	1	0	1	0	0	1	1	0	1
1	1	37	1	166	69.9	-2.20	25.3	116	68	174	59	115.00	5.40	0	1.00	1	1	1	0	1	1	1	0	1	1	1
0	0	75	2	156	70.9	4.30	29.2	155	81	176	61	115.00	6.20	1	1.00	1	0	1	1	0	1	1	0	0	1	0
1	1	76	2	152	51.4	-.90	22.3	148	73	162	65	97.00	5.50	1	1.00	1	0	1	1	1	1	1	1	0	1	1

1	1	55	1	162	60.7	-4.60	23.1	118	82	161	42	119.00	5.50	1	1.00	0	0	1	0	1	0	0	1	1	1	1
1	1	67	2	152	53.1	1.10	23.1	130	69	174	57	117.00	6.20	1	1.00	1	0	1	1	1	0	0	1	0	0	1
1	1	78	1	158	72.5	-4.10	28.8	135	88	202	69	133.00	5.90	0	1.00	1	0	1	1	1	1	1	0	0	1	1
0	0	48	2	150	49.5	-1.70	21.9	105	67	285	78	207.00	5.70	0	2.00	1	1	1	0	1	1	1	1	1	0	1
1	1	40	2	157	53.6	3.30	21.7	107	71	174	72	102.00	5.40	1	1.00	0	1	1	0	0	1	0	1	1	1	1
0	0	78	1	157	54.5	-2.00	22.2	126	68	121	60	61.00	5.20	1	1.00	1	0	1	0	1	0	0	1	1	1	1
1	1	76	2	145	46.2	-.30	22.0	158	73	174	50	124.00	5.80	1	1.00	1	0	1	0	1	1	1	1	0	1	1
1	1	62	2	146	56.1	.60	26.4	142	91	205	78	127.00	5.00	1	1.00	1	0	1	0	0	0	0	0	0	1	1
1	1	68	2	154	62.9	1.00	26.3	90	49	164	62	102.00	5.60	1	1.00	1	0	1	0	1	0	0	0	1	1	1
1	1	82	1	160	58.0	.10	22.5	131	70	152	69	83.00	5.10	1	1.00	1	0	1	1	1	1	1	1	0	1	1
1	1	70	2	150	45.1	-2.90	19.9	148	66	237	88	149.00	5.70	1	1.00	1	1	1	0	1	0	1	1	0	0	1
1	1	82	1	161	67.2	.50	25.8	133	66	169	46	123.00	5.60	1	1.00	1	0	1	0	1	1	1	0	0	1	1
1	1	74	2	152	45.1	-2.30	19.4	121	68	184	53	131.00	5.60	1	1.00	1	0	1	0	1	1	1	1	1	1	1
1	1	65	2	148	51.4	-6.20	23.6	202	115	279	84	195.00	5.60	1	1.00	1	1	1	0	1	1	1	1	0	0	1
1	1	59	1	159	58.3	-.30	23.1	108	64	162	41	121.00	9.90	0	1.00	1	0	1	1	1	1	1	1	1	1	0
1	1	34	2	161	58.3	2.00	22.4	90	49	191	55	136.00	5.40	0	1.00	1	1	1	0	1	1	1	1	1	1	1
1	1	65	2	148	67.2	-2.60	30.6	152	82	178	66	112.00	5.40	1	1.00	1	0	1	0	1	1	1	0	0	0	1
1	1	61	2	144	55.0	-1.50	26.6	142	82	167	56	111.00	5.90	1	1.00	1	0	1	0	1	1	0	0	0	0	1
1	1	65	2	148	46.5	-3.50	21.3	174	89	195	84	111.00	6.00	1	1.00	1	0	1	0	1	0	1	1	0	1	1
0	1	81	2	155	67.7	.50	28.1	146	68	158	51	107.00	6.00	1	1.00	1	0	1	1	1	0	1	0	0	0	1
1	1	54	1	159	59.6	.70	23.5	180	116	257	76	181.00	5.50	1	1.00	0	1	1	0	1	1	1	1	0	0	1
1	1	41	2	162	63.4	.50	24.3	129	74	181	72	109.00	5.00	1	1.00	0	1	1	0	1	0	0	1	1	1	1
1	1	71	2	152	52.3	-1.50	22.6	175	94	283	77	206.00	5.30	1	1.00	0	0	1	1	1	1	0	1	0	0	1
0	0	51	1	163	65.8	1.70	24.8	143	98	182	75	107.00	4.60	1	1.00	0	1	1	0	1	1	1	1	0	1	1
1	1	63	2	149	50.2	-1.60	22.6	135	76	147	84	63.00	5.60	1	1.00	1	0	1	0	1	0	1	1	1	1	1
0	1	67	2	145	35.9	1.60	16.9	107	66	214	99	115.00	5.80	1	1.00	1	0	1	1	1	1	1	1	1	1	1
1	1	63	2	137	42.9	-1.80	22.9	98	53	150	33	117.00	5.50	1	1.00	1	1	1	0	1	1	1	1	1	0	1
0	0	55	2	158	74.4	.10	29.8	147	94	238	87	151.00	5.80	1	1.00	1	1	1	0	1	0	0	0	0	0	1
1	1	59	2	151	53.1	-1.50	23.3	149	77	239	63	176.00	6.10	1	1.00	1	0	1	1	0	1	1	1	0	0	1
0	0	34	1	172	66.8	-3.50	22.5	124	80	193	54	139.00	5.00	1	1.00	0	0	1	1	1	0	0	1	1	1	1
0	0	64	1	162	62.9	1.00	23.9	142	81	197	88	109.00	5.10	0	1.00	0	0	1	0	0	0	1	1	0	1	1
1	1	71	2	152	50.5	-2.50	21.7	192	114	215	58	157.00	5.80	1	1.00	1	0	1	1	1	0	0	1	0	1	1
1	1	45	1	173	72.4	-7.60	24.3	151	101	216	48	168.00	5.20	1	1.00	0	0	1	1	1	1	1	1	0	1	1
1	1	47	2	159	65.9	-1.10	25.9	122	69	187	83	104.00	5.20	1	1.00	1	0	1	0	1	1	1	0	1	1	1
1	1	39	1	167	63.6	-6.20	22.8	131	78	275	54	221.00	5.60	0	1.00	0	0	0	0	0	1	1	1	1	0	1
1	1	74	2	152	57.6	-1.00	24.8	142	75	165	84	81.00	5.50	1	1.00	1	0	1	1	1	1	1	1	0	0	1
1	1	43	2	159	51.2	-2.70	20.3	116	66	205	103	102.00	5.10	1	1.00	1	0	1	0	0	1	0	1	1	1	1
1	1	73	2	141	44.4	-.30	22.4	88	48	127	66	61.00	6.10	1	1.00	1	0	1	1	1	1	0	1	0	0	0
0	0	62	1	170	68.8	1.40	23.7	141	82	231	88	143.00	5.40	0	1.00	0	0	1	0	0	0	0	1	0	0	1

0	0	58	2	152	57.6	-2.50	24.9	129	81	187	52	135.00	5.60	1	1.00	1	0	1	1	0	1	1	1	0	1	1
1	1	18	1	168	86.2	1.20	30.6	122	69	199	49	150.00	5.40	1	1.00	1	0	1	1	1	1	1	0	1	1	1
1	1	77	1	158	57.9	-1.80	23.0	122	68	159	52	107.00	5.70	1	1.00	1	0	1	0	1	1	1	1	0	1	1
1	1	77	1	160	63.2	-1.60	24.8	143	82	240	64	176.00	6.30	0	1.00	0	0	1	0	1	1	1	1	0	0	1
1	1	77	2	153	50.5	-2.50	21.4	124	83	222	82	140.00	5.70	1	1.00	1	0	1	0	1	1	1	1	0	0	1
0	0	78	1	164	62.3	-.70	23.1	125	76	197	71	126.00	5.30	1	1.00	1	0	1	0	1	1	0	1	1	1	1
1	1	68	1	177	66.3	-1.40	21.1	100	63	246	53	193.00	7.90	1	1.00	1	0	1	0	1	1	1	1	1	0	0
0	0	39	2	149	75.1	3.50	34.0	128	64	210	63	147.00	5.20	0	1.00	1	0	1	1	1	1	0	0	1	1	1
0	0	40	1	172	81.4	.10	27.4	136	93	214	53	161.00	5.50	0	1.00	0	0	1	0	1	1	1	0	0	1	1
0	0	76	1	155	66.6	-1.00	27.6	126	68	186	60	126.00	5.70	0	1.00	1	0	1	1	1	0	1	0	0	1	1
1	1	68	2	143	52.1	2.20	25.3	126	65	206	44	162.00	5.60	1	1.00	1	1	1	1	1	1	1	0	0	1	1
1	1	27	2	162	85.1	2.50	32.3	119	68	171	63	108.00	5.60	1	1.00	1	1	1	1	1	0	0	0	1	1	1
1	1	65	1	163	72.7	-3.90	27.2	135	78	213	46	167.00	5.70	1	1.00	0	0	1	1	1	0	0	0	0	0	1
1	1	76	1	165	68.4	-2.00	25.0	133	74	144	38	106.00	6.00	1	1.00	1	1	1	0	1	1	1	0	0	0	1
1	1	61	2	143	52.8	-6.10	25.7	98	63	197	76	121.00	5.10	1	1.00	0	0	1	0	1	0	0	0	1	1	1
1	1	34	2	153	47.2	-.20	20.1	108	63	184	75	109.00	5.30	1	1.00	1	1	1	1	1	1	1	1	1	1	1
0	0	65	1	164	61.7	-1.20	22.9	137	79	153	41	112.00	6.80	0	1.00	0	0	1	0	1	1	1	1	0	1	0
1	1	29	2	158	67.1	-1.50	27.0	138	81	217	77	140.00	5.50	1	1.00	1	1	1	0	1	1	1	0	1	1	1
0	0	70	2	147	50.2	2.70	23.3	117	73	235	50	185.00	5.30	1	1.00	1	0	1	0	0	0	0	1	1	0	1
1	1	68	2	148	56.6	.20	25.9	136	90	229	89	140.00	5.70	1	1.00	1	1	1	0	0	0	1	0	0	0	1
0	0	60	1	165	57.1	-.10	20.9	138	80	230	64	166.00	5.40	0	2.00	0	0	1	1	0	0	1	1	1	0	1
1	1	58	1	174	80.7	.50	26.6	165	99	252	84	168.00	5.00	0	1.00	0	0	1	1	0	1	0	0	0	0	1
1	1	67	1	160	50.1	.80	19.6	127	90	198	70	128.00	5.80	0	1.00	1	1	1	0	1	1	1	1	0	1	1
1	1	65	2	154	50.8	.90	21.3	122	68	205	68	137.00	5.60	1	1.00	1	0	1	1	1	0	1	1	1	1	1
1	1	30	1	162	71.5	2.20	27.2	117	67	174	48	126.00	5.10	1	1.00	1	0	1	0	1	1	1	0	1	1	1
0	0	79	2	155	62.3	2.00	26.0	137	72	177	61	116.00	6.30	1	1.00	1	1	1	1	1	0	1	0	1	1	0
0	0	69	2	150	54.3	.80	24.0	138	80	145	61	84.00	5.20	1	1.00	1	0	1	1	1	1	1	1	1	1	1
0	0	47	2	156	49.9	-2.60	20.6	108	69	243	120	123.00	5.60	1	1.00	1	0	1	1	1	0	1	1	1	0	1
1	1	43	2	159	47.0	-1.80	18.7	111	65	161	67	94.00	6.80	1	1.00	0	0	1	0	1	0	1	1	1	1	0
1	1	73	1	158	63.9	1.00	25.6	152	76	235	48	187.00	6.80	1	1.00	1	0	1	1	1	1	1	0	0	0	0
1	1	75	2	152	58.8	-2.40	25.3	112	69	165	53	112.00	5.40	1	1.00	0	1	1	1	1	0	1	0	1	1	1
0	0	72	1	157	55.4	1.80	22.3	126	73	202	78	124.00	5.20	1	1.00	0	0	1	1	1	0	0	1	1	1	1
0	0	68	1	155	65.3	-3.20	27.0	123	76	186	47	139.00	5.90	1	1.00	1	0	1	1	1	1	1	0	0	1	1
0	0	76	1	165	71.2	-.80	26.2	143	95	177	32	145.00	5.60	0	1.00	1	0	1	1	1	1	1	0	0	0	1
1	0	29	1	165	61.7	1.00	22.6	103	64	180	65	115.00	5.30	1	1.00	1	0	1	0	1	1	1	1	1	1	1
1	1	74	2	154	47.8	-2.30	20.1	132	67	190	61	129.00	6.40	1	1.00	1	1	1	1	1	1	1	1	0	1	0
1	1	56	2	156	98.5	-3.50	40.5	155	89	249	47	202.00	5.30	1	1.00	1	0	1	0	0	0	0	0	0	0	1
0	0	74	1	166	59.8	.80	21.7	106	68	187	65	122.00	5.40	1	1.00	1	1	1	0	1	1	1	1	1	1	1
1	1	69	2	159	57.6	-.30	22.6	171	108	188	58	130.00	6.00	1	1.00	1	0	0	1	1	1	1	1	0	1	1

0	0	59	2	155	67.7	-1.50	28.3	180	101	240	91	149.00	5.50	1	1.00	1	0	1	0	0	0	1	0	0	0	1
1	1	72	1	152	71.6	-2.80	31.0	143	88	153	52	101.00	5.00	1	1.00	0	1	1	1	1	1	1	0	0	1	1
1	1	35	2	161	57.0	.50	22.0	110	64	186	90	96.00	5.20	1	1.00	1	0	1	0	1	0	1	1	1	1	1
1	1	75	1	162	69.5	-4.20	26.6	121	71	204	54	150.00	5.70	1	1.00	1	0	1	1	1	1	0	0	0	1	1
1	1	62	1	157	55.7	-1.80	22.5	105	61	197	42	155.00	5.50	0	1.00	1	1	1	0	1	1	1	1	1	1	1
1	1	49	1	167	90.5	-.40	32.3	127	73	264	55	209.00	5.20	0	1.00	1	0	1	0	1	0	1	0	1	0	1
1	0	71	2	153	41.5	3.40	17.8	141	79	223	91	132.00	5.60	1	1.00	1	1	1	0	0	0	1	1	0	0	1
1	1	72	2	153	55.0	-2.00	23.5	140	80	229	73	156.00	5.90	1	1.00	1	1	1	1	1	1	1	1	0	0	1
1	1	71	2	158	65.6	-.70	26.3	108	62	143	51	92.00	6.00	1	1.00	1	0	1	1	1	1	1	0	1	0	1
0	0	68	2	142	49.5	.60	24.5	110	56	195	60	135.00	5.40	1	1.00	1	1	1	0	1	1	0	1	1	0	1
1	1	39	2	156	67.0	.00	27.4	137	82	240	73	167.00	7.10	1	1.00	1	0	1	1	1	0	0	0	0	0	0
0	0	53	2	149	47.9	-1.10	21.4	147	84	213	59	154.00	5.60	1	1.00	1	1	1	1	1	0	0	1	0	1	1
1	1	50	2	158	61.8	-1.80	24.7	156	101	209	62	147.00	5.10	1	1.00	1	0	1	0	0	0	1	1	0	1	1
0	0	48	2	155	51.1	.00	21.2	97	54	252	72	180.00	5.00	1	1.00	1	0	1	0	0	0	1	1	1	0	1
1	1	40	1	164	62.4	.00	23.3	123	79	179	44	135.00	5.40	1	1.00	0	0	1	1	1	1	1	1	1	1	1
1	1	44	2	162	70.7	.10	27.0	155	102	178	65	113.00	5.30	1	1.00	1	1	0	1	1	1	1	0	0	1	1
0	0	66	1	163	69.4	1.10	26.0	140	85	212	51	161.00	6.10	0	1.00	0	0	1	0	1	1	1	0	0	1	1
0	0	42	2	152	72.0	6.00	31.0	105	59	214	58	156.00	5.50	1	1.00	1	0	0	1	0	0	0	0	1	1	1
0	0	48	1	176	75.9	-.30	24.5	118	81	223	65	158.00	5.20	1	1.00	0	1	1	1	1	0	1	1	0	0	1
0	0	70	1	160	68.7	-1.40	26.7	108	71	184	93	91.00	5.50	1	1.00	0	0	1	0	1	0	1	0	0	1	1
1	1	57	2	158	58.3	1.40	23.4	112	59	267	92	175.00	5.60	1	1.00	1	0	1	1	1	1	0	1	1	0	1
0	0	41	2	157	47.2	.30	19.1	110	60	211	92	119.00	5.10	1	1.00	1	0	1	1	1	0	1	1	1	1	1
0	0	29	2	157	43.8	.00	17.8	122	72	198	100	98.00	5.40	1	1.00	1	0	0	0	1	1	1	1	1	1	1
1	1	61	2	156	57.0	-1.50	23.4	134	73	258	98	160.00	5.50	1	1.00	1	1	1	1	1	1	1	1	1	0	1
1	1	60	2	158	63.1	-2.00	25.3	142	81	269	64	205.00	5.90	1	1.00	1	0	1	0	0	1	0	0	0	0	1
1	1	71	2	146	49.7	-.50	23.1	139	65	214	61	153.00	5.90	1	1.00	1	0	1	1	1	1	1	1	1	0	1
0	0	70	2	150	54.8	.60	24.2	110	65	297	117	180.00	5.90	1	1.00	1	1	1	0	1	1	0	1	1	0	1
1	1	69	2	149	42.9	-2.20	19.4	93	52	164	70	94.00	5.40	1	1.00	1	0	1	0	1	0	0	1	1	1	1
1	1	54	1	166	65.9	-2.90	23.8	146	90	218	56	162.00	5.70	1	1.00	0	1	1	0	1	1	1	1	0	1	1
1	1	58	2	155	49.5	-2.20	20.5	133	87	235	103	132.00	5.80	1	1.00	1	0	1	1	1	0	1	1	1	0	1
1	1	47	1	169	74.5	1.50	26.0	122	80	194	55	139.00	5.80	0	1.00	0	0	1	0	1	1	1	0	1	1	1
1	1	58	1	163	70.5	.10	26.5	108	72	292	36	256.00	5.50	1	1.00	0	0	1	1	1	0	0	0	1	0	1
0	0	61	2	155	51.7	.20	21.5	122	72	219	65	154.00	5.60	1	1.00	1	1	1	0	1	0	1	1	1	0	1
0	0	70	2	152	51.0	.30	22.0	161	91	224	91	133.00	5.70	1	1.00	1	1	1	0	1	0	0	1	0	0	1
0	0	61	2	148	46.4	.60	21.2	132	71	196	77	119.00	5.40	1	1.00	1	0	1	0	0	0	1	1	0	0	1
0	0	49	1	157	46.7	1.00	18.9	152	101	157	91	66.00	5.40	1	1.00	0	0	1	0	1	1	1	1	0	1	1
0	0	39	2	157	64.0	.70	25.9	149	86	214	77	137.00	5.60	1	1.00	1	0	1	0	1	0	0	0	0	1	1
0	0	76	1	158	65.5	1.70	26.1	130	74	226	73	153.00	5.30	1	1.00	1	0	1	0	0	0	0	0	1	0	1
0	0	73	1	160	63.0	-.30	24.6	141	69	203	34	169.00	6.00	1	1.00	1	0	1	0	1	1	1	1	0	0	1

1	1	63	2	147	42.3	1.40	19.6	150	84	232	61	171.00	5.40	1	1.00	1	0	1	0	1	0	0	1	0	0	1
1	1	70	2	148	54.1	-1.00	24.6	133	65	239	87	152.00	5.90	1	1.00	1	1	1	0	1	0	1	1	0	0	1
1	1	72	2	147	44.2	-1.40	20.5	106	65	321	36	285.00	5.80	1	1.00	1	0	1	1	1	0	0	1	1	0	1
1	1	79	1	158	65.0	-.30	25.9	117	60	198	110	88.00	5.80	1	1.00	0	0	1	1	1	1	1	0	0	1	1
1	1	66	2	143	45.9	-1.60	22.3	124	75	244	103	141.00	6.10	1	1.00	1	1	1	0	1	1	0	1	1	0	1
0	0	67	1	162	59.2	-3.30	22.5	105	72	216	71	145.00	5.20	1	1.00	0	0	1	0	0	0	0	1	1	1	1
1	1	70	1	166	75.5	.40	27.2	146	84	221	46	175.00	6.60	1	1.00	1	0	1	0	1	1	1	0	0	0	0
1	1	71	2	150	52.2	2.50	23.2	137	89	269	55	214.00	5.80	1	1.00	1	1	1	0	1	0	1	1	1	0	1
1	1	71	2	151	58.5	-.20	25.5	129	76	226	53	173.00	5.70	1	1.00	0	0	1	1	1	0	1	0	1	0	1
0	0	68	1	162	68.9	-1.80	26.1	149	88	191	61	130.00	5.90	1	1.00	0	0	1	1	1	0	1	0	0	1	1
0	0	71	1	160	63.2	1.90	24.7	125	62	271	62	209.00	7.30	1	1.00	1	0	1	0	1	0	0	1	0	0	0
1	1	66	2	153	64.7	5.90	27.8	116	70	255	60	195.00	5.50	1	1.00	1	0	1	1	1	1	0	0	1	0	1
0	0	67	1	175	69.8	-2.20	22.8	123	75	192	59	133.00	5.80	1	1.00	0	1	1	0	1	0	0	1	0	1	1
1	1	58	2	157	54.1	-2.70	22.0	125	79	245	63	182.00	5.50	1	1.00	1	0	1	0	0	0	1	1	1	0	1
1	1	78	1	161	49.9	-.60	19.3	143	92	206	92	114.00	5.40	1	1.00	0	0	1	1	1	0	1	1	0	1	1
1	1	74	2	152	43.0	.20	18.7	144	77	209	52	157.00	5.50	1	1.00	1	0	1	1	1	1	1	1	0	0	1
1	1	70	1	169	68.7	-.70	24.0	112	73	202	43	159.00	6.00	0	1.00	0	1	1	1	1	1	1	1	1	1	1
0	0	71	1	171	72.0	1.50	24.7	100	59	183	47	136.00	5.90	0	1.00	1	1	1	0	1	1	1	1	0	1	1
1	1	51	2	159	48.3	-2.10	19.1	147	87	246	82	164.00	5.20	1	1.00	1	0	1	0	1	0	1	1	0	0	1
1	1	75	1	149	51.7	4.90	23.3	124	66	255	72	183.00	7.40	1	1.00	1	1	1	1	1	1	1	1	0	0	0
0	0	76	2	142	48.8	3.10	24.2	154	78	184	93	91.00	5.70	1	1.00	1	0	1	0	0	1	1	1	0	1	1
1	1	69	2	149	49.4	.70	22.1	122	67	207	74	133.00	5.60	1	1.00	1	0	1	1	1	1	1	1	1	1	1
1	1	81	1	160	54.1	.70	21.2	124	77	202	67	135.00	5.70	1	1.00	1	0	1	1	1	1	1	1	0	0	1
0	0	88	2	141	57.4	-1.90	28.8	138	71	232	62	170.00	5.60	1	1.00	1	0	1	1	1	1	1	0	0	0	1
1	1	57	2	153	50.2	-.40	21.4	139	80	210	95	115.00	5.60	1	1.00	1	0	1	0	1	0	1	1	1	1	1
0	0	66	2	148	61.0	2.40	27.7	141	83	277	53	224.00	5.70	1	1.00	1	0	1	0	1	1	1	0	0	0	1
1	1	65	2	144	56.4	-.30	27.1	124	84	162	67	95.00	6.00	1	1.00	1	0	1	1	1	1	1	0	0	0	1
1	1	76	2	162	61.1	-.90	23.3	121	68	244	74	170.00	5.60	1	1.00	1	1	1	1	1	0	1	1	1	0	1
1	1	66	2	147	48.2	1.70	22.4	153	82	207	54	153.00	5.30	1	1.00	1	0	1	0	1	0	1	1	0	1	1
0	0	71	1	166	68.9	1.60	24.8	127	80	206	76	130.00	5.70	1	1.00	0	0	1	0	1	1	1	1	0	1	1
1	1	79	1	158	67.1	-3.10	26.9	150	93	273	57	216.00	5.60	1	1.00	1	1	1	0	1	0	0	0	0	0	1
0	1	77	1	166	58.2	1.70	21.0	122	75	218	65	153.00	5.60	1	1.00	1	1	1	0	1	1	1	1	1	1	1
0	1	64	2	154	43.3	-.60	18.3	126	80	224	59	165.00	5.80	0	1.00	1	0	1	0	1	0	0	1	1	0	1
1	1	72	2	151	52.1	.70	22.8	118	79	191	49	142.00	6.30	1	1.00	1	0	1	1	1	1	0	1	1	0	1
0	0	70	2	151	53.6	2.40	23.6	143	75	235	59	176.00	8.20	1	1.00	1	0	1	0	1	0	0	1	0	0	0
0	0	77	2	145	43.8	1.70	20.7	123	61	237	47	190.00	5.60	1	1.00	1	0	1	1	1	0	0	1	1	0	1
0	0	71	1	156	71.8	-.20	29.5	133	83	194	73	121.00	6.40	1	1.00	0	1	1	1	1	1	1	0	0	1	1
1	1	55	1	171	67.9	1.20	23.3	100	69	180	62	118.00	5.70	0	1.00	0	1	1	1	1	1	1	1	1	1	1
1	1	73	2	151	50.7	.50	22.3	128	80	181	70	111.00	5.40	1	1.00	0	0	1	1	1	0	1	1	1	1	1

1	1	60	2	154	59.9	-1.90	25.4	113	67	198	65	133.00	5.80	1	1.00	1	1	1	1	1	1	0	0	1	0	1
1	1	67	1	160	60.3	-.40	23.6	109	59	243	109	134.00	5.70	1	1.00	0	1	1	1	1	1	1	1	1	0	1
1	1	73	1	173	72.6	-.60	24.3	106	70	185	36	149.00	5.60	1	1.00	1	1	1	1	1	1	1	1	1	0	1
0	0	70	2	146	51.3	.80	23.9	125	76	242	68	174.00	6.00	1	1.00	1	0	1	1	1	1	1	1	1	0	1
1	1	67	2	148	49.8	4.00	22.7	135	88	173	42	131.00	5.40	1	1.00	1	0	1	1	1	1	1	1	0	0	1
1	1	67	1	165	62.8	.00	22.9	138	94	215	80	135.00	6.50	1	1.00	0	1	1	1	1	1	1	1	0	1	0
1	1	55	2	160	60.0	.40	23.5	112	76	229	72	157.00	5.50	1	1.00	1	0	1	1	0	1	1	1	1	0	1
1	1	64	1	161	56.0	-2.30	21.6	144	79	234	55	179.00	5.60	1	1.00	1	1	1	0	1	0	1	1	0	0	1
1	1	51	2	155	63.8	1.20	26.5	130	83	289	44	245.00	5.40	1	1.00	1	1	1	1	0	0	1	0	1	0	1
1	1	52	1	162	62.5	1.20	23.8	121	79	179	52	127.00	5.30	1	1.00	1	1	1	0	1	1	1	1	1	1	1
1	1	66	1	164	67.9	-2.30	25.3	147	85	193	73	120.00	5.70	1	1.00	0	1	1	0	1	0	1	0	0	1	1
1	1	73	2	144	44.7	2.30	21.4	100	52	219	66	153.00	5.30	1	1.00	1	1	1	0	1	0	0	1	1	0	1
1	1	71	1	162	48.4	.40	18.3	117	72	187	66	121.00	5.90	1	1.00	1	0	1	0	1	1	1	1	1	1	1
1	1	85	1	161	58.9	-.30	22.6	129	55	211	54	157.00	5.30	1	1.00	0	0	1	0	1	0	0	1	0	1	1
1	1	77	2	146	60.9	1.70	28.5	155	83	193	59	134.00	5.60	1	1.00	1	0	1	1	1	1	1	0	0	1	1
1	1	75	1	160	60.2	-2.40	23.5	140	79	146	36	110.00	5.60	1	1.00	0	1	1	1	1	1	1	1	0	0	1
1	1	66	2	146	35.7	.30	16.7	106	62	235	101	134.00	5.80	1	1.00	1	0	1	0	1	0	0	1	1	0	0
1	1	63	2	154	62.6	.30	26.2	152	87	226	75	151.00	5.70	1	1.00	1	0	1	0	1	1	0	0	0	0	1
1	1	61	2	148	55.4	-6.20	25.1	131	74	174	55	119.00	6.80	1	1.00	1	0	1	1	1	1	1	0	0	1	0
1	1	85	1	163	58.9	-.80	22.1	135	77	213	48	165.00	4.80	1	1.00	1	0	1	1	1	1	1	1	0	1	1
1	1	52	2	169	57.7	.10	20.2	94	62	177	79	98.00	5.60	1	1.00	1	1	1	0	0	1	1	1	1	1	1
1	1	80	1	160	58.2	-2.00	22.8	156	70	126	52	74.00	5.20	1	1.00	0	0	1	1	1	1	1	1	0	1	1
0	0	38	2	152	45.5	.20	19.6	98	58	180	73	107.00	5.10	1	1.00	1	0	1	0	1	1	1	1	1	1	1
0	1	66	2	157	52.9	1.50	21.5	115	67	266	61	205.00	5.20	1	1.00	1	1	1	0	1	0	0	1	1	0	1
1	1	77	2	145	56.3	-2.70	26.7	174	88	201	40	161.00	8.40	1	1.00	1	0	1	1	1	1	1	0	0	1	0
1	1	60	2	159	55.6	.00	22.1	101	63	235	61	174.00	5.50	1	1.00	1	1	1	1	1	0	0	1	1	0	1
1	1	74	2	137	42.1	-.20	22.5	127	69	198	52	146.00	5.20	1	1.00	1	0	1	1	1	1	1	1	0	1	1
1	1	64	2	154	46.8	.50	19.7	121	76	227	52	175.00	5.80	1	1.00	1	0	1	1	1	1	1	1	1	0	1
0	0	69	2	152	59.5	1.50	25.9	163	88	215	51	164.00	5.90	1	1.00	1	1	1	0	1	0	0	0	0	1	1
0	0	78	1	164	78.4	-.20	29.2	157	86	219	44	175.00	6.20	1	1.00	0	1	1	0	1	1	1	0	0	1	1
1	1	78	1	160	60.6	.50	23.7	123	71	218	41	177.00	5.60	0	1.00	1	0	1	1	1	0	1	1	1	1	1
1	1	80	2	144	48.8	-.10	23.4	120	59	182	69	113.00	5.70	1	1.00	1	0	1	0	1	0	1	1	0	0	1
1	1	76	1	165	71.3	2.00	26.1	158	85	184	46	138.00	5.50	1	1.00	0	0	1	1	1	1	1	0	0	1	1
1	1	74	1	168	69.6	-.10	24.5	125	74	303	57	246.00	5.70	1	1.00	0	1	1	1	1	1	0	1	1	0	1
0	0	69	2	146	34.9	-.30	16.4	115	69	215	88	127.00	5.50	1	1.00	1	0	1	1	1	1	0	1	1	1	1
1	1	90	1	164	69.2	-1.40	25.8	122	54	186	42	144.00	6.70	0	1.00	0	0	1	1	1	1	1	0	0	1	0
1	1	70	1	160	61.0	-1.60	23.9	91	61	130	46	84.00	5.90	1	1.00	0	1	1	1	1	1	1	1	1	1	1
1	1	34	1	177	90.3	3.30	28.9	121	69	222	31	191.00	5.10	1	1.00	1	0	0	0	1	1	1	0	1	0	1
0	0	81	2	145	50.0	2.30	23.8	125	73	218	67	151.00	5.60	1	1.00	1	0	1	0	0	0	1	1	1	1	1

1	1	73	1	167	58.2	3.10	20.7	123	77	194	55	139.00	5.00	1	1.00	0	0	1	0	1	1	1	1	1	1	1
0	0	78	1	156	56.5	1.80	23.3	122	70	194	35	159.00	6.10	1	1.00	1	0	1	0	1	1	1	1	0	0	1
1	1	57	1	168	63.1	1.70	22.4	115	73	176	76	100.00	5.80	1	1.00	0	0	1	1	1	0	1	1	1	1	1
1	1	59	2	145	53.1	2.40	25.2	107	68	237	48	189.00	6.10	0	2.00	0	0	1	0	1	0	1	0	1	0	1
1	1	68	2	147	54.9	1.30	25.5	154	78	201	39	162.00	5.50	1	1.00	1	1	1	0	1	0	0	0	0	0	1
1	1	74	2	152	53.3	-2.00	22.9	139	78	203	44	159.00	5.30	1	1.00	1	0	1	0	1	1	0	1	1	0	1
1	1	71	1	153	52.6	2.50	22.6	131	71	187	70	117.00	6.50	0	1.00	1	0	1	0	1	1	1	1	1	1	0
1	1	67	1	163	62.4	4.10	23.5	130	82	216	69	147.00	6.10	1	1.00	1	0	1	0	1	1	1	1	1	1	1
1	1	58	2	151	50.8	-1.10	22.2	118	67	245	79	166.00	5.40	1	1.00	1	1	1	0	0	1	1	1	1	0	1
1	1	64	2	149	45.6	-.50	20.6	106	66	199	88	111.00	5.40	1	1.00	1	1	1	1	1	1	1	1	1	1	1
1	1	65	2	159	53.1	-.60	20.9	92	53	213	74	139.00	5.70	1	1.00	1	0	1	1	1	1	1	1	1	1	1
1	1	82	2	146	52.3	-1.30	24.4	130	79	220	72	148.00	6.10	1	1.00	1	1	1	1	1	1	1	1	1	0	1
1	1	64	2	153	44.4	-.90	19.0	92	51	223	73	150.00	5.90	1	1.00	0	0	1	1	1	1	1	1	1	0	1
1	1	41	2	153	61.7	5.20	26.2	109	62	210	74	136.00	4.90	1	1.00	1	0	1	0	1	0	0	0	1	1	1
1	1	71	2	146	50.2	.10	23.5	122	74	270	96	174.00	5.60	1	1.00	1	0	1	1	1	1	1	1	1	0	1
1	1	73	1	164	82.2	1.40	30.6	121	82	152	34	118.00	5.50	0	1.00	1	0	1	1	0	1	1	0	1	0	1
0	0	69	2	149	45.1	-.50	20.2	127	73	222	96	126.00	6.40	1	1.00	1	0	1	0	0	1	1	1	1	0	0
1	1	69	2	145	46.9	-1.90	22.3	121	71	209	71	138.00	5.50	1	1.00	1	0	1	0	1	0	0	1	1	1	1
0	0	67	2	155	67.5	-.20	27.9	135	80	186	46	140.00	5.50	1	1.00	1	0	1	0	1	1	0	0	0	1	1
1	1	72	1	166	68.8	-1.10	25.0	122	72	178	69	109.00	5.90	1	1.00	1	0	1	1	1	1	1	0	1	1	1
1	1	66	2	152	49.4	3.20	21.5	93	59	170	85	85.00	5.60	1	1.00	1	1	1	1	1	1	1	1	0	0	1
1	1	74	2	148	52.6	-1.80	24.1	104	60	218	71	147.00	5.70	1	1.00	1	1	1	1	1	1	1	1	1	1	1
0	0	37	2	164	48.2	.00	17.8	108	66	164	72	92.00	5.00	1	1.00	1	1	1	1	1	1	1	1	1	1	1
0	0	56	2	164	75.3	2.00	27.9	133	75	249	69	180.00	5.80	1	1.00	1	0	1	1	1	1	1	0	0	0	1
1	1	84	1	156	46.7	1.70	19.3	97	56	151	72	79.00	5.40	1	1.00	0	1	1	1	1	1	1	1	1	1	1
0	0	83	2	149	40.7	2.40	18.4	127	71	227	83	144.00	5.60	1	1.00	1	0	1	0	1	0	1	1	1	0	1
1	1	65	1	173	61.6	1.60	20.6	123	81	210	88	122.00	5.20	1	1.00	0	1	1	1	1	1	0	1	1	1	1
1	1	59	2	154	53.5	-.30	22.5	144	88	216	55	161.00	5.80	1	1.00	1	1	1	1	1	1	1	1	0	1	0
1	1	76	1	175	64.7	-2.70	21.2	138	94	184	36	148.00	6.80	1	1.00	0	0	1	0	1	1	1	1	0	0	0
1	1	60	1	159	65.0	-4.60	25.7	132	83	286	80	206.00	5.40	1	1.00	0	0	1	1	0	0	0	0	1	0	1
1	1	48	2	170	54.4	-2.40	18.9	103	63	164	73	91.00	5.40	1	1.00	1	0	1	0	0	1	1	1	1	1	1
1	1	67	2	140	48.3	-2.50	24.6	154	86	179	50	129.00	6.00	1	1.00	1	1	1	1	1	1	1	1	0	1	1
0	0	31	1	179	73.1	1.80	22.9	128	77	208	55	153.00	5.30	1	1.00	1	0	1	0	1	1	1	1	1	1	1
1	1	85	1	142	52.4	-2.30	26.1	130	59	189	59	130.00	5.20	1	1.00	1	0	1	1	1	1	1	0	0	1	1
0	0	38	2	163	49.9	6.30	18.7	130	71	193	79	114.00	5.10	0	1.00	0	0	1	0	1	0	1	1	1	1	1
1	1	76	2	138	54.3	-3.10	28.5	143	80	197	53	144.00	6.00	1	1.00	1	1	1	0	1	0	0	0	0	1	1
1	1	85	2	141	52.5	-.50	26.3	155	80	210	44	166.00	5.80	1	1.00	1	1	1	1	1	1	1	0	0	1	1
1	1	62	2	153	58.8	-3.30	25.0	137	73	214	60	154.00	6.00	1	1.00	0	1	1	1	0	1	1	0	0	1	1
1	1	59	1	155	65.0	-1.00	26.9	137	77	211	63	148.00	5.90	1	1.00	0	1	1	0	1	0	1	0	0	1	1

1	1	85	1	159	58.9	.20	23.2	114	65	179	43	136.00	5.40	1	1.00	1	0	1	0	1	0	1	1	1	1	1
1	1	65	2	146	48.9	-1.20	23.0	123	64	209	55	154.00	5.50	1	1.00	1	0	1	0	1	0	0	1	0	1	1
0	0	67	2	151	58.0	-3.70	25.3	130	74	197	61	136.00	6.70	1	1.00	1	0	1	0	1	0	0	0	0	1	0
0	0	78	1	151	55.3	-.70	24.2	127	72	156	49	107.00	6.30	1	1.00	1	1	1	0	1	1	1	1	1	1	0
1	1	76	1	157	61.5	-.30	24.9	115	75	189	51	138.00	5.80	1	1.00	0	0	1	1	1	1	1	1	1	1	1
1	1	52	2	162	68.3	-.30	26.0	122	72	219	45	174.00	6.60	1	1.00	1	1	1	0	1	1	1	0	1	1	0
1	1	63	1	165	63.9	.20	23.3	139	89	222	96	126.00	5.20	1	1.00	0	1	1	0	0	1	1	1	0	0	1
1	1	72	2	138	55.2	1.30	29.0	137	70	146	61	85.00	5.50	1	1.00	1	0	1	1	1	0	0	0	0	1	1
1	1	74	2	145	42.5	3.50	20.3	114	68	176	44	132.00	5.30	1	1.00	1	1	1	1	1	1	1	1	1	1	1
1	1	78	2	137	43.8	-.90	23.4	145	82	271	119	152.00	5.50	1	1.00	1	0	1	1	1	1	1	1	0	0	1
1	1	19	2	140	43.5	-3.40	22.0	123	68	190	95	95.00	5.30	1	1.00	1	1	1	0	0	0	1	1	1	1	1
1	1	57	2	154	49.6	-1.50	20.8	144	86	226	100	126.00	5.40	1	1.00	1	0	1	1	1	0	1	1	0	0	0
1	1	72	1	163	65.4	-2.30	24.6	149	91	156	47	109.00	5.50	1	1.00	1	0	1	0	1	0	1	1	0	1	1
1	1	85	1	154	57.2	1.70	24.2	137	65	179	35	144.00	5.60	1	1.00	1	0	1	1	1	1	1	1	0	0	1
1	1	61	2	153	45.1	-.50	19.1	121	77	162	68	94.00	5.40	1	1.00	0	1	1	1	1	0	1	1	0	1	1
1	1	84	2	138	55.2	1.10	28.8	157	79	175	44	131.00	6.10	1	1.00	1	1	1	1	1	0	1	0	0	0	1
1	1	67	2	149	69.7	-2.40	31.5	146	90	187	43	144.00	6.40	1	1.00	1	0	1	1	1	1	0	0	0	1	1
1	1	66	2	155	56.4	-7.10	23.6	157	82	231	72	159.00	6.00	1	1.00	1	1	1	1	1	1	1	1	0	0	1
1	1	75	2	144	53.4	1.90	25.7	159	76	167	56	111.00	5.40	1	1.00	1	0	1	0	1	1	1	0	0	1	1
0	0	67	2	157	48.1	-1.20	19.5	127	77	215	64	151.00	6.10	1	1.00	0	0	1	1	1	0	0	1	0	0	1
1	1	77	2	144	71.5	-.50	34.4	151	84	166	57	109.00	5.70	1	1.00	1	0	1	0	1	1	1	0	0	1	1
1	1	71	2	151	49.2	1.20	21.5	124	77	200	77	123.00	5.70	1	1.00	1	1	1	1	1	0	0	1	0	0	1
1	1	64	2	149	52.5	-.90	23.6	142	92	206	54	152.00	6.00	1	1.00	1	0	1	0	0	1	1	1	0	1	1
0	0	79	1	159	55.1	-2.40	21.7	139	72	194	55	139.00	5.60	0	1.00	0	0	1	0	1	1	1	1	0	1	1
1	1	56	2	148	48.8	-2.10	22.3	111	68	296	74	222.00	5.80	1	1.00	0	0	1	1	1	1	1	1	1	0	1
1	1	54	2	161	59.7	3.90	23.0	103	54	222	56	166.00	5.70	1	1.00	1	0	1	1	1	1	0	1	1	0	1
1	1	73	1	150	58.5	-1.40	25.8	124	74	161	60	101.00	5.90	1	1.00	0	0	1	1	1	1	1	0	0	1	1
1	1	74	1	158	69.8	-1.40	27.8	126	72	203	52	151.00	5.70	1	1.00	0	0	1	0	1	0	0	0	1	1	1
0	0	56	2	157	45.9	-.90	18.5	113	74	194	81	113.00	5.60	1	1.00	0	0	1	0	1	1	1	1	1	1	1
0	0	75	2	150	46.3	-.90	20.7	118	61	170	50	120.00	6.70	1	1.00	1	0	1	0	1	1	0	1	0	1	0
1	1	67	2	151	54.2	-.10	23.7	140	77	183	66	117.00	5.70	1	1.00	1	0	1	1	1	1	1	1	0	1	1
1	1	60	2	160	61.5	-1.20	23.9	110	67	310	62	248.00	6.10	1	1.00	1	0	1	0	1	1	0	1	1	0	1
1	1	62	2	149	40.8	-1.20	18.2	102	55	218	78	140.00	5.40	1	1.00	0	1	1	1	1	1	1	1	1	1	1
1	1	38	2	152	53.7	-1.90	23.2	119	65	197	77	120.00	5.00	0	1.00	0	1	1	0	1	1	1	1	1	1	1
1	1	72	1	174	65.0	-.70	21.5	150	82	223	45	178.00	5.50	1	1.00	1	1	1	1	1	1	1	1	0	0	1
1	1	77	1	161	65.1	.10	25.2	130	72	229	50	179.00	5.40	0	1.00	1	1	1	1	1	0	1	0	1	0	1
0	0	56	1	168	79.7	6.40	28.1	110	69	226	64	162.00	6.00	1	1.00	1	0	1	0	1	1	0	0	1	0	1
1	1	62	1	168	71.3	1.40	25.2	139	84	226	42	184.00	5.70	1	1.00	1	0	1	1	1	1	1	0	0	0	1
1	1	60	1	167	65.4	1.40	23.3	131	80	251	65	186.00	5.70	1	1.00	1	1	1	0	1	1	1	1	1	0	1

1	1	63	2	144	53.0	.10	25.4	111	77	241	46	195.00	5.80	1	1.00	1	0	1	1	1	1	1	0	1	0	1
0	0	79	2	138	43.1	1.20	22.7	140	90	228	70	158.00	5.70	1	1.00	1	0	1	1	1	1	1	1	0	0	1
1	1	62	1	165	75.3	-1.30	27.6	122	79	181	39	142.00	7.60	1	1.00	0	0	1	1	1	0	0	0	0	0	0
1	1	41	1	167	67.5	1.10	24.2	152	101	259	47	212.00	5.40	0	1.00	0	1	1	0	0	1	1	1	0	0	1
1	1	64	2	152	50.4	-2.00	21.9	110	77	239	63	176.00	5.20	1	1.00	1	1	1	0	1	0	0	1	1	0	1
0	0	61	1	168	69.5	.00	24.5	123	77	176	48	128.00	5.50	1	1.00	1	1	1	0	1	1	0	1	0	1	1
0	0	74	2	146	51.1	-.30	23.9	106	76	295	53	242.00	5.30	1	1.00	1	1	1	0	1	1	1	1	0	0	1
0	0	70	2	149	51.5	-.80	23.1	109	61	186	54	132.00	5.70	1	1.00	1	0	1	1	1	0	0	1	1	1	1
0	0	71	1	163	72.7	.70	27.2	174	107	195	86	109.00	5.70	0	1.00	0	1	1	1	1	1	1	0	0	1	1
0	0	36	1	172	65.4	-.80	22.1	111	65	231	71	160.00	5.70	1	1.00	0	1	1	1	1	1	1	1	1	0	1
0	0	67	1	167	53.7	-.20	19.2	109	65	197	76	121.00	6.30	1	1.00	0	1	1	1	1	1	0	1	1	1	1
1	1	64	1	164	61.6	-3.00	22.9	110	65	200	84	116.00	5.40	0	1.00	0	0	1	0	1	0	0	1	1	1	1
0	0	69	2	159	51.2	.30	20.2	116	69	220	66	154.00	5.10	1	1.00	1	0	0	0	1	1	1	1	0	0	1
1	1	68	1	162	61.3	2.40	23.5	126	79	202	82	120.00	5.50	0	1.00	0	0	1	1	1	1	1	1	1	1	1
0	0	27	2	156	50.9	1.60	20.9	97	54	183	79	104.00	5.00	1	1.00	0	0	0	1	1	1	1	1	1	1	1
1	1	75	2	149	47.4	.70	21.4	112	69	157	60	97.00	5.30	1	1.00	1	0	1	1	1	1	0	1	0	1	1
1	1	66	1	167	64.5	-1.40	23.2	120	75	158	65	93.00	5.40	1	1.00	1	0	1	1	1	0	0	1	1	1	1
1	1	29	1	176	77.9	6.70	25.2	115	69	174	48	126.00	5.40	0	1.00	1	1	1	0	1	1	1	0	1	1	1
1	1	78	1	163	56.4	4.70	21.2	129	71	216	48	168.00	6.30	1	1.00	0	0	1	0	1	1	1	1	1	1	1
1	1	70	2	154	76.5	-1.00	32.4	141	76	249	61	188.00	6.40	1	1.00	0	0	1	0	1	0	0	0	0	0	1
1	1	69	1	156	69.1	-1.60	28.3	141	85	212	47	165.00	6.90	1	1.00	0	0	1	0	1	0	1	0	0	1	0
0	0	77	1	169	57.9	-.70	20.2	122	71	207	45	162.00	5.70	1	1.00	0	0	1	1	1	1	0	1	1	1	1
0	0	73	1	160	52.5	-4.10	20.4	122	80	216	50	166.00	5.60	1	1.00	1	1	1	1	1	1	1	1	1	1	1
0	0	59	2	157	52.5	-.40	21.2	123	75	219	45	174.00	5.40	1	1.00	0	0	1	1	1	0	1	1	1	1	1
1	1	80	1	158	62.8	2.80	25.3	149	89	211	47	164.00	5.80	1	1.00	0	0	1	1	1	1	1	0	0	1	1
0	0	76	2	153	49.7	.00	21.2	158	102	216	48	168.00	5.60	1	1.00	1	0	1	0	1	0	0	1	0	1	1
1	1	71	2	148	56.0	-2.00	25.5	132	71	172	50	122.00	5.50	1	1.00	1	0	1	0	1	0	0	0	1	1	1
1	1	30	2	159	50.6	-.10	20.1	105	67	157	73	84.00	5.50	1	1.00	1	1	1	1	1	1	1	1	1	1	1
1	1	52	1	166	64.7	.80	23.3	147	90	138	51	87.00	5.60	0	1.00	0	0	1	0	1	1	0	1	0	0	1
1	1	75	1	155	74.6	-.90	31.1	136	85	183	56	127.00	5.50	1	1.00	0	0	1	1	1	1	1	0	0	1	1
1	1	71	1	154	66.2	-.50	27.7	148	85	163	70	93.00	6.90	1	1.00	0	1	1	1	1	0	1	0	0	1	0
1	1	71	1	168	59.6	1.20	21.0	168	93	214	97	117.00	5.00	1	1.00	0	0	1	1	1	1	1	1	0	1	1
1	1	49	2	160	62.1	1.60	24.1	117	73	238	47	191.00	5.00	1	1.00	0	0	1	0	0	1	0	1	1	0	1
1	1	55	1	172	82.3	-.80	27.7	118	72	259	56	203.00	7.90	0	1.00	1	1	1	0	0	0	0	0	1	0	0
1	1	72	1	160	69.6	-.90	27.0	146	79	210	84	126.00	5.20	0	1.00	0	1	1	0	1	1	1	0	0	1	1
1	1	30	2	157	51.9	-1.50	20.9	103	60	176	81	95.00	4.90	1	1.00	1	1	1	0	1	1	1	1	1	1	1
1	1	67	2	154	56.3	-.50	23.8	138	78	184	61	123.00	5.30	1	1.00	1	1	1	0	0	1	1	1	1	1	1
1	1	73	2	150	65.2	-.10	28.9	135	78	242	69	173.00	6.00	1	1.00	1	0	1	1	1	0	0	0	0	0	0
1	1	56	2	151	49.7	-3.40	21.8	122	68	255	77	178.00	5.50	1	1.00	1	1	1	1	0	0	1	1	0	0	1

1	1	52	1	166	74.1	.90	26.7	133	76	206	52	154.00	5.30	1	1.00	0	0	1	0	1	0	0	0	0	1	1
0	0	69	2	155	56.1	2.30	23.4	142	75	229	75	154.00	6.10	1	1.00	1	1	1	1	1	1	0	1	0	0	1
0	0	38	2	160	53.7	2.00	20.9	107	63	164	75	89.00	5.40	0	1.00	1	1	1	0	1	1	0	1	1	1	1
1	0	59	1	166	57.2	-1.00	20.7	117	65	265	99	166.00	5.30	1	1.00	1	1	1	0	1	0	1	1	1	0	1
0	0	58	2	149	46.5	-1.20	21.0	111	59	191	49	142.00	5.50	1	1.00	1	0	1	1	1	0	1	1	1	1	1
1	1	74	2	155	46.1	-2.50	19.1	84	45	260	48	212.00	6.20	1	1.00	1	0	1	1	1	0	1	1	0	0	0
1	1	74	2	144	61.0	.90	29.3	148	79	213	63	150.00	5.70	1	1.00	1	0	1	1	1	1	1	0	0	0	1
1	1	63	1	169	62.3	-2.00	21.8	105	70	209	64	145.00	5.50	0	1.00	0	0	1	0	1	0	1	1	1	1	1
1	1	75	2	147	46.2	-.20	21.4	141	73	177	47	130.00	5.80	1	1.00	1	0	1	0	1	1	1	1	0	1	1
0	0	58	1	129	47.9	-1.70	28.6	161	84	223	43	180.00	5.80	0	1.00	0	0	1	0	1	0	0	0	0	0	1
1	1	43	1	169	67.0	-1.80	23.4	132	84	218	68	150.00	6.40	1	1.00	0	0	1	0	0	1	1	1	1	1	1
0	0	61	1	167	67.4	1.50	24.2	171	111	175	61	114.00	5.10	1	1.00	0	0	1	0	1	1	1	1	0	1	1
0	1	62	1	168	66.1	-1.70	23.3	126	76	177	46	131.00	5.20	0	1.00	0	0	1	0	1	0	0	1	1	1	1
0	0	62	1	160	62.5	-3.70	24.5	132	82	166	49	117.00	5.60	1	1.00	1	0	1	1	1	1	1	1	1	0	1
1	1	73	2	142	41.0	-.20	20.4	130	74	206	65	141.00	5.80	1	1.00	1	0	1	1	1	1	1	1	1	1	1
1	1	52	2	149	49.8	3.10	22.4	130	80	206	90	116.00	5.40	1	1.00	1	1	1	1	0	1	1	1	1	1	1
1	1	46	2	154	66.2	-1.60	27.9	137	92	191	71	120.00	5.00	1	1.00	1	0	1	1	0	1	1	0	0	1	1
1	1	42	1	167	57.7	.60	20.5	113	78	147	86	61.00	5.40	1	1.00	0	0	1	1	1	1	1	1	1	1	1
0	0	42	1	169	69.9	5.90	24.4	171	115	186	57	129.00	5.20	1	1.00	1	0	0	0	1	1	1	1	0	1	1
0	0	52	1	171	90.1	-.50	30.8	152	104	134	53	81.00	5.30	1	1.00	0	0	1	1	0	1	1	0	0	1	1
1	1	76	2	144	61.5	1.50	29.8	138	78	192	92	100.00	6.10	1	1.00	1	0	1	0	1	1	1	0	0	0	1
1	1	71	2	140	58.4	-.60	29.7	142	82	216	78	138.00	5.40	1	1.00	1	0	1	0	1	0	1	0	0	1	1
1	1	71	2	151	49.2	.10	21.6	128	85	217	85	132.00	5.80	1	1.00	1	0	1	1	1	0	1	1	0	1	1
1	1	43	1	174	75.4	-.20	24.9	119	73	199	57	142.00	5.50	1	1.00	0	0	1	0	1	0	1	1	1	1	1
1	1	36	2	157	62.6	7.60	25.3	116	73	158	60	98.00	5.30	1	1.00	1	0	1	0	1	0	0	0	1	1	1
1	1	80	1	156	61.1	.70	25.2	136	67	185	55	130.00	5.80	1	1.00	0	0	1	1	0	1	1	0	0	1	1
0	0	48	2	160	50.8	-2.60	19.9	126	72	243	105	138.00	5.70	1	1.00	1	0	1	0	0	0	0	1	1	0	1
1	1	80	1	155	62.8	2.90	26.2	116	78	173	53	120.00	6.30	1	1.00	1	0	1	0	1	0	1	0	0	1	1
0	0	74	2	150	63.3	5.60	28.3	115	64	247	58	189.00	6.00	1	1.00	1	0	1	0	1	0	0	0	1	0	1
1	1	69	2	152	61.1	-1.80	26.3	137	79	224	89	135.00	5.70	1	1.00	1	0	1	0	1	0	0	0	0	0	1
1	1	74	1	161	71.5	-3.00	27.5	143	83	171	54	117.00	5.70	1	1.00	0	0	1	0	1	1	1	0	0	0	1
1	1	44	1	168	80.0	.50	28.4	118	75	211	51	160.00	5.40	0	1.00	0	1	1	0	1	1	1	0	0	1	1
1	1	56	2	155	54.2	-5.00	22.6	135	94	238	54	184.00	5.50	1	1.00	1	0	1	0	0	0	1	1	0	0	1
1	1	29	2	160	42.6	1.80	16.7	98	64	193	89	104.00	5.10	0	1.00	1	1	1	0	1	0	1	1	1	1	1
1	1	64	2	160	48.8	3.70	19.1	117	64	197	78	119.00	6.30	1	1.00	1	0	1	1	1	1	1	1	1	1	1
1	1	71	2	143	53.1	2.40	25.9	144	70	194	55	139.00	5.20	1	1.00	1	0	1	0	1	0	0	0	0	0	1
1	1	64	2	153	61.8	-.10	26.5	98	65	161	54	107.00	5.90	1	1.00	1	0	1	0	1	1	1	0	0	1	1
1	1	81	1	164	59.1	4.00	22.0	136	74	195	71	124.00	5.70	1	1.00	1	0	1	1	1	1	1	1	0	1	1
1	1	66	2	147	52.1	-1.30	24.0	109	61	198	85	113.00	5.80	1	1.00	1	0	1	0	1	1	0	1	1	1	1

1	1	73	2	146	45.2	1.20	21.2	162	90	170	43	127.00	5.60	1	1.00	1	0	1	0	1	1	1	1	0	1	1
1	1	47	2	154	47.0	-3.20	19.8	106	56	224	79	145.00	5.40	1	1.00	0	0	1	0	1	0	1	1	1	0	1
1	1	52	1	157	67.8	1.40	27.4	148	84	257	54	203.00	5.70	0	2.00	0	0	1	0	1	1	1	0	0	0	1
1	1	73	2	158	55.0	4.60	22.1	118	66	170	96	74.00	5.20	1	1.00	1	0	1	0	1	0	0	1	0	1	1
1	1	57	2	157	55.6	2.50	22.6	143	78	186	70	116.00	5.00	1	1.00	1	0	1	0	0	0	1	1	0	1	1
1	1	76	1	161	65.1	2.30	25.2	127	77	200	71	129.00	5.70	1	1.00	1	1	1	1	1	1	1	0	1	1	1
1	1	70	1	160	69.0	3.50	27.0	110	62	196	88	108.00	5.50	1	1.00	0	0	1	0	1	1	1	0	0	1	1
1	1	42	2	161	49.7	-1.30	19.1	109	58	144	53	91.00	5.00	0	1.00	0	0	1	0	1	1	1	1	1	1	1
1	1	71	2	152	52.0	.70	22.6	119	58	183	49	134.00	6.00	1	1.00	1	1	1	1	1	0	0	1	0	1	1
1	1	29	2	149	44.4	1.10	19.9	116	69	198	109	89.00	5.10	1	1.00	1	0	1	1	1	1	1	1	1	1	1
1	1	70	2	147	58.4	-1.00	27.2	151	74	150	83	67.00	5.90	1	1.00	1	0	1	1	1	0	1	0	0	1	0
1	1	71	2	144	46.7	-.30	22.6	132	70	172	75	97.00	5.90	1	1.00	1	1	1	1	1	1	1	1	1	1	1
0	0	88	2	147	38.3	-2.60	17.7	147	75	117	59	58.00	5.60	1	1.00	1	1	1	1	1	1	1	1	0	1	1
1	1	77	1	169	68.2	-1.20	23.8	132	83	187	88	99.00	5.40	1	1.00	0	0	1	0	1	1	1	1	0	1	1
1	1	70	1	168	68.5	-4.40	24.1	138	86	213	87	126.00	6.20	1	1.00	0	0	1	1	1	0	0	1	0	1	1
0	0	42	1	172	62.4	.10	20.9	127	83	195	133	62.00	5.30	0	1.00	0	0	1	0	1	0	0	1	1	1	1
1	1	54	2	154	63.1	3.90	26.7	110	71	253	72	181.00	6.00	1	1.00	1	0	1	0	0	1	1	0	1	0	1
1	1	70	2	148	49.2	-1.10	22.3	143	79	270	62	208.00	5.20	1	1.00	1	1	1	1	1	1	1	1	0	0	1
1	1	71	1	164	61.4	-2.70	22.9	143	86	283	87	196.00	5.40	1	1.00	1	0	1	1	0	0	0	1	0	0	1
1	1	31	2	147	54.5	2.00	25.3	115	68	148	65	83.00	4.90	1	1.00	1	1	1	0	1	0	1	0	1	1	1
1	1	64	2	159	46.3	.10	18.4	193	108	140	92	48.00	5.30	1	1.00	1	0	1	0	1	0	1	1	0	1	1
1	1	61	1	164	70.7	-1.50	26.3	166	100	214	80	134.00	5.50	0	1.00	0	0	1	0	1	0	1	0	0	1	1
1	1	66	2	148	32.8	-1.90	14.9	122	68	211	89	122.00	5.70	1	1.00	1	0	1	1	1	0	0	1	1	1	1
1	1	61	2	161	57.0	1.20	21.8	106	64	274	57	217.00	5.10	1	1.00	1	0	1	0	1	0	0	1	1	0	1
1	1	37	2	157	56.0	-.10	22.6	108	60	196	93	103.00	5.10	1	1.00	1	1	1	0	1	1	1	1	1	1	1
0	0	63	1	164	60.0	-.20	22.4	122	80	168	37	131.00	8.00	1	1.00	0	0	1	0	1	1	1	1	1	0	0
0	0	83	2	139	39.2	-3.50	20.2	135	74	194	63	131.00	6.30	1	1.00	1	0	1	0	1	1	0	1	0	1	1
1	1	67	2	155	61.7	-1.40	25.5	129	74	183	95	88.00	6.10	1	1.00	1	0	1	1	1	1	1	0	0	0	1
0	0	74	2	150	52.1	1.20	23.0	130	72	170	54	116.00	5.70	1	1.00	1	1	1	1	1	1	1	1	0	1	1
1	1	44	1	178	69.9	2.40	22.1	138	88	228	53	175.00	5.30	0	1.00	0	0	1	0	1	1	1	1	1	0	1
0	0	41	2	161	62.1	5.80	23.8	113	71	177	75	102.00	5.30	1	1.00	0	0	1	1	1	0	0	1	1	1	1
0	0	69	2	158	45.3	-.80	18.2	125	62	173	71	102.00	6.60	1	1.00	1	0	1	0	1	1	1	1	0	1	0
1	1	61	2	154	46.0	-1.20	19.4	114	67	216	48	168.00	5.70	1	1.00	1	0	1	0	1	1	1	1	1	1	1
0	0	71	2	157	57.5	.40	23.4	124	76	159	55	104.00	4.70	1	1.00	1	0	1	1	1	1	1	1	0	1	1
0	0	80	2	146	44.1	-.80	20.6	152	74	176	73	103.00	5.20	1	1.00	1	0	1	0	1	1	1	1	0	1	1
1	1	57	2	148	79.3	.20	36.3	133	61	178	54	124.00	5.70	1	1.00	1	0	1	1	1	1	1	0	0	0	0
1	1	49	2	161	60.3	-1.60	23.2	95	59	151	76	75.00	5.60	1	1.00	1	0	1	0	1	1	1	1	1	1	1
1	1	62	2	148	63.8	.80	29.0	122	82	184	66	118.00	6.30	0	1.00	0	0	1	0	1	0	0	0	1	1	1
0	0	58	2	147	68.9	.20	31.7	127	84	293	62	231.00	5.30	1	1.00	1	1	1	0	1	0	0	0	1	0	1

0	0	77	2	149	50.1	-1.70	22.5	138	66	179	71	108.00	5.40	1	1.00	1	0	1	0	1	0	0	1	0	1	1
1	1	70	2	154	57.2	-1.60	24.1	134	69	276	92	184.00	5.50	1	1.00	1	1	1	1	1	0	0	1	1	0	1
1	1	62	2	151	55.8	.20	24.4	105	73	184	41	143.00	5.60	1	1.00	1	0	1	1	1	1	1	1	1	1	1
0	0	68	2	156	62.4	2.00	25.6	130	76	185	64	121.00	5.80	1	1.00	1	1	1	0	1	0	0	0	0	1	1
1	1	72	1	161	67.1	-1.20	25.9	123	73	175	40	135.00	5.80	1	1.00	1	1	1	0	0	0	0	0	1	1	1
1	1	67	2	144	51.6	-.70	24.8	130	74	185	67	118.00	6.10	1	1.00	1	0	1	1	1	1	1	1	0	1	0
1	1	64	2	151	50.9	1.60	22.4	136	88	186	51	135.00	5.60	1	1.00	1	0	1	0	0	0	1	1	1	1	1
1	1	70	1	157	60.8	-.20	24.7	148	90	196	45	151.00	5.70	1	1.00	0	0	1	1	1	1	1	1	0	1	1
0	0	47	1	171	102.5	-6.00	35.2	124	76	142	53	89.00	5.40	0	1.00	0	1	1	0	1	1	1	0	1	1	1
1	1	54	2	165	59.8	-2.90	22.0	179	100	231	77	154.00	5.80	1	1.00	1	0	1	1	1	0	1	1	0	0	1
1	1	78	2	148	53.1	-4.30	24.1	125	71	228	71	157.00	5.60	1	1.00	1	0	1	1	1	1	0	1	1	0	1
0	0	41	2	161	50.4	2.10	19.5	115	62	192	100	92.00	5.30	1	1.00	1	1	1	1	0	1	0	1	1	1	1
1	1	63	1	170	73.6	-2.20	25.5	138	85	210	50	160.00	5.50	1	1.00	0	0	1	0	1	1	0	0	1	1	1
0	0	31	2	154	65.0	-2.10	27.5	109	56	182	57	125.00	5.10	1	1.00	0	0	1	0	1	1	1	0	1	1	1
0	0	63	2	154	56.0	-3.00	23.4	132	74	288	53	235.00	5.20	1	1.00	1	1	1	0	1	0	0	1	0	0	1
0	0	63	2	151	50.1	.90	21.9	127	77	195	68	127.00	5.10	1	1.00	1	0	1	1	1	1	1	1	1	1	1
0	0	77	2	150	47.1	.10	20.8	109	58	205	57	148.00	4.80	1	1.00	1	1	1	1	1	0	1	1	1	1	1
0	0	71	1	160	68.2	-1.40	26.5	108	71	189	37	152.00	6.00	1	1.00	1	0	1	0	0	0	0	0	0	0	1
1	1	40	1	180	78.9	1.10	24.4	120	65	215	59	156.00	5.40	0	1.00	1	0	1	0	1	1	1	1	1	1	1
0	0	69	2	146	47.1	-.70	22.0	156	107	167	60	107.00	6.00	1	1.00	1	0	1	0	0	1	1	1	0	0	1
1	1	35	2	154	41.1	.00	17.3	108	68	224	75	149.00	5.30	1	1.00	1	1	0	0	1	0	1	1	1	0	1
1	1	63	2	158	56.4	-1.00	22.6	142	79	177	57	120.00	5.60	1	1.00	1	0	1	0	1	0	0	1	0	1	1
1	1	71	2	156	47.5	-2.30	19.4	144	70	174	45	129.00	6.60	1	1.00	1	0	1	0	0	1	1	1	0	0	0
1	1	66	2	151	56.3	-2.30	24.7	118	78	182	46	136.00	5.40	1	1.00	1	0	1	0	0	0	0	1	1	1	1
1	1	77	1	164	60.7	.50	22.4	131	81	164	67	97.00	5.10	1	1.00	0	0	1	0	1	1	1	1	1	1	1
1	1	68	1	145	52.1	.00	24.8	126	63	195	46	149.00	5.90	1	1.00	0	0	1	1	1	1	1	1	0	1	0
1	1	67	2	151	52.5	-1.60	23.1	112	72	193	87	106.00	5.90	1	1.00	1	0	1	1	1	0	0	1	1	1	1
0	0	67	2	145	51.0	1.10	24.3	108	61	178	65	113.00	5.90	1	1.00	1	0	1	1	1	1	1	1	0	1	1
1	1	67	2	153	65.5	-2.40	28.0	139	78	214	51	163.00	6.00	1	1.00	1	0	1	0	1	1	1	0	0	1	1
0	0	45	2	158	64.5	6.20	25.7	122	71	237	96	141.00	5.20	1	1.00	1	0	1	0	0	0	0	0	1	0	1
1	1	66	1	166	61.2	-.80	22.2	125	77	145	55	90.00	7.00	1	1.00	1	0	1	0	1	1	0	1	1	1	0
1	1	37	1	173	87.4	-13.40	29.0	143	80	196	53	143.00	5.70	1	1.00	1	0	1	0	1	0	1	0	0	1	1
0	0	76	1	159	64.2	-.50	25.4	129	73	185	56	129.00	6.30	1	1.00	0	0	1	0	1	1	0	0	0	1	1
1	1	53	1	169	67.1	-.90	23.5	163	107	277	52	225.00	6.00	1	1.00	0	0	1	0	1	0	1	1	0	0	1
0	0	70	2	149	43.5	.50	19.5	137	80	210	68	142.00	5.30	1	1.00	1	0	1	0	1	1	0	1	1	1	1
0	0	76	2	145	58.5	1.40	27.6	135	76	219	54	165.00	6.40	1	1.00	0	0	1	1	1	1	1	0	0	1	1
0	0	78	1	165	59.7	-3.80	22.0	132	64	209	62	147.00	7.20	1	1.00	1	0	1	0	1	0	0	1	0	1	0
1	1	54	1	181	70.5	-4.80	21.4	138	91	251	97	154.00	5.30	1	1.00	0	0	1	0	0	1	0	1	0	0	1
0	0	52	2	154	39.6	-1.90	16.6	128	65	248	85	163.00	5.10	0	1.00	0	0	0	1	0	0	0	1	1	0	1

1	1	50	2	148	44.2	-3.20	20.1	154	92	271	66	205.00	5.90	1	1.00	1	0	1	0	1	0	0	1	0	0	1
1	1	61	2	150	56.1	-2.30	24.8	112	69	346	42	304.00	6.20	1	1.00	1	1	1	0	1	1	1	1	1	0	1
0	0	32	1	174	63.3	4.40	20.9	147	80	195	78	117.00	5.00	1	1.00	1	1	1	0	1	1	1	1	0	1	1
1	1	75	2	149	62.8	.00	28.2	144	79	262	51	211.00	5.80	1	1.00	1	0	1	1	1	1	1	0	0	0	1
0	0	61	1	163	65.5	1.80	24.6	146	88	187	78	109.00	5.80	0	1.00	0	1	1	1	0	0	0	1	0	1	1
1	1	43	2	160	80.3	-1.00	31.4	132	86	224	38	186.00	6.10	0	1.00	1	0	1	0	1	1	1	0	1	0	1
1	1	39	2	172	69.4	3.80	23.5	122	78	213	72	141.00	5.50	1	1.00	1	0	1	1	1	0	0	1	1	1	1
1	1	70	1	155	56.1	-.70	23.2	149	81	193	62	131.00	5.70	1	1.00	1	0	1	0	1	0	0	1	0	1	1
1	1	53	2	153	64.1	.50	27.4	113	67	249	44	205.00	5.70	1	1.00	1	0	1	0	0	0	1	0	1	0	1
0	1	38	1	176	70.7	-.30	22.9	127	75	165	57	108.00	5.60	1	1.00	0	0	1	1	1	1	1	1	1	1	1
1	1	70	2	149	44.3	1.30	19.9	141	79	268	62	206.00	5.40	1	1.00	1	0	1	0	1	0	1	1	0	0	1
1	1	75	1	154	63.0	3.40	26.4	144	85	144	43	101.00	6.00	1	1.00	1	0	1	1	1	1	1	0	0	1	1
1	1	39	2	155	47.0	-5.10	19.5	135	88	246	103	143.00	5.30	1	1.00	0	0	1	0	0	0	0	1	1	0	1
1	1	67	2	155	62.9	-.50	26.1	151	82	263	52	211.00	5.90	1	1.00	1	0	1	0	1	1	1	0	0	0	1
0	0	39	2	162	53.4	2.80	20.3	102	56	279	51	228.00	5.50	0	1.00	1	1	1	0	1	0	1	1	1	0	1
0	0	76	2	148	43.4	-.10	19.7	139	69	234	88	146.00	5.40	1	1.00	1	0	1	0	1	1	0	1	0	0	1
0	0	71	2	150	48.1	3.90	21.3	157	84	197	78	119.00	5.50	1	1.00	1	0	1	1	1	0	1	1	0	0	1
1	1	84	1	162	57.1	.20	21.8	150	83	244	51	193.00	5.90	1	1.00	1	0	1	1	1	1	0	1	0	0	1
0	0	77	2	152	61.0	2.20	26.4	132	76	236	70	166.00	6.10	1	1.00	1	0	1	1	1	1	0	0	1	0	1
0	0	74	2	136	51.5	.40	27.8	129	62	207	93	114.00	5.90	1	1.00	1	0	1	0	1	0	1	0	0	1	0
0	0	73	1	153	60.4	1.00	25.9	139	75	210	48	162.00	5.40	1	1.00	0	0	1	0	1	1	1	0	1	1	1
0	0	76	2	153	69.0	-1.00	29.4	140	73	219	60	159.00	5.40	1	1.00	1	0	1	0	1	1	1	0	0	0	1
1	1	52	2	155	52.0	-1.60	21.7	137	78	166	51	115.00	5.30	1	1.00	1	0	1	0	1	1	0	1	1	1	1
0	0	51	1	169	77.6	-4.80	27.1	133	83	238	46	192.00	7.40	0	1.00	1	1	1	0	0	0	0	0	1	0	0
1	1	64	1	170	64.1	3.10	22.1	126	82	161	58	103.00	5.90	0	1.00	0	1	1	1	1	1	1	1	0	1	1
1	1	61	2	149	56.8	.00	25.4	102	69	191	57	134.00	6.00	1	1.00	1	0	1	1	0	1	1	0	1	1	1
0	0	51	2	156	55.6	1.10	22.9	137	86	179	30	149.00	5.10	1	1.00	1	0	1	0	1	0	0	1	0	0	1
0	0	80	1	152	65.1	.10	28.1	135	65	255	54	201.00	6.10	1	1.00	1	0	1	0	1	0	0	0	1	0	1
0	0	64	1	167	59.0	-1.00	21.1	137	77	221	83	138.00	6.10	0	1.00	0	0	1	0	1	1	1	1	1	0	1
0	0	59	2	161	48.1	-1.40	18.4	102	71	228	75	153.00	5.90	1	1.00	1	0	1	0	1	0	0	1	1	0	1
1	1	74	2	154	49.6	-.40	20.8	121	66	198	61	137.00	5.70	1	1.00	1	0	1	0	1	1	0	1	1	1	1
0	0	45	2	154	53.1	-4.50	22.3	113	69	242	55	187.00	5.20	0	1.00	1	1	1	1	0	1	1	1	1	0	1
1	1	66	1	170	76.3	6.00	26.4	120	88	189	31	158.00	6.30	1	1.00	1	0	1	1	1	0	1	0	1	0	1
1	1	78	1	159	55.3	1.10	21.8	124	59	167	60	107.00	6.70	1	1.00	1	1	1	1	1	1	1	1	1	1	0
0	0	69	2	151	50.3	.60	22.1	121	70	240	42	198.00	5.40	1	1.00	1	1	1	0	1	0	0	1	1	0	1
1	1	73	1	157	65.0	2.30	26.3	141	84	247	74	173.00	5.80	1	1.00	0	1	1	1	1	0	1	0	0	0	1
1	1	64	1	172	58.8	-5.20	19.7	96	54	156	52	104.00	5.30	0	2.00	1	0	1	0	0	0	1	1	1	1	1
0	0	60	2	150	46.8	3.00	20.9	146	89	179	48	131.00	5.90	1	1.00	1	0	1	0	0	1	1	1	0	1	1
1	1	74	1	154	45.0	-.20	18.9	128	66	228	55	173.00	5.20	0	1.00	1	1	1	1	1	1	1	1	1	0	1

1	1	76	2	143	50.7	1.30	24.7	146	83	226	67	159.00	5.80	1	1.00	1	1	1	1	1	1	0	1	0	0	1
1	1	71	2	159	55.7	1.00	22.1	141	78	197	51	146.00	5.20	1	1.00	1	0	1	1	1	1	1	1	0	1	1
1	1	64	2	151	43.8	-.30	19.1	123	69	250	57	193.00	5.60	1	1.00	1	0	1	1	1	1	1	1	0	0	1
1	1	78	2	147	50.2	.50	23.3	185	97	158	41	117.00	5.50	1	1.00	1	0	1	1	1	0	0	1	0	1	1
0	0	40	1	173	97.5	.90	32.4	122	83	299	52	247.00	5.80	1	1.00	0	0	1	0	0	0	1	0	1	0	1
1	1	43	2	164	58.9	1.60	21.9	103	60	203	73	130.00	5.70	0	1.00	1	0	1	0	1	0	0	1	1	1	1
1	1	67	1	164	46.2	.90	17.2	133	86	210	87	123.00	5.50	0	1.00	1	0	0	1	1	0	0	1	1	1	1
1	1	40	2	150	46.9	3.00	20.7	118	72	184	59	125.00	5.40	1	1.00	0	0	1	0	1	0	0	1	1	1	1
1	1	71	2	147	46.9	-.60	21.8	126	72	215	46	169.00	5.50	1	1.00	1	0	1	1	1	0	1	1	1	1	1
1	1	61	2	154	55.0	1.50	23.2	102	66	235	64	171.00	5.70	1	1.00	0	1	1	0	1	0	0	1	1	0	1
0	0	68	1	152	53.2	-.70	22.9	142	86	213	45	168.00	5.30	0	1.00	1	1	1	0	0	0	0	1	0	1	1
0	0	80	2	150	36.1	1.20	16.0	153	87	243	94	149.00	6.10	1	1.00	1	1	1	0	1	1	0	1	0	0	1
1	1	66	1	167	60.8	.30	21.8	117	72	242	65	177.00	5.70	1	1.00	0	0	1	1	0	0	1	1	1	0	1
1	1	65	2	158	55.1	.40	22.0	141	81	204	71	133.00	5.40	1	1.00	1	1	1	1	1	1	1	1	0	1	1
1	1	58	2	153	62.0	-.10	26.4	112	73	276	94	182.00	5.70	1	1.00	1	0	1	0	1	1	1	0	1	0	1
1	1	73	1	168	65.7	-.40	23.3	148	82	202	71	131.00	6.50	1	1.00	0	0	1	0	1	1	1	1	0	1	0
0	0	65	2	152	57.2	.70	24.6	115	78	223	79	144.00	5.30	1	1.00	1	0	1	0	0	0	0	1	1	0	1
0	0	64	1	157	63.6	3.50	25.7	104	70	210	39	171.00	5.80	1	1.00	0	0	1	1	0	0	0	0	1	0	1
0	0	74	1	168	90.5	1.50	32.1	151	75	218	51	167.00	5.80	1	1.00	1	0	1	1	1	1	1	0	0	1	1
1	1	76	2	146	58.1	.00	27.4	156	82	160	36	124.00	5.60	1	1.00	1	0	1	1	1	1	1	0	0	0	1
1	1	36	2	157	57.1	2.50	23.1	106	64	206	67	139.00	5.70	1	1.00	1	0	1	0	1	1	1	1	1	1	1
1	1	68	1	163	64.0	.10	24.1	136	89	174	62	112.00	5.50	1	1.00	0	1	1	1	1	0	1	1	1	1	1
1	1	64	1	160	54.6	.50	21.3	150	83	296	102	194.00	5.40	1	1.00	0	0	1	1	0	1	1	1	0	0	1
0	0	83	1	158	62.0	2.60	24.8	123	73	229	32	197.00	5.40	1	1.00	1	0	1	1	1	1	1	1	1	0	1
0	0	69	2	149	43.2	2.60	19.3	125	73	274	93	181.00	5.70	1	1.00	1	0	1	1	1	1	1	1	1	0	1
1	1	68	2	150	51.6	.60	22.8	138	82	224	112	112.00	5.50	1	1.00	1	1	1	0	1	1	1	1	1	0	1
0	0	84	1	158	56.3	-2.10	22.5	128	64	171	66	105.00	5.60	1	1.00	1	0	1	0	1	0	1	1	0	1	1
1	1	65	2	151	46.5	1.00	20.4	152	79	170	58	112.00	5.60	1	1.00	1	0	1	1	1	0	0	1	0	1	1
0	1	38	2	168	74.8	3.80	26.5	116	69	175	55	120.00	5.30	0	1.00	1	0	1	0	1	1	1	0	1	1	1
1	1	80	1	166	60.0	-5.00	21.8	125	76	169	61	108.00	5.90	1	1.00	0	1	1	1	1	1	1	1	1	1	1
1	1	53	1	160	62.7	-.80	24.6	128	81	191	58	133.00	6.30	1	1.00	0	0	1	0	1	1	1	1	1	1	1
1	1	70	2	149	68.2	1.00	30.7	123	68	272	50	222.00	5.80	1	1.00	1	1	1	1	1	0	1	0	1	0	1
1	1	67	2	160	53.0	-3.40	20.6	107	72	181	62	119.00	5.30	1	1.00	1	0	1	0	1	1	1	1	1	1	1
1	1	61	2	154	64.8	-.80	27.2	136	83	216	65	151.00	5.60	1	1.00	1	0	1	0	1	1	1	0	1	1	1
0	0	66	1	162	65.7	.30	25.1	117	72	175	42	133.00	5.80	1	1.00	1	0	1	0	1	0	1	0	1	1	1
1	1	64	1	176	73.1	-.10	23.5	156	92	165	51	114.00	5.60	0	1.00	0	1	1	1	1	1	1	1	0	1	1
1	1	44	2	157	73.3	3.30	29.8	111	57	194	62	132.00	5.40	1	1.00	1	0	1	0	0	1	1	0	1	1	1
1	1	70	1	145	48.8	1.00	23.1	127	78	213	63	150.00	5.80	1	1.00	1	1	1	1	1	1	1	1	1	1	1
1	1	47	1	160	58.3	2.30	22.7	113	72	194	88	106.00	5.40	1	1.00	0	0	1	1	0	1	1	1	1	1	1

1	1	63	2	157	53.6	1.40	21.8	157	87	303	66	237.00	6.30	1	1.00	1	0	1	0	1	0	1	1	0	0	1
1	1	67	2	153	62.4	2.90	26.7	121	71	198	84	114.00	5.30	1	1.00	1	1	1	1	1	1	1	0	1	1	1
1	1	61	1	165	61.7	-.70	22.6	105	58	230	50	180.00	5.60	1	1.00	1	0	1	0	1	1	1	1	1	0	1
1	1	69	1	161	68.5	-.80	26.4	109	64	243	50	193.00	5.80	1	1.00	0	1	1	0	1	1	1	0	1	0	1
1	1	56	2	149	50.0	.00	22.6	144	95	241	72	169.00	5.80	1	1.00	1	0	1	0	1	1	1	1	0	0	1
1	1	80	2	135	37.0	-1.60	20.3	157	82	260	82	178.00	5.00	1	1.00	1	0	1	0	1	0	0	1	0	0	1
1	1	70	2	154	53.9	.50	22.6	152	93	214	38	176.00	7.20	1	1.00	1	0	1	0	1	1	0	1	0	0	0
1	1	34	1	173	54.6	-1.20	18.2	107	62	152	56	96.00	5.40	0	1.00	0	1	1	1	0	0	1	1	1	1	1
1	1	69	2	149	41.9	1.20	18.8	141	80	195	75	120.00	5.50	1	1.00	1	0	1	0	1	1	1	1	0	1	1
1	1	77	1	156	56.4	.70	23.1	132	74	220	77	143.00	5.20	0	1.00	0	1	1	1	1	1	1	1	1	0	1
1	1	72	2	149	49.2	.90	22.3	136	72	214	59	155.00	5.00	1	1.00	1	1	1	1	1	0	0	1	1	1	1
1	1	57	2	150	45.8	-4.60	20.4	95	52	163	64	99.00	5.20	1	1.00	1	1	1	0	0	1	1	1	1	1	1
1	1	69	2	139	38.4	1.70	19.8	120	66	216	69	147.00	5.20	1	1.00	1	0	1	0	1	0	0	1	1	1	1
1	1	79	2	156	66.4	3.70	27.3	143	66	244	68	176.00	5.70	1	1.00	1	0	1	1	1	1	1	0	0	0	1
0	0	65	1	159	62.4	1.90	24.6	129	72	176	60	116.00	5.80	1	1.00	0	0	1	0	1	1	1	1	1	1	1
0	0	39	1	171	89.0	1.10	30.4	122	75	212	46	166.00	5.60	1	1.00	0	0	1	1	1	1	1	0	1	1	1
1	1	42	2	153	47.1	9.70	20.0	121	67	154	48	106.00	4.60	1	1.00	1	0	1	0	0	0	1	1	1	1	1
1	1	37	2	161	53.1	4.60	20.5	119	67	206	61	145.00	5.40	1	1.00	1	0	1	0	1	0	1	1	1	1	1
0	0	71	2	149	77.7	2.10	35.1	139	77	242	52	190.00	6.30	1	1.00	1	0	1	0	0	0	1	0	0	0	1
1	1	71	1	165	64.1	.50	23.6	150	92	207	92	115.00	5.60	0	1.00	0	0	1	0	1	1	1	1	0	1	1
1	1	71	2	155	62.8	.30	26.1	133	71	186	69	117.00	6.30	1	1.00	1	0	1	0	1	0	1	0	1	1	1
1	1	28	2	146	41.0	2.70	19.2	96	57	141	52	89.00	5.70	1	1.00	1	0	1	0	1	1	1	1	1	1	1
1	1	73	2	148	46.1	.50	20.9	147	79	185	49	136.00	7.20	1	1.00	1	0	1	0	1	0	0	1	0	1	0
0	0	57	2	154	61.4	-1.70	25.9	125	76	219	50	169.00	5.70	1	1.00	1	0	1	0	1	1	1	0	0	1	1
0	0	73	1	159	65.3	-2.50	25.9	163	88	172	42	130.00	5.50	1	1.00	1	0	1	1	1	1	1	0	0	1	1
0	0	77	2	149	52.1	-.80	23.5	132	68	200	72	128.00	5.60	1	1.00	0	0	1	0	1	1	0	1	1	1	1
1	1	69	1	160	68.0	2.30	26.6	141	83	181	49	132.00	5.60	1	1.00	1	0	1	1	1	0	1	0	0	1	1
1	1	48	2	163	53.6	1.50	20.2	105	57	205	76	129.00	5.20	1	1.00	1	0	1	1	1	1	1	1	1	1	1
1	1	69	2	154	54.4	3.80	22.8	107	70	165	48	117.00	5.80	1	1.00	1	0	1	1	1	1	1	1	1	1	1
1	1	63	2	151	51.3	2.10	22.5	118	79	308	56	252.00	6.20	1	1.00	1	0	1	0	1	1	0	1	1	0	1
0	0	73	2	155	53.4	.20	22.2	141	77	225	39	186.00	5.60	1	1.00	1	0	1	1	1	0	0	1	0	0	1
1	1	55	2	148	44.9	.10	20.5	101	64	223	95	128.00	5.50	1	1.00	0	0	1	1	1	1	1	1	1	0	1
1	1	60	1	164	67.5	-2.80	25.2	123	80	159	67	92.00	5.30	0	1.00	0	0	1	1	1	1	0	0	1	1	1
0	0	63	2	154	66.0	.40	27.7	101	65	208	61	147.00	5.80	1	1.00	0	0	1	0	0	0	1	0	1	0	1
1	1	73	1	161	52.1	3.90	20.0	142	77	165	53	112.00	6.20	0	1.00	1	0	1	1	1	1	1	1	0	1	0
0	0	52	2	154	50.1	.40	21.0	106	71	205	76	129.00	5.40	1	1.00	1	0	1	0	1	1	1	1	1	1	1
1	1	69	1	167	58.7	-1.50	21.1	162	68	161	55	106.00	7.70	0	1.00	0	0	1	1	1	0	1	1	0	1	0
0	0	61	2	150	52.5	-1.00	23.2	130	83	279	59	220.00	6.10	1	1.00	1	0	1	1	0	0	1	1	1	0	1
1	1	62	2	150	62.6	2.00	27.9	157	85	234	64	170.00	5.60	1	1.00	1	0	1	1	1	0	1	0	0	0	1

1	1	63	1	155	56.6	1.20	23.7	163	83	188	76	112.00	6.30	1	1.00	0	0	1	0	0	0	0	1	0	1	1
1	1	78	2	143	56.1	1.30	27.3	125	59	299	66	233.00	6.20	1	1.00	1	1	1	1	1	1	1	0	1	0	1
1	1	49	2	160	74.3	1.40	28.9	127	84	242	48	194.00	6.00	1	1.00	0	0	1	0	1	0	1	0	1	0	1
1	1	59	2	152	63.9	2.80	27.6	144	73	234	62	172.00	6.20	1	1.00	1	1	1	1	1	0	1	0	0	0	1
0	0	63	1	168	66.0	3.90	23.4	117	75	226	48	178.00	5.60	1	1.00	1	0	1	0	1	0	1	1	1	0	1
1	1	63	1	157	68.0	1.30	27.5	114	67	152	51	101.00	5.80	1	1.00	0	1	1	0	0	0	1	0	1	1	1
0	0	68	1	163	66.4	.60	24.8	103	59	171	90	81.00	5.80	1	1.00	0	0	1	0	1	1	1	1	1	1	1
0	0	52	2	161	87.1	.40	33.6	120	68	187	58	129.00	5.90	1	1.00	1	0	1	0	0	0	0	0	1	0	1
1	1	45	1	165	61.0	-6.30	22.4	123	77	172	44	128.00	6.50	1	1.00	1	1	1	0	1	1	0	1	1	1	0
1	1	72	1	158	51.0	-1.40	20.3	136	71	205	69	136.00	6.30	1	1.00	1	0	1	1	1	1	0	1	1	1	1
0	0	75	2	148	44.7	-.90	20.3	122	78	206	57	149.00	6.30	1	1.00	1	1	1	1	0	1	1	1	1	1	0
1	1	79	2	147	60.4	-.70	27.9	133	70	176	59	117.00	6.20	1	1.00	1	1	1	1	1	1	1	0	1	1	1
1	1	75	1	163	71.4	-.80	26.8	146	88	171	54	117.00	6.80	1	1.00	1	0	1	0	1	0	1	0	0	1	0
1	1	63	2	157	44.0	-.40	17.9	112	60	289	52	237.00	5.80	1	1.00	1	1	1	1	0	0	0	1	1	0	1
0	0	64	1	165	72.3	-1.30	26.7	130	75	220	42	178.00	6.00	0	1.00	1	0	1	0	1	1	0	0	0	0	1
1	1	48	2	155	47.6	-3.40	19.8	100	66	200	84	116.00	5.40	0	1.00	1	1	1	1	1	1	1	1	1	1	1
1	1	56	2	154	57.7	-.60	24.4	108	65	228	67	161.00	5.30	1	1.00	1	1	1	0	1	1	1	1	1	0	1
1	1	79	2	147	53.2	.80	24.6	127	59	162	68	94.00	5.50	1	1.00	1	0	1	0	1	0	0	1	0	1	1
1	1	74	2	146	61.4	-.50	28.9	128	77	286	74	212.00	6.20	1	1.00	1	0	1	1	1	1	1	0	0	0	0
0	0	74	2	146	42.3	-1.60	19.7	105	63	163	60	103.00	5.70	1	1.00	1	0	1	0	1	0	0	1	1	1	1
1	1	76	1	162	69.3	3.90	26.5	130	79	233	62	171.00	5.80	1	1.00	1	0	1	1	0	1	0	0	1	0	1
0	0	69	2	145	44.1	-.20	20.9	120	74	210	93	117.00	5.50	1	1.00	1	0	1	0	1	1	1	1	1	0	1
0	0	77	2	147	38.9	-1.20	17.9	121	71	207	61	146.00	5.70	1	1.00	1	1	1	0	1	0	0	1	0	1	1
1	1	43	1	169	73.2	-2.40	25.6	118	72	242	43	199.00	5.40	1	1.00	1	0	1	0	1	1	1	0	1	0	1
1	1	63	2	148	71.1	-3.60	32.4	125	70	200	58	142.00	7.20	1	1.00	1	0	1	1	1	1	0	0	0	1	0
1	1	79	2	148	61.1	-2.70	28.0	109	68	200	38	162.00	8.00	1	1.00	1	0	1	1	1	1	0	0	0	0	0
1	1	77	2	145	49.1	-1.40	23.5	158	80	230	55	175.00	5.30	1	1.00	1	0	1	0	1	1	1	1	0	0	1
1	1	76	2	144	36.0	-.10	17.2	105	54	228	68	160.00	5.60	1	1.00	1	1	1	1	1	1	1	1	1	0	1
1	1	68	2	144	42.6	.60	20.4	116	76	173	81	92.00	5.70	1	1.00	1	0	1	0	1	0	0	1	1	1	1
1	1	77	2	142	51.9	.70	25.9	143	77	196	56	140.00	5.80	1	1.00	1	1	1	0	1	1	1	0	0	1	1
1	1	60	2	149	43.1	1.10	19.4	140	83	198	92	106.00	5.80	1	1.00	1	1	1	1	1	1	1	1	0	1	1
1	1	70	1	157	59.3	-6.70	24.0	137	80	137	50	87.00	5.80	0	1.00	0	0	1	1	1	1	1	1	0	1	1
0	0	40	1	171	71.0	1.30	24.2	109	62	220	47	173.00	5.50	1	1.00	0	1	1	0	1	1	1	1	1	0	1
0	0	62	1	168	62.1	-2.20	21.9	122	81	172	47	125.00	5.20	1	1.00	1	0	1	1	1	1	1	1	0	1	1
1	1	72	1	166	73.3	-4.20	26.5	154	84	172	65	107.00	6.50	0	1.00	0	0	1	1	1	1	1	0	0	1	0
1	1	75	2	132	53.5	1.10	30.6	108	57	167	43	124.00	5.90	1	1.00	1	1	1	1	1	1	1	0	1	1	1
1	1	59	2	155	67.1	-1.30	28.1	153	78	200	65	135.00	5.30	1	1.00	1	1	1	0	0	1	1	0	0	1	1
1	1	75	2	144	42.0	-2.70	20.1	155	82	263	83	180.00	5.80	1	1.00	1	1	1	1	1	1	1	1	0	0	1
1	1	84	1	161	68.4	-1.00	26.4	149	80	226	50	176.00	5.60	1	1.00	0	0	1	1	1	1	1	0	0	0	1

1	1	71	2	146	40.3	-1.20	19.0	142	82	231	85	146.00	5.40	1	1.00	1	0	1	1	1	0	1	1	0	0	1
1	1	73	1	167	54.6	-2.00	19.6	159	81	157	71	86.00	5.40	0	1.00	0	0	1	0	1	1	1	1	0	1	1
1	1	76	2	159	55.9	5.20	22.0	113	61	188	56	132.00	5.30	1	1.00	1	0	1	1	1	0	0	1	1	1	1
1	1	56	2	161	66.0	-2.30	25.6	124	70	213	66	147.00	5.90	1	1.00	1	1	1	0	1	0	0	0	1	1	1
1	1	77	1	167	65.8	.30	23.7	136	78	167	42	125.00	5.40	1	1.00	1	1	1	1	1	1	1	1	0	1	1
1	1	59	2	153	55.2	.90	23.4	114	68	265	74	191.00	5.20	1	1.00	1	0	1	0	1	0	0	1	1	0	1
0	0	67	1	167	45.6	2.40	16.3	140	91	214	78	136.00	5.90	1	1.00	1	1	1	0	1	1	1	1	0	1	1
0	0	69	2	143	47.3	1.30	23.2	142	98	216	48	168.00	5.80	1	1.00	1	0	1	0	1	0	0	1	0	1	1
0	0	74	1	156	53.9	-1.70	22.1	132	74	150	49	101.00	5.70	1	1.00	0	1	1	1	1	1	0	1	0	0	1
1	1	69	1	164	68.4	-1.20	25.4	128	72	190	55	135.00	6.20	1	1.00	1	0	1	0	0	1	1	0	0	1	1
0	0	70	2	157	47.4	2.20	19.3	157	87	218	52	166.00	5.40	1	1.00	1	1	1	1	1	1	1	1	0	1	1
1	1	60	1	174	72.5	1.70	24.0	139	92	210	48	162.00	5.50	1	1.00	0	0	1	1	1	1	1	1	0	1	1
1	1	80	2	141	41.5	.20	20.9	141	68	188	40	148.00	6.50	1	1.00	1	1	1	1	1	1	1	1	0	1	0
1	1	40	2	149	52.5	-.60	23.8	107	69	247	39	208.00	5.50	1	1.00	1	0	0	0	1	1	0	1	1	0	1
1	1	61	2	159	65.3	.80	25.7	159	93	193	45	148.00	5.50	1	1.00	1	0	1	1	1	1	0	0	0	1	1
1	1	62	2	151	59.5	1.90	26.0	153	94	198	59	139.00	5.90	1	1.00	1	0	1	1	1	1	1	0	0	1	1
1	1	67	2	153	49.5	-.20	21.1	116	72	255	42	213.00	5.50	1	1.00	1	0	1	0	1	1	1	1	1	0	1
0	0	83	2	147	55.9	1.10	25.9	155	73	197	44	153.00	5.40	1	1.00	1	0	1	1	1	0	1	0	0	1	1
0	1	67	2	150	39.0	1.40	17.3	101	62	204	117	87.00	6.30	1	1.00	1	0	1	1	1	1	0	1	1	1	0
1	1	58	2	149	57.6	.30	25.8	107	73	266	70	196.00	6.00	1	1.00	1	1	1	0	0	0	0	0	1	0	1
0	0	51	1	168	61.6	.10	21.9	162	97	212	34	178.00	6.00	0	1.00	1	1	1	1	1	1	1	1	0	0	1
0	0	77	2	138	46.3	-3.70	24.2	172	97	220	51	169.00	5.60	1	1.00	1	1	1	1	0	0	1	1	0	0	1
1	1	77	2	150	54.8	3.10	24.4	128	70	217	47	170.00	6.00	1	1.00	1	1	1	0	1	1	1	1	0	1	1
0	0	71	2	146	42.6	1.70	19.9	112	61	236	49	187.00	5.60	1	1.00	1	0	1	0	0	1	0	1	1	0	1
0	0	70	2	147	45.8	.20	21.1	140	57	198	47	151.00	6.40	1	1.00	1	0	1	1	1	0	1	1	0	1	0
1	1	65	1	160	63.8	3.00	24.9	143	95	182	65	117.00	5.80	0	2.00	0	1	1	0	1	0	0	1	0	1	1
0	0	80	1	159	63.5	5.60	25.0	128	76	206	52	154.00	5.30	1	1.00	1	0	1	0	1	1	1	0	1	1	1
1	1	73	1	164	66.9	2.10	24.9	138	72	205	54	151.00	5.80	0	1.00	0	0	1	0	1	0	1	1	0	1	1
1	1	41	2	159	64.2	-3.30	25.4	118	67	236	62	174.00	5.40	1	1.00	1	1	1	0	0	1	1	0	1	0	1
1	1	64	2	151	39.7	1.00	17.4	126	74	198	78	120.00	5.80	0	1.00	1	1	1	0	0	0	0	1	1	1	1
0	0	61	1	171	69.7	-.10	23.9	110	64	197	44	153.00	5.30	1	1.00	0	0	1	1	1	1	1	1	1	1	1
1	1	58	2	157	59.7	2.20	24.3	131	68	216	47	169.00	5.30	0	1.00	1	1	1	1	1	1	1	1	1	1	1
0	0	74	2	146	58.9	2.10	27.5	115	67	235	50	185.00	5.30	1	1.00	1	0	1	0	1	0	1	0	0	0	1
0	0	80	2	140	39.0	.60	19.8	172	85	200	67	133.00	6.00	1	1.00	1	0	1	0	1	1	1	1	0	1	1
1	1	74	2	152	53.0	-2.50	22.9	156	84	221	56	165.00	5.20	1	1.00	1	0	1	1	1	0	0	1	0	0	1
1	1	49	2	148	54.9	-3.50	25.1	153	92	295	70	225.00	5.60	1	1.00	1	0	1	1	1	1	1	0	0	0	1
0	0	63	2	153	72.9	-1.20	31.1	134	75	151	58	93.00	5.90	1	1.00	1	0	1	0	1	1	0	0	0	1	1
0	0	69	1	161	58.6	-.20	22.5	159	89	206	54	152.00	5.60	0	1.00	0	0	1	0	1	0	1	1	0	1	1
0	0	80	2	148	53.1	3.10	24.4	142	87	200	57	143.00	5.50	1	1.00	1	0	1	0	0	0	0	1	0	1	1

1	1	75	1	159	61.9	-1.40	24.3	117	63	179	52	127.00	5.60	1	1.00	1	1	1	0	1	1	1	1	1	1	1
1	1	46	1	166	65.5	-1.40	23.7	103	67	140	54	86.00	4.60	0	1.00	1	0	1	1	1	1	1	1	1	1	1
0	0	63	1	163	67.6	-3.10	25.5	133	99	221	34	187.00	5.50	1	1.00	1	0	1	0	1	1	1	0	0	0	1
0	0	60	2	159	56.0	.50	22.2	111	71	219	59	160.00	5.90	1	1.00	0	0	1	0	1	0	1	1	1	1	1
0	0	72	2	136	50.3	3.70	27.2	170	86	222	59	163.00	5.40	1	1.00	1	1	1	1	1	1	1	0	0	0	1
0	0	64	1	161	65.0	-1.30	25.2	165	88	195	40	155.00	5.20	1	1.00	1	0	1	1	1	1	1	0	0	1	1
0	0	90	2	132	37.4	-.40	21.5	137	79	117	43	74.00	5.40	1	1.00	1	0	1	1	1	0	1	1	1	1	1
0	0	46	1	162	56.4	2.90	21.4	94	52	188	50	138.00	5.90	0	1.00	1	0	1	0	1	0	0	1	1	1	1
1	1	75	1	168	55.3	-.10	19.5	92	54	205	81	124.00	6.00	1	1.00	1	1	1	0	1	0	1	1	1	1	0
1	1	64	2	154	55.8	1.00	23.4	121	78	161	34	127.00	5.90	1	1.00	1	0	1	1	0	0	0	1	1	0	1
0	0	57	2	159	56.7	.80	22.3	120	71	202	92	110.00	5.90	1	1.00	0	1	1	0	1	0	0	1	1	1	1
1	1	77	1	158	63.9	-.20	25.4	139	68	189	59	130.00	6.10	1	1.00	0	0	1	1	1	1	1	0	1	1	1
1	1	73	2	160	57.8	-.10	22.6	145	65	205	56	149.00	5.40	1	1.00	1	0	1	1	1	1	1	1	0	1	1
0	0	81	2	152	63.6	1.50	27.4	133	78	155	42	113.00	6.00	1	1.00	1	0	1	1	1	0	0	0	0	0	0
1	1	69	2	150	42.0	.70	18.5	133	69	231	77	154.00	5.20	1	1.00	1	1	1	1	1	0	0	1	1	0	1
0	0	69	1	165	64.3	-2.90	23.6	146	80	202	24	178.00	5.80	1	1.00	1	0	1	0	1	1	1	1	0	0	1
1	1	47	2	154	50.1	3.10	21.1	112	68	167	65	102.00	5.20	1	1.00	1	0	1	1	1	0	1	1	1	1	1
0	0	75	1	163	61.0	-3.70	23.0	122	75	197	52	145.00	5.30	0	1.00	0	0	1	0	1	0	0	1	0	1	1
1	1	88	2	135	40.4	.00	22.0	136	65	170	46	124.00	6.00	1	1.00	1	0	1	1	1	1	1	1	0	1	1
1	1	41	1	161	66.3	1.30	25.5	98	64	220	43	177.00	5.40	1	1.00	1	1	1	0	0	1	1	0	1	0	1
1	1	58	1	166	75.6	1.60	27.3	144	82	204	95	109.00	5.70	1	1.00	0	1	1	1	1	1	1	0	0	1	1
1	1	56	1	162	69.1	1.60	26.2	144	87	210	49	161.00	5.50	1	1.00	0	1	1	1	1	1	1	0	0	1	1
1	1	33	2	162	84.0	-1.50	31.8	117	72	178	39	139.00	5.20	1	1.00	0	1	1	1	0	0	1	0	1	0	1
1	1	41	2	152	44.3	-1.00	19.0	108	60	194	74	120.00	5.50	1	1.00	1	0	1	0	0	1	1	1	1	1	1
1	1	67	2	151	41.4	.60	18.2	110	62	190	89	101.00	5.40	1	1.00	1	1	1	1	0	0	0	1	1	1	1
1	1	57	2	153	51.6	1.60	21.9	133	78	232	82	150.00	6.10	1	1.00	1	1	1	0	1	1	1	1	0	0	1
1	1	49	1	155	64.3	-8.40	26.6	128	92	219	59	160.00	5.50	0	1.00	1	0	0	0	1	0	1	0	0	1	1
1	1	74	2	146	49.5	-1.00	23.1	137	80	192	79	113.00	6.10	1	1.00	1	0	1	1	1	1	0	1	1	1	1
1	1	64	1	168	58.8	-.90	20.7	136	84	151	65	86.00	4.80	1	1.00	0	0	1	0	1	0	0	1	1	1	1
0	0	58	2	152	42.1	-2.10	18.1	126	70	245	59	186.00	4.70	1	1.00	1	0	1	0	0	1	0	1	1	0	1
0	0	46	1	171	78.4	3.10	26.8	120	78	223	58	165.00	5.30	1	1.00	0	1	1	0	1	0	1	0	1	0	1
1	1	64	1	166	65.5	1.10	23.8	159	89	164	60	104.00	5.30	1	1.00	1	0	1	0	1	1	1	1	0	1	1
1	1	52	2	153	61.5	-2.50	26.2	169	106	280	55	225.00	5.20	1	1.00	1	0	1	0	1	0	0	0	0	0	1
1	1	65	1	160	75.0	-.70	29.4	144	88	234	60	174.00	5.30	0	1.00	1	0	1	0	1	1	1	0	0	0	1
0	0	72	2	141	47.3	-.70	23.9	123	64	227	96	131.00	5.40	1	1.00	1	1	1	0	1	0	0	1	1	0	1
0	0	44	2	151	46.0	.10	20.1	104	63	187	76	111.00	5.40	1	1.00	1	0	1	0	0	0	1	1	1	1	1
0	0	74	2	145	47.8	-.90	22.6	144	73	211	71	140.00	5.30	1	1.00	1	1	1	0	0	0	0	1	0	1	1
1	1	79	1	157	65.1	3.40	26.4	126	79	177	48	129.00	5.50	1	1.00	0	0	1	0	0	1	1	0	1	1	1
0	1	48	2	148	47.4	-2.40	21.6	130	82	264	91	173.00	5.10	1	1.00	1	0	1	1	1	1	1	1	1	0	1

1	1	61	1	173	63.7	.70	21.2	121	71	199	45	154.00	4.90	1	1.00	0	0	1	0	1	1	1	1	1	1	1
1	1	73	2	152	69.6	.90	30.0	134	86	160	71	89.00	5.40	1	1.00	1	0	1	1	1	1	1	0	1	1	1
0	0	58	1	167	52.9	.30	18.9	118	65	227	115	112.00	6.10	1	1.00	0	1	1	0	1	1	1	1	1	0	1
0	0	66	2	152	54.3	-.10	23.5	116	65	147	53	94.00	5.90	1	1.00	1	0	1	1	0	1	1	1	0	0	1
1	1	42	2	161	58.1	1.70	22.3	109	61	172	77	95.00	4.40	1	1.00	1	0	1	0	1	0	0	1	1	1	1
1	1	47	2	160	48.8	-.80	19.1	119	81	239	89	150.00	5.50	1	1.00	1	1	1	0	1	0	0	1	1	0	1
1	1	33	2	160	47.9	-1.20	18.7	110	59	160	76	84.00	5.70	1	1.00	1	1	1	0	1	0	1	1	1	1	1
1	1	74	1	175	57.8	-1.50	18.8	143	81	155	47	108.00	5.60	1	1.00	1	1	1	0	1	0	0	1	0	1	1
1	1	67	2	154	63.6	-3.00	26.8	125	76	137	55	82.00	5.60	1	1.00	1	1	1	0	0	0	0	0	1	1	1
0	0	65	2	148	51.6	1.00	23.6	134	88	166	52	114.00	5.90	1	1.00	1	0	1	1	1	0	0	1	0	1	1
0	0	65	2	157	62.5	-1.10	25.3	129	84	192	57	135.00	6.00	1	1.00	1	1	1	1	1	1	0	0	1	1	1
0	0	46	2	159	41.1	-.70	16.3	97	62	276	64	212.00	5.80	0	1.00	1	1	1	1	1	1	1	1	1	0	1
0	0	63	2	156	63.7	-.40	26.1	121	76	216	40	176.00	7.00	1	1.00	1	0	1	1	0	1	1	0	0	0	0
0	0	62	1	169	68.1	-.80	23.9	144	89	186	62	124.00	5.30	1	1.00	0	0	1	0	0	0	0	1	0	1	1
1	1	58	2	159	64.8	1.30	25.6	138	82	205	51	154.00	5.60	1	1.00	1	0	1	1	1	1	1	0	1	1	1
1	1	68	2	144	50.8	-1.40	24.5	132	74	189	93	96.00	5.10	1	1.00	1	1	1	0	1	0	0	1	0	1	1
1	1	75	1	165	68.4	-.80	25.1	122	79	206	70	136.00	5.60	1	1.00	0	0	1	1	0	1	1	0	1	1	1
1	1	72	2	144	55.8	-4.20	26.9	146	80	185	73	112.00	5.80	1	1.00	1	0	1	0	1	0	0	0	0	1	1
0	1	80	1	161	53.7	-.90	20.6	131	74	217	95	122.00	5.50	0	1.00	0	0	1	1	0	1	1	1	1	1	1
0	0	46	1	171	69.4	-2.60	23.6	120	87	227	71	156.00	5.40	0	1.00	0	0	1	1	1	1	1	1	1	0	1
1	1	49	1	179	68.3	-.40	21.2	119	73	176	64	112.00	5.50	0	1.00	1	1	1	0	0	1	1	1	1	1	1
0	0	79	1	162	63.0	2.10	23.9	105	56	165	96	69.00	5.00	0	1.00	1	1	1	0	1	1	1	1	1	1	1
0	0	73	2	148	49.4	2.10	22.4	120	68	247	45	202.00	5.50	1	1.00	1	0	1	0	1	0	0	1	0	0	1
1	1	62	1	166	54.3	-.90	19.6	117	79	249	76	173.00	5.50	1	1.00	1	1	1	0	1	1	1	1	1	0	1
0	0	70	2	144	56.0	.70	27.1	125	77	179	64	115.00	5.80	1	1.00	1	1	1	0	1	1	1	0	1	1	1
1	1	63	2	156	62.5	.40	25.6	115	67	204	69	135.00	5.10	1	1.00	1	0	1	0	0	0	1	0	0	1	1
1	1	71	2	143	44.6	-.40	21.6	127	78	162	45	117.00	5.20	1	1.00	1	0	1	0	1	0	0	1	1	1	1
1	1	68	1	159	60.1	-2.90	23.6	132	80	226	39	187.00	7.50	0	1.00	0	1	1	1	1	1	1	1	1	0	0
1	1	76	1	164	59.1	1.00	21.9	134	86	205	83	122.00	5.00	1	1.00	0	1	1	0	1	1	1	1	1	1	1
1	1	87	1	154	52.1	-1.70	22.0	125	72	161	67	94.00	5.50	1	1.00	0	1	1	1	1	1	1	1	1	1	1
1	1	46	2	152	51.8	2.20	22.3	123	72	215	86	129.00	5.40	1	1.00	0	0	1	0	1	0	1	1	1	1	1
1	1	80	2	149	54.1	.50	24.3	136	69	223	57	166.00	4.90	1	1.00	1	0	1	1	1	0	1	1	0	0	1
0	0	74	2	146	57.6	-1.50	27.1	127	76	166	35	131.00	6.00	1	1.00	1	1	1	0	1	0	0	0	1	0	1
1	1	51	2	166	46.7	2.90	16.9	130	82	209	68	141.00	5.40	1	1.00	1	1	1	1	1	0	0	1	1	0	1
1	1	37	2	165	54.6	2.20	20.1	100	54	201	62	139.00	5.20	0	1.00	1	0	1	0	0	0	1	1	1	1	1
1	1	70	2	153	57.0	3.20	24.4	111	52	195	61	134.00	5.50	1	1.00	1	1	1	1	1	1	1	1	1	1	1
1	1	52	2	155	56.6	-2.80	23.6	136	84	335	74	261.00	5.40	1	1.00	1	1	1	0	1	1	1	1	1	0	1
1	1	33	2	155	43.5	-.40	18.1	99	61	178	76	102.00	5.20	1	1.00	1	0	1	1	1	1	1	1	1	1	1
1	1	33	2	156	43.4	-.50	17.8	97	61	234	87	147.00	5.20	1	1.00	1	0	1	1	1	1	1	1	1	0	1

1	1	49	1	169	67.2	-2.90	23.5	121	80	208	44	164.00	6.50	1	1.00	0	1	1	0	1	1	1	1	0	1	0
0	0	41	1	170	54.6	-.10	18.8	104	60	156	82	74.00	5.50	0	1.00	1	1	1	0	1	0	0	1	1	1	1
1	1	64	2	146	46.9	-.60	22.0	124	74	248	50	198.00	5.20	1	1.00	1	0	1	1	1	1	0	1	1	0	1
1	1	76	1	178	74.2	-.50	23.4	146	85	153	56	97.00	6.10	1	1.00	0	0	1	1	1	1	1	1	0	1	1
1	1	25	1	164	98.8	-1.40	36.8	131	76	163	41	122.00	5.60	0	1.00	1	1	1	1	1	0	0	0	1	1	1
1	1	63	2	149	52.6	-1.70	23.8	118	78	210	65	145.00	5.70	1	1.00	1	0	1	1	1	0	0	1	1	1	1
1	1	64	2	162	46.1	-.30	17.6	121	79	189	69	120.00	5.50	1	1.00	1	0	1	1	1	0	1	1	1	1	1
1	1	27	2	151	42.5	-4.60	18.6	124	78	181	71	110.00	5.30	1	1.00	1	0	1	0	1	1	1	1	1	1	1
1	1	44	1	184	80.9	-.60	23.9	116	74	258	57	201.00	5.20	1	1.00	1	0	1	0	1	0	0	1	1	0	1
0	0	50	2	144	47.9	-2.50	23.0	147	81	259	54	205.00	4.90	1	1.00	0	0	1	0	0	1	1	1	0	0	1
1	1	72	2	150	53.2	.40	23.7	135	71	230	72	158.00	5.90	1	1.00	1	0	1	0	1	1	0	1	0	0	1
1	1	70	2	155	50.9	-2.90	21.2	122	63	195	65	130.00	5.40	1	1.00	1	0	1	1	1	0	0	1	1	1	1
1	1	76	1	157	64.5	-4.20	26.3	110	67	216	47	169.00	5.50	1	1.00	1	0	1	1	1	1	0	0	1	1	1
0	0	65	2	155	58.4	.90	24.4	136	83	195	43	152.00	5.60	1	1.00	1	1	1	0	1	1	1	1	1	1	1
1	1	72	2	152	62.3	-2.00	27.1	115	72	147	46	101.00	5.80	1	1.00	1	0	1	0	1	1	0	0	1	1	1
1	1	50	2	162	75.5	-2.50	28.6	149	93	193	74	119.00	5.80	1	1.00	1	0	1	1	0	0	0	0	0	1	1
0	0	22	1	172	87.0	5.80	29.5	143	86	192	66	126.00	5.00	1	1.00	1	0	1	0	1	1	1	0	0	1	1
0	0	72	2	162	52.9	.30	20.2	142	85	212	82	130.00	5.30	1	1.00	1	1	1	1	1	1	1	1	0	1	1
0	0	44	1	165	71.4	-.90	26.3	111	64	262	43	219.00	5.90	0	1.00	1	1	1	0	1	0	0	0	1	0	1
1	1	42	2	163	62.3	2.00	23.3	122	71	213	93	120.00	5.40	1	1.00	0	0	1	0	1	1	1	1	1	1	1
0	0	70	2	159	57.2	2.20	22.7	120	65	162	47	115.00	5.40	1	1.00	1	0	1	0	0	0	0	1	1	1	1
0	0	75	1	173	69.7	-3.40	23.2	115	69	134	67	67.00	5.00	1	1.00	1	1	1	1	1	1	1	1	1	1	1
1	1	77	2	147	51.7	-1.60	23.9	115	72	177	45	132.00	5.90	1	1.00	1	0	1	1	1	1	0	1	1	1	1
0	1	82	2	148	48.1	-1.30	21.9	135	74	196	47	149.00	5.50	1	1.00	0	0	1	0	1	0	0	1	0	0	1
0	1	61	2	158	48.9	-1.50	19.6	120	71	229	57	172.00	5.30	1	1.00	0	0	1	0	1	1	1	1	1	0	1
0	0	81	2	159	66.0	-2.10	26.2	141	77	201	70	131.00	5.90	1	1.00	1	0	1	1	1	1	1	0	0	0	1
0	0	64	2	153	58.7	-.30	25.0	160	86	307	50	257.00	6.50	1	1.00	1	0	1	0	1	0	0	0	0	0	0
1	1	72	2	162	56.3	-1.80	21.4	141	77	209	68	141.00	5.90	1	1.00	1	1	1	1	1	0	1	1	0	1	1
1	1	26	1	178	117.8	-5.30	37.0	121	64	174	57	117.00	6.40	1	1.00	1	1	1	0	1	0	1	0	1	1	1
1	1	72	2	156	60.2	-2.70	24.8	142	75	208	43	165.00	5.70	1	1.00	1	0	1	0	1	0	0	1	0	1	1
0	0	29	2	156	76.2	-3.40	31.1	116	74	231	83	148.00	4.90	1	1.00	1	0	1	1	1	1	1	0	1	0	1
1	1	59	2	148	77.1	-3.50	35.0	131	66	179	62	117.00	6.30	1	1.00	1	1	1	1	1	1	1	0	1	0	0
1	1	65	2	153	56.6	.60	24.2	117	78	208	75	133.00	5.40	1	1.00	0	0	1	1	1	0	0	1	1	1	1
0	0	77	2	141	46.0	-1.40	23.0	135	70	190	68	122.00	5.70	1	1.00	1	1	1	1	1	1	1	1	0	1	1
1	1	73	1	155	69.2	-.30	28.6	119	71	225	70	155.00	5.10	1	1.00	0	0	1	1	1	1	1	0	0	0	1
1	1	72	1	160	67.5	-2.40	26.4	156	89	193	68	125.00	6.00	1	1.00	0	0	1	1	0	1	1	0	0	1	0
1	1	39	2	147	45.6	-.80	21.1	116	66	168	56	112.00	5.60	1	1.00	0	0	1	0	1	1	1	1	1	1	1
1	1	85	2	144	58.6	.00	28.2	132	75	197	59	138.00	6.50	1	1.00	1	0	1	1	1	1	1	0	0	0	0
0	0	63	1	160	53.9	1.80	21.0	130	80	257	55	202.00	5.90	1	1.00	0	1	1	1	0	1	0	1	1	0	1

0	0	78	2	146	49.4	1.60	23.2	114	62	240	61	179.00	5.00	1	1.00	1	1	1	0	0	1	1	1	1	0	1
1	1	66	2	152	57.0	-.20	24.6	129	72	193	66	127.00	5.70	1	1.00	1	0	1	1	1	1	1	1	1	0	1
0	0	68	1	161	68.9	1.30	26.5	133	73	147	54	93.00	5.20	1	1.00	0	1	1	1	1	1	1	0	1	1	1
1	1	62	2	152	60.8	1.10	26.3	116	82	167	54	113.00	5.50	1	1.00	1	0	1	0	1	1	1	0	0	0	1
0	0	63	2	156	67.5	-.50	27.6	149	84	237	64	173.00	6.10	1	1.00	1	0	1	1	1	0	0	0	0	0	1
1	1	25	1	167	58.4	-2.30	21.0	102	55	178	67	111.00	5.20	1	1.00	0	0	1	1	1	0	1	1	1	1	1
1	1	76	1	161	59.5	-2.60	23.0	156	82	185	53	132.00	6.90	1	1.00	1	0	1	0	0	0	0	1	0	1	0
0	0	57	2	155	45.7	1.40	18.9	153	92	237	98	139.00	5.90	1	1.00	1	0	1	0	1	0	1	1	0	0	1
1	1	71	1	165	63.0	.90	23.1	133	64	157	50	107.00	6.10	1	1.00	1	0	1	0	1	0	1	1	0	1	0
1	1	66	2	145	53.8	-1.00	25.5	153	78	142	62	80.00	5.90	1	1.00	0	0	1	0	1	0	0	0	0	0	1
1	1	44	1	172	83.8	-4.80	28.4	127	75	203	50	153.00	7.90	1	1.00	1	0	1	1	1	1	1	0	1	0	0
1	1	57	2	162	71.4	3.10	27.3	147	82	148	69	79.00	5.80	1	1.00	1	1	1	1	1	0	1	0	0	0	1
0	0	74	2	150	42.1	-1.30	18.7	129	83	178	55	123.00	5.30	1	1.00	1	1	1	0	1	1	0	1	0	1	1
1	1	44	2	165	60.1	.80	22.0	119	65	169	80	89.00	5.10	1	1.00	1	0	1	0	1	0	1	1	1	1	1
1	1	66	2	151	48.4	.30	21.2	141	86	217	58	159.00	5.40	1	1.00	1	1	1	1	1	0	1	1	0	0	1
0	0	69	2	147	48.2	9.60	22.2	144	77	212	77	135.00	5.80	1	1.00	1	0	1	0	1	0	0	1	0	1	1
0	0	52	2	156	43.1	1.20	17.6	112	68	252	122	130.00	6.00	1	1.00	0	1	1	0	1	0	1	1	1	0	1
0	0	52	1	177	68.7	-1.30	22.0	114	72	175	47	128.00	5.90	0	1.00	1	1	1	1	0	1	1	1	1	1	1
1	1	79	2	139	73.3	-6.40	37.7	174	85	197	41	156.00	7.10	1	1.00	1	1	1	1	1	1	1	0	0	1	0
1	1	69	1	159	58.0	.00	22.8	166	95	175	58	117.00	5.30	1	1.00	0	0	1	0	1	1	1	1	0	1	1
0	0	61	2	166	54.9	.00	20.0	127	90	210	98	112.00	5.60	1	1.00	1	0	1	1	1	1	1	1	0	1	1
1	1	69	1	163	57.7	-2.30	21.6	130	80	153	48	105.00	5.80	1	1.00	0	1	1	0	1	0	1	1	1	1	1
0	0	71	2	151	48.7	-.40	21.4	142	72	178	73	105.00	5.80	1	1.00	1	0	1	0	1	0	1	1	0	0	1
0	0	73	2	151	51.5	-.70	22.4	108	56	224	63	161.00	5.40	1	1.00	1	0	1	1	1	1	0	1	1	0	1
0	0	79	1	154	63.1	.30	26.7	115	66	143	32	111.00	5.80	1	1.00	0	0	1	0	1	1	1	0	0	0	1
0	0	70	2	142	43.8	.90	21.6	96	64	178	78	100.00	5.70	1	1.00	1	0	1	1	1	0	0	1	1	1	1
0	0	49	2	153	55.6	-4.00	23.7	158	102	169	65	104.00	5.00	1	1.00	1	0	1	0	0	1	1	1	0	1	1
1	1	73	2	142	47.5	.10	23.4	143	77	216	65	151.00	5.90	1	1.00	1	0	1	0	1	1	1	1	0	1	1
1	1	35	2	170	68.2	-.10	23.5	106	57	241	52	189.00	5.50	1	1.00	1	1	0	0	1	0	0	1	1	0	1
0	0	73	1	164	61.8	-2.40	22.9	97	57	211	60	151.00	5.40	1	1.00	0	0	1	1	1	1	1	1	0	1	1
0	0	47	1	171	74.3	-3.80	25.4	116	73	201	95	106.00	5.40	1	1.00	0	1	1	0	1	1	1	0	1	1	1
0	0	71	2	154	74.0	-1.80	31.1	126	73	217	54	163.00	6.50	1	1.00	1	0	1	0	1	0	0	0	1	0	0
0	0	61	2	156	42.0	1.10	17.2	101	67	158	71	87.00	5.30	1	1.00	1	0	1	0	1	0	1	1	1	1	1
1	1	65	2	156	50.3	-2.80	20.7	132	83	222	44	178.00	6.00	1	1.00	1	0	1	1	1	1	1	1	0	0	1
0	0	42	1	175	76.9	-2.90	25.1	107	63	214	55	159.00	5.50	1	1.00	0	0	1	0	0	1	1	0	1	1	1
0	0	64	2	159	61.6	.80	24.3	107	62	254	65	189.00	5.90	0	1.00	0	0	1	1	1	0	1	1	0	0	1
1	1	38	2	160	53.4	2.10	20.7	98	60	191	60	131.00	5.10	0	1.00	1	1	1	1	1	1	1	1	1	1	1
1	1	75	1	157	60.3	-1.20	24.4	162	83	190	75	115.00	5.20	0	1.00	1	1	1	0	1	1	1	1	0	1	1
1	1	72	1	154	58.2	-1.10	24.4	128	71	251	78	173.00	5.40	1	1.00	0	0	1	0	0	0	1	1	1	0	1

0	0	34	1	178	57.0	-1.10	17.9	131	85	265	85	180.00	5.40	1	1.00	1	1	1	0	1	1	1	1	1	0	1
0	0	43	2	161	48.4	-1.80	18.5	111	76	197	86	111.00	5.50	0	1.00	1	0	1	0	1	0	0	1	1	1	1
1	1	56	2	160	55.2	-1.10	21.6	128	77	213	77	136.00	5.10	1	1.00	1	1	1	1	1	0	1	1	1	1	1
1	1	67	2	152	50.3	.10	21.8	163	89	235	97	138.00	5.70	1	1.00	1	0	1	1	1	1	1	1	0	0	1
0	0	73	1	164	63.7	1.80	23.8	109	64	168	46	122.00	6.20	1	1.00	0	1	1	0	1	1	1	1	0	1	1
1	1	61	2	155	49.7	1.00	20.6	139	77	231	66	165.00	5.60	1	1.00	1	1	1	1	1	1	1	1	1	0	1
1	1	41	2	157	44.5	-.80	18.1	127	87	229	98	131.00	5.50	0	1.00	0	0	1	1	1	1	1	1	1	0	1
1	1	54	2	154	50.8	-1.60	21.3	103	63	251	40	211.00	5.50	1	1.00	1	0	1	0	0	0	0	1	1	0	1
1	1	77	1	156	50.5	-.70	20.7	164	81	172	77	95.00	5.40	1	1.00	0	0	1	1	1	0	1	1	0	1	1
1	1	72	1	156	58.6	2.40	24.1	138	75	175	63	112.00	5.70	1	1.00	0	0	1	1	1	0	0	1	1	1	0
1	1	58	2	151	45.7	-3.10	19.9	161	109	320	138	182.00	5.40	1	1.00	1	0	1	1	1	1	1	1	0	0	1
1	1	48	2	153	39.1	.00	16.6	137	84	219	65	154.00	5.40	1	1.00	0	0	1	0	1	1	1	1	1	1	1
0	0	61	2	155	64.4	-.10	26.8	109	68	244	60	184.00	6.40	1	1.00	1	0	1	0	0	1	0	0	1	0	1
0	0	58	2	158	58.9	1.90	23.5	109	74	264	57	207.00	5.00	1	1.00	1	0	1	1	1	0	0	1	0	0	1
0	0	66	2	148	49.0	-.30	22.4	105	66	223	61	162.00	5.20	1	1.00	1	1	1	0	1	0	1	1	0	0	1
1	1	77	1	164	54.5	-1.40	20.2	148	77	180	56	124.00	5.50	1	1.00	0	1	1	1	1	1	1	1	0	1	1
1	1	51	1	170	66.3	.90	22.9	100	63	260	50	210.00	5.50	0	1.00	1	0	1	1	1	1	1	1	1	0	1
1	1	49	2	154	50.9	.40	21.4	101	67	244	58	186.00	5.70	0	1.00	0	0	1	0	1	1	1	1	1	0	1
0	0	62	2	150	59.2	1.70	26.4	125	74	223	86	137.00	5.80	1	1.00	1	0	1	1	1	1	1	0	1	0	1
1	1	64	2	152	46.9	1.40	20.4	130	83	212	61	151.00	5.30	1	1.00	1	0	1	0	1	0	1	1	1	1	1
0	0	70	1	165	57.3	-.10	21.1	138	78	181	70	111.00	5.30	1	1.00	0	0	1	1	1	1	1	1	0	1	1
0	0	77	2	145	51.2	.70	24.4	159	84	219	64	155.00	5.10	1	1.00	1	0	1	0	1	0	1	1	0	1	1
0	0	64	2	153	52.6	5.40	22.4	123	77	246	86	160.00	5.50	1	1.00	1	1	1	1	1	1	0	1	1	0	1
1	1	41	1	160	60.4	.50	23.5	117	83	204	64	140.00	6.80	0	1.00	1	0	1	1	1	0	0	1	1	1	0
0	0	40	2	162	45.9	1.30	17.5	106	69	171	44	127.00	5.40	1	1.00	1	1	1	1	1	1	1	1	1	1	1
1	1	64	2	165	63.7	-2.30	23.2	112	62	204	59	145.00	5.20	1	1.00	1	0	1	1	1	0	1	1	1	1	1
1	1	70	1	162	59.3	2.10	22.4	145	76	150	34	116.00	7.00	1	1.00	1	1	1	1	1	1	1	1	0	0	0
1	1	70	1	167	62.5	-2.80	22.3	151	87	170	88	82.00	5.90	1	1.00	0	1	1	1	1	1	1	1	0	1	1
1	1	69	2	150	52.4	.20	23.3	114	60	190	65	125.00	5.60	1	1.00	1	1	1	1	1	0	0	1	1	1	1
0	0	67	1	166	64.6	2.00	23.5	114	70	198	62	136.00	5.40	1	1.00	1	0	1	0	0	0	0	1	1	1	1
0	0	62	2	152	58.9	-1.20	25.4	129	84	251	69	182.00	6.00	1	1.00	0	0	1	0	0	1	1	0	1	0	1
1	1	69	2	144	38.8	1.90	18.6	141	78	260	97	163.00	5.40	0	2.00	1	0	1	0	1	0	1	1	0	0	1
1	1	69	2	151	42.2	-1.60	18.5	134	78	234	57	177.00	5.80	1	1.00	1	0	1	0	1	0	1	1	0	0	1
1	1	69	1	166	53.8	1.50	19.4	93	58	161	51	110.00	5.50	1	1.00	1	0	1	0	1	0	1	1	1	1	1
1	1	55	1	155	52.8	-2.60	22.0	137	78	206	89	117.00	6.50	1	1.00	0	1	1	1	1	1	1	1	1	1	0
1	1	78	2	147	53.7	.10	25.0	122	67	182	56	126.00	5.40	1	1.00	1	0	1	1	1	1	1	0	1	1	1
0	0	65	2	148	43.6	.90	19.8	145	93	250	69	181.00	5.40	1	1.00	1	0	1	1	1	0	1	1	0	0	1
0	0	90	2	146	52.1	-1.10	24.5	156	81	194	53	141.00	5.80	1	1.00	1	0	1	1	1	1	0	1	0	1	1
0	0	68	2	151	48.9	1.60	21.5	132	71	261	62	199.00	6.00	1	1.00	1	0	1	0	1	0	0	1	0	0	0

1	1	57	2	150	48.2	1.20	21.4	114	68	262	60	202.00	4.80	1	1.00	1	1	1	1	1	1	1	1	1	0	1
1	1	71	1	148	48.9	2.10	22.4	133	73	165	70	95.00	5.60	1	1.00	1	0	1	1	1	0	1	1	1	1	1
1	1	59	1	155	59.7	1.10	24.7	144	86	213	58	155.00	11.00	1	1.00	0	1	1	1	1	1	1	1	0	1	0
0	0	44	2	149	46.5	.80	20.9	101	54	217	65	152.00	5.30	1	1.00	1	1	1	0	1	0	0	1	1	1	1
0	0	61	2	154	53.0	-1.30	22.3	130	75	233	105	128.00	5.70	1	1.00	0	0	1	1	1	1	0	1	1	0	1
1	1	61	2	147	55.5	1.70	25.6	153	85	279	52	227.00	5.90	1	1.00	1	0	1	0	1	1	1	0	0	0	1
1	1	40	2	151	57.4	1.50	25.3	105	74	218	64	154.00	5.70	1	1.00	1	0	1	1	1	1	1	0	1	1	1
0	0	76	1	163	55.3	-1.40	20.9	99	70	174	54	120.00	5.60	1	1.00	1	0	1	1	1	1	1	1	0	1	1
1	1	69	2	140	49.5	-1.90	25.1	129	74	220	63	157.00	5.80	1	1.00	1	1	1	0	1	0	0	0	1	0	1
1	1	53	2	161	69.7	1.40	26.8	159	93	219	58	161.00	5.40	1	1.00	1	1	1	1	1	1	1	0	0	1	1
0	0	50	1	174	63.4	1.10	21.0	129	84	206	77	129.00	5.80	1	1.00	1	0	1	1	1	1	1	1	1	1	1
1	1	67	2	147	53.1	1.30	24.5	109	63	227	89	138.00	5.50	1	1.00	1	1	1	0	1	0	1	1	1	0	1
1	1	81	2	153	50.0	-1.40	21.3	144	78	256	53	203.00	6.40	1	1.00	1	0	1	1	1	1	1	1	0	0	1
0	0	74	1	150	64.6	-1.70	28.5	135	86	188	31	157.00	5.60	1	1.00	0	1	1	1	1	1	1	0	0	0	1
1	1	63	1	159	59.5	1.30	23.5	161	92	210	79	131.00	5.30	1	1.00	0	0	1	0	1	1	1	1	0	1	1
1	1	69	1	159	59.1	-.30	23.5	155	76	224	79	145.00	5.90	1	1.00	0	1	1	1	1	0	1	1	0	0	1
0	1	69	2	152	49.9	.20	21.6	116	74	191	51	140.00	5.90	1	1.00	1	0	1	1	1	1	1	1	1	1	1
1	1	67	2	155	50.2	-1.60	21.0	108	68	229	75	154.00	5.80	1	1.00	1	0	1	0	1	0	0	1	1	0	1
1	1	60	2	151	48.9	1.70	21.3	157	84	270	73	197.00	5.10	1	1.00	1	1	1	0	1	0	0	1	0	0	1
1	1	73	2	154	50.2	.70	21.3	155	85	200	59	141.00	5.70	1	1.00	1	0	1	1	1	1	1	1	0	1	1
1	1	72	2	138	49.4	-.70	25.9	151	80	227	68	159.00	5.70	1	1.00	1	0	1	1	1	1	1	0	0	0	1
1	1	51	1	164	59.5	-.50	22.2	128	76	183	68	115.00	5.10	1	1.00	0	0	1	0	0	0	0	1	0	1	1
1	1	82	2	154	61.6	2.40	25.8	147	87	238	89	149.00	6.00	1	1.00	1	0	1	0	1	0	0	0	0	0	1
1	1	64	1	166	44.2	1.80	16.1	99	68	205	46	159.00	5.70	0	1.00	1	1	1	1	0	1	1	1	1	1	0
1	1	62	2	152	55.6	-.50	23.9	116	75	283	56	227.00	6.60	1	1.00	1	0	1	1	1	1	0	1	1	0	0
1	1	71	2	150	55.2	-3.10	24.6	108	70	198	51	147.00	5.30	1	1.00	1	0	1	1	1	0	1	1	1	1	1
1	1	63	1	161	44.6	1.50	17.1	134	82	217	83	134.00	5.80	1	1.00	0	1	1	1	1	1	1	1	1	1	1
1	1	54	2	147	47.7	3.00	22.0	123	76	256	48	208.00	5.50	1	1.00	0	0	0	0	0	0	0	1	1	0	1
1	1	64	2	155	60.5	2.00	25.1	125	80	290	121	169.00	5.80	1	1.00	1	0	1	0	1	1	0	0	1	0	1
1	1	70	2	144	49.8	.80	23.9	114	63	287	42	245.00	5.80	1	1.00	1	0	1	1	1	1	1	1	1	0	1
1	1	54	1	169	78.3	-1.60	27.5	117	81	233	46	187.00	6.00	1	1.00	1	0	1	0	0	1	1	0	1	0	1
1	1	65	1	173	55.3	-1.00	18.3	114	75	178	46	132.00	5.50	1	1.00	1	0	1	0	1	1	1	1	0	1	1
1	1	60	2	155	44.9	-1.50	18.7	92	53	160	63	97.00	5.50	1	1.00	1	0	1	1	0	0	0	1	1	1	1
1	1	74	2	147	50.5	.90	23.4	149	80	166	66	100.00	5.30	1	1.00	1	1	1	1	1	1	1	1	0	1	1
1	1	74	2	143	50.9	-2.70	25.0	117	63	227	64	163.00	7.50	1	1.00	1	1	1	1	1	1	1	0	1	0	0
1	1	63	1	161	61.3	1.00	23.7	121	73	153	40	113.00	5.10	1	1.00	1	0	1	0	1	0	0	1	1	1	1
0	0	56	2	152	47.3	2.40	20.5	146	76	203	76	127.00	5.10	1	1.00	1	0	1	0	0	1	1	1	0	1	1
1	1	65	1	166	83.4	2.70	30.1	116	72	184	63	121.00	6.40	1	1.00	0	1	1	1	1	1	1	0	0	1	1
1	1	72	1	158	58.1	-.70	23.2	119	57	156	62	94.00	5.90	1	1.00	0	0	1	1	0	1	1	1	0	1	1

0	0	76	2	149	56.4	4.40	25.4	115	62	220	54	166.00	5.40	1	1.00	1	1	1	0	1	0	1	0	0	0	1
1	1	34	2	158	50.7	-4.40	20.3	109	68	216	70	146.00	5.20	1	1.00	1	0	1	0	1	1	1	1	1	1	1
0	0	70	1	163	66.1	2.80	24.7	129	80	295	42	253.00	5.40	1	1.00	0	0	1	0	1	1	0	1	1	0	1
1	1	69	2	154	60.1	.20	25.2	99	59	225	41	184.00	5.10	1	1.00	1	0	1	1	1	0	1	0	0	0	1
1	1	55	2	157	51.6	-1.00	20.8	125	80	237	73	164.00	5.40	0	1.00	1	1	1	1	1	1	0	1	1	0	1
1	1	68	2	148	46.8	-2.00	21.4	103	59	228	55	173.00	6.20	1	1.00	1	1	1	0	1	1	0	1	1	0	0
0	0	79	1	162	69.0	.20	26.2	116	74	177	62	115.00	5.30	1	1.00	0	1	1	1	1	0	1	0	1	1	1
1	1	28	2	158	77.5	6.60	31.0	112	70	187	48	139.00	5.00	1	1.00	1	0	0	0	1	0	1	0	1	1	1
1	1	72	2	151	45.8	-2.10	20.2	118	66	251	38	213.00	5.60	1	1.00	1	0	1	0	1	0	1	1	0	0	1
0	0	74	2	154	53.0	5.40	22.4	129	63	160	69	91.00	5.90	1	1.00	1	0	1	0	1	0	0	1	0	1	0
1	1	57	2	146	50.2	-.10	23.5	121	78	234	61	173.00	5.60	1	1.00	1	0	1	0	0	0	1	1	1	0	1
1	1	68	2	142	45.1	3.80	22.4	156	97	198	61	137.00	5.60	1	1.00	1	0	1	0	1	0	0	1	0	1	1
1	1	62	1	170	68.8	-4.90	23.7	137	82	234	35	199.00	10.20	0	1.00	1	0	1	0	0	1	1	1	1	0	0
1	1	49	2	154	46.0	2.60	19.3	86	59	235	59	176.00	5.80	1	1.00	1	0	1	1	1	1	1	1	1	0	1
0	0	50	2	149	62.0	1.20	27.9	188	98	211	67	144.00	5.50	1	1.00	1	0	1	0	1	1	1	0	0	1	1
0	0	76	1	160	53.0	2.40	20.5	138	83	157	66	91.00	5.60	0	1.00	0	0	1	0	1	1	1	1	1	1	1
1	1	76	2	158	53.7	.50	21.4	131	73	239	58	181.00	6.00	1	1.00	1	1	1	0	1	1	1	1	1	0	1
0	0	56	2	161	58.9	-1.10	22.6	118	66	244	61	183.00	5.30	1	1.00	1	0	1	0	1	0	1	1	1	0	1
1	1	77	2	138	49.8	1.20	26.0	156	87	186	61	125.00	6.10	1	1.00	1	0	1	0	1	1	0	0	0	1	1
0	0	41	1	173	64.6	2.10	21.4	115	74	290	49	241.00	5.40	0	1.00	1	0	0	0	0	1	0	1	1	0	1
0	1	68	1	162	67.4	.80	25.7	118	66	200	55	145.00	5.70	1	1.00	1	1	1	1	1	1	1	0	0	1	1
0	0	68	2	144	46.6	.20	22.4	126	63	238	42	196.00	5.70	1	1.00	1	0	1	0	1	0	0	1	0	0	1
0	0	69	1	159	68.4	1.00	27.1	123	77	171	50	121.00	5.90	1	1.00	1	0	1	0	0	1	1	0	1	1	0
0	0	59	2	152	60.3	5.70	25.9	100	72	224	69	155.00	6.50	1	1.00	1	0	1	1	0	1	0	0	0	0	0
1	1	37	2	165	49.4	3.90	18.2	120	64	194	58	136.00	5.30	0	1.00	1	0	1	0	1	0	0	1	1	1	1
1	1	55	2	152	51.9	-.50	22.6	130	78	175	72	103.00	6.30	1	1.00	1	1	1	1	1	0	0	1	0	1	1
1	1	67	1	153	58.1	2.10	24.9	131	88	223	70	153.00	5.70	1	1.00	0	1	1	1	1	1	1	1	0	0	1
0	0	62	1	166	50.7	2.70	18.3	109	68	210	84	126.00	5.90	1	1.00	1	1	1	0	1	0	1	1	1	1	1
1	1	70	1	170	82.1	-1.50	28.3	132	73	205	55	150.00	8.70	1	1.00	1	1	1	1	1	0	0	0	1	1	0
1	1	70	2	148	57.1	1.40	25.9	144	82	200	66	134.00	6.10	1	1.00	1	0	1	1	1	0	1	0	0	1	0
1	1	63	1	171	65.1	-.30	22.2	93	67	175	58	117.00	5.30	1	1.00	0	1	1	1	1	1	1	1	1	1	1
1	1	64	2	159	59.2	-3.30	23.2	93	59	252	53	199.00	6.20	1	1.00	1	0	1	1	1	1	1	1	1	0	1
1	1	65	2	146	58.8	.80	27.5	129	83	281	59	222.00	6.30	1	1.00	1	0	1	1	1	1	1	0	1	0	1
0	1	69	2	152	43.1	1.60	18.6	142	75	196	58	138.00	5.70	1	1.00	1	1	1	1	1	1	1	1	0	1	1
1	1	73	2	150	51.7	-.90	22.9	131	71	217	43	174.00	6.00	1	1.00	1	1	1	1	1	1	1	1	0	1	1
0	0	66	2	143	42.0	-.30	20.5	140	72	204	68	136.00	5.80	1	1.00	1	0	1	1	1	1	0	1	0	1	1
1	1	73	2	147	52.8	.00	24.4	152	103	236	52	184.00	5.70	1	1.00	1	1	1	1	0	0	0	1	0	0	1
1	1	72	2	150	59.7	1.60	26.7	128	70	224	84	140.00	5.10	1	1.00	1	1	1	0	1	0	0	0	1	0	1
0	0	66	1	156	61.5	-2.20	25.3	99	62	170	58	112.00	5.60	1	1.00	1	0	1	1	1	1	1	0	1	1	1

1	1	40	2	160	46.7	.40	18.3	94	57	248	81	167.00	5.60	1	1.00	1	0	1	0	1	1	1	1	1	0	1
1	1	68	1	151	63.8	-1.90	27.9	124	70	174	48	126.00	5.50	1	1.00	1	1	1	0	1	1	1	0	0	1	1
1	1	79	2	138	43.9	1.10	23.1	162	84	145	42	103.00	5.40	1	1.00	1	0	1	1	1	1	1	1	0	1	1
0	0	76	2	147	42.9	.90	19.9	149	82	230	69	161.00	5.40	1	1.00	1	0	1	0	1	0	0	1	0	0	1
0	0	68	1	166	64.8	-1.30	23.6	93	54	211	44	167.00	6.70	1	1.00	0	0	1	0	1	1	1	1	1	1	0
1	1	70	2	147	69.3	-.50	32.2	117	84	178	57	121.00	6.00	1	1.00	1	1	1	1	1	1	1	0	0	1	1
1	1	66	2	158	63.0	-.90	25.1	104	65	173	44	129.00	5.90	1	1.00	1	0	1	0	1	1	0	0	1	1	1
1	1	70	1	156	54.3	-.50	22.4	137	85	165	45	120.00	6.20	1	1.00	1	0	1	1	1	1	1	1	1	1	1
1	1	69	2	158	53.4	-2.90	21.5	123	83	162	68	94.00	6.00	1	1.00	1	0	1	1	1	1	0	1	1	1	1
1	1	72	1	162	68.8	2.90	26.3	127	90	217	60	157.00	5.90	0	1.00	1	1	1	0	1	1	1	0	0	1	1
1	1	65	1	162	53.6	-2.70	20.3	115	75	200	107	93.00	5.50	0	1.00	0	1	1	1	1	1	1	1	1	1	1
1	1	89	2	150	49.0	-2.30	21.8	132	69	226	80	146.00	5.20	1	1.00	1	0	1	1	1	1	1	1	0	0	1
0	0	69	2	138	39.6	1.80	20.8	122	68	214	78	136.00	5.60	1	1.00	1	0	1	0	0	0	0	1	1	1	1
0	0	27	1	170	61.4	-2.90	21.2	110	65	205	71	134.00	5.10	0	1.00	0	0	1	0	0	0	0	1	1	1	1
1	1	37	2	151	56.3	-5.00	24.8	122	68	209	46	163.00	5.40	1	1.00	1	0	1	1	1	1	1	1	1	1	1
1	1	70	2	141	56.9	-2.10	28.8	127	87	193	60	133.00	6.00	1	1.00	1	0	1	1	1	0	1	0	1	0	1
0	0	76	2	127	33.9	-1.70	21.1	131	77	226	58	168.00	6.40	1	1.00	1	0	1	1	1	1	1	1	0	0	1
1	1	89	2	137	31.4	.20	16.6	128	63	227	58	169.00	5.70	1	1.00	1	0	1	1	1	1	1	1	1	0	1
1	1	71	1	156	58.9	3.50	24.0	128	76	207	72	135.00	5.50	1	1.00	0	0	1	1	1	1	1	1	1	1	1
1	1	77	1	163	72.6	-1.50	27.4	145	84	198	58	140.00	5.90	1	1.00	1	0	1	1	1	0	1	0	0	1	1
1	1	68	2	149	76.9	.30	34.8	138	84	199	61	138.00	5.70	1	1.00	1	0	1	1	1	1	1	0	0	1	1
1	1	43	2	148	49.4	2.00	22.4	97	53	173	68	105.00	5.90	1	1.00	1	1	1	0	1	0	1	1	1	1	1
1	1	75	2	144	49.2	2.70	23.6	143	81	178	62	116.00	4.60	1	1.00	1	0	1	1	1	0	1	1	0	1	1
1	1	78	1	164	71.3	-.70	26.5	120	68	227	41	186.00	5.80	1	1.00	0	0	1	1	1	1	1	0	0	0	1
1	1	77	2	144	42.7	-.30	20.6	127	62	171	60	111.00	5.70	1	1.00	1	0	1	0	1	0	0	1	1	1	1
1	1	81	1	155	65.3	1.10	27.2	145	76	196	54	142.00	5.80	1	1.00	1	1	1	1	1	1	1	0	0	1	1
1	1	66	1	156	56.1	-1.00	22.9	123	73	190	46	144.00	5.70	0	1.00	1	0	1	1	1	1	0	1	1	1	1
1	1	68	2	149	61.2	3.10	27.4	112	62	206	60	146.00	5.20	1	1.00	0	0	1	0	1	1	1	0	1	1	1
1	1	63	2	155	51.2	-.90	21.2	111	71	203	73	130.00	5.50	1	1.00	1	0	1	0	0	1	0	1	1	1	1
1	1	76	2	150	54.1	-1.50	23.9	148	78	212	71	141.00	5.50	1	1.00	1	0	1	0	1	0	1	1	0	1	1
1	1	75	1	158	50.3	-2.10	20.2	100	65	228	92	136.00	5.40	1	1.00	0	0	1	1	1	1	1	1	1	0	1
0	0	70	2	155	56.5	2.10	23.6	114	72	237	79	158.00	6.30	1	1.00	1	0	1	0	1	1	1	1	1	0	0
1	1	75	2	153	55.1	-.70	23.6	157	80	223	46	177.00	5.40	1	1.00	1	0	1	0	0	0	0	1	0	0	1
1	1	53	2	154	56.3	-6.10	23.8	124	69	218	40	178.00	6.00	1	1.00	1	0	1	0	1	0	1	1	1	1	1
1	1	69	1	161	65.6	-1.30	25.4	110	66	166	67	99.00	5.60	1	1.00	0	0	1	1	1	1	1	0	1	1	1
1	1	60	2	156	71.2	1.60	29.2	134	82	188	85	103.00	6.10	1	1.00	0	0	1	1	1	1	1	0	0	0	1
0	0	56	2	161	51.0	-.60	19.7	121	74	225	70	155.00	5.10	1	1.00	1	0	1	0	1	0	1	1	1	0	1
1	1	58	2	163	62.5	1.60	23.4	126	83	196	80	116.00	5.50	1	1.00	1	0	1	0	1	0	0	1	1	1	1
1	1	63	2	148	51.0	2.70	23.2	105	66	177	66	111.00	5.20	1	1.00	1	1	1	1	1	0	0	1	0	1	1

0	0	71	1	167	66.7	1.70	24.0	145	67	208	42	166.00	6.60	1	1.00	0	0	1	0	0	1	0	1	0	1	0
0	0	72	2	140	44.2	2.50	22.7	120	70	251	97	154.00	5.80	1	1.00	1	0	1	1	1	0	0	1	0	0	1
1	1	76	1	165	55.4	.60	20.2	139	80	267	50	217.00	6.00	0	1.00	0	1	1	1	1	1	1	1	0	0	1
0	0	59	2	147	53.6	1.70	24.8	121	76	172	61	111.00	5.90	1	1.00	1	0	1	1	1	0	1	1	1	0	1
0	0	66	1	164	66.1	-.50	24.6	130	87	258	50	208.00	5.40	0	1.00	0	0	1	1	1	1	1	1	0	0	1
1	1	80	1	160	57.2	.10	22.4	132	77	213	69	144.00	6.70	1	1.00	0	0	1	1	1	1	1	1	1	1	0
1	1	53	2	146	62.5	2.70	29.3	130	81	239	52	187.00	9.00	1	1.00	1	0	1	0	1	0	1	0	1	0	0
0	0	64	2	154	54.8	1.30	23.0	120	70	181	66	115.00	5.40	1	1.00	1	0	1	1	1	0	1	1	1	1	1
0	0	55	1	164	58.6	-.20	21.8	105	59	190	58	132.00	5.20	0	1.00	0	0	1	1	1	1	1	1	1	1	1
1	1	71	1	167	81.0	-.30	28.9	135	81	166	49	117.00	5.90	1	1.00	0	0	1	0	1	0	1	0	0	1	1
1	1	77	2	141	34.9	1.80	17.5	125	64	182	61	121.00	6.50	1	1.00	1	0	1	1	1	0	1	1	1	1	0
1	1	33	1	162	71.3	.30	27.2	119	77	167	75	92.00	5.70	1	1.00	1	0	1	0	1	0	1	0	1	1	1
0	0	84	2	141	50.8	-.70	25.6	139	73	223	44	179.00	6.20	1	1.00	1	0	1	1	1	1	1	0	1	0	1
0	0	73	2	153	54.3	.30	23.1	163	95	204	52	152.00	5.40	1	1.00	1	1	1	1	1	1	1	1	0	1	1
1	1	40	2	156	44.0	-.40	18.0	110	72	172	59	113.00	5.60	1	1.00	1	0	1	0	1	1	1	1	1	1	1
1	1	50	1	173	71.6	-3.30	24.0	103	74	228	40	188.00	9.70	0	1.00	0	0	1	1	1	0	0	1	1	0	0
0	0	64	2	149	60.4	1.70	27.2	128	66	238	54	184.00	5.70	1	1.00	1	0	1	1	1	1	0	0	1	0	1
1	1	67	2	150	61.5	-3.10	27.3	137	83	185	70	115.00	5.60	1	1.00	1	1	1	0	1	0	0	0	0	1	1
1	1	48	2	153	52.9	-1.70	22.5	128	81	191	56	135.00	5.30	1	1.00	1	0	1	0	0	1	1	1	1	1	1
1	1	69	1	164	60.9	1.80	22.6	138	87	196	82	114.00	5.80	0	1.00	0	1	1	0	1	0	0	1	0	1	1
1	1	27	2	154	46.0	.60	19.3	100	56	193	61	132.00	5.50	1	1.00	1	0	1	1	1	1	1	1	1	1	1
1	1	75	2	143	50.8	-3.10	24.7	127	66	173	70	103.00	4.20	1	1.00	1	0	1	1	1	0	0	1	0	1	0
0	0	72	2	152	61.4	4.40	26.7	134	68	266	55	211.00	5.70	1	1.00	1	0	1	1	1	1	1	0	0	0	1
0	0	41	1	166	76.4	-1.10	27.7	147	93	258	52	206.00	5.30	1	1.00	0	0	1	1	0	1	1	0	0	0	1
1	1	63	2	150	51.5	-.10	23.0	101	57	171	53	118.00	5.30	1	1.00	1	0	1	1	1	0	0	1	1	1	1
0	0	47	1	181	66.6	1.60	20.2	115	77	187	69	118.00	4.90	0	1.00	0	0	0	0	1	0	0	1	1	1	1
1	1	60	2	155	60.7	-3.50	25.1	119	64	252	54	198.00	5.60	1	1.00	1	1	1	1	0	1	1	0	1	0	1
1	1	68	2	142	46.2	1.30	22.8	107	67	213	66	147.00	5.50	1	1.00	1	1	1	0	1	1	1	1	1	1	1
1	1	72	2	154	54.7	.10	22.9	135	79	172	67	105.00	5.70	1	1.00	1	0	1	0	1	0	0	1	0	1	1
1	1	82	1	154	51.4	-.90	21.7	138	65	211	46	165.00	6.00	1	1.00	1	1	1	1	1	1	1	1	0	1	1
1	1	53	1	170	54.3	3.40	18.7	151	91	189	66	123.00	5.40	0	1.00	1	0	1	0	0	1	1	1	0	1	1
1	1	78	2	147	52.8	.80	24.4	154	84	167	38	129.00	6.10	1	1.00	1	1	1	1	1	0	1	1	0	0	1
1	1	37	1	174	78.9	4.20	26.2	130	76	267	55	212.00	5.60	0	1.00	1	0	1	1	1	0	1	0	1	0	1
1	1	41	2	156	53.6	-1.00	21.9	108	70	197	94	103.00	5.00	1	1.00	1	0	1	1	1	0	1	1	1	1	1
1	1	74	2	155	57.6	-3.30	24.0	109	57	118	38	80.00	4.30	1	1.00	1	0	1	1	1	1	1	1	1	0	1
0	0	69	1	163	79.1	2.30	29.6	177	98	161	62	99.00	8.00	0	1.00	0	0	1	0	1	1	1	0	0	1	0
0	0	66	2	154	69.4	.10	29.3	131	67	200	47	153.00	5.90	1	1.00	1	0	1	1	0	1	1	0	0	1	1
0	0	62	2	155	69.9	-7.20	29.0	110	65	165	57	108.00	6.20	1	1.00	1	0	1	0	1	1	1	0	0	1	0
1	1	68	1	156	59.9	2.20	24.5	125	81	182	50	132.00	5.50	1	1.00	0	1	1	1	1	1	1	1	0	1	1

1	1	79	2	141	44.4	-1.70	22.2	114	64	207	53	154.00	5.40	1	1.00	1	0	1	0	1	0	0	1	1	1	1
0	0	44	2	153	62.3	5.20	26.5	121	67	187	55	132.00	4.70	1	1.00	1	1	1	1	1	1	1	0	1	1	1
1	1	65	2	153	53.3	.30	22.7	125	76	286	60	226.00	6.20	1	1.00	1	0	1	1	1	1	1	1	1	0	1
0	0	73	1	165	57.7	.20	21.2	150	76	185	49	136.00	5.20	1	1.00	0	1	1	1	1	1	1	1	0	1	1
1	1	82	1	154	62.0	4.40	26.0	127	66	170	37	133.00	6.40	1	1.00	1	0	1	0	1	1	1	0	0	0	0
1	1	62	2	163	59.3	-2.70	22.3	124	78	287	47	240.00	5.60	1	1.00	1	0	1	1	1	0	1	1	1	0	1
1	1	66	2	153	64.4	-.30	27.3	117	61	156	70	86.00	5.60	1	1.00	1	0	1	0	1	0	0	0	1	1	1
1	1	66	2	146	53.0	.30	24.9	168	97	182	43	139.00	5.70	1	1.00	1	1	1	0	1	1	1	1	0	1	1
1	1	72	2	151	49.9	.60	21.7	111	69	173	57	116.00	5.50	1	1.00	1	1	1	0	1	1	1	1	0	1	1
1	1	60	2	145	61.3	-2.50	29.1	118	73	243	67	176.00	5.90	1	1.00	1	1	1	0	1	1	1	0	1	0	1
1	1	74	2	145	44.1	4.70	20.9	121	68	191	80	111.00	5.40	1	1.00	1	0	1	0	1	1	1	1	0	1	1
1	1	75	1	173	67.8	.50	22.5	114	77	148	51	97.00	4.70	0	1.00	0	1	1	1	0	1	1	1	0	1	1
1	1	65	2	155	53.6	.30	22.4	104	64	245	40	205.00	5.60	1	1.00	1	0	1	1	1	1	1	1	1	0	1
1	1	57	2	154	49.6	1.90	20.9	102	55	217	65	152.00	5.40	1	1.00	1	1	1	0	1	1	1	1	1	1	1
1	1	62	1	159	68.1	-1.30	26.9	135	72	174	40	134.00	5.80	0	1.00	1	0	1	0	1	1	0	0	1	1	1
0	0	44	2	151	59.6	1.40	26.1	113	64	196	84	112.00	4.90	1	1.00	0	0	1	1	1	0	0	0	1	1	1
1	1	47	2	154	56.0	.50	23.6	125	72	159	55	104.00	5.10	0	2.00	0	0	1	0	1	1	1	1	1	1	1
1	1	25	2	151	55.5	-.80	24.4	109	63	149	54	95.00	5.40	1	1.00	1	0	1	1	1	0	1	1	1	1	1
1	1	70	2	154	64.3	-1.70	27.1	140	75	192	57	135.00	6.00	1	1.00	1	0	1	1	1	1	1	0	0	0	1
1	1	47	2	162	66.1	-3.00	25.1	119	68	258	46	212.00	5.70	1	1.00	1	0	1	1	1	1	1	0	1	0	1
1	1	78	2	135	47.6	-1.50	26.2	131	79	216	53	163.00	5.80	1	1.00	1	1	1	0	0	1	1	0	1	1	1
1	1	74	2	138	39.5	-1.60	20.6	100	59	150	64	86.00	5.80	1	1.00	1	0	1	0	1	0	0	1	1	1	1
1	1	76	2	138	39.8	.20	20.8	147	75	191	61	130.00	5.40	1	1.00	1	0	1	0	1	1	1	1	0	1	1
0	1	58	1	161	57.9	.50	22.4	149	89	184	70	114.00	5.70	0	2.00	0	1	1	1	1	1	1	1	0	1	1
1	1	61	2	152	56.9	3.80	24.5	158	84	186	93	93.00	5.60	1	1.00	1	0	1	0	0	0	1	1	0	1	1
1	1	60	1	172	69.1	-.90	23.3	151	81	201	67	134.00	6.00	1	1.00	0	0	1	0	0	0	1	1	0	1	1
1	1	56	2	156	59.5	-2.00	24.3	115	71	241	105	136.00	5.10	1	1.00	0	0	1	0	0	0	1	1	1	0	1
0	0	72	2	149	50.0	.80	22.6	126	68	169	70	99.00	5.80	1	1.00	1	0	1	1	0	0	0	1	1	1	1
1	1	45	2	161	54.3	.60	21.0	130	83	152	70	82.00	5.50	1	1.00	1	0	1	0	0	1	1	1	0	1	1
1	1	43	2	165	55.3	-.10	20.2	103	55	169	76	93.00	4.70	1	1.00	1	1	1	1	1	1	1	1	1	1	1
0	0	35	2	157	64.6	.90	26.1	102	60	205	59	146.00	5.30	1	1.00	1	0	1	0	1	1	1	0	1	1	1
1	1	29	2	165	54.0	2.20	19.9	95	56	163	77	86.00	5.10	0	1.00	1	0	0	1	1	1	1	1	1	1	1
0	0	87	2	144	54.0	1.50	26.0	138	64	204	65	139.00	5.50	1	1.00	1	0	1	1	1	1	1	0	0	1	1
0	0	77	1	156	51.6	2.20	21.1	95	64	164	79	85.00	5.80	1	1.00	1	0	1	1	1	1	0	1	1	0	1
0	0	46	1	164	81.4	1.50	30.4	139	85	221	63	158.00	5.70	0	1.00	0	1	1	0	0	0	0	0	1	0	1
0	0	50	2	165	48.0	1.80	17.5	110	59	244	102	142.00	5.20	1	1.00	1	0	1	1	1	0	0	1	1	0	1
1	1	69	1	160	64.7	-1.80	25.2	137	95	169	51	118.00	5.70	1	1.00	0	0	1	0	1	1	1	0	0	1	1
1	1	39	1	177	68.2	3.70	21.8	112	66	136	78	58.00	5.00	0	1.00	0	0	1	0	1	0	0	1	1	1	1
0	0	75	1	167	70.1	-2.80	25.2	158	92	143	49	94.00	5.30	1	1.00	1	0	1	0	0	0	0	0	0	1	1

1	1	70	1	154	44.5	3.50	18.8	119	63	146	60	86.00	5.80	1	1.00	1	1	1	0	1	1	1	1	1	1	0
0	0	74	1	162	58.3	.60	22.1	119	83	203	40	163.00	6.50	1	1.00	1	1	1	1	1	1	0	1	1	1	0
1	1	50	2	150	42.3	.20	18.9	148	80	135	74	61.00	5.90	1	1.00	1	0	1	0	0	0	0	1	0	1	1
1	1	59	1	170	52.9	2.10	18.3	136	79	220	95	125.00	5.30	0	2.00	0	0	1	0	0	1	1	1	1	0	1
1	1	43	2	156	46.1	-1.40	19.0	100	62	146	56	90.00	5.20	1	1.00	1	1	1	1	1	1	1	1	1	1	1
1	1	63	1	165	59.6	2.50	21.9	140	85	175	60	115.00	6.50	0	1.00	0	1	1	1	1	1	1	1	0	1	0
0	0	61	1	169	67.5	-1.40	23.7	121	81	176	43	133.00	5.90	1	1.00	1	0	1	0	0	1	1	1	0	1	1
1	1	64	1	165	75.7	-.90	27.6	137	96	236	31	205.00	6.40	1	1.00	0	0	1	1	1	1	1	0	0	0	1
1	1	59	2	154	44.7	-.10	18.8	146	82	187	107	80.00	5.40	1	1.00	1	0	1	0	0	0	0	1	0	1	1
1	1	55	2	162	64.3	-1.10	24.3	125	72	230	47	183.00	5.70	1	1.00	0	0	1	1	0	1	0	1	1	0	1
1	1	68	2	137	45.3	.30	24.1	158	75	212	61	151.00	5.50	1	1.00	1	0	1	1	1	0	1	1	0	1	1
1	1	45	2	154	60.4	-2.60	25.5	136	90	217	66	151.00	4.60	1	1.00	1	1	1	0	1	0	0	0	0	1	1
1	1	23	2	156	49.7	-1.10	20.5	100	54	172	73	99.00	4.80	1	1.00	1	0	1	1	1	1	1	1	1	1	1
1	1	70	2	146	43.7	-.60	20.5	123	66	202	81	121.00	5.60	1	1.00	1	0	1	1	1	1	1	1	1	1	1
1	1	58	2	153	53.2	-9.60	22.6	130	76	244	68	176.00	5.80	1	1.00	1	0	1	1	1	0	0	1	1	0	1
1	1	57	2	154	50.1	1.00	21.1	122	63	174	67	107.00	5.30	1	1.00	1	0	1	0	1	0	0	1	1	0	1
1	1	64	1	169	76.9	-12.60	26.8	153	83	247	77	170.00	8.20	1	1.00	1	1	1	1	1	1	1	0	0	0	0
1	1	72	2	140	42.7	-2.10	21.8	145	79	216	88	128.00	5.20	1	1.00	0	1	1	1	1	1	1	1	0	1	1
1	1	84	2	150	53.7	-.90	23.8	117	68	181	64	117.00	5.20	1	1.00	1	0	1	1	1	1	1	1	1	1	1
1	1	70	1	159	57.7	-.10	22.7	160	93	159	61	98.00	5.60	0	1.00	1	1	1	0	1	1	0	1	0	1	1
0	0	71	2	149	51.1	-1.40	23.1	120	66	115	54	61.00	5.60	1	1.00	1	0	1	0	1	0	1	1	1	1	1
1	1	48	2	157	53.4	8.30	21.7	129	81	227	104	123.00	5.40	1	1.00	1	0	1	1	0	0	1	1	1	0	1
1	1	65	2	152	48.1	-1.50	20.7	119	70	184	64	120.00	5.60	1	1.00	0	1	1	0	1	1	1	1	1	1	1
1	1	80	2	148	48.3	.60	22.1	146	76	178	70	108.00	5.40	1	1.00	1	0	1	1	1	0	1	1	0	1	0
1	1	41	2	157	56.1	-1.90	22.7	106	57	167	51	116.00	5.30	0	1.00	1	0	1	0	0	0	0	1	1	1	1
1	1	66	1	163	61.8	-2.30	23.1	120	78	183	41	142.00	7.80	1	1.00	1	0	1	0	1	1	1	1	1	1	0
0	1	67	2	156	53.8	.40	22.0	130	68	211	95	116.00	5.80	1	1.00	0	1	1	0	1	1	0	1	0	1	1
1	1	47	2	158	62.2	-4.50	25.0	129	80	146	37	109.00	5.60	1	1.00	1	0	1	0	0	0	1	0	1	0	1
1	1	71	1	168	64.1	-1.10	22.8	141	76	136	46	90.00	4.60	1	1.00	0	0	1	1	1	1	1	1	0	0	1
1	1	63	2	160	74.0	-8.80	28.7	103	54	207	76	131.00	5.30	1	1.00	1	0	1	1	0	0	0	0	1	1	1
1	1	49	2	163	54.3	.00	20.5	120	71	212	80	132.00	5.80	1	1.00	0	0	1	1	1	1	1	1	1	1	1
1	1	69	2	159	81.7	-1.10	32.1	142	80	204	55	149.00	5.70	1	1.00	1	0	1	0	1	0	1	0	0	1	1
1	1	59	2	143	48.8	-3.10	23.7	154	84	214	104	110.00	5.00	1	1.00	0	1	1	0	0	0	1	1	0	1	1
1	1	69	2	153	51.2	-3.90	21.9	130	70	166	61	105.00	5.80	1	1.00	1	1	1	1	1	1	1	1	1	1	1
0	0	47	1	168	66.0	-2.60	23.2	116	67	184	49	135.00	5.90	1	1.00	1	0	1	0	1	1	1	1	1	1	1
1	1	75	2	155	49.7	.40	20.8	127	79	185	72	113.00	5.40	1	1.00	1	0	1	1	1	1	0	1	0	1	1
0	0	67	1	171	79.0	-.30	26.9	152	94	163	63	100.00	5.30	1	1.00	0	0	1	1	1	1	1	0	0	1	1
1	1	70	2	156	44.0	-1.00	18.1	98	65	189	82	107.00	5.80	1	1.00	1	0	1	0	1	1	0	1	1	1	1
1	1	67	2	156	67.4	-2.00	27.7	150	76	204	52	152.00	5.10	1	1.00	1	0	1	0	1	1	0	0	0	1	1

0	0	63	2	149	66.6	-.70	30.0	121	59	185	63	122.00	5.60	1	1.00	0	1	1	0	0	1	1	0	0	1	1
0	0	66	2	151	38.6	-.10	16.8	136	90	182	47	135.00	5.30	1	1.00	1	0	1	0	1	0	1	1	0	0	1
1	1	68	1	163	55.6	-2.90	21.0	123	80	168	49	119.00	5.70	1	1.00	0	1	1	1	1	1	1	1	1	1	1
1	1	71	2	147	75.2	-.90	34.6	122	69	237	56	181.00	7.50	1	1.00	1	0	1	1	1	1	1	0	0	0	0
0	0	74	1	165	66.7	-9.80	24.4	124	76	166	44	122.00	6.70	1	1.00	1	0	1	1	1	1	1	1	0	1	0
0	0	37	1	171	117.2	-8.20	40.0	155	94	271	48	223.00	5.60	1	1.00	0	0	1	1	1	0	0	0	0	0	1
1	1	72	2	148	50.1	-.30	22.9	117	68	157	68	89.00	5.20	1	1.00	0	0	1	1	1	1	1	1	1	1	1
0	0	60	2	158	48.8	.30	19.4	128	76	252	63	189.00	5.70	1	1.00	1	0	1	0	1	1	1	1	1	0	1
0	0	79	2	140	43.9	3.10	22.3	139	75	197	59	138.00	5.70	1	1.00	1	0	1	0	0	1	1	1	0	1	1
0	0	74	2	151	47.2	3.70	20.7	112	73	231	69	162.00	6.30	1	1.00	1	1	1	1	0	1	1	1	1	0	1
1	1	52	2	157	61.0	-5.70	24.9	124	85	223	72	151.00	4.90	1	1.00	1	1	1	0	1	1	1	1	1	0	1
0	0	27	2	150	44.0	1.80	19.4	126	69	143	88	55.00	4.70	1	1.00	1	0	1	1	1	1	1	1	1	1	1
1	1	62	1	160	61.4	-1.30	24.1	110	64	245	57	188.00	5.30	1	1.00	0	0	1	0	1	1	1	1	1	0	1
0	0	61	2	152	52.7	1.30	22.7	150	82	213	63	150.00	5.70	1	1.00	1	1	1	0	0	0	1	1	0	1	1
0	0	71	1	157	79.6	-2.50	32.4	136	90	125	39	86.00	5.10	1	1.00	1	0	1	1	1	1	0	0	0	0	1
1	1	49	2	150	48.9	-.70	21.7	132	73	195	83	112.00	5.40	1	1.00	0	0	1	1	1	0	1	1	1	1	1
1	1	58	1	167	65.0	-.90	23.2	123	89	211	63	148.00	4.90	1	1.00	0	1	1	0	0	1	1	1	1	1	1
0	0	66	2	145	50.4	.30	23.8	119	66	215	58	157.00	5.40	1	1.00	1	0	1	0	0	0	1	1	0	1	1
1	1	56	2	155	53.4	2.00	22.3	138	81	186	72	114.00	5.20	1	1.00	1	0	1	0	0	0	0	1	0	1	1
0	0	72	1	159	61.1	-2.00	24.0	129	72	222	58	164.00	5.30	1	1.00	0	1	1	0	1	1	1	1	0	0	1
0	0	71	2	150	49.5	.80	22.0	118	62	211	54	157.00	6.10	1	1.00	1	0	1	0	1	0	0	1	1	0	0
1	1	75	2	142	44.3	.20	22.0	160	83	180	70	110.00	5.40	1	1.00	0	0	1	1	1	1	1	1	0	1	1
1	1	29	1	159	41.3	-.20	16.3	133	83	139	66	73.00	4.80	1	1.00	1	0	1	1	1	1	1	1	1	1	1
1	1	58	2	156	49.4	3.20	20.2	128	74	214	61	153.00	5.70	1	1.00	0	1	0	1	1	1	1	1	1	1	1
0	0	79	1	173	57.1	-2.90	19.1	132	67	233	61	172.00	5.40	1	1.00	1	0	1	0	1	0	0	1	1	0	1
1	1	64	1	176	71.7	-2.70	23.0	135	86	193	53	140.00	6.10	1	1.00	0	0	1	1	1	1	0	1	0	1	1
1	1	59	2	152	50.9	2.00	22.0	110	71	210	87	123.00	6.00	1	1.00	1	0	0	1	1	1	0	1	1	0	1
0	0	46	1	170	70.1	-.70	24.3	112	68	181	63	118.00	5.10	0	1.00	0	1	1	1	1	1	1	1	1	1	1
1	1	63	2	153	65.3	1.90	27.9	123	82	181	64	117.00	5.40	1	1.00	1	0	1	0	0	0	1	0	1	1	1
1	1	64	2	141	42.9	2.00	21.7	157	80	221	55	166.00	5.60	1	1.00	1	1	1	1	1	1	1	1	0	0	1
1	1	71	1	164	69.7	-2.40	25.8	140	80	213	65	148.00	5.40	1	1.00	0	0	1	1	1	0	1	0	0	1	1
0	0	70	2	143	39.3	1.50	19.1	103	66	169	86	83.00	5.00	1	1.00	1	0	1	0	1	1	1	1	1	1	1
1	1	62	1	166	66.1	-2.60	24.1	124	78	178	75	103.00	5.50	1	1.00	0	0	1	1	0	1	1	1	0	1	1
0	0	73	1	152	40.6	2.30	17.5	114	75	231	137	94.00	5.60	0	1.00	0	0	1	0	0	0	1	1	0	0	1
1	1	65	2	144	42.1	2.30	20.3	128	73	213	70	143.00	6.10	1	1.00	1	1	1	1	1	1	1	1	0	1	1
1	1	48	2	158	56.9	-1.90	22.7	116	69	219	54	165.00	5.40	1	1.00	0	1	1	1	1	1	1	1	1	1	1
1	1	71	1	156	64.9	-3.50	26.6	119	66	153	73	80.00	5.60	0	1.00	0	0	1	0	1	0	1	0	0	1	1
0	0	58	1	159	57.0	-3.30	22.5	129	92	194	49	145.00	5.40	1	1.00	0	1	1	1	0	0	1	1	0	1	1
1	1	76	2	142	50.7	-1.20	25.2	125	84	218	53	165.00	5.70	1	1.00	1	0	1	1	1	1	1	0	1	1	1

0	0	54	2	154	50.0	1.00	21.0	105	65	200	77	123.00	6.20	1	1.00	1	0	1	0	0	0	1	1	1	1	1
1	1	75	1	150	57.3	.70	25.3	141	71	202	49	153.00	5.80	1	1.00	0	0	1	1	1	1	1	0	0	1	1
1	1	66	1	162	56.8	-.20	21.6	104	59	147	73	74.00	5.60	0	1.00	0	1	1	1	1	1	1	1	1	1	1
1	1	62	2	146	45.1	1.10	21.0	115	58	193	73	120.00	5.90	1	1.00	1	0	1	0	1	0	1	1	1	1	1
1	1	63	2	155	68.9	3.10	28.6	153	76	210	48	162.00	6.20	1	1.00	1	0	1	0	1	0	1	0	0	1	1
1	1	63	2	156	61.3	.20	25.3	144	95	294	60	234.00	5.40	1	1.00	1	0	1	1	1	0	0	0	0	0	1
0	0	41	2	158	46.8	-1.10	18.8	119	78	210	74	136.00	5.20	0	1.00	0	0	1	0	0	0	1	1	1	1	1
0	0	47	2	160	70.6	-2.90	27.5	121	73	208	59	149.00	5.30	1	1.00	0	0	1	1	1	0	1	0	1	1	1
1	1	63	1	163	64.2	.60	24.1	110	74	221	49	172.00	5.30	1	1.00	0	0	1	0	1	1	1	1	0	0	1
0	0	59	2	158	71.4	1.20	28.5	145	88	226	67	159.00	4.80	1	1.00	1	0	1	0	0	0	1	0	0	0	1
1	1	65	2	153	62.9	-.60	26.9	133	80	236	69	167.00	7.00	1	1.00	1	0	1	1	1	0	1	0	1	0	0
1	1	69	1	165	58.0	-.30	21.3	130	63	185	117	68.00	5.80	1	1.00	0	0	1	0	1	0	0	1	0	1	1
1	1	74	2	153	56.3	-1.70	24.1	136	87	235	45	190.00	5.40	1	1.00	1	0	1	1	0	0	0	1	1	0	1
1	1	82	1	160	60.7	4.10	23.6	131	76	188	53	135.00	5.40	0	2.00	0	0	1	0	0	1	1	1	1	1	1
0	0	69	2	151	49.8	1.00	21.8	121	79	249	58	191.00	5.90	1	1.00	0	0	1	0	1	1	1	1	0	0	1
0	0	77	1	170	68.8	2.20	23.6	146	82	176	35	141.00	6.20	1	1.00	1	0	1	1	1	1	1	1	0	0	1
0	0	79	2	150	48.2	.20	21.4	121	61	231	55	176.00	5.50	1	1.00	1	0	1	0	0	1	1	1	0	0	1
1	1	74	2	148	41.9	.20	19.0	109	54	174	81	93.00	5.70	1	1.00	1	0	1	1	1	1	1	1	1	1	1
1	1	74	2	145	44.7	-2.10	21.2	111	62	227	46	181.00	5.20	1	1.00	1	1	1	1	1	0	1	1	1	0	1
0	0	68	2	147	48.1	.20	22.2	128	69	238	83	155.00	5.30	1	1.00	1	1	1	0	0	0	0	1	0	0	1
1	1	72	2	149	48.8	.10	21.9	130	79	181	37	144.00	5.70	1	1.00	1	0	1	0	1	1	1	1	0	0	1
0	0	77	2	135	46.9	-.80	25.8	96	53	182	79	103.00	5.90	1	1.00	1	0	1	0	1	0	1	0	1	1	1
0	0	78	1	165	62.7	-2.10	23.0	110	69	221	52	169.00	7.20	1	1.00	1	0	1	0	0	1	1	1	1	0	0
0	0	68	1	166	83.2	-.10	30.0	142	88	147	57	90.00	5.70	1	1.00	0	0	1	1	1	1	1	0	0	1	1
1	1	74	2	147	56.3	-3.90	26.2	104	64	234	57	177.00	6.60	1	1.00	1	0	1	0	1	0	1	0	0	0	0
0	0	57	2	142	42.6	.20	21.2	140	89	248	78	170.00	5.60	1	1.00	1	0	1	0	0	0	0	1	0	0	1
1	1	72	1	164	67.4	-1.90	25.0	139	74	193	56	137.00	5.60	0	1.00	1	1	1	0	1	1	1	0	1	1	1
1	1	75	2	152	64.8	-4.70	27.9	114	65	124	41	83.00	6.40	1	1.00	1	0	1	1	1	1	1	0	0	1	1
0	0	70	1	160	57.1	.90	22.2	175	84	250	101	149.00	5.60	1	1.00	0	1	1	1	1	1	1	1	0	0	1
1	1	59	2	146	46.6	-.70	21.8	122	75	194	72	122.00	5.10	1	1.00	0	0	1	1	1	0	0	1	1	1	1
1	1	75	2	144	46.3	-.40	22.4	128	74	206	68	138.00	5.50	1	1.00	1	0	1	1	1	1	1	1	0	1	1
0	0	59	1	162	60.9	2.10	23.3	126	86	183	67	116.00	5.00	1	1.00	0	1	1	0	1	1	1	1	0	1	1
1	1	75	2	146	52.4	1.30	24.6	137	69	185	71	114.00	5.70	1	1.00	1	0	1	1	1	0	0	1	1	0	1
0	0	62	1	161	59.5	-.80	22.8	167	88	199	43	156.00	8.10	1	1.00	0	1	1	0	1	1	1	1	0	1	0
1	1	73	2	140	47.8	-.30	24.3	134	75	207	60	147.00	5.70	1	1.00	1	0	1	0	1	0	0	1	1	1	1
1	1	77	1	155	53.1	1.60	22.1	134	67	195	77	118.00	5.50	1	1.00	1	0	1	0	1	0	1	1	1	1	1
0	0	55	1	161	61.6	2.20	23.7	145	100	201	57	144.00	5.50	0	1.00	0	1	1	1	1	0	0	1	0	1	1
1	1	57	2	147	77.0	2.50	35.5	139	98	244	73	171.00	6.00	1	1.00	1	0	1	0	1	1	1	0	0	0	1
1	1	53	2	164	62.5	-.50	23.1	144	87	231	123	108.00	5.40	1	1.00	0	0	1	0	0	0	0	1	0	0	1

1	1	43	2	153	51.5	-.10	22.0	113	74	214	116	98.00	4.80	1	1.00	0	0	1	0	1	0	0	1	1	1	1
1	1	45	2	157	60.2	2.00	24.4	108	69	181	58	123.00	5.40	1	1.00	0	1	1	0	1	1	1	1	1	1	1
1	1	70	2	146	54.2	1.40	25.3	133	83	179	82	97.00	5.60	1	1.00	1	0	1	0	1	0	1	0	1	1	1
1	1	72	2	147	47.1	.40	21.9	155	75	174	54	120.00	5.90	1	1.00	1	0	1	0	1	0	0	1	0	1	1
1	1	69	1	164	61.3	-1.60	22.8	124	75	203	44	159.00	5.70	1	1.00	1	1	1	0	1	1	1	1	0	0	1
1	1	44	1	159	79.0	.40	31.0	121	74	184	55	129.00	5.40	1	1.00	1	0	1	0	1	1	1	0	1	1	1
1	1	64	2	152	46.6	-.20	20.0	102	62	200	70	130.00	5.10	1	1.00	1	0	1	1	0	0	0	1	1	1	1
0	0	59	1	174	75.1	-1.10	24.8	134	88	187	42	145.00	5.20	0	1.00	0	0	1	0	0	0	0	1	1	1	1
1	1	74	1	173	77.2	1.10	25.8	138	90	174	57	117.00	5.50	1	1.00	0	0	1	1	1	1	1	0	0	1	1
1	1	61	2	157	56.8	.50	23.0	124	73	187	78	109.00	4.40	1	1.00	1	1	1	1	1	0	1	1	1	1	1
1	1	30	2	157	54.0	-.70	22.0	109	63	157	69	88.00	5.00	1	1.00	0	0	0	0	1	1	1	1	1	1	1
0	0	71	2	147	58.8	2.50	27.0	112	70	166	60	106.00	5.80	1	1.00	1	0	1	1	1	0	0	0	1	1	1
0	0	66	1	161	59.4	-1.30	23.0	106	69	222	53	169.00	5.40	1	1.00	0	1	1	0	1	0	1	1	1	0	1
1	1	85	2	148	53.1	-4.80	24.2	122	63	149	46	103.00	5.70	1	1.00	1	0	1	1	1	0	1	1	0	1	1
1	1	76	2	142	53.2	1.80	26.5	116	67	172	48	124.00	5.70	1	1.00	1	0	1	0	1	0	0	0	0	1	1
0	0	66	2	148	52.0	1.00	23.6	112	71	245	85	160.00	5.30	1	1.00	1	1	1	0	1	0	1	1	1	0	1
1	1	55	1	162	58.7	1.60	22.2	126	83	221	67	154.00	5.30	0	1.00	0	1	1	1	0	1	1	1	1	0	1
0	0	44	1	169	78.2	.30	27.3	124	84	205	46	159.00	5.40	1	1.00	0	0	1	0	0	1	1	0	1	1	1
0	0	75	2	153	45.3	.90	19.3	119	64	208	71	137.00	5.60	1	1.00	1	0	1	0	1	0	0	1	1	1	1
1	1	78	1	162	53.0	-4.90	20.1	131	77	194	86	108.00	6.70	1	1.00	0	0	1	1	1	1	1	1	0	1	0
1	1	62	2	144	53.4	1.40	25.7	140	73	206	53	153.00	5.60	1	1.00	1	0	1	0	1	0	1	0	0	1	1
1	1	61	1	169	71.2	-1.80	24.8	124	77	190	42	148.00	6.10	1	1.00	0	1	1	1	1	1	1	1	1	1	0
0	0	48	2	160	64.3	1.50	25.0	103	68	196	70	126.00	5.10	1	1.00	1	0	1	0	0	1	1	0	1	1	1
0	0	36	2	145	48.4	.70	23.1	111	74	236	79	157.00	5.10	1	1.00	0	1	1	1	0	1	1	1	1	0	1
1	1	68	2	149	44.6	.40	20.1	92	54	255	80	175.00	5.60	1	1.00	1	0	1	1	1	1	1	1	1	0	1
0	0	76	2	149	42.9	.70	19.4	124	62	217	71	146.00	5.60	1	1.00	1	0	1	0	1	0	0	1	1	1	1
1	1	70	1	171	60.5	-.50	20.6	128	78	179	77	102.00	5.40	1	1.00	0	0	1	1	1	0	1	1	0	1	1
1	1	70	2	151	50.0	-1.80	22.0	130	69	286	44	242.00	5.70	1	1.00	1	0	1	1	1	1	1	1	1	0	1
1	1	59	2	155	59.6	.10	24.8	136	78	185	48	137.00	5.60	1	1.00	1	0	1	1	1	0	1	1	1	0	1
1	1	69	1	167	81.1	.60	29.1	142	78	180	54	126.00	5.00	1	1.00	0	1	1	1	1	0	1	0	0	1	1
0	0	66	2	155	58.6	1.40	24.5	114	70	199	58	141.00	6.20	1	1.00	1	1	1	1	0	1	0	1	1	1	1
1	1	58	2	156	61.5	.40	25.3	135	79	233	58	175.00	5.40	1	1.00	0	1	1	1	1	1	1	0	1	0	1
1	1	37	1	168	69.2	-5.40	24.4	102	61	239	57	182.00	5.80	1	1.00	1	0	1	1	0	0	1	1	1	0	1
1	1	47	2	163	78.0	-4.20	29.5	125	71	152	48	104.00	5.90	1	1.00	1	0	1	0	0	0	0	0	1	1	1
1	1	77	1	169	61.1	.50	21.3	129	64	177	45	132.00	5.50	0	1.00	1	0	1	1	1	1	1	1	1	1	1
1	1	89	2	136	37.0	-1.30	20.1	88	54	178	53	125.00	5.20	1	1.00	1	0	1	1	1	1	1	1	1	1	1
1	1	39	1	180	87.1	-1.70	27.0	106	67	187	44	143.00	5.10	1	1.00	0	0	1	1	1	0	0	0	1	1	1
1	1	62	2	149	45.0	-2.00	20.4	147	81	249	73	176.00	5.60	1	1.00	1	0	1	1	1	1	0	1	0	0	1
1	1	59	1	165	63.8	-.50	23.5	149	104	345	57	288.00	5.10	1	1.00	0	0	1	0	1	0	0	1	0	0	1

1	1	54	2	151	48.2	-1.30	21.2	131	89	250	70	180.00	5.10	1	1.00	1	1	1	0	1	0	0	1	1	0	1
1	1	54	1	164	77.2	-3.00	28.8	149	106	219	76	143.00	5.50	1	1.00	0	0	1	0	1	1	1	0	0	1	1
1	1	67	1	168	61.0	1.30	21.5	108	61	309	55	254.00	5.80	1	1.00	0	0	1	0	1	0	0	1	0	0	1
1	1	49	1	174	67.7	6.60	22.2	119	76	93	41	52.00	5.80	1	1.00	1	0	1	1	0	0	0	1	1	1	0
1	1	81	1	160	52.8	-1.00	20.5	103	50	132	48	84.00	5.50	1	1.00	1	1	1	0	1	0	1	1	0	1	1
1	1	69	2	145	55.9	-3.30	26.6	190	95	235	65	170.00	6.40	1	1.00	1	0	1	1	1	0	1	0	0	0	1
1	1	85	2	142	53.5	-.90	26.5	134	64	265	53	212.00	6.10	1	1.00	1	0	1	1	1	1	1	0	0	0	1
1	1	79	2	142	50.4	4.10	24.9	109	71	241	87	154.00	5.30	1	1.00	1	0	1	0	1	1	0	1	0	0	1
1	1	60	2	143	42.2	-2.90	20.7	100	55	229	49	180.00	5.10	1	1.00	1	0	1	1	1	1	1	1	1	0	1
1	1	70	1	154	64.2	-1.40	27.2	145	72	193	64	129.00	5.80	1	1.00	0	0	1	0	1	0	1	0	0	1	1
1	1	65	2	146	42.8	2.10	20.1	141	76	225	87	138.00	6.00	1	1.00	1	0	1	0	0	1	0	1	0	0	1
1	1	78	2	157	64.0	1.70	25.8	119	71	170	62	108.00	5.50	1	1.00	1	1	1	1	1	1	1	0	0	1	1
1	1	68	2	151	55.7	1.10	24.5	137	82	203	85	118.00	5.60	1	1.00	1	0	1	1	1	1	1	1	0	1	1
1	1	90	2	143	35.1	.60	17.1	120	72	163	68	95.00	5.30	1	1.00	1	0	1	0	1	0	0	1	0	1	1
1	1	62	2	151	35.1	2.90	15.4	122	69	211	70	141.00	5.50	1	1.00	1	0	1	0	1	1	1	1	1	1	1
0	0	32	2	152	56.6	1.70	24.4	124	82	183	55	128.00	5.40	0	2.00	0	1	1	1	0	1	1	1	1	1	1
1	1	68	2	153	53.5	1.70	22.8	142	78	233	83	150.00	6.30	1	1.00	1	1	1	0	1	0	0	1	0	0	1
0	0	35	1	176	70.8	-1.00	22.9	128	75	176	63	113.00	5.10	0	1.00	0	1	1	0	1	1	0	1	1	1	1
1	1	67	1	166	65.8	.90	23.9	133	85	250	63	187.00	6.20	0	1.00	0	0	1	0	1	0	1	1	0	0	1
1	1	64	1	167	75.3	-.60	26.9	125	78	173	46	127.00	5.70	1	1.00	0	1	1	0	1	1	1	0	1	1	1
1	1	80	1	162	55.6	1.00	21.1	141	77	155	57	98.00	5.50	1	1.00	0	0	1	1	1	1	0	1	0	1	1
1	1	49	2	160	49.7	6.20	19.4	110	62	167	70	97.00	5.20	1	1.00	1	0	1	1	0	1	1	1	1	1	1
1	1	58	2	160	62.3	3.20	24.3	114	69	260	103	157.00	5.30	1	1.00	1	0	1	1	1	0	1	1	1	0	1
1	1	72	1	164	70.0	2.00	26.1	116	65	218	59	159.00	5.60	1	1.00	1	0	1	1	1	1	1	0	0	1	1
0	0	63	2	152	46.1	.80	19.9	100	58	230	77	153.00	6.20	1	1.00	1	0	1	1	1	1	1	1	1	0	1
0	0	50	2	154	43.5	.20	18.4	107	75	267	82	185.00	5.40	1	1.00	1	0	0	0	1	0	0	1	1	0	1
1	1	67	2	149	52.1	-2.50	23.4	126	65	190	72	118.00	5.20	1	1.00	1	1	1	0	1	0	0	1	0	1	1
1	1	63	2	144	47.4	1.70	22.7	110	73	198	68	130.00	5.40	1	1.00	1	0	1	1	1	0	0	1	1	1	1
0	0	61	2	157	55.2	1.90	22.5	121	66	241	69	172.00	5.30	1	1.00	1	1	1	0	0	1	0	1	1	0	1
0	0	68	2	145	50.7	5.60	24.2	122	74	277	92	185.00	5.60	1	1.00	1	0	1	0	1	1	1	1	0	0	1
1	1	65	1	164	54.5	2.90	20.3	144	89	186	58	128.00	5.30	0	1.00	0	1	1	0	1	1	1	1	0	1	1
1	1	84	1	157	65.2	3.20	26.4	130	69	163	48	115.00	5.90	1	1.00	1	0	1	0	0	0	1	0	1	1	1
1	1	77	1	156	45.1	.60	18.6	130	73	150	72	78.00	6.30	1	1.00	0	0	1	0	1	1	1	1	1	1	1
0	0	44	1	176	91.1	-2.70	29.4	111	74	196	41	155.00	5.50	0	1.00	0	0	1	0	1	1	1	0	1	1	1
1	1	71	2	157	59.8	.70	24.2	155	90	238	55	183.00	5.50	1	1.00	1	0	1	1	1	1	1	1	0	0	1
1	1	71	2	150	62.7	.20	28.0	127	63	235	53	182.00	5.70	1	1.00	1	1	1	1	1	1	1	0	0	0	1
1	1	78	1	159	63.5	-2.80	25.0	152	83	179	65	114.00	6.80	0	1.00	0	0	1	1	1	1	1	0	0	1	0
1	1	70	2	153	43.5	-.70	18.6	137	75	231	65	166.00	5.60	1	1.00	1	0	1	0	1	0	0	1	1	0	1
1	1	63	2	152	56.8	1.10	24.7	116	59	150	44	106.00	5.80	1	1.00	1	0	1	0	1	0	1	1	0	1	1

0	0	78	2	149	48.8	1.90	21.8	127	74	168	78	90.00	5.30	1	1.00	1	0	1	1	1	0	1	1	1	1	1
1	1	71	2	153	54.8	1.40	23.5	121	75	236	67	169.00	6.80	1	1.00	1	0	1	1	1	1	0	1	1	0	0
0	0	62	1	170	64.6	3.70	22.2	108	65	186	72	114.00	5.20	1	1.00	0	1	1	1	1	1	1	1	1	1	1
0	0	70	2	155	68.2	1.30	28.5	157	77	221	73	148.00	5.40	1	1.00	1	0	1	1	1	0	1	0	0	0	1
0	0	74	1	156	49.5	-1.40	20.2	130	69	164	97	67.00	6.60	1	1.00	1	0	1	0	1	1	0	1	0	1	0
1	1	54	1	166	66.6	-1.60	24.1	126	79	178	73	105.00	5.00	1	1.00	0	0	1	1	1	0	0	1	0	1	1
1	1	64	2	153	41.3	-1.00	17.7	100	59	215	62	153.00	5.80	1	1.00	1	0	1	1	1	1	0	1	1	0	1
0	0	63	1	152	60.6	-2.20	26.3	129	64	224	54	170.00	5.90	1	1.00	1	0	1	0	0	1	1	0	1	0	1
1	1	68	2	152	48.8	.00	21.2	149	85	211	59	152.00	5.90	1	1.00	1	1	1	0	1	1	1	1	0	1	1
1	1	78	2	153	57.4	-3.10	24.6	121	66	189	68	121.00	5.00	1	1.00	1	0	1	1	1	1	1	1	0	1	1
1	1	64	1	169	65.6	.20	23.0	112	81	183	54	129.00	5.40	1	1.00	0	1	1	0	1	1	1	1	1	1	1
1	1	43	2	158	45.4	.30	18.3	100	65	162	65	97.00	5.20	1	1.00	1	0	1	0	1	0	1	1	1	1	1
1	1	73	2	152	57.2	.30	24.6	132	81	200	64	136.00	5.30	1	1.00	1	0	1	1	1	1	1	1	1	1	1
0	0	69	2	154	54.7	.40	23.1	120	74	193	84	109.00	5.90	1	1.00	1	0	1	0	1	0	0	1	1	1	1
1	1	77	1	171	79.7	2.10	27.3	127	71	188	43	145.00	5.40	1	1.00	0	0	1	0	1	1	1	0	0	1	1
0	0	69	1	164	57.1	3.10	21.3	123	67	195	44	151.00	5.30	1	1.00	0	0	1	0	1	1	1	1	1	1	1
1	1	62	2	151	45.7	-.90	20.0	131	68	217	67	150.00	6.90	1	1.00	1	1	1	1	1	0	0	1	0	1	0
0	0	42	1	172	65.1	1.60	21.9	113	67	216	65	151.00	5.30	1	1.00	0	0	1	0	0	0	1	1	1	1	1
0	0	59	2	149	53.8	.10	24.3	116	70	185	68	117.00	5.90	1	1.00	1	0	1	1	1	1	0	1	1	1	1
0	0	81	2	142	51.4	.00	25.3	150	77	182	57	125.00	5.40	1	1.00	1	0	1	0	1	0	1	0	0	1	1
1	1	62	1	153	52.4	-3.10	22.5	161	88	195	71	124.00	6.00	1	1.00	0	1	1	1	1	1	1	1	0	1	1
1	1	75	2	155	58.3	-.80	24.3	169	68	167	51	116.00	5.40	1	1.00	1	0	1	1	1	1	1	1	0	1	1
0	0	73	1	158	58.9	.70	23.7	109	57	178	70	108.00	5.80	1	1.00	0	0	1	0	0	0	0	1	0	1	0
1	1	42	2	163	44.4	.40	16.7	90	57	187	85	102.00	5.20	1	1.00	1	1	1	1	1	1	1	1	1	1	1
1	1	70	1	164	59.5	.60	22.1	140	78	147	55	92.00	5.50	1	1.00	0	0	1	1	1	1	1	1	0	1	1
1	1	82	1	157	64.9	1.10	26.3	125	70	183	64	119.00	6.00	1	1.00	0	1	1	1	1	0	1	0	1	1	1
1	1	62	2	149	41.2	1.60	18.5	103	59	168	72	96.00	5.80	1	1.00	1	0	1	1	0	1	0	1	1	1	1
1	1	69	1	163	77.9	.20	29.3	133	77	210	55	155.00	6.70	0	1.00	1	1	1	1	1	1	1	0	0	1	0
1	1	80	2	138	50.4	-.60	26.3	157	85	213	43	170.00	5.90	1	1.00	1	0	1	1	1	1	1	0	0	1	1
1	1	78	2	151	48.4	-.10	21.1	153	82	179	56	123.00	5.50	1	1.00	1	0	1	0	1	0	1	1	0	1	1
1	1	63	2	144	64.5	-2.80	31.3	124	76	196	58	138.00	5.30	1	1.00	1	0	1	0	1	0	1	0	1	0	1
1	1	41	2	153	51.8	1.00	22.2	127	79	214	58	156.00	5.60	1	1.00	1	1	1	1	1	1	1	1	1	1	1
1	1	84	1	157	64.5	-3.70	26.0	127	72	157	41	116.00	6.00	1	1.00	1	0	1	0	1	1	1	0	0	1	1
1	1	90	2	139	38.2	.60	19.7	140	83	205	63	142.00	5.80	1	1.00	1	0	1	1	1	0	0	1	0	1	1
1	1	75	2	145	45.2	-1.20	21.3	129	73	233	62	171.00	5.20	1	1.00	1	0	1	1	1	0	1	1	1	0	1
1	1	59	2	157	62.0	-2.00	25.3	121	65	247	70	177.00	5.50	1	1.00	1	0	1	0	1	1	1	0	1	0	1
1	1	81	2	152	54.1	.40	23.5	134	75	216	34	182.00	5.50	1	1.00	0	0	1	1	1	1	1	1	0	0	1
1	1	70	2	141	44.9	.10	22.6	112	77	222	52	170.00	5.60	1	1.00	1	0	1	1	1	1	1	1	1	0	1
0	0	73	2	148	47.2	-.90	21.6	134	70	179	36	143.00	6.30	1	1.00	1	0	1	1	1	1	1	1	0	0	0

1	1	70	1	157	60.1	.30	24.2	160	86	263	63	200.00	5.80	1	1.00	1	1	1	1	1	1	1	1	0	0	1
1	1	70	1	159	51.5	-2.10	20.2	122	71	161	45	116.00	5.90	1	1.00	1	0	1	1	1	0	1	1	0	1	1
1	1	81	2	148	43.0	-.20	19.6	140	72	232	83	149.00	6.00	1	1.00	0	1	1	1	1	1	1	1	0	0	1
1	1	71	1	163	58.5	.40	22.0	104	71	200	82	118.00	5.70	1	1.00	0	1	1	0	1	1	0	1	1	1	1
1	1	62	2	152	52.5	-2.50	22.8	110	65	168	65	103.00	6.00	1	1.00	1	0	1	1	1	0	0	1	1	1	1
1	1	61	2	149	59.2	-.60	26.7	128	86	195	56	139.00	6.20	1	1.00	1	1	1	0	0	0	1	0	0	0	1
1	1	56	2	151	65.6	-.60	28.7	165	96	237	53	184.00	5.60	1	1.00	1	0	1	0	1	0	1	0	0	0	1
1	1	76	2	149	43.9	.70	19.7	118	77	201	55	146.00	5.70	1	1.00	1	1	1	1	1	0	1	1	1	1	1
1	1	76	1	161	66.8	-1.50	25.8	129	65	224	101	123.00	8.00	1	1.00	0	1	1	1	1	1	1	0	0	0	0
1	1	69	1	154	48.1	-1.60	20.2	125	73	187	62	125.00	5.00	1	1.00	0	1	1	0	1	0	1	1	1	1	1
1	1	59	1	166	58.6	-1.40	21.1	153	95	202	73	129.00	5.20	1	1.00	0	1	1	0	1	0	0	1	0	1	1
0	0	71	2	153	59.2	-.40	25.2	128	82	207	56	151.00	5.50	1	1.00	1	0	1	1	1	0	1	0	1	1	1
0	0	62	1	162	73.2	1.20	27.9	119	70	216	50	166.00	5.70	0	1.00	0	0	1	0	0	1	1	0	0	1	1
0	0	68	2	151	57.4	-3.00	25.2	113	74	182	59	123.00	5.80	1	1.00	0	0	1	0	1	0	0	0	0	1	1
0	0	80	1	152	52.4	2.00	22.6	95	66	202	65	137.00	5.50	1	1.00	1	0	1	0	1	1	0	1	1	1	1
0	0	74	2	143	50.3	-1.00	24.6	116	71	195	72	123.00	5.70	1	1.00	1	0	1	1	1	1	1	1	1	0	1
0	0	88	2	140	34.4	-1.80	17.5	163	79	188	73	115.00	5.50	1	1.00	1	0	1	1	1	1	1	1	0	1	1
0	1	57	2	149	56.3	-1.40	25.3	157	82	221	58	163.00	5.40	1	1.00	1	0	1	0	0	0	0	0	0	0	1
1	1	61	2	151	35.5	.80	15.5	88	56	252	109	143.00	5.60	1	1.00	1	0	1	1	1	1	1	1	1	0	1
1	1	79	2	147	40.6	1.30	18.8	131	81	253	77	176.00	5.60	1	1.00	1	1	1	1	1	1	1	1	0	0	1
0	0	67	2	149	49.9	1.40	22.5	146	81	229	45	184.00	5.30	1	1.00	1	0	1	0	1	0	0	1	0	0	1
1	1	69	1	154	52.8	-.10	22.2	121	74	294	70	224.00	5.50	1	1.00	0	0	1	1	1	1	1	1	1	0	1
0	0	84	2	138	44.5	-1.20	23.2	132	82	252	48	204.00	5.40	1	1.00	1	0	1	1	1	0	0	1	1	0	1
1	1	30	2	160	73.2	1.90	28.6	132	74	211	61	150.00	5.60	1	1.00	1	0	0	0	1	0	0	0	1	1	1
1	1	45	2	161	79.8	8.40	30.7	137	72	205	69	136.00	5.80	1	1.00	1	0	1	1	1	0	0	0	1	1	1
1	1	62	2	155	54.6	-5.30	22.7	129	69	251	72	179.00	5.50	1	1.00	1	1	1	0	1	1	0	1	1	0	1
0	0	49	2	154	48.5	-2.50	20.4	125	80	207	76	131.00	5.50	1	1.00	1	0	1	0	0	0	1	1	1	1	1
1	1	24	2	152	59.0	1.00	25.5	105	57	187	57	130.00	5.10	1	1.00	0	1	1	0	0	1	1	0	1	1	1
0	0	50	2	167	59.3	-1.20	21.1	104	56	214	87	127.00	5.80	1	1.00	0	0	1	0	1	0	0	1	1	1	1
1	1	26	1	174	56.8	.30	18.7	95	66	128	55	73.00	5.10	0	1.00	1	1	1	0	1	1	1	1	1	1	1
0	0	69	1	161	65.5	1.20	25.3	149	82	190	79	111.00	9.10	1	1.00	0	0	1	0	1	0	0	0	0	1	0
0	1	46	1	173	76.5	-1.50	25.5	107	67	205	52	153.00	5.60	1	1.00	1	0	1	0	1	1	0	0	1	1	1
1	1	68	1	155	50.3	-.30	21.0	128	80	208	68	140.00	5.40	1	1.00	1	0	1	1	1	1	1	1	1	1	1
0	0	71	1	160	62.5	-2.00	24.3	138	86	185	45	140.00	5.60	1	1.00	1	1	1	0	1	1	1	1	1	1	1
1	1	62	1	171	83.7	-2.60	28.4	140	87	210	35	175.00	5.80	0	1.00	0	0	1	1	1	1	0	0	0	0	1
1	1	67	1	165	62.5	2.50	23.0	123	69	156	49	107.00	5.50	1	1.00	0	1	1	1	1	1	1	1	1	1	1
1	1	79	1	161	66.8	-2.60	25.7	161	62	176	45	131.00	6.00	1	1.00	1	0	1	1	1	1	1	0	0	1	1
1	1	80	2	154	59.8	-1.10	25.3	145	78	162	47	115.00	5.60	1	1.00	1	0	1	1	1	1	1	0	0	0	1
1	1	81	1	144	52.1	-.20	25.1	117	71	218	48	170.00	5.50	0	2.00	0	0	1	1	1	1	1	0	1	1	1

0	0	72	1	161	63.1	4.30	24.4	152	85	224	81	143.00	5.20	1	1.00	1	0	1	0	1	1	1	1	0	0	1
1	1	61	2	154	55.9	4.30	23.5	141	79	216	71	145.00	6.20	1	1.00	1	1	1	0	0	0	0	1	0	0	1
1	1	71	2	155	55.3	-3.30	23.0	136	70	188	81	107.00	7.20	1	1.00	1	0	1	1	1	1	1	1	1	0	0
0	0	70	2	147	54.7	2.30	25.4	127	77	156	36	120.00	5.20	1	1.00	1	0	1	1	1	1	1	0	1	0	1
0	0	61	2	149	62.8	4.20	28.2	116	69	187	47	140.00	5.60	1	1.00	1	0	1	1	1	1	1	0	0	1	1
0	1	60	2	162	44.3	-2.00	16.8	123	79	244	64	180.00	5.30	1	1.00	1	0	1	0	1	1	1	1	0	0	1
1	1	68	2	156	53.5	.10	22.0	108	70	215	101	114.00	5.70	1	1.00	1	0	1	1	1	0	1	1	1	0	1
0	1	79	2	139	39.5	2.20	20.5	129	68	171	54	117.00	4.90	1	1.00	1	0	1	0	1	1	1	1	0	1	1
1	1	71	1	158	59.6	-.70	23.9	160	84	205	58	147.00	5.40	0	1.00	0	1	1	0	0	0	1	1	0	1	1
1	1	77	1	155	62.9	-.10	26.1	145	69	218	67	151.00	7.00	1	1.00	0	0	1	0	1	1	1	0	0	1	0
1	1	68	2	158	72.3	-.10	28.8	136	86	241	56	185.00	6.00	1	1.00	1	1	1	1	1	1	0	0	0	0	1
1	1	46	2	161	39.0	-.60	15.0	107	66	196	121	75.00	5.60	1	1.00	1	1	1	1	0	1	1	1	1	1	1
1	1	69	1	157	54.2	-2.50	22.0	130	67	195	93	102.00	6.10	1	1.00	0	1	1	0	1	1	1	1	0	1	1
1	1	40	2	159	49.2	-1.00	19.4	93	65	213	98	115.00	5.20	1	1.00	0	0	1	1	1	1	1	1	1	1	1
1	1	89	1	161	65.4	1.40	25.2	103	57	239	58	181.00	5.40	1	1.00	1	0	1	1	1	1	1	0	0	0	1
0	0	45	2	150	52.0	1.30	23.0	110	73	181	80	101.00	4.80	1	1.00	0	1	1	0	1	1	0	1	1	1	1
1	1	73	2	147	46.2	-.30	21.5	126	72	189	42	147.00	5.50	1	1.00	1	1	1	0	1	1	1	1	0	1	1
0	0	68	1	163	65.8	3.50	24.8	132	77	168	89	79.00	6.20	0	2.00	0	0	1	0	1	0	1	1	1	1	1
1	1	67	1	162	72.2	2.50	27.5	163	97	222	51	171.00	5.60	0	2.00	0	0	1	1	1	1	1	0	0	0	1
1	1	86	2	137	36.7	-.80	19.6	133	76	201	78	123.00	4.90	1	1.00	1	1	1	0	1	1	1	1	0	0	1
1	1	49	2	160	71.7	.80	28.0	137	82	226	45	181.00	5.50	1	1.00	1	0	1	0	1	1	0	0	1	0	1
0	0	57	2	151	42.2	-.20	18.6	113	70	199	82	117.00	5.90	1	1.00	1	1	1	0	1	1	0	1	1	1	1
0	0	42	2	159	52.8	-1.40	20.9	102	63	204	104	100.00	4.70	0	2.00	1	0	1	0	0	0	1	1	1	1	1
0	0	50	1	174	66.7	4.80	21.9	125	76	216	73	143.00	5.20	1	1.00	1	1	1	0	1	1	1	1	1	1	1
1	1	75	1	156	58.2	3.90	23.7	165	90	219	79	140.00	5.90	1	1.00	1	1	1	0	0	0	1	1	0	0	1
1	1	52	2	161	52.4	-1.70	20.2	128	71	216	90	126.00	5.70	1	1.00	1	1	1	1	1	1	1	1	1	1	1
1	1	58	2	153	54.8	3.10	23.4	125	68	255	87	168.00	5.90	1	1.00	1	0	1	1	0	1	1	1	1	0	1
1	1	75	1	161	65.7	-3.20	25.4	150	87	149	65	84.00	5.50	1	1.00	0	0	1	1	1	0	1	0	0	1	1
0	0	72	2	148	46.8	-1.50	21.3	138	73	168	49	119.00	6.60	1	1.00	1	1	1	0	1	0	0	1	0	1	0
1	1	68	2	154	51.8	-1.70	21.8	139	86	202	66	136.00	6.50	1	1.00	1	0	1	0	1	0	1	1	0	1	0
1	1	79	2	162	79.2	1.60	30.3	129	76	210	72	138.00	5.40	1	1.00	1	0	0	1	1	1	1	0	0	1	1
1	1	64	2	152	62.7	.10	26.9	137	86	165	64	101.00	5.20	1	1.00	1	0	1	0	0	1	0	0	0	0	1
1	1	69	1	163	58.9	-.40	22.0	119	74	249	70	179.00	5.90	0	1.00	0	1	1	0	1	0	1	1	1	0	1
0	0	80	1	146	68.4	-.70	31.9	115	66	235	41	194.00	5.40	1	1.00	1	0	1	0	1	1	1	0	1	0	1
0	0	58	1	168	76.1	-7.80	26.9	97	75	247	68	179.00	5.80	1	1.00	1	0	1	1	1	1	1	0	1	0	1
1	1	76	1	163	63.0	-.10	23.7	121	63	225	52	173.00	6.00	0	1.00	1	1	1	1	1	1	0	1	1	0	1
0	0	48	1	176	85.5	-5.50	27.7	113	61	233	59	174.00	5.30	0	1.00	1	1	1	0	1	1	1	0	1	0	1
1	1	56	2	158	53.9	-1.20	21.7	143	82	258	68	190.00	5.40	1	1.00	1	0	1	1	0	1	1	1	0	0	1
0	0	59	2	151	77.0	2.20	33.9	140	84	165	56	109.00	5.80	1	1.00	1	1	1	0	1	0	0	0	0	1	1

0	0	75	2	155	48.1	2.10	20.0	142	84	199	47	152.00	5.50	1	1.00	1	0	1	1	1	0	1	1	0	1	1
0	0	67	2	156	59.2	-.90	24.3	107	62	240	50	190.00	5.80	1	1.00	1	1	1	0	1	0	0	1	1	0	1
0	0	64	2	145	57.0	1.30	27.0	118	71	109	27	82.00	5.60	1	1.00	1	1	1	0	1	0	1	0	1	0	1
1	1	71	2	143	39.6	.60	19.2	112	61	221	72	149.00	5.60	1	1.00	1	0	1	1	1	0	0	1	1	0	1
1	1	61	2	154	66.4	2.10	27.8	109	60	283	73	210.00	5.50	1	1.00	1	1	1	1	1	1	1	0	1	0	1
1	1	78	1	158	63.4	.50	25.3	134	83	213	60	153.00	5.30	1	1.00	1	1	1	1	1	1	1	0	0	1	1
0	0	81	1	152	53.1	.50	23.1	128	74	200	78	122.00	5.80	1	1.00	0	1	1	1	1	1	1	1	0	1	1
1	1	79	2	150	53.8	-.80	23.9	119	77	161	55	106.00	5.70	1	1.00	1	1	1	1	1	1	1	1	1	1	1
0	0	66	2	148	54.1	1.30	24.7	123	69	254	73	181.00	5.30	1	1.00	1	0	1	0	1	1	0	1	1	0	1
1	1	63	2	156	55.5	1.50	22.9	102	54	245	66	179.00	5.60	1	1.00	1	0	1	0	1	1	0	1	1	0	1
0	0	77	1	154	67.4	3.20	28.4	160	107	197	55	142.00	5.80	1	1.00	1	0	1	1	1	1	1	0	0	1	1
1	1	38	2	158	48.8	-.10	19.4	110	75	188	66	122.00	4.80	1	1.00	1	1	1	0	1	0	1	1	1	1	1
1	1	77	2	149	51.4	.70	23.0	99	55	237	88	149.00	5.60	1	1.00	1	0	1	0	1	1	1	1	1	0	1
1	1	63	2	148	43.9	1.40	20.1	110	70	205	80	125.00	5.40	1	1.00	1	0	1	0	1	0	0	1	1	1	1
1	1	71	2	150	57.5	-1.80	25.5	125	74	232	73	159.00	5.30	1	1.00	1	0	1	1	1	0	0	0	1	0	1
1	1	50	2	159	56.4	1.60	22.1	117	76	218	76	142.00	5.70	1	1.00	1	1	1	1	1	1	1	1	1	1	1
1	1	54	2	152	56.6	-.30	24.4	123	79	185	54	131.00	5.60	1	1.00	1	0	1	1	1	1	1	1	1	1	1
0	0	73	2	147	60.9	2.60	28.1	121	72	221	52	169.00	5.10	1	1.00	1	0	1	1	1	1	1	0	0	0	1
1	1	75	1	168	52.1	-.20	18.5	118	68	166	83	83.00	5.10	0	1.00	0	0	1	0	1	0	0	1	1	1	1
1	1	63	1	164	56.5	.80	21.0	145	85	199	68	131.00	7.30	0	1.00	0	1	1	1	0	1	0	1	0	1	0
1	1	72	1	164	58.3	.90	21.7	121	68	175	100	75.00	5.20	1	1.00	0	1	1	1	1	1	1	1	0	1	1
1	1	83	2	152	45.8	-.90	19.8	150	56	191	84	107.00	5.50	1	1.00	1	0	1	1	1	1	1	1	0	1	1
1	1	63	1	164	79.0	2.30	29.2	132	73	161	41	120.00	5.40	1	1.00	1	0	1	0	1	1	1	0	0	1	1
0	0	57	1	173	76.0	-.90	25.2	121	77	240	62	178.00	5.30	1	1.00	1	1	1	1	1	1	1	0	1	0	1
1	1	44	2	153	47.5	-1.60	20.2	107	64	170	74	96.00	5.10	1	1.00	1	0	1	0	1	0	1	1	1	1	1
1	1	62	2	149	71.0	-2.50	32.0	129	82	264	52	212.00	6.30	1	1.00	1	1	1	1	1	0	0	0	1	0	1
1	1	69	2	151	47.1	1.30	20.6	136	90	213	50	163.00	5.90	1	1.00	1	0	1	0	1	1	1	1	0	1	1
1	1	71	2	155	61.4	-1.20	25.4	141	76	151	72	79.00	5.00	1	1.00	1	1	1	1	1	0	1	0	0	1	1
0	1	82	1	158	52.4	.40	21.0	131	70	155	46	109.00	5.30	0	1.00	0	1	1	1	1	1	1	1	1	1	1
1	1	60	1	169	66.8	-.10	23.5	163	83	234	67	167.00	6.40	1	1.00	0	0	0	1	1	1	1	1	0	0	0
1	1	57	2	158	46.9	-1.00	18.7	100	60	265	106	159.00	5.20	1	1.00	1	0	0	0	1	0	1	1	1	0	1
1	1	67	2	153	50.5	.80	21.6	146	82	260	93	167.00	5.40	1	1.00	1	1	1	0	1	0	0	1	0	0	1
1	1	80	2	139	41.5	.00	21.4	127	69	210	64	146.00	5.70	1	1.00	1	0	1	0	1	0	0	1	0	1	1
1	1	64	1	175	72.5	3.80	23.7	133	78	161	53	108.00	7.80	1	1.00	0	0	1	1	0	0	1	1	1	0	0
0	1	68	1	162	59.9	.20	22.8	122	79	143	58	85.00	5.40	1	1.00	0	0	1	0	1	1	1	1	1	1	1
1	1	44	2	161	57.1	-.20	22.0	136	92	160	80	80.00	5.10	1	1.00	0	0	1	1	1	0	0	1	0	1	1
1	1	62	2	154	59.3	1.70	25.1	109	66	215	54	161.00	5.70	1	1.00	1	1	1	0	1	0	0	0	1	1	1
0	0	44	2	160	65.9	2.20	25.8	142	91	228	58	170.00	5.40	1	1.00	1	0	1	0	0	0	0	0	0	0	1
1	1	54	2	145	67.9	-4.80	32.2	146	93	298	108	190.00	6.10	1	1.00	1	0	1	1	1	1	1	0	0	0	1

1	0	84	1	153	47.8	-.90	20.4	106	64	208	77	131.00	6.10	1	1.00	0	0	1	1	1	0	0	1	1	1	1
1	1	75	1	164	67.8	-4.40	25.1	119	65	200	55	145.00	5.40	1	1.00	1	0	1	1	1	0	0	0	0	1	1
1	1	80	1	169	69.6	-1.80	24.4	112	58	105	46	59.00	5.50	1	1.00	1	0	1	1	1	1	1	1	0	1	1
1	1	77	1	160	76.9	-1.90	29.9	157	83	188	56	132.00	6.60	1	1.00	1	0	1	1	1	1	1	0	0	1	0
0	0	58	2	158	69.0	2.80	27.7	175	109	175	59	116.00	5.20	1	1.00	1	1	1	1	0	0	0	0	0	1	1
0	0	35	2	154	52.7	-3.30	22.1	106	63	177	59	118.00	4.90	1	1.00	0	0	1	0	1	1	1	1	1	1	1
0	0	61	2	158	55.5	.70	22.1	114	64	175	51	124.00	5.50	1	1.00	1	1	1	0	1	1	1	1	1	1	1
1	1	62	2	148	49.6	-1.30	22.6	138	85	217	64	153.00	5.30	1	1.00	1	0	1	1	1	1	0	1	1	1	1
0	0	57	2	147	46.3	.90	21.3	122	78	213	64	149.00	5.10	1	1.00	1	0	1	1	1	1	1	1	1	1	1
1	1	65	1	166	66.6	-3.30	24.2	130	73	168	46	122.00	6.80	0	1.00	0	0	1	0	0	0	1	1	0	1	0
0	0	63	1	165	84.0	-3.60	30.7	123	88	245	51	194.00	5.80	1	1.00	0	0	1	1	1	1	1	0	0	0	1
0	0	50	2	150	53.3	1.10	23.8	135	86	177	56	121.00	5.70	1	1.00	1	0	1	0	1	0	0	1	1	1	1
0	0	63	2	145	51.1	-.60	24.3	111	70	214	76	138.00	6.10	1	1.00	1	0	1	1	1	0	1	1	1	1	1
1	1	69	1	164	64.9	-2.80	24.1	127	69	157	76	81.00	5.10	0	1.00	0	1	1	0	1	0	1	1	0	1	1
0	0	33	1	163	53.9	-2.10	20.2	100	56	143	56	87.00	5.20	0	2.00	1	0	1	0	1	0	1	1	1	1	1
1	1	42	1	168	85.4	1.20	30.2	121	74	184	44	140.00	5.30	0	1.00	1	0	1	0	1	0	1	0	1	1	1
1	1	72	2	152	47.4	-3.20	20.5	146	77	227	47	180.00	5.60	1	1.00	1	0	1	0	0	0	0	1	0	0	1
0	0	75	1	172	74.2	-.20	24.9	144	72	165	69	96.00	5.40	1	1.00	0	0	1	1	0	1	1	1	0	1	1
0	0	52	1	167	66.7	-4.40	23.8	130	79	180	46	134.00	5.20	1	1.00	1	0	1	0	0	1	1	1	1	1	1
0	0	48	1	166	60.6	-2.10	22.1	137	74	190	92	98.00	5.20	1	1.00	0	0	1	1	0	0	1	1	1	1	1
0	0	51	1	164	70.8	-.90	26.3	145	99	217	42	175.00	5.50	1	1.00	1	0	1	0	1	0	1	0	0	1	1
0	0	75	2	152	57.7	2.50	24.8	133	68	191	66	125.00	5.60	1	1.00	1	0	1	1	1	1	1	1	0	1	1
1	1	41	2	155	60.8	-3.90	25.4	153	78	209	62	147.00	6.60	1	1.00	1	0	1	0	1	0	0	0	0	1	0
0	0	39	1	174	64.4	.00	21.3	113	71	180	69	111.00	5.40	0	1.00	0	0	0	0	0	0	0	1	1	1	1
1	1	71	2	145	50.2	-3.00	23.7	123	69	185	78	107.00	5.40	1	1.00	1	0	1	1	1	1	1	1	1	0	1
1	1	63	2	152	60.9	1.30	26.3	147	86	175	104	71.00	6.10	1	1.00	0	0	1	0	1	1	0	0	0	1	0
1	1	65	2	145	40.5	2.20	19.1	127	69	198	64	134.00	5.90	1	1.00	1	0	1	1	1	1	1	1	1	1	1
1	1	69	2	153	63.3	-2.30	27.0	111	73	217	73	144.00	5.30	1	1.00	1	0	1	1	1	1	1	0	1	1	1
1	1	82	1	155	59.0	-1.50	24.5	127	77	193	67	126.00	6.30	0	1.00	0	0	1	0	0	1	1	1	0	1	1
1	1	55	2	154	43.8	-.90	18.5	157	77	214	78	136.00	6.00	1	1.00	1	0	1	1	1	1	0	1	0	1	1
0	0	58	2	154	55.8	.80	23.5	116	75	233	71	162.00	5.30	1	1.00	1	0	1	1	0	0	0	1	1	0	1
1	1	28	2	151	43.2	.50	19.0	112	63	225	92	133.00	4.80	1	1.00	1	0	1	0	1	1	1	1	1	0	1
1	1	48	1	163	70.4	2.90	26.6	153	109	203	77	126.00	5.60	1	1.00	0	0	1	0	1	1	0	0	0	1	1
0	0	81	2	145	49.0	-1.20	23.3	149	89	131	57	74.00	6.20	1	1.00	1	0	1	1	1	1	1	1	0	0	1
0	0	58	1	172	67.5	-1.10	22.7	102	64	226	52	174.00	5.50	1	1.00	1	0	0	0	1	1	1	1	1	0	1
0	0	58	2	154	51.7	-2.70	21.7	134	75	251	76	175.00	5.70	1	1.00	1	0	1	0	0	1	1	1	1	0	1
0	0	68	1	162	54.9	-.60	21.0	159	103	198	119	79.00	5.20	1	1.00	0	0	1	1	0	0	1	1	0	1	1
1	1	66	2	146	45.2	-1.10	21.1	98	65	208	65	143.00	5.80	0	1.00	0	1	1	1	1	1	1	1	1	1	1
1	1	67	1	155	60.8	-.20	25.1	122	87	231	57	174.00	5.50	1	1.00	0	0	1	1	1	0	1	0	1	0	1

1	1	74	1	169	67.9	-2.20	23.8	158	81	166	81	85.00	5.20	1	1.00	0	0	1	1	1	1	1	1	0	1	1
1	1	77	1	164	70.3	-1.90	26.1	137	66	178	57	121.00	5.60	1	1.00	0	0	1	1	1	1	0	0	0	1	1
1	0	74	1	174	61.0	1.00	20.2	113	69	167	71	96.00	6.00	1	1.00	0	0	1	1	1	1	0	1	0	1	1
1	1	65	2	156	66.2	-2.60	27.1	137	79	186	52	134.00	5.80	1	1.00	1	0	1	1	1	1	1	0	1	1	1
1	1	60	2	157	42.0	-.30	16.9	146	76	229	100	129.00	5.60	1	1.00	1	1	1	1	1	1	1	1	0	0	1
1	1	71	1	170	78.4	-3.20	27.0	120	84	191	55	136.00	7.10	1	1.00	1	0	1	0	0	0	1	0	1	1	0
1	1	62	2	156	51.2	-1.10	21.1	138	84	250	62	188.00	5.40	1	1.00	1	0	1	1	0	1	0	1	0	0	1
1	1	70	1	168	70.2	-3.10	24.9	150	84	190	62	128.00	5.30	0	1.00	0	0	1	1	1	1	1	1	0	1	1
0	0	40	2	158	48.7	-.20	19.4	112	54	172	71	101.00	4.90	0	1.00	1	0	1	1	1	1	1	1	1	1	1
1	1	66	2	149	32.7	.10	14.6	152	84	199	92	107.00	5.10	1	1.00	1	0	1	1	1	0	0	1	0	1	1
0	0	88	1	157	60.1	-2.60	24.2	133	68	160	48	112.00	5.40	1	1.00	1	1	1	1	1	1	1	1	1	1	1
1	1	66	2	145	48.3	-2.20	23.0	137	78	269	98	171.00	5.80	1	1.00	1	0	1	1	1	0	0	1	1	0	1
1	1	68	2	150	51.2	-1.00	22.8	113	73	163	38	125.00	6.30	1	1.00	1	0	1	0	1	0	0	1	1	0	1
0	0	74	2	154	59.7	-2.70	25.3	112	64	186	37	149.00	5.20	1	1.00	1	0	1	0	1	1	1	0	1	0	1
1	1	81	2	156	48.1	2.00	19.8	124	71	200	96	104.00	5.70	1	1.00	1	1	1	1	1	1	1	1	1	1	1
1	1	48	1	166	55.9	-1.90	20.3	111	74	203	57	146.00	5.40	0	1.00	0	0	1	1	1	1	1	1	1	1	1
0	0	47	2	161	48.4	-.60	18.6	136	77	234	82	152.00	6.50	1	1.00	1	0	1	0	1	1	1	1	1	0	0
1	1	35	2	159	44.4	1.20	17.4	98	61	195	93	102.00	5.40	0	2.00	1	1	1	1	1	1	1	1	1	1	1
1	1	43	2	165	57.4	2.50	21.1	118	79	241	99	142.00	5.10	1	1.00	0	0	1	1	1	0	0	1	1	0	1
0	0	36	2	151	45.1	2.50	19.9	95	55	192	71	121.00	5.40	1	1.00	1	0	1	0	1	1	1	1	1	1	1
1	1	62	1	166	63.0	-1.40	22.9	140	81	188	71	117.00	5.30	1	1.00	0	0	1	0	1	1	1	1	0	1	1
0	0	53	1	171	59.6	-.80	20.3	127	83	225	70	155.00	5.50	0	1.00	0	1	1	0	1	0	1	1	1	0	1
1	1	69	2	158	61.6	.50	24.7	148	88	220	54	166.00	6.00	1	1.00	1	0	1	1	1	1	1	1	0	0	1
1	1	62	2	151	58.8	-1.80	25.9	158	83	235	61	174.00	9.40	1	1.00	1	0	1	0	1	1	0	0	0	0	0
1	1	53	2	156	71.6	1.50	29.5	130	75	188	61	127.00	6.00	0	1.00	1	1	1	1	1	0	0	0	0	1	1
1	1	63	2	149	48.9	-2.50	22.1	151	80	246	58	188.00	5.60	1	1.00	1	1	1	0	1	1	1	1	0	0	1
0	0	74	2	148	52.6	3.50	24.0	131	75	232	69	163.00	5.60	1	1.00	1	0	1	0	0	0	0	1	0	0	1
1	1	51	2	151	50.3	.70	22.1	123	83	247	50	197.00	5.50	1	1.00	0	0	1	0	1	0	1	1	1	0	1
1	1	56	1	170	74.9	-2.70	25.9	125	86	191	73	118.00	5.20	1	1.00	0	1	1	0	1	1	1	0	1	1	1
0	0	56	2	152	47.9	.20	20.8	114	60	308	114	194.00	5.50	1	1.00	1	0	1	0	0	1	1	1	1	0	1
1	1	46	2	161	52.3	-6.70	20.1	180	92	334	75	259.00	5.50	1	1.00	1	0	1	1	1	0	1	1	0	0	1
0	0	64	2	151	52.3	.90	22.9	101	66	179	50	129.00	5.70	1	1.00	0	0	1	1	1	0	1	1	1	1	1
0	0	67	1	162	59.5	.10	22.6	146	84	183	54	129.00	6.30	1	1.00	1	1	1	0	1	0	1	1	0	1	0
1	1	65	1	163	57.0	1.60	21.5	128	71	204	77	127.00	5.70	1	1.00	0	0	1	1	1	1	1	1	1	1	1
0	0	64	2	152	57.5	5.40	24.9	128	74	290	63	227.00	5.50	1	1.00	1	0	1	0	1	0	0	1	1	0	1
1	1	77	2	144	46.2	.20	22.1	182	93	236	104	132.00	5.20	1	1.00	1	0	1	1	1	0	0	1	0	0	1
0	0	73	1	163	61.3	.60	23.0	112	70	176	58	118.00	4.80	1	1.00	1	1	1	0	1	1	1	1	1	1	1
1	1	75	1	162	66.1	-2.60	25.2	119	80	206	58	148.00	5.60	0	1.00	0	0	1	0	1	0	1	0	1	1	1
1	1	72	2	153	47.1	2.60	20.1	108	59	228	88	140.00	5.90	1	1.00	1	0	1	1	1	1	0	1	0	0	1

0	0	51	2	171	66.0	.50	22.5	112	72	209	88	121.00	5.70	1	1.00	1	0	1	0	1	0	0	1	1	1	1
1	1	78	2	139	50.9	-2.10	26.3	148	81	184	75	109.00	7.30	1	1.00	1	0	1	1	1	1	1	0	0	1	0
1	1	76	2	143	46.8	.40	22.8	154	97	205	56	149.00	5.40	1	1.00	1	0	1	1	1	0	1	1	0	1	1
1	1	64	1	161	63.3	1.70	24.4	182	98	195	55	140.00	6.20	0	1.00	0	1	1	1	1	1	1	1	0	1	1
1	1	76	2	142	62.8	-4.40	31.0	132	76	159	53	106.00	6.70	1	1.00	1	0	1	0	1	1	0	0	0	1	0
1	1	43	2	157	53.5	-4.10	21.7	104	63	194	122	72.00	4.70	1	1.00	0	0	1	1	1	0	0	1	1	1	1
1	1	77	1	160	54.3	1.20	21.3	116	76	176	57	119.00	5.30	1	1.00	0	0	1	1	1	1	1	1	0	1	1
0	0	81	1	160	48.8	.10	18.9	137	77	199	65	134.00	6.10	0	1.00	0	0	1	0	1	1	1	1	1	1	1
0	0	59	2	156	51.6	1.30	21.1	149	93	264	71	193.00	5.80	1	1.00	0	1	1	0	1	1	1	1	0	0	1
1	1	62	2	160	57.2	-5.00	22.3	119	71	270	57	213.00	5.40	1	1.00	1	0	1	1	1	0	0	1	1	0	1
1	1	54	2	167	66.0	.50	23.7	103	71	246	65	181.00	5.60	1	1.00	1	1	1	0	1	1	1	1	1	0	1
1	1	63	2	154	58.6	1.70	24.6	125	74	197	44	153.00	6.50	1	1.00	1	1	1	1	1	0	1	1	0	1	0
1	1	67	1	158	53.6	1.70	21.4	132	74	243	72	171.00	6.10	0	1.00	0	0	1	0	0	1	1	1	0	0	1
1	1	69	2	153	49.5	.10	21.0	124	68	283	64	219.00	5.80	1	1.00	1	1	1	1	1	1	1	1	1	0	1
1	1	41	2	155	50.1	-2.00	20.7	102	63	188	107	81.00	5.70	1	1.00	1	1	1	0	1	0	1	1	1	1	1
1	1	76	2	158	60.4	-.70	24.1	149	83	222	43	179.00	5.40	1	1.00	1	0	1	1	0	0	0	1	0	0	1
0	0	74	1	163	63.6	1.10	23.8	112	73	196	39	157.00	5.90	1	1.00	1	0	1	0	1	0	0	1	0	0	1
1	1	73	2	142	52.9	-1.60	26.1	111	64	207	58	149.00	5.20	1	1.00	1	0	1	1	1	0	0	0	0	1	1
0	0	70	2	153	46.7	-.20	19.8	135	76	235	59	176.00	5.50	1	1.00	1	0	1	1	1	1	1	1	0	0	1
0	0	52	2	152	59.8	4.70	25.8	112	68	181	63	118.00	5.50	1	1.00	1	0	1	1	1	0	0	0	1	1	1
0	0	71	1	162	58.2	11.00	22.0	119	66	193	59	134.00	5.80	1	1.00	1	0	1	0	1	0	0	1	0	1	1
1	1	69	2	148	53.0	1.80	24.1	117	61	215	86	129.00	5.70	1	1.00	1	1	1	1	1	1	1	1	1	1	1
1	1	79	2	138	43.7	-.80	22.9	141	72	266	68	198.00	5.60	1	1.00	1	0	1	1	1	0	1	1	0	0	1
1	1	60	2	155	50.3	.70	20.9	128	79	171	41	130.00	5.60	1	1.00	1	0	1	0	1	0	0	1	0	1	1
1	1	61	2	152	47.1	2.00	20.4	128	67	223	73	150.00	5.60	1	1.00	1	0	1	1	1	1	0	1	1	0	1
1	1	71	2	150	50.1	-.30	22.4	117	63	239	62	177.00	5.90	1	1.00	1	0	1	0	1	1	1	1	1	0	1
0	0	64	2	153	82.3	-5.60	35.2	144	82	174	75	99.00	5.20	1	1.00	1	0	1	0	0	1	1	0	0	0	1
1	1	58	2	150	68.3	-5.70	30.4	127	83	247	58	189.00	5.90	1	1.00	1	0	1	0	1	1	1	0	1	0	1
1	1	57	2	158	58.0	.50	23.1	121	73	240	54	186.00	5.70	1	1.00	1	0	1	1	1	0	1	1	1	0	1
1	1	73	2	158	50.5	.70	20.1	105	64	158	70	88.00	5.90	1	1.00	1	1	1	1	1	1	1	1	0	1	1
0	0	64	2	146	42.4	3.80	19.9	105	75	245	85	160.00	6.30	1	1.00	1	1	1	0	1	1	0	1	0	0	1
1	1	66	2	154	58.7	-.30	24.7	126	73	187	71	116.00	5.70	1	1.00	1	0	1	0	1	1	0	1	1	1	1
0	0	66	2	150	47.4	3.90	21.1	153	84	212	73	139.00	5.60	1	1.00	1	0	1	0	0	1	0	1	0	1	1
1	1	71	2	140	54.2	.70	27.6	126	72	286	60	226.00	6.40	1	1.00	1	1	1	1	1	1	1	0	0	0	0
0	0	75	2	150	46.1	-.50	20.3	96	57	156	37	119.00	5.90	1	1.00	1	0	1	0	1	1	1	1	1	0	1
1	1	69	1	157	56.9	-1.50	23.2	122	72	161	102	59.00	5.50	0	1.00	0	1	1	1	1	1	1	1	1	1	1
1	1	59	2	147	61.0	2.40	28.4	98	67	253	63	190.00	5.50	1	1.00	0	1	1	1	1	1	1	0	1	0	1
1	1	51	2	155	57.1	1.00	23.7	129	72	185	69	116.00	5.40	0	1.00	1	0	0	0	1	0	1	1	1	1	1
1	1	84	1	164	66.6	-.80	24.7	135	67	178	63	115.00	5.30	0	1.00	0	0	1	1	1	1	1	1	0	1	1

1	1	60	1	168	72.3	-.90	25.7	111	76	246	45	201.00	5.70	1	1.00	1	1	1	0	1	1	1	0	1	0	1
1	1	73	2	151	57.9	.80	25.3	141	88	176	53	123.00	5.60	1	1.00	1	0	1	0	1	1	0	0	0	0	1
0	0	56	2	160	61.6	4.70	23.9	156	111	263	56	207.00	5.60	1	1.00	1	0	1	1	0	0	1	1	0	0	1
0	0	82	1	159	72.6	4.10	28.7	138	76	228	67	161.00	7.00	1	1.00	0	0	1	1	1	1	1	0	0	0	0
1	1	63	2	152	55.5	1.20	23.9	123	71	225	64	161.00	5.50	1	1.00	1	0	1	0	1	0	1	1	0	0	1
0	0	74	1	165	59.8	2.70	22.0	160	91	193	65	128.00	5.70	0	1.00	1	0	1	0	1	0	1	1	0	1	1
1	1	85	1	155	41.5	-2.20	17.2	159	83	171	41	130.00	5.90	1	1.00	1	0	1	0	1	1	1	1	0	1	1
1	1	75	1	167	70.5	.70	25.4	122	72	240	43	197.00	5.20	1	1.00	0	0	1	1	1	1	1	0	1	0	1
1	1	70	2	156	48.9	-2.20	20.0	107	68	199	78	121.00	5.10	1	1.00	1	1	1	0	1	1	1	1	1	1	1
1	1	70	2	147	54.7	-.30	25.4	169	95	251	52	199.00	5.50	1	1.00	1	0	1	0	1	0	1	0	0	0	1
1	1	49	2	154	56.5	.10	23.7	97	56	142	40	102.00	5.40	1	1.00	1	1	1	0	1	0	0	1	1	1	1
1	1	44	2	160	46.5	-.60	18.2	119	83	200	84	116.00	5.00	1	1.00	0	0	0	0	0	0	0	1	1	1	1
1	1	78	2	148	41.0	-.40	18.8	119	71	174	41	133.00	5.40	1	1.00	1	0	1	1	1	0	0	1	1	1	1
1	1	57	1	170	77.5	1.10	26.8	131	85	231	42	189.00	6.40	1	1.00	0	0	0	0	1	0	0	0	1	0	1
1	1	69	2	153	51.2	2.40	22.0	120	77	223	75	148.00	5.80	1	1.00	1	1	1	0	1	0	1	1	0	0	1
1	1	77	2	147	49.3	3.80	22.7	132	81	280	79	201.00	5.80	1	1.00	1	1	1	1	1	1	1	1	1	0	1
1	1	78	2	154	61.7	-.90	26.0	162	89	171	32	139.00	5.80	1	1.00	1	0	1	1	1	1	1	0	0	0	1
1	1	78	2	146	45.2	.00	21.2	117	67	250	89	161.00	5.30	1	1.00	1	0	1	1	1	0	0	1	1	0	1
0	0	63	2	150	55.4	2.80	24.7	156	98	259	49	210.00	5.50	1	1.00	1	0	1	1	1	0	0	1	0	0	1
1	1	49	2	146	44.2	.50	20.7	131	82	239	78	161.00	6.10	1	1.00	1	1	1	1	1	1	1	1	1	0	1
1	1	75	1	150	54.9	.50	24.4	135	77	141	62	79.00	5.00	1	1.00	0	0	1	0	1	0	0	1	0	1	1
1	1	58	1	159	51.3	-2.50	20.3	134	84	228	82	146.00	5.70	1	1.00	0	0	1	0	1	1	1	1	1	0	1
0	0	75	2	142	46.6	.60	22.9	129	73	200	44	156.00	5.60	1	1.00	1	1	1	0	0	1	1	1	1	1	1
1	1	87	2	143	43.1	.90	21.0	145	69	140	68	72.00	5.80	1	1.00	1	1	1	0	1	1	0	1	0	1	1
1	1	40	2	162	52.8	2.10	20.0	99	67	195	83	112.00	5.20	1	1.00	1	0	1	1	1	1	1	1	1	1	1
1	1	63	2	150	46.8	.10	20.8	112	64	225	77	148.00	5.60	1	1.00	1	0	1	1	1	1	1	1	1	0	1
1	1	60	2	143	48.7	1.70	23.7	120	63	200	60	140.00	6.00	1	1.00	1	0	1	0	1	1	0	1	1	1	1
1	1	72	1	166	72.7	-.20	26.5	130	75	192	47	145.00	5.30	1	1.00	0	0	1	0	1	1	1	0	0	1	1
1	1	46	2	163	63.8	-.10	24.0	138	83	220	48	172.00	6.00	1	1.00	1	0	1	0	1	0	1	1	1	0	1
1	1	79	2	155	51.0	.80	21.2	139	82	150	76	74.00	6.10	1	1.00	1	0	1	1	1	1	0	1	1	1	1
0	0	62	2	148	47.9	-1.10	21.8	113	73	211	56	155.00	5.70	1	1.00	1	0	1	0	0	1	1	1	0	1	1
0	1	73	1	159	66.3	-4.90	26.1	120	73	215	66	149.00	5.60	1	1.00	0	0	1	0	0	1	1	0	0	1	1
1	1	69	1	153	52.2	1.60	22.1	173	75	203	49	154.00	7.00	0	1.00	0	1	1	0	1	1	1	1	0	1	0
1	1	73	2	148	53.1	-1.30	24.4	135	72	186	63	123.00	5.90	1	1.00	1	0	1	1	1	1	1	1	1	1	1
1	1	75	2	146	47.3	1.50	22.0	119	63	194	37	157.00	5.90	1	1.00	1	0	1	1	1	1	1	1	1	0	1
0	0	80	2	147	49.0	.80	22.6	146	71	213	54	159.00	5.70	1	1.00	1	0	1	1	1	1	1	1	0	1	1
1	1	79	2	144	46.8	-1.40	22.7	105	58	134	50	84.00	6.10	1	1.00	1	0	1	1	1	0	0	1	0	1	1
1	1	76	2	152	56.2	-3.00	24.4	126	69	203	46	157.00	7.90	1	1.00	1	0	1	1	1	0	0	1	0	1	0
1	1	75	2	145	48.9	.60	23.2	126	66	182	49	133.00	5.20	1	1.00	1	0	1	1	1	1	1	1	0	1	1

1	1	61	1	170	71.3	-1.40	24.5	132	81	166	43	123.00	8.40	0	1.00	0	0	1	0	1	0	1	1	1	1	0
1	1	55	2	145	49.8	-.70	23.5	141	90	254	49	205.00	5.50	1	1.00	1	0	1	0	0	0	0	1	0	0	1
1	1	68	2	149	47.7	-1.20	21.4	111	61	178	78	100.00	3.60	1	1.00	1	0	1	1	1	0	0	1	1	1	1
1	1	52	1	169	71.2	1.30	24.8	134	87	222	90	132.00	5.60	1	1.00	0	1	1	1	1	0	1	1	1	0	1
1	1	62	2	151	62.6	-1.50	27.6	131	74	188	63	125.00	6.60	1	1.00	1	1	1	0	0	0	1	0	0	1	0
1	1	78	2	145	58.0	-2.00	27.4	125	56	192	92	100.00	5.60	1	1.00	1	0	1	0	1	1	0	0	1	1	1
0	0	62	1	171	78.4	-2.50	26.7	138	88	209	73	136.00	5.50	1	1.00	0	1	1	0	0	1	0	0	0	1	1
1	1	29	2	155	41.9	-.60	17.5	117	73	162	101	61.00	5.60	0	1.00	0	0	0	0	1	1	1	1	1	1	1
1	1	66	1	165	64.8	1.40	23.7	132	70	223	42	181.00	5.70	1	1.00	0	0	1	1	1	1	1	1	1	0	1
1	1	60	2	149	57.8	-2.30	25.9	156	88	196	61	135.00	5.70	1	1.00	1	0	1	0	1	0	0	0	0	1	1
1	1	79	2	142	56.8	-.20	28.2	137	78	230	48	182.00	5.90	1	1.00	1	0	1	1	1	0	1	0	0	0	1
1	1	64	2	145	49.9	3.10	23.7	109	58	200	51	149.00	5.50	1	1.00	1	1	1	1	1	1	1	1	1	1	1
0	0	62	2	155	51.7	1.20	21.6	110	65	211	51	160.00	6.90	1	1.00	1	0	1	0	0	0	1	1	1	1	0
0	0	68	2	150	66.8	4.40	29.7	149	105	184	88	96.00	5.20	0	2.00	0	0	1	1	1	1	1	0	0	1	1
1	1	57	2	163	66.8	-.80	25.2	126	75	252	59	193.00	6.30	1	1.00	1	0	1	0	0	0	0	0	1	0	1
0	0	71	1	161	65.4	2.40	25.1	94	59	222	51	171.00	5.90	0	2.00	0	0	1	1	1	1	1	0	0	0	1
0	0	81	2	148	42.1	1.10	19.3	129	74	235	69	166.00	6.80	1	1.00	1	0	1	1	1	0	0	1	0	0	0
1	1	59	2	156	50.6	.80	20.9	103	53	170	44	126.00	5.70	1	1.00	1	0	1	1	1	1	1	1	1	1	1
1	1	60	2	143	41.4	1.20	20.1	127	70	234	59	175.00	5.60	1	1.00	1	0	1	1	1	1	1	1	1	0	1
1	1	73	1	158	72.6	-.30	28.9	125	76	174	43	131.00	5.50	1	1.00	0	1	1	1	1	1	1	0	0	1	1
1	1	48	2	138	53.9	4.70	28.1	144	85	149	50	99.00	4.90	1	1.00	1	0	1	0	1	1	1	0	0	1	0
1	1	67	1	163	78.7	.50	29.7	142	79	171	65	106.00	6.60	1	1.00	0	0	1	1	1	0	1	0	0	1	0
0	0	68	2	158	57.8	.60	23.0	101	62	241	42	199.00	6.20	1	1.00	1	1	1	1	1	1	1	1	1	0	1
1	1	74	1	164	62.9	.40	23.3	145	89	222	64	158.00	5.60	1	1.00	0	1	1	0	1	1	1	1	0	0	1
1	1	60	1	161	65.1	.10	25.0	114	69	149	52	97.00	6.90	1	1.00	1	0	1	0	1	1	1	0	0	1	0
1	1	77	1	171	65.2	-1.80	22.2	138	73	194	59	135.00	5.50	1	1.00	0	1	1	1	1	1	1	1	1	1	1
0	0	51	2	158	67.9	-.60	27.0	115	74	321	65	256.00	5.60	1	1.00	1	1	1	1	1	1	1	0	1	0	1
0	0	69	1	171	63.2	-1.80	21.7	112	68	200	72	128.00	5.30	1	1.00	0	1	1	1	1	1	1	1	1	1	1
1	1	49	2	159	62.2	1.20	24.7	133	84	207	82	125.00	5.30	1	1.00	0	1	1	0	0	1	1	1	1	1	1
0	0	74	1	161	61.2	.20	23.6	124	73	244	52	192.00	5.80	1	1.00	1	0	1	1	1	1	1	1	0	0	1
0	0	76	2	145	40.3	1.70	19.1	104	64	260	94	166.00	5.90	1	1.00	1	0	1	0	0	0	0	1	0	0	1
0	0	64	2	157	48.1	2.40	19.6	143	80	198	54	144.00	5.60	1	1.00	1	0	1	1	1	0	1	1	0	1	1
1	1	68	2	154	48.7	-1.20	20.6	133	68	169	53	116.00	5.70	1	1.00	1	0	1	1	1	1	1	1	1	1	1
1	1	77	1	161	55.2	-.50	21.1	120	63	194	58	136.00	5.40	1	1.00	1	1	1	1	1	1	1	1	1	1	1
1	1	26	1	161	60.6	4.30	23.4	120	57	173	39	134.00	5.60	0	1.00	1	0	1	1	1	0	1	1	1	0	1
1	1	77	2	143	39.9	1.60	19.4	170	94	230	93	137.00	5.60	1	1.00	1	0	1	1	1	1	1	1	0	0	1
1	1	82	1	143	51.0	3.00	24.8	123	66	268	57	211.00	7.30	1	1.00	0	1	1	0	1	0	1	1	0	0	0
1	1	58	2	151	54.5	-3.60	23.9	119	65	192	41	151.00	5.90	1	1.00	1	0	1	0	1	1	1	1	0	1	1
1	1	69	2	153	59.7	-.30	25.5	174	103	190	70	120.00	5.90	1	1.00	1	0	1	1	1	1	1	0	0	1	1

1	1	56	1	170	72.2	-1.20	24.8	126	83	206	38	168.00	6.90	1	1.00	0	1	1	0	1	1	1	1	1	0	0
1	1	83	1	159	71.6	-1.40	28.5	156	68	185	40	145.00	6.20	1	1.00	0	0	1	1	1	1	0	0	0	1	1
1	1	58	1	160	61.0	.10	23.8	144	84	199	83	116.00	6.00	1	1.00	1	0	1	0	1	0	1	1	0	1	1
0	0	75	1	163	55.0	.20	20.7	146	76	198	54	144.00	6.70	1	1.00	1	0	1	0	1	1	1	1	0	0	0
1	1	58	2	157	57.9	-1.30	23.6	135	77	202	48	154.00	5.70	1	1.00	1	1	1	1	1	0	0	1	0	1	1
0	0	69	1	159	64.9	.40	25.7	128	76	225	43	182.00	5.20	1	1.00	0	0	1	0	1	0	1	0	0	0	1
1	1	75	2	145	43.6	-.80	20.7	123	66	220	54	166.00	5.50	1	1.00	1	0	1	1	1	1	1	1	0	0	1
1	1	74	2	144	49.7	-.30	23.9	117	63	223	61	162.00	5.70	1	1.00	1	0	1	1	1	1	1	1	0	0	1
1	1	78	1	166	68.7	-1.20	25.0	125	74	188	43	145.00	6.40	0	1.00	1	1	1	1	1	1	1	0	1	1	1
1	1	66	2	150	49.1	.40	21.8	132	74	200	58	142.00	5.80	1	1.00	1	0	1	0	0	0	0	1	1	1	1
1	1	61	2	150	49.3	.50	21.8	121	72	223	52	171.00	6.20	1	1.00	1	1	1	1	1	1	1	1	1	0	1
0	0	63	1	165	76.8	-1.20	28.0	156	102	238	60	178.00	5.60	1	1.00	1	0	1	0	1	1	0	0	0	0	1
1	1	71	1	155	58.6	-.70	24.2	115	63	165	80	85.00	6.80	1	1.00	0	1	1	1	1	1	1	1	0	1	0
1	1	70	2	151	45.2	.90	19.8	114	66	250	77	173.00	5.80	1	1.00	1	0	1	1	1	0	1	1	1	0	1
1	1	79	1	159	61.3	-1.20	24.3	124	76	192	53	139.00	7.00	1	1.00	1	1	1	1	1	1	1	1	0	1	0
1	1	38	2	138	44.1	.90	23.0	101	56	208	69	139.00	5.10	1	1.00	1	0	1	0	0	0	0	1	1	1	1
1	1	76	1	164	68.4	-.40	25.4	152	83	219	65	154.00	5.70	1	1.00	0	0	1	1	1	0	1	0	0	1	1
1	1	67	1	166	68.9	3.50	24.9	124	83	234	71	163.00	5.60	1	1.00	0	0	1	1	1	1	1	1	1	0	1
1	1	52	2	152	53.7	.20	23.3	131	74	240	85	155.00	5.40	1	1.00	1	0	1	0	1	0	1	1	1	0	1
1	1	68	2	149	47.7	.10	21.5	136	66	247	79	168.00	5.80	1	1.00	1	0	1	1	1	0	1	1	1	0	1
1	1	63	1	167	64.7	.20	23.0	158	79	291	78	213.00	5.90	1	1.00	0	0	1	1	1	1	1	1	0	0	1
0	0	61	2	155	53.8	4.20	22.4	135	77	245	92	153.00	5.30	0	2.00	0	0	1	0	0	1	1	1	0	0	1
1	1	72	2	151	64.9	-1.10	28.3	159	84	213	46	167.00	5.80	1	1.00	1	1	1	1	1	0	1	0	0	1	1
0	0	57	1	163	64.5	-.70	24.3	150	84	192	46	146.00	5.40	1	1.00	0	0	1	1	1	1	1	1	0	1	1
0	0	73	2	146	66.9	3.80	31.2	165	104	228	53	175.00	5.60	1	1.00	1	0	1	0	1	1	0	0	0	0	1
1	1	76	2	148	46.7	-.60	21.3	144	78	210	33	177.00	5.90	1	1.00	1	0	1	1	0	0	0	1	0	0	1
1	1	72	2	149	61.2	-12.50	27.5	143	90	200	45	155.00	5.80	1	1.00	1	1	1	1	1	0	0	0	0	1	1
1	1	34	1	163	84.3	1.90	31.8	109	64	200	35	165.00	5.90	0	1.00	1	0	0	0	1	0	1	0	1	0	1
0	0	37	2	170	74.5	-6.50	25.6	104	65	204	57	147.00	5.20	1	1.00	1	1	1	1	1	1	1	0	1	1	1
0	0	62	2	156	51.6	-1.30	21.2	115	72	218	82	136.00	5.40	1	1.00	1	1	1	0	1	0	1	1	0	1	1
1	1	51	2	162	65.6	.80	25.0	110	70	193	63	130.00	5.40	1	1.00	0	0	1	1	1	0	1	0	1	1	1
0	0	46	1	170	65.4	2.40	22.6	164	99	190	51	139.00	5.80	1	1.00	0	0	1	1	1	1	1	1	0	1	1
0	0	21	2	157	49.2	-2.00	19.8	106	57	224	78	146.00	5.20	1	1.00	1	0	0	0	1	0	0	1	1	0	1
1	1	62	2	145	38.4	.20	18.1	90	52	192	73	119.00	5.60	1	1.00	0	0	1	0	0	0	0	1	1	1	1
0	0	76	2	152	55.1	1.10	23.8	166	95	199	53	146.00	6.60	1	1.00	1	1	1	0	1	0	1	1	0	1	0
0	1	70	2	146	47.7	-.10	22.4	161	98	191	71	120.00	5.30	1	1.00	1	0	1	0	1	0	0	1	0	1	1
0	0	69	1	160	52.9	2.40	20.5	126	75	203	53	150.00	5.70	0	2.00	1	0	1	0	1	0	1	1	1	1	0
1	1	72	1	154	52.8	-1.30	22.1	143	74	138	64	74.00	5.40	1	1.00	0	1	1	0	1	0	1	1	0	1	1
1	1	53	2	151	50.5	-.80	22.2	136	85	226	75	151.00	5.80	1	1.00	0	0	1	1	1	0	0	1	1	0	1

0	0	57	2	152	61.1	1.40	26.3	127	85	217	56	161.00	5.50	1	1.00	0	0	1	0	1	1	1	0	0	1	1
1	1	65	1	172	59.9	-3.30	20.1	135	89	214	60	154.00	5.60	1	1.00	1	1	1	1	1	1	1	1	1	1	1
0	0	63	2	152	59.3	-3.50	25.5	116	65	176	35	141.00	5.60	1	1.00	1	0	0	0	0	0	0	0	1	0	1
0	0	57	2	149	54.9	1.40	24.7	158	89	224	44	180.00	5.80	1	1.00	1	0	1	0	0	0	0	1	0	0	1
0	0	77	1	158	63.8	2.60	25.6	120	63	131	43	88.00	5.80	1	1.00	1	1	1	1	1	0	1	0	1	1	1
1	1	73	2	153	50.3	-.30	21.4	151	92	238	51	187.00	6.20	1	1.00	1	0	1	1	1	1	1	1	0	0	1
0	1	27	2	163	48.0	.80	18.1	103	58	146	64	82.00	5.30	0	1.00	1	0	1	0	1	1	1	1	1	1	1
1	1	71	2	144	52.6	2.80	25.2	116	66	175	48	127.00	5.70	1	1.00	1	1	1	0	1	0	1	0	1	1	1
1	1	35	1	171	80.4	-5.70	27.4	109	59	231	32	199.00	5.40	0	1.00	0	0	1	0	1	1	1	0	1	0	1
1	1	22	2	160	57.3	-2.60	22.4	98	57	236	76	160.00	5.60	1	1.00	0	0	1	1	1	1	1	1	1	0	1
0	0	72	2	151	79.3	-3.40	34.7	129	62	182	52	130.00	5.90	1	1.00	1	1	1	1	1	0	1	0	0	0	1
1	1	26	2	156	52.0	-2.20	21.5	97	60	225	67	158.00	5.10	1	1.00	1	1	1	0	0	1	1	1	1	0	1
1	1	74	2	160	51.7	2.90	20.3	152	91	186	71	115.00	6.20	1	1.00	1	0	1	1	1	0	0	1	0	1	0
1	1	67	2	148	58.4	-1.30	26.5	129	75	224	86	138.00	5.40	1	1.00	1	1	1	1	1	1	1	0	1	0	1
1	1	54	2	153	49.2	.20	20.9	122	67	212	72	140.00	5.70	1	1.00	1	1	1	0	1	0	0	1	1	1	1
1	1	78	1	169	70.9	-.40	24.8	118	77	191	50	141.00	5.40	1	1.00	0	1	1	1	1	0	0	1	1	1	1
0	1	74	1	160	55.4	2.50	21.6	113	53	177	61	116.00	5.70	1	1.00	0	0	1	1	0	0	1	1	0	0	1
1	1	69	1	167	63.5	-2.30	22.8	104	71	206	65	141.00	5.30	1	1.00	0	1	1	1	1	1	1	1	1	1	1
1	1	68	1	157	47.0	.60	19.0	133	78	172	77	95.00	5.60	1	1.00	0	0	1	1	1	1	1	1	0	1	1
0	0	57	2	157	42.1	1.30	16.9	100	65	220	53	167.00	5.60	1	1.00	1	0	0	0	0	0	0	1	1	0	1
0	0	67	2	156	54.6	-.90	22.4	150	94	200	74	126.00	5.80	1	1.00	1	0	1	0	0	1	0	1	0	1	1
0	0	68	1	166	55.5	-3.90	20.1	126	75	178	59	119.00	5.50	1	1.00	0	0	1	0	1	0	0	1	1	1	1
1	1	78	1	156	49.8	-.90	20.4	132	65	199	99	100.00	5.40	1	1.00	1	1	1	1	1	1	1	1	1	1	1
0	0	76	2	156	53.1	-1.10	21.9	157	82	186	50	136.00	5.50	1	1.00	1	0	1	1	1	1	1	1	0	1	1
1	1	58	2	156	48.3	1.60	19.8	124	88	245	62	183.00	5.40	1	1.00	0	1	0	0	1	0	0	1	1	0	1
0	0	65	1	166	59.3	-.10	21.6	97	56	177	80	97.00	5.30	1	1.00	0	0	1	0	0	0	0	1	1	1	1
1	1	69	2	158	49.2	.40	19.6	126	82	212	93	119.00	5.50	1	1.00	1	1	1	0	1	1	1	1	1	0	1
1	1	70	1	167	56.7	1.20	20.3	123	68	146	74	72.00	5.20	1	1.00	1	1	1	1	1	1	1	1	1	1	1
0	1	69	2	149	45.8	2.60	20.5	134	74	188	74	114.00	5.60	1	1.00	1	1	1	0	1	0	0	1	1	1	1
1	1	76	2	150	69.5	1.70	30.7	140	90	214	44	170.00	6.00	1	1.00	1	0	1	0	1	1	1	0	0	1	1
0	0	57	1	167	67.3	4.20	24.0	125	76	176	45	131.00	5.50	1	1.00	0	1	1	0	1	0	0	1	0	1	1
1	1	74	2	144	50.4	-.90	24.1	136	79	228	40	188.00	6.00	1	1.00	1	0	1	1	1	1	1	1	1	0	1
1	1	61	2	147	56.4	1.10	25.9	134	83	274	49	225.00	5.70	1	1.00	1	0	1	0	1	1	1	0	1	0	1
0	0	43	2	160	71.4	2.00	27.8	121	67	190	49	141.00	5.60	0	1.00	1	0	1	0	0	1	0	0	1	1	1
1	1	67	2	150	43.5	-4.00	19.2	114	68	209	55	154.00	6.00	1	1.00	1	0	1	0	1	1	1	1	1	1	1
1	1	73	2	159	52.4	-.50	20.8	144	79	207	54	153.00	5.40	1	1.00	1	0	1	1	1	1	1	1	0	1	1
1	1	70	2	145	50.6	.60	24.1	120	69	192	76	116.00	5.50	1	1.00	1	0	1	1	1	1	1	1	0	0	1
1	1	72	2	153	63.0	.80	27.0	105	57	234	53	181.00	5.40	1	1.00	1	0	1	1	1	1	1	0	1	0	1
1	1	65	2	138	58.2	-1.40	30.3	152	84	161	42	119.00	5.30	1	1.00	0	0	1	1	1	1	1	0	0	0	1

1	1	59	2	146	41.1	-.80	19.2	167	94	182	61	121.00	5.10	1	1.00	1	0	1	0	0	0	1	1	0	1	1
1	1	45	1	162	68.8	-.10	26.2	160	105	234	48	186.00	5.40	1	1.00	1	0	1	0	1	0	1	0	0	0	1
1	1	67	2	155	61.0	.70	25.5	135	75	217	85	132.00	7.30	1	1.00	1	0	1	0	1	1	1	0	1	1	0
0	0	55	2	150	45.3	-.20	20.2	113	71	168	66	102.00	5.30	1	1.00	1	0	1	1	1	0	0	1	1	1	1
0	0	44	2	157	55.8	-1.20	22.6	121	84	196	69	127.00	6.30	1	1.00	1	0	1	0	1	1	0	1	1	1	0
1	1	71	2	147	49.8	-1.30	23.1	130	68	177	53	124.00	5.70	1	1.00	1	0	1	0	1	0	0	1	0	1	1
1	1	73	1	167	68.2	.80	24.3	109	68	198	39	159.00	7.00	1	1.00	1	0	1	0	0	1	0	1	1	0	0
1	1	76	2	145	53.8	.30	25.4	110	68	172	76	96.00	5.50	1	1.00	1	1	1	1	1	0	1	0	1	1	1
1	1	68	2	148	63.0	1.80	28.7	120	69	177	61	116.00	5.70	0	1.00	1	0	1	0	0	0	0	0	0	0	1
0	0	62	1	173	69.7	.20	23.1	130	85	182	77	105.00	4.90	1	1.00	0	1	1	0	1	0	0	1	1	1	1
1	1	63	2	148	77.0	-.70	35.2	131	80	205	49	156.00	6.70	1	1.00	1	0	1	0	1	1	1	0	1	1	0
1	1	76	2	148	63.4	4.40	28.8	132	89	322	75	247.00	6.00	1	1.00	0	0	1	1	1	0	0	0	0	0	1
1	1	86	2	147	54.3	.00	24.9	158	67	207	48	159.00	5.60	1	1.00	1	0	1	1	1	1	1	1	0	0	1
1	1	58	2	153	52.8	.00	22.6	152	86	263	80	183.00	5.60	1	1.00	0	1	1	1	1	0	1	1	0	0	1
0	0	59	1	163	77.9	-2.20	29.3	140	86	183	62	121.00	5.60	0	1.00	0	1	1	0	0	0	1	0	0	1	1
0	0	69	2	146	50.0	1.10	23.5	143	82	196	66	130.00	5.60	1	1.00	1	0	1	0	1	0	0	1	0	1	1
0	0	49	1	158	43.5	1.80	17.5	103	59	156	57	99.00	5.20	1	1.00	1	0	1	0	0	0	0	1	1	1	1
0	0	53	1	182	74.9	.10	22.6	114	74	170	47	123.00	5.40	1	1.00	0	0	1	0	0	1	1	1	1	1	1
0	0	28	2	157	46.0	.10	18.7	105	69	219	70	149.00	5.20	1	1.00	1	1	1	0	1	0	1	1	1	1	1
1	1	72	1	166	63.1	-.10	22.9	144	82	219	38	181.00	5.50	0	1.00	0	1	1	1	1	1	1	1	0	0	1
0	0	68	1	163	53.6	.10	20.0	93	61	182	80	102.00	5.30	1	1.00	0	0	1	0	0	0	0	1	1	1	1
0	0	69	2	156	53.4	1.90	21.9	144	81	193	60	133.00	6.00	1	1.00	1	1	1	0	0	1	1	1	0	1	1
0	0	70	2	155	55.9	.60	23.3	119	70	195	47	148.00	5.20	1	1.00	1	0	1	0	0	0	0	1	0	1	1
1	1	65	2	151	44.9	.60	19.6	139	74	171	64	107.00	5.20	1	1.00	1	0	1	1	1	1	1	1	1	1	1
1	1	78	2	143	45.7	.10	22.3	144	92	237	102	135.00	5.50	1	1.00	1	1	1	1	1	1	1	1	0	0	1
0	0	71	2	136	45.8	1.70	24.7	156	71	251	64	187.00	5.40	1	1.00	1	1	1	1	0	1	1	1	0	0	1
0	0	30	1	179	77.6	2.50	24.2	135	70	130	50	80.00	5.10	1	1.00	0	0	1	0	1	1	1	1	1	1	1
1	1	55	1	163	71.9	-1.90	27.0	132	90	151	56	95.00	5.30	0	1.00	0	0	1	0	1	1	1	0	0	1	1
0	0	57	1	163	62.8	-1.80	23.7	131	79	199	47	152.00	6.50	1	1.00	1	0	1	0	1	0	1	1	1	1	0
1	1	77	1	163	59.4	.90	22.3	120	77	166	61	105.00	5.40	1	1.00	1	0	1	1	1	1	1	1	0	1	1
1	1	76	1	157	60.6	-.90	24.6	99	66	209	54	155.00	5.20	1	1.00	0	0	1	0	1	1	1	1	0	1	1
1	1	50	2	154	48.7	-1.50	20.5	128	82	236	84	152.00	5.50	1	1.00	1	0	1	0	0	0	1	1	1	0	1
1	1	59	1	160	63.9	-.40	24.9	159	96	191	45	146.00	5.80	1	1.00	0	1	1	1	1	1	0	1	0	1	1
1	1	68	1	151	51.3	.40	22.4	138	87	159	49	110.00	4.90	1	1.00	0	0	1	1	1	1	1	1	1	0	1
0	0	71	2	151	55.7	-.10	24.5	154	85	216	45	171.00	6.20	1	1.00	1	0	1	1	0	0	0	1	0	1	1
1	1	73	1	158	66.3	.30	26.6	142	87	199	86	113.00	5.30	1	1.00	0	1	1	1	0	0	0	0	0	1	1
1	1	81	2	143	51.0	.60	24.8	144	77	175	58	117.00	6.50	1	1.00	1	1	1	1	1	0	1	1	0	0	0
1	1	34	1	159	58.5	1.90	23.1	124	72	174	61	113.00	5.30	0	1.00	1	0	1	0	0	0	1	1	1	1	1
1	1	63	1	161	59.2	2.90	22.8	125	77	196	47	149.00	5.00	1	1.00	0	1	1	0	0	1	0	1	1	1	1

1	1	67	2	150	55.1	1.70	24.3	137	81	233	54	179.00	5.40	1	1.00	1	1	1	0	1	0	0	1	0	0	1
0	0	50	1	175	73.7	-.10	24.2	109	69	208	57	151.00	5.10	0	1.00	0	0	1	0	1	0	1	1	1	1	1
1	1	61	1	168	63.3	1.90	22.4	124	65	205	52	153.00	5.50	1	1.00	0	0	1	1	1	1	1	1	0	1	1
1	1	76	2	150	51.3	2.30	22.8	115	65	159	57	102.00	5.60	1	1.00	1	0	1	0	1	0	1	1	0	1	0
1	1	66	2	149	54.3	1.70	24.5	151	86	228	53	175.00	5.40	1	1.00	1	0	1	0	1	1	1	1	0	0	1
1	1	67	1	162	67.4	-1.40	25.5	108	65	156	54	102.00	5.70	1	1.00	0	1	1	0	1	0	1	0	1	1	1
1	1	43	1	179	110.1	-3.10	34.4	134	82	199	37	162.00	7.60	1	1.00	1	0	1	0	1	0	0	0	0	0	0
1	1	54	1	168	88.6	.60	31.3	135	82	184	48	136.00	6.30	1	1.00	0	0	1	0	1	1	0	0	0	0	1
1	1	54	2	154	57.5	-1.90	24.1	146	96	190	71	119.00	5.20	1	1.00	1	0	1	0	0	0	1	1	0	0	1
1	1	76	1	168	71.5	-4.00	25.2	214	130	190	43	147.00	5.70	1	1.00	0	1	1	1	1	1	1	0	0	1	1
1	1	49	2	156	44.4	.80	18.3	93	56	154	54	100.00	5.10	0	1.00	1	0	1	1	0	0	0	1	1	1	1
1	1	63	2	156	53.6	.20	22.0	137	79	177	69	108.00	5.70	1	1.00	1	0	1	0	1	0	0	1	0	0	1
0	0	33	2	159	53.9	5.10	21.4	114	67	177	71	106.00	5.00	1	1.00	1	0	1	0	1	0	1	1	1	1	1
0	0	37	2	145	43.4	-.10	20.7	111	71	230	96	134.00	5.70	1	1.00	0	0	1	1	0	0	0	1	1	0	1
1	1	71	1	168	69.5	-2.00	24.5	153	94	158	37	121.00	5.30	1	1.00	0	1	1	0	1	1	1	1	0	0	1
0	0	53	2	161	67.6	-2.30	26.0	136	77	296	78	218.00	5.40	1	1.00	1	0	1	0	0	0	1	0	0	0	1
1	1	70	1	162	62.0	-.80	23.5	109	63	104	45	59.00	5.80	0	1.00	1	0	1	0	1	0	0	1	0	0	1
0	0	67	1	171	72.1	-6.40	24.7	159	95	229	60	169.00	5.40	1	1.00	0	0	1	0	0	1	1	1	0	0	1
1	1	59	1	165	64.7	-1.10	23.8	126	79	230	60	170.00	6.70	0	1.00	0	0	1	1	1	1	1	1	1	0	0
1	1	74	2	149	52.2	-4.80	23.6	141	70	179	75	104.00	6.70	1	1.00	1	0	1	1	1	1	1	1	0	0	0
1	1	60	1	171	74.4	-9.40	25.5	127	86	217	53	164.00	5.20	1	1.00	0	0	0	0	0	0	0	0	1	1	1
1	1	70	2	132	49.6	-.30	28.5	151	72	193	63	130.00	5.40	1	1.00	1	0	1	0	1	1	1	0	0	1	0
1	1	80	2	143	62.2	-1.00	30.5	145	79	220	56	164.00	5.90	1	1.00	1	0	1	1	1	0	1	0	0	0	1
1	1	41	1	162	50.9	-5.00	19.3	140	86	228	64	164.00	5.60	0	1.00	1	0	1	1	1	0	1	1	0	0	1
0	0	69	1	165	68.7	-.80	25.3	119	78	165	64	101.00	6.00	0	1.00	0	0	1	0	1	0	1	0	1	1	1
1	1	44	2	151	41.9	-3.70	18.3	122	79	197	85	112.00	4.80	0	1.00	0	1	1	0	0	1	1	1	1	1	1
1	1	65	2	150	57.0	-1.70	25.4	118	78	209	69	140.00	6.50	1	1.00	1	1	1	0	1	1	1	0	0	1	0
0	0	74	1	167	65.7	-1.70	23.4	134	79	163	33	130.00	6.30	0	1.00	1	0	1	0	1	0	0	1	1	0	1
0	0	72	2	156	55.4	-.40	22.6	139	74	158	52	106.00	6.20	1	1.00	1	0	1	0	0	0	0	1	0	0	1
0	0	44	1	171	92.1	2.00	31.5	120	72	134	47	87.00	5.40	0	1.00	0	0	1	0	1	0	1	0	0	1	1
1	1	33	2	161	52.6	-1.10	20.2	106	65	182	91	91.00	5.00	1	1.00	0	1	1	1	1	0	0	1	1	1	1
1	1	80	1	161	55.8	-.50	21.6	158	97	199	44	155.00	5.30	1	1.00	0	1	1	1	1	1	1	1	0	1	1
0	0	34	2	161	73.0	-1.50	28.0	99	73	186	51	135.00	5.40	0	1.00	1	1	1	1	1	0	0	0	1	1	1
0	0	37	1	163	78.7	1.70	29.4	128	75	199	35	164.00	5.10	1	1.00	1	0	0	0	1	0	0	0	1	0	1
0	0	70	1	165	81.9	-2.60	29.9	129	82	191	29	162.00	5.80	0	1.00	0	0	1	0	1	1	1	0	0	0	1
1	1	63	1	167	63.7	.90	22.9	115	65	198	71	127.00	6.10	1	1.00	0	1	1	1	1	1	1	1	1	1	1
1	1	70	2	149	53.5	1.50	24.2	113	69	207	83	124.00	5.90	1	1.00	1	1	1	0	1	0	1	1	1	1	1
1	1	68	2	144	44.9	-.40	21.5	90	56	202	72	130.00	5.70	1	1.00	1	0	1	0	1	0	0	1	1	1	1
1	1	71	1	159	61.5	-1.90	24.3	134	82	174	48	126.00	5.40	1	1.00	0	0	1	1	1	1	1	1	1	1	1

1	1	75	1	174	78.4	.20	25.8	141	73	187	48	139.00	5.80	1	1.00	1	1	1	1	1	1	1	0	0	1	1
0	0	62	1	153	60.9	1.00	25.8	132	84	183	62	121.00	5.10	0	1.00	0	1	0	0	1	0	1	0	0	1	1
0	0	65	2	155	54.1	2.30	22.6	102	65	178	56	122.00	5.80	1	1.00	1	0	1	0	1	1	1	1	0	1	1
1	1	48	2	165	75.6	-4.80	27.8	118	78	190	73	117.00	5.30	1	1.00	0	1	1	0	0	1	1	0	1	1	1
1	1	30	2	158	57.3	-2.10	22.9	112	61	194	45	149.00	5.50	0	1.00	1	0	1	0	0	1	1	1	1	1	1
1	1	68	1	161	63.4	2.50	24.3	115	70	187	78	109.00	5.90	1	1.00	0	0	1	1	0	1	1	1	1	1	1
0	0	73	2	151	50.8	2.10	22.2	127	81	242	49	193.00	6.70	1	1.00	1	0	1	1	1	1	1	1	1	0	0
1	1	63	2	157	51.0	-1.50	20.7	129	67	227	42	185.00	6.00	1	1.00	1	0	1	0	1	0	0	1	0	0	0
0	0	61	2	148	52.0	-.30	23.9	118	68	226	54	172.00	5.40	1	1.00	1	0	1	1	0	0	0	1	1	0	1
1	1	61	2	150	62.5	.00	27.8	118	69	197	29	168.00	7.70	1	1.00	1	0	1	1	1	0	1	0	1	0	0
1	1	71	2	152	54.0	.30	23.4	126	67	216	64	152.00	6.10	1	1.00	1	0	1	0	0	1	0	1	0	1	1
1	1	62	2	160	61.8	-3.30	24.2	141	81	218	66	152.00	5.80	1	1.00	1	0	1	0	0	0	1	1	0	1	1
0	0	77	2	140	51.1	-2.10	25.9	129	74	194	70	124.00	5.50	1	1.00	1	0	1	1	1	1	0	0	1	1	1
1	1	52	2	161	51.6	-2.30	19.8	89	57	159	65	94.00	6.20	1	1.00	1	0	1	0	1	1	0	1	0	1	0
1	1	74	1	163	67.1	-2.50	25.4	139	68	146	67	79.00	5.80	1	1.00	1	0	1	1	1	0	1	0	1	1	1
1	1	62	2	151	47.8	-.40	20.8	151	82	214	51	163.00	5.60	1	1.00	1	0	1	1	1	1	1	1	0	1	1
1	1	64	1	168	71.2	-2.30	25.2	115	68	218	49	169.00	5.30	1	1.00	0	0	1	0	0	0	1	0	1	1	1
1	1	74	1	158	50.7	-.70	20.3	104	61	164	45	119.00	5.80	1	1.00	1	0	1	1	1	1	1	1	1	0	1
1	1	42	1	173	68.4	-1.40	22.8	117	70	238	88	150.00	5.40	1	1.00	1	0	1	0	1	0	0	1	1	0	1
1	1	68	2	147	39.1	4.80	18.1	100	54	215	65	150.00	5.80	1	1.00	1	0	1	1	1	1	1	1	1	1	1
0	0	80	1	149	53.2	-1.50	23.9	138	71	160	33	127.00	5.50	1	1.00	0	0	1	0	1	0	1	1	0	0	1
1	1	59	2	157	66.2	-1.90	26.9	124	78	180	38	142.00	5.90	1	1.00	1	0	1	1	1	1	1	0	1	0	1
0	0	83	2	147	52.7	2.40	24.5	142	72	165	63	102.00	5.50	1	1.00	1	0	1	1	1	1	1	1	0	0	1
1	1	70	2	148	45.6	-.40	20.9	155	78	195	49	146.00	6.00	1	1.00	1	1	1	0	1	1	1	1	0	0	1
1	1	68	2	145	48.6	-1.90	23.0	199	106	221	89	132.00	5.90	1	1.00	1	0	1	1	1	1	1	1	0	0	1
0	0	67	1	172	71.2	-1.90	24.0	123	74	202	39	163.00	5.70	0	1.00	0	1	0	0	1	1	1	1	1	0	1
0	0	47	1	169	61.6	-3.90	21.5	129	88	233	74	159.00	7.30	0	1.00	0	0	1	0	0	0	1	1	0	0	0
1	1	72	2	152	54.7	-1.60	23.7	110	69	212	72	140.00	5.40	1	1.00	1	0	1	1	1	1	1	1	0	1	1
0	0	81	2	145	42.4	-.30	20.1	120	69	165	67	98.00	5.10	1	1.00	1	1	1	1	1	1	0	1	1	1	1
0	0	61	1	170	55.3	-.40	19.2	148	88	116	83	33.00	5.10	0	1.00	0	0	0	1	1	1	1	1	0	1	1
1	1	44	1	178	81.1	-6.30	25.5	125	86	238	42	196.00	5.80	1	1.00	1	0	0	0	0	1	0	0	1	0	1
0	0	39	2	140	38.5	-2.40	19.5	131	78	153	80	73.00	4.80	0	1.00	0	0	1	0	0	1	1	1	1	1	1
1	1	68	2	158	60.3	-2.00	24.1	147	82	223	46	177.00	5.90	1	1.00	1	1	1	0	1	0	0	1	0	0	1
1	1	51	1	172	87.0	-1.40	29.5	151	92	305	50	255.00	5.60	1	1.00	1	1	1	0	1	0	1	0	0	0	1
0	0	42	2	166	78.6	1.70	28.5	99	66	243	61	182.00	5.60	1	1.00	0	0	1	1	1	1	0	0	1	0	1
0	0	43	1	166	66.5	-2.70	24.1	137	89	266	41	225.00	9.30	0	1.00	0	0	1	1	1	1	1	1	0	0	0
1	1	80	1	155	64.1	-4.20	26.7	138	77	200	65	135.00	5.60	1	1.00	0	1	1	0	1	1	1	0	1	1	1
1	1	68	1	158	68.6	-3.70	27.6	161	87	235	104	131.00	5.50	1	1.00	0	0	1	1	1	1	1	0	0	0	1
0	0	70	1	165	49.0	-.70	18.1	162	86	219	121	98.00	5.20	0	1.00	0	0	0	0	1	1	1	1	0	1	1

1	1	62	2	159	66.8	1.10	26.4	116	65	211	56	155.00	5.40	1	1.00	1	0	1	0	1	1	1	0	1	1	1
1	1	63	1	169	71.4	2.80	25.0	114	77	182	60	122.00	5.40	1	1.00	0	0	1	1	1	1	1	0	1	1	1
0	0	68	2	142	55.2	-1.30	27.3	133	71	213	78	135.00	5.60	1	1.00	1	1	1	1	1	1	1	0	0	1	1
1	1	30	2	162	73.8	-8.80	28.0	123	68	198	40	158.00	5.10	1	1.00	1	0	0	1	1	0	0	0	1	1	1
1	1	26	2	148	34.7	-1.70	15.9	115	60	268	87	181.00	5.00	0	1.00	1	0	0	1	1	0	0	1	1	0	1
0	0	41	2	162	59.5	.00	22.6	107	61	194	78	116.00	5.20	1	1.00	1	0	1	1	1	1	1	1	1	1	1
1	1	70	2	154	49.4	-1.90	20.9	146	80	246	78	168.00	5.60	1	1.00	1	1	1	1	1	0	1	1	0	0	1
0	0	68	2	150	65.9	.10	29.2	125	78	248	84	164.00	6.10	1	1.00	1	1	1	0	0	1	1	0	1	0	1
1	1	68	1	166	62.9	.80	22.8	133	81	236	44	192.00	5.30	1	1.00	0	0	1	1	1	0	1	1	0	0	1
0	0	72	2	146	49.9	1.40	23.5	141	79	236	72	164.00	5.00	1	1.00	1	0	1	0	1	0	0	1	0	0	1
0	0	38	2	157	64.9	-1.20	26.2	137	93	208	67	141.00	5.20	1	1.00	0	0	1	0	1	1	1	0	0	1	1
0	0	66	1	157	49.5	-1.60	20.0	132	81	231	83	148.00	5.40	1	1.00	0	0	1	1	1	0	1	1	1	0	1
1	1	50	1	171	64.4	-.20	21.9	112	75	249	61	188.00	5.40	0	1.00	0	0	1	0	0	0	1	1	1	0	1
0	0	60	2	153	49.2	5.20	20.9	150	81	281	82	199.00	5.30	1	1.00	1	0	1	0	1	0	1	1	0	0	1
0	0	74	1	163	74.1	.70	27.8	135	79	248	39	209.00	7.10	1	1.00	1	1	1	0	1	0	0	0	0	0	0
1	1	67	2	144	43.7	-.40	21.0	149	84	247	64	183.00	5.50	1	1.00	1	1	1	1	1	1	1	1	0	0	1
1	1	56	1	178	85.7	-1.30	27.0	123	83	232	68	164.00	5.10	1	1.00	0	0	1	0	1	1	0	0	1	0	1
0	0	81	1	152	57.4	-2.30	24.7	110	76	135	21	114.00	5.50	1	1.00	1	0	1	0	1	0	0	1	0	0	1
1	1	81	2	149	50.7	2.70	22.9	162	89	210	68	142.00	5.40	1	1.00	1	0	1	0	1	1	1	1	0	1	1
1	1	69	1	160	63.8	1.50	24.9	132	70	151	63	88.00	7.40	1	1.00	0	0	1	0	1	1	1	1	1	1	0
0	0	79	2	146	45.5	3.90	21.3	111	67	187	39	148.00	5.70	1	1.00	1	0	1	0	1	0	0	1	0	0	1
1	1	51	2	159	50.2	.70	19.9	102	70	182	62	120.00	5.50	1	1.00	1	0	1	0	1	0	0	1	1	1	1
0	0	76	2	147	45.9	2.50	21.2	133	80	215	62	153.00	5.20	1	1.00	1	0	1	1	1	0	0	1	1	1	1
1	1	50	2	154	57.2	1.30	23.9	95	61	206	70	136.00	5.10	1	1.00	1	1	1	1	1	1	0	1	1	1	1
1	1	59	1	165	69.2	-4.70	25.5	137	86	224	58	166.00	6.80	1	1.00	0	0	1	1	1	1	1	0	0	0	0
1	1	79	2	139	48.5	-1.10	25.0	110	67	266	72	194.00	5.50	1	1.00	1	0	1	1	1	0	0	0	1	0	1
0	0	71	1	167	64.5	1.80	23.1	136	92	178	69	109.00	5.60	1	1.00	0	0	1	1	1	0	1	1	0	1	1
1	1	71	2	144	49.8	-1.10	23.9	134	82	204	57	147.00	5.80	1	1.00	1	1	1	1	1	1	0	1	0	1	1
1	1	68	1	165	63.6	-2.10	23.3	106	63	198	82	116.00	6.20	1	1.00	0	0	1	0	1	0	1	1	1	1	0
1	1	66	2	144	45.2	.80	21.7	159	78	223	64	159.00	6.00	1	1.00	1	1	1	0	1	1	1	1	0	0	1
1	1	67	2	150	51.1	-2.70	22.8	126	76	307	55	252.00	5.60	1	1.00	1	1	1	0	1	0	0	1	0	0	1
1	1	67	1	166	60.0	.80	21.8	140	82	229	92	137.00	5.10	1	1.00	0	0	1	0	1	0	1	1	0	0	1
1	1	78	1	167	73.3	1.20	26.2	142	90	253	66	187.00	6.00	1	1.00	0	0	1	1	1	1	1	0	0	0	1
1	1	61	2	152	52.2	-2.40	22.5	125	74	213	42	171.00	5.10	1	1.00	1	0	1	0	0	0	0	1	1	1	1
1	1	64	2	162	64.0	-.10	24.4	148	85	289	62	227.00	6.00	1	1.00	0	0	1	1	1	1	1	1	0	0	1
1	1	58	2	160	54.8	-.30	21.4	166	85	203	51	152.00	5.80	1	1.00	1	1	1	1	0	1	1	1	0	0	1
1	1	70	1	160	54.4	-.50	21.3	152	86	206	53	153.00	5.30	1	1.00	1	0	1	1	1	1	1	1	0	1	1
1	1	61	2	149	47.9	-3.60	21.6	139	89	226	59	167.00	5.30	1	1.00	1	0	1	0	0	1	0	1	0	0	1
1	1	72	1	165	70.0	.80	25.6	126	83	227	53	174.00	6.80	0	1.00	0	1	1	0	1	1	1	0	0	0	0

1	1	62	2	148	48.6	2.10	22.2	133	80	198	75	123.00	5.90	1	1.00	1	1	1	1	1	1	1	1	1	1	1
1	1	55	2	151	65.2	2.10	28.6	116	81	262	47	215.00	7.90	1	1.00	0	0	1	0	1	0	0	0	1	0	0
1	1	56	1	170	74.0	.80	25.6	138	77	222	58	164.00	5.80	1	1.00	1	1	1	0	1	1	1	0	0	0	1
0	0	77	2	147	43.0	-6.00	19.8	148	78	161	52	109.00	6.90	1	1.00	1	0	1	1	1	0	1	1	0	1	0
0	0	76	1	158	54.7	-.10	22.0	102	64	145	52	93.00	4.60	1	1.00	0	0	1	1	1	1	1	1	0	1	1
1	1	53	1	162	58.0	-1.70	22.0	170	111	240	88	152.00	5.80	0	1.00	0	1	1	0	0	1	1	1	0	0	1
1	1	54	2	145	54.3	-1.90	25.9	122	71	201	43	158.00	5.50	1	1.00	0	1	1	0	0	0	1	0	1	1	1
1	1	60	1	162	72.5	-.50	27.5	130	71	195	45	150.00	5.80	1	1.00	0	0	1	0	1	1	1	0	0	1	1
0	0	66	2	148	56.2	1.60	25.6	124	69	280	74	206.00	6.10	1	1.00	1	0	1	1	1	1	1	0	0	0	1
1	1	68	2	148	42.2	1.00	19.1	162	89	206	103	103.00	5.30	1	1.00	1	1	1	1	1	0	0	1	0	1	1
1	1	62	1	161	61.6	-3.70	23.7	107	75	183	53	130.00	6.90	1	1.00	0	0	1	0	1	0	0	1	0	0	0
1	1	73	1	156	75.3	-13.50	30.8	142	84	156	49	107.00	6.40	0	2.00	0	0	1	1	0	0	1	0	0	1	1
1	1	69	2	151	43.7	-.10	19.1	107	57	236	58	178.00	6.30	1	1.00	1	0	1	1	1	1	1	1	1	0	1
1	1	82	1	155	60.6	1.30	25.2	139	77	228	54	174.00	6.30	1	1.00	0	1	1	0	1	1	1	0	1	0	1
1	1	83	2	145	57.1	.00	27.1	136	71	218	61	157.00	5.90	1	1.00	1	0	1	1	1	1	1	0	1	1	0
0	0	69	2	150	48.3	-.20	21.4	106	61	195	53	142.00	5.50	1	1.00	1	1	1	0	1	0	1	1	1	1	1
0	0	71	1	157	63.2	2.50	25.8	125	65	194	47	147.00	5.50	1	1.00	0	0	1	0	1	0	1	0	1	1	1
1	1	74	1	156	63.0	.30	26.0	143	81	205	55	150.00	6.20	1	1.00	1	1	1	1	1	1	1	0	0	0	0
1	1	47	2	161	55.0	-1.20	21.3	113	68	205	66	139.00	5.80	1	1.00	1	0	1	1	1	1	1	1	1	1	1
1	1	72	2	141	40.0	-1.30	20.0	131	81	226	67	159.00	5.60	1	1.00	1	0	1	0	1	1	1	1	0	0	1
1	1	78	2	148	50.3	1.40	23.0	165	86	274	86	188.00	6.30	1	1.00	1	0	1	1	1	1	1	1	0	0	1
1	1	68	2	140	44.4	.40	22.8	139	75	265	95	170.00	6.00	1	1.00	1	0	1	1	1	0	0	1	1	0	1
0	0	66	2	152	53.9	3.90	23.3	127	76	206	52	154.00	5.50	1	1.00	1	0	1	0	1	1	0	1	1	1	1
1	1	77	1	161	54.2	-.40	21.0	143	74	162	72	90.00	5.70	1	1.00	1	1	1	1	1	1	1	1	0	1	1
0	0	62	2	154	56.1	.70	23.7	116	65	227	108	119.00	6.30	1	1.00	1	0	1	1	1	1	1	1	0	0	1
0	0	72	2	153	65.4	1.40	28.0	125	81	246	70	176.00	5.60	1	1.00	1	1	1	0	0	0	0	0	1	0	1
1	1	64	2	142	50.0	-1.30	24.9	96	56	202	56	146.00	5.30	1	1.00	1	0	1	1	1	0	1	1	1	1	1
0	0	36	2	152	46.8	3.00	20.2	100	55	186	49	137.00	5.60	1	1.00	1	0	1	1	1	1	0	1	1	1	1
1	1	65	2	150	59.8	1.80	26.7	132	73	282	74	208.00	5.70	1	1.00	1	0	1	1	1	1	1	0	1	0	1
0	0	62	1	168	81.8	.10	29.0	157	111	243	44	199.00	5.50	1	1.00	1	1	1	0	1	0	0	0	0	0	1
1	1	58	1	174	76.6	.40	25.1	143	67	261	85	176.00	5.70	0	1.00	0	1	1	1	1	1	1	0	0	0	1
0	0	79	2	147	56.2	.20	25.8	131	66	159	70	89.00	5.70	1	1.00	1	0	1	0	1	0	1	0	0	1	1
1	1	78	1	148	56.9	-.30	25.9	125	79	219	44	175.00	6.20	1	1.00	1	1	1	1	1	1	1	0	1	1	1
0	0	72	2	148	52.3	2.70	23.7	130	72	205	83	122.00	5.00	1	1.00	1	0	1	1	1	1	1	1	1	0	1
0	0	60	1	165	56.9	1.80	20.9	124	76	227	83	144.00	5.80	1	1.00	0	1	1	1	1	1	0	1	1	0	1
1	1	71	1	158	51.8	.00	20.6	153	85	222	79	143.00	5.50	1	1.00	0	0	1	1	1	1	1	1	0	0	1
0	0	56	1	168	60.8	4.90	21.6	147	102	272	83	189.00	5.50	0	2.00	0	1	1	0	1	1	1	1	0	0	1
1	1	78	1	159	56.2	-2.20	22.3	113	65	199	81	118.00	5.40	1	1.00	1	1	1	1	1	1	1	1	1	1	1
1	1	80	1	152	48.6	.30	20.9	155	79	193	60	133.00	8.70	1	1.00	1	1	1	1	1	1	1	1	0	1	0

1	1	78	2	142	47.7	-.60	23.6	139	68	194	62	132.00	5.00	1	1.00	1	1	1	0	1	1	1	1	1	1	1
1	1	80	1	151	59.5	.30	26.1	130	69	172	51	121.00	5.50	1	1.00	1	1	1	1	1	1	1	0	0	1	1
0	1	69	2	148	54.6	-.20	24.8	118	78	224	71	153.00	5.20	1	1.00	1	1	1	0	1	1	1	1	0	0	1
1	1	67	1	161	50.7	-.80	19.4	141	85	192	65	127.00	7.40	1	1.00	1	1	1	1	1	1	1	1	0	1	0
1	1	72	2	155	57.9	3.40	24.0	162	94	206	48	158.00	5.60	1	1.00	1	1	1	0	1	0	0	1	0	1	1
1	1	36	2	154	46.2	.60	19.5	148	72	179	75	104.00	5.30	1	1.00	1	0	1	0	1	1	1	1	0	1	1
1	1	53	1	169	78.1	-1.20	27.3	129	93	175	40	135.00	5.50	0	1.00	0	0	1	0	1	1	1	0	0	1	1
1	1	41	1	168	84.6	2.10	29.9	121	69	241	59	182.00	5.50	1	1.00	1	0	1	0	0	0	1	0	1	0	1
1	1	76	2	147	52.3	-.50	24.2	100	58	243	58	185.00	5.70	1	1.00	1	0	1	1	1	0	0	1	0	0	1
1	1	64	1	161	67.8	-2.00	26.0	135	87	192	52	140.00	5.80	1	1.00	0	1	1	1	1	1	1	0	1	1	1
1	1	59	2	146	52.0	-2.50	24.4	131	78	230	58	172.00	5.50	1	1.00	1	0	1	0	1	0	1	1	1	0	1
1	1	75	2	148	60.8	-1.40	27.9	141	75	319	63	256.00	10.10	1	1.00	1	0	1	1	1	1	1	0	0	0	0
1	1	73	1	166	50.3	-.60	18.2	147	74	189	76	113.00	5.60	0	1.00	0	0	1	0	1	1	1	1	0	1	1
1	1	69	2	145	45.3	-1.20	21.6	121	64	276	86	190.00	6.10	1	1.00	1	0	1	0	1	0	1	1	1	0	1
1	1	62	2	146	39.8	-2.10	18.7	110	61	168	54	114.00	5.70	1	1.00	1	0	1	0	1	0	0	1	1	0	1
0	0	74	2	148	49.8	2.00	22.6	157	71	223	59	164.00	5.70	1	1.00	1	0	1	0	1	0	0	1	0	0	1
0	0	71	2	146	46.7	6.50	22.0	138	86	185	81	104.00	5.60	1	1.00	1	0	1	0	1	0	0	1	0	1	1
0	1	69	2	145	65.6	-8.50	31.2	114	70	241	63	178.00	5.60	1	1.00	1	1	1	0	0	1	1	0	0	0	1
0	0	68	2	154	59.9	-1.40	25.2	121	71	207	34	173.00	5.20	1	1.00	1	0	1	0	0	1	0	0	0	0	1
0	0	51	2	152	46.5	1.40	20.1	115	74	184	82	102.00	5.60	1	1.00	1	1	1	0	1	0	1	1	1	1	1
1	1	52	2	158	57.7	-1.10	22.9	108	61	292	62	230.00	5.90	1	1.00	0	0	1	1	1	1	0	1	1	0	1
1	1	67	1	161	62.2	.80	23.9	119	69	179	69	110.00	5.30	1	1.00	0	1	1	1	1	1	1	1	0	1	1
1	1	79	1	167	67.5	-.20	24.3	139	76	206	56	150.00	6.00	1	1.00	1	0	1	1	1	1	1	1	0	1	0
1	1	38	2	167	57.2	-.40	20.5	114	66	176	72	104.00	5.20	1	1.00	1	1	1	1	1	1	1	1	1	1	1
1	1	72	2	156	56.1	1.10	23.2	169	96	215	76	139.00	5.70	1	1.00	1	0	1	0	1	0	1	1	0	1	1
1	1	58	2	149	47.8	-.20	21.6	171	107	226	102	124.00	5.50	1	1.00	1	0	1	0	0	1	1	1	0	0	1
0	0	73	2	156	58.1	5.60	23.9	105	57	188	77	111.00	5.90	1	1.00	1	0	1	1	1	1	1	1	0	1	1
0	0	60	2	154	57.0	1.70	24.1	130	75	212	55	157.00	5.90	1	1.00	1	0	1	1	1	1	1	1	1	1	1
0	0	73	2	144	47.7	2.20	22.9	128	75	198	73	125.00	5.50	1	1.00	1	0	1	0	1	0	0	1	1	1	1
0	0	67	1	154	64.0	-.50	27.0	116	72	196	47	149.00	5.10	1	1.00	1	0	1	0	1	1	1	0	1	1	1
1	1	86	1	151	49.9	3.40	21.9	133	74	200	64	136.00	5.40	1	1.00	1	0	1	1	1	0	1	1	0	1	1
0	0	87	2	153	60.2	1.90	25.6	145	82	191	47	144.00	5.40	1	1.00	1	0	1	1	1	1	1	0	0	1	1
1	1	72	2	137	43.0	.70	22.7	110	63	244	62	182.00	5.60	1	1.00	1	0	1	0	1	0	0	1	1	0	1
0	0	42	2	155	55.8	-.40	23.3	117	77	164	65	99.00	5.80	1	1.00	1	0	1	0	1	0	1	1	1	1	1
1	1	70	1	157	63.9	6.70	25.8	163	92	188	49	139.00	5.40	1	1.00	0	1	1	1	1	1	1	0	0	1	1
0	1	68	2	147	49.5	1.20	22.7	98	58	192	58	134.00	5.70	1	1.00	1	0	1	1	1	0	1	1	1	0	1
0	0	64	2	151	51.8	2.30	22.6	140	77	202	78	124.00	6.50	1	1.00	1	0	1	0	0	1	0	1	0	1	0
1	1	72	2	150	56.6	2.80	25.2	135	78	157	63	94.00	5.60	1	1.00	1	0	1	1	1	1	1	0	0	1	1
1	1	66	1	166	66.5	-1.00	24.1	174	86	184	99	85.00	4.90	1	1.00	0	1	1	1	1	1	1	1	0	1	1

0	0	64	1	168	57.6	-2.80	20.5	125	80	169	69	100.00	6.00	0	1.00	0	1	1	1	1	1	1	1	1	1	1
0	0	44	1	170	70.2	3.10	24.4	119	76	204	59	145.00	5.50	0	1.00	1	0	1	0	1	0	1	1	1	1	1
1	1	55	2	155	72.5	4.00	30.0	112	78	207	42	165.00	5.40	1	1.00	1	0	1	1	1	1	1	0	0	1	1
0	0	62	1	155	64.9	1.40	26.9	158	98	186	58	128.00	5.40	0	1.00	0	0	1	0	0	0	1	0	0	1	1
1	1	79	2	146	46.1	4.70	21.7	125	66	170	56	114.00	5.70	1	1.00	1	0	0	1	1	1	1	1	1	1	1
1	1	86	2	144	53.9	.90	25.8	136	76	214	73	141.00	6.30	1	1.00	1	0	1	1	1	0	0	0	1	1	1
1	1	77	2	131	40.3	-.60	23.5	117	67	236	51	185.00	6.10	1	1.00	1	1	1	0	1	0	1	1	0	0	1
1	1	61	2	165	60.0	2.10	22.1	136	78	158	77	81.00	6.00	1	1.00	0	0	1	1	1	1	1	1	0	1	1
1	1	71	1	167	66.6	-2.10	23.8	113	77	237	70	167.00	6.30	0	1.00	0	0	1	0	1	0	0	1	1	0	1
1	1	75	2	151	54.8	.80	23.9	126	70	197	65	132.00	5.80	1	1.00	1	0	1	1	1	0	1	1	1	1	1
1	1	70	1	172	67.7	2.30	22.8	148	77	157	61	96.00	5.60	1	1.00	0	1	1	1	1	1	0	1	0	1	1
1	1	56	2	147	47.8	.80	22.0	130	94	218	54	164.00	5.90	1	1.00	1	0	1	0	0	1	1	1	0	1	1
0	1	83	2	145	38.2	-1.30	18.2	134	70	174	56	118.00	6.10	1	1.00	1	0	1	0	1	0	0	1	0	0	1
1	1	45	1	180	76.5	-1.30	23.6	115	81	268	45	223.00	5.50	0	1.00	1	1	1	0	0	1	1	1	1	0	1
1	1	74	2	151	52.0	-.30	22.8	151	75	227	84	143.00	5.70	1	1.00	1	1	1	1	0	1	0	1	0	0	1
0	0	82	1	157	65.1	-1.60	26.3	129	68	204	54	150.00	5.90	1	1.00	0	0	1	0	1	1	0	0	0	1	1
1	1	57	2	157	49.4	1.80	20.1	117	89	230	79	151.00	5.30	1	1.00	0	0	1	0	0	1	1	1	1	0	1
0	0	61	1	164	64.6	-.20	23.9	127	85	292	62	230.00	5.60	1	1.00	1	0	1	1	0	0	0	1	1	0	1
1	1	57	2	162	74.7	-.10	28.3	135	98	246	51	195.00	5.40	1	1.00	1	0	1	1	1	1	1	0	0	0	1
0	0	74	2	137	41.9	-2.60	22.2	116	69	175	51	124.00	5.40	1	1.00	1	0	1	0	1	0	0	1	1	1	1
1	1	71	2	153	59.4	-1.10	25.5	132	81	220	60	160.00	5.90	1	1.00	1	1	1	1	1	1	0	0	0	0	1
1	1	61	1	161	62.0	-.80	23.8	173	80	215	59	156.00	5.50	0	1.00	0	0	1	1	1	1	1	1	0	1	1
1	1	69	2	158	54.4	1.10	21.8	132	80	210	74	136.00	5.80	1	1.00	1	1	1	1	1	1	1	1	1	1	1
0	0	70	2	153	69.1	2.00	29.5	122	76	207	78	129.00	5.70	1	1.00	1	0	1	0	1	1	1	0	1	0	1
1	1	64	1	156	55.6	-2.20	22.7	170	91	203	56	147.00	5.70	1	1.00	0	0	1	1	1	1	1	1	0	1	1
1	1	59	1	169	63.4	-.60	22.2	148	88	199	60	139.00	5.30	1	1.00	1	0	1	0	1	0	1	1	0	1	1
1	1	79	1	165	81.4	-3.00	29.7	153	93	196	71	125.00	6.00	1	1.00	1	0	1	1	1	0	1	0	0	1	1
1	1	85	2	146	46.3	1.30	21.7	154	65	195	62	133.00	5.60	1	1.00	1	0	1	0	1	0	0	1	0	1	1
1	1	68	1	156	54.7	-.90	22.6	149	89	163	67	96.00	5.80	1	1.00	0	0	1	1	1	1	1	1	0	1	1
1	1	73	2	144	52.7	-2.50	25.5	140	78	216	70	146.00	5.50	1	1.00	1	0	1	0	1	1	1	0	0	1	1
1	1	57	2	152	51.0	1.00	22.1	138	92	273	54	219.00	5.40	1	1.00	1	1	1	1	1	1	1	1	0	0	1
1	1	81	2	141	38.8	1.10	19.4	114	65	188	64	124.00	5.70	1	1.00	1	1	1	1	1	1	0	1	0	1	1
1	1	79	1	158	61.6	-2.10	24.5	132	67	194	35	159.00	5.60	1	1.00	1	1	1	1	1	1	1	1	1	0	1
1	1	77	1	152	45.8	.90	19.7	90	59	186	59	127.00	7.00	1	1.00	1	1	1	1	1	1	1	1	0	1	0
1	1	72	2	147	43.1	.20	19.9	106	63	196	81	115.00	5.40	1	1.00	1	1	1	1	1	0	0	1	1	1	1
1	1	75	1	154	68.8	.30	28.8	103	63	171	54	117.00	6.20	1	1.00	1	1	1	1	1	1	0	0	1	1	0
1	1	75	2	150	44.8	-.90	19.8	117	64	192	45	147.00	5.70	1	1.00	1	1	1	1	1	0	1	1	1	1	1
1	1	68	2	155	51.6	2.40	21.4	139	91	184	61	123.00	5.40	1	1.00	1	0	1	0	1	1	1	1	0	1	1
1	1	74	2	152	55.6	-.40	23.9	123	71	209	60	149.00	5.40	1	1.00	1	0	1	1	1	1	1	1	0	1	1

1	1	69	1	149	52.0	-.40	23.4	118	68	216	65	151.00	6.30	1	1.00	0	1	1	1	1	1	1	1	1	1	1
1	1	71	1	162	87.7	-3.40	33.6	137	81	195	60	135.00	6.00	1	1.00	0	0	1	1	1	1	1	0	0	1	1
1	1	56	1	164	61.4	1.60	22.9	136	80	204	71	133.00	5.30	1	1.00	0	1	1	0	0	0	1	1	1	1	1
1	1	56	2	159	46.9	1.50	18.5	132	80	170	98	72.00	5.20	0	1.00	0	0	1	1	1	1	1	1	1	1	1
0	0	70	2	145	46.5	1.10	22.2	143	80	252	60	192.00	5.50	1	1.00	1	0	1	0	1	1	1	1	0	0	1
0	0	72	2	145	50.5	-2.50	24.0	131	65	212	53	159.00	6.30	1	1.00	1	0	1	1	1	1	0	1	0	1	0
1	1	77	2	145	49.1	-.50	23.3	144	81	224	71	153.00	5.50	1	1.00	1	0	1	1	1	1	1	1	0	0	1
1	1	63	2	150	43.2	-.70	19.2	126	70	213	70	143.00	5.50	1	1.00	1	0	1	1	1	1	1	1	0	1	1
0	0	41	2	155	47.0	1.40	19.5	117	80	212	78	134.00	5.60	1	1.00	1	0	1	1	1	0	1	1	1	1	1
1	1	63	1	169	80.0	2.10	28.1	104	64	180	57	123.00	7.00	1	1.00	0	1	1	0	1	1	1	0	1	1	0
1	1	81	1	158	60.2	1.00	24.0	127	74	192	58	134.00	5.60	1	1.00	1	0	1	1	1	1	1	1	1	1	1
1	1	69	2	152	58.1	.70	25.1	144	85	205	62	143.00	5.80	1	1.00	0	0	1	1	1	0	1	0	0	1	1
0	0	23	2	161	47.0	-1.00	18.1	104	62	152	80	72.00	5.20	1	1.00	1	0	1	0	0	1	1	1	1	1	1
0	0	51	2	152	41.4	1.00	17.8	111	64	150	63	87.00	6.30	1	1.00	1	0	1	0	1	0	0	1	1	1	1
0	0	58	2	150	44.2	.40	19.5	105	66	236	58	178.00	5.80	1	1.00	1	0	1	0	1	1	1	1	1	0	1
0	0	72	1	164	75.9	3.10	28.3	136	83	151	40	111.00	9.00	1	1.00	1	0	1	0	1	0	1	0	0	1	0
1	1	65	2	148	65.4	.50	29.7	104	64	246	47	199.00	6.00	1	1.00	1	0	1	0	1	1	1	0	1	0	1
1	1	63	1	159	61.0	-.60	24.0	135	81	227	46	181.00	6.30	1	1.00	1	1	1	1	0	1	1	1	0	0	1
1	1	41	2	153	57.4	2.30	24.3	110	61	226	61	165.00	5.50	1	1.00	1	0	1	0	1	1	1	1	1	0	1
1	1	80	1	162	55.1	.20	21.1	134	71	179	76	103.00	5.30	1	1.00	0	0	1	1	1	1	1	1	0	1	1
1	1	59	1	164	50.0	-2.70	18.7	111	64	131	73	58.00	5.60	0	1.00	0	0	1	0	1	1	1	1	1	1	1
1	1	81	2	146	39.3	.30	18.3	130	73	218	82	136.00	6.00	1	1.00	1	0	1	0	1	0	0	1	1	1	1
0	1	81	1	152	57.1	-2.60	24.8	134	79	235	59	176.00	5.90	1	1.00	1	0	1	0	1	0	0	1	1	0	1
1	1	73	1	158	64.8	-.90	26.0	112	72	229	47	182.00	5.90	1	1.00	0	0	1	0	1	1	1	0	0	0	1
1	1	36	1	162	76.9	-.50	29.4	126	82	238	48	190.00	5.50	0	1.00	0	1	1	0	1	1	1	0	1	0	1
0	0	41	2	146	40.5	.00	19.0	99	64	165	58	107.00	5.00	1	1.00	0	0	1	0	1	0	0	1	1	1	1
1	1	34	2	163	52.3	.70	19.6	102	61	196	51	145.00	5.80	1	1.00	0	1	0	1	1	1	0	1	1	1	1
0	0	62	2	154	60.7	-1.60	25.5	124	72	219	61	158.00	6.50	1	1.00	1	0	1	0	1	0	0	0	0	0	0
1	1	76	2	146	48.5	.70	22.9	116	70	185	71	114.00	5.30	1	1.00	1	0	1	1	1	1	1	1	1	1	1
0	0	48	2	155	46.0	3.30	19.1	103	66	227	67	160.00	5.90	1	1.00	1	0	1	1	1	1	1	1	1	0	1
1	1	63	1	170	65.2	.30	22.6	96	57	179	85	94.00	6.00	0	1.00	0	1	1	0	1	0	1	1	1	1	1
0	0	66	1	171	74.0	.60	25.2	132	87	198	45	153.00	5.50	1	1.00	0	1	1	0	1	1	1	0	1	1	1
0	1	29	1	182	75.9	2.00	22.8	105	56	194	47	147.00	5.30	0	1.00	0	1	1	0	1	1	1	1	1	1	1
1	1	27	2	148	51.8	3.00	23.6	96	59	200	71	129.00	5.10	0	1.00	1	1	0	0	0	0	1	1	1	1	1
1	1	44	2	146	44.1	-2.00	20.5	148	78	278	62	216.00	5.20	1	1.00	0	1	1	1	1	1	1	1	0	0	1
0	0	66	2	150	57.0	1.40	25.3	119	74	221	57	164.00	5.50	1	1.00	1	0	1	0	1	1	1	0	1	0	1
1	1	71	2	149	53.8	1.60	24.2	131	83	219	59	160.00	5.80	1	1.00	1	1	1	1	1	1	1	1	0	1	1
1	1	72	1	156	61.4	-1.70	25.2	107	57	165	66	99.00	5.30	1	1.00	0	0	1	1	1	1	0	0	1	1	1
1	1	68	2	151	49.6	2.90	21.7	93	52	176	61	115.00	5.80	1	1.00	1	0	1	0	1	0	0	1	1	0	1

1	1	45	2	154	54.6	2.00	22.9	119	76	255	58	197.00	4.90	0	1.00	1	0	1	0	0	1	1	1	1	0	1
0	0	39	2	158	58.3	9.40	23.2	118	71	189	56	133.00	5.00	1	1.00	1	1	1	0	1	0	1	1	1	1	1
1	1	53	2	158	64.9	-10.70	26.0	123	80	260	57	203.00	5.60	1	1.00	0	1	1	0	0	0	0	0	1	0	1
0	0	70	2	154	52.8	.60	22.2	120	70	167	71	96.00	5.40	1	1.00	1	0	1	1	1	1	1	1	1	0	1
0	0	60	2	146	40.8	.40	19.0	98	57	205	79	126.00	6.10	1	1.00	1	0	1	0	0	0	1	1	1	1	1
1	1	68	2	149	47.1	1.90	21.2	107	60	201	59	142.00	9.30	1	1.00	1	1	1	1	1	1	1	1	1	1	0
0	0	73	1	150	49.3	2.30	21.9	115	69	187	50	137.00	5.90	1	1.00	0	1	1	1	1	1	0	1	1	1	1
1	1	82	1	164	61.4	-2.30	22.7	153	82	133	53	80.00	7.00	1	1.00	1	1	1	1	1	1	1	1	0	1	0
0	0	61	2	149	41.9	1.70	18.7	97	53	186	61	125.00	6.50	1	1.00	1	1	1	1	1	1	1	1	1	1	0
1	1	60	2	150	43.3	.90	19.2	120	70	242	67	175.00	5.90	1	1.00	1	0	1	0	1	1	1	1	1	0	1
1	1	63	2	153	53.7	.50	22.9	125	76	342	68	274.00	5.90	1	1.00	1	1	1	1	1	0	1	1	1	0	1
0	0	66	2	153	77.2	-1.40	32.8	138	87	261	76	185.00	5.50	1	1.00	1	0	1	1	1	1	0	0	1	0	1
1	1	76	1	167	73.9	-2.30	26.6	143	80	168	42	126.00	6.70	1	1.00	1	0	1	1	1	1	1	0	0	1	0
1	1	64	2	145	43.2	-.60	20.5	94	62	158	77	81.00	5.60	1	1.00	1	0	1	0	1	1	1	1	1	1	1
1	1	61	1	161	53.1	2.80	20.3	148	90	183	62	121.00	5.10	1	1.00	0	1	0	0	1	1	1	1	0	1	1
1	1	75	1	163	67.7	-.20	25.5	132	73	207	64	143.00	5.70	1	1.00	0	0	1	1	1	0	1	0	0	1	1
1	1	64	2	146	50.2	-1.40	23.5	140	87	237	50	187.00	5.20	1	1.00	1	0	1	1	0	1	1	1	0	0	1
1	1	81	1	158	70.5	-1.50	28.1	115	66	154	58	96.00	5.30	1	1.00	0	0	1	1	1	1	1	0	1	1	1
0	0	79	2	135	44.9	2.90	24.7	130	66	215	57	158.00	5.00	1	1.00	1	0	1	1	0	0	1	1	0	1	1
1	1	59	1	166	56.2	-1.00	20.3	133	78	227	92	135.00	5.10	1	1.00	0	0	1	0	1	0	1	1	1	0	1
1	1	76	1	163	65.1	-.50	24.5	172	89	142	46	96.00	6.20	1	1.00	0	0	1	1	1	1	1	1	0	1	1
1	1	83	1	146	59.2	3.40	27.9	169	92	220	92	128.00	5.50	1	1.00	1	1	1	0	1	0	1	0	0	0	0
0	0	67	1	169	71.1	-1.60	24.7	115	75	234	70	164.00	5.70	1	1.00	0	0	1	1	1	1	0	1	1	0	1
1	1	25	1	166	81.2	-1.50	29.5	121	70	175	54	121.00	4.60	1	1.00	1	1	1	1	1	1	1	0	1	1	1
1	1	64	2	154	44.3	-.80	18.6	152	90	229	80	149.00	5.80	1	1.00	0	0	1	0	1	0	0	1	0	0	1
1	1	67	2	149	50.7	-2.60	22.8	156	82	237	50	187.00	5.70	1	1.00	1	1	1	0	1	0	1	1	0	0	1
1	1	63	2	144	40.0	-.80	19.3	86	61	181	65	116.00	5.60	1	1.00	1	1	1	1	1	1	1	1	1	1	1
1	1	78	1	153	61.9	-1.10	26.4	125	69	171	53	118.00	5.20	1	1.00	1	0	1	0	0	1	1	0	0	1	1
1	1	79	1	158	56.8	-1.10	22.6	128	68	213	70	143.00	5.60	1	1.00	1	0	1	0	1	0	0	1	1	1	1
1	1	77	1	169	59.6	-1.30	20.8	115	68	214	62	152.00	4.90	0	1.00	1	0	1	1	1	1	1	1	1	1	1
1	1	27	2	157	69.2	-6.00	28.1	116	72	225	43	182.00	5.20	1	1.00	1	0	1	0	1	1	1	0	1	0	1
1	1	49	1	170	103.6	-.30	35.8	142	90	339	44	295.00	6.60	0	1.00	0	0	0	1	1	1	0	0	0	0	0
1	1	46	2	153	56.4	-1.20	23.9	103	55	167	62	105.00	5.10	1	1.00	1	1	1	0	1	0	0	1	1	1	1
1	1	34	1	171	67.6	2.80	23.0	111	69	167	63	104.00	5.10	1	1.00	0	1	1	0	1	1	1	1	1	1	1
1	1	75	1	154	58.7	-1.70	24.6	120	79	208	67	141.00	5.20	1	1.00	0	0	1	1	0	1	1	1	0	1	1
1	1	71	1	166	60.4	-2.90	22.0	99	56	178	76	102.00	5.60	1	1.00	1	0	1	1	0	1	1	1	1	1	1
1	1	77	2	147	54.6	-3.30	25.4	162	83	152	47	105.00	5.70	1	1.00	1	1	1	1	1	1	1	0	0	1	1
1	1	75	2	152	56.0	-3.00	24.1	128	68	200	42	158.00	6.00	1	1.00	1	0	1	0	0	0	1	1	0	1	1
0	0	68	1	156	63.2	1.20	25.9	130	83	178	36	142.00	5.10	1	1.00	0	0	1	0	0	1	0	0	1	0	1

1	1	70	1	155	53.5	-.80	22.2	125	79	256	83	173.00	5.40	0	1.00	0	1	1	0	1	1	1	1	1	0	1
1	1	59	2	156	57.4	.50	23.6	125	71	221	63	158.00	6.10	1	1.00	1	1	1	0	0	0	0	1	0	0	1
0	0	43	1	165	64.1	-.90	23.6	138	83	235	81	154.00	5.60	0	1.00	0	0	1	0	1	1	1	1	1	0	1
1	1	68	1	163	62.0	.30	23.2	109	67	195	59	136.00	5.50	1	1.00	0	1	1	0	1	1	1	1	0	1	1
1	1	78	1	152	55.1	.20	23.8	148	86	203	44	159.00	6.20	0	1.00	1	1	1	0	1	1	1	1	0	1	1
0	0	76	1	160	57.4	1.10	22.5	100	59	236	68	168.00	5.40	1	1.00	1	0	1	0	1	1	0	1	1	0	1
0	0	79	2	137	47.3	-.60	25.1	131	63	190	45	145.00	6.20	1	1.00	1	0	1	0	1	1	0	0	0	1	1
0	0	60	2	159	51.7	3.10	20.3	119	77	261	54	207.00	5.80	1	1.00	1	1	1	0	1	1	1	1	1	0	1
0	0	66	2	143	46.7	1.30	22.8	124	76	214	78	136.00	6.40	1	1.00	1	1	1	0	1	1	1	1	0	1	1
1	1	61	1	164	58.7	2.70	21.7	111	68	204	61	143.00	5.80	1	1.00	1	0	1	0	1	1	1	1	1	1	1
1	1	62	2	158	50.6	-.80	20.2	121	66	180	51	129.00	5.90	1	1.00	1	1	1	0	1	1	1	1	1	1	1
0	0	64	2	151	46.7	-1.60	20.6	101	54	217	66	151.00	5.40	1	1.00	1	0	1	1	1	1	1	1	1	1	1
1	1	70	2	154	55.5	-.80	23.3	102	55	223	73	150.00	5.70	1	1.00	1	0	1	1	1	1	0	1	1	0	1
0	0	79	2	145	44.5	-.30	21.1	144	71	192	53	139.00	5.80	1	1.00	1	0	1	0	1	0	0	1	0	1	0
1	1	62	2	160	80.7	-5.20	31.6	111	65	335	76	259.00	7.40	1	1.00	1	0	0	0	0	0	0	0	1	0	0
1	1	39	2	154	53.6	1.10	22.5	108	64	306	80	226.00	5.40	1	1.00	1	0	1	0	0	0	0	1	1	0	1
0	0	75	1	152	53.4	1.20	23.1	142	77	184	58	126.00	5.70	1	1.00	1	0	1	0	1	0	0	1	0	1	1
1	1	69	1	160	54.7	-1.50	21.4	125	76	244	82	162.00	5.60	1	1.00	0	0	1	0	1	0	0	1	1	0	1
1	1	45	1	178	105.8	-1.00	33.3	137	78	206	58	148.00	5.80	0	1.00	0	0	1	1	1	1	1	0	1	1	0
1	1	65	1	174	78.1	.30	25.7	125	78	229	69	160.00	5.80	1	1.00	0	1	1	0	1	1	1	0	0	0	1
1	1	61	2	162	53.3	-2.20	20.4	139	82	174	55	119.00	6.00	1	1.00	1	0	1	1	1	1	1	1	0	1	0
1	1	43	1	166	79.5	.90	28.7	152	98	223	53	170.00	5.00	0	1.00	1	1	1	1	1	1	1	0	0	0	1
1	1	61	2	150	54.4	1.90	24.2	104	69	154	38	116.00	5.20	1	1.00	1	0	1	0	0	0	1	1	0	0	1
1	1	67	1	155	44.8	-2.40	18.6	136	80	169	86	83.00	5.30	0	1.00	0	0	1	0	1	1	1	1	1	1	1
1	1	86	1	154	56.9	.50	23.9	105	63	163	64	99.00	4.90	1	1.00	1	1	1	0	0	0	1	1	1	1	1
1	1	43	2	160	63.0	-.70	24.7	106	58	203	63	140.00	5.40	1	1.00	1	0	1	0	0	0	1	1	1	1	1
1	1	38	2	156	52.5	2.50	21.5	102	57	179	90	89.00	5.00	1	1.00	1	1	1	0	0	1	0	1	1	1	1
1	1	71	2	144	57.2	.20	27.6	153	86	196	81	115.00	6.00	1	1.00	1	0	1	0	0	1	0	0	0	0	1
1	1	79	1	166	75.1	-1.50	27.1	148	65	202	44	158.00	6.90	1	1.00	1	0	1	1	1	0	1	0	0	1	0
1	1	74	1	167	67.1	-.90	24.0	146	88	213	48	165.00	5.40	1	1.00	0	0	1	1	0	1	1	1	0	1	1
1	1	74	2	144	56.2	-4.90	27.0	141	79	212	57	155.00	6.00	1	1.00	1	0	1	0	1	1	1	0	0	1	1
1	1	65	1	161	55.6	-2.70	21.5	112	73	179	77	102.00	5.00	0	1.00	0	0	1	0	1	0	1	1	1	1	1
0	0	66	2	157	53.0	-.90	21.4	111	74	181	48	133.00	5.30	1	1.00	1	0	1	1	1	1	1	1	1	1	1
1	1	70	1	164	65.3	-4.00	24.2	162	80	219	44	175.00	6.20	1	1.00	1	1	1	0	1	1	1	1	0	1	0
1	1	64	2	137	36.9	-1.80	19.6	139	85	255	78	177.00	5.10	1	1.00	1	0	1	1	1	1	1	1	1	0	1
1	1	77	2	141	43.1	-.90	21.6	154	70	166	45	121.00	5.40	1	1.00	1	0	1	0	1	0	0	1	0	1	1
1	1	60	1	171	72.3	-.10	24.8	137	91	225	34	191.00	5.60	0	1.00	1	0	1	1	1	1	1	1	0	0	1
1	1	59	2	159	54.4	-1.20	21.5	102	64	266	78	188.00	5.70	1	1.00	1	0	1	0	0	0	0	1	1	0	1
0	0	80	2	145	49.7	1.30	23.6	133	76	173	72	101.00	5.50	1	1.00	1	0	1	0	1	1	1	1	0	1	1

1	0	31	1	177	85.8	.60	27.3	111	63	181	84	97.00	5.10	1	1.00	1	0	1	1	1	1	1	0	1	1	1
0	0	26	2	164	89.4	4.30	33.1	132	77	157	73	84.00	5.10	1	1.00	1	0	1	0	0	0	1	0	1	1	1
1	1	34	1	154	57.1	-4.30	24.1	114	66	198	47	151.00	5.80	0	1.00	1	0	1	0	1	0	0	1	1	1	1
1	1	29	2	146	42.6	-2.80	20.0	111	60	236	89	147.00	4.90	1	1.00	1	0	1	0	1	0	1	1	1	0	1
0	0	77	2	149	46.2	-3.20	20.7	138	76	183	83	100.00	5.50	1	1.00	1	0	1	1	1	0	0	1	0	1	1
0	0	64	2	148	54.7	-1.60	25.1	132	81	216	90	126.00	4.90	1	1.00	0	1	1	1	1	1	1	0	0	0	1
1	1	57	2	155	53.4	-.70	22.3	115	67	241	89	152.00	5.70	1	1.00	1	0	1	1	1	1	1	1	1	0	1
0	0	75	1	168	58.9	-.70	20.8	89	53	148	45	103.00	5.80	0	1.00	1	1	1	1	1	1	1	1	1	1	1
0	0	66	2	160	57.2	2.20	22.2	138	79	187	48	139.00	5.30	1	1.00	1	0	1	0	1	0	0	1	1	1	1
1	1	74	2	154	46.4	-.60	19.5	100	53	199	91	108.00	5.90	1	1.00	0	0	1	1	1	1	1	1	1	1	1
1	1	40	2	161	55.1	-.10	21.2	93	55	153	73	80.00	5.40	1	1.00	0	1	1	1	1	1	1	1	1	1	1
0	0	56	1	164	69.1	-.40	25.8	130	84	151	48	103.00	5.90	1	1.00	0	0	1	0	1	0	0	0	1	1	1
1	1	55	2	150	43.8	-.70	19.4	118	76	197	68	129.00	5.60	1	1.00	1	1	1	0	1	0	0	1	1	1	1
1	1	73	2	143	46.5	-.20	22.8	130	70	194	58	136.00	5.90	1	1.00	1	0	1	1	1	0	0	1	1	1	0
0	1	77	2	152	52.0	-2.40	22.5	143	82	198	61	137.00	5.60	1	1.00	1	0	1	1	0	1	0	1	0	1	1
1	1	36	1	169	63.2	-.60	22.2	124	65	220	62	158.00	5.30	1	1.00	0	1	1	1	1	0	0	1	1	0	1
1	1	77	2	152	60.7	-1.80	26.3	136	79	203	55	148.00	5.50	1	1.00	1	1	1	0	1	0	1	0	0	1	1
0	0	69	2	153	45.9	1.90	19.7	99	59	249	66	183.00	5.80	1	1.00	1	0	1	0	0	0	1	1	1	0	1
1	1	71	1	166	63.2	-2.90	22.9	160	78	194	57	137.00	5.50	0	1.00	0	1	1	0	0	0	1	1	0	1	1
1	1	67	2	147	51.8	-1.50	23.8	98	59	174	62	112.00	5.80	1	1.00	1	0	1	0	1	1	1	1	1	0	1
0	0	70	2	155	50.0	-1.70	20.9	136	76	173	57	116.00	5.10	1	1.00	1	0	1	1	1	1	1	1	1	1	1
0	1	73	2	146	57.2	-2.30	26.7	131	73	188	67	121.00	6.90	1	1.00	1	1	1	1	1	1	0	0	0	1	0
1	1	77	1	162	47.6	-.70	18.0	102	66	162	74	88.00	5.70	1	1.00	1	0	1	1	1	0	1	1	0	1	1
1	1	70	1	174	75.0	-3.20	24.7	110	66	197	67	130.00	5.80	1	1.00	1	1	1	0	0	0	1	1	1	1	1
0	0	77	2	149	44.9	.50	20.1	146	78	211	65	146.00	5.60	1	1.00	1	0	1	0	1	0	1	1	0	1	1
1	1	74	2	143	46.9	1.30	23.0	131	79	230	71	159.00	5.40	1	1.00	1	0	1	1	1	1	1	1	1	0	1
1	1	55	2	146	56.5	-1.50	26.6	164	95	201	47	154.00	5.80	1	1.00	1	1	1	1	1	1	1	0	0	1	1
0	1	63	1	168	55.6	-1.30	19.7	124	72	277	68	209.00	5.30	0	1.00	1	1	1	1	1	1	1	1	1	0	1
1	1	48	1	175	85.9	-2.80	28.1	130	74	200	51	149.00	6.20	0	2.00	0	0	1	0	0	0	0	0	0	1	1
1	1	72	2	160	50.3	1.00	19.7	135	77	187	69	118.00	4.90	1	1.00	1	0	1	1	1	1	1	1	0	1	1
1	1	77	2	150	63.1	-.10	28.0	146	80	134	58	76.00	5.40	1	1.00	1	0	1	1	1	1	0	0	0	1	1
0	1	64	2	159	63.2	-1.20	24.9	112	62	225	67	158.00	5.50	1	1.00	1	0	1	0	1	0	0	1	0	0	1
0	0	38	2	158	62.6	.80	25.2	121	63	163	68	95.00	5.30	1	1.00	1	0	1	0	1	0	1	0	1	1	1
1	1	70	2	152	63.5	-.10	27.6	129	75	221	68	153.00	5.20	1	1.00	1	0	1	1	1	1	1	0	0	0	1
1	1	60	2	147	48.1	-1.80	22.2	139	80	218	45	173.00	5.50	1	1.00	1	0	1	0	0	0	1	1	1	1	1
1	1	83	2	136	40.4	-.50	21.8	130	75	164	75	89.00	5.60	1	1.00	1	1	1	1	1	1	1	1	0	1	1
0	0	75	2	149	52.9	.70	23.9	132	86	136	68	68.00	5.70	1	1.00	1	0	0	0	1	0	0	1	0	1	1
0	0	56	2	147	54.8	1.20	25.3	137	82	244	52	192.00	5.20	1	1.00	1	0	1	0	0	0	0	0	1	0	1
1	1	76	2	144	43.2	-.50	20.7	119	64	225	67	158.00	5.30	1	1.00	1	0	1	1	1	0	0	1	1	0	1

1	1	67	2	153	54.5	-.70	23.4	112	69	186	69	117.00	5.30	1	1.00	0	0	1	0	1	1	1	1	0	1	1
1	1	68	2	156	52.3	-.30	21.4	121	66	149	56	93.00	6.80	1	1.00	1	0	1	1	1	0	1	1	0	0	0
1	1	72	1	155	55.1	-.40	22.9	143	84	188	96	92.00	5.40	1	1.00	0	0	1	1	1	1	1	1	0	1	1
1	1	73	1	164	64.7	-2.80	24.0	130	65	167	57	110.00	6.10	1	1.00	0	0	1	1	1	1	1	1	0	1	0
1	1	74	1	158	56.2	-2.90	22.5	110	59	175	54	121.00	5.50	1	1.00	1	1	1	1	1	1	1	1	1	1	1
1	1	60	2	143	54.7	-.90	26.7	128	54	192	68	124.00	6.20	1	1.00	1	0	1	1	1	0	1	0	0	0	0
0	1	46	1	161	73.0	-2.20	28.3	105	74	161	64	97.00	5.30	0	2.00	0	0	1	1	1	1	1	0	1	1	1
0	0	64	2	150	62.9	-.80	28.0	132	75	278	63	215.00	5.20	1	1.00	1	0	1	0	0	0	0	0	1	0	1
1	1	64	2	152	55.8	.70	24.0	155	84	183	76	107.00	5.60	1	1.00	1	0	1	1	1	1	1	1	0	1	1
1	1	35	2	162	53.1	3.50	20.3	112	70	270	57	213.00	5.30	0	1.00	1	0	1	0	1	1	1	1	1	0	1
0	1	51	1	177	74.6	4.80	23.8	137	80	195	37	158.00	5.60	0	1.00	0	0	1	0	1	0	0	1	1	0	1
0	0	59	2	158	59.7	-2.20	24.0	120	78	226	69	157.00	6.80	1	1.00	1	1	1	1	1	0	1	1	1	0	0
1	1	68	1	165	72.0	-3.20	26.3	146	88	204	61	143.00	5.50	1	1.00	1	1	0	1	1	1	1	0	0	1	1
1	1	70	2	151	59.1	-1.10	25.9	134	76	189	86	103.00	5.20	1	1.00	1	0	1	1	1	0	0	0	0	0	1
1	1	71	1	165	80.1	-4.90	29.5	102	57	227	55	172.00	6.70	1	1.00	0	0	1	1	1	1	1	0	0	0	0
1	1	71	1	164	54.7	-.20	20.3	141	84	209	87	122.00	5.70	1	1.00	0	1	1	0	1	1	1	1	0	1	1
0	0	63	2	146	39.3	.40	18.4	156	101	199	96	103.00	5.30	1	1.00	1	0	1	0	1	0	0	1	0	1	1
1	1	74	1	166	66.1	-2.70	24.0	120	67	141	45	96.00	5.50	1	1.00	1	0	1	1	1	1	1	1	0	1	1
0	0	42	2	156	69.5	-2.10	28.6	154	95	249	45	204.00	5.90	1	1.00	1	1	1	0	0	0	0	0	0	0	1
1	1	64	2	163	50.4	-.40	19.0	157	89	245	86	159.00	5.40	1	1.00	1	0	1	0	0	1	0	1	0	0	1
1	1	45	2	168	67.6	3.50	23.9	102	71	247	69	178.00	5.30	1	1.00	1	0	0	1	1	0	0	1	1	0	1
0	0	61	2	145	44.5	-1.40	21.3	114	80	251	77	174.00	6.00	1	1.00	1	0	1	0	0	1	1	1	1	0	1
0	0	72	2	142	50.7	-1.80	25.0	148	88	206	47	159.00	5.80	1	1.00	1	0	1	0	1	0	0	0	0	1	1
0	0	35	2	164	62.1	4.20	23.0	109	65	215	89	126.00	5.40	1	1.00	1	0	0	1	1	0	0	1	1	1	1
0	0	41	2	155	49.4	.70	20.5	114	67	169	72	97.00	4.90	0	1.00	1	1	1	0	1	0	0	1	1	1	1
0	0	73	2	149	74.1	3.50	33.4	160	84	212	62	150.00	7.80	1	1.00	1	0	1	1	1	1	0	0	0	0	0
0	0	29	2	160	47.3	.10	18.4	99	56	232	98	134.00	5.20	0	1.00	1	0	1	0	0	1	1	1	1	0	1
0	0	62	2	154	56.3	-1.40	23.8	107	71	216	60	156.00	5.70	1	1.00	1	0	1	1	1	1	1	1	1	1	1
0	0	48	1	166	49.6	2.30	17.9	108	73	208	76	132.00	5.20	1	1.00	0	0	1	1	1	1	1	1	1	1	1
0	0	69	1	156	60.2	-.80	24.6	139	73	212	49	163.00	6.10	0	1.00	0	1	1	1	1	1	1	1	0	1	0
1	1	41	2	155	49.1	-3.40	20.4	95	66	237	93	144.00	4.70	0	1.00	0	0	1	0	1	1	1	1	1	0	1
0	0	56	1	175	75.7	-2.20	24.6	128	70	186	71	115.00	5.30	1	1.00	0	0	1	0	1	0	1	1	0	1	1
1	1	76	1	169	68.8	-1.30	24.0	127	72	188	62	126.00	5.70	1	1.00	1	0	1	0	1	0	0	1	1	1	1
1	1	49	2	144	48.1	-.80	23.3	119	75	292	73	219.00	6.00	1	1.00	1	0	1	0	1	1	0	1	1	0	1
1	1	59	2	155	52.7	1.00	22.0	130	85	284	73	211.00	6.10	1	1.00	1	0	1	1	1	1	1	1	1	0	1
1	1	73	1	161	55.7	.60	21.5	117	64	196	51	145.00	6.30	1	1.00	0	1	1	0	1	1	1	1	0	1	1
0	0	51	2	165	64.0	-.30	23.5	125	74	168	58	110.00	5.90	1	1.00	1	0	1	0	0	0	1	1	1	1	1
0	0	65	2	151	49.6	1.70	21.6	96	54	211	41	170.00	4.90	1	1.00	1	1	1	0	1	0	0	1	1	1	1
0	0	71	2	144	48.4	2.50	23.4	126	69	217	48	169.00	6.20	1	1.00	1	0	1	0	1	1	0	1	1	0	1

0	0	44	2	159	66.4	1.70	26.2	123	84	162	76	86.00	5.30	1	1.00	1	0	1	0	0	0	0	0	1	1	1
0	0	54	1	161	68.1	1.60	26.4	108	65	211	62	149.00	5.60	1	1.00	1	0	1	0	1	0	0	0	1	1	1
1	1	61	2	162	65.3	-1.00	24.8	112	75	202	84	118.00	5.30	1	1.00	1	0	1	0	1	0	0	1	1	1	1
1	1	78	1	160	59.0	-3.40	23.1	122	55	176	46	130.00	5.70	1	1.00	1	0	1	0	1	0	0	1	0	1	0
1	1	67	2	154	55.4	-.50	23.3	142	75	192	98	94.00	5.30	1	1.00	0	0	1	0	1	1	1	1	0	1	1
0	0	64	2	159	48.6	.30	19.1	115	62	176	46	130.00	5.30	1	1.00	1	0	1	1	1	1	0	1	1	1	1
0	0	38	2	153	42.2	-.20	18.1	106	71	218	93	125.00	5.80	1	1.00	1	0	1	0	0	1	1	1	1	1	1
1	1	70	1	181	73.5	-1.90	22.3	126	78	198	63	135.00	5.20	1	1.00	0	1	1	1	1	1	1	1	1	1	1
0	0	83	2	139	42.6	.60	21.9	110	59	160	75	85.00	5.10	1	1.00	1	0	1	0	1	0	1	1	1	0	1
1	1	64	2	150	51.4	-1.00	22.7	141	73	230	97	133.00	5.80	1	1.00	1	0	1	0	1	0	0	1	0	0	1
1	1	62	2	152	40.0	1.00	17.4	112	70	219	68	151.00	5.80	1	1.00	0	0	1	1	1	1	0	1	1	1	1
1	1	68	1	159	66.0	-3.30	26.1	138	87	193	49	144.00	5.60	1	1.00	0	0	1	0	0	0	1	0	0	1	1
1	1	72	2	153	43.4	-2.60	18.6	110	75	192	70	122.00	5.70	1	1.00	0	0	1	1	1	1	1	1	1	1	1
0	0	60	2	159	53.0	2.60	21.0	134	71	267	91	176.00	5.50	1	1.00	1	0	1	0	0	0	0	1	1	0	1
1	1	73	1	165	66.6	-5.60	24.5	123	88	132	46	86.00	5.60	1	1.00	1	0	1	1	1	1	1	1	0	1	1
0	0	70	2	154	50.3	-1.60	21.1	109	58	190	58	132.00	5.70	1	1.00	1	0	1	0	0	0	1	1	1	1	1
1	1	77	2	143	44.1	-1.30	21.5	150	84	234	83	151.00	5.00	1	1.00	1	0	1	1	1	1	1	1	0	0	1
1	1	76	2	141	46.7	-2.10	23.4	134	94	219	90	129.00	6.20	1	1.00	1	0	1	1	1	1	0	1	0	1	1
1	1	63	2	144	49.1	.40	23.8	126	76	212	67	145.00	5.40	1	1.00	1	1	1	1	1	1	1	1	1	1	1
1	1	32	2	155	48.3	.20	20.1	131	79	205	105	100.00	5.10	1	1.00	0	1	1	1	1	1	1	1	1	1	1
1	1	69	1	162	52.1	5.40	19.9	132	70	159	53	106.00	5.30	1	1.00	0	0	1	1	1	0	1	1	0	1	1
0	0	65	2	154	52.1	-1.40	22.0	143	89	219	69	150.00	5.60	1	1.00	1	0	1	0	1	1	1	1	0	1	1
0	0	72	2	151	47.8	-.40	20.9	126	72	193	55	138.00	5.40	1	1.00	1	0	1	0	0	1	0	1	0	0	1
1	1	67	2	149	46.8	.60	21.0	138	78	232	91	141.00	5.80	1	1.00	1	1	1	1	1	0	1	1	1	0	1
1	1	38	2	163	53.9	3.10	20.2	118	81	225	108	117.00	5.20	1	1.00	1	1	1	1	1	1	1	1	1	0	1
0	0	70	1	166	50.8	2.90	18.4	131	80	238	99	139.00	7.10	0	1.00	0	0	1	0	0	1	1	1	1	0	0
1	1	73	1	158	68.9	.90	27.5	130	73	187	65	122.00	6.20	0	1.00	1	0	1	0	1	0	1	0	1	1	0
1	1	71	1	163	68.6	.60	25.9	105	68	197	60	137.00	5.40	1	1.00	0	0	1	1	1	1	1	0	1	1	1
1	1	30	2	143	60.9	-.10	29.6	114	64	195	67	128.00	5.20	1	1.00	1	0	1	1	1	1	1	0	1	1	1
1	1	83	2	141	46.1	-1.50	23.1	143	84	279	92	187.00	6.10	1	1.00	1	1	1	1	1	1	1	1	0	0	1
1	1	72	2	150	60.4	1.70	26.8	148	86	222	50	172.00	5.90	1	1.00	1	1	1	1	1	1	1	0	0	0	1
1	1	71	1	162	54.9	.60	21.0	134	78	197	65	132.00	5.60	0	1.00	0	0	1	0	1	1	1	1	1	1	1
1	1	68	1	168	73.6	-.50	26.2	127	81	212	61	151.00	5.20	1	1.00	0	1	1	1	1	1	1	0	1	1	1
1	1	75	2	151	75.1	-.60	32.8	152	82	199	64	135.00	5.80	1	1.00	1	0	1	1	1	1	1	0	0	1	1
1	1	48	2	155	51.8	-2.00	21.6	129	80	183	76	107.00	5.30	1	1.00	0	0	1	0	1	1	1	1	1	1	1
1	1	68	2	141	46.8	-1.80	23.4	127	71	217	62	155.00	5.50	1	1.00	0	1	1	0	1	0	1	1	0	1	1
0	0	33	2	153	69.5	4.80	29.8	128	79	195	58	137.00	5.20	1	1.00	1	0	1	1	1	0	1	0	1	1	1
1	1	73	1	171	69.3	-8.50	23.6	107	61	214	44	170.00	5.30	1	1.00	1	0	1	0	1	1	1	1	0	1	1
1	1	37	2	156	57.8	-.20	23.6	107	66	182	73	109.00	5.40	1	1.00	1	0	1	0	1	0	0	1	1	1	1

1	1	80	2	153	48.3	.70	20.6	149	85	231	67	164.00	5.50	1	1.00	1	0	1	1	1	1	1	1	0	0	1
1	1	59	1	171	64.4	-2.10	22.0	125	75	185	44	141.00	5.50	1	1.00	0	0	1	0	0	0	1	1	1	1	1
1	1	65	2	153	52.8	-1.10	22.5	102	58	205	70	135.00	5.80	1	1.00	1	1	1	0	0	1	1	1	1	0	1
1	1	40	2	159	59.1	.10	23.4	109	62	199	42	157.00	5.10	1	1.00	1	0	1	1	0	0	1	1	1	1	1
1	1	60	2	141	47.8	2.30	23.9	118	72	194	69	125.00	5.50	1	1.00	1	0	1	0	1	0	0	1	1	1	1
1	1	82	2	136	35.4	-.20	19.1	156	78	284	59	225.00	5.50	1	1.00	1	0	1	1	1	1	1	1	0	0	1
1	1	67	1	160	49.7	-.50	19.4	123	74	219	60	159.00	4.70	0	1.00	0	0	1	1	1	1	1	1	0	1	1
0	0	62	2	149	52.9	-1.20	23.7	110	58	202	72	130.00	5.40	1	1.00	1	0	1	0	1	0	0	1	1	0	1
0	0	66	2	147	44.9	-2.00	20.6	180	104	265	49	216.00	6.10	1	1.00	1	0	1	1	0	1	1	1	0	0	0
0	0	64	2	162	69.3	2.40	26.3	137	83	222	40	182.00	6.30	1	1.00	1	0	1	0	1	0	0	0	1	0	0
1	1	70	1	171	68.5	.10	23.4	122	69	131	40	91.00	7.20	1	1.00	0	0	1	0	1	0	1	1	0	1	0
1	1	78	1	156	61.1	-2.00	25.2	129	63	182	48	134.00	5.20	1	1.00	1	0	1	0	1	1	1	0	1	1	1
1	1	66	2	149	61.6	-1.50	27.5	161	93	241	76	165.00	6.00	1	1.00	1	0	1	1	1	1	1	0	0	0	1
1	1	78	2	150	55.6	-1.30	24.7	148	85	202	85	117.00	6.40	1	1.00	1	0	1	1	1	1	1	1	0	1	1
1	1	66	2	147	40.5	1.10	18.7	160	86	184	56	128.00	5.00	1	1.00	1	0	1	1	1	1	1	1	0	1	1
1	1	64	2	155	54.7	1.90	22.6	99	62	213	70	143.00	4.90	1	1.00	1	0	1	1	1	1	1	1	1	1	1
1	1	77	1	154	60.7	-.40	25.6	122	75	223	51	172.00	5.10	1	1.00	1	0	1	1	1	0	1	0	1	0	1
1	1	79	1	154	52.1	.10	21.8	118	70	205	73	132.00	5.80	1	1.00	0	0	1	1	1	1	0	1	1	1	1
0	0	50	2	151	66.7	-1.70	29.0	123	83	242	106	136.00	6.20	1	1.00	1	0	1	1	1	1	1	0	0	0	1
1	1	42	1	170	72.4	1.40	25.1	129	80	181	45	136.00	5.50	0	1.00	1	1	1	0	1	1	1	0	1	1	1
1	1	77	1	153	58.9	-.50	25.1	114	59	235	44	191.00	5.90	1	1.00	1	0	1	1	1	1	1	0	0	0	1
0	0	62	2	156	52.0	2.80	21.5	116	77	211	62	149.00	5.60	1	1.00	1	0	1	1	0	1	0	1	0	1	1
1	1	69	2	151	48.6	1.10	21.3	96	53	161	64	97.00	6.00	1	1.00	1	0	1	1	1	1	0	1	1	1	1
0	0	82	2	145	58.3	-.90	27.8	121	59	270	43	227.00	6.00	1	1.00	1	0	1	0	1	1	1	0	0	0	1
1	1	79	2	140	34.7	1.60	17.8	131	67	229	86	143.00	5.80	1	1.00	1	0	1	1	1	0	1	1	1	0	1
0	0	67	2	145	65.0	.70	31.0	114	73	217	52	165.00	6.00	1	1.00	1	0	1	0	1	0	0	0	0	0	1
1	1	58	1	167	62.3	.40	22.4	89	66	225	110	115.00	5.00	1	1.00	0	0	1	1	1	0	1	1	1	0	1
0	0	85	1	158	61.3	-.90	24.5	151	93	234	61	173.00	5.50	1	1.00	0	1	1	1	1	1	1	1	0	0	1
1	1	70	2	152	42.4	2.30	18.4	98	54	220	101	119.00	5.40	1	1.00	1	0	1	1	1	1	1	1	1	0	1
1	1	69	2	148	43.8	2.30	20.1	114	70	227	61	166.00	6.00	1	1.00	1	0	1	0	1	0	0	1	1	0	1
1	1	76	1	160	65.4	-5.80	25.4	143	81	241	46	195.00	6.80	1	1.00	0	1	1	1	1	1	1	0	0	0	0
0	0	57	2	142	53.4	-.60	26.5	108	68	216	32	184.00	6.30	1	1.00	1	0	1	0	1	0	1	0	0	0	1
1	1	70	2	142	44.0	1.00	21.7	133	70	181	66	115.00	6.20	1	1.00	1	0	1	0	1	1	0	1	1	1	1
0	0	61	1	173	74.2	3.00	24.8	130	78	190	56	134.00	5.60	0	1.00	0	0	1	1	1	1	1	1	0	1	1
0	0	61	2	154	64.5	2.50	27.2	126	84	155	64	91.00	5.80	1	1.00	1	0	1	0	1	0	1	0	0	1	1
0	0	73	1	170	70.0	1.00	24.1	154	95	209	53	156.00	6.10	1	1.00	0	0	1	1	1	0	1	1	0	1	1
1	1	64	2	153	47.8	1.70	20.5	130	83	205	92	113.00	6.00	1	1.00	1	0	1	0	0	0	0	1	1	0	1
0	0	67	1	164	50.9	2.20	18.9	118	80	189	69	120.00	5.50	1	1.00	0	0	1	0	0	0	0	1	1	1	1
1	1	59	1	170	73.2	6.20	25.2	139	99	189	33	156.00	6.60	1	1.00	1	0	1	0	0	0	0	0	0	0	0

0	0	79	2	150	58.1	-1.70	25.9	113	62	208	62	146.00	5.30	1	1.00	1	1	1	1	1	0	0	0	1	1	1
1	1	78	2	154	60.1	-1.60	25.3	121	76	202	63	139.00	6.00	1	1.00	1	1	1	0	1	0	0	0	1	0	1
1	1	69	1	168	70.2	-.60	24.7	152	84	146	57	89.00	5.70	1	1.00	0	0	1	1	1	0	1	1	0	1	1
1	1	31	2	158	85.8	1.20	34.3	157	84	222	64	158.00	5.60	1	1.00	1	0	1	1	0	1	1	0	0	0	1
1	1	64	1	160	62.4	-2.60	24.3	135	87	194	72	122.00	5.50	1	1.00	0	0	1	0	1	0	0	1	1	1	1
1	1	59	2	160	53.8	2.80	21.0	126	72	190	68	122.00	5.50	1	1.00	1	0	1	1	1	0	0	1	0	1	1
1	1	66	1	170	88.9	-4.90	30.7	123	71	139	43	96.00	5.50	1	1.00	0	0	1	1	1	0	0	0	1	1	1
1	1	78	2	149	57.5	1.70	25.7	155	85	215	83	132.00	5.30	1	1.00	1	0	1	1	1	0	1	0	0	1	1
1	1	64	1	158	64.0	.90	25.7	170	96	183	78	105.00	5.90	1	1.00	0	0	1	0	1	0	1	0	0	1	1
0	0	68	1	153	49.2	.80	20.9	119	74	239	87	152.00	5.60	0	1.00	1	1	1	1	1	0	1	1	1	0	1
1	1	60	1	165	61.3	.30	22.4	143	90	239	101	138.00	5.80	1	1.00	0	1	1	1	1	0	0	1	0	0	1
0	0	73	1	166	66.6	2.90	24.2	135	84	170	40	130.00	5.70	1	1.00	0	0	1	1	1	1	1	1	0	1	1
0	0	63	2	155	53.1	2.10	22.2	127	69	223	62	161.00	6.70	1	1.00	1	0	1	1	1	1	0	1	0	0	0
1	1	63	2	150	58.0	-.20	25.8	145	92	176	60	116.00	5.60	1	1.00	1	0	1	1	1	1	1	0	0	1	1
0	0	58	2	149	51.2	1.30	23.1	116	60	203	48	155.00	5.40	1	1.00	1	0	1	0	1	0	0	1	1	1	1
0	0	64	1	159	55.4	2.30	21.9	130	76	205	71	134.00	5.30	0	1.00	0	0	0	0	1	0	0	1	1	1	0
0	0	62	2	153	46.4	-.40	19.8	121	73	239	82	157.00	5.50	1	1.00	1	0	1	0	0	0	0	1	0	0	1
1	1	62	1	169	70.3	-2.30	24.4	115	70	175	31	144.00	5.70	1	1.00	0	1	1	1	1	1	1	1	1	0	1
0	0	67	2	153	45.6	-.10	19.4	134	71	239	62	177.00	6.00	1	1.00	1	0	1	0	1	0	0	1	1	0	1
1	1	61	2	151	50.6	-1.10	22.1	117	62	184	79	105.00	5.90	1	1.00	1	1	1	0	1	1	0	1	1	1	1
1	1	61	1	165	67.0	.20	24.5	100	62	185	43	142.00	5.60	0	1.00	1	0	1	0	1	1	0	1	1	1	1
1	1	65	1	170	69.1	-1.20	23.8	142	82	165	35	130.00	5.20	1	1.00	1	0	1	1	1	1	1	1	0	0	1
1	1	74	2	153	41.6	1.30	17.7	174	88	201	75	126.00	5.40	1	1.00	1	0	1	0	1	1	1	1	0	1	1
1	1	47	1	176	71.7	-.20	23.0	111	75	240	40	200.00	5.20	1	1.00	1	0	0	0	0	0	0	1	1	0	1
1	1	70	1	158	66.9	-.90	26.9	104	71	174	47	127.00	5.70	1	1.00	1	0	1	0	1	0	1	0	1	0	1
0	0	55	2	163	58.1	3.50	21.9	114	71	231	62	169.00	5.20	1	1.00	1	0	1	0	1	0	0	1	1	0	1
1	1	80	2	136	39.2	-1.20	21.0	154	85	170	73	97.00	5.90	1	1.00	1	1	1	1	1	1	1	1	0	1	0
0	0	76	2	149	42.5	-.50	19.2	127	92	234	79	155.00	5.50	1	1.00	1	0	1	0	1	0	1	1	0	0	1
1	1	76	2	164	71.9	-.50	26.7	127	77	232	56	176.00	5.60	1	1.00	1	1	1	1	1	1	1	0	1	0	1
0	0	76	1	161	73.6	-1.00	28.4	130	79	226	51	175.00	6.20	0	1.00	1	0	1	0	1	0	1	0	0	0	1
0	0	51	2	154	45.0	-1.00	19.0	128	87	192	59	133.00	4.90	1	1.00	1	0	1	1	0	0	1	1	1	1	1
1	1	61	2	135	38.9	.20	21.2	149	92	158	61	97.00	5.90	1	1.00	1	0	1	0	1	1	1	1	0	1	1
1	1	58	2	157	68.2	1.60	27.7	126	72	200	75	125.00	6.10	1	1.00	1	1	1	0	1	0	0	0	1	1	1
1	1	63	1	170	70.7	-.80	24.5	125	85	213	58	155.00	5.80	1	1.00	1	0	1	1	1	1	1	1	0	0	1
1	1	41	2	161	56.4	-1.00	21.6	106	62	199	83	116.00	5.50	1	1.00	0	0	1	0	1	0	0	1	1	1	1
1	1	59	2	155	31.5	-2.50	13.0	98	61	167	74	93.00	5.10	1	1.00	0	0	1	0	1	0	1	1	1	1	1
1	1	50	1	158	70.7	10.00	28.4	136	85	236	77	159.00	5.50	1	1.00	1	1	1	1	1	1	1	0	1	0	1
1	1	61	2	151	53.7	-.70	23.7	107	74	181	62	119.00	5.40	1	1.00	1	0	1	0	1	0	0	1	0	1	1
1	1	80	2	143	51.8	-.80	25.1	170	71	197	52	145.00	6.70	1	1.00	1	1	1	1	1	1	1	0	0	1	0

0	0	58	1	169	57.2	-.40	20.0	134	90	172	79	93.00	5.40	0	1.00	0	1	1	0	1	1	1	1	0	1	0
1	1	59	2	164	58.5	.20	21.8	98	63	246	79	167.00	6.00	1	1.00	1	0	1	1	1	1	1	1	1	0	1
1	1	74	2	148	63.7	-2.80	28.9	145	83	209	40	169.00	6.80	1	1.00	1	0	1	1	1	1	1	0	0	1	0
1	1	51	1	168	75.6	-2.40	26.8	129	74	282	39	243.00	7.80	0	1.00	0	0	1	0	1	1	1	0	1	0	0
1	1	71	1	164	62.9	1.20	23.5	143	80	204	51	153.00	5.80	1	1.00	1	0	1	1	1	1	1	1	0	1	1
1	1	80	1	169	66.8	.50	23.2	173	91	198	56	142.00	5.70	1	1.00	0	0	1	0	1	1	1	1	0	0	1
1	1	82	2	131	51.9	-1.60	30.1	155	76	207	57	150.00	5.50	1	1.00	1	0	1	1	1	1	1	0	0	1	1
1	1	71	1	169	56.9	-.30	19.9	111	65	178	71	107.00	5.90	0	1.00	0	0	1	0	1	1	1	1	1	1	1
1	1	83	2	146	70.4	-3.00	33.0	172	72	231	49	182.00	6.90	1	1.00	1	1	1	1	1	0	1	0	0	0	0
1	1	70	2	152	53.4	1.40	23.0	136	76	207	100	107.00	5.40	1	1.00	1	1	1	1	1	1	1	1	1	1	1
1	1	74	2	139	47.2	.60	24.5	145	73	165	74	91.00	5.70	1	1.00	1	0	1	0	1	0	0	1	0	1	1
1	1	62	1	163	54.6	-.70	20.4	134	78	182	75	107.00	5.50	0	1.00	0	0	1	0	1	1	1	1	0	1	1
0	0	71	2	150	65.6	-1.40	29.3	118	68	252	53	199.00	5.80	1	1.00	1	0	1	0	1	1	1	0	1	0	1
0	0	74	1	162	68.5	2.50	26.1	108	61	175	75	100.00	5.20	1	1.00	0	0	1	1	1	1	1	0	1	1	1
1	1	68	2	133	49.3	.50	27.9	146	85	236	85	151.00	5.80	1	1.00	1	1	1	0	1	0	1	0	0	0	1
1	1	76	1	161	60.7	.20	23.5	148	72	172	76	96.00	5.50	1	1.00	0	0	1	1	1	1	1	1	0	1	1
1	1	75	1	152	58.1	5.80	25.0	109	58	204	74	130.00	5.20	0	1.00	0	1	1	1	1	1	1	0	0	1	1
1	1	68	1	151	45.9	-.30	20.0	182	104	192	51	141.00	6.80	1	1.00	0	0	1	0	1	0	0	1	0	1	0
1	1	60	2	155	55.3	-1.00	23.1	112	64	251	58	193.00	5.80	1	1.00	0	1	1	0	1	1	1	1	1	0	1
1	1	85	2	146	56.2	-2.10	26.4	133	62	201	40	161.00	6.80	1	1.00	1	1	1	1	1	1	1	0	0	1	0
1	1	77	2	148	63.8	-2.30	29.1	177	94	201	53	148.00	5.40	1	1.00	1	0	1	1	1	1	1	0	0	1	1
1	1	63	2	155	55.3	.30	23.1	109	60	286	55	231.00	5.70	1	1.00	1	0	1	0	0	1	1	1	1	0	1
0	0	74	2	156	48.4	1.00	20.0	116	66	214	98	116.00	5.50	1	1.00	1	0	1	0	1	1	0	1	1	1	1
1	1	41	1	158	68.9	-2.60	27.7	134	85	227	58	169.00	5.60	0	1.00	0	1	1	1	1	1	1	0	1	0	1
1	1	69	2	143	45.7	-1.30	22.4	133	71	306	46	260.00	5.60	1	1.00	1	1	1	0	1	1	0	1	1	0	1
0	0	63	2	151	47.4	1.00	20.6	132	76	266	98	168.00	5.90	1	1.00	1	0	1	1	1	1	0	1	1	0	1
1	1	80	2	147	39.8	.40	18.4	123	74	240	67	173.00	5.60	1	1.00	1	0	1	1	1	1	1	1	1	0	1
0	1	70	2	148	50.6	-.10	23.0	118	67	111	27	84.00	5.90	1	1.00	1	1	1	1	1	0	1	1	0	0	1
1	1	57	1	171	58.1	1.50	19.9	148	95	186	70	116.00	5.40	1	1.00	0	0	1	1	1	1	1	1	0	1	1
1	1	73	2	142	55.5	-2.40	27.4	111	67	197	52	145.00	5.60	1	1.00	0	0	1	1	1	1	1	0	1	1	1
1	1	72	2	147	62.5	-1.10	28.7	156	88	220	51	169.00	5.30	1	1.00	1	0	1	1	1	1	0	0	0	0	1
1	1	70	2	145	41.5	.50	19.6	121	70	193	76	117.00	5.40	1	1.00	0	0	1	0	1	0	0	1	1	1	1
0	0	71	2	152	50.7	1.50	21.9	101	64	188	58	130.00	6.10	1	1.00	0	1	1	1	1	0	1	1	1	1	1
1	1	70	2	148	49.5	.00	22.5	139	80	313	82	231.00	5.50	1	1.00	1	0	1	0	1	0	0	1	1	0	1
1	1	35	1	166	61.3	.60	22.2	112	73	194	58	136.00	5.30	1	1.00	1	0	0	1	1	1	1	1	1	1	1
0	0	42	2	164	50.6	-.40	18.7	111	62	208	70	138.00	5.20	1	1.00	0	1	1	0	1	1	1	1	1	1	1
1	1	36	2	153	39.7	3.00	17.0	93	56	181	63	118.00	5.40	1	1.00	1	0	1	0	0	0	1	1	1	1	1
0	0	71	1	160	70.2	3.10	27.3	109	77	165	60	105.00	4.20	1	1.00	1	0	1	0	1	1	1	0	1	1	1
0	0	33	2	158	46.7	.70	18.6	101	66	161	71	90.00	5.00	1	1.00	1	0	1	0	1	0	1	1	1	1	1

0	0	51	1	168	65.7	1.10	23.4	113	79	218	60	158.00	5.70	1	1.00	1	1	1	0	1	1	1	1	1	1	1
0	0	48	2	158	50.8	-1.30	20.2	110	66	224	81	143.00	5.30	1	1.00	0	0	1	1	0	0	0	1	1	0	1
0	0	62	2	162	44.1	.20	16.8	102	59	188	79	109.00	5.80	1	1.00	1	0	1	0	1	0	0	1	1	1	1
0	0	40	1	183	64.1	3.20	19.2	120	86	136	92	44.00	5.60	0	1.00	0	1	0	0	0	0	1	1	1	1	1
0	0	71	2	142	42.4	.50	20.9	135	78	203	57	146.00	6.40	1	1.00	1	1	1	0	1	1	1	1	0	1	1
1	1	69	1	168	60.5	-.30	21.3	149	81	256	110	146.00	5.70	1	1.00	0	1	1	1	1	1	1	1	0	0	1
1	1	67	2	139	54.9	-1.40	28.2	139	86	163	63	100.00	6.60	1	1.00	1	0	1	1	1	1	1	0	0	1	0
1	1	51	2	163	66.1	-2.40	25.0	142	96	312	57	255.00	6.00	1	1.00	1	0	1	1	1	1	1	0	0	0	1
0	0	46	1	167	72.4	-1.80	26.0	117	68	201	61	140.00	5.90	1	1.00	0	0	1	1	1	1	1	0	1	1	1
0	0	44	2	152	42.2	3.40	18.3	102	56	169	65	104.00	4.60	1	1.00	1	1	1	1	1	1	1	1	1	1	1
1	1	64	2	156	57.3	1.80	23.6	119	75	193	52	141.00	6.70	1	1.00	1	0	1	1	1	1	0	1	1	1	0
1	1	77	2	142	47.4	.00	23.5	123	72	215	38	177.00	5.60	1	1.00	1	1	1	1	1	0	1	1	1	0	1
0	0	69	2	158	49.7	-3.70	19.9	131	72	202	67	135.00	5.50	1	1.00	1	0	1	1	1	1	0	1	0	1	1
1	1	48	2	151	75.1	.80	33.1	131	85	212	79	133.00	5.80	1	1.00	1	0	1	0	1	1	1	0	0	1	1
1	1	59	2	156	47.8	-.60	19.5	105	61	189	55	134.00	5.60	1	1.00	1	0	1	1	1	1	1	1	1	1	1
0	1	62	2	147	45.6	-.90	21.2	94	47	181	64	117.00	5.20	1	1.00	1	1	1	0	0	1	0	1	1	1	1
1	1	60	2	147	44.2	-.10	20.3	129	72	229	68	161.00	6.00	1	1.00	1	0	1	0	1	1	1	1	1	0	1
0	0	64	2	155	60.2	2.20	25.0	123	80	158	52	106.00	5.40	1	1.00	1	0	1	0	1	1	0	0	1	0	1
0	0	64	2	143	54.9	.10	26.9	146	79	229	75	154.00	5.60	1	1.00	1	0	1	0	0	1	1	0	0	0	1
1	1	69	1	172	70.2	.50	23.7	122	74	182	42	140.00	5.70	0	1.00	0	1	1	1	1	1	1	1	1	1	1
0	0	62	2	153	49.8	-.50	21.2	136	72	195	66	129.00	5.20	1	1.00	1	0	1	0	1	0	0	1	1	1	1
1	1	66	2	153	63.5	1.60	27.1	110	63	166	75	91.00	5.90	1	1.00	1	0	1	1	1	1	0	0	0	1	1
1	1	50	2	153	54.2	.50	23.2	195	108	216	63	153.00	5.40	1	1.00	1	0	1	0	0	0	0	1	0	1	1
1	1	78	2	145	50.8	-.40	24.0	125	68	217	51	166.00	5.70	1	1.00	1	0	1	0	1	0	1	1	1	1	1
1	1	61	1	171	80.6	2.90	27.4	100	61	195	36	159.00	6.00	0	1.00	0	1	1	1	1	0	0	0	1	0	1
0	0	43	2	165	58.2	3.50	21.2	138	83	163	74	89.00	5.30	1	1.00	0	0	1	0	1	1	0	1	1	1	1
0	0	63	2	156	56.3	-2.20	23.2	146	87	205	57	148.00	6.60	1	1.00	1	0	1	1	1	1	0	1	0	1	0
1	1	63	2	156	59.5	-.20	24.3	154	80	199	43	156.00	5.50	1	1.00	1	0	1	1	1	0	1	1	0	0	1
1	1	55	2	155	71.9	.10	29.8	113	70	270	98	172.00	5.90	1	1.00	1	1	1	0	1	1	1	0	1	0	1
1	1	56	2	147	46.7	1.40	21.6	121	79	191	73	118.00	5.60	1	1.00	1	0	1	1	0	1	1	1	1	0	1
1	1	66	2	151	52.5	1.70	22.9	106	68	263	63	200.00	5.70	1	1.00	1	1	1	0	1	0	1	1	0	0	1
1	1	72	2	153	52.6	.00	22.4	117	66	250	67	183.00	5.70	1	1.00	1	1	1	0	0	1	1	1	0	0	1
1	1	71	2	148	47.3	-1.20	21.6	139	79	208	67	141.00	5.60	1	1.00	1	1	1	1	1	1	0	1	1	1	1
1	1	58	2	151	56.8	.80	24.9	161	94	246	63	183.00	5.60	1	1.00	1	1	1	0	0	1	1	1	0	0	1
1	1	53	2	156	71.4	4.70	29.3	140	75	278	66	212.00	5.80	1	1.00	1	0	1	1	1	0	0	0	0	0	1
1	1	59	2	154	50.7	1.60	21.3	158	86	253	54	199.00	5.60	1	1.00	1	0	1	0	0	0	1	1	0	0	1
1	1	52	2	148	49.6	.00	22.6	127	76	197	75	122.00	5.20	1	1.00	1	1	1	0	0	0	1	1	1	1	1
1	1	76	2	154	64.6	-1.10	27.2	133	75	185	48	137.00	5.70	1	1.00	1	0	1	0	1	1	0	0	0	1	1
1	1	62	1	160	52.3	.50	20.4	149	78	204	59	145.00	5.80	1	1.00	0	0	1	1	0	1	1	1	0	1	1

1	1	21	1	163	77.2	2.20	29.0	123	68	187	41	146.00	5.70	0	1.00	1	1	1	0	1	1	1	0	1	1	1
0	0	45	1	168	64.8	-.30	23.0	106	69	178	64	114.00	5.30	0	1.00	1	0	1	0	1	0	0	1	1	1	1
1	1	79	1	167	51.4	-.50	18.4	116	70	169	66	103.00	5.60	1	1.00	0	0	1	0	1	1	1	1	1	1	1
1	1	76	2	159	61.4	-1.70	24.4	138	77	183	46	137.00	5.40	1	1.00	1	0	1	0	0	1	1	1	0	1	1
0	0	77	1	168	68.8	2.70	24.4	132	81	180	51	129.00	5.40	1	1.00	1	0	1	0	0	1	1	1	0	1	1
1	1	40	2	151	45.8	-1.10	20.2	105	58	156	61	95.00	5.20	1	1.00	0	1	1	1	1	1	1	1	1	1	1
0	0	57	2	155	64.4	1.70	26.7	140	80	184	57	127.00	5.60	1	1.00	1	0	1	1	1	1	1	0	0	1	1
0	0	75	2	154	66.4	3.50	27.9	160	86	193	64	129.00	5.90	1	1.00	1	0	1	0	1	1	1	0	0	0	0
1	1	42	1	173	70.8	.40	23.5	138	86	175	70	105.00	5.60	1	1.00	1	0	1	0	1	0	0	1	1	1	1
1	1	48	2	154	54.9	.50	23.2	127	76	255	82	173.00	5.40	1	1.00	0	0	1	0	1	0	1	1	1	0	1
0	0	58	1	168	63.5	.60	22.4	103	72	218	60	158.00	5.60	1	1.00	0	0	1	1	1	0	1	1	1	1	1
0	0	33	2	154	55.0	-5.40	23.2	92	51	192	71	121.00	5.60	1	1.00	1	0	0	1	1	0	0	1	1	1	1
0	0	60	1	176	60.5	1.60	19.4	114	74	224	103	121.00	6.00	1	1.00	0	0	1	1	1	1	1	1	1	0	1
1	1	58	2	154	46.8	1.20	19.6	162	85	192	64	128.00	5.30	1	1.00	1	1	1	1	1	1	1	1	0	1	1
0	0	30	2	154	41.8	1.10	17.5	100	54	159	78	81.00	4.60	1	1.00	1	1	1	0	0	1	1	1	1	1	1
0	0	36	1	165	64.9	-.10	23.8	141	93	206	57	149.00	5.00	1	1.00	1	0	1	0	1	1	1	1	0	1	1
0	0	37	2	159	50.9	.20	20.0	133	80	223	72	151.00	6.00	1	1.00	1	1	1	0	1	1	1	1	1	0	1
1	1	49	1	174	68.4	.10	22.6	109	72	220	70	150.00	5.40	0	1.00	1	0	1	0	1	0	0	1	1	0	1
0	0	69	2	145	53.4	-2.50	25.4	162	88	290	77	213.00	6.60	1	1.00	1	0	1	0	0	0	0	0	0	0	0
1	1	64	2	153	72.9	2.80	31.2	115	70	184	77	107.00	5.30	1	1.00	1	0	1	0	1	0	1	0	0	1	1
1	1	67	2	156	68.0	-1.80	27.9	142	77	255	37	218.00	5.70	1	1.00	1	0	1	0	1	0	0	0	0	0	1
0	0	71	1	158	47.5	.90	19.0	143	82	172	76	96.00	6.40	1	1.00	0	0	1	0	0	0	1	1	0	1	1
1	1	62	2	156	49.2	-2.40	20.2	128	72	238	68	170.00	5.10	1	1.00	1	0	1	0	0	0	0	1	1	0	1
0	0	59	2	158	55.4	5.70	22.2	113	69	187	87	100.00	5.20	1	1.00	1	0	1	0	0	0	0	1	1	1	1
1	1	39	2	158	55.3	.10	22.0	108	59	155	67	88.00	5.30	0	1.00	1	1	1	0	1	1	0	1	1	1	1
1	1	62	2	154	43.2	.40	18.1	124	78	216	75	141.00	5.20	1	1.00	1	0	1	1	1	0	1	1	1	1	1
1	1	77	2	144	48.9	.80	23.7	129	69	154	68	86.00	6.20	1	1.00	1	0	1	1	0	0	1	1	0	1	1
0	0	67	1	154	60.2	2.00	25.4	103	60	174	60	114.00	5.90	1	1.00	0	0	1	1	0	1	1	0	1	1	1
1	1	32	2	159	47.2	1.60	18.7	95	51	183	92	91.00	5.30	1	1.00	1	1	1	0	1	1	1	1	1	1	1
1	1	55	2	150	48.4	-.20	21.4	97	56	201	67	134.00	5.80	1	1.00	1	0	1	1	1	0	1	1	1	1	1
1	1	72	2	149	56.3	-.70	25.3	152	84	194	80	114.00	5.20	1	1.00	1	0	1	0	1	0	0	0	0	0	1
1	1	75	1	159	65.1	.50	25.7	171	90	197	66	131.00	6.90	1	1.00	1	0	1	0	1	1	1	0	0	1	0
1	1	33	1	164	70.8	-1.80	26.2	121	63	170	59	111.00	5.30	0	1.00	1	1	1	0	1	1	1	0	1	1	1
1	1	85	1	157	58.9	.30	23.8	124	60	224	71	153.00	5.70	1	1.00	0	0	1	1	0	1	1	1	0	0	1
1	1	73	2	151	67.1	-2.90	29.2	156	82	217	51	166.00	6.90	1	1.00	1	0	1	0	1	1	0	0	0	1	0
1	1	43	2	161	63.9	-.60	24.6	131	81	196	78	118.00	5.30	1	1.00	0	0	1	0	1	0	1	1	1	1	1
1	1	46	1	167	77.3	-2.90	27.7	129	80	233	78	155.00	5.40	0	1.00	0	0	1	0	1	1	1	0	1	0	1
1	1	44	1	175	78.9	-3.40	25.8	124	76	181	68	113.00	5.50	1	1.00	0	1	1	0	0	1	1	0	1	1	1
1	1	77	1	166	74.9	.60	27.0	120	67	205	55	150.00	7.00	1	1.00	0	1	1	1	1	1	1	0	0	1	0

1	1	76	1	157	65.2	.00	26.5	140	76	204	30	174.00	6.20	1	1.00	1	1	1	1	1	1	1	0	0	0	1
1	1	68	2	150	59.1	-.20	26.2	135	70	223	75	148.00	5.20	1	1.00	1	1	1	0	1	1	1	0	1	0	1
1	1	88	2	145	42.7	-.10	20.3	121	66	206	76	130.00	5.30	1	1.00	1	0	0	0	1	0	0	1	1	1	1
1	1	38	2	158	57.7	-1.30	22.9	114	68	178	49	129.00	5.40	0	1.00	1	0	1	0	1	1	1	1	1	1	1
0	0	46	2	161	63.3	-1.60	24.3	123	74	167	61	106.00	4.90	1	1.00	0	0	1	0	1	1	1	1	1	1	1
1	1	74	1	163	65.5	-1.00	24.6	127	72	175	60	115.00	5.40	1	1.00	1	0	1	1	1	1	1	1	1	1	1
1	1	73	2	148	53.6	-5.80	24.4	165	86	264	69	195.00	5.60	1	1.00	1	1	1	1	1	1	1	1	0	0	1
0	0	68	2	152	55.4	-.40	24.1	138	70	226	52	174.00	5.70	1	1.00	1	0	1	0	0	0	1	1	1	0	1
0	0	67	2	150	43.6	-.80	19.4	132	73	257	68	189.00	5.40	1	1.00	1	0	1	0	0	1	1	1	1	0	1
1	1	73	1	164	53.6	-.70	19.9	171	96	168	63	105.00	7.40	1	1.00	1	0	1	0	1	0	1	1	0	1	0
1	1	70	1	160	84.4	-4.20	33.0	192	106	204	50	154.00	6.40	1	1.00	0	0	1	0	0	0	1	0	0	1	1
0	0	68	1	167	61.6	-5.30	22.1	103	69	182	65	117.00	5.80	1	1.00	0	1	1	0	1	0	1	1	0	1	1
1	1	67	2	154	50.4	1.40	21.2	128	75	214	67	147.00	5.60	0	1.00	1	0	1	0	1	0	0	1	0	1	1
1	1	67	1	166	66.5	1.10	24.1	140	93	161	46	115.00	5.70	1	1.00	1	0	1	0	1	1	1	1	0	1	1
1	1	73	2	145	52.0	-3.80	24.8	177	104	201	62	139.00	5.30	1	1.00	1	0	1	0	1	0	1	1	0	1	1
1	1	74	1	161	60.1	-1.40	23.2	124	68	194	57	137.00	5.50	0	1.00	0	1	1	1	1	1	1	1	1	1	1
1	1	43	2	154	57.0	.80	23.9	178	94	193	46	147.00	4.50	1	1.00	1	0	1	1	1	1	1	1	0	1	1
1	1	50	1	169	60.5	1.00	21.2	104	63	142	49	93.00	5.30	1	1.00	1	0	1	1	1	0	1	1	1	1	1
1	1	63	2	162	56.4	-3.10	21.4	115	60	290	78	212.00	5.90	1	1.00	1	0	1	0	1	0	1	1	1	0	1
0	0	60	2	154	48.6	-1.10	20.5	110	64	238	75	163.00	5.30	1	1.00	1	0	1	1	1	0	1	1	1	0	1
0	0	80	2	145	45.5	-.80	21.5	113	65	241	86	155.00	5.80	1	1.00	1	0	1	1	1	0	1	1	0	0	1
1	1	33	1	166	79.1	-3.20	28.8	119	74	186	43	143.00	5.50	0	1.00	0	1	1	1	1	1	1	0	1	1	1
0	0	63	1	182	80.9	-1.60	24.4	154	81	227	63	164.00	5.80	0	1.00	0	0	1	0	1	1	1	1	0	0	1
0	0	74	1	163	66.0	-1.20	24.9	107	70	201	61	140.00	6.10	1	1.00	0	0	1	0	1	0	1	1	1	1	1
0	0	77	1	159	48.9	-1.80	19.3	139	76	178	81	97.00	5.30	1	1.00	0	0	1	0	1	1	0	1	0	1	1
0	0	53	2	159	56.5	1.90	22.2	140	89	212	76	136.00	5.40	1	1.00	1	1	1	0	1	0	0	1	0	1	1
0	1	42	2	163	54.2	2.90	20.5	116	66	171	92	79.00	5.10	1	1.00	1	0	1	0	0	1	1	1	1	1	1
1	1	71	2	156	72.0	-4.40	29.5	164	78	219	112	107.00	7.00	1	1.00	1	0	1	0	1	0	1	0	0	1	0
1	1	58	2	157	50.6	-1.90	20.5	132	74	214	43	171.00	6.60	1	1.00	1	0	1	1	0	1	0	1	1	1	0
1	1	68	2	155	68.3	1.70	28.3	139	81	189	42	147.00	5.90	1	1.00	1	1	1	0	1	1	1	0	1	1	1
1	1	37	1	167	88.1	.80	31.7	113	61	201	33	168.00	5.80	1	1.00	1	0	1	0	1	0	0	0	1	0	1
0	0	62	2	161	60.3	4.40	23.2	121	68	189	58	131.00	6.00	1	1.00	1	0	1	0	0	1	1	1	1	1	1
0	1	61	2	156	59.4	-.60	24.2	138	79	196	84	112.00	6.10	1	1.00	1	0	1	1	1	1	1	1	1	1	1
0	0	56	2	154	51.1	.10	21.6	135	79	243	102	141.00	5.30	1	1.00	1	0	1	0	0	1	1	1	1	0	1
1	1	75	2	153	47.7	1.10	20.3	119	83	217	67	150.00	5.40	1	1.00	1	0	1	1	1	1	1	1	1	1	1
0	0	64	2	151	54.1	2.60	23.7	171	92	259	119	140.00	6.20	1	1.00	1	0	1	0	1	1	0	1	0	0	1
1	1	64	1	166	60.9	1.50	22.0	119	72	202	54	148.00	5.80	1	1.00	0	0	1	1	1	1	1	1	0	0	1
0	0	57	2	154	50.0	2.80	21.1	122	66	209	82	127.00	5.50	1	1.00	1	1	1	0	0	0	0	1	1	1	1
0	0	58	1	167	68.8	2.00	24.6	126	78	236	46	190.00	5.90	0	1.00	1	0	1	0	1	1	0	1	1	0	1

1	1	62	2	155	62.4	.60	26.1	157	88	241	55	186.00	5.70	1	1.00	0	0	1	1	1	1	1	0	0	0	1
1	1	72	1	163	59.1	-1.30	22.3	138	78	189	64	125.00	5.40	0	1.00	0	0	1	1	1	1	1	1	1	1	1
0	0	69	2	155	49.8	2.50	20.8	157	87	189	63	126.00	5.80	1	1.00	1	0	1	1	1	1	0	1	0	1	1
1	1	64	2	150	54.8	-.30	24.3	170	103	243	48	195.00	5.90	0	1.00	1	0	1	1	1	0	1	1	0	0	1
1	1	82	1	164	54.1	-.90	20.0	125	74	176	66	110.00	5.30	1	1.00	1	0	1	1	1	1	1	1	1	1	1
1	1	43	2	156	56.7	1.70	23.2	115	65	154	64	90.00	5.60	1	1.00	1	0	1	0	1	0	0	1	1	1	1
1	1	56	2	153	67.9	2.10	29.0	130	72	196	47	149.00	5.70	1	1.00	1	0	1	0	1	1	1	0	0	0	1
0	0	73	2	147	43.0	-2.60	19.9	146	79	169	55	114.00	5.60	1	1.00	1	0	1	1	1	1	1	1	0	1	1
0	0	78	2	147	55.1	-1.20	25.4	97	55	161	61	100.00	5.20	1	1.00	1	1	1	0	1	0	0	0	1	1	1
1	1	67	1	166	62.9	.00	22.8	185	110	224	77	147.00	5.50	1	1.00	0	0	1	1	1	1	0	1	0	0	1
1	1	42	2	152	50.3	1.70	21.7	120	74	185	98	87.00	5.40	1	1.00	1	0	1	0	0	0	0	1	1	1	1
0	0	63	2	154	46.5	-1.50	19.5	108	61	244	67	177.00	5.80	1	1.00	1	0	0	0	1	0	0	1	1	0	1
1	1	69	2	149	56.8	-1.30	25.6	120	65	216	72	144.00	6.10	1	1.00	1	0	1	1	1	1	1	0	1	1	1
0	0	66	1	169	67.0	-.90	23.4	118	73	173	62	111.00	6.20	1	1.00	0	1	1	0	1	1	1	1	1	1	1
0	0	69	1	160	50.9	1.30	19.9	113	69	186	58	128.00	5.50	1	1.00	1	0	1	0	1	1	1	1	1	1	1
1	1	70	1	171	79.4	-1.30	27.2	129	77	220	72	148.00	5.20	1	1.00	0	1	1	1	1	1	1	0	0	0	1
1	1	60	2	151	61.2	-1.00	26.8	138	83	221	55	166.00	5.60	1	1.00	1	0	1	1	1	0	0	0	1	0	1
1	1	74	1	162	58.4	1.20	22.1	134	68	211	67	144.00	5.50	1	1.00	1	0	1	0	1	1	1	1	1	1	1
1	1	45	2	160	74.7	5.20	29.2	114	64	228	48	180.00	5.60	0	1.00	1	0	1	1	1	1	1	0	1	0	1
1	1	40	2	164	63.2	-10.10	23.4	123	66	220	50	170.00	5.40	1	1.00	1	0	1	0	1	0	1	1	1	0	1
1	1	56	1	167	67.9	2.80	24.4	136	86	219	88	131.00	5.40	0	1.00	0	0	1	0	0	0	1	1	1	0	1
1	1	63	1	162	60.4	1.30	23.1	146	85	219	60	159.00	5.60	0	1.00	0	0	1	1	1	1	1	1	0	1	1
1	1	34	1	176	89.1	5.50	28.6	157	86	174	28	146.00	5.70	1	1.00	1	0	0	0	1	0	1	0	0	0	1
0	0	33	2	150	48.6	.70	21.7	119	62	233	87	146.00	5.00	1	1.00	1	0	1	1	1	1	1	1	1	0	1
0	0	49	1	172	86.0	-1.10	29.0	115	79	230	41	189.00	5.50	0	1.00	1	0	1	0	1	1	1	0	1	0	1
1	1	55	2	165	55.7	-1.20	20.4	111	56	292	41	251.00	5.80	1	1.00	1	0	1	1	1	1	1	1	1	0	1
0	0	40	1	168	61.9	-1.40	21.8	130	77	208	61	147.00	5.50	0	1.00	0	0	0	0	1	1	1	1	1	1	1
1	1	45	1	165	66.5	-3.80	24.3	118	72	209	55	154.00	5.10	0	1.00	0	0	0	0	0	1	1	1	1	1	1
0	0	37	2	150	49.0	-.50	21.7	109	63	171	80	91.00	5.30	1	1.00	1	1	1	0	1	1	1	1	1	1	1
1	1	73	2	144	50.9	.10	24.5	103	56	259	63	196.00	6.20	1	1.00	1	0	1	1	1	1	1	1	1	0	1
1	1	56	2	152	61.4	-1.30	26.6	112	74	256	78	178.00	5.80	1	1.00	1	1	1	0	1	1	1	0	1	0	1
1	1	53	2	153	43.6	-.90	18.6	128	76	238	83	155.00	5.40	1	1.00	1	0	1	1	1	1	0	1	1	0	1
1	1	57	2	158	65.5	3.10	26.1	137	82	225	58	167.00	5.60	1	1.00	1	1	1	1	0	0	1	0	1	0	1
1	1	68	1	155	53.3	1.40	22.2	99	58	244	86	158.00	6.10	1	1.00	1	0	1	0	1	0	1	1	1	0	1
1	1	47	2	154	41.9	-.50	17.6	128	67	236	101	135.00	5.50	1	1.00	1	0	1	1	1	0	1	1	1	0	1
1	1	65	2	154	57.0	-8.00	24.1	139	75	295	82	213.00	5.30	1	1.00	1	0	1	1	1	1	1	1	1	0	1
1	1	57	2	153	62.6	.30	26.6	132	76	267	41	226.00	6.40	1	1.00	1	0	1	0	1	0	0	0	0	0	1
1	1	75	2	153	60.6	.80	25.8	127	86	213	52	161.00	5.80	1	1.00	1	1	1	0	0	1	0	0	0	0	1
1	1	74	2	146	49.4	1.40	23.2	144	77	226	66	160.00	5.50	1	1.00	1	0	1	1	1	0	1	1	0	0	1

1	1	82	2	145	39.6	.40	18.9	140	75	230	81	149.00	5.90	1	1.00	1	0	1	0	1	1	0	1	0	0	1
1	1	77	1	168	76.9	-.30	27.2	123	75	198	50	148.00	5.70	1	1.00	0	0	1	0	1	1	1	0	1	1	1
1	1	70	2	144	46.9	-.10	22.6	154	82	238	71	167.00	5.70	1	1.00	1	0	1	0	1	0	1	1	0	0	1
0	0	65	1	171	63.5	3.30	21.6	116	73	107	54	53.00	5.10	1	1.00	0	0	1	0	1	1	1	1	0	1	1
1	1	73	2	149	44.3	2.40	19.9	123	77	150	69	81.00	5.40	1	1.00	1	0	1	0	1	0	1	1	1	1	1
1	1	60	2	160	71.9	1.40	28.1	167	97	217	47	170.00	5.60	1	1.00	1	0	1	1	1	0	0	0	0	1	1
1	1	59	1	169	77.0	1.10	27.0	144	85	205	45	160.00	5.90	0	1.00	0	0	1	0	0	1	1	0	0	1	1
0	0	82	1	161	59.2	-.10	22.8	134	75	153	60	93.00	6.00	1	1.00	0	1	1	0	1	1	1	1	1	1	1
1	1	59	2	147	50.9	4.00	23.4	125	69	259	66	193.00	5.50	1	1.00	1	1	1	1	1	1	1	1	1	0	1
1	1	79	2	141	41.7	-2.20	20.8	139	75	225	69	156.00	5.50	1	1.00	1	1	1	1	1	1	1	1	1	0	1
0	0	63	2	152	49.2	2.80	21.3	129	84	217	57	160.00	5.40	1	1.00	1	0	1	1	0	1	1	1	1	1	1
1	1	68	2	151	49.6	3.80	21.8	107	61	204	35	169.00	5.30	1	1.00	1	0	1	0	1	0	1	1	0	0	1
1	1	66	2	155	55.2	1.40	22.9	131	76	256	66	190.00	5.70	1	1.00	0	0	1	0	1	1	1	1	0	0	1
1	1	63	1	171	62.4	-.20	21.4	152	91	233	61	172.00	5.70	1	1.00	0	1	1	0	0	1	1	1	0	0	1
0	0	68	2	158	58.3	-2.30	23.3	112	67	171	57	114.00	5.90	1	1.00	1	0	1	0	1	1	1	1	1	1	1
0	0	69	1	157	44.7	1.30	18.1	132	71	173	51	122.00	5.80	0	2.00	1	0	1	1	1	1	0	1	0	1	1
0	0	60	1	153	65.8	-1.00	27.9	118	77	215	53	162.00	5.90	1	1.00	1	1	1	0	1	0	0	0	1	1	1
0	0	75	2	149	47.3	.60	21.3	138	81	210	84	126.00	5.40	1	1.00	1	0	1	0	1	1	1	1	1	1	1
1	1	62	2	153	38.3	.40	16.2	96	57	191	89	102.00	5.80	1	1.00	1	0	1	1	1	0	1	1	1	1	1
1	1	80	2	155	99.3	8.90	41.1	147	60	231	64	167.00	5.90	1	1.00	1	0	1	1	1	1	1	0	0	0	1
1	1	54	2	161	53.4	-1.80	20.5	123	78	230	75	155.00	5.30	0	1.00	0	1	0	0	1	0	0	1	1	0	1
0	1	45	2	153	53.4	-.70	22.7	120	74	230	49	181.00	5.40	1	1.00	0	0	1	0	1	0	1	1	1	0	1
0	0	78	2	158	71.7	2.30	28.5	126	75	192	42	150.00	5.90	1	1.00	0	0	1	0	1	1	1	0	0	1	1
1	1	64	2	150	55.3	1.10	24.6	138	93	276	83	193.00	5.40	1	1.00	1	0	1	0	1	0	0	1	0	0	1
1	1	66	2	144	50.4	1.10	24.4	141	82	269	72	197.00	5.40	1	1.00	1	0	1	1	1	1	1	1	0	0	1
1	1	58	2	147	43.3	.30	20.0	135	83	224	68	156.00	5.30	1	1.00	1	0	1	1	1	0	1	1	1	0	1
1	1	57	2	149	57.2	3.80	25.9	124	76	270	51	219.00	5.80	1	1.00	1	1	1	0	1	1	0	0	1	0	1
1	1	62	2	151	47.8	.90	21.0	93	66	294	61	233.00	6.20	1	1.00	1	0	1	1	1	1	1	1	0	0	1
1	1	37	2	163	58.9	.30	22.1	101	58	179	35	144.00	5.10	1	1.00	1	1	1	1	1	0	1	1	1	0	1
1	1	33	2	165	51.4	-1.10	18.9	134	96	161	77	84.00	4.90	1	1.00	0	1	1	0	0	1	0	1	0	1	1
1	1	62	1	163	51.9	-2.10	19.6	107	67	207	45	162.00	5.90	1	1.00	0	0	1	0	0	1	0	1	1	1	1
0	0	67	2	158	56.8	3.30	22.8	144	85	192	55	137.00	6.00	1	1.00	1	0	1	1	1	0	1	1	0	0	1
0	0	40	2	159	54.4	-3.10	21.5	91	53	181	87	94.00	5.30	0	1.00	1	0	1	0	1	1	1	1	1	1	1
0	0	74	1	154	49.4	.90	20.8	129	78	217	67	150.00	5.30	1	1.00	1	0	1	0	1	0	0	1	1	1	1
1	1	70	1	162	52.3	.30	19.9	114	58	211	55	156.00	5.70	1	1.00	1	0	1	1	1	1	1	1	0	1	1
1	1	69	2	148	51.2	.50	23.5	149	75	193	51	142.00	5.60	1	1.00	1	0	1	1	1	1	1	1	0	1	1
1	1	75	1	159	50.3	-1.50	20.0	176	88	168	105	63.00	6.10	1	1.00	0	0	1	1	1	0	1	1	0	1	1
1	1	58	2	156	49.2	2.20	20.2	118	78	167	66	101.00	5.60	1	1.00	1	0	1	1	1	1	1	1	1	1	1
0	0	69	2	153	63.7	3.30	27.2	148	85	229	87	142.00	5.60	1	1.00	1	0	1	0	1	0	0	0	0	0	1

1	1	65	2	149	50.3	-.40	22.5	129	80	224	76	148.00	5.80	1	1.00	1	0	1	1	1	0	1	1	1	0	1
1	1	58	2	152	46.4	-.40	20.0	124	68	227	73	154.00	5.80	1	1.00	1	1	1	1	1	1	1	1	1	0	1
1	1	74	2	148	66.1	.50	30.3	128	77	228	73	155.00	6.10	1	1.00	1	0	1	0	1	1	1	0	0	0	1
1	1	76	1	161	69.5	-1.60	26.9	129	74	198	55	143.00	5.70	1	1.00	1	1	1	1	1	1	1	0	0	1	1
1	1	78	1	153	58.9	-1.00	25.0	122	73	216	59	157.00	4.50	1	1.00	0	0	1	1	1	1	1	0	0	1	1
0	0	77	2	149	79.6	1.30	35.7	139	81	194	53	141.00	5.90	1	1.00	1	0	1	1	1	0	1	0	0	1	0
1	1	76	1	144	42.0	-1.60	20.1	135	69	172	77	95.00	4.60	0	1.00	1	0	1	1	1	0	1	1	1	1	1
1	1	69	1	164	66.1	-1.40	24.4	144	81	234	59	175.00	5.40	1	1.00	0	0	1	0	1	1	1	1	0	0	1
1	1	84	1	152	50.0	-.50	21.7	145	81	154	60	94.00	5.20	1	1.00	0	0	1	1	1	1	1	1	0	1	1
1	1	48	2	162	55.5	-2.10	21.1	125	71	180	49	131.00	5.50	1	1.00	1	0	1	0	0	1	1	1	1	1	1
1	1	75	2	148	51.7	-2.00	23.4	149	78	249	57	192.00	5.70	1	1.00	1	1	1	1	1	0	0	1	0	0	1
1	1	58	2	152	50.9	2.40	22.0	108	71	183	63	120.00	6.30	1	1.00	1	0	1	1	1	0	1	1	1	0	1
1	1	67	2	154	53.6	.40	22.4	101	61	221	70	151.00	5.70	1	1.00	0	0	1	1	1	1	1	1	1	0	1
1	1	55	2	163	53.0	-1.90	19.9	98	67	294	78	216.00	6.20	1	1.00	0	0	1	0	0	0	1	1	1	0	1
1	1	84	2	147	42.3	-.70	19.6	142	84	265	71	194.00	5.50	1	1.00	1	1	1	1	1	1	1	1	0	0	1
1	1	71	1	166	72.7	3.80	26.4	138	79	167	74	93.00	6.30	0	2.00	0	0	1	0	1	1	1	0	0	1	1
1	1	79	1	160	50.8	.60	19.9	187	104	183	59	124.00	5.60	1	1.00	0	0	1	1	1	1	1	1	0	1	1
1	1	77	1	162	57.2	-.30	21.7	138	78	204	75	129.00	5.60	1	1.00	0	1	1	0	1	1	1	1	0	1	1
0	0	66	1	162	66.9	-1.00	25.4	161	95	223	54	169.00	6.20	1	1.00	0	0	1	0	1	0	1	0	0	0	1
0	0	68	2	155	56.1	.50	23.4	134	84	231	52	179.00	5.50	1	1.00	1	0	1	0	1	0	0	1	1	0	1
1	1	64	2	154	48.0	.90	20.3	137	81	191	69	122.00	5.50	1	1.00	1	1	1	1	1	1	1	1	1	1	1
1	1	74	1	163	75.1	-1.40	28.3	150	85	196	67	129.00	5.20	1	1.00	0	0	1	0	1	1	1	0	0	1	1
0	1	72	1	160	69.4	2.20	26.9	151	97	284	120	164.00	5.60	1	1.00	0	0	1	0	1	1	1	0	0	0	1
1	1	65	2	159	66.2	2.30	26.0	138	90	229	79	150.00	5.20	1	1.00	1	0	1	1	0	1	1	0	0	0	1
1	1	78	2	155	62.0	-2.70	25.7	126	66	179	54	125.00	6.10	1	1.00	1	0	1	1	1	0	0	0	0	1	1
1	1	72	2	149	48.0	.80	21.6	98	61	216	69	147.00	5.60	1	1.00	1	1	1	1	1	1	1	1	1	1	1
1	1	85	1	159	38.7	2.40	15.4	101	44	183	86	97.00	5.30	1	1.00	0	0	1	0	1	1	1	1	0	1	1
1	1	72	2	141	55.9	-1.70	28.2	139	80	228	60	168.00	5.80	1	1.00	1	0	1	1	1	1	1	0	0	0	0
1	1	21	1	166	44.8	.40	16.2	126	76	167	62	105.00	5.10	1	1.00	1	0	0	0	1	1	0	1	1	1	1
0	0	60	2	158	55.2	2.50	22.2	126	78	161	50	111.00	6.70	1	1.00	1	1	1	0	1	0	0	1	0	0	0
0	0	68	2	146	50.4	.60	23.6	151	85	252	56	196.00	5.40	1	1.00	1	1	1	1	1	0	0	1	0	0	1
0	0	73	1	164	72.8	-2.80	26.9	187	107	214	57	157.00	5.60	1	1.00	0	1	1	1	1	1	1	0	0	1	1
1	1	73	2	150	50.1	.60	22.2	109	68	258	74	184.00	5.60	1	1.00	1	1	1	1	1	0	0	1	0	0	1
0	0	78	1	155	52.0	1.80	21.7	114	67	175	44	131.00	6.20	1	1.00	1	1	1	0	1	1	1	1	0	1	0
1	1	73	2	146	43.2	.80	20.3	106	65	159	64	95.00	6.20	1	1.00	1	1	1	0	1	0	1	1	1	0	1
1	1	73	2	150	63.5	3.50	28.4	137	86	207	53	154.00	5.60	1	1.00	1	0	1	1	1	1	1	0	1	1	1
1	1	63	2	157	59.8	2.00	24.2	137	78	183	45	138.00	6.00	1	1.00	1	0	1	1	1	1	0	1	1	0	1
1	1	74	2	149	54.0	1.20	24.3	135	72	242	70	172.00	5.00	1	1.00	1	0	1	0	1	0	1	1	0	0	1
0	0	50	1	166	74.8	2.60	27.0	112	75	258	41	217.00	5.70	1	1.00	0	0	1	0	0	1	1	0	1	0	1

0	0	78	1	161	63.0	.80	24.4	135	72	174	62	112.00	6.60	1	1.00	0	0	1	1	1	1	1	1	0	1	0
1	1	36	1	174	64.6	2.40	21.4	123	83	183	59	124.00	5.40	0	1.00	0	0	1	0	1	0	0	1	1	1	1
1	1	62	2	152	49.3	2.40	21.4	173	88	239	47	192.00	5.70	1	1.00	1	0	1	1	0	0	1	1	0	0	1
0	0	75	2	153	66.2	.00	28.3	158	79	233	70	163.00	6.20	1	1.00	1	0	1	1	1	0	0	0	0	0	1
0	0	77	2	138	56.1	-.60	29.4	126	72	197	69	128.00	5.70	1	1.00	1	1	1	0	1	1	1	0	0	1	1
1	1	55	2	152	57.3	-.70	24.8	138	89	223	60	163.00	5.50	1	1.00	1	0	1	1	0	0	0	1	1	0	1
0	1	78	2	142	53.2	-2.10	26.5	116	73	238	76	162.00	6.80	1	1.00	1	0	1	0	1	1	1	0	1	0	0
0	0	52	2	153	48.4	2.80	20.7	118	70	209	115	94.00	5.30	1	1.00	1	0	1	1	1	0	0	1	1	1	1
1	1	71	2	145	39.9	-.80	19.0	136	73	229	94	135.00	5.50	1	1.00	1	1	1	0	1	1	1	1	1	0	1
1	1	81	1	150	45.9	3.50	20.3	134	76	216	67	149.00	5.70	1	1.00	1	0	1	0	1	0	1	1	1	1	1
0	0	73	1	164	70.1	1.00	26.0	116	82	163	45	118.00	7.20	1	1.00	1	0	1	1	0	0	0	0	1	1	0
1	1	63	2	153	39.3	-.40	16.8	139	78	232	82	150.00	5.60	1	1.00	1	0	1	1	1	1	0	1	1	0	1
0	0	74	2	152	53.1	2.90	23.1	93	51	221	80	141.00	6.00	1	1.00	1	0	1	1	0	1	1	1	0	0	1
1	1	60	1	157	63.5	-3.20	25.6	139	88	233	72	161.00	7.10	1	1.00	1	0	1	0	0	0	1	0	1	0	0
1	1	73	2	154	57.6	-.60	24.3	113	69	215	68	147.00	5.90	1	1.00	1	0	1	1	1	1	1	1	0	0	1
1	1	80	1	164	64.3	1.60	23.8	132	80	129	41	88.00	6.30	1	1.00	1	0	1	0	1	1	1	1	0	1	1
0	0	61	2	154	69.6	1.10	29.5	123	79	152	37	115.00	6.00	1	1.00	1	1	1	0	0	0	1	0	0	0	1
1	1	82	1	161	73.0	1.30	28.3	114	56	219	61	158.00	5.80	1	1.00	0	0	1	1	1	1	0	0	0	0	1
1	1	50	2	159	47.7	3.50	18.8	110	75	226	72	154.00	5.20	1	1.00	1	0	1	0	0	1	1	1	1	0	1
1	1	46	1	171	68.2	-4.70	23.2	102	74	239	53	186.00	5.30	1	1.00	0	0	1	0	0	0	1	1	1	0	1
1	1	19	2	167	58.2	-.90	20.9	100	55	159	60	99.00	5.50	1	1.00	1	0	0	1	1	0	1	1	1	1	1
1	1	67	2	150	38.1	1.00	16.8	117	60	141	52	89.00	5.60	1	1.00	1	0	1	1	1	1	1	1	1	1	1
0	0	69	1	159	61.3	-8.10	24.3	120	65	257	56	201.00	8.30	0	2.00	1	0	1	0	1	1	1	1	0	0	0
0	0	58	2	150	63.9	-1.20	28.3	127	71	245	85	160.00	5.90	1	1.00	1	0	1	1	0	1	0	0	1	0	1
0	0	42	2	162	58.1	2.40	22.1	114	58	171	84	87.00	5.60	0	1.00	0	0	0	1	0	1	1	1	1	1	1
0	0	81	2	144	52.5	1.20	25.4	149	80	198	53	145.00	5.60	1	1.00	1	0	1	0	1	1	1	0	0	1	1
1	1	75	2	138	43.5	-1.70	22.9	120	70	180	45	135.00	5.40	1	1.00	1	0	1	1	1	1	1	1	1	1	1
1	1	73	1	163	65.4	-1.50	24.5	139	81	197	47	150.00	5.30	1	1.00	1	0	1	1	1	1	1	1	0	1	1
1	1	77	1	157	66.3	1.00	26.7	114	66	183	43	140.00	5.70	1	1.00	0	1	1	0	1	0	1	0	1	1	0
0	0	70	1	164	56.9	2.90	21.2	133	74	207	85	122.00	5.80	1	1.00	1	1	1	0	1	1	1	1	1	1	1
1	1	80	2	149	43.0	1.60	19.2	121	64	192	87	105.00	6.40	1	1.00	1	1	1	1	1	0	0	1	0	0	0
0	0	35	2	157	51.3	1.70	20.7	103	66	160	66	94.00	4.80	1	1.00	1	0	0	0	0	0	0	1	1	1	1
1	1	70	2	155	60.7	-1.00	25.1	150	78	197	78	119.00	5.90	1	1.00	1	0	1	1	1	0	0	0	0	0	1
1	1	56	2	154	60.7	.10	25.5	110	77	256	64	192.00	5.20	1	1.00	1	0	1	1	1	0	1	0	1	0	1
1	1	34	1	162	83.7	-1.60	31.8	122	70	241	34	207.00	5.60	1	1.00	1	1	1	1	1	1	1	0	1	0	1
1	1	61	2	161	51.3	-1.40	19.9	111	63	231	84	147.00	5.70	1	1.00	1	0	1	0	0	0	0	1	1	0	1
1	1	60	2	143	45.8	1.60	22.5	96	61	249	45	204.00	5.80	1	1.00	1	0	1	0	0	0	0	1	1	0	1
1	1	55	2	153	48.1	-.40	20.6	112	64	230	108	122.00	5.90	1	1.00	1	0	1	0	1	1	1	1	1	0	1
0	0	77	1	149	53.6	-3.10	24.1	129	87	226	78	148.00	6.30	1	1.00	1	0	1	0	1	1	1	1	0	0	1

1	1	77	1	160	58.0	-1.50	22.7	110	71	158	41	117.00	5.80	1	1.00	1	1	1	1	1	1	0	1	1	1	1
0	0	85	2	135	34.4	-1.20	18.8	142	72	259	75	184.00	7.60	1	1.00	1	0	1	1	1	1	1	1	0	0	0
1	1	73	1	165	63.1	-2.20	23.2	133	87	198	52	146.00	5.70	1	1.00	1	0	1	0	1	1	0	1	1	1	1
1	1	90	2	143	42.3	1.30	20.7	163	80	216	67	149.00	5.80	1	1.00	1	0	1	1	1	0	1	1	0	1	1
1	1	76	2	152	64.5	-1.50	27.8	139	85	176	37	139.00	5.60	1	1.00	1	1	1	0	1	1	1	0	1	0	1
1	1	75	2	156	52.5	-2.50	21.4	134	75	183	69	114.00	5.60	1	1.00	1	1	1	1	1	1	1	1	1	1	1
1	1	49	1	163	48.1	.10	18.1	102	67	217	77	140.00	5.60	1	1.00	1	0	1	1	1	0	1	1	1	1	1
0	0	77	2	145	47.9	.00	22.7	144	83	247	81	166.00	5.20	1	1.00	1	0	1	1	1	0	0	1	0	0	1
0	0	77	1	166	68.5	-2.00	24.8	140	82	188	69	119.00	6.60	1	1.00	0	0	1	1	1	1	1	1	0	1	0
1	1	60	1	160	51.7	-5.10	20.2	119	73	186	66	120.00	5.70	1	1.00	0	0	1	0	1	0	1	1	0	1	1
1	1	63	2	140	65.2	-2.50	33.3	120	81	183	56	127.00	6.70	1	1.00	1	1	1	0	1	1	1	0	1	1	0
1	1	66	1	164	64.9	-.60	24.1	154	78	184	46	138.00	6.30	0	1.00	1	1	1	1	1	1	1	1	0	1	0
0	0	42	2	153	62.1	-1.40	26.5	163	85	202	66	136.00	5.90	1	1.00	0	1	1	1	1	0	1	0	0	1	1
1	1	82	2	138	41.0	.40	21.6	156	83	195	69	126.00	6.80	1	1.00	1	0	1	1	1	1	1	1	0	1	0
1	1	86	2	149	54.5	.00	24.5	150	79	217	62	155.00	5.70	1	1.00	1	1	1	1	1	1	1	1	0	1	1
1	1	83	2	129	35.9	.00	21.7	140	81	198	67	131.00	5.50	1	1.00	1	0	1	0	1	0	1	1	0	1	1
1	1	83	1	163	54.7	-1.90	20.6	123	75	191	82	109.00	5.80	1	1.00	1	0	1	1	1	1	0	1	1	1	1
1	1	80	2	148	43.5	-1.20	19.8	128	74	170	77	93.00	6.00	1	1.00	1	1	1	1	1	1	1	1	0	1	1
1	1	64	2	148	67.4	-1.20	30.8	167	93	168	81	87.00	6.50	1	1.00	1	0	1	1	1	0	1	0	0	1	0
1	1	90	1	157	52.3	-.90	21.2	119	57	148	43	105.00	5.20	1	1.00	1	1	1	1	1	0	0	1	0	1	1
1	1	73	2	144	43.5	.60	20.8	128	73	207	76	131.00	5.50	1	1.00	1	0	1	0	1	0	1	1	1	1	1
1	1	75	1	153	73.7	-2.40	31.4	148	86	250	66	184.00	6.20	1	1.00	0	1	1	1	1	1	1	0	0	0	1
1	1	52	1	175	67.0	-.70	21.9	130	85	249	62	187.00	5.90	0	1.00	0	0	1	1	1	1	1	1	1	0	1
1	1	71	1	153	56.5	-2.60	24.2	99	65	175	62	113.00	5.60	0	1.00	1	1	1	1	1	0	1	1	0	1	1
0	0	77	2	146	44.5	.50	21.0	128	71	227	59	168.00	5.60	1	1.00	1	0	1	0	1	0	0	1	1	0	1
0	0	36	2	158	54.3	-.70	21.8	112	73	184	82	102.00	5.40	1	1.00	0	0	1	0	1	0	0	1	1	1	1
0	0	62	2	155	55.6	-.50	23.1	140	76	251	52	199.00	5.80	1	1.00	1	1	1	1	1	0	1	1	0	0	1
1	1	57	2	149	49.7	2.90	22.4	98	62	301	88	213.00	5.40	1	1.00	1	1	1	1	1	0	0	1	1	0	1
1	1	34	2	161	52.3	-.80	20.1	133	85	182	59	123.00	5.50	1	1.00	1	0	1	0	1	1	0	1	1	1	1
0	0	50	2	147	52.4	.90	24.2	118	70	295	63	232.00	6.20	1	1.00	0	0	1	0	0	1	1	1	1	0	1
1	1	70	1	159	70.9	-3.90	28.0	155	81	151	31	120.00	5.10	1	1.00	0	1	1	0	0	1	1	0	0	0	1
1	1	34	2	158	83.9	-.70	33.6	128	71	259	77	182.00	5.30	1	1.00	1	1	1	1	1	0	1	0	1	0	1
1	1	71	2	148	53.2	-.90	24.2	129	90	230	77	153.00	6.20	1	1.00	1	1	1	1	1	0	0	1	0	0	1
0	0	68	2	142	44.5	1.20	22.1	133	75	286	65	221.00	5.80	1	1.00	1	0	0	0	0	1	1	1	1	0	1
1	1	75	1	158	65.2	.20	26.2	153	82	173	73	100.00	5.40	0	1.00	0	0	1	1	1	0	0	0	0	1	1
1	1	79	2	146	52.5	-3.30	24.7	147	80	232	70	162.00	6.30	1	1.00	1	0	1	0	1	0	0	1	0	0	1
1	1	28	1	168	51.3	.40	18.2	122	65	203	84	119.00	5.60	1	1.00	0	1	1	1	0	0	1	1	1	1	1
0	0	75	1	160	56.9	.60	22.1	117	60	218	43	175.00	5.40	1	1.00	1	0	1	0	0	1	0	1	0	1	1
1	1	62	1	172	68.5	3.60	23.2	120	85	195	69	126.00	5.20	1	1.00	0	0	1	0	0	1	0	1	1	1	1

1	1	39	2	164	51.5	-.60	19.1	101	61	173	87	86.00	5.50	1	1.00	1	0	1	1	1	0	1	1	1	1	1
1	1	39	2	161	54.5	3.10	20.9	96	56	190	65	125.00	5.60	1	1.00	0	0	1	0	0	1	1	1	1	1	1
0	0	73	2	146	65.6	1.20	30.9	132	79	203	46	157.00	6.60	1	1.00	1	0	1	1	0	0	0	0	1	1	0
0	0	70	2	149	49.8	3.10	22.4	142	78	201	63	138.00	5.60	1	1.00	1	0	1	1	1	0	1	1	0	1	1
0	0	50	1	170	79.4	-1.90	27.4	125	80	221	47	174.00	5.80	1	1.00	0	0	1	0	0	1	0	0	0	0	1
1	1	39	1	167	84.7	-2.40	30.4	130	75	271	46	225.00	5.80	1	1.00	0	0	0	0	1	0	1	0	1	0	1
1	1	56	1	162	60.9	-1.80	23.1	129	81	174	53	121.00	7.40	1	1.00	0	1	1	1	1	0	1	1	1	1	0
1	1	56	1	165	66.0	-4.20	24.2	134	89	231	62	169.00	5.70	1	1.00	1	0	1	1	1	0	0	1	0	0	1
0	0	73	1	160	53.8	.40	21.0	114	69	199	62	137.00	5.10	1	1.00	0	0	1	0	1	1	1	1	0	1	1
0	0	36	1	186	84.6	-3.60	24.4	130	78	227	48	179.00	5.60	1	1.00	0	1	1	0	1	1	1	1	1	0	1
0	0	69	2	161	57.9	7.20	22.3	117	62	162	59	103.00	5.80	1	1.00	1	0	1	0	1	0	0	1	1	0	1
1	1	76	1	156	73.8	-3.80	30.4	144	91	174	41	133.00	5.30	1	1.00	1	0	1	1	1	0	1	0	0	1	1
1	1	44	2	149	47.2	.70	21.2	101	55	204	82	122.00	5.50	1	1.00	0	0	1	0	1	0	1	1	1	1	1
0	0	67	2	148	59.4	1.50	27.0	111	77	244	66	178.00	5.50	1	1.00	1	0	1	0	1	0	0	0	0	0	1
0	0	79	2	150	53.3	-1.50	23.6	142	81	225	64	161.00	5.70	1	1.00	1	0	1	1	1	1	0	1	0	0	1
1	1	75	1	157	52.1	-2.50	21.2	126	66	175	53	122.00	5.70	1	1.00	1	1	1	1	1	1	1	1	1	1	1
1	1	36	2	155	64.1	.00	26.8	128	70	191	64	127.00	5.20	1	1.00	1	0	1	1	1	0	1	0	1	1	1
0	0	70	1	166	68.0	-1.60	24.5	99	65	171	49	122.00	6.20	1	1.00	1	1	1	0	1	0	0	1	1	1	1
1	1	29	2	153	46.5	-3.00	19.7	105	61	148	77	71.00	5.10	0	1.00	0	0	1	0	0	0	0	1	1	1	1
1	1	23	2	159	63.1	-.20	24.8	100	60	197	72	125.00	4.50	1	1.00	1	1	1	1	1	1	1	1	1	1	1
0	0	80	1	166	79.9	2.50	29.1	126	72	177	65	112.00	5.90	1	1.00	0	1	1	1	1	1	1	0	0	1	1
0	1	70	2	150	51.6	1.40	22.8	132	69	217	51	166.00	5.60	1	1.00	1	0	1	0	1	0	0	1	1	1	1
0	0	44	2	162	61.4	8.80	23.5	128	72	181	68	113.00	5.20	1	1.00	1	1	1	1	1	0	0	1	1	1	1
1	1	76	2	145	38.8	1.70	18.5	116	73	196	54	142.00	5.50	1	1.00	1	0	1	1	1	1	1	1	1	1	1
1	1	67	2	150	52.4	.60	23.4	138	75	233	68	165.00	5.90	1	1.00	1	1	1	1	1	0	1	1	1	0	1
1	1	67	2	152	40.3	-.30	17.5	136	77	181	82	99.00	5.90	1	1.00	1	1	1	1	1	1	1	1	1	0	1
1	1	26	2	165	85.0	-6.20	31.3	122	75	234	55	179.00	13.30	1	1.00	1	1	0	0	1	0	0	0	1	0	0
1	1	68	1	174	69.0	-1.60	22.6	145	89	213	46	167.00	5.50	1	1.00	0	0	1	1	1	0	0	1	0	1	1
1	1	64	2	147	65.0	-2.80	30.0	124	72	173	55	118.00	9.60	1	1.00	1	1	1	1	0	0	0	0	0	1	0
1	1	78	1	156	59.7	.30	24.5	139	80	225	111	114.00	5.80	0	1.00	0	0	1	1	1	1	1	1	1	0	1
1	1	67	2	156	44.8	-.20	18.3	132	73	241	81	160.00	5.60	1	1.00	1	0	1	1	1	0	0	1	1	0	1
1	1	63	1	171	72.5	-7.20	24.9	131	83	247	58	189.00	6.90	1	1.00	1	0	1	0	0	1	1	1	1	0	0
1	1	74	2	138	50.3	-.60	26.3	137	80	179	56	123.00	5.60	1	1.00	1	1	1	1	1	1	1	0	1	1	1
0	0	41	2	152	43.5	1.10	18.7	100	59	195	79	116.00	5.60	1	1.00	1	0	0	0	1	0	0	1	1	1	1
1	1	70	2	151	43.5	.60	19.1	138	73	225	93	132.00	5.60	1	1.00	1	0	1	0	1	0	1	1	1	0	1
1	1	46	2	159	50.6	.70	19.9	106	69	197	88	109.00	5.00	1	1.00	0	1	1	1	1	1	1	1	1	1	1
1	1	47	2	156	57.2	2.10	23.6	118	71	170	73	97.00	5.30	1	1.00	1	0	1	1	1	1	0	1	1	1	1
0	0	70	2	150	60.9	-2.40	27.2	143	76	228	57	171.00	6.60	1	1.00	1	0	1	1	1	1	1	0	0	0	0
1	1	78	2	137	46.3	-.30	24.7	145	78	174	63	111.00	5.60	1	1.00	1	1	1	0	1	0	0	1	0	1	1

1	1	72	1	171	62.3	3.30	21.3	164	86	210	57	153.00	6.00	1	1.00	0	0	1	1	1	1	1	1	0	1	1
1	1	73	1	153	56.2	-1.00	24.1	140	89	185	66	119.00	5.40	1	1.00	0	0	1	1	1	1	1	1	0	1	1
1	1	71	2	151	52.8	.60	23.2	153	82	241	64	177.00	5.60	1	1.00	1	0	1	1	1	1	1	1	0	0	1
1	1	71	1	162	52.6	.70	20.1	134	71	170	61	109.00	5.40	0	1.00	0	0	1	0	1	0	1	1	1	1	1
1	1	78	1	156	56.9	1.10	23.4	128	79	231	48	183.00	5.90	0	2.00	1	0	1	0	1	1	1	1	1	0	1
1	1	68	2	148	47.8	-1.40	21.7	131	73	179	70	109.00	6.00	1	1.00	1	0	1	1	1	0	1	1	1	1	1
0	0	76	1	167	65.4	2.10	23.4	126	72	199	54	145.00	5.70	1	1.00	0	0	1	0	0	1	1	1	0	1	1
1	1	74	2	148	51.2	1.50	23.3	106	63	171	72	99.00	5.70	1	1.00	1	0	1	1	1	0	1	1	1	1	1
0	0	57	2	152	58.2	-1.00	25.3	146	82	164	56	108.00	5.70	1	1.00	1	1	1	1	1	1	1	0	0	1	1
0	0	43	2	161	80.4	-5.70	31.0	134	71	164	60	104.00	5.00	1	1.00	1	0	1	1	0	0	0	0	1	1	1
1	1	59	1	167	71.8	-2.70	25.8	120	75	241	49	192.00	5.50	0	1.00	1	0	1	0	1	1	1	0	1	0	1
1	1	48	1	174	62.7	.40	20.8	106	64	236	55	181.00	5.20	0	1.00	0	1	1	0	1	1	1	1	1	0	1
1	1	73	1	158	60.3	-2.80	24.2	122	74	196	52	144.00	5.80	1	1.00	0	0	1	1	1	1	0	1	0	1	1
1	1	69	2	143	46.1	.50	22.4	141	73	196	71	125.00	5.70	1	1.00	1	0	1	0	1	1	1	1	0	1	1
1	1	78	1	158	54.1	2.20	21.7	129	77	213	57	156.00	5.30	1	1.00	1	0	1	0	1	0	0	1	1	1	1
1	1	80	2	149	49.5	-1.00	22.4	152	78	168	59	109.00	5.70	1	1.00	1	0	1	1	1	1	1	1	0	1	1
0	0	44	2	154	55.1	1.90	23.3	126	69	165	83	82.00	5.00	1	1.00	1	0	1	0	1	1	1	1	1	1	1
1	1	55	2	148	46.9	1.80	21.3	128	67	210	102	108.00	5.10	1	1.00	1	0	1	0	1	0	0	1	1	1	1
1	1	69	1	166	70.0	-1.90	25.4	153	88	200	76	124.00	5.60	1	1.00	0	0	1	0	0	0	1	0	0	1	1
1	1	37	1	169	87.4	-1.80	30.4	135	72	243	66	177.00	5.60	1	1.00	0	0	0	0	1	1	1	0	1	0	1
1	1	51	1	162	67.4	-1.60	25.5	137	84	236	78	158.00	5.30	1	1.00	0	1	1	1	0	1	1	0	1	0	1
1	1	43	1	166	57.1	-6.70	20.6	113	68	223	59	164.00	5.80	0	1.00	0	1	1	0	1	1	1	1	1	0	1
1	1	75	2	134	39.6	.10	22.0	120	68	197	67	130.00	5.50	1	1.00	1	1	1	0	1	1	0	1	0	1	1
1	1	70	2	157	51.8	.90	21.1	133	73	145	62	83.00	5.80	1	1.00	1	0	1	1	1	0	1	1	1	1	1
0	0	69	1	162	81.5	-2.00	31.1	152	91	194	55	139.00	5.70	1	1.00	0	1	0	1	1	1	1	0	0	1	1
1	1	45	2	153	49.3	-.60	21.1	104	62	190	38	152.00	5.60	1	1.00	1	0	1	0	0	0	0	1	1	0	1
1	1	83	2	153	57.5	2.80	24.4	144	81	204	71	133.00	5.60	1	1.00	1	0	1	1	1	0	0	1	0	1	1
0	0	61	2	151	44.4	2.60	19.4	102	54	204	52	152.00	5.50	1	1.00	1	0	1	0	1	0	0	1	1	1	1
1	1	61	1	162	67.7	-.30	25.9	131	80	220	76	144.00	5.60	1	1.00	0	0	1	0	1	0	1	0	1	0	1
0	0	71	1	167	69.1	.90	24.7	126	73	171	53	118.00	5.70	1	1.00	1	0	1	1	1	1	1	1	0	0	1
1	1	53	2	152	51.0	.10	22.1	98	70	190	88	102.00	5.40	1	1.00	0	1	1	0	1	1	0	1	1	1	1
1	1	64	1	171	71.3	-2.80	24.5	121	69	152	55	97.00	8.10	1	1.00	0	1	1	0	1	1	1	1	0	1	0
1	1	64	1	171	55.7	-.20	18.9	148	88	168	87	81.00	4.90	0	1.00	0	0	1	0	1	1	0	1	0	1	1
0	0	31	2	163	76.4	3.10	28.8	128	69	152	49	103.00	5.10	1	1.00	1	1	1	1	1	1	1	0	1	1	1
0	0	33	1	170	51.4	4.80	17.8	101	66	267	68	199.00	5.50	0	1.00	1	0	0	0	1	0	0	1	1	0	1
0	0	45	1	167	59.3	-.10	21.1	134	96	209	114	95.00	5.40	0	1.00	0	1	1	0	1	1	0	1	0	1	1
1	1	28	2	158	46.0	-1.10	18.4	99	63	201	82	119.00	5.00	1	1.00	0	1	1	1	1	0	0	1	1	1	1
0	0	50	1	171	57.1	-4.90	19.4	127	78	165	58	107.00	5.30	1	1.00	1	1	1	0	1	1	1	1	1	1	1
1	1	61	2	153	61.3	-.70	26.1	158	85	194	68	126.00	5.20	1	1.00	1	0	1	1	1	0	1	0	0	1	1

1	1	68	2	156	63.0	-.70	25.8	133	82	192	65	127.00	5.70	1	1.00	1	1	1	1	1	1	0	0	1	0	1
0	0	39	2	163	60.7	.70	22.9	122	76	187	87	100.00	4.60	1	1.00	1	0	0	0	1	1	1	1	1	1	1
1	1	52	2	162	62.1	2.60	23.6	116	58	240	49	191.00	5.20	0	1.00	1	0	1	0	1	1	1	1	1	0	1
1	1	25	1	165	88.3	2.40	32.4	157	92	230	48	182.00	5.30	0	1.00	1	1	1	0	1	0	1	0	0	0	1
1	1	57	2	162	54.2	-.70	20.5	99	60	207	97	110.00	5.50	1	1.00	1	0	1	0	1	0	1	1	1	1	1
1	1	62	2	148	56.1	.00	25.5	117	69	161	58	103.00	5.60	1	1.00	1	0	1	0	1	1	1	0	0	0	1
1	1	63	1	166	59.6	-.30	21.6	152	92	191	88	103.00	5.10	1	1.00	0	0	1	0	1	1	0	1	0	1	1
1	1	74	2	140	55.2	.50	28.3	126	76	230	52	178.00	6.00	1	1.00	1	0	1	0	1	1	0	0	0	0	1
1	1	66	1	172	102.0	2.10	34.5	151	84	160	56	104.00	6.20	1	1.00	0	0	1	1	1	1	1	0	0	0	0
1	1	53	1	162	70.5	-3.00	26.9	149	92	267	61	206.00	6.60	1	1.00	0	0	1	1	1	1	1	0	0	0	0
1	1	80	2	135	42.7	-.20	23.6	145	77	237	72	165.00	5.50	1	1.00	1	1	1	0	1	0	1	1	0	0	1
1	1	75	2	157	62.4	-.90	25.1	122	68	220	52	168.00	5.20	1	1.00	1	0	1	1	1	0	0	0	0	0	1
1	1	65	2	153	60.5	-5.00	25.8	131	71	149	37	112.00	4.70	1	1.00	1	1	1	0	1	0	0	0	1	0	1
1	1	51	2	159	68.7	-4.60	27.3	112	64	179	101	78.00	4.90	1	1.00	1	0	1	0	0	0	1	0	1	1	1
1	1	73	2	152	63.5	2.00	27.6	146	79	224	74	150.00	5.30	1	1.00	1	0	1	0	1	0	1	0	0	0	1
1	1	66	1	164	67.6	-2.70	25.1	146	72	165	37	128.00	5.00	0	1.00	1	0	1	0	0	0	0	0	0	0	1
0	0	72	2	152	57.2	.10	24.9	143	91	227	48	179.00	6.30	1	1.00	1	0	1	1	1	1	1	1	0	0	1
0	0	69	1	159	60.6	-2.40	24.0	116	71	175	40	135.00	5.40	1	1.00	1	0	1	1	1	0	1	1	1	1	1
0	0	53	2	153	58.1	-3.20	24.7	121	70	223	67	156.00	5.60	1	1.00	1	0	1	1	0	0	1	1	0	0	1
1	1	39	1	162	68.6	-1.00	26.0	124	81	245	72	173.00	5.00	0	1.00	0	0	0	0	1	0	1	0	1	0	1
1	1	72	1	160	64.7	.80	25.4	166	91	202	59	143.00	5.40	1	1.00	1	0	1	0	1	0	1	0	0	1	1
0	0	23	1	175	70.5	3.40	23.0	118	71	186	63	123.00	5.40	1	1.00	1	0	0	0	1	1	0	1	1	1	1
0	0	68	2	152	60.9	-2.10	26.5	108	71	214	64	150.00	6.10	1	1.00	1	0	1	0	0	1	0	0	1	1	1
0	0	60	2	149	48.9	1.60	22.1	114	68	162	75	87.00	5.80	1	1.00	1	0	1	0	1	0	0	1	1	1	1
1	1	74	2	145	55.4	-3.60	26.2	121	68	208	68	140.00	5.70	1	1.00	1	0	1	1	1	0	1	0	0	1	1
1	1	58	2	155	64.3	-.50	26.7	110	69	251	55	196.00	5.20	1	1.00	1	0	0	1	1	1	1	0	1	0	1
0	0	56	2	148	60.2	5.60	27.4	132	86	226	55	171.00	5.70	1	1.00	1	1	1	1	0	1	1	0	1	0	1
1	1	46	2	149	46.3	-1.10	20.7	101	56	224	94	130.00	5.20	1	1.00	0	0	1	0	1	1	1	1	1	0	1
1	1	35	2	149	46.0	-1.90	20.6	120	64	181	81	100.00	5.10	1	1.00	1	0	1	1	1	1	1	1	1	1	1
1	1	59	2	158	64.0	2.50	25.7	106	61	208	67	141.00	5.00	1	1.00	1	0	1	0	1	1	1	0	1	1	1
1	1	80	2	147	57.7	-1.00	26.5	149	83	179	57	122.00	6.00	1	1.00	1	0	1	1	1	0	1	0	0	0	1
1	1	71	1	164	64.9	-1.80	24.1	141	67	176	86	90.00	5.70	0	1.00	0	0	1	0	1	1	1	1	0	1	1
1	1	24	2	150	58.0	-3.90	25.7	124	71	209	86	123.00	5.10	1	1.00	1	1	1	0	1	1	1	0	1	1	1
1	1	50	2	170	75.5	-2.40	26.2	130	76	189	89	100.00	5.20	1	1.00	0	1	1	1	1	1	1	0	1	1	1
0	0	40	1	177	69.2	-1.50	22.0	103	62	175	41	134.00	5.20	0	1.00	1	1	1	0	1	1	1	1	1	1	1
1	1	72	1	165	60.4	-6.20	22.2	120	69	183	93	90.00	5.40	1	1.00	1	0	1	0	1	1	1	1	1	1	1
0	0	45	2	152	55.0	.50	23.7	123	70	184	77	107.00	5.30	0	1.00	1	1	1	1	1	1	1	1	1	1	1
1	1	46	2	154	68.7	-2.80	29.1	138	78	212	74	138.00	7.30	1	1.00	1	0	1	1	1	1	1	0	1	1	0
1	1	39	2	162	49.0	-4.90	18.7	103	62	170	79	91.00	5.20	1	1.00	1	1	1	0	1	1	0	1	1	1	1

0	0	82	1	170	80.7	-3.30	27.8	130	75	178	39	139.00	6.40	1	1.00	1	0	1	0	0	1	1	0	1	0	1
0	0	73	1	169	60.3	-2.20	21.1	110	64	207	83	124.00	5.60	0	1.00	0	0	1	0	1	1	1	1	1	1	1
0	0	44	2	160	46.7	1.60	18.1	99	58	240	97	143.00	5.60	1	1.00	1	0	1	1	1	1	1	1	1	0	1
1	1	68	2	144	53.4	-3.40	25.6	116	64	236	45	191.00	6.10	1	1.00	1	0	1	1	1	0	1	0	1	0	1
1	1	49	2	154	44.7	1.00	18.8	125	65	185	74	111.00	5.00	1	1.00	1	0	1	0	1	1	1	1	1	1	1
1	1	79	2	141	31.7	-1.40	15.9	144	83	158	67	91.00	5.80	1	1.00	1	0	1	1	1	1	1	1	0	0	1
1	1	69	2	151	56.1	-1.00	24.5	134	73	199	47	152.00	5.80	1	1.00	1	0	1	1	0	1	1	1	0	1	1
0	0	50	2	155	58.9	-4.10	24.4	92	62	190	90	100.00	5.80	1	1.00	1	0	1	0	0	0	0	1	1	1	1
1	1	61	1	165	73.9	-1.80	27.2	117	71	174	44	130.00	5.30	1	1.00	0	1	1	0	1	1	1	0	0	1	1
1	1	55	1	162	68.0	-.30	26.0	131	87	227	66	161.00	5.40	1	1.00	0	0	1	0	1	0	0	0	1	0	1
0	0	68	1	164	74.3	1.20	27.5	148	97	189	35	154.00	5.90	0	1.00	0	1	1	1	0	1	1	0	0	0	1
1	1	63	2	148	49.0	.80	22.5	154	87	244	67	177.00	5.80	1	1.00	1	1	1	0	1	1	1	1	0	0	1
0	0	54	2	151	50.0	-.80	21.8	158	90	185	75	110.00	5.30	1	1.00	1	0	1	0	1	0	1	1	0	1	1
0	0	78	1	160	55.1	-.30	21.4	139	82	203	77	126.00	5.20	1	1.00	1	0	1	0	1	0	0	1	1	1	1
1	1	34	2	161	56.9	-.20	22.0	111	73	194	65	129.00	5.20	1	1.00	1	0	1	0	1	1	1	1	1	1	1
1	1	76	1	164	68.3	.70	25.3	126	71	116	66	50.00	5.00	1	1.00	0	0	0	1	1	1	1	0	0	1	1
1	1	81	1	156	57.1	1.30	23.5	125	61	102	31	71.00	6.20	1	1.00	1	0	1	0	1	0	0	1	0	0	1
0	0	71	2	148	53.9	-.50	24.6	126	78	201	66	135.00	5.50	1	1.00	1	0	1	0	1	1	0	1	0	1	1
0	0	44	2	158	48.6	-.10	19.4	94	62	160	54	106.00	5.40	1	1.00	1	0	1	1	1	1	1	1	1	1	1
0	0	72	2	159	63.4	.80	24.9	147	75	224	63	161.00	5.40	1	1.00	1	0	1	1	1	1	1	1	0	0	1
1	1	82	1	171	69.4	-4.80	23.8	137	75	179	56	123.00	6.20	1	1.00	1	0	1	1	1	1	1	1	1	1	1
1	1	70	1	154	51.7	-1.70	21.7	121	69	147	51	96.00	6.00	0	1.00	1	1	1	0	0	0	1	1	1	1	1
1	1	61	2	151	57.9	.60	25.4	130	74	187	62	125.00	5.30	1	1.00	1	0	1	0	0	1	1	0	1	1	1
1	1	66	2	153	66.6	-.30	28.4	147	76	161	57	104.00	6.30	1	1.00	1	0	1	1	1	1	0	0	0	1	1
1	1	37	2	163	55.8	2.00	21.0	147	80	142	60	82.00	5.10	1	1.00	1	1	1	0	1	0	1	1	0	1	1
0	0	75	2	149	47.8	1.20	21.6	121	78	168	89	79.00	5.90	1	1.00	1	0	1	0	1	1	1	1	0	0	1
1	1	72	2	148	49.6	-4.70	22.7	126	76	199	62	137.00	6.20	1	1.00	1	0	1	1	1	1	1	1	1	1	1
1	1	48	2	165	63.1	-1.70	23.1	126	69	224	62	162.00	5.20	1	1.00	1	0	1	0	1	1	1	1	1	0	1
1	1	63	2	160	48.9	.40	19.0	135	74	196	74	122.00	5.80	1	1.00	1	0	1	1	1	1	0	1	1	1	1
0	0	75	1	164	60.3	-2.10	22.3	112	76	153	70	83.00	5.50	0	1.00	0	0	1	1	1	0	1	1	0	1	1
1	1	70	2	150	48.3	-1.00	21.5	122	72	244	70	174.00	5.80	1	1.00	1	1	1	1	1	0	1	1	1	0	1
1	1	73	2	149	45.3	-.50	20.4	112	63	174	71	103.00	5.70	1	1.00	1	0	1	1	1	1	0	1	0	1	1
1	1	51	2	155	45.4	.20	18.8	101	64	288	117	171.00	5.30	1	1.00	0	1	1	1	1	1	1	1	1	0	1
1	1	48	2	160	56.3	-3.30	22.0	136	78	263	69	194.00	5.90	1	1.00	1	0	1	1	1	1	1	1	1	0	1
1	1	55	1	174	73.7	.30	24.3	148	104	237	53	184.00	5.40	1	1.00	0	0	1	0	1	0	1	1	0	0	1
1	1	83	1	155	53.6	-1.70	22.2	88	58	190	70	120.00	7.90	1	1.00	1	0	1	1	1	1	0	1	0	1	0
1	1	56	2	144	44.2	1.90	21.4	131	73	235	54	181.00	5.30	1	1.00	1	0	1	1	1	0	1	1	1	0	1
0	0	57	2	162	58.9	-2.30	22.4	116	78	190	75	115.00	6.00	1	1.00	1	0	1	0	1	0	1	1	1	1	1
1	1	56	2	163	53.7	-1.50	20.3	135	82	223	90	133.00	5.70	1	1.00	1	1	1	1	1	1	1	1	1	0	1

0	0	68	2	158	53.6	2.00	21.4	128	71	238	37	201.00	6.30	1	1.00	1	0	1	1	1	1	1	1	1	0	1
1	1	46	2	165	58.4	-.30	21.3	120	81	178	66	112.00	5.70	1	1.00	1	0	1	0	1	1	1	1	1	1	1
0	0	68	1	168	59.4	4.10	20.9	129	75	205	85	120.00	5.10	0	1.00	0	0	1	1	1	0	1	1	1	1	1
1	1	61	2	152	46.4	.90	20.1	117	73	204	85	119.00	5.40	1	1.00	1	0	1	1	1	1	0	1	1	0	1
1	1	48	2	165	69.0	-2.60	25.3	130	78	253	91	162.00	5.50	1	1.00	1	0	1	1	1	1	1	0	1	0	1
1	1	45	2	164	53.1	-.30	19.6	107	66	202	87	115.00	5.40	1	1.00	0	0	1	1	1	1	1	1	1	1	1
1	1	39	2	158	41.9	-1.40	16.8	112	70	191	81	110.00	5.40	0	1.00	1	0	1	0	1	1	0	1	1	1	1
1	1	70	2	157	56.1	-5.10	22.7	152	84	249	67	182.00	5.90	1	1.00	1	0	1	1	1	0	1	1	0	0	1
1	1	47	2	156	49.5	-1.00	20.3	126	74	230	82	148.00	5.40	1	1.00	1	0	0	0	0	0	0	1	1	0	1
1	1	62	2	157	54.3	.20	22.1	105	56	151	55	96.00	5.90	1	1.00	1	0	1	0	0	0	0	1	0	1	0
1	1	35	1	177	67.2	-.80	21.3	107	62	278	99	179.00	6.60	1	1.00	1	0	1	1	1	1	1	1	1	0	0
1	1	30	2	151	76.1	-.20	33.1	125	72	232	72	160.00	5.80	1	1.00	1	0	1	0	1	1	1	0	1	0	1
1	1	71	1	172	72.8	-2.90	24.7	140	84	236	84	152.00	5.90	1	1.00	0	0	1	0	1	0	0	1	0	0	1

Explanation of variables in the data of present study.

Variables	Variable labels	Value labels
LP_2011_d	Living condition in 2011	0, a temporary housing group; 1, not temporary housing group
LP_2012_d	Living condition in 2012	0, a temporary housing group; 1, not temporary housing group
age	Age	
sex	Sex	1, males; 2, females
height	Height (cm)	
weight	Body weight (kg)	
dweight	The mean change in body weight from 2011 to 2012	
BMI	Body mass index (kg/m^2)	
sbp	Systolic blood pressure (mmHg)	
dbp	Diastolic blood pressure (mmHg)	
TC	Total cholesterol (mg/dl)	
HDL	High-density lipoprotein cholesterol (mg/dl)	
nonHDLC	Non high-density lipoprotein cholesterol (mg/dl)	
Hba1c_N	Glycemic hemoglobin; HbA1c (NGSP) (%)	
smoke_ph1	Smoking status (2011)	0, current smokers; 1, not current smokers
R_quitSM	Quitting smoking	0, quitting smoking; 1, not quitting smoking
drink_ph1	Drinkers (2011)	0, drinkers; 1, non-drinkers
pas	Physical activity (2011)	0, low physical activity; 1, normal physical activity
q3_1c	The average number of meals per day (2011)	0, small number of meals (< 3 times); 1, normal number of meals ($\geq$ 3 times)
q13c	Economic status (2011)	0, poor economic status; 1, normal
q5_2_c	Occupational status (2011)	0, unemployed; 1, not unemployed
k6c	Psychological distress (2011)	0, psychological distress; 1, no psychological distress
aisc	Insomnia (2011)	0, insomnia; 1, no insomnia
obesity	Overweight (2011)	0, overweight; 1, non overweight
R_t1ht	Hypertension (2011)	0, hypertension; 1, non hypertension
R_t1lipid_o	Dyslipidemia (2011)	0, dyslipidemia; 1, non dyslipidemia
t1dmt_i	Diabetes mellitus (2011)	0, diabetes mellitus; 1, non diabetes mellitus
